# Genome-Wide Transcriptome Analysis Reveals the Comprehensive Response of Two Susceptible Poplar Sections to *Marssonina brunnea* Infection

**DOI:** 10.3390/genes9030154

**Published:** 2018-03-12

**Authors:** Yanfeng Zhang, Longyan Tian, Dong-Hui Yan, Wei He

**Affiliations:** 1 The Key Open Laboratory of Forest Protection affiliated to State Forestry Administration of China, Research Institute of Forest Ecology, Environment and Protection, Chinese Academy of Forestry, Beijing 100091, China; zhangyanfeng@bjfu.edu.cn; 2 Beijing Key Laboratory for Forest Pest Control, Beijing Forestry University, Beijing 100083, China; tianlongyan@bjfu.edu.cn

**Keywords:** poplar, *Marssonina* leaf spot fisease of poplar, *Marssonina brunnea*, transcriptome, differentially expressed genes, response, interaction

## Abstract

*Marssonina* leaf spot disease of poplar (MLDP), caused by the hemibiotrophic pathogen *Marssonina brunnea*, frequently results in damage to many poplar species. In nature, two formae speciales of *M. brunnea* exist that are susceptible to different poplar subgenera. *Marssonina brunnea* f. sp. *monogermtubi* infects poplar hosts from *Populus* sect. *Aigeiros* (Aig), while *M. brunnea* f. sp. *multigermtubi* always infects poplar hosts from *Populus* sect. *Leuce Duby* (Leu). Based on the fungal penetration structures, a comprehensive transcriptomic approach was used to investigate the gene expression patterns of these two poplar subgenera at three crucial infection stages. MLDP significantly altered the expression patterns of many genes involved in mitogen activated protein kinase (MAPKs) and calcium signaling, transcription factors, primary and secondary metabolism, and other processes in both poplar subgenera. However, major differences in gene expression were also observed between the two poplar subgenera. Aig was most responsive at the initial infection stage, while Leu largely interacted with *M. brunnea* at the necrotrophic phase. Furthermore, the differentially expressed genes (DEGs) involved in pathways related to biotic stress also differed substantially between the two poplar subgenera. Further analysis indicated that the genes involved in cell wall metabolism and phenylpropanoid metabolism were differentially expressed in the progression of the disease. By examining the expression patterns of genes related to the defense against disease, we found that several genes annotated with causing hypersensitive cell death were upregulated at the necrotrophic phase of MLDP, inferring that plant immune response potentially happened at this infection stage. The present research elucidated the potential molecular differences between the two susceptible interaction systems in MLDP and provided novel insight into the temporal regulation of genes during the susceptible response. To the best of our knowledge, this study also constitutes the first to reveal the molecular mechanisms of poplar in response to the transition of hemibiotrophic fungal pathogens from the biotrophic phase to the necrotrophic phase.

## 1. Introduction

Plant–pathogen interaction networks are complex, and long-term co-evolution has resulted in the establishment of two primary means of communication between plants and their pathogens. Plants attempt to recognize pathogens and defend themselves against potential pathogens on their surface, while pathogens, in contrast, endeavor to manipulate the biology of the plant to complete their growth and reproduction [1]. Understanding the molecular mechanisms of plant–pathogen interactions is crucial for elucidating disease development. Many studies have detailed the types of plant molecular defense mechanisms in response to unsusceptible pathogens [2]. However, investigations into the pathogenesis between susceptible plants and pathogens could help develop models of plant disease as well as provide information regarding susceptible genes, which would facilitate the prediction of pathogen and host fitness in varying environments and inform resistance breeding programs [3]. Importantly, the elucidation of disease resistance requires an understanding of susceptibility.

Poplars (*Populus* spp.) are important commercial tree species that are globally distributed and have become the model trees for forests [4]. The genus *Populus* comprises six sections: *Abaso*, *Leuce*, *Leucoides*, *Aigeiros*, *Turanga*, and *Tacamahaca* [5]. Most poplar species tolerate a variety of diseases throughout their lifespan, and many pathogens can seriously impact wood production and even cause death in poplars. *Marssonina* leaf disease of poplar (MLDP), which mostly causes early defoliation, is one of the main diseases affecting poplar [6]. The average loss to wood production attributable to MLDP could reach 30% annually [7]. Considering that the area of poplar plantations exceeds 8.5 million ha in China [8], research on this disease has become more necessary. Previous studies of MLDP have described the disease symptomatology [9,10] and the physiological responses of poplars to MLDP have also been reported. As MLDP progresses, the concentration of ozone might accelerate the disease on older leaves and might increase the resistance to *Marssonina* spp. on the young leaves [11]. Erickson et al. noted declines in leaf photosynthesis as a result of a disruption to photosynthesis, including reduced leaf stomatal conductance [12]. At the molecular level, several secreted proteins, including potential effectors, were identified in *Marssonina brunnea* [13]. For poplar hosts, the expression of two *Populus deltoids* lipoxygenase genes (*PdLOX1* and *PdLOX2*), and two extracellular proteins, *PdPGIP2 and PdPGIP4*, were both upregulated under the penetration of *M. brunnea* [14,15]. Additionally, *PtrWRKY* genes contained in the complete WRKY domain in *Populus* spp. were also induced during MLDP development [16]. Using microarray hybridization, a previous study discovered that 1160 poplar genes involved in categories such as metabolism were found to be induced in MLDP [17]. However, most of these studies were focused on special genes and lacked complete molecular data on this disease.

In China, MLDP is mainly caused by *M. brunnea*, which belongs to the family Dermateaceae [18]. Reports indicate that *M. brunnea* has two formae speciales: *M. brunnea* f. sp. *monogermtubi* and *M. brunnea* f. sp. *multigermtubi* [10,18,19]. In nature, *M. brunnea* f. sp. *monogermtubi* infects poplar hosts from *Populus* sect. *Aigeiros* (Aig), while *M. brunnea* f. sp. *multigermtubi* always infects poplar hosts from *Populus* sect. *Leuce* Duby (Leu) [10,19]. It is worth emphasizing that the pathogens and hosts in different susceptible pathogenetic systems are normally incompatible [10,18,19]. The specificity of *M. brunnea* towards Aig and Leu implies complex interactions between plants and pathogens. Transcriptome analysis of *M. brunnea* f. sp. *multigermtubi* on the leaves of resistant and susceptible poplar has been conducted [20]. In previous studies, we elucidated the complex infection process of MLDP and verified that *M. brunnea* is a typical hemibiotrophic pathogen [21]. All these studies inform the research of MLDP. Although some molecular information exists regarding the interactions of MLDP, present analyses on the function of specific genes and the transcriptome have all focused on MLDP caused by *M. brunnea* f. sp. *multigermtubi*. An assessment of the interactions between *M. brunnea* f. sp. *monogermtubi* and Leu is still lacking and limits the understanding of the differences between the MLDPs caused by the two formae speciales. Additionally, it is worth noting that previous studies were all designed and analyzed without considering the concrete microscopic histopathology and hemibiotrophic traits of *M. brunnea*. Additional research is required to systematically recognize and analyze MLDP more accurately.

The aims of the current study were three-fold: (i) to identify poplar genes that are differentially expressed in response to MLDP in two poplar sections; (ii) to explore crucial genes associated with the development of MLDP and further analyze the physiological and biochemical changes caused by pathogenic infection of *M. brunnea* in poplar; and (iii) to elucidate the potential different molecular response mechanisms of the two poplar sections to MLDP. To achieve this, RNA-Seq analysis was used to analyze the transcription profiles of 31,952 poplar genes and global patterns in poplar gene expression within two types of MLDP, *M. brunnea* f. sp. *multigermtubi* infecting Aig, and *M. brunnea* f. sp. *monogermtubi* infecting Leu, following three important infection stages. This enabled the identification of significantly differentially expressed genes (DEGs) during disease development and revealed the major metabolic processes and signal pathways in poplar associated with the development of MLDP. These results are important for understanding the molecular mechanisms of MLDP pathogenesis and indirectly inform breeding programs.

## 2. Materials and Methods

### 2.1. Host and Pathogen

The cultivars of poplar used were *Populus deltoids* cv. *Zj-2* clones belonging to Aig, and *Populus alba* × *Populus alba* var. *pyramidalis* clones belonging to Leu. They were both obtained from the poplar seedling station of the Poplar Experimental Bureau (Datong, Shanxi Province, China). Annual poplar cuttings were cultured in a glasshouse maintained at 25 ± 2 °C under a relative humidity (RH) of 50–55% during the day, and at 22 ± 2 °C under 55–65% RH at night. Light during the day was supplied from 08:00 to 20:00 by sunlight lamps. The studied fungi were *M. brunnea* f. sp. *monogermtubi* (MO45) isolated from *Populus tomentosa* in Beijing, and *M. brunnea* f. sp. *multigermtubi* (MU39) isolated from *Populus nigra* (Hsienyang, Shaanxi Province, China). During isolation, the infected leaves were first washed by sterile water, sterilized about 30 s in 75% ethanol, and then kept in moist conditions for 2 days at 25 °C for developing fruit bodies. When the white conidia were produced, they were transferred onto potato-dextrose agar (PDA) containing 50 µg/mL streptomycin sulfate using an inoculating needle. Finally, the isolates were cultured on PDA medium at 25 °C in the dark and then stored in tubes filled with PDA inclined medium at 4 °C. 

### 2.2. Microscopy

The strain method was used to visualize the MLDP infection process by microscopy. Briefly, the inoculated leaves were collected and cut into 1 × 1 cm pieces. After de-coloring for 12 h in 0.15% saturated trichloroacetic acid bleaching liquid (solvent, 3:1 alcohol:chloroform solution), the samples were transferred into a mixed stain solution (saturated chloral hydrate and aniline blue (5:1)) and stained for nearly 4 h at 58 °C. The samples were then observed under a light microscope (Leica DM2500, Leica Microsystems, Wetzlar, Germany).

### 2.3. Inoculation and Sample Collection

The inoculum was obtained by washing the conidia from the incubated leaves with sterile distilled water and then adjusting the concentrations to approximately 5 × 10^5^ conidia/mL. Fully expanded healthy leaves were collected from the poplar seedlings, sterilized in 75% ethanol for 20 s, washed with sterile water three times, and then placed on wet filter paper upon a layer of water agar medium in Petri dishes. The spore suspension was sprayed onto the surface of the leaves, and the inoculated leaves were incubated in a manual climatic incubator at 25 °C and 100% RH under a 12 h photoperiod (08:00 to 20:00) using sunlight lamps. For observation, the inoculated leaves were harvested at six h post inoculation (hpi), 36 hpi, and 96 hpi. In the control group (CK), the leaves were sprayed with distilled water and harvested at six hpi. The samples were then immediately frozen in liquid nitrogen and stored at −80 °C for RNA isolation. Inoculations on detached leaves have proven to be available for the research of MLDP [10,13,20,22].

### 2.4. RNA Isolation and Sequencing

The RNA extraction of the samples used TRIzol reagent (Invitrogen, Carlsbad, CA, USA) according to the instruction manual. The quality of all RNA was confirmed by agarose gel electrophoresis and determined with a NanoDrop 2000 spectrophotometer (Thermo Scientific, Vilnius, Lithuania). The mRNA was isolated by NEBNext Poly (A) mRNA magnetic isolation module (New England Biolabs, Ipswich, MA, USA). The complementary DNA (cDNA) library was constructed following the manufacturer instructions of the NEBNext ultra RNA library prep kit for Illumina (New England Biolabs) and NEBNext Multiplex Oligos for Illumina (New England Biolabs). Briefly, the enriched mRNA was fragmented into approximately 200 nt RNA inserts, which were used to synthesize the first-strand cDNA and the second-strand cDNA. The double-stranded cDNA was synthesized via end-repair/dAdenosine-tail and adaptor ligation. The suitable fragments were isolated using Agencourt AMPure XP beads (Beckman Coulter, Inc., Brea, CA, USA) and enriched by PCR amplification. Finally, the constructed cDNA libraries were sequenced on a flow cell using an Illumina HiSeq™ 2500 sequencing platform (Illumina; San Diego, CA, USA). The RNA-seq data are available from the National Center for Biotechnology Information (NCBI) database with the BioProject number PRJNA397718. 

### 2.5. Reads Mapping

Low quality reads were filtered by excluding those with only adaptors, and unknown nucleotides were removed. The clean reads filtered from the raw reads were mapped to the v3.0 genome of *Populus trichocarpa*, downloaded from Phytozome [23] using Tophat2 package v2.0.9 software [24] with some modified parameters (read-mismatches: 4; read-edit-dist: 4; max-intron-length: 5,000,000; library-type: fr-unstranded; num-threads: 8; mate-inner-dist: 40) and other options default, which allows multiple alignments per read and a maximum of four mismatches when mapping the reads to the reference genome. The aligned records from the alignments in BAM/SAM format were further examined to remove potential duplicate molecules. Transcripts were assembled using Cufflinks version 2.2.1 [25], which was used to evaluate transcript expression by the normalized RNA-seq fragment counts to measure the relative abundances of the transcripts with default parameters. Following normalization, gene counts in each sample were recorded, and expression levels were obtained. The fragments per kilobase of exon per million fragments mapped (FPKM) value was used for estimating gene expression levels.

### 2.6. Differentially Expressed Genes Analysis and Gene Function Annotation

For the two poplar sections, DEGs were analyzed using DESeq2 [26] in R package using three replicates per sample. A false discovery rate (FDR) *p*-value less than 0.001 and a threshold fold change (log_2_) ≥ 2 was used to call differentially expressed genes between two samples. The identification of unique or overlapping genes within the different expression data of the samples and the generation of Venn diagrams were constructed using Draw Venn Diagram [27]. Heat maps and hierarchical clustering analyses were produced in Multiple Experiment Viewer (MeV) version 4.9.0 [28]. Common expression trend analysis was based on STEM software [29] using the default parameters.

Gene Ontology (GO) term annotation and GO enrichment for poplar genes were obtained by online analysis on agriGO version 2.0 [30] with *P. trichocarpa* as the reference set. The GO enrichment was completed under a significance threshold of 0.05 following Hochberg FDR correction. The enriched GO terms were then summarized using the online analysis tool REVIGO [31]. Kyoto Encyclopedia of Genes and Genomes (KEGG) enrichment was performed using the R [32] package *clusterProfile* [33] and visualized using the R package *Pathview* [34]. Additionally, the gene enrichment of DEGs in biotic stress pathway was visualized using MapMan [35].

### 2.7. Quantitative Reverse-Transcription PCR

RNA samples were firstly purified with DNase I (RNase-free) (TaKaRa, Dalian, China). Quantitative reverse-transcription (qRT)-PCR was performed with Oligo-DT and SuperScript III reverse transcriptase (Invitrogen). All qRT-PCR reactions were performed with SuperReal Premix Plus (SYBR green kits; TIANGEN, Beijing, China) and carried out on an ABI 7500 real-time PCR system (Applied Biosystems, Foster City, CA, USA). Relative expression levels were calculated using the ΔΔCT method [36], with 60S [37] used as the internal control. All primers used in this study are listed in Table S1.

## 3. Results

### 3.1. The Disease Development of Leaf Spot Disease of Poplar

**Figure 1:**
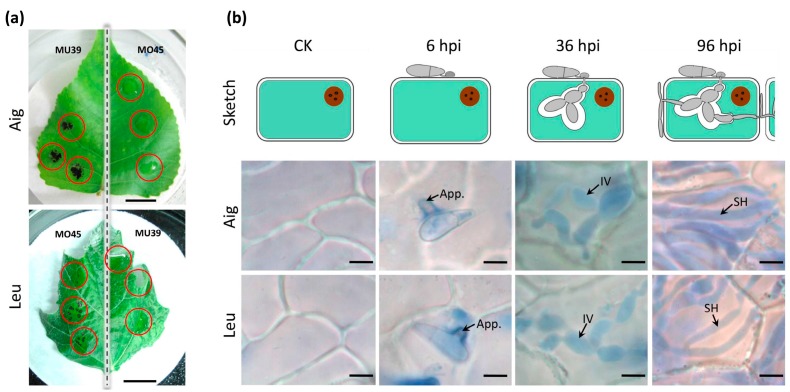
Poplar leaves infected by *Marssonina brunnea*, and the penetration process of MLDP. (**a**) Aigeiros (Aig) is susceptible to *M. brunnea* f. sp. *multigermtubi* (MU39) and resistant to *M. brunnea* f. sp. *monogermtubi* (MO45). Leu is susceptible to MO45 and resistant to MU39. Scale bar = 1 cm. (**b**) At six hours post inoculation (hpi), appressorium (App) started to appear on the surface of plant cell. At 36 hpi, infective vesicles (IV) appeared within epidemic cells. At 96 hpi, secondary hyphae (SH) appeared. Scale bar = 5 μm.

The susceptible and resistant systems of MLDP were identified based on the inoculation experiment. Specifically, Aig was susceptible to MU39 and resistant to MO45, while Leu was susceptible to MO45 and resistant to MU39 (Figure 1a). Histopathology assessment confirmed three infection stages of the disease. At six hpi, appressoria had developed on the surface of the poplar leaves (Figure 1b). At 36 hpi, infective vesicles (IV) had developed in the host cells (Figure 1b), and the pathogens were in the biotrophic phase. At 96 hpi, secondary hyphae (SH) appeared (Figure 1b), and the pathogens began to transform into the necrotrophic phase. Transcriptome experiments were conducted according to the three disease developmental stages. 

### 3.2. Summary of RNA Sequencing and Assembly

**Figure 2:**
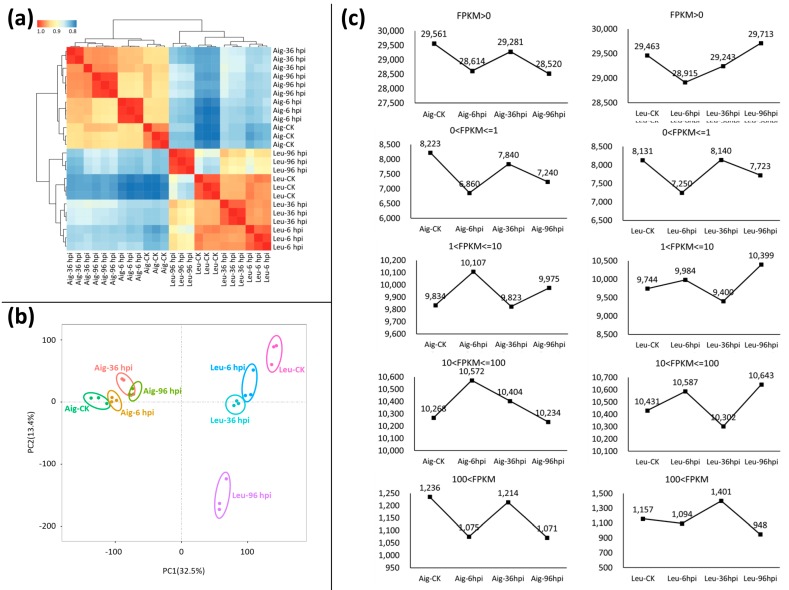
Correlation among RNA-seq samples, and distribution of gene expression values in two poplars. (**a**) Heat map of the Pearson correlation of sequenced samples based on gene expression level. Clustering was conducted according to the expression data of all expressed genes. (**b**) Principal component analysis (PCA) of transcript levels of RNA-seq samples. The analysis was performed on R using the expression data of all expressed genes. (**c**) Distribution of gene expression values among developmental stages examined. The FPKM value of each gene was calculated by the average of FPKM values of three biological replicates. FPKM: fragments per kilobase of exon per million fragments mapped.

In total, 248.79 GB clean data were obtained from the RNA-Seq of the 24 samples. The clean data of each sample exceeded 5.58 GB. The reads of an average quality score greater than 30 (Q 30%) value of the samples all exceeded 85%. The percentage of mapped reads across all samples to the genome of *P. trichocarpa* ranged from 50.58% to 73.55% (Table 1). Among these mapping ratios, the samples of Aig had higher values ranging from 67.2% to 73.55%, while the Leu samples had lower values ranging from 50.58% to 59.39% (Table 1). The correlation indexes between the replicated samples and the same treatment always exceeded 0.9 and were higher than the values between the different treatment samples (Figure 2a). A similar result was obtained in the principle components analysis (PCA) (Figure 2b). Replicated samples grouped together, and the Aig and Leu samples respectively clustered together (Figure 2b). The distribution of samples in Leu was relatively scattered, suggesting large changes at the gene expression level in these samples (Figure 2b).

From 28,520 to 29,561 genes were expressed at the level (0 < FPKM) in Aig, while from 28,915 to 29,713 genes were noted in Leu (Figure 2c). In general, the expression patterns of the three levels (0 < FPKM ≤ 1, 1 < FPKM ≤ 10, and 100 < FPKM) were similar for Aig and Leu (Figure 2c). In the classes (0 < FPKM ≤ 1 and 100 < FPKM), the samples of CK and 36 hpi in the two poplars both involved more genes, while the samples at six hpi had the fewest genes (Figure 2c). In the class (100 < FPKM), the majority of genes were detected in the samples at 36 hpi in the two poplars following penetration (Figure 2c). In the class (1 < FPKM ≤ 10), the gene expression of samples at six hpi and 96 hpi was highest (Figure 2c). The expression patterns at the level (10 < FPKM ≤ 100) differed between the two poplars (Figure 2c). In this class, the samples at six hpi in Aig exhibited the highest number of genes (10,572), which decreased along with the progression of the disease. Conversely, the highest numbers of genes were present at six hpi and 96 hpi in Leu (Figure 2c).

To validate the expression patterns identified by the RNA-Seq, 18 common genes with different expression levels were selected to perform qRT-PCR analysis for Aig and Leu (Figure S1). Analysis of the RNA-Seq and qRT-PCR datasets substantiated the expression results generated by the RNA-Seq.

### 3.3. Analysis of Differentially Expressed Genes

**Figure 3:**
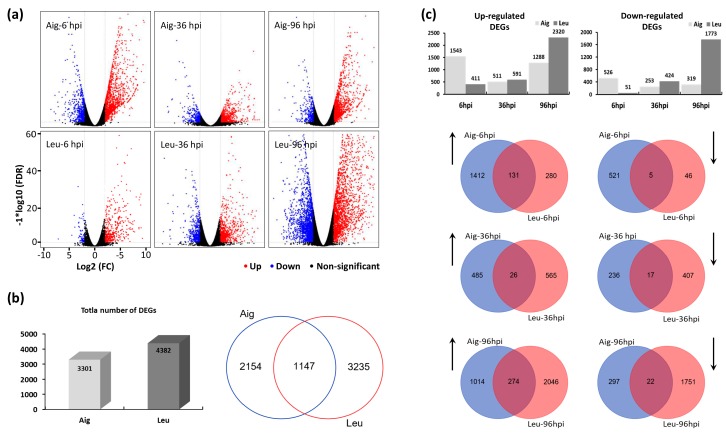
The number of differentially expressed genes (DEGs) in infection stages of two poplars. (**a**) Volcano Plot shows the number upregulated and downregulated DEGs in different stages of two poplars. (**b**) Histogram shows the number of all DEGs detected in two poplars. Venn diagram shows the overlapping of DEGs detected in two poplars. (**c**) Histogram shows the up or downregulated DGEs of poplars in different infection stages. Venn diagram shows the overlapping of up or downregulated DEGs between two poplar sections at three infection stages. Arrows indicate up or downregulation.

The volcano plot indicated that the expression patterns of the significantly regulated genes in the two poplar clones differed. The expression levels of the samples at six hpi and 96 hpi were higher than at 36 hpi in Aig (Figure 3a). However, a different expression pattern was observed in Leu, with the lowest expression level occurring at six hpi and the number of significantly regulated genes increasing as the disease progressed (Figure 3a). A total of 3301 genes in Aig and 4382 genes in Leu exhibited differential regulation following inoculation with *M. brunnea* (Figure 3b). The Venn diagram showed that 1147 genes were shared between the two poplar clones (Figure 3b). However, when analyzing the gene regulation patterns at the same infection stages, the number of overlapping genes was lower (Figure 3c). These results implied that the responses of the two poplars to *M. brunnea* might differ. Additionally, no common genes were invariably found to be downregulated during pathogen infection between the two poplar clones (Figure S2). However, five genes, including the Leucine-rich repeat transmembrane protein kinase (Potri.005G043700), the S-locus lectin protein kinase family protein (Potri.004G027400), the protein kinase Potri.012G054700, the Pectin methylesterase inhibitor superfamily (Potri.002G202600) and the Phosphate translocator 2 (Potri.004G019900) were always upregulated upon *M. brunnea* penetration in both Aig and Leu (Figure S2). These five genes might be important for the development of MLDP. 

#### 3.3.1. GO Enrichment Analysis of Differentially Expressed Genes

**Figure 4:**
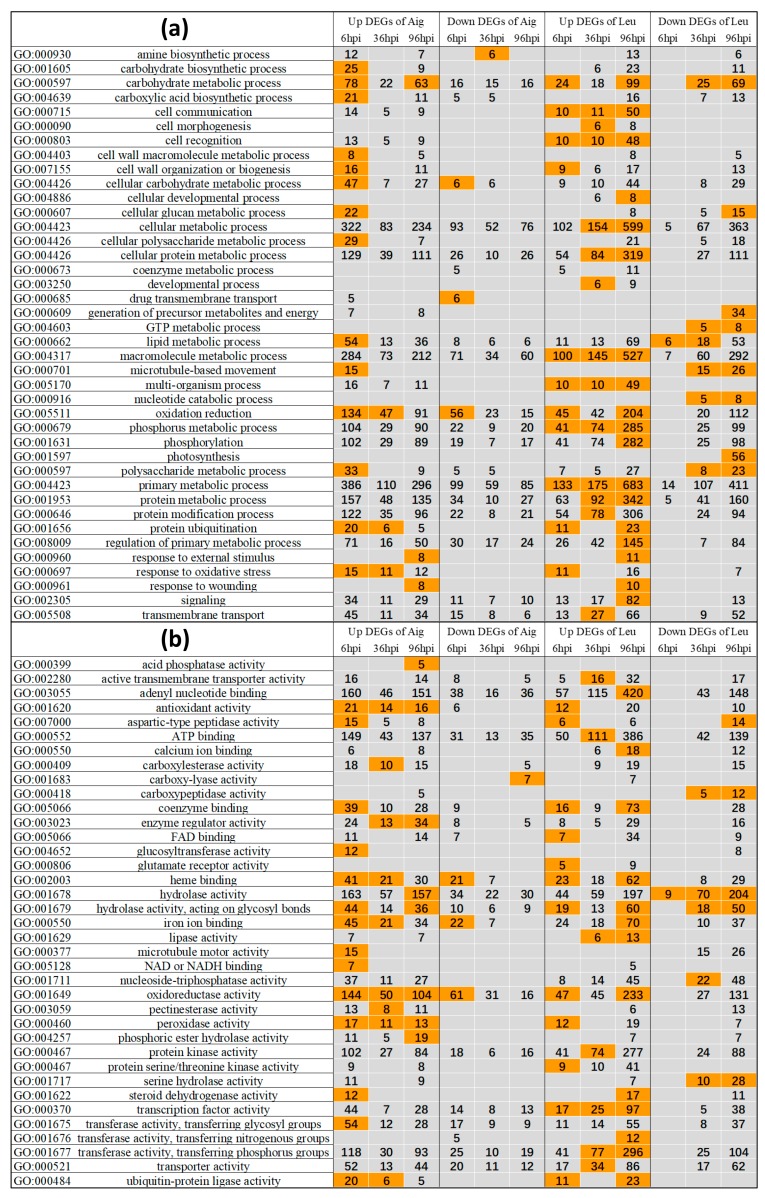
GO enrichment of up or down DEGs of two poplar sections in three infection stages. (**a**) Processes. (**b**) Functions. The GO enrichment was completed under a significance threshold of 0.05 following Hochberg false discovery rate (FDR) correction. The numbers in the diagram indicates the gene number belong to corresponding GO terms. Enriched GO terms were marked by orange.

At six hpi, a large amount of DEGs involved in different biological processes and functions were upregulated in Aig, including carbohydrate metabolic processes (GO:0005975), cell wall organization or biogenesis (GO:0071554), lipid metabolic processes (GO:0006629), microtubule-based movement (GO:0007018), protein ubiquitination (GO:0016567), antioxidant activity (GO:0016209), microtubule motor activity (GO:0003777), oxidoreductase activity (GO:0016491), transferase activity transferring glycosyl groups (GO:0016757), and other terms (Figure 4; Table S2). Furthermore, 99 genes involved in primary metabolic processes (GO:0044238) and 61 genes involved in oxidoreductase activity were significantly downregulated at six hpi in Aig (Figure 4; Table S2). At 36 hpi and 96 hpi, few biological processes were enriched in Aig. Of these processes, the GO term *response to external stress* (GO:0009605) was only enriched at 96 hpi (Figure 4; Table S2). Additionally, the number of DEGs in most of the GO terms was lowest at 36 hpi, implying little activity at this stage. The GO enrichment analysis suggested that Aig reacted more strongly in response to the early stage of MLDP.

The GO enrichment pattern of Leu differed from that of Aig. As the disease progressed, the DEG number of GO terms generally kept increasing (Figure 4; Table S2). The upregulated DEGs of Leu were enriched in biological processes and functions including carbohydrate metabolic processes, cell communication (GO:0007154), cellular metabolic processes (GO:0044237), phosphorus metabolic processes (GO:0006793), protein ubiquitination, signaling (GO:0023052), adenyl nucleotide binding (GO:0030554), calcium ion binding (GO:0005509), oxidoreductase activity, transcription factor (TF) activity (GO:0003700), and others (Figure 4; Table S2). As with Aig, the GO term *response to external stress* was also enriched at 96 hpi in Leu (Figure 4, Table S2). Furthermore, the downregulated DEGs were enriched in carbohydrate metabolic processes, generation of precursor metabolites and energy (GO:0006091), photosynthesis (GO:0015979), hydrolase activity (GO:0016787), and other terms at 96 hpi (Figure 4, Table S2). In these enriched GO terms, photosynthesis was only enriched by downregulated DEGs at 96 hpi, and, furthermore, most of the genes involved in serine hydrolase activity (GO:0017171) were downregulated at 96 hpi (Figure 4, Table S2). The GO enrichments of Leu exhibited much stronger interactions in the latter period of MLDP.

#### 3.3.2. Analysis of Biotic Stress Response Differentially Expressed Genes

**Figure 5:**
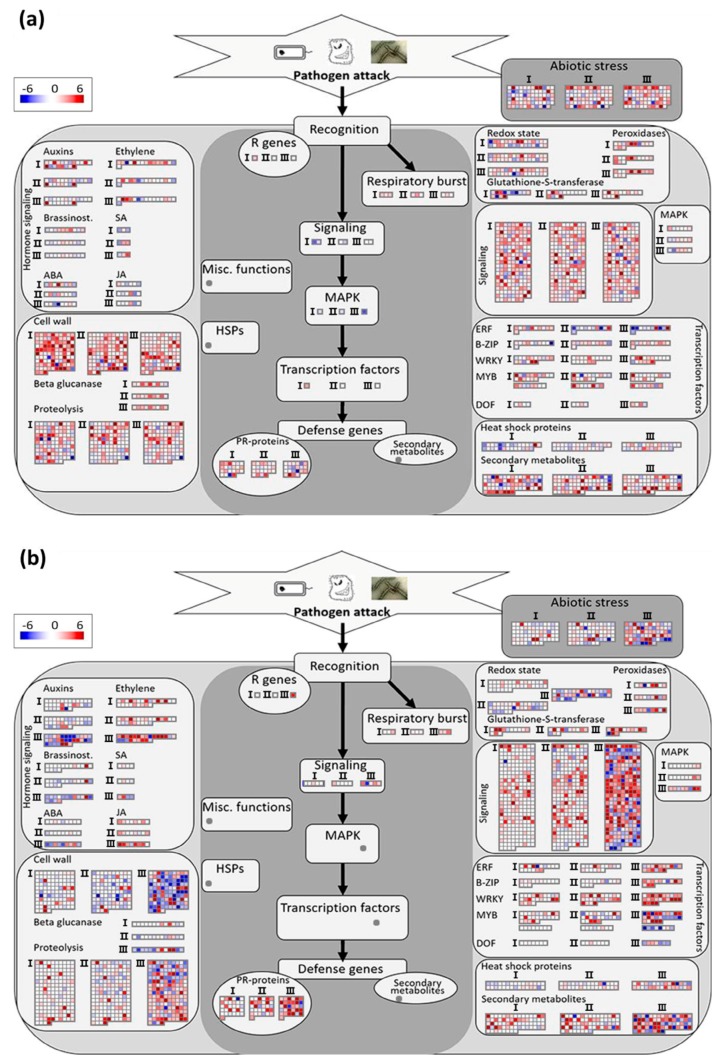
MapMan analysis shows the reactions of two poplar sections after infection: (**a**,**b**) the biotic stress overview of Aig and Leu, respectively. Biotic stress overview finished with installed toolkit in the MapMan after integration of log_2_(fold change) data of all detected DEGs in two poplars. Genes in the main panel (colored with dark gray) are proved in response to biotic stress. Genes on the left and right sides are putatively involved in biotic stress pathways. Up and downregulated transcripts are shown in red and blue, respectively. Roman numerals indicate different infection stages: I represents six hpi, II represents 36 hpi, and III represents 96 hpi. The scale showing the value of log_2_(fold change) from −6 to +6. HSPs: heat shock proteins; MAPK: mitogen activated protein kinase; SA: salicylic acid; JA: jasmonic acid; ABA: abscisic acid; ERF: ETS2 repressor factors; B-ZIP: basic leucine-zipper; MYB: myeloblastosis; DOF: DNA-binding One Zinc Finger; PR-proteins: pathogenesis-related proteins; R genes: resistance genes.

MapMan was used for the analysis of biotic stress signaling pathways. We also further identified the much more crucial genes from the results of MapMan. In Aig, these genes in most of the classifications were significantly expressed at six hpi. In the *auxins* classification, eight genes, including Potri.002G207800 (*TIR1*), Potri.002G082400 (*ILL6*), and Potri.002G234000 (*ATB2*) were downregulated at six hpi (Figure 5a; Table S4). Two genes, including Potri.006G037000 (*ATPIN1/PIN1*) and Potri.013G144300 (*DFL2*), were significantly upregulated at six hpi and 36 hpi, and then downregulated at 96 hpi (Figure 5a; Table S4). Two genes, Potri.006G126500 and Potri.001G031600, were consistently upregulated in the three stages (Figure 5a; Table S4). In the classification *ethylene*, Potri.002G113900 (*ACS8*) was significantly downregulated at six hpi (Figure 5a; Table S4). In the classification *brassinost.*, three genes (Potri.005G124000, Potri.008G067500, and Potri.001G032800) annotated with “cytochrome P450 superfamily protein”, and two genes (Potri.001G386900 and Potri.005G126400) annotated related to “brassinosteroid signaling positive regulator” remained downregulated (Figure 5a; Table S4). Potri.001G389200 (*UGT74E2*), involved in the *salicylic acid* (*SA*) signaling pathway, was always significantly downregulated (Figure 5a; Table S4). Potri.001G024300, involved in the *abscisic acid* (*ABA*) signaling pathway, was significantly upregulated at six hpi and 36 hpi, and then significantly downregulated at 96 hpi (Figure 5a; Table S4). Most genes involved in the classification (*cell wall*), including genes related to pectin metabolism, cellulose metabolism, and saccharide metabolism, were all upregulated during the three infection stages (Figure 5a; Table S4). However, the expressions of several genes in the cell wall, including Potri.018G003100 (*ARA1*), Potri.001G136200 (*CSLD3*), Potri.008G153000 (frigida-like protein), Potri.010G109400 (plant invertase/pectin methylesterase inhibitor superfamily), Potri.018G094800 (*XTH22*), and three other genes encoding expansins (Potri.004G123200, Potri.010G167200, and Potri.006G108000), remained downregulated during the disease progression (Figure 5a; Table S4). Genes in the classification *β glucanase* were mostly upregulated (Figure 5a; Table S4). In the classification *Proteolysis*, the genes annotated with “eukaryotic aspartyl protease family protein”, the genes annotated with “RING/FYVE/PHD zinc finger superfamily protein”, the genes annotated with “serine carboxypeptidase-like”, and most of the genes annotated with “subtilase family protein” remained upregulated, and two genes (Potri.014G108700 and Potri.002G182800) were sharply downregulated at six hpi and significantly upregulated at 96 hpi (Figure 5a; Table S4). More than half of the genes in *Glutathione-S-transferase* exhibited downregulation at six hpi (Figure 5a). In the classification *Signaling*, 25 genes were related to calcium, indicating that the calcium ion signaling pathway might be important (Figure 5a; Table S4). Two genes in *mitogen activated protein kinase* (*MAPK*), including Potri.012G043200 (*MKK9*) and Potri.001G400500 (*RLP33*), remained downregulated as the disease progressed and were significantly reduced at 96 hpi (Figure 5a; Table S4). ETS2 repressor factors (ERF) were mostly upregulated at six hpi and downregulated at 96 hpi (Figure 5a; Table S4). Additionally, most genes encoded by heat shock proteins were downregulated at six hpi (Figure 5a).

In Leu, the majority of genes in the classification of *auxins* were prominently downregulated at 96 hpi (Figure 5b; Table S5). Most genes in the *Ethylene* classification were upregulated in the three stages, and Potri.002G113900 (*ACS8*) showed no expressional changes in the initial two infection stages, but was sharply upregulated at 96 hpi. Additionally, four genes in this class, namely Potri.001G017600, Potri.001G079900 (*ERF-1*), Potri.001G079600, and Potri.001G048200 remained downregulated with a decreasing tendency as the disease progressed (Figure 5b; Table S5). Most genes in the class *brassinost.*, including five genes related to “cytochrome P450”, were always downregulated (Figure 5b; Table S5). With regards to the genes involved in the classification of *SA*, Potri.007G021400 and Potri.007G117200 always showed downregulation (Figure 5b; Table S5). Six genes involved in the classification *jasmonic acid* (*JA*) remained upregulated as the disease developed (Figure 5b). In the classification *cell wall*, most genes, including four genes annotated with “arabinogalactan protein”, one gene annotated with “putative cell wall protein precursor”, five genes annotated with “expansin”, nine genes annotated with “fasciclin-like arabinogalactan”, 11 genes annotated with “pectin lyase-like superfamily protein”, and six genes annotated with “xyloglucan hydrolase” were commonly downregulated at the three infection stages (Figure 5b; Table S5). In the class *proteolysis*, most genes encoding eukaryotic aspartyl protease family proteins, seven genes annotated with “serine carboxypeptidase-like”, and the genes encoding subtilases remained downregulated. Furthermore, 29 out of 36 genes containing a RING domain were significantly upregulated at 96 hpi, and five out of six genes encoding zinc finger (C3HC4-type RING finger) family proteins remained upregulated as the disease progressed (Figure 5B; Table S5). Three genes involved in *respiratory burst* were consistently upregulated and reached the highest expression level at 96 hpi (Figure 5b). Three genes annotated with “cytochrome b561/ferric reductase transmembrane protein family” and most genes encoding thioredoxin in the classification of *Redox state* were downregulated (Figure 5b; Table S5). Eleven out of 13 genes encoding glutathione-S-transferase and six out of eight genes encoding peroxidases were significantly upregulated at 96 hpi (Figure 5b; Table S5). In the classification of *signaling*, almost all genes related to “calcium and genes encoded wall-associated kinase family protein” were upregulated throughout the course of the disease. However, the genes encoding proteins containing IQ-domains and genes encoding leucine-rich receptor-like protein kinase family proteins mostly exhibited downregulation (Figure 5b; Table S5). Six out of eight genes involved in *MAPK* signaling pathways were upregulated (Figure 5b). In the classification of *transcription factors*, genes encoding ERF TFs, basic leucine-zipper (B-ZIP) TFs, and WRKY TFs were mostly upregulated (Figure 5b; Table S5). Of the genes encoding heat shock proteins, four genes annotated with chaperone DnaJ-domain superfamily protein and two genes annotated with double Clp-N motif-containing P-loop nucleoside triphosphate hydrolases superfamily protein were downregulated at 96 hpi (Figure 5b; Table S5). Comparing the genes involved in the same classification of Aig and Leu, *DNA-binding one zinc finger* (*DOF*), *Respiratory burst*, and *SA* had no common genes (Figure S3). The other classifications all contained some common genes; however, only a few genes were shared between the two poplars (Figure S3).

### 3.4. Analysis of Co-Expression

**Figure 6:**
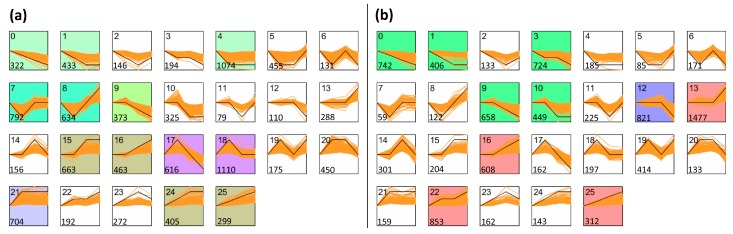
Co-expression patterns of genes of two poplar sections: (**a**) the co-expression patterns of genes for Aig; and (**b**) the co-expression patterns of genes for *Leuce Duby* (Leu). Co-expression analysis was finished using STEM software [29] with inputting log_2_(FPKM + 1) data of all genes of two poplars. Orange lines represented the expression patterns of genes. Black lines represented the expression model of clusters. Clusters were remarked by numbers on the top left corner in profiles. Number on the bottom left corner of a profile show the number of included genes in the profile. The colorized graphs have significances less than *p*-value 0.05. Non-white profiles of the same color represent profiles grouped into a single cluster.

STEM software [29] was used for elucidating the co-expression of genes from the two poplar clones during *M. brunnea* penetration. A total of 26 clusters were revealed in each poplar clone, with the gene numbers within clusters ranging from 1110 to 79 in Aig and from 1477 to 59 in Leu (Figure 6). Among these clusters, three clusters presented gene expressions with an upregulated trend (cluster 16, cluster 22, and cluster 25) following *M. brunnea* infection, while three clusters exhibited downregulated trends, including cluster 0, cluster 3, and cluster 9 (Figure 6). As the disease progressed, the level of different expression of the genes involved in uptrend and downtrend clusters was higher, implying that these genes might be closely associated with the development of MLDP. 

For Aig, the genes in the uptrend clusters were enriched in three GO terms of biological processes and four GO terms of molecular function (Table S6). Additionally, six pathways involved in phenylpropanoid biosynthesis (Ko00940), photosynthesis (Ko00195), cyanoamino acid metabolism (Ko00460), valine, leucine, and isoleucine degradation (Ko00280), sesquiterpenoid and triterpenoid biosynthesis (Ko0090), and regulation of autophagy (Ko04140) were enriched in the KEGG analysis (Table S7). In the downtrend clusters, the genes were classified in 17 biological processes, 16 molecular functions, and 20 cellular components. Among these classifications, a high percentage of genes mapped to biological groups of gene expression, translation, cellular localization, intracellular transport, and protein transport, and a high percentage of genes mapped to functional groups of binding, protein binding, nucleic acid binding, hydrolase activity, acting on acid anhydrides, zinc ion binding, and structural molecule activity (Table S7). Nine pathways were enriched in the KEGG analysis, including spliceosome (Ko03040), RNA transport (Ko03013), ribosome (Ko03010), protein processing in endoplasmic reticulum (Ko04141), mRNA surveillance pathway (Ko03015), ribosome biogenesis in eukaryotes (Ko03008), carbon fixation in photosynthetic organisms (Ko00710), tricarboxylic acid cycle (TCA cycle) (Ko00020), and N-Glycan biosynthesis (Ko00510) (Table S7).

**Figure 7:**
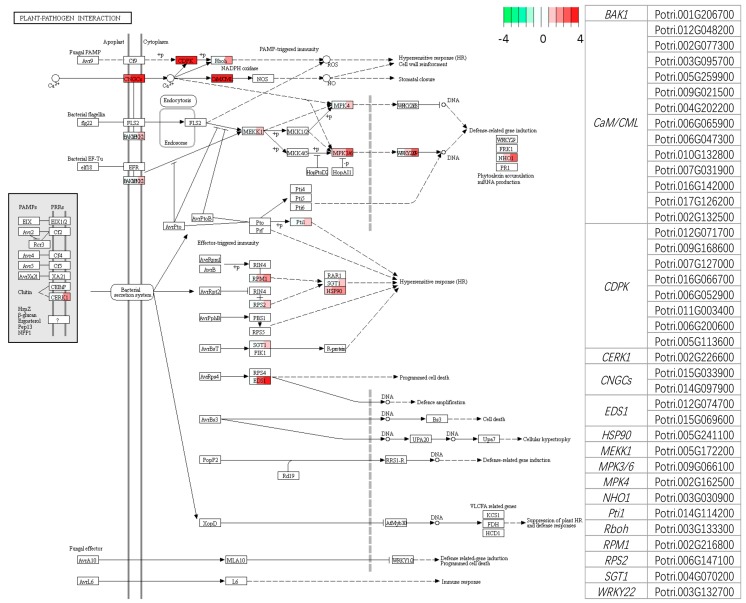
KEGG map (ko04626, plant-pathogen interaction) of Leu-MO45 infection system. The genes involved in plant-pathogen interaction were enriched by genes with uptrend expression in Leu based on clusterProfile, a R package. For each box, from left to right, contrasts 6hpi_vs_CK, 36hpi_vs_CK, and 96hpi_vs_CK, are depicted in color scale representing log_2_(fold change) values. The scale shows the value of log_2_(fold change) from −4 (green) to +4 (red). The list on the right shows the detailed transcripts of Leu of each corresponding gene in KEGG map.

For Leu, the uptrend genes represented GO enrichment of processes in 29 classes and functions in 23 classes (Table S6). The KEGG enrichment analysis showed that 22 pathways were enriched. The plant-pathogen interaction (Ko04626), protein processing (Ko04141), and endocytosis (Ko04144) included more genes in these pathways (Table S7). The expression profiles of the genes involved in plant-pathogen interactions are shown in Figure 7, and the genes related to calcium ion were highly expressed, including *CNGCs*, *CDPK*, and *CALM*, and furthermore, gene *EDS1*, which indirectly regulates programmed cell death, also exhibited a high expression profile as the disease progressed (Figure 7). The downtrend genes were involved in GO terms including 21 processes, 16 functions, and 15 cellular components (Table S6). Fifteen pathways were enriched in the KEGG analysis, and 33 genes were involved in plant hormone signal transduction (Table S7). The genes that regulated cell enlargement, including *AUX1*, *AUX/IAA*, *CH3*, and *SAUR* were more highly downregulated (Figure S4). Additionally, *JAZ* and *MYC2*, which indirectly regulate the stress response in poplar, were significantly downregulated at 96 hpi (Figure S4).

**Figure 8:**
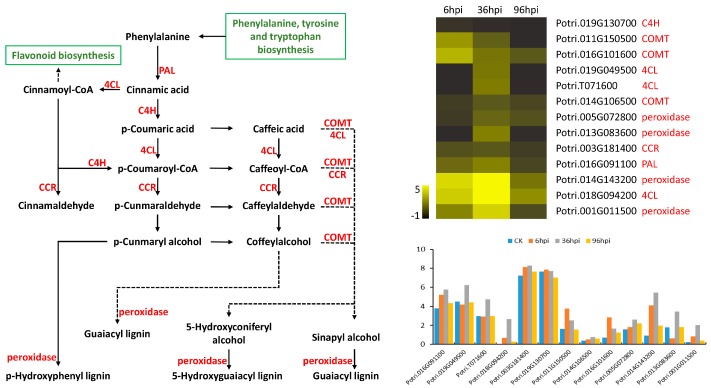
The gene expression pattern of some genes involved in the phenylpropanoid biosynthesis pathway of Aig. Pathway shows genes (in red) and productions in the pathway. The row of heat map represents a gene and the column represents an infection stage. The gene id and annotation are shown on the right of heat map. Histogram shows the gene expression of each gene with the value of log_2_(FPKM + 1). Related pathways are showed in boxes (green). *4CL*: 4-coumarate-CoA ligase; *C4H*: cinnamate 4-hydroxylase; *CCR*: cinnamoyl-CoA reductase; *COMT*: caffeic acid 3-*O*-methyltransferase; *PAL*: phenylalanine ammonia-lyase.

Four clusters, including cluster 12, cluster 14, cluster 20, and cluster 23 were upregulated in the biotrophic stages (36 hpi) and downregulated expression from the biotrophic stages to the necrotrophic stage (96 hpi) (Figure 6). As *M. brunnea* expands slowly in the biotrophic stages and rapidly in the necrotrophic stages, the genes in these four clusters might function to effectively limit the growth of pathogens in poplars. For Aig, the genes involved in the four clusters were enriched in GO terms including six processes and two functions, and 96 genes were involved in transport, and 11 genes were involved in transferase activity (Table S6). The KEGG enrichment of these genes indicated that 19 genes were involved in starch and sucrose metabolism (Ko00500), 13 genes were involved in phenylpropanoid biosynthesis (Ko00940), 12 genes were involved in flavonoid biosynthesis (Ko00941), as well as six other pathways (Table S7). Within the starch and sucrose metabolism pathway, the associated genes control the formation of several enzymes that prompt the formation of d-glucose and dextrin (Figure S5). Phenylpropanoid biosynthesis genes included one gene encoding phenylalanine ammonia-lyase (*PAL*), three genes encoding 4-coumarate-CoA ligase (*4CL*), one gene encoding cinnamoyl-CoA reductase (*CCR*), one gene encoding cinnamate 4-hydroxylase (*C4H*), three genes encoding caffeic acid 3-*O*-methyltransferase (*COMT*), and four genes encoding peroxidases (Figure 8). Among these genes, the expression levels of *CCR* (Potri.003G181400) and *C4H* (Potri.019G130700) were highest (Figure 8). The genes involved in flavonoid biosynthesis included two *CHS*, one *CHI*, two *F3M*, two *F3H*, two *DFR*, one *LDOX*, and two *LAR* (Figure S6). Of these genes, *CHS* (Potri.014G145100), *CHI* (Potri.010G213000), *F3M* (Potri.013G073300), and *LDOX* (Potri.001G113100) exhibited the highest expression levels (Figure S6).

For Leu, the genes exhibiting upregulated expression in the biotrophic stages and downregulated expression in the necrotrophic stage were enriched in GO terms including nine processes, and metabolic processes contained the most genes (612), 24 of which were classified in generation of precursor metabolites and energy (Table S6). Eleven pathways were identified in the KEGG enrichment analysis, including ribosome biogenesis in eukaryotes (Ko03008), purine metabolism (Ko00230), carbon fixation in photosynthetic organisms (Ko00710), fatty acid elongation (Ko00062), and others (Table S7). The expression patterns of 24 genes involved in ribosome biogenesis in eukaryotes indicated that *NOP1* (Potri.015G147500), *NOP56* (Potri.012G092900), *SNU13* (Potri.013G116800), and *NOP10* (Potri.005G226300) maintained higher expression values compared to the other genes (Figure S7). Furthermore, seven genes were also involved in flavonoid biosynthesis. Of these genes, Potri.005G028000 and Potri.006G141400 were most highly expressed at six hpi and 36 hpi, and then sharply decreased at 96 hpi (Figure S8).

### 3.5. Expression Analysis of Genes Encoding Disease Resistant Proteins, Receptor-Like Kinases, Chitinases, and Defensins

**Figure 9:**
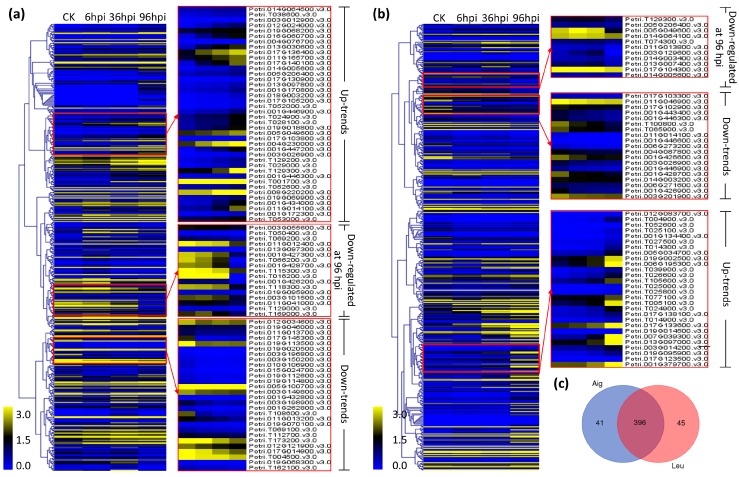
Clustering of gene expression of genes annotated with disease resistant gene in two poplar sections: (**a**) clustering result of Aig; and (**b**) clustering result of Leu. The clustering is finished using hierarchical analysis according to values of log_2_(FPKM + 1) for each disease resistant gene. The clustering is performed on Multiple Experiment Viewer (MeV). The row of heat map represents a gene and the column represents an infection stage. The expression level of genes involved in “up-trend” keep increasing as the disease development. The expression level of genes involved in “down-trend” keep decreasing as the disease development. The expression of genes involved in “down-regulated at 96 hpi” sharply deceased at 96 hpi compared with 36 hpi. (**c**) Venn diagram shows the overlapping disease resistant genes between Aig and Leu.

Of the genes encoding disease resistant proteins in Aig, 40 genes, of which five (Potri.T001700, Potri.004G230000, Potri.008G220200, Potri.T129300, and Potri.017G136400) exhibited the highest expression levels, indicated upregulated expression as the disease progressed (Figure 9a). Seventeen genes showed an upregulated trend during the initial two infection stages, and a downregulated trend at 96 hpi, four of which (Potri.T066200, Potri.T016200, Potri.001G428700, and Potri.001G427300) decreased sharply at the third infection stage (Figure 9a). Additionally, 28 genes exhibited a downregulated trend of expression, and the five most highly expressed genes included Potri.012G034600, Potri.019G113500, Potri.T173200, Potri.T004500, and Potri.005G100700 (Figure 9a). In Leu, 28 genes encoding disease resistant proteins exhibited upregulated expression during *M. brunnea* infection, including Potri.013G097000, Potri.017G133600, Potri.001G379700, Potri.006G195300, and Potri.007G039300, which were most highly expressed at 96 hpi (Figure 9b). Twenty genes showed downregulated expression, with one gene (Potri.011G046900) that putatively encodes a disease resistance protein in the Toll/interleukin-1 receptor (TIR) class, and five genes (Potri.T065900, Potri.001G428700, Potri.001G426600, Potri.T100800, and Potri.003G201900) that encode NB-ARC domain-containing disease resistance proteins, exhibiting a maximum drop between six hpi and 96 hpi (Figure 9b). Eleven genes were upregulated at the initial two infection stages and exhibited downregulated expressed at 96 hpi, five of which were highly differentially expressed, including Potri.005G049600 (leucine-rich repeat (LRR) family), Potri.003G129600 (TIR-NBS class), Potri.013G007400 (CC-NBS-LRR class), and Potri.011G013800 and Potri.017G104300 (TIR-NBS-LRR class) (Figure 9b). Many disease resistant proteins were shared between the two sections; however, 41 genes were specific to Aig, while 45 genes were specific to Leu (Figure 9c).

Receptor-like protein kinases (RLKs) are transmembrane proteins that contain putative amino-terminal extracellular domains and carboxyl-terminal intracellular kinase domains, and participate in many biological processes, including plant disease resistance [38]. For Aig, a total of 26 genes maintained upregulated expression as MLDP progressed, and five genes, including Potri.004G024400 (*CRK10*), Potri.014G147300, Potri.019G099200, Potri.T090500 (*CRK25*), and Potri.003G166100 (*SERK2*) were most highly expressed (Figure S9A). Seventeen genes, containing six *RLK1*s and four *RLK2*s, showed a downregulated trend, and the five genes that exhibited initial high expression levels were Potri.019G011400 (*RLK1*), Potri.013G059900 (*RLK1*), Potri.017G094400 (*RLK2*), Potri.014G144600 (*RLK2*), and Potri.013G036600 (Figure S9a). In Leu, of the 32 genes that displayed upregulated expression following inoculation with *M. brunnea*, Potri.001G014100 (*RLK1*), Potri.012G124100, Potri.004G024900 (*CRK25*), Potri.018G111700 (*CRK34*), and Potri.004G023800 (*CRK10*) exhibited the largest difference between six hpi and 96 hpi (Figure S9b). There were 17 genes that were downregulated. Of these, 14 were annotated with leucine-rich receptor-like protein kinase family proteins, and the first five genes that showed the largest difference between six hpi and 96 hpi all encoded leucine-rich receptor-like protein kinase family proteins (Figure S9b). The majority of disease resistant proteins were common between the two genera; however, 17 genes were specific to Aig, and 19 genes were specific to Leu (Figure S9b).

Chitinases are important for disease resistance in plants. A total 25 genes in Aig and 23 genes in Leu expressed chitinases (Figure S10a,b). The Venn diagram indicated that most of the genes were included in the overlapping region; however, three genes (Potri.002G242000, Potri.014G093000, and Potri.013G125100) were specifically involved in Aig, and one gene (Potri.002G186500) was specifically involved in Leu (Figure S10c). Plant defensins respond to pathogen attack in a variety of ways including by disrupting microbial membranes and trigging many other defense responses. Only one gene (Potri.002G033200) was found to code defensin-like proteins in Aig, and this gene was significantly upregulated during the initial infection with *M. brunnea* and exhibited a downregulated trend as the disease progressed (Figure S10d). As no defensin protein or defensin-like protein genes were detected as significantly expressed in Leu, the gene Potri.002G033200 might play an important role for the patholgenicity of *M. brunnea.*

## 4. Discussion

MLDP has been studied for more than 30 years. However, previous research on the interactions of this disease neglected its unique infection process as a hemibiotrophic pathogen [20,39,40]. The unique interaction of MLDP with the plant host provides important molecular information for the breeding of resistant poplar varieties. However, few data exist for assessing the differences in the response to this disease between Aig and Leu. To precisely evaluate the interactions between the host and pathogen around characteristic infection structures in MLDP, we analyzed the changes in genomic expression of host poplars to elucidate the possible molecular responses.

In previous research, RNA-Seq samples of susceptible MLDP were only collected at two-time points without a control group or microscope histopathology analysis [20]. In this study, the RNA-Seq samples included a control group and three infection groups at the crucial stages of disease development, including the stages with characteristic infection structures including appressoria (six hpi), IVs (36 hpi), and SH (96 hpi). The appressoria constitute important structures for fungal pathogens in many plant diseases, and many interactions have been found between the hosts and pathogens relating to the appearance of the structure [41,42]. In typical hemibiotrophic fungal pathogens such as *Magnaporthe oryzae* and *Colletotrichum* spp., IVs are formed in the biotrophic phase and perform many functions including the secretion of effectors [43,44,45]. SH are symbolic of the necrotrophic infection stage in hemibiotrophic pathogens, and these fungi predominantly generate SH for killing plant cells rather than for growth within living host cells [46,47,48]. We performed RNA-Seq analysis based on the formations of these three fungal infection structures with the aim of obtaining important molecular information on MLDP.

In this work, a larger number of RNA-Seq clean data (248.79 GB) were obtained in comparison to existing genomic transcriptome studies in MLDP (27.3 GB) [20]. To further assess the biological and molecular changes associated with the progression of the disease in the two poplars, we performed GO enrichment, pathway enrichment, and co-expression analysis. The findings are discussed below. 

### 4.1. Recognition and Signaling

Plants have two immune systems in pathogen defense: pathogen-associated molecular patterns (PAMP)-triggered immunity (PTI) and effector-triggered immunity (ETI) [49]. RLKs are crucial for defense against disease in plants and other biological processes, such as the RLKs that contain LRR domains that are reported to act as pattern recognition receptors (PRRs) in the recognition of PAMPs in plants and PTI [48]. In this study, a total of 29 LRR-RLKs were expressed. Of these LRR-RLKs genes, only one (Potri.013G103200) was specific to Aig and one (Potri.003G175700) was specific to Leu (Figure S10a). To the best of our knowledge, the functions of the two genes have not been clearly established in poplar, including their regulatory pattern in response to pathogens. After infection by hemibiotrophic pathogens, many resistance genes encoding NBS-LRR plant proteins can recognize effectors and trigger ETI [49,50]. Considering that *M. brunnea* is a typical hemibiotrophic pathogen, these NBS-LRR proteins might have important functions for poplars in MLDP resistance. We identified 145 and 141 genes containing NBS-LRR domains in Aig and Leu respectively, and of these genes, 16 and 13 were respectively associated with Aig and Leu (Figure S10b). Additionally, the gene (Potri.003G149800) annotated as encoding RPP8, which was reported to be involved in the response to challenge from *Hyaloperonospora arabidopsidis* in *Arabidopsis thaliana* [51], was relatively highly expressed in both the poplar clones (Figure S10c). As LRR-RLKs and NBS-LRRs are crucial in triggering plant immune systems, the differences of these two protein families between Aig and Leu might be important.

Following the recognition of fungal pathogens, MAPK signaling appears to cross-talk with a variety of stress responses to initiate plant immunity [52]. Some research also reported that fungal pathogens and drought could activate MAPK in poplars [53,54]. In Aig, seven genes including *MPK20*, *MPKK9*, and *MAPKKK18* in MAPK pathways exhibited differential expression as the disease progressed (Table S4). In *Arabidopsis*, the activation of *MPKK9* could induce ethylene biosynthesis, which is important in mediating several types of induced resistance [55,56]. In Leu, eight genes including *MPK15* (Potri.008G130000), *MPK7* (Potri.007G020100), *MPKK9* (Potri.015G030700), *MPK3* (Potri.009G066100), *MAPKKK18* (Potri.014G155000), and *MAPKKK5* (Potri.002G129100) were differentially expressed (Table S5). *MAPKs* have been shown to be a key component in the induced response when infected by *Melampsora* leaf rust in poplar, and two *MAPKs*, the orthologs of *A. thaliana MPK3* and *MPK6*, exhibit important roles in this pathway [57]. *MPK3* was also detected as differentially expressed in Leu, and it is interesting to note that the expression pattern of the gene maintained an upregulated trend as the disease progressed, which corroborates the research of Boyle et al. [57]. In fact, *MPK3* was also detected as highly expressed in Aig, and decreased in expression following inoculation. Furthermore, the *MPKK9*-*MPK3* modules have been confirmed to play essential roles in plant physiology [55]. *MPKK9* and *MPK3* exhibited almost consistent expression trends in Aig and Leu.

Calcium ions act as an important secondary messenger in many other biological processes in plant cells [58]. Changes in calcium signaling have been reported in response to biotic stress, and some of the elicitors secreted by fungal pathogens were shown to increase these changes in calcium signaling [58]. Twenty-five and 38 DEGs in Aig and Leu were, respectively, found to be related to calcium signaling (Tables S4 and S5). Of these genes, five were orthologous with *A. thaliana* calcium-dependent protein kinases (CDPKs) and most of them were upregulated when infected by *M. brunnea*. CDPKs can phosphorylate special substrates in PTI and ETI to control transcriptional reprogramming of immune genes and the hypersensitive response [59]. As the CDPKs in this study generally exhibited upregulated expression trends, they might be important in disease development. Calmodulins (CAMs) are crucial in calcium ion signaling and also regulate several biological processes including hormone biosynthesis and phosphorylation. In soybean and tobacco, several CAM and CAM-like (CML) proteins have been reported to enhance resistance to a wide spectrum of pathogens [60,61]. In Aig, *CAM8* (Potri.005G052800), which maintained downregulated expression following infection by *M. brunnea*, constitutes a uniquely differentially expressed CAM gene. The downregulation of pathogens induced by CAM was previously found to impact disease resistance in tobacco [62]. In Leu, the genes orthologs with *A. thaliana* CML were all upregulated during disease development (Table S5). Additionally, Potri.013G029100 annotated with *BON3*, which was reported to be a negative regulator of several receptor-like genes [63], was drastically upregulated as the disease progressed in Leu (Table S5). Many genes related to calcium signaling were differentially expressed in both Aig and Leu (Figure 5), indicating the important role of this signaling molecule in response to MLDP in poplars.

### 4.2. Transcriptional Regulation

TFs are key proteins that decode organism genomes to express a precise set of RNAs and proteins by binding to specific DNA sequences [64]. In plants, TFs participate in the disease response and are necessary in disease resistance [65]. Many TFs were differentially expressed in the two poplars in response to MLDP. As the disease progressed, ERF TFs were mostly downregulated in Aig and upregulated in Leu (Figure 5). As previously reported, ERF TFs regulate the expression of developmental genes, pathogenesis-related genes, and some genes in hormone biosynthesis in response to stress [66]. Most of the ERFs in Aig were downregulated as MLDP progressed, which might weaken the defense capability of poplar. Conversely, many ERFs in Leu were significantly upregulated at 96 hpi. TGA factors are believed to regulate SA signaling in response to pathogens and trigger disease resistance in plants [67]. One gene (Potri.006G058800) encoding TGA10 factors was downregulated during the initial two infection stages and then upregulated at 96 hpi in both Aig and Leu (Figure 5; Tables S4 and S5). WRKY genes deter pathogens by influencing diverse metabolic pathways and cellular physiological processes such as JA and SA signaling [68]. The upregulated WRKY TFs at 96 hpi might regulate JA and SA signaling in Leu (Figure 5; Tables S4 and S5). Furthermore, myeloblastosis (MYB) genes were downregulated at 96 hpi in Leu. MYB TFs also control many plant-specific processes including the hormone and disease response [69,70]. In the defense against pathogens, most TFs are reported to regulate hormone signaling to trigger hypersensitive responses and other processes [71,72]. In general, during *M. brunnea* infection, many TFs in Aig and Leu were differentially regulated, implying changes in hormone signaling pathways. However, many differences also exist between the two poplar sections. Most TFs in Aig were upregulated at the initial stage of MLDP, and many TFs in Leu were upregulated at the latter stage of MLDP. These differences might influence hormone signaling pathways in the two poplars. In Leu, many TFs associated with hormone signaling pathways were significantly upregulated at 96 hpi, implying a strong hormone signaling response. In fact, many genes involved in ethylene signaling, SA signaling, and JA signaling also showed significant upregulation at 96 hpi in Leu. Hypersensitive cell death in plants was previously reported to successfully protect against biotrophic pathogens, and necrotrophic pathogens were reported to resist the hypersensitive responses of host plants and utilize the dead tissue [73]. The necrotrophic phase of hemibiotrophic pathogens is considered to be similar to necrotrophic pathogens [74]. Considering that hormone signaling could trigger a hypersensitive response in plants, and that many genes associated with plant hypersensitive responses in plant–pathogen interaction pathways were also significantly upregulated at 96 hpi in Leu (Figure 7), hypersensitive responses possibly occurred at the necrotrophic phase of *M. brunnea* in Leu. However, further studies are required to verify this.

### 4.3. Primary and Secondary Metabolic Responses

Phytopathogens have been reported to influence the primary metabolism of plants to achieve successful infection [75,76]. Pathogens regulate photosynthesis for several reasons including carbon utilization, and the downregulation of photosynthesis is considered to be a general response of plants to susceptible pathogens [75,76,77]. The expressions of many genes related to photosynthesis were also downregulated in both Aig and Leu, but varied more widely in Leu, implying a more general control of photosynthesis for *M. brunnea* f. sp. *monogermtubi*. However, co-expression analysis showed that six genes exhibiting upregulated expression were enriched in photosynthesis pathways for Aig (Table S6). Most downregulated genes in photosynthesis at 96 hpi in Aig were annotated with photosystem reaction center proteins (Table S6). Many genes (over than 90%) in photosynthesis were downregulated at 96 hpi in Leu, and these genes were also significantly enriched in photosynthesis in the GO and KEGG analysis (Tables S2, S6 and S7). These results indicated that the pathogen might interfere with photosynthesis in the two poplars. However, the expression of several genes in photosynthesis was also slightly downregulated in resistant plants inoculated with pathogens [77]. Carbohydrate metabolism could change in response to pathogens, as plants offer metabolizable carbohydrates to fungi [76]. In both Aig and Leu, the upregulated DEGs were significantly enriched in carbohydrate metabolic process at six hpi and 96 hpi, and furthermore, some of the downregulated DEGs were also enriched in this metabolic process (Tables S2 and S6). These results suggested that changes in carbohydrate metabolism were common in MLDP on Aig and Leu. Similarly, in response to the infection of *M. oryzae*, which is also a hemibiotrophic pathogen, the genes of the plant host were also highly enriched in carbohydrate metabolic processes based on GO analysis [78]. It is worth mentioning that Potri.017G040800, which is involved in carbohydrate metabolism and was annotated with beta-amylase 5 (*BAM5*), was always significantly downregulated during disease progression in both Aig and Leu (Figure S10d). Previous studies showed that BAM was able to induce changes in maltose accumulation, which has been shown to protect plant membranes [79].

Many studies have shown that plants are able to synthesize and release secondary metabolites to protect against a wide variety of microorganisms [80]. In response to *M. brunnea*, a few genes involved in phenylpropanoid biosynthesis were upregulated in both poplars. Additionally, 13 genes, which were upregulated at the biotrophic stages and downregulated at the necrotrophic stage, were also enriched in phenylpropanoid biosynthesis pathways in Aig (Table S7). General phenylpropanoid metabolism participates in the synthesis of many other secondary metabolites [81]. SA, as a major substance for many plants in the response to pathogens, is also produced via phenylpropanoid biosynthesis, and has been shown to be important in the biotic and abiotic stress response [82]. Changing phenylpropanoid metabolism might also regulate the production of SA and assist the host to defend against MLDP. Furthermore, various forms of lignin, which are a major component of certain plant cell walls [83,84], are also produced in phenylpropanoid pathways. The cell wall of the host plant has been reported to be a dynamic barrier against pathogens [85]. Most of the DEGs related to cell wall were upregulated in Aig but downregulated in Leu (Figure 5). The distinct expression patterns of the genes involved in the cell wall between the two poplar sections probably account for the differences in *M. brunnea* expansion observed between two poplars. The cell walls of plants constitute an early defense against fungal penetration. Several upregulated DEGs annotated with pectin methylesterase inhibitors were enriched in pectinesterase activity in Aig; however, these genes in Leu were mostly downregulated at 96 hpi (Tables S4 and S5). Pectin also defends against pathogens, and the overexpression of pectin methylesterase inhibitors has been proven to be effective in restricting fungal pathogens [86]. Considering these results, we hypothesize that even though it does not prevent the penetration of *M. brunnea*, reinforcing the cell walls might limit the expansion of the pathogen. However, further studies are required to verify this. Flavonoids are also synthesized via the phenylpropanoid pathway and play important roles in the interactions between plants and other organisms [87]. In Aig and Leu, the genes upregulated at the initial infection stages and downregulated at 96 hpi were both enriched in the flavonoid biosynthesis pathway (Table S7). Based on the above discussion, we believe that the phenylpropanoid metabolism pathway significantly changes in response to MLDP.

In summary, *M. brunnea* triggered many changes in gene expression and altered the primary and secondary metabolism of the host cells in both two poplar sections. For poplars, the genes in MAPK signaling and calcium signaling were closely related with the reactions caused by the infection of *M. brunnea*. The genes associated with cell wall metabolism, photosynthesis, and phenylpropanoid metabolism played important roles for poplar in response to MLDP. Besides, hypersensitive responses probably happen during the development of MLDP. Furthermore, many important genes showed different expression patterns between the two poplars.

## 5. Conclusions

This study presents a large set of DEGs and elucidated their temporal regulation during specific stages of disease development. These data are important for understanding the molecular mechanism of the development of this disease. By comparing two different susceptible interaction systems of this disease, we provide useful resources for revealing the molecular mechanisms of this complicated phenomenon. Many genes related to cell wall metabolism and phenylpropanoid metabolism present similar expression trends with the disease development between two poplars. However, major differences in gene expression were also observed between the two poplar subgenera. Aig was most responsive at the initial infection stage, while Leu largely interacted with *M. brunnea* at the necrotrophic phase. Our findings bring a novel resource for understanding the interactions between *M. brunnea* and poplar. There could be quantitative differences in virulence and in turn qualitative differences in gene expression between poplar species from the same section. It is worth mentioning that our results could not completely represent all poplar species of the two poplar sections. To completely understand recognitions on the level of poplar sections, more poplars species should be studied. To the best of our knowledge, this study also constitutes the first to reveal the molecular mechanisms of poplar in response to the transition of hemibiotrophic fungal pathogens from the biotrophic phase to the necrotrophic phase. Further investigations will be focused on functional mapping and validation of the crucial genes. In practice, this study provides useful information for guiding the development of poplar resistant varieties toward *M. brunnea*.

## Figures and Tables

**Table 1:** Statistics of reads mapped onto the poplar reference genomes.

Sample	Replicate 1		Replicate 2		Replicate 3	
	Total Reads ^a^	Mapped Reads ^b^	Total Reads	Mapped Reads	Total Reads	Mapped Reads
Aig-CK	39,795,332	29,021,681 (72.93%)	42,248,380	30,635,622 (72.51%)	37,445,738	27,135,653 (72.47%)
Aig-6hpi	109,470,584	79,720,175 (72.82%)	66,456,714	47,641,251 (71.69%)	74,871,338	54,150,365 (72.32%)
Aig-36hpi	59,014,678	42,284,488 (71.65%)	75,926,010	55,006,529 (72.45%)	73,458,950	54,031,383 (73.55%)
Aig-96hpi	62,112,734	42,113,564 (67.80%)	66,706,786	44,826,241 (67.20%)	88,780,204	61,825,740 (69.64%)
Leu-CK	39,274,080	21,132,514 (53.81%)	44,843,154	24,443,778 (54.51%)	61,385,552	33,190,001 (54.07%)
Leu-6hpi	82,348,652	43,884,193 (53.29%)	71,055,446	36,835,745 (51.84%)	88,607,696	46,844,883 (52.87%)
Leu-36hpi	89,169,026	51,010,245 (57.21%)	74,696,580	44,364,679 (59.39%)	73,203,752	42,947,392 (58.67%)
Leu-96hpi	69,355,868	35,080,780 (50.58%)	92,556,954	47,437,815 (51.25%)	85,561,166	44,456,042 (51.96%)

^a^ Total Reads, the number of reads generated from RNA-seq after filtration. ^b^ Mapped Reads, the number of reads mapped to the reference genomics, percentages calculated for the total read. Aig: Aigeiros; Leu: *Leuce Duby*; hpi: hours post inoculation.

## Supplementary Materials

The following are available online at http://www.mdpi.com/2073-4425/9/3/154/s1. Figure S1: Validation of RNA-Seq expression patterns in two poplar sections using qRT-PCR; Figure S2: Common DEGs kept up or downregulation in three infection stages between two poplar sections; Figure S3: Common genes of each classification in Figure 5 between two poplar sections; Figure S4: KEGG map (ko04075, plant hormone signal transduction) of Leu-MO45 infection system; Figure S5: KEGG map (ko00500, starch and sucrose metabolism) of Aig-MU39 infection system; Figure S6: The gene expression pattern of some genes involved in the flavonoid biosynthesis pathway of Aig; Figure S7: The gene expression pattern of some genes involved in the ribosome biogenesis in eukaryotes pathway of Leu; Figure S8: The expression pattern of some genes involved in the flavonoid biosynthesis pathway of Leu; Figure S9: Clustering of gene expression of genes encoding RLKs in two poplar sections; Figure S10: Clustering of gene expression of genes encoding chitinases in two poplar sections; Figure S11 Analysis of several genes played important roles in plant in response to pathogens, Table S1: RT-PCR primers used in this study; Table S2: Details of GO terms enriched by up and downregulated DEGs of Aig at 6, 36 and 96 hpi of three infection stages in Figure 4; Table S3: Details of GO terms enriched by up and down regulated DEGs of Leu at 6, 36 and 96 hpi of three infection stages in Figure 4; Table S4: Details of Mapman analysis for Aig in Figure 5a; Table S5: Details of Mapman analysis for Leu in Figure 5b; Table S6: GO enrichment analysis of genes selected according to co-expression analysis (Figure 6); Table S7: KEGG enrichment analysis of genes selected according to co-expression analysis (Figure 6).
