# Supplementary material for: Genome-Wide Transcriptome Analysis Reveals the Comprehensive Response of Two Susceptible Poplar Sections to Marssonina brunnea Infection
Source: Genes (Basel). 2018 Mar 12;9(3):154. doi: 10.3390/genes9030154 (PMC5867875; doi:10.3390/genes9030154)
Supplement: Supplementary file 1 [file genes-09-00154-s001.pdf]

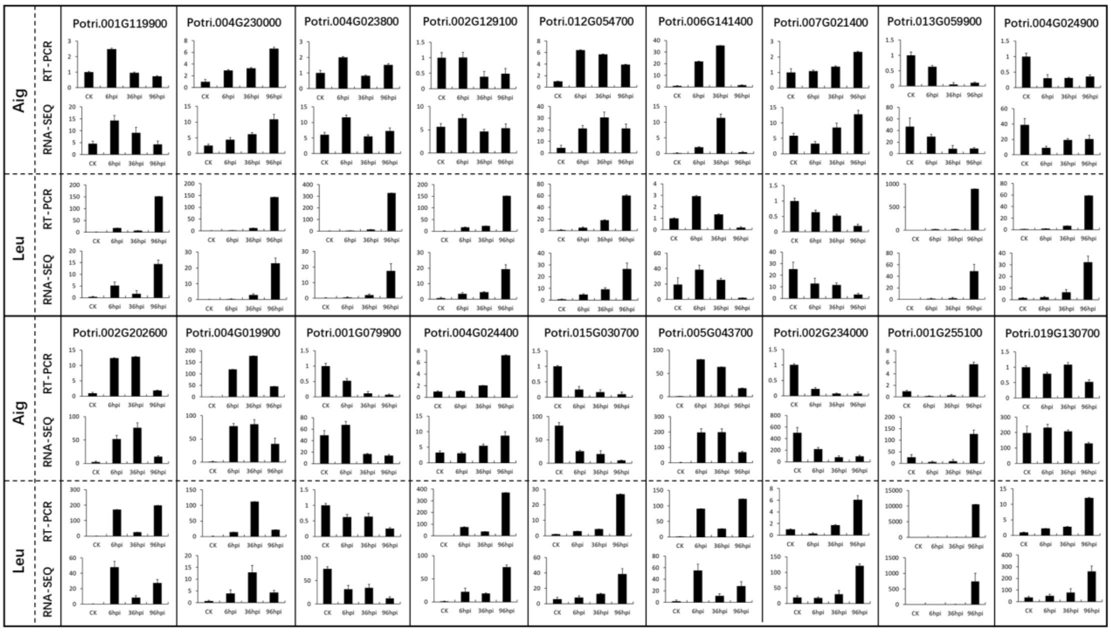

Supplementary Figure S1: Validation of RNA-Seq expression patterns in two poplar sections using qRT-PCR.

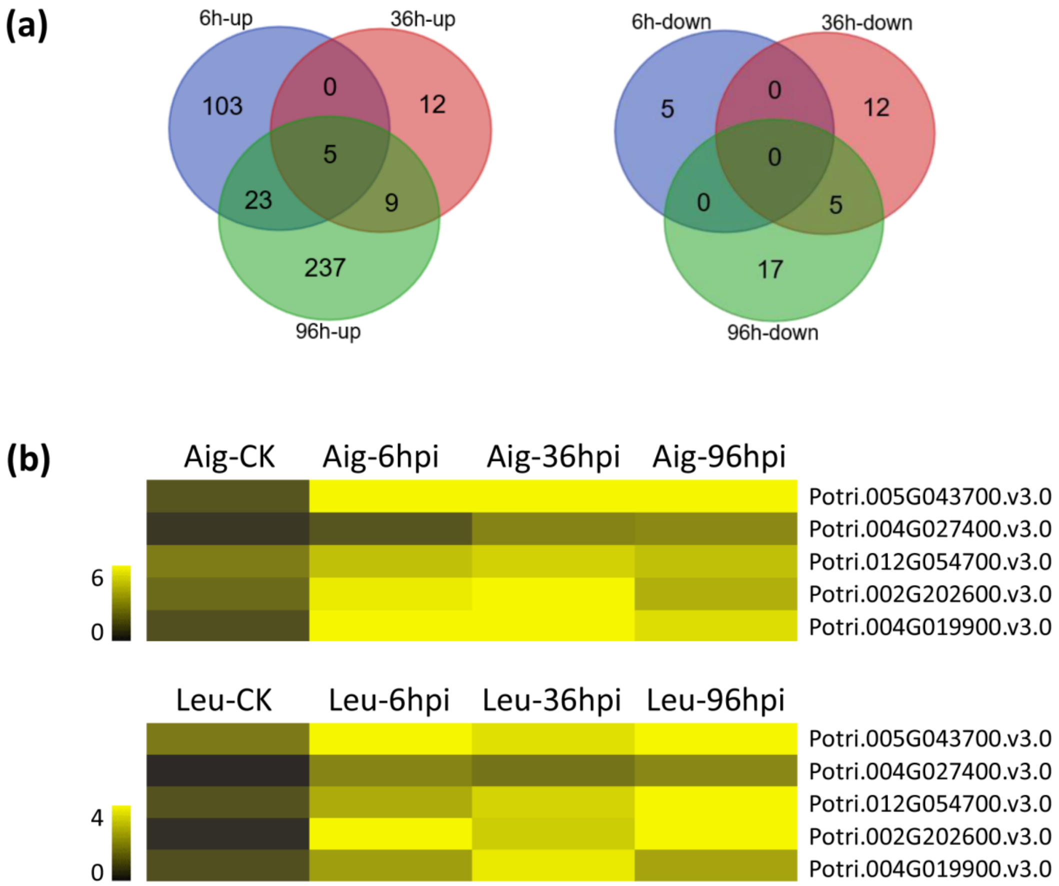

Supplementary Figure S2: Common DEGs kept up- or down-regulation in three infection stages between two poplar sections.

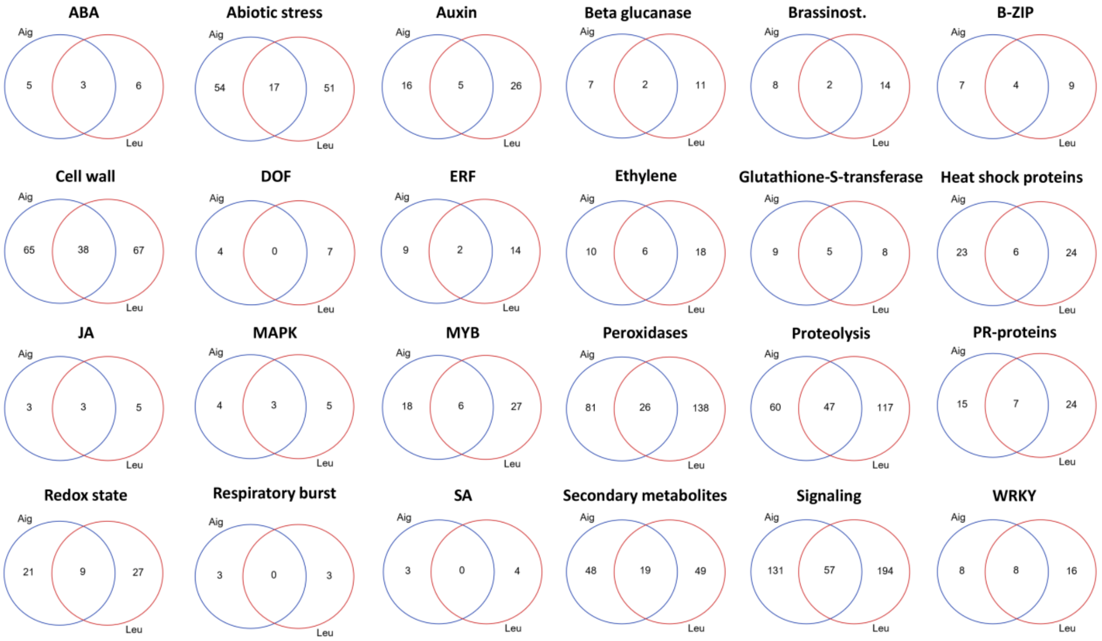

Supplementary Figure S3: Common genes of each classification in Figure 5 between two poplar sections.

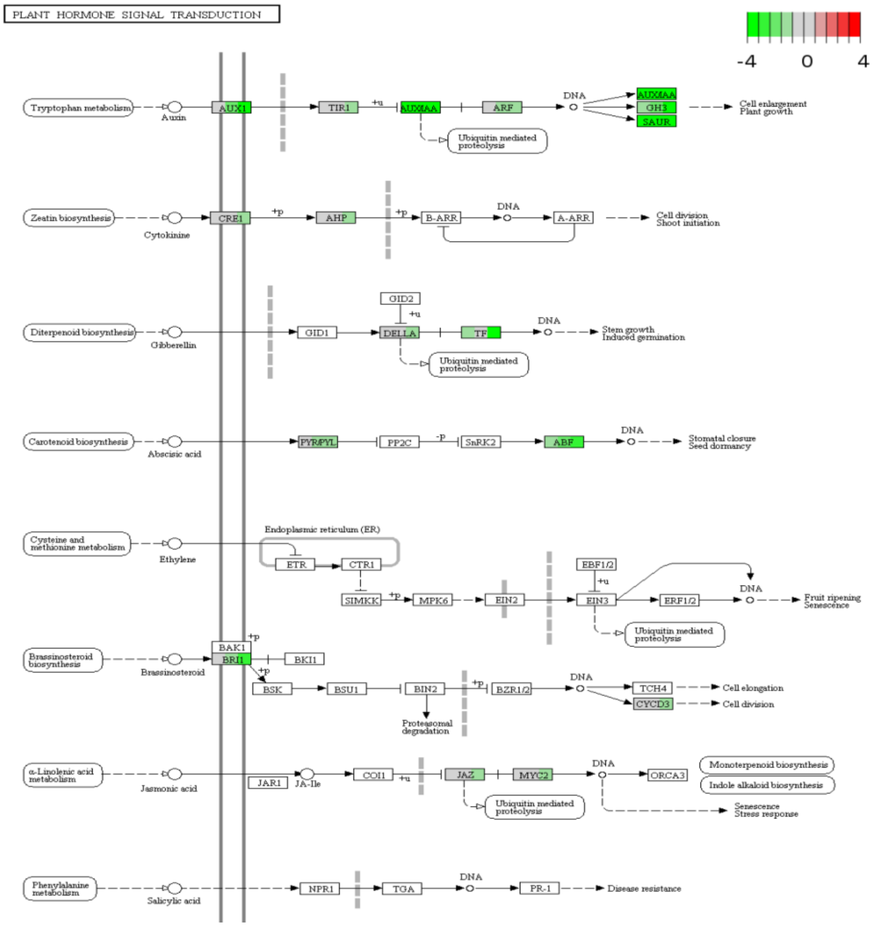

AHP	Potri.009G145800
SAUR	Potri.T156700
	Potri.004G164400
	Potri.004G165600
	Potri.004G165300
	Potri.004G164300
	Potri.009G126500
	Potri.009G126300
GH3	Potri.002G206400
	Potri.013G144300
BRI1	Potri.005G086500
CYCD3	Potri.009G097800
DELLA	Potri.004G089800
ABF	Potri.009G101200
	Potri.004G140600
CRE1	Potri.014G164700
AUX/IAA	Potri.008G172400
	Potri.013G041400
	Potri.003G056900
	Potri.002G044900
	Potri.003G051300
	Potri.006G255200
	Potri.005G053800
	Potri.002G108000
JAZ	Potri.015G035800
AUX1	Potri.004G172800
	Potri.016G113600
ARF	Potri.006G077800
MYC2	Potri.014G103700
TF	Potri.014G111400
	Potri.005G207200
PYR/PYL	Potri.001G092500
TIR1	Potri.002G207800

Supplementary Figure S4: KEGG map (ko04075, plant hormone signal transduction) of Leu-MO45 infection system.

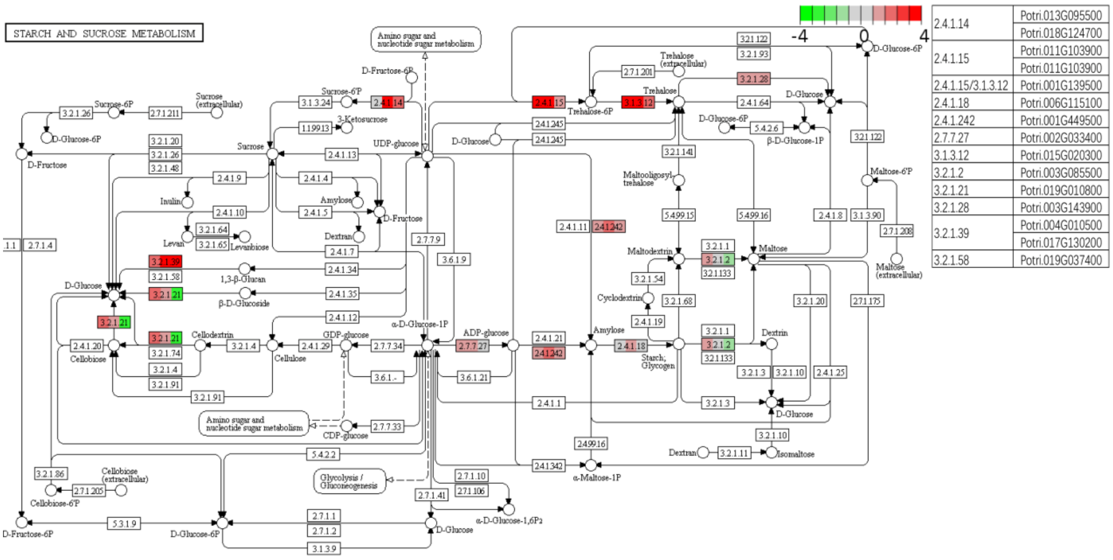

Supplementary Figure S5: KEGG map (ko00500, starch and sucrose metabolism) of Aig-MU39 infection system.

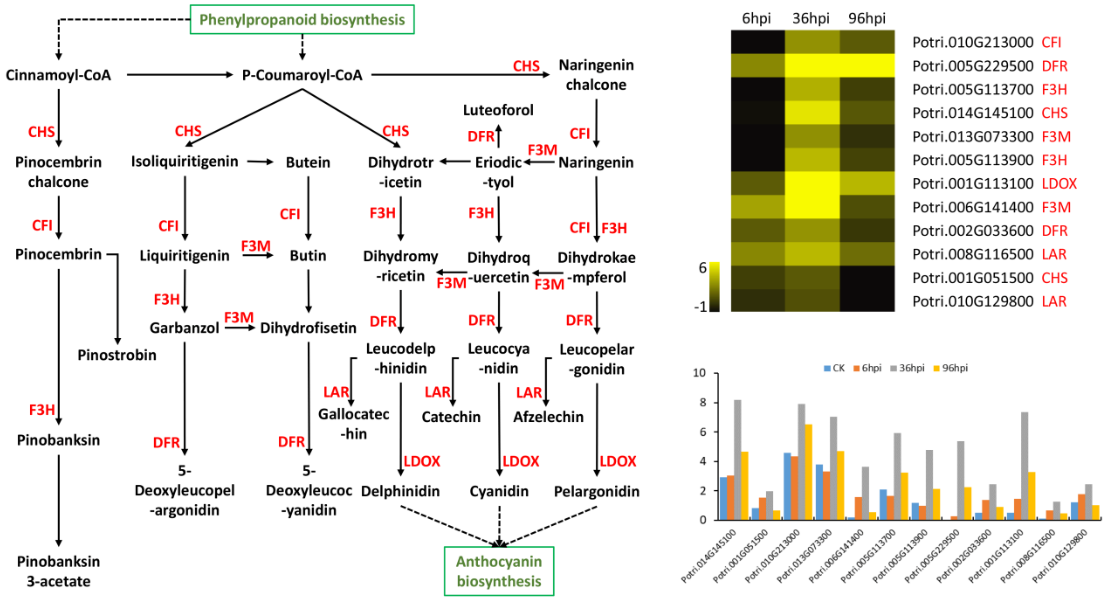

Supplementary Figure S6: The gene expression pattern of some genes involved in the flavonoid biosynthesis pathway of Aig.

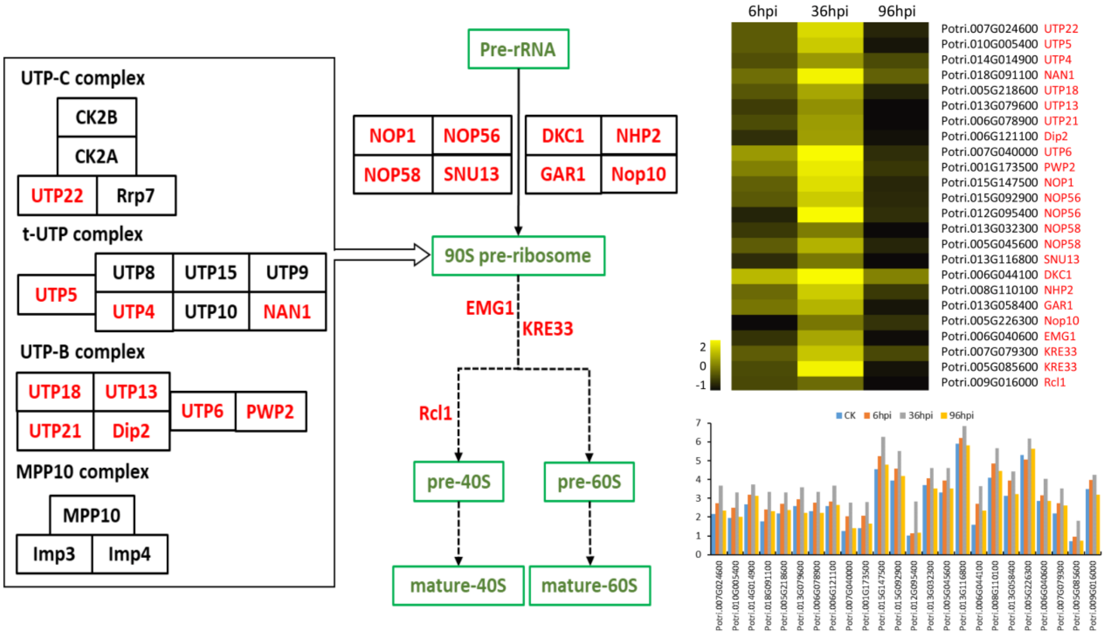

Supplementary Figure S7: The gene expression pattern of some genes involved in the ribosome biogenesis in eukaryotes pathway of Leu.

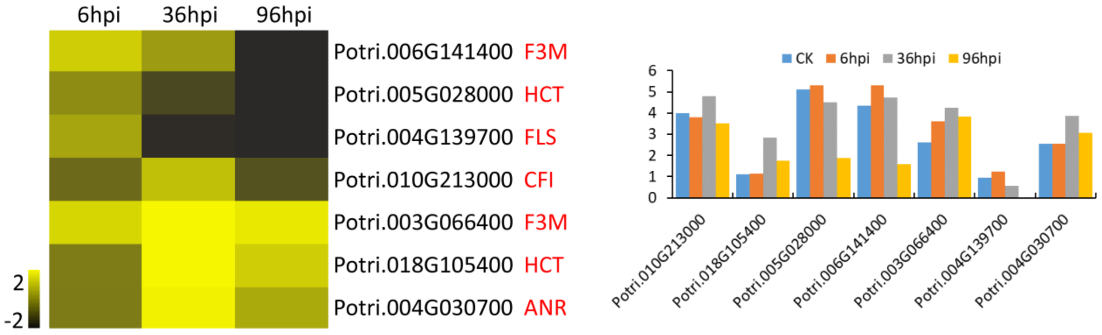

Supplementary Figure S8: The expression pattern of some genes involved in the flavonoid biosynthesis pathway of Leu.

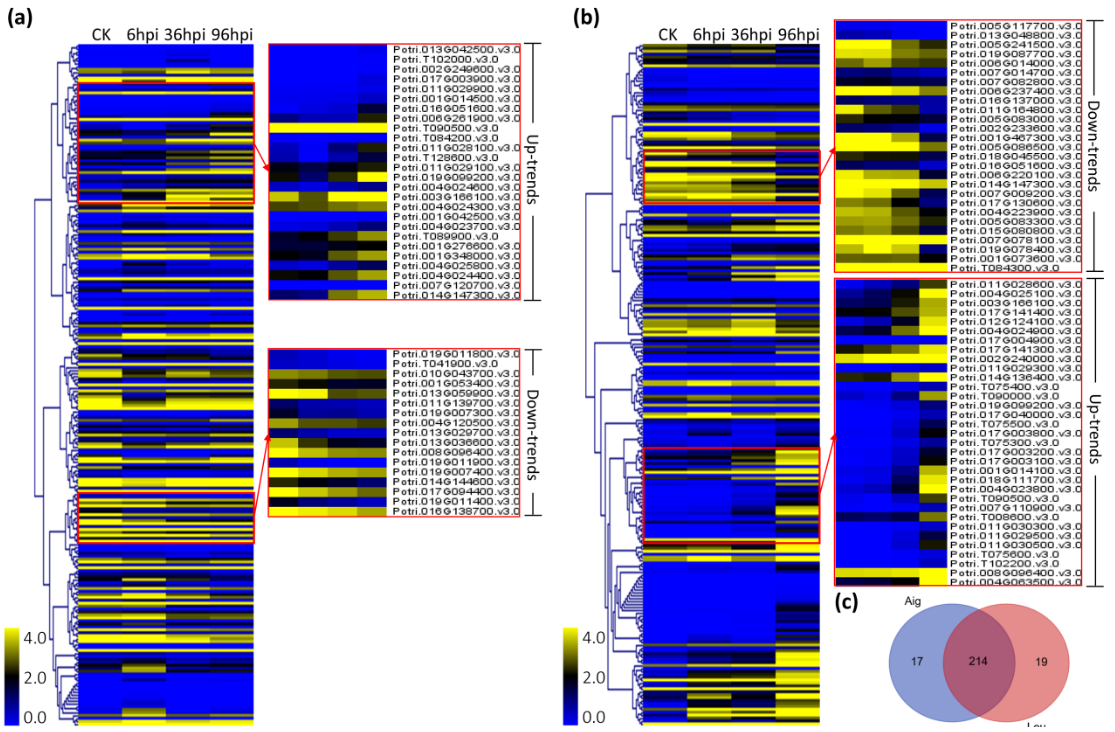

Supplementary Figure S9: Clustering of gene expression of genes encoding RLKs in two poplar sections.

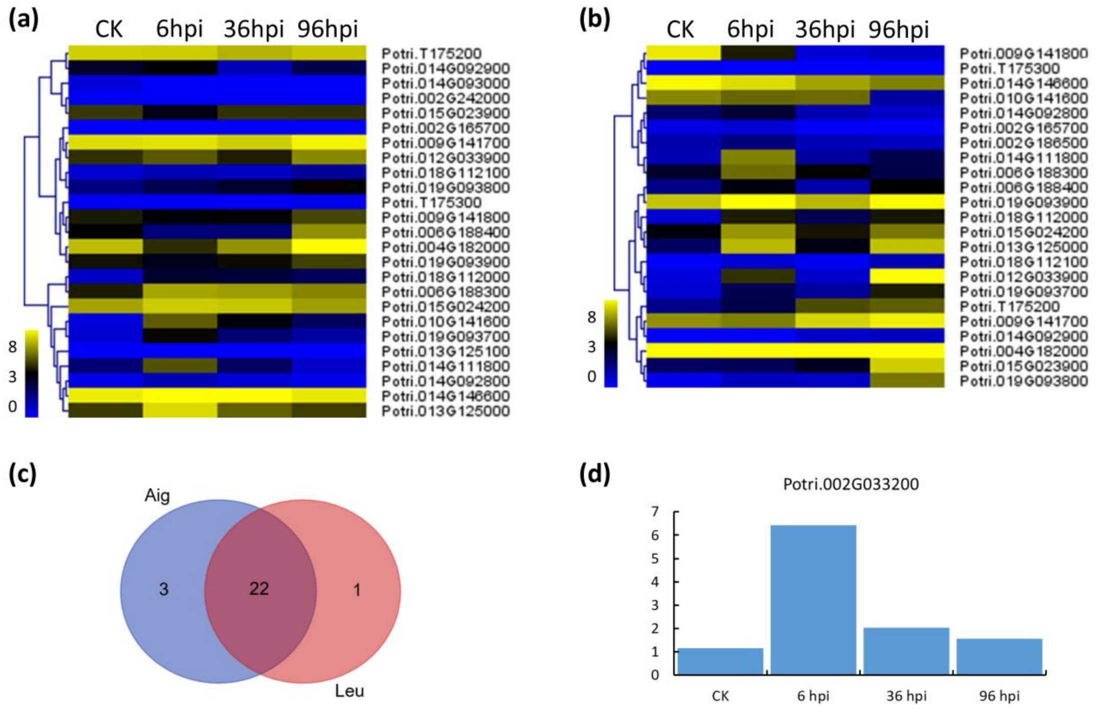

Supplementary Figure S10: Clustering of gene expression of genes encoding chitinases in two poplar sections.

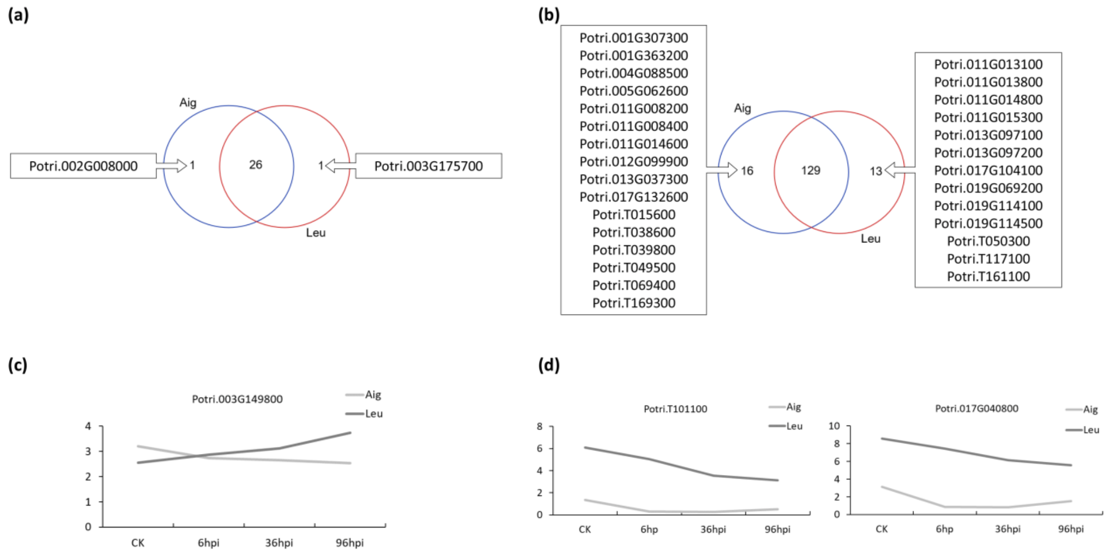

Supplementary Figure S11 Analysis of several genes played important roles in plant in response to pathogens.

Supplementary Table S1: RT-PCR primers used in this study.

Gene ID	Gene description	Forward primers (5’ → 3’)	Reverse primers (5’ → 3’)	Length of production
Potri.007G093700	60S	AGGTGAACTCTTGATGCTTCGTCTT	CTTCTCTTCCATTGCCTGTCCAACT	177
Potri.001G119900	SAUR-like auxin-responsive protein family	TGGAGGTACCCAAAGGACAC	AAACTCCGAATACCCACACG	185
Potri.004G230000	Disease resistance protein (TIR-NBS-LRR class) family	GCAATCCAAGAATCGCAAAT	ACCCTCTCCATGTCATCCTG	230
Potri.004G023800	CRK10, cysteine-rich RLK (RECEPTOR-like protein kinase) 10	GCCGCAGTTGCTATTTAAGG	TCTTGCATTTTCGCATTCTG	207
Potri.002G129100	MAPKKK5, mitogen-activated protein kinase kinase kinase 5	GATCCAAAATCTGCCGAATG	GGGTGAAATTGCGGACTATG	214
Potri.012G054700	Protein kinase superfamily protein	AAGGTTCGTATGGCTCTTGG	AGAAGAGATGGACGCCATTG	184
Potri.006G141400	TT7, Cytochrome P450 superfamily protein	ATTGAAACTGCCACCAGGTC	TACTTGCCAGCAGCAGTTTG	234
Potri.007G021400	S-adenosyl-L-methionine-dependent methyltransferases superfamily protein	CGCAGTTCCAAGAGGACTTC	TCATCCTTGGTTGGTGCATA	238
Potri.013G059900	RLK1, receptor-like protein kinase 1	GATCCAAGCATTCCCTCAAA	CATTATCGCCTTCCTGCATT	183
Potri.004G024900	CRK25, cysteine-rich RLK (RECEPTOR-like protein kinase) 25	CACTTGTGTCCAATGCAACC	CGCAAGAAGCACAAATCGTA	214
Potri.002G202600	Plant invertase/pectin methylesterase inhibitor superfamily	CGGTGATTACTGGGAATCGT	CTTCGAAGAGCTACAGCCTGA	151
Potri.004G019900	GPT2, glucose-6-phosphate/phosphate translocator 2	TCTTGGGCTACAAGGATTGC	AGGCTCGCCACTCTTGATTA	162
Potri.001G079900	ERF1, ethylene responsive element binding factor 1	GATGAGCTGGATCTGGAAGC	CTTTGCCGTCTCGTAAGTCC	207
Potri.004G024400	CRK10, cysteine-rich RLK (RECEPTOR-like protein kinase) 10	TGTCAGCAACAAAAGCCTTG	CCTAGCCAAGCCAAAATCTG	220
Potri.015G030700	MKK9, MAP kinase kinase 9	CCTCGCTTCCCTTTACCTCT	AGAATCTGTACGGCGAAGGA	249
Potri.005G043700	Leucine-rich repeat transmembrane protein kinase protein	CGGCAAACACCTCAATTTCT	AGGGAGACATGGATCACCAG	194
Potri.002G234000	NAD(P)-linked oxidoreductase superfamily protein	GCATTGATACCAGCGTTCCT	CAATCCCAATGCCAAGTTCT	225
Potri.001G255100	Glycosyl hydrolase superfamily protein	GCCATTGCTTATCCCACCTA	CATAACAAACCCCGATGGAT	172
Potri.019G130700	C4H, cinnamate-4-hydroxylase	GGCTGCAACTCTTCAAGGAC	GATGGTTCACAAGCTCAGCA	231

Supplementary Table S2: Details of GO terms enriched by up-regulated DEGs of Aig at **6 hpi** of three infection stages in Figure 4

Process go term	Description	Matched counts	Matched genes
GO:0009309	amine biosynthetic process	12	Potri.009G061700 Potri.001G300900 Potri.008G074500 Potri.002G236800 Potri.013G061800 Potri.009G096600 Potri.004G190900 Potri.008G099300 Potri.005G073300 Potri.002G099200 Potri.002G189200 Potri.006G062600
GO:0016051	carbohydrate biosynthetic process	25	Potri.004G117800 Potri.002G257900 Potri.006G052600 Potri.004G189900 Potri.004G059600 Potri.011G069600 Potri.018G103900 Potri.016G054900 Potri.014G125100 Potri.005G116200 Potri.013G082200 Potri.017G059100 Potri.007G127000 Potri.002G066600 Potri.015G126900 Potri.018G029400 Potri.011G103900 Potri.005G077200 Potri.001G237200 Potri.001G139500 Potri.006G181900 Potri.011G120100 Potri.012G047400 Potri.012G047300 Potri.001G320000
GO:0005975	carbohydrate metabolic process	78	Potri.007G099800 Potri.001G226000 Potri.010G141600 Potri.014G146100 Potri.001G227300 Potri.007G127000 Potri.014G122200 Potri.004G117800 Potri.019G032600 Potri.010G145800 Potri.002G023900 Potri.011G094400 Potri.013G082200 Potri.004G189900 Potri.015G126900 Potri.004G059600 Potri.014G158400 Potri.006G037300 Potri.001G071000 Potri.011G069600 Potri.014G157600 Potri.006G002100 Potri.018G103900 Potri.016G054900 Potri.016G043400 Potri.008G094200 Potri.005G116200 Potri.014G125100 Potri.006G235100 Potri.019G125000 Potri.013G060400 Potri.002G066600 Potri.017G059100 Potri.009G080600 Potri.013G125000 Potri.012G045900 Potri.001G171900 Potri.018G029400 Potri.018G095100 Potri.007G010000 Potri.001G320000 Potri.011G120100 Potri.001G449100 Potri.014G111800 Potri.011G015600 Potri.011G103900 Potri.005G201200 Potri.005G077200 Potri.004G021000 Potri.018G112000 Potri.003G159700 Potri.006G181900 Potri.002G257900 Potri.016G057400 Potri.001G237200 Potri.011G152400 Potri.006G052600 Potri.001G139500 Potri.011G156100 Potri.002G202400 Potri.005G007200 Potri.002G014300 Potri.002G236200 Potri.001G061100 Potri.005G257800 Potri.010G159900 Potri.018G024500 Potri.005G167800 Potri.012G047400 Potri.012G047300 Potri.005G237700 Potri.001G299000 Potri.006G188300 Potri.007G018100 Potri.006G062600 Potri.015G041300 Potri.019G093700 Potri.006G071200
GO:0046394	carboxylic acid biosynthetic process	21	Potri.004G190900 Potri.001G300900 Potri.001G463800 Potri.011G010400 Potri.018G032200 Potri.010G212500 Potri.008G074500 Potri.006G249200 Potri.002G236800 Potri.013G061800 Potri.009G096600 Potri.009G116700 Potri.008G099300 Potri.006G095800 Potri.014G180300 Potri.005G073300 Potri.009G061700 Potri.002G099200 Potri.002G189200 Potri.006G062600 Potri.010G212600
GO:0007154	cell communication	14	Potri.011G037300 Potri.004G027400 Potri.011G128700 Potri.011G039100 Potri.001G412000 Potri.004G027800 Potri.006G053700 Potri.001G413700 Potri.013G086100 Potri.011G037100 Potri.T022600 Potri.011G125200 Potri.015G105500 Potri.T023900
GO:0008037	cell recognition	13	Potri.011G037300 Potri.004G027400 Potri.011G128700 Potri.011G039100 Potri.001G412000 Potri.004G027800 Potri.006G053700 Potri.001G413700 Potri.013G086100 Potri.011G037100 Potri.T022600 Potri.011G125200

			Potri.T023900
GO:0044036	cell wall macromolecule metabolic process	8	Potri.013G102300 Potri.010G141600 Potri.008G030200 Potri.014G111800 Potri.002G152600 Potri.004G183500 Potri.013G125000 Potri.019G093700
GO:0071554	cell wall organization or biogenesis	16	Potri.013G102300 Potri.010G141600 Potri.011G135000 Potri.014G149700 Potri.015G013700 Potri.002G202600 Potri.004G117800 Potri.008G030200 Potri.014G111800 Potri.002G145500 Potri.004G183500 Potri.002G152600 Potri.003G191200 Potri.002G202500 Potri.013G125000 Potri.019G093700
GO:0044262	cellular carbohydrate metabolic process	47	Potri.004G117800 Potri.010G145800 Potri.006G062600 Potri.006G052600 Potri.004G189900 Potri.004G059600 Potri.001G071000 Potri.011G069600 Potri.016G043400 Potri.016G054900 Potri.005G116200 Potri.014G125100 Potri.006G235100 Potri.019G125000 Potri.013G082200 Potri.017G059100 Potri.009G080600 Potri.018G024500 Potri.012G045900 Potri.002G066600 Potri.019G032600 Potri.015G126900 Potri.018G095100 Potri.018G029400 Potri.006G037300 Potri.004G021000 Potri.005G007200 Potri.011G015600 Potri.011G103900 Potri.005G201200 Potri.005G077200 Potri.002G257900 Potri.014G146100 Potri.006G181900 Potri.001G237200 Potri.013G060400 Potri.001G139500 Potri.011G156100 Potri.011G120100 Potri.002G236200 Potri.001G061100 Potri.005G257800 Potri.003G159700 Potri.018G103900 Potri.007G127000 Potri.001G320000 Potri.006G071200
GO:0006073	cellular glucan metabolic process	22	Potri.004G117800 Potri.002G257900 Potri.006G052600 Potri.004G059600 Potri.001G071000 Potri.011G069600 Potri.016G054900 Potri.014G125100 Potri.019G125000 Potri.013G082200 Potri.002G066600 Potri.018G095100 Potri.018G029400 Potri.004G021000 Potri.005G007200 Potri.005G201200 Potri.014G146100 Potri.006G181900 Potri.002G236200 Potri.003G159700 Potri.018G103900 Potri.006G071200
GO:0044237	cellular metabolic process	322	Potri.010G103100 Potri.001G121400 Potri.001G246400 Potri.013G116400 Potri.011G057000 Potri.005G195000 Potri.001G467300 Potri.005G095400 Potri.019G008900 Potri.002G033600 Potri.008G099300 Potri.006G095800 Potri.014G164700 Potri.014G180300 Potri.010G074300 Potri.001G161600 Potri.010G188600 Potri.007G017100 Potri.019G028700 Potri.013G086100 Potri.002G030900 Potri.009G081800 Potri.014G155000 Potri.011G139800 Potri.001G046100 Potri.001G412000 Potri.005G116200 Potri.004G095700 Potri.011G153300 Potri.007G067900 Potri.003G080600 Potri.001G218100 Potri.013G059600 Potri.006G051700 Potri.004G047600 Potri.017G059100 Potri.014G012800 Potri.001G020900 Potri.012G047300 Potri.T148900 Potri.004G099400 Potri.009G020400 Potri.001G352400 Potri.T003400 Potri.004G051700 Potri.008G074500 Potri.002G070500 Potri.016G134000 Potri.011G010500 Potri.014G126100 Potri.001G372300 Potri.011G103900 Potri.003G061700 Potri.011G163700 Potri.003G183100 Potri.012G042600 Potri.002G189200 Potri.001G237200 Potri.007G147300 Potri.004G027400 Potri.006G052600 Potri.005G057500 Potri.003G107600 Potri.001G042400 Potri.011G128700 Potri.001G216100 Potri.005G257800 Potri.010G113900 Potri.014G136300 Potri.009G116700 Potri.016G043400 Potri.018G057000 Potri.001G145700 Potri.016G069500 Potri.016G028700 Potri.004G185000 Potri.001G448400 Potri.014G036600 Potri.019G018500 Potri.009G139800 Potri.001G385600 Potri.014G050400 Potri.018G045500 Potri.017G031600 Potri.010G212500 Potri.006G105300 Potri.008G160200 Potri.008G102500 Potri.005G111700 Potri.006G053700 Potri.006G062600 Potri.010G097700 Potri.006G079600 Potri.011G049600 Potri.019G073300 Potri.004G015500

			Potri.002G045000 Potri.001G452200 Potri.006G037300 Potri.011G037300 Potri.004G190900 Potri.004G205000<br>Potri.001G300900 Potri.006G099100 Potri.011G058300 Potri.016G054900 Potri.006G249200 Potri.010G123500<br>Potri.014G104800 Potri.018G083600 Potri.013G061800 Potri.008G137700 Potri.008G148200 Potri.009G119700<br>Potri.001G044500 Potri.002G099200 Potri.009G080600 Potri.006G071600 Potri.007G135100 Potri.001G046400<br>Potri.009G118300 Potri.014G017200 Potri.004G014700 Potri.009G061700 Potri.011G028800 Potri.011G010400<br>Potri.002G065600 Potri.003G117500 Potri.005G073300 Potri.018G029400 Potri.011G120100 Potri.007G044800<br>Potri.007G047900 Potri.011G015600 Potri.002G236800 Potri.005G201200 Potri.005G077200 Potri.004G021000<br>Potri.017G016700 Potri.009G040200 Potri.006G066100 Potri.004G065400 Potri.001G139500 Potri.011G156100<br>Potri.010G221200 Potri.005G223100 Potri.005G181800 Potri.002G023400 Potri.014G068700 Potri.002G142800<br>Potri.016G128300 Potri.003G093400 Potri.005G218200 Potri.001G071000 Potri.012G047400 Potri.001G099000<br>Potri.T022600 Potri.015G043400 Potri.009G010000 Potri.002G034400 Potri.003G182200 Potri.006G202700<br>Potri.019G078400 Potri.006G148800 Potri.012G108500 Potri.004G135500 Potri.016G144100 Potri.003G156500<br>Potri.004G209300 Potri.006G025800 Potri.004G117800 Potri.002G257900 Potri.011G106400 Potri.009G009600<br>Potri.019G032600 Potri.001G463800 Potri.007G048800 Potri.005G087200 Potri.002G019300 Potri.011G037700<br>Potri.014G101100 Potri.010G223300 Potri.018G043900 Potri.007G023600 Potri.003G167700 Potri.004G051800<br>Potri.011G069600 Potri.002G201600 Potri.018G134100 Potri.014G125100 Potri.002G105300 Potri.001G343100<br>Potri.006G235100 Potri.010G087900 Potri.004G226900 Potri.T157700 Potri.002G036200 Potri.019G125000<br>Potri.014G146100 Potri.001G104600 Potri.002G066600 Potri.012G033200 Potri.001G217700 Potri.012G054700<br>Potri.004G026400 Potri.017G135000 Potri.018G024500 Potri.012G055700 Potri.001G044400 Potri.008G038900<br>Potri.018G032200 Potri.013G057900 Potri.001G280200 Potri.013G082200 Potri.004G027800 Potri.011G169600<br>Potri.006G072400 Potri.018G138700 Potri.013G064300 Potri.002G240800 Potri.005G141400 Potri.009G145000<br>Potri.010G212600 Potri.006G208100 Potri.002G198000 Potri.011G068500 Potri.008G140500 Potri.002G251700<br>Potri.T052100 Potri.002G100600 Potri.004G139900 Potri.018G095100 Potri.012G031700 Potri.001G061100<br>Potri.009G096600 Potri.006G181900 Potri.007G140800 Potri.012G124200 Potri.001G270000 Potri.006G143500<br>Potri.009G100400 Potri.017G088200 Potri.007G110600 Potri.013G003700 Potri.001G216300 Potri.001G289500<br>Potri.001G168200 Potri.005G215600 Potri.004G154000 Potri.005G051600 Potri.004G059600 Potri.009G010400<br>Potri.010G145800 Potri.019G048800 Potri.010G004200 Potri.004G175100 Potri.004G189900 Potri.001G092900<br>Potri.016G072300 Potri.004G005300 Potri.006G236200 Potri.019G057300 Potri.012G032900 Potri.003G187000<br>Potri.004G083900 Potri.014G106300 Potri.015G093100 Potri.002G009300 Potri.006G146300 Potri.016G050500<br>Potri.008G081000 Potri.009G016100 Potri.009G118100 Potri.009G140300 Potri.001G413700 Potri.006G202600<br>Potri.011G037100 Potri.005G187600 Potri.013G060400 Potri.T023800 Potri.001G310500 Potri.010G137300<br>Potri.012G045900 Potri.006G070500 Potri.005G189500 Potri.018G045100 Potri.011G067400 Potri.015G126900<br>Potri.T096300 Potri.016G126300 Potri.009G027000 Potri.001G095200 Potri.015G104800 Potri.014G195200<br>Potri.011G125200 Potri.006G188600 Potri.016G070500 Potri.006G193700 Potri.012G134000 Potri.005G007200

			Potri.007G007400 Potri.007G127000 Potri.008G067300 Potri.015G110400 Potri.008G077900 Potri.012G021700 Potri.006G219600 Potri.002G236200 Potri.019G004700 Potri.011G039100 Potri.003G196000 Potri.016G069400 Potri.003G159700 Potri.018G103900 Potri.018G138800 Potri.013G036300 Potri.001G320000 Potri.011G109800 Potri.014G034700 Potri.003G100200 Potri.004G055700 Potri.006G071200
GO:0044264	cellular polysaccharide metabolic process	29	Potri.004G117800 Potri.002G257900 Potri.006G052600 Potri.004G189900 Potri.004G059600 Potri.001G071000 Potri.011G069600 Potri.016G054900 Potri.014G125100 Potri.005G116200 Potri.019G125000 Potri.013G082200 Potri.017G059100 Potri.007G127000 Potri.002G066600 Potri.018G095100 Potri.018G029400 Potri.004G021000 Potri.005G007200 Potri.005G201200 Potri.014G146100 Potri.001G237200 Potri.006G181900 Potri.011G120100 Potri.002G236200 Potri.003G159700 Potri.018G103900 Potri.001G320000 Potri.006G071200
GO:0044267	cellular protein metabolic process	129	Potri.010G103100 Potri.001G246400 Potri.013G116400 Potri.001G467300 Potri.019G008900 Potri.004G065400 Potri.001G161600 Potri.019G028700 Potri.009G081800 Potri.011G139800 Potri.006G202600 Potri.001G412000 Potri.019G078400 Potri.007G048800 Potri.010G188600 Potri.007G067900 Potri.001G218100 Potri.006G051700 Potri.004G154000 Potri.009G020400 Potri.T003400 Potri.002G070500 Potri.016G134000 Potri.011G010500 Potri.011G163700 Potri.012G042600 Potri.004G027400 Potri.005G057500 Potri.001G042400 Potri.011G128700 Potri.001G216100 Potri.014G136300 Potri.016G069500 Potri.009G027000 Potri.001G280200 Potri.001G385600 Potri.018G045500 Potri.005G189500 Potri.011G169600 Potri.006G053700 Potri.010G097700 Potri.006G079600 Potri.011G049600 Potri.011G037300 Potri.004G205000 Potri.006G099100 Potri.011G058300 Potri.008G160200 Potri.018G083600 Potri.008G137700 Potri.003G107600 Potri.003G183100 Potri.007G127000 Potri.004G014700 Potri.011G028800 Potri.004G015500 Potri.003G117500 Potri.007G044800 Potri.007G047900 Potri.T022600 Potri.006G066100 Potri.010G221200 Potri.005G181800 Potri.014G068700 Potri.019G048800 Potri.012G047400 Potri.013G036300 Potri.006G202700 Potri.004G135500 Potri.016G144100 Potri.004G209300 Potri.010G113900 Potri.011G106400 Potri.009G009600 Potri.009G100400 Potri.004G095700 Potri.002G019300 Potri.011G037700 Potri.014G101100 Potri.014G155000 Potri.010G087900 Potri.009G010400 Potri.002G036200 Potri.012G033200 Potri.012G054700 Potri.004G026400 Potri.017G135000 Potri.018G024500 Potri.004G027800 Potri.018G138700 Potri.013G064300 Potri.002G198000 Potri.011G068500 Potri.008G140500 Potri.002G251700 Potri.004G175100 Potri.007G140800 Potri.012G124200 Potri.017G088200 Potri.007G110600 Potri.013G003700 Potri.001G216300 Potri.011G039100 Potri.003G167700 Potri.004G005300 Potri.004G226900 Potri.004G083900 Potri.016G070500 Potri.015G093100 Potri.002G009300 Potri.006G146300 Potri.009G016100 Potri.009G140300 Potri.001G413700 Potri.011G037100 Potri.001G217700 Potri.T023800 Potri.011G067400 Potri.016G126300 Potri.001G095200 Potri.014G195200 Potri.018G134100 Potri.012G021700 Potri.019G004700 Potri.005G051600 Potri.013G086100 Potri.018G138800 Potri.011G125200 Potri.016G069400
GO:0006855	multidrug transport	5	Potri.002G102100 Potri.016G053600 Potri.003G121400 Potri.019G086100 Potri.005G102800
GO:0006091	generation of precursor metabolites and energy	7	Potri.019G032600 Potri.013G060400 Potri.005G257800 Potri.011G015600 Potri.006G235100 Potri.009G010000 Potri.008G067300

GO:0006629	lipid metabolic process	54	Potri.001G463800 Potri.002G165800 Potri.T095600 Potri.010G212500 Potri.001G263200 Potri.007G127000<br>Potri.017G133800 Potri.002G033600 Potri.004G189900 Potri.009G116700 Potri.001G173700 Potri.017G133900<br>Potri.017G134100 Potri.018G032200 Potri.001G046100 Potri.001G252100 Potri.004G054600 Potri.006G249200<br>Potri.013G116400 Potri.001G252900 Potri.014G180300 Potri.013G153000 Potri.T157700 Potri.002G034400<br>Potri.005G187600 Potri.017G059100 Potri.010G236800 Potri.001G046400 Potri.014G160100 Potri.011G010400<br>Potri.019G024700 Potri.006G228200 Potri.009G057900 Potri.015G104800 Potri.003G196000 Potri.003G061700<br>Potri.001G191400 Potri.011G156100 Potri.015G145900 Potri.010G212600 Potri.001G237200 Potri.008G077900<br>Potri.011G120100 Potri.004G139900 Potri.002G128300 Potri.012G047400 Potri.006G095800 Potri.015G043400<br>Potri.009G118300 Potri.016G117500 Potri.001G320000 Potri.011G089700 Potri.003G100200 Potri.008G068400
GO:0043170	macromolecule metabolic process	284	Potri.010G103100 Potri.014G146100 Potri.001G246400 Potri.013G116400 Potri.011G057000 Potri.005G195000<br>Potri.019G064800 Potri.001G467300 Potri.005G095400 Potri.019G008900 Potri.005G257800 Potri.002G030900<br>Potri.001G158600 Potri.010G074300 Potri.001G161600 Potri.010G188600 Potri.019G028700 Potri.013G086100<br>Potri.009G081800 Potri.014G155000 Potri.011G139800 Potri.006G202600 Potri.001G412000 Potri.005G116200<br>Potri.004G095700 Potri.011G153300 Potri.007G067900 Potri.006G025800 Potri.001G218100 Potri.013G059600<br>Potri.006G051700 Potri.002G152600 Potri.017G059100 Potri.014G012800 Potri.012G047300 Potri.T148900<br>Potri.004G099400 Potri.008G058000 Potri.001G352400 Potri.006G236200 Potri.004G051700 Potri.008G081000<br>Potri.005G063000 Potri.016G134000 Potri.011G010500 Potri.005G007200 Potri.001G440300 Potri.014G126100<br>Potri.001G372300 Potri.011G163700 Potri.003G183100 Potri.012G042600 Potri.001G237200 Potri.011G120100<br>Potri.004G027400 Potri.006G052600 Potri.005G057500 Potri.003G107600 Potri.001G042400 Potri.011G128700<br>Potri.001G216100 Potri.010G113900 Potri.014G136300 Potri.011G169600 Potri.016G043400 Potri.018G057000<br>Potri.016G069500 Potri.014G074600 Potri.001G448400 Potri.014G036600 Potri.005G218200 Potri.009G139800<br>Potri.001G385600 Potri.018G045500 Potri.017G031600 Potri.006G148800 Potri.004G014700 Potri.006G105300<br>Potri.008G160200 Potri.008G102500 Potri.005G111700 Potri.006G053700 Potri.006G062600 Potri.010G097700<br>Potri.014G018900 Potri.006G079600 Potri.011G049600 Potri.019G073300 Potri.002G045000 Potri.001G452200<br>Potri.011G037300 Potri.004G205000 Potri.006G099100 Potri.011G058300 Potri.009G002200 Potri.010G223300<br>Potri.010G123500 Potri.004G047600 Potri.018G083600 Potri.001G163600 Potri.008G137700 Potri.008G148200<br>Potri.009G119700 Potri.018G095100 Potri.011G066800 Potri.009G080600 Potri.006G071600 Potri.007G135100<br>Potri.013G125000 Potri.007G127000 Potri.013G102300 Potri.011G028800 Potri.004G015500 Potri.002G065600<br>Potri.003G117500 Potri.001G044500 Potri.018G029400 Potri.007G044800 Potri.007G047900 Potri.011G015600<br>Potri.016G128300 Potri.005G201200 Potri.004G021000 Potri.017G016700 Potri.007G035900 Potri.006G066100<br>Potri.004G065400 Potri.010G221200 Potri.005G223100 Potri.005G181800 Potri.002G023400 Potri.014G068700<br>Potri.002G142800 Potri.019G048800 Potri.003G093400 Potri.001G071000 Potri.012G047400 Potri.001G099000<br>Potri.T022600 Potri.013G036300 Potri.003G182200 Potri.006G202700 Potri.019G078400 Potri.012G108500<br>Potri.004G135500 Potri.016G144100 Potri.003G156500 Potri.004G209300 Potri.002G092100 Potri.003G080600

			Potri.006G068900 Potri.004G117800 Potri.002G257900 Potri.011G106400 Potri.009G009600 Potri.019G032600 Potri.009G100400 Potri.007G048800 Potri.005G087200 Potri.002G019300 Potri.011G037700 Potri.019G002100 Potri.014G101100 Potri.004G183500 Potri.018G043900 Potri.007G023600 Potri.003G167700 Potri.004G051800 Potri.011G069600 Potri.010G220100 Potri.014G125100 Potri.001G343100 Potri.006G235100 Potri.010G087900 Potri.004G226900 Potri.009G010400 Potri.002G036200 Potri.019G125000 Potri.012G055700 Potri.002G066600 Potri.012G033200 Potri.012G054700 Potri.004G026400 Potri.017G135000 Potri.018G024500 Potri.001G044400 Potri.008G038900 Potri.001G280200 Potri.013G082200 Potri.004G027800 Potri.010G201400 Potri.006G072400 Potri.018G138700 Potri.013G064300 Potri.013G060400 Potri.005G141400 Potri.006G181900 Potri.002G124500 Potri.006G208100 Potri.002G198000 Potri.011G068500 Potri.005G204600 Potri.008G140500 Potri.002G251700 Potri.T052100 Potri.002G100600 Potri.012G031700 Potri.018G015100 Potri.007G140800 Potri.012G124200 Potri.001G270000 Potri.006G143500 Potri.017G088200 Potri.007G110600 Potri.013G003700 Potri.001G216300 Potri.018G014600 Potri.010G141600 Potri.008G203200 Potri.002G201600 Potri.004G154000 Potri.005G051600 Potri.004G059600 Potri.009G020400 Potri.010G004200 Potri.004G175100 Potri.004G189900 Potri.001G028200 Potri.001G092900 Potri.016G072300 Potri.004G005300 Potri.T003400 Potri.017G084000 Potri.003G076300 Potri.004G083900 Potri.014G106300 Potri.004G215400 Potri.015G093100 Potri.002G009300 Potri.018G103900 Potri.016G050500 Potri.009G016100 Potri.014G164700 Potri.009G140300 Potri.001G413700 Potri.011G037100 Potri.012G032900 Potri.001G217700 Potri.T023800 Potri.010G137300 Potri.002G054900 Potri.005G189500 Potri.018G045100 Potri.011G067400 Potri.002G070500 Potri.016G126300 Potri.009G027000 Potri.001G095200 Potri.002G105300 Potri.014G195200 Potri.011G125200 Potri.016G070500 Potri.006G193700 Potri.012G134000 Potri.014G111800 Potri.007G007400 Potri.018G134100 Potri.008G030200 Potri.016G034300 Potri.012G021700 Potri.002G236200 Potri.019G004700 Potri.006G146300 Potri.011G039100 Potri.016G069400 Potri.003G159700 Potri.016G054900 Potri.018G138800 Potri.011G076700 Potri.001G320000 Potri.014G104800 Potri.004G055700 Potri.019G093700 Potri.006G071200
GO:0007018	microtubule-based movement	15	Potri.001G360200 Potri.009G122100 Potri.014G125700 Potri.010G227000 Potri.011G165200 Potri.007G014800 Potri.002G201000 Potri.002G235500 Potri.005G021100 Potri.001G455300 Potri.003G053800 Potri.011G146700 Potri.001G000800 Potri.001G416300 Potri.001G233700
GO:0051704	multi-organism process	16	Potri.011G037300 Potri.004G027400 Potri.011G128700 Potri.011G039100 Potri.001G412000 Potri.004G027800 Potri.006G053700 Potri.001G413700 Potri.013G086100 Potri.012G047400 Potri.011G037100 Potri.T022600 Potri.005G054000 Potri.011G125200 Potri.012G047300 Potri.T023900
GO:0055114	oxidation reduction	134	Potri.001G365400 Potri.007G108400 Potri.016G125000 Potri.002G165800 Potri.T160100 Potri.002G033600 Potri.001G113900 Potri.006G141400 Potri.001G331100 Potri.007G017100 Potri.001G362600 Potri.010G096800 Potri.001G046100 Potri.002G156000 Potri.012G006800 Potri.008G073800 Potri.018G063300 Potri.001G007100 Potri.003G159800 Potri.017G059100 Potri.006G087100 Potri.001G113100 Potri.004G140900 Potri.008G064000 Potri.008G074500 Potri.002G191900 Potri.016G102300 Potri.001G237200 Potri.007G147300 Potri.001G307500

			Potri.016G132700 Potri.011G156000 Potri.009G107600 Potri.016G043400 Potri.007G108600 Potri.001G046400 Potri.016G117500 Potri.001G459500 Potri.004G017700 Potri.013G007800 Potri.003G112700 Potri.007G115500 Potri.006G228200 Potri.002G065300 Potri.008G069300 Potri.009G096600 Potri.010G193100 Potri.019G121700 Potri.001G300900 Potri.014G180300 Potri.010G023600 Potri.006G096900 Potri.009G080600 Potri.001G378400 Potri.009G118300 Potri.004G106600 Potri.018G046600 Potri.007G053400 Potri.006G137300 Potri.T107300 Potri.006G151600 Potri.016G132800 Potri.013G064200 Potri.011G156100 Potri.019G064600 Potri.004G146000 Potri.011G158700 Potri.001G111500 Potri.009G010000 Potri.015G003500 Potri.005G247700 Potri.T069600 Potri.001G252900 Potri.010G055400 Potri.001G229500 Potri.T107400 Potri.005G138400 Potri.001G268600 Potri.019G064200 Potri.008G161600 Potri.001G003100 Potri.015G110400 Potri.013G106200 Potri.001G458700 Potri.005G043400 Potri.016G117100 Potri.002G254200 Potri.002G040700 Potri.001G364900 Potri.001G440700 Potri.018G033400 Potri.007G033300 Potri.008G073700 Potri.008G158300 Potri.006G062600 Potri.002G013700 Potri.001G020900 Potri.017G075100 Potri.T052100 Potri.011G155600 Potri.018G051300 Potri.018G134300 Potri.001G219300 Potri.007G122100 Potri.009G145400 Potri.001G463800 Potri.018G146100 Potri.008G198600 Potri.004G189900 Potri.013G074700 Potri.019G057300 Potri.006G129900 Potri.002G034400 Potri.005G187600 Potri.004G235400 Potri.011G152800 Potri.001G351000 Potri.001G015400 Potri.006G101100 Potri.004G118600 Potri.016G112000 Potri.015G104800 Potri.006G004500 Potri.008G094300 Potri.005G108900 Potri.001G470100 Potri.005G143900 Potri.008G077900 Potri.014G143200 Potri.011G120200 Potri.004G015300 Potri.001G320000 Potri.009G107700 Potri.003G100200
GO:0006793	phosphorus metabolic process	104	Potri.001G467300 Potri.019G008900 Potri.004G065400 Potri.001G246400 Potri.009G081800 Potri.011G139800 Potri.001G412000 Potri.019G078400 Potri.007G048800 Potri.010G188600 Potri.007G067900 Potri.001G218100 Potri.006G051700 Potri.004G154000 Potri.009G020400 Potri.T003400 Potri.011G010500 Potri.011G163700 Potri.004G027400 Potri.001G042400 Potri.011G128700 Potri.014G136300 Potri.016G043400 Potri.001G280200 Potri.001G385600 Potri.018G045500 Potri.005G189500 Potri.011G169600 Potri.006G053700 Potri.001G161600 Potri.006G079600 Potri.011G049600 Potri.011G037300 Potri.006G099100 Potri.011G058300 Potri.008G160200 Potri.003G107600 Potri.003G183100 Potri.007G127000 Potri.004G014700 Potri.011G028800 Potri.004G015500 Potri.003G117500 Potri.007G044800 Potri.007G047900 Potri.T022600 Potri.006G066100 Potri.010G221200 Potri.005G181800 Potri.014G068700 Potri.019G048800 Potri.013G003700 Potri.009G010000 Potri.004G135500 Potri.016G144100 Potri.004G209300 Potri.011G106400 Potri.009G009600 Potri.009G100400 Potri.004G095700 Potri.002G019300 Potri.011G037700 Potri.014G155000 Potri.010G087900 Potri.009G010400 Potri.002G036200 Potri.012G033200 Potri.012G054700 Potri.004G026400 Potri.004G027800 Potri.018G138700 Potri.013G064300 Potri.011G068500 Potri.008G140500 Potri.002G251700 Potri.010G097700 Potri.014G050400 Potri.007G140800 Potri.012G124200 Potri.017G088200 Potri.019G004700 Potri.011G039100 Potri.004G175100 Potri.004G005300 Potri.004G226900 Potri.015G093100 Potri.002G009300 Potri.009G140300 Potri.001G413700 Potri.011G037100 Potri.001G217700 Potri.T023800 Potri.006G070500 Potri.011G067400 Potri.016G126300 Potri.001G095200

			Potri.014G195200 Potri.018G134100 Potri.016G070500 Potri.005G051600 Potri.013G086100 Potri.018G138800 Potri.013G036300 Potri.011G125200
GO:0016310	phosphorylation	102	Potri.001G467300 Potri.019G008900 Potri.004G065400 Potri.001G246400 Potri.009G081800 Potri.011G139800 Potri.001G412000 Potri.019G078400 Potri.007G048800 Potri.010G188600 Potri.007G067900 Potri.001G218100 Potri.006G051700 Potri.004G154000 Potri.009G020400 Potri.T003400 Potri.011G010500 Potri.011G163700 Potri.004G027400 Potri.001G042400 Potri.011G128700 Potri.014G136300 Potri.001G280200 Potri.001G385600 Potri.018G045500 Potri.005G189500 Potri.011G169600 Potri.006G053700 Potri.001G161600 Potri.006G079600 Potri.011G049600 Potri.011G037300 Potri.006G099100 Potri.011G058300 Potri.008G160200 Potri.003G107600 Potri.003G183100 Potri.007G127000 Potri.004G014700 Potri.011G028800 Potri.004G015500 Potri.003G117500 Potri.007G044800 Potri.T022600 Potri.006G066100 Potri.010G221200 Potri.005G181800 Potri.014G068700 Potri.019G048800 Potri.013G003700 Potri.009G010000 Potri.004G135500 Potri.016G144100 Potri.004G209300 Potri.011G106400 Potri.009G009600 Potri.009G100400 Potri.004G095700 Potri.002G019300 Potri.011G037700 Potri.014G155000 Potri.010G087900 Potri.009G010400 Potri.002G036200 Potri.012G033200 Potri.012G054700 Potri.004G026400 Potri.004G027800 Potri.018G138700 Potri.013G064300 Potri.011G068500 Potri.008G140500 Potri.002G251700 Potri.010G097700 Potri.014G050400 Potri.007G140800 Potri.012G124200 Potri.017G088200 Potri.019G004700 Potri.011G039100 Potri.004G175100 Potri.004G005300 Potri.004G226900 Potri.015G093100 Potri.002G009300 Potri.009G140300 Potri.001G413700 Potri.011G037100 Potri.001G217700 Potri.T023800 Potri.006G070500 Potri.011G067400 Potri.016G126300 Potri.001G095200 Potri.014G195200 Potri.018G134100 Potri.016G070500 Potri.005G051600 Potri.013G086100 Potri.018G138800 Potri.013G036300 Potri.011G125200
GO:0005976	polysaccharide metabolic process	33	Potri.010G141600 Potri.004G117800 Potri.002G257900 Potri.006G052600 Potri.004G189900 Potri.004G059600 Potri.001G071000 Potri.011G069600 Potri.016G054900 Potri.014G125100 Potri.005G116200 Potri.019G125000 Potri.013G082200 Potri.017G059100 Potri.013G125000 Potri.007G127000 Potri.002G066600 Potri.018G095100 Potri.018G029400 Potri.004G021000 Potri.014G111800 Potri.005G201200 Potri.014G146100 Potri.005G007200 Potri.001G237200 Potri.006G181900 Potri.011G120100 Potri.002G236200 Potri.003G159700 Potri.018G103900 Potri.001G320000 Potri.019G093700 Potri.006G071200
GO:0044238	primary metabolic process	386	Potri.019G064800 Potri.019G008900 Potri.002G033600 Potri.008G099300 Potri.006G095800 Potri.001G173700 Potri.001G161600 Potri.009G081800 Potri.011G139800 Potri.001G412000 Potri.004G095700 Potri.011G153300 Potri.014G125100 Potri.001G218100 Potri.013G059600 Potri.017G059100 Potri.T148900 Potri.T003400 Potri.019G024700 Potri.008G074500 Potri.016G134000 Potri.011G103900 Potri.003G061700 Potri.001G191400 Potri.011G163700 Potri.001G042400 Potri.011G128700 Potri.014G136300 Potri.004G190900 Potri.014G074600 Potri.010G212500 Potri.011G094400 Potri.006G079600 Potri.001G452200 Potri.011G037300 Potri.006G249200 Potri.018G083600 Potri.013G061800 Potri.008G137700 Potri.003G107600 Potri.018G095100 Potri.011G066800 Potri.011G010400 Potri.007G047900 Potri.018G112000 Potri.003G093400 Potri.012G047400 Potri.015G041300 Potri.004G135500 Potri.002G257900 Potri.011G106400 Potri.002G019300 Potri.019G002100 Potri.005G215600

			Potri.001G252900 Potri.009G010400 Potri.001G104600 Potri.002G066600 Potri.012G033200 Potri.004G027800<br>Potri.010G201400 Potri.017G133800 Potri.002G124500 Potri.011G152400 Potri.004G175100 Potri.002G100600<br>Potri.009G096600 Potri.005G167800 Potri.007G140800 Potri.006G143500 Potri.017G088200 Potri.013G003700<br>Potri.001G216300 Potri.010G141600 Potri.001G227300 Potri.010G145800 Potri.004G005300 Potri.003G076300<br>Potri.012G108500 Potri.001G413700 Potri.005G187600 Potri.002G054900 Potri.009G016100 Potri.005G111700<br>Potri.015G104800 Potri.014G195200 Potri.004G215400 Potri.006G193700 Potri.016G057400 Potri.019G004700<br>Potri.005G051600 Potri.010G159900 Potri.002G128300 Potri.005G237700 Potri.014G104800 Potri.003G100200<br>Potri.016G069400 Potri.006G071200 Potri.005G095400 Potri.001G145700 Potri.010G074300 Potri.007G044800<br>Potri.001G046100 Potri.003G156500 Potri.003G080600 Potri.019G125000 Potri.006G072400 Potri.014G012800<br>Potri.004G099400 Potri.008G058000 Potri.006G236200 Potri.004G051700 Potri.008G081000 Potri.001G440300<br>Potri.014G126100 Potri.012G042600 Potri.002G189200 Potri.007G147300 Potri.005G057500 Potri.018G057000<br>Potri.009G027000 Potri.019G018500 Potri.009G139800 Potri.017G031600 Potri.006G105300 Potri.014G122200<br>Potri.011G067400 Potri.010G097700 Potri.011G049600 Potri.006G037300 Potri.001G300900 Potri.006G099100<br>Potri.011G058300 Potri.008G160200 Potri.010G123500 Potri.014G180300 Potri.006G071600 Potri.013G125000<br>Potri.001G046400 Potri.011G028800 Potri.002G065600 Potri.003G117500 Potri.001G044500 Potri.018G029400<br>Potri.011G015600 Potri.005G077200 Potri.017G016700 Potri.006G066100 Potri.014G068700 Potri.002G142800<br>Potri.001G092900 Potri.001G099000 Potri.015G043400 Potri.003G182200 Potri.004G209300 Potri.006G002100<br>Potri.006G025800 Potri.004G117800 Potri.009G140300 Potri.007G023600 Potri.004G051800 Potri.001G343100<br>Potri.002G036200 Potri.006G235100 Potri.013G082200 Potri.012G054700 Potri.004G026400 Potri.010G236800<br>Potri.018G138700 Potri.002G240800 Potri.008G077900 Potri.010G212600 Potri.011G068500 Potri.009G119700<br>Potri.002G014300 Potri.001G299000 Potri.007G018100 Potri.001G168200 Potri.014G158400 Potri.002G201600<br>Potri.001G263200 Potri.011G039100 Potri.003G167700 Potri.004G189900 Potri.001G028200 Potri.016G072300<br>Potri.004G059600 Potri.003G187000 Potri.015G093100 Potri.005G116200 Potri.012G045900 Potri.018G045100<br>Potri.001G095200 Potri.014G106300 Potri.001G320000 Potri.011G125200 Potri.019G093700 Potri.010G103100<br>Potri.T095600 Potri.013G116400 Potri.001G467300 Potri.006G052600 Potri.001G163600 Potri.008G148200<br>Potri.007G067900 Potri.012G047300 Potri.005G063000 Potri.003G196000 Potri.001G121400 Potri.002G099200<br>Potri.013G060400 Potri.002G251700 Potri.T052100 Potri.008G094200 Potri.001G352400 Potri.009G116700<br>Potri.016G069500 Potri.016G117500 Potri.005G189500 Potri.011G169600 Potri.014G018900 Potri.012G124200<br>Potri.004G205000 Potri.012G021700 Potri.016G054900 Potri.007G007400 Potri.009G080600 Potri.007G135100<br>Potri.004G055700 Potri.014G017200 Potri.004G014700 Potri.004G015500 Potri.014G036600 Potri.004G065400<br>Potri.001G449100 Potri.002G236800 Potri.005G201200 Potri.004G021000 Potri.011G156100 Potri.006G202700<br>Potri.006G068900 Potri.007G048800 Potri.011G037100 Potri.014G101100 Potri.010G087900 Potri.013G153000<br>Potri.001G044400 Potri.014G160100 Potri.009G057900 Potri.005G007200 Potri.013G064300 Potri.006G208100<br>Potri.002G198000 Potri.004G139900 Potri.012G031700 Potri.001G061100 Potri.001G270000 Potri.007G099800

			Potri.006G188300 Potri.001G289500 Potri.001G463800 Potri.009G020400 Potri.010G004200 Potri.018G015100<br>Potri.014G157600 Potri.001G252100 Potri.002G009300 Potri.016G050500 Potri.007G110600 Potri.006G202600<br>Potri.T023800 Potri.019G032600 Potri.006G188600 Potri.008G068400 Potri.010G220100 Potri.016G034300<br>Potri.006G219600 Potri.002G236200 Potri.016G070500 Potri.005G223100 Potri.018G103900 Potri.018G138800<br>Potri.011G089700 Potri.018G032200 Potri.002G165800 Potri.011G057000 Potri.002G023900 Potri.005G257800<br>Potri.002G030900 Potri.001G158600 Potri.001G246400 Potri.019G028700 Potri.008G102500 Potri.002G034400<br>Potri.002G045000 Potri.010G188600 Potri.006G051700 Potri.004G154000 Potri.001G171900 Potri.004G226900<br>Potri.002G070500 Potri.011G010500 Potri.017G134100 Potri.014G111800 Potri.001G372300 Potri.014G146100<br>Potri.001G237200 Potri.011G120100 Potri.004G027400 Potri.001G216100 Potri.016G043400 Potri.016G028700<br>Potri.001G448400 Potri.005G218200 Potri.001G385600 Potri.018G045500 Potri.006G148800 Potri.006G228200<br>Potri.006G053700 Potri.006G062600 Potri.011G076700 Potri.019G073300 Potri.003G183100 Potri.007G127000<br>Potri.009G118300 Potri.009G061700 Potri.004G047600 Potri.005G073300 Potri.001G280200 Potri.016G128300<br>Potri.T022600 Potri.007G035900 Potri.015G145900 Potri.001G139500 Potri.002G202400 Potri.005G181800<br>Potri.002G023400 Potri.019G048800 Potri.001G071000 Potri.013G036300 Potri.016G144100 Potri.002G092100<br>Potri.010G113900 Potri.009G009600 Potri.009G100400 Potri.017G133900 Potri.014G164700 Potri.011G037700<br>Potri.010G223300 Potri.018G043900 Potri.014G155000 Potri.011G069600 Potri.T157700 Potri.012G134000<br>Potri.009G040200 Potri.012G055700 Potri.010G221200 Potri.017G135000 Potri.018G024500 Potri.008G038900<br>Potri.007G010000 Potri.005G141400 Potri.005G204600 Potri.008G140500 Potri.005G087200 Potri.001G226000<br>Potri.018G014600 Potri.008G203200 Potri.005G195000 Potri.004G083900 Potri.013G086100 Potri.006G146300<br>Potri.019G078400 Potri.012G032900 Potri.001G217700 Potri.010G137300 Potri.015G126900 Potri.T096300<br>Potri.016G126300 Potri.002G105300 Potri.004G054600 Potri.018G134100 Potri.006G181900 Potri.017G084000<br>Potri.003G159700 Potri.009G002200
GO:0019538	protein metabolic process	157	Potri.010G103100 Potri.001G246400 Potri.013G116400 Potri.019G064800 Potri.001G467300 Potri.019G008900<br>Potri.004G065400 Potri.001G158600 Potri.001G161600 Potri.019G028700 Potri.009G081800 Potri.011G139800<br>Potri.006G202600 Potri.001G412000 Potri.019G078400 Potri.007G048800 Potri.010G188600 Potri.007G067900<br>Potri.001G218100 Potri.006G051700 Potri.004G154000 Potri.008G058000 Potri.T003400 Potri.005G063000<br>Potri.016G134000 Potri.011G010500 Potri.001G440300 Potri.011G163700 Potri.003G183100 Potri.012G042600<br>Potri.004G027400 Potri.005G057500 Potri.001G042400 Potri.011G128700 Potri.001G216100 Potri.014G136300<br>Potri.016G069500 Potri.014G074600 Potri.001G280200 Potri.001G385600 Potri.018G045500 Potri.005G189500<br>Potri.011G169600 Potri.006G053700 Potri.010G097700 Potri.014G018900 Potri.006G079600 Potri.011G049600<br>Potri.011G037300 Potri.004G205000 Potri.006G099100 Potri.011G058300 Potri.009G002200 Potri.018G083600<br>Potri.001G163600 Potri.008G137700 Potri.003G107600 Potri.011G066800 Potri.007G127000 Potri.004G014700<br>Potri.011G028800 Potri.004G015500 Potri.003G117500 Potri.009G027000 Potri.007G044800 Potri.007G047900<br>Potri.T022600 Potri.007G035900 Potri.006G066100 Potri.010G221200 Potri.005G181800 Potri.014G068700

			Potri.019G048800 Potri.012G047400 Potri.013G036300 Potri.006G202700 Potri.004G135500 Potri.016G144100<br>Potri.004G209300 Potri.002G092100 Potri.010G113900 Potri.006G068900 Potri.011G106400 Potri.009G009600<br>Potri.009G100400 Potri.004G095700 Potri.002G019300 Potri.011G037700 Potri.019G002100 Potri.014G101100<br>Potri.014G155000 Potri.010G087900 Potri.009G010400 Potri.002G036200 Potri.012G033200 Potri.012G054700<br>Potri.004G026400 Potri.017G135000 Potri.018G024500 Potri.004G027800 Potri.010G201400 Potri.018G138700<br>Potri.013G064300 Potri.002G124500 Potri.002G198000 Potri.011G068500 Potri.005G204600 Potri.008G140500<br>Potri.002G251700 Potri.003G167700 Potri.018G015100 Potri.007G140800 Potri.012G124200 Potri.017G088200<br>Potri.007G110600 Potri.013G003700 Potri.001G216300 Potri.011G039100 Potri.009G020400 Potri.004G175100<br>Potri.001G028200 Potri.018G014600 Potri.008G203200 Potri.004G005300 Potri.004G226900 Potri.003G076300<br>Potri.004G083900 Potri.016G070500 Potri.004G215400 Potri.015G093100 Potri.002G009300 Potri.006G146300<br>Potri.009G016100 Potri.009G140300 Potri.001G413700 Potri.011G037100 Potri.001G217700 Potri.T023800<br>Potri.002G054900 Potri.011G067400 Potri.002G070500 Potri.016G126300 Potri.001G095200 Potri.014G195200<br>Potri.010G220100 Potri.018G134100 Potri.016G034300 Potri.012G021700 Potri.019G004700 Potri.005G051600<br>Potri.017G084000 Potri.013G086100 Potri.008G160200 Potri.018G138800 Potri.011G076700 Potri.011G125200<br>Potri.016G069400
GO:0006464	protein modification process	122	Potri.010G103100 Potri.001G246400 Potri.001G467300 Potri.019G008900 Potri.004G065400 Potri.001G161600<br>Potri.009G081800 Potri.011G139800 Potri.006G202600 Potri.001G412000 Potri.019G078400 Potri.007G048800<br>Potri.010G188600 Potri.007G067900 Potri.001G218100 Potri.006G051700 Potri.004G154000 Potri.009G020400<br>Potri.T003400 Potri.002G070500 Potri.016G134000 Potri.011G010500 Potri.011G163700 Potri.012G042600<br>Potri.004G027400 Potri.005G057500 Potri.001G042400 Potri.011G128700 Potri.001G216100 Potri.014G136300<br>Potri.016G069500 Potri.001G280200 Potri.001G385600 Potri.018G045500 Potri.005G189500 Potri.011G169600<br>Potri.006G053700 Potri.010G097700 Potri.006G079600 Potri.011G049600 Potri.011G037300 Potri.004G205000<br>Potri.006G099100 Potri.011G058300 Potri.008G160200 Potri.018G083600 Potri.008G137700 Potri.003G107600<br>Potri.003G183100 Potri.007G127000 Potri.004G014700 Potri.011G028800 Potri.004G015500 Potri.003G117500<br>Potri.007G044800 Potri.007G047900 Potri.T022600 Potri.006G066100 Potri.010G221200 Potri.005G181800<br>Potri.014G068700 Potri.019G048800 Potri.012G047400 Potri.013G036300 Potri.006G202700 Potri.004G135500<br>Potri.016G144100 Potri.004G209300 Potri.010G113900 Potri.011G106400 Potri.009G009600 Potri.009G100400<br>Potri.004G095700 Potri.002G019300 Potri.011G037700 Potri.014G101100 Potri.014G155000 Potri.010G087900<br>Potri.009G010400 Potri.002G036200 Potri.012G033200 Potri.012G054700 Potri.004G026400 Potri.017G135000<br>Potri.018G024500 Potri.004G027800 Potri.018G138700 Potri.013G064300 Potri.011G068500 Potri.008G140500<br>Potri.002G251700 Potri.007G140800 Potri.012G124200 Potri.017G088200 Potri.013G003700 Potri.001G216300<br>Potri.011G039100 Potri.004G175100 Potri.004G005300 Potri.004G226900 Potri.004G083900 Potri.016G070500<br>Potri.015G093100 Potri.002G009300 Potri.009G016100 Potri.009G140300 Potri.001G413700 Potri.011G037100<br>Potri.001G217700 Potri.T023800 Potri.011G067400 Potri.016G126300 Potri.001G095200 Potri.014G195200

			Potri.018G134100 Potri.007G110600 Potri.019G004700 Potri.005G051600 Potri.013G086100 Potri.018G138800 Potri.011G125200 Potri.016G069400
GO:0016567	protein ubiquitination	20	Potri.010G103100 Potri.004G205000 Potri.006G202600 Potri.004G083900 Potri.005G057500 Potri.010G113900 Potri.001G216100 Potri.009G016100 Potri.002G070500 Potri.016G134000 Potri.018G083600 Potri.008G137700 Potri.016G069500 Potri.012G042600 Potri.006G202700 Potri.014G101100 Potri.017G135000 Potri.016G069400 Potri.001G216300 Potri.007G110600
GO:0080090	regulation of primary metabolic process	71	Potri.011G057000 Potri.017G031600 Potri.002G201600 Potri.003G080600 Potri.005G223100 Potri.006G105300 Potri.006G193700 Potri.005G095400 Potri.010G004200 Potri.014G164700 Potri.002G030900 Potri.010G074300 Potri.005G087200 Potri.019G073300 Potri.005G195000 Potri.010G223300 Potri.001G452200 Potri.018G043900 Potri.007G023600 Potri.018G045100 Potri.004G051800 Potri.004G051700 Potri.001G372300 Potri.016G050500 Potri.004G055700 Potri.014G104800 Potri.001G099000 Potri.002G045000 Potri.012G134000 Potri.006G025800 Potri.008G148200 Potri.013G059600 Potri.012G032900 Potri.012G055700 Potri.014G036600 Potri.006G072400 Potri.014G012800 Potri.006G071600 Potri.007G135100 Potri.004G099400 Potri.005G111700 Potri.001G352400 Potri.006G236200 Potri.008G038900 Potri.002G065600 Potri.004G047600 Potri.001G044500 Potri.007G007400 Potri.002G105300 Potri.014G126100 Potri.016G128300 Potri.017G016700 Potri.005G141400 Potri.011G153300 Potri.006G208100 Potri.009G119700 Potri.002G100600 Potri.002G023400 Potri.012G031700 Potri.002G142800 Potri.001G092900 Potri.005G218200 Potri.014G106300 Potri.018G057000 Potri.001G270000 Potri.016G072300 Potri.001G448400 Potri.003G182200 Potri.012G108500 Potri.006G148800 Potri.009G139800
GO:0006979	response to oxidative stress	15	Potri.016G125000 Potri.016G132700 Potri.014G143200 Potri.002G065300 Potri.T160100 Potri.007G053400 Potri.006G129900 Potri.004G015300 Potri.001G458700 Potri.012G006800 Potri.015G003500 Potri.005G108900 Potri.001G351000 Potri.007G122100 Potri.016G132800
GO:0023052	signaling	34	Potri.006G053700 Potri.005G215700 Potri.018G046800 Potri.010G074300 Potri.014G164700 Potri.011G037300 Potri.001G252100 Potri.008G160200 Potri.014G103600 Potri.001G412000 Potri.001G413700 Potri.011G037100 Potri.015G105500 Potri.T023900 Potri.004G201600 Potri.005G111700 Potri.004G027800 Potri.010G107100 Potri.005G062700 Potri.T022600 Potri.008G100000 Potri.019G002500 Potri.004G027400 Potri.011G061500 Potri.012G053200 Potri.011G128700 Potri.011G039100 Potri.019G048800 Potri.013G086100 Potri.017G103300 Potri.011G125200 Potri.005G198800 Potri.016G097800 Potri.010G080900
GO:0055085	transmembrane transport	45	Potri.010G211400 Potri.015G008100 Potri.012G020500 Potri.006G112200 Potri.001G139700 Potri.016G004800 Potri.003G145600 Potri.001G139600 Potri.010G089800 Potri.016G111000 Potri.006G110500 Potri.016G035300 Potri.013G083300 Potri.002G092500 Potri.010G055200 Potri.004G215600 Potri.006G096800 Potri.005G102800 Potri.016G024400 Potri.013G131100 Potri.005G246600 Potri.019G086100 Potri.001G185700 Potri.012G043000 Potri.009G138900 Potri.016G053600 Potri.005G167300 Potri.005G169300 Potri.014G136500 Potri.014G077500 Potri.014G113200 Potri.010G174300 Potri.003G197100 Potri.001G027100 Potri.001G248600 Potri.001G007300 Potri.001G251200 Potri.012G070300 Potri.002G102100 Potri.003G121400 Potri.006G158900 Potri.012G144000

			Potri.001G331800 Potri.013G065800 Potri.004G019900
Function go term	Description	Matched counts	Matched genes
GO:0022804	active transmembrane transporter activity	16	Potri.003G197100 Potri.010G055200 Potri.005G215600 Potri.005G102800 Potri.001G007300 Potri.008G100300 Potri.006G188600 Potri.001G139700 Potri.002G102100 Potri.016G053600 Potri.003G121400 Potri.001G331800 Potri.001G139600 Potri.014G116000 Potri.019G086100 Potri.014G113200
GO:0030554	adenyl nucleotide binding	160	Potri.008G098800 Potri.013G116400 Potri.009G122100 Potri.001G467300 Potri.007G014800 Potri.019G008900 Potri.008G099300 Potri.004G065400 Potri.010G074300 Potri.001G161600 Potri.009G081800 Potri.011G139800 Potri.T130200 Potri.010G055200 Potri.001G412000 Potri.019G078400 Potri.007G048800 Potri.010G188600 Potri.007G067900 Potri.001G218100 Potri.006G051700 Potri.003G159800 Potri.014G012800 Potri.004G154000 Potri.009G020400 Potri.T003400 Potri.T149700 Potri.010G062500 Potri.011G010500 Potri.001G455300 Potri.011G163700 Potri.002G189200 Potri.014G113200 Potri.004G027400 Potri.001G042400 Potri.011G128700 Potri.011G156000 Potri.014G136300 Potri.003G044900 Potri.001G280200 Potri.001G385600 Potri.018G045500 Potri.003G053800 Potri.001G440700 Potri.011G067400 Potri.006G053700 Potri.010G097700 Potri.002G235500 Potri.006G079600 Potri.011G049600 Potri.005G181800 Potri.011G037300 Potri.016G101100 Potri.012G069800 Potri.006G099100 Potri.011G058300 Potri.008G160200 Potri.003G107600 Potri.003G183100 Potri.007G127000 Potri.001G459500 Potri.004G014700 Potri.011G028800 Potri.004G015500 Potri.014G125700 Potri.003G117500 Potri.007G044800 Potri.T022600 Potri.009G025300 Potri.006G137300 Potri.001G348200 Potri.006G066100 Potri.019G002500 Potri.011G111200 Potri.001G246400 Potri.014G068700 Potri.011G158700 Potri.019G048800 Potri.013G003700 Potri.002G201000 Potri.011G037700 Potri.001G111500 Potri.013G036300 Potri.004G135500 Potri.016G144100 Potri.004G209300 Potri.010G227000 Potri.014G164700 Potri.011G106400 Potri.009G009600 Potri.005G021100 Potri.009G100400 Potri.004G095700 Potri.012G020800 Potri.002G019300 Potri.001G139600 Potri.014G155000 Potri.010G087900 Potri.004G226900 Potri.009G010400 Potri.002G036200 Potri.T096100 Potri.012G033200 Potri.012G054700 Potri.004G026400 Potri.001G233700 Potri.010G221200 Potri.017G140100 Potri.011G146700 Potri.004G027800 Potri.011G169600 Potri.002G254200 Potri.018G138700 Potri.013G064300 Potri.007G020900 Potri.001G020900 Potri.001G360200 Potri.011G068500 Potri.008G140500 Potri.002G251700 Potri.T052100 Potri.013G057900 Potri.007G140800 Potri.012G124200 Potri.017G088200 Potri.012G053200 Potri.005G051600 Potri.001G139700 Potri.004G175100 Potri.004G005300 Potri.004G230300 Potri.016G070500 Potri.015G093100 Potri.002G009300 Potri.009G160600 Potri.009G140300 Potri.001G413700 Potri.011G037100 Potri.001G217700 Potri.T023800 Potri.001G000800 Potri.001G416300 Potri.006G070500 Potri.005G189500 Potri.005G111700 Potri.016G126300 Potri.001G095200 Potri.014G195200 Potri.001G134400 Potri.001G470100 Potri.018G134100 Potri.003G197100 Potri.016G056600 Potri.019G004700 Potri.011G039100 Potri.011G165200 Potri.017G084000 Potri.013G086100 Potri.018G138800 Potri.011G125200
GO:0016209	antioxidant activity	21	Potri.016G125000 Potri.001G351000 Potri.015G110400 Potri.016G132700 Potri.014G143200 Potri.002G065300

			Potri.006G137300 Potri.004G015300 Potri.007G053400 Potri.T160100 Potri.006G129900 Potri.018G063300 Potri.001G458700 Potri.016G132800 Potri.003G159800 Potri.015G003500 Potri.005G108900 Potri.012G006800 Potri.007G122100 Potri.019G057300 Potri.013G007800
GO:0070001	aspartic-type peptidase activity	15	Potri.008G058000 Potri.005G063000 Potri.005G204600 Potri.002G092100 Potri.006G068900 Potri.019G064800 Potri.001G158600 Potri.010G201400 Potri.018G015100 Potri.001G028200 Potri.018G014600 Potri.008G203200 Potri.002G054900 Potri.019G002100 Potri.003G076300
GO:0005524	ATP binding	149	Potri.008G098800 Potri.013G116400 Potri.009G122100 Potri.001G467300 Potri.007G014800 Potri.019G008900 Potri.008G099300 Potri.004G065400 Potri.010G074300 Potri.001G161600 Potri.009G081800 Potri.011G139800 Potri.T130200 Potri.010G055200 Potri.001G412000 Potri.019G078400 Potri.007G048800 Potri.010G188600 Potri.007G067900 Potri.001G218100 Potri.006G051700 Potri.014G012800 Potri.004G154000 Potri.009G020400 Potri.T003400 Potri.T149700 Potri.010G062500 Potri.011G010500 Potri.001G455300 Potri.011G163700 Potri.002G189200 Potri.014G113200 Potri.004G027400 Potri.001G042400 Potri.011G128700 Potri.014G136300 Potri.003G044900 Potri.001G280200 Potri.001G385600 Potri.018G045500 Potri.003G053800 Potri.011G067400 Potri.006G053700 Potri.010G097700 Potri.002G235500 Potri.006G079600 Potri.011G049600 Potri.005G181800 Potri.011G037300 Potri.016G101100 Potri.012G069800 Potri.006G099100 Potri.011G058300 Potri.008G160200 Potri.003G107600 Potri.003G183100 Potri.007G127000 Potri.004G014700 Potri.011G028800 Potri.004G015500 Potri.014G125700 Potri.003G117500 Potri.007G044800 Potri.T022600 Potri.009G025300 Potri.001G348200 Potri.006G066100 Potri.019G002500 Potri.011G111200 Potri.001G246400 Potri.014G068700 Potri.019G048800 Potri.013G003700 Potri.002G201000 Potri.011G037700 Potri.013G036300 Potri.004G135500 Potri.016G144100 Potri.004G209300 Potri.010G227000 Potri.014G164700 Potri.011G106400 Potri.009G009600 Potri.005G021100 Potri.009G100400 Potri.004G095700 Potri.012G020800 Potri.002G019300 Potri.001G139600 Potri.014G155000 Potri.010G087900 Potri.004G226900 Potri.009G010400 Potri.002G036200 Potri.T096100 Potri.012G033200 Potri.012G054700 Potri.004G026400 Potri.001G233700 Potri.010G221200 Potri.017G140100 Potri.011G146700 Potri.004G027800 Potri.011G169600 Potri.018G138700 Potri.013G064300 Potri.007G020900 Potri.001G360200 Potri.011G068500 Potri.008G140500 Potri.002G251700 Potri.013G057900 Potri.007G140800 Potri.012G124200 Potri.017G088200 Potri.012G053200 Potri.011G039100 Potri.001G139700 Potri.004G175100 Potri.004G005300 Potri.004G230300 Potri.016G070500 Potri.015G093100 Potri.002G009300 Potri.009G160600 Potri.009G140300 Potri.001G413700 Potri.011G037100 Potri.001G217700 Potri.T023800 Potri.001G000800 Potri.001G416300 Potri.006G070500 Potri.005G189500 Potri.005G111700 Potri.016G126300 Potri.001G095200 Potri.014G195200 Potri.001G134400 Potri.018G134100 Potri.003G197100 Potri.016G056600 Potri.019G004700 Potri.005G051600 Potri.011G165200 Potri.017G084000 Potri.013G086100 Potri.018G138800 Potri.011G125200
GO:0005509	calcium ion binding	6	Potri.001G024800 Potri.001G024900 Potri.010G107100 Potri.005G215700 Potri.007G092500 Potri.010G080900
GO:0004091	carboxylesterase activity	18	Potri.015G145900 Potri.T107600 Potri.011G135000 Potri.001G263200 Potri.014G149700 Potri.015G013700 Potri.015G128700 Potri.004G054600 Potri.009G057900 Potri.007G108300 Potri.016G001600 Potri.001G173700

			Potri.002G202600 Potri.002G145500 Potri.015G128200 Potri.010G109300 Potri.002G202500 Potri.001G119300
GO:0050662	coenzyme binding	39	Potri.016G120100 Potri.001G440700 Potri.T143400 Potri.002G033600 Potri.004G189900 Potri.013G074700 Potri.009G096600 Potri.001G459500 Potri.001G046100 Potri.009G118100 Potri.002G156000 Potri.005G116200 Potri.001G237200 Potri.012G098300 Potri.003G159800 Potri.002G254200 Potri.009G080600 Potri.001G046400 Potri.002G034400 Potri.004G118600 Potri.004G185000 Potri.015G104800 Potri.017G059100 Potri.004G054100 Potri.018G046600 Potri.006G137300 Potri.009G145000 Potri.001G470100 Potri.001G020900 Potri.011G156100 Potri.T052100 Potri.011G158700 Potri.011G156000 Potri.016G043400 Potri.001G111500 Potri.009G118300 Potri.001G320000 Potri.008G094300 Potri.003G100200
GO:0030234	enzyme regulator activity	24	Potri.011G135000 Potri.019G080600 Potri.001G309900 Potri.015G128200 Potri.002G202500 Potri.002G145500 Potri.T107600 Potri.011G110100 Potri.015G128700 Potri.001G119300 Potri.009G028300 Potri.015G105500 Potri.019G124500 Potri.016G001600 Potri.007G108300 Potri.008G062000 Potri.019G124700 Potri.014G149700 Potri.007G111500 Potri.010G109300 Potri.005G137500 Potri.002G202600 Potri.015G013700 Potri.003G097900
GO:0050660	FAD binding	11	Potri.T052100 Potri.001G440700 Potri.011G158700 Potri.011G156000 Potri.006G137300 Potri.001G111500 Potri.003G159800 Potri.002G254200 Potri.001G020900 Potri.001G470100 Potri.001G459500
GO:0046527	glucosyltransferase activity	12	Potri.002G066600 Potri.011G069600 Potri.018G029400 Potri.006G181900 Potri.018G103900 Potri.016G054900 Potri.013G082200 Potri.004G117800 Potri.002G257900 Potri.006G052600 Potri.014G125100 Potri.004G059600
GO:0020037	heme binding	41	Potri.001G365400 Potri.009G145400 Potri.016G125000 Potri.007G115500 Potri.002G165800 Potri.018G146100 Potri.002G065300 Potri.T160100 Potri.001G113900 Potri.006G141400 Potri.001G331100 Potri.007G122100 Potri.001G362600 Potri.019G064200 Potri.001G003100 Potri.006G228200 Potri.013G106200 Potri.006G129900 Potri.012G006800 Potri.001G458700 Potri.004G235400 Potri.002G242500 Potri.001G351000 Potri.004G140900 Potri.001G364900 Potri.004G106600 Potri.007G053400 Potri.007G033300 Potri.005G108900 Potri.016G132800 Potri.005G143900 Potri.019G064600 Potri.016G132700 Potri.014G143200 Potri.018G051300 Potri.018G134300 Potri.009G110800 Potri.004G015300 Potri.015G003500 Potri.004G017700 Potri.011G155600
GO:0016787	hydrolase activity	163	Potri.011G135000 Potri.019G064800 Potri.005G049300 Potri.007G014800 Potri.001G158600 Potri.001G173700 Potri.014G158400 Potri.008G098800 Potri.010G055200 Potri.003G156500 Potri.006G110900 Potri.019G125000 Potri.014G012800 Potri.005G204800 Potri.008G058000 Potri.019G024700 Potri.001G310500 Potri.T149700 Potri.006G188100 Potri.001G440300 Potri.001G191400 Potri.014G146100 Potri.015G031300 Potri.002G023900 Potri.014G113200 Potri.014G149700 Potri.008G094200 Potri.012G116000 Potri.016G028700 Potri.014G074600 Potri.013G127500 Potri.008G196800 Potri.009G122100 Potri.014G022900 Potri.011G094400 Potri.001G191000 Potri.002G235500 Potri.011G076700 Potri.005G021100 Potri.015G031400 Potri.015G128200 Potri.012G069800 Potri.015G128700 Potri.009G002200 Potri.010G123500 Potri.001G163600 Potri.005G007200 Potri.011G066800 Potri.013G125000 Potri.014G125700 Potri.018G095100 Potri.006G062200 Potri.007G108300 Potri.002G257700 Potri.007G099800 Potri.011G022300 Potri.005G201200 Potri.007G047900 Potri.018G112000 Potri.001G348200 Potri.015G145900 Potri.T107600 Potri.002G202400 Potri.001G071000 Potri.001G000800 Potri.015G041300

			Potri.009G025300 Potri.006G002100 Potri.002G092100 Potri.006G068900 Potri.010G227000 Potri.008G081000 Potri.004G021000 Potri.003G053800 Potri.014G122200 Potri.001G139600 Potri.019G002100 Potri.006G198800 Potri.002G202500 Potri.010G220100 Potri.002G145500 Potri.001G227300 Potri.005G063000 Potri.004G054600 Potri.001G119300 Potri.013G153000 Potri.T157700 Potri.009G040200 Potri.001G104600 Potri.011G146700 Potri.010G236800 Potri.001G233700 Potri.001G044400 Potri.014G160100 Potri.007G010000 Potri.009G057900 Potri.010G201400 Potri.016G034300 Potri.006G005400 Potri.002G124500 Potri.001G360200 Potri.005G204600 Potri.011G152400 Potri.007G035900 Potri.015G013700 Potri.001G061100 Potri.014G018900 Potri.005G167800 Potri.002G236200 Potri.001G299000 Potri.006G188300 Potri.007G018100 Potri.001G289500 Potri.001G226000 Potri.001G168200 Potri.010G141600 Potri.018G121500 Potri.005G215600 Potri.001G263200 Potri.010G193500 Potri.001G171900 Potri.010G109300 Potri.001G139700 Potri.018G015100 Potri.001G028200 Potri.018G014600 Potri.008G203200 Potri.003G076300 Potri.002G201000 Potri.001G449100 Potri.004G215400 Potri.001G252100 Potri.009G160600 Potri.003G034600 Potri.001G455300 Potri.010G137300 Potri.002G054900 Potri.001G416300 Potri.010G062500 Potri.015G048700 Potri.002G202600 Potri.016G001600 Potri.014G157600 Potri.006G188600 Potri.008G068400 Potri.011G089700 Potri.009G069900 Potri.014G111800 Potri.016G057400 Potri.008G086700 Potri.003G197100 Potri.016G056600 Potri.011G081600 Potri.011G165200 Potri.010G159900 Potri.017G084000 Potri.003G159700 Potri.005G237700 Potri.001G331800 Potri.009G103800 Potri.010G105900 Potri.019G093700 Potri.006G071200
GO:0016798	hydrolase activity, acting on glycosyl bonds	44	Potri.001G226000 Potri.010G141600 Potri.014G146100 Potri.001G227300 Potri.008G081000 Potri.002G023900 Potri.011G094400 Potri.014G122200 Potri.014G158400 Potri.001G071000 Potri.018G121500 Potri.003G159700 Potri.014G157600 Potri.006G002100 Potri.003G156500 Potri.019G125000 Potri.010G137300 Potri.013G125000 Potri.001G044400 Potri.001G171900 Potri.007G010000 Potri.018G095100 Potri.006G062200 Potri.001G449100 Potri.014G111800 Potri.007G099800 Potri.005G201200 Potri.004G021000 Potri.018G112000 Potri.005G007200 Potri.016G057400 Potri.002G202400 Potri.011G152400 Potri.002G236200 Potri.010G159900 Potri.005G167800 Potri.008G094200 Potri.005G237700 Potri.001G299000 Potri.006G188300 Potri.007G018100 Potri.015G041300 Potri.019G093700 Potri.006G071200
GO:0005506	iron ion binding	45	Potri.001G365400 Potri.009G145400 Potri.016G125000 Potri.001G463800 Potri.007G115500 Potri.002G165800 Potri.018G146100 Potri.002G065300 Potri.T160100 Potri.001G113900 Potri.006G141400 Potri.001G331100 Potri.007G122100 Potri.012G006800 Potri.001G362600 Potri.019G064200 Potri.001G003100 Potri.006G228200 Potri.013G106200 Potri.006G129900 Potri.014G180300 Potri.001G458700 Potri.004G235400 Potri.003G159800 Potri.002G242500 Potri.001G351000 Potri.004G140900 Potri.001G364900 Potri.004G106600 Potri.007G053400 Potri.007G033300 Potri.006G137300 Potri.005G108900 Potri.016G132800 Potri.005G143900 Potri.019G064600 Potri.016G132700 Potri.014G143200 Potri.018G051300 Potri.018G134300 Potri.009G110800 Potri.004G015300 Potri.015G003500 Potri.004G017700 Potri.011G155600
GO:0016298	lipase activity	7	Potri.001G252100 Potri.001G263200 Potri.004G054600 Potri.009G057900 Potri.008G068400 Potri.001G173700

			Potri.015G145900
GO:0003777	microtubule motor activity	15	Potri.001G360200 Potri.009G122100 Potri.014G125700 Potri.010G227000 Potri.011G165200 Potri.007G014800 Potri.002G201000 Potri.002G235500 Potri.005G021100 Potri.001G455300 Potri.003G053800 Potri.011G146700 Potri.001G000800 Potri.001G416300 Potri.001G233700
GO:0051287	NAD or NADH binding	7	Potri.004G118600 Potri.009G096600 Potri.018G046600 Potri.002G156000 Potri.016G043400 Potri.013G074700 Potri.008G094300
GO:0017111	nucleoside-triphosphatase activity	37	Potri.001G289500 Potri.009G122100 Potri.005G215600 Potri.010G227000 Potri.007G014800 Potri.001G139700 Potri.002G235500 Potri.005G021100 Potri.003G053800 Potri.001G139600 Potri.012G069800 Potri.010G055200 Potri.009G160600 Potri.008G098800 Potri.009G040200 Potri.001G104600 Potri.011G146700 Potri.001G416300 Potri.001G233700 Potri.014G125700 Potri.T149700 Potri.010G062500 Potri.011G022300 Potri.006G188600 Potri.001G455300 Potri.009G025300 Potri.001G348200 Potri.014G113200 Potri.001G360200 Potri.003G197100 Potri.016G056600 Potri.011G165200 Potri.017G084000 Potri.002G201000 Potri.001G000800 Potri.001G331800 Potri.010G105900
GO:0016491	oxidoreductase activity	144	Potri.001G365400 Potri.007G108400 Potri.016G125000 Potri.002G165800 Potri.T160100 Potri.002G033600 Potri.001G113900 Potri.006G141400 Potri.001G331100 Potri.007G017100 Potri.001G362600 Potri.010G096800 Potri.001G046100 Potri.002G156000 Potri.012G006800 Potri.008G073800 Potri.018G063300 Potri.001G007100 Potri.003G159800 Potri.017G059100 Potri.006G087100 Potri.001G113100 Potri.004G140900 Potri.008G064000 Potri.008G074500 Potri.002G191900 Potri.016G102300 Potri.001G237200 Potri.007G147300 Potri.001G307500 Potri.016G132700 Potri.011G156000 Potri.009G107600 Potri.016G043400 Potri.004G060700 Potri.007G108600 Potri.001G046400 Potri.016G117500 Potri.001G459500 Potri.004G017700 Potri.013G007800 Potri.003G112700 Potri.007G115500 Potri.006G228200 Potri.002G065300 Potri.008G069300 Potri.009G096600 Potri.010G193100 Potri.019G121700 Potri.001G300900 Potri.014G180300 Potri.010G023600 Potri.006G096900 Potri.009G080600 Potri.001G378400 Potri.009G118300 Potri.004G106600 Potri.018G046600 Potri.007G053400 Potri.006G137300 Potri.T107300 Potri.006G206500 Potri.006G151600 Potri.016G132800 Potri.013G064200 Potri.011G156100 Potri.019G064600 Potri.004G146000 Potri.011G158700 Potri.008G194100 Potri.001G111500 Potri.009G010000 Potri.002G034400 Potri.015G003500 Potri.005G247700 Potri.T069600 Potri.001G252900 Potri.010G055400 Potri.001G229500 Potri.T107400 Potri.005G138400 Potri.001G268600 Potri.007G040900 Potri.019G064200 Potri.008G161600 Potri.001G003100 Potri.015G110400 Potri.013G106200 Potri.001G458700 Potri.005G136200 Potri.005G043400 Potri.016G117100 Potri.002G236700 Potri.002G254200 Potri.002G040700 Potri.001G364900 Potri.001G440700 Potri.018G033400 Potri.007G033300 Potri.008G073700 Potri.008G158300 Potri.006G062600 Potri.002G013700 Potri.001G020900 Potri.017G075100 Potri.T052100 Potri.011G155600 Potri.018G051300 Potri.018G134300 Potri.001G219300 Potri.007G122100 Potri.009G145400 Potri.001G463800 Potri.018G146100 Potri.008G198600 Potri.004G189900 Potri.013G074700 Potri.019G057300 Potri.012G087800 Potri.006G129900 Potri.001G327800 Potri.005G116200 Potri.005G187600 Potri.004G235400 Potri.011G152800 Potri.001G351000

			Potri.001G015400 Potri.006G101100 Potri.004G118600 Potri.016G112000 Potri.015G104800 Potri.006G004500 Potri.008G094300 Potri.005G108900 Potri.001G470100 Potri.004G199900 Potri.005G143900 Potri.008G077900 Potri.014G143200 Potri.011G120200 Potri.004G015300 Potri.001G320000 Potri.009G107700 Potri.003G100200
GO:0030599	pectinesterase activity	13	Potri.002G145500 Potri.T107600 Potri.011G135000 Potri.015G128700 Potri.014G149700 Potri.015G013700 Potri.016G001600 Potri.007G108300 Potri.010G109300 Potri.002G202600 Potri.015G128200 Potri.002G202500 Potri.001G119300
GO:0004601	peroxidase activity	17	Potri.016G125000 Potri.016G132700 Potri.014G143200 Potri.002G065300 Potri.006G137300 Potri.T160100 Potri.007G053400 Potri.006G129900 Potri.004G015300 Potri.001G458700 Potri.012G006800 Potri.003G159800 Potri.015G003500 Potri.005G108900 Potri.001G351000 Potri.007G122100 Potri.016G132800
GO:0042578	phosphoric ester hydrolase activity	11	Potri.001G168200 Potri.001G252100 Potri.001G061100 Potri.007G047900 Potri.003G034600 Potri.002G257700 Potri.001G191000 Potri.006G110900 Potri.008G068400 Potri.008G196800 Potri.010G193500
GO:0004672	protein kinase activity	102	Potri.001G467300 Potri.019G008900 Potri.004G065400 Potri.010G074300 Potri.001G246400 Potri.009G081800 Potri.011G139800 Potri.001G412000 Potri.019G078400 Potri.007G048800 Potri.010G188600 Potri.007G067900 Potri.001G218100 Potri.006G051700 Potri.004G154000 Potri.009G020400 Potri.T003400 Potri.011G010500 Potri.011G163700 Potri.004G027400 Potri.001G042400 Potri.011G128700 Potri.014G136300 Potri.001G280200 Potri.001G385600 Potri.018G045500 Potri.005G189500 Potri.011G067400 Potri.006G053700 Potri.001G161600 Potri.006G079600 Potri.011G049600 Potri.011G037300 Potri.006G099100 Potri.011G058300 Potri.008G160200 Potri.003G107600 Potri.003G183100 Potri.007G127000 Potri.004G014700 Potri.011G028800 Potri.004G015500 Potri.003G117500 Potri.007G044800 Potri.T022600 Potri.006G066100 Potri.010G221200 Potri.005G181800 Potri.014G068700 Potri.019G048800 Potri.013G003700 Potri.013G036300 Potri.004G135500 Potri.016G144100 Potri.004G209300 Potri.014G164700 Potri.011G106400 Potri.009G009600 Potri.009G100400 Potri.004G095700 Potri.002G019300 Potri.011G037700 Potri.014G155000 Potri.010G087900 Potri.009G010400 Potri.002G036200 Potri.012G033200 Potri.012G054700 Potri.004G026400 Potri.004G027800 Potri.011G169600 Potri.018G138700 Potri.013G064300 Potri.011G068500 Potri.008G140500 Potri.002G251700 Potri.010G097700 Potri.007G140800 Potri.012G124200 Potri.017G088200 Potri.019G004700 Potri.011G039100 Potri.004G175100 Potri.004G005300 Potri.004G226900 Potri.015G093100 Potri.002G009300 Potri.009G140300 Potri.001G413700 Potri.011G037100 Potri.001G217700 Potri.T023800 Potri.005G111700 Potri.016G126300 Potri.001G095200 Potri.014G195200 Potri.018G134100 Potri.016G070500 Potri.005G051600 Potri.013G086100 Potri.018G138800 Potri.011G125200
GO:0004674	protein serine/threonine kinase activity	9	Potri.011G037300 Potri.011G128700 Potri.001G412000 Potri.011G039100 Potri.011G037100 Potri.001G413700 Potri.002G036200 Potri.T022600 Potri.T023800
GO:0017171	serine hydrolase activity	11	Potri.004G215400 Potri.016G034300 Potri.007G035900 Potri.009G002200 Potri.001G163600 Potri.001G440300 Potri.014G018900 Potri.011G076700 Potri.014G074600 Potri.010G220100 Potri.002G124500
GO:0016229	steroid dehydrogenase activity	12	Potri.001G046100 Potri.011G156100 Potri.002G034400 Potri.015G104800 Potri.002G033600 Potri.004G189900 Potri.001G237200 Potri.001G320000 Potri.017G059100 Potri.003G100200 Potri.001G046400 Potri.009G118300

GO:0003700	transcription factor activity	44	Potri.011G057000 Potri.002G201600 Potri.003G080600 Potri.005G223100 Potri.006G105300 Potri.010G004200<br>Potri.002G030900 Potri.016G072300 Potri.005G087200 Potri.019G073300 Potri.005G195000 Potri.010G223300<br>Potri.018G043900 Potri.018G045100 Potri.004G051800 Potri.004G051700 Potri.001G372300 Potri.012G108500<br>Potri.001G352400 Potri.012G134000 Potri.006G025800 Potri.008G148200 Potri.013G059600 Potri.012G032900<br>Potri.007G007400 Potri.006G072400 Potri.006G071600 Potri.007G135100 Potri.008G038900 Potri.002G065600<br>Potri.004G047600 Potri.001G044500 Potri.014G126100 Potri.016G128300 Potri.003G182200 Potri.005G141400<br>Potri.009G119700 Potri.002G100600 Potri.002G023400 Potri.012G031700 Potri.002G142800 Potri.001G092900<br>Potri.001G099000 Potri.006G193700
GO:0016757	transferase activity, transferring glycosyl groups	54	Potri.002G132900 Potri.014G125000 Potri.004G117800 Potri.016G086400 Potri.006G052600 Potri.013G082200<br>Potri.002G151400 Potri.004G059600 Potri.016G016100 Potri.001G071000 Potri.011G069600 Potri.006G022300<br>Potri.006G131000 Potri.018G103900 Potri.016G054900 Potri.009G095300 Potri.014G125100 Potri.019G125000<br>Potri.010G129400 Potri.002G066600 Potri.012G068300 Potri.018G024500 Potri.015G045500 Potri.006G023000<br>Potri.016G021600 Potri.016G020400 Potri.017G052000 Potri.018G095100 Potri.018G029400 Potri.004G021000<br>Potri.003G076800 Potri.001G100700 Potri.003G131400 Potri.005G201200 Potri.014G073800 Potri.002G257900<br>Potri.014G146100 Potri.003G191200 Potri.014G029900 Potri.005G007200 Potri.006G023700 Potri.007G031700<br>Potri.006G181900 Potri.006G047200 Potri.009G077200 Potri.008G116900 Potri.001G416800 Potri.003G159700<br>Potri.002G236200 Potri.008G006500 Potri.014G040300 Potri.010G121800 Potri.016G019400 Potri.006G071200
GO:0016772	transferase activity, transferring phosphorus-containing groups	118	Potri.013G116400 Potri.001G467300 Potri.019G008900 Potri.005G257800 Potri.004G065400 Potri.010G074300<br>Potri.001G246400 Potri.008G102500 Potri.011G139800 Potri.001G412000 Potri.019G078400 Potri.007G048800<br>Potri.010G188600 Potri.007G067900 Potri.001G218100 Potri.006G051700 Potri.T148900 Potri.004G154000<br>Potri.009G020400 Potri.T003400 Potri.011G010500 Potri.003G196000 Potri.011G163700 Potri.013G060400<br>Potri.004G027400 Potri.001G042400 Potri.011G128700 Potri.014G136300 Potri.003G044900 Potri.001G280200<br>Potri.001G385600 Potri.018G045500 Potri.005G189500 Potri.011G067400 Potri.006G053700 Potri.001G161600<br>Potri.006G079600 Potri.011G049600 Potri.011G037300 Potri.006G099100 Potri.011G058300 Potri.008G160200<br>Potri.003G107600 Potri.003G183100 Potri.009G081800 Potri.007G127000 Potri.004G014700 Potri.011G028800<br>Potri.004G015500 Potri.003G117500 Potri.007G044800 Potri.011G015600 Potri.T022600 Potri.006G066100<br>Potri.010G221200 Potri.005G181800 Potri.014G068700 Potri.019G048800 Potri.013G003700 Potri.019G032600<br>Potri.013G036300 Potri.004G135500 Potri.016G144100 Potri.004G209300 Potri.014G164700 Potri.011G106400<br>Potri.009G009600 Potri.009G100400 Potri.004G095700 Potri.002G019300 Potri.011G037700 Potri.014G155000<br>Potri.006G235100 Potri.010G087900 Potri.004G226900 Potri.009G010400 Potri.002G036200 Potri.012G033200<br>Potri.012G054700 Potri.004G026400 Potri.008G060100 Potri.004G027800 Potri.011G169600 Potri.018G138700<br>Potri.013G064300 Potri.011G068500 Potri.008G140500 Potri.002G251700 Potri.010G097700 Potri.004G139900<br>Potri.013G057900 Potri.007G140800 Potri.012G124200 Potri.006G143500 Potri.017G088200 Potri.019G004700<br>Potri.011G039100 Potri.004G175100 Potri.004G005300 Potri.004G230300 Potri.015G093100 Potri.002G009300

			Potri.009G140300 Potri.001G413700 Potri.011G037100 Potri.001G217700 Potri.T023800 Potri.006G070500 Potri.005G111700 Potri.016G126300 Potri.001G095200 Potri.014G195200 Potri.018G134100 Potri.016G070500 Potri.005G051600 Potri.013G086100 Potri.018G138800 Potri.011G125200
GO:0005215	transporter activity	52	Potri.010G211400 Potri.015G008100 Potri.004G032300 Potri.005G215600 Potri.006G172100 Potri.001G139700 Potri.003G050900 Potri.001G123700 Potri.012G071400 Potri.010G089800 Potri.001G139600 Potri.014G116000 Potri.016G111000 Potri.013G083300 Potri.002G092500 Potri.016G055300 Potri.010G055200 Potri.006G096800 Potri.005G102800 Potri.016G024400 Potri.006G268200 Potri.013G131100 Potri.008G100300 Potri.019G086100 Potri.001G185700 Potri.016G089500 Potri.002G225500 Potri.012G043000 Potri.007G044000 Potri.003G148200 Potri.016G053600 Potri.005G167300 Potri.006G188600 Potri.005G169300 Potri.014G136500 Potri.014G113200 Potri.001G374600 Potri.010G174300 Potri.003G197100 Potri.001G027100 Potri.001G248600 Potri.010G222700 Potri.005G216000 Potri.012G070300 Potri.002G102100 Potri.006G034000 Potri.001G157000 Potri.003G121400 Potri.006G158900 Potri.012G144000 Potri.001G331800 Potri.001G007300
GO:0004842	ubiquitin-protein ligase activity	20	Potri.010G103100 Potri.004G205000 Potri.006G202600 Potri.004G083900 Potri.005G057500 Potri.010G113900 Potri.001G216100 Potri.009G016100 Potri.002G070500 Potri.016G134000 Potri.018G083600 Potri.008G137700 Potri.016G069500 Potri.012G042600 Potri.006G202700 Potri.014G101100 Potri.017G135000 Potri.016G069400 Potri.001G216300 Potri.007G110600

Supplementary Table S2 (cont.): Details of GO terms enriched by up-regulated DEGs of Aig at **36 hpi** of three infection stages in Figure 4

Process go term	Description	Matched counts	Matched genes
GO:0005975	carbohydrate metabolic process	22	Potri.006G002100 Potri.009G087600 Potri.015G058400 Potri.001G403900 Potri.001G227300 Potri.012G047300 Potri.006G037300 Potri.007G010000 Potri.015G020300 Potri.001G449100 Potri.016G057400 Potri.010G160200 Potri.002G093300 Potri.011G152400 Potri.004G010500 Potri.001G061100 Potri.001G100000 Potri.005G167800 Potri.012G047400 Potri.012G045900 Potri.001G320000 Potri.006G188300
GO:0007154	cell communication	5	Potri.004G027400 Potri.011G125200 Potri.011G128700 Potri.T021900 Potri.T023900
GO:0008037	cell recognition	5	Potri.004G027400 Potri.011G125200 Potri.011G128700 Potri.T021900 Potri.T023900
GO:0044262	cellular carbohydrate metabolic process	7	Potri.002G093300 Potri.015G020300 Potri.006G037300 Potri.015G058400 Potri.001G320000 Potri.001G061100 Potri.012G045900
GO:0044237	cellular metabolic process	83	Potri.004G186200 Potri.T096300 Potri.001G457000 Potri.001G061100 Potri.011G072300 Potri.015G058400 Potri.010G004200 Potri.003G167700 Potri.004G108200 Potri.006G079600 Potri.011G150700 Potri.002G124800 Potri.014G046300 Potri.003G162800 Potri.015G129500 Potri.008G102500 Potri.008G206200 Potri.003G111900 Potri.015G093100 Potri.002G009300 Potri.007G140800 Potri.008G204900 Potri.001G343100 Potri.007G110600 Potri.002G036200 Potri.004G095700 Potri.001G393200 Potri.007G067900 Potri.010G113900 Potri.001G218100 Potri.002G053000 Potri.019G045000 Potri.005G073300 Potri.006G037300 Potri.001G217700 Potri.012G054700 Potri.013G025900 Potri.013G099500 Potri.012G045900 Potri.T148900 Potri.008G157400 Potri.001G287200 Potri.004G015500 Potri.T023800 Potri.005G039800 Potri.006G141500 Potri.015G020300 Potri.004G205000 Potri.005G131600 Potri.015G074600 Potri.012G108500 Potri.012G123700 Potri.009G026800 Potri.T021900 Potri.008G064700 Potri.011G125200 Potri.011G068500 Potri.007G146000 Potri.006G202700 Potri.007G147300 Potri.004G027400 Potri.002G093300 Potri.016G050500 Potri.014G147300 Potri.005G057500 Potri.009G119700 Potri.005G254900 Potri.002G100600 Potri.016G070500 Potri.011G128700 Potri.005G051600 Potri.019G048800 Potri.014G106300 Potri.012G047400 Potri.018G057000 Potri.009G010000 Potri.012G047300 Potri.001G320000 Potri.001G280200 Potri.008G106700 Potri.005G093200 Potri.016G069400 Potri.009G139800
GO:0044267	cellular protein metabolic process	39	Potri.010G113900 Potri.011G072300 Potri.006G141500 Potri.003G167700 Potri.004G108200 Potri.006G079600 Potri.011G150700 Potri.003G162800 Potri.014G147300 Potri.004G205000 Potri.015G093100 Potri.002G009300 Potri.007G140800 Potri.007G110600 Potri.002G036200 Potri.004G095700 Potri.001G393200 Potri.007G067900 Potri.001G218100 Potri.001G217700 Potri.T023800 Potri.012G054700 Potri.005G254900 Potri.004G186200 Potri.004G015500 Potri.T021900 Potri.008G064700 Potri.004G027400 Potri.011G068500 Potri.005G057500 Potri.016G070500 Potri.011G128700 Potri.005G051600 Potri.019G048800 Potri.012G047400 Potri.001G280200 Potri.011G125200 Potri.006G202700 Potri.016G069400
GO:0006629	lipid metabolic process	13	Potri.016G116000 Potri.001G252100 Potri.T095600 Potri.008G204900 Potri.017G134100 Potri.002G128300

			Potri.012G047400 Potri.010G221700 Potri.009G026800 Potri.018G132100 Potri.001G173700 Potri.001G320000 Potri.011G089700
GO:0043170	macromolecule metabolic process	73	Potri.004G186200 Potri.014G026500 Potri.010G113900 Potri.011G072300 Potri.006G141500 Potri.010G004200 Potri.003G167700 Potri.004G108200 Potri.006G079600 Potri.011G150700 Potri.002G124800 Potri.001G041700 Potri.003G162800 Potri.015G129500 Potri.013G025900 Potri.008G206200 Potri.003G111900 Potri.015G093100 Potri.002G009300 Potri.007G140800 Potri.016G050500 Potri.001G343100 Potri.005G141600 Potri.002G036200 Potri.004G095700 Potri.001G393200 Potri.007G067900 Potri.001G287200 Potri.001G218100 Potri.012G108500 Potri.001G217700 Potri.T023800 Potri.012G054700 Potri.008G102500 Potri.002G104600 Potri.012G047300 Potri.T148900 Potri.008G157400 Potri.003G076300 Potri.004G015500 Potri.005G039800 Potri.004G205000 Potri.005G131600 Potri.015G074600 Potri.006G036500 Potri.011G146300 Potri.T021900 Potri.011G125200 Potri.019G064300 Potri.011G068500 Potri.006G204700 Potri.008G064700 Potri.004G027400 Potri.002G093300 Potri.014G147300 Potri.005G057500 Potri.009G119700 Potri.002G100600 Potri.016G070500 Potri.011G128700 Potri.005G051600 Potri.019G048800 Potri.014G106300 Potri.012G047400 Potri.018G057000 Potri.005G254900 Potri.001G320000 Potri.001G280200 Potri.008G106700 Potri.006G202700 Potri.007G110600 Potri.016G069400 Potri.009G139800
GO:0051704	multi-organism process	7	Potri.004G027400 Potri.011G128700 Potri.012G047400 Potri.T021900 Potri.011G125200 Potri.012G047300 Potri.T023900
GO:0055114	oxidation reduction	47	Potri.001G365400 Potri.006G137500 Potri.016G125000 Potri.001G457000 Potri.011G158700 Potri.T160100 Potri.006G094700 Potri.006G129900 Potri.019G064600 Potri.014G102700 Potri.005G108900 Potri.001G011500 Potri.019G121700 Potri.019G064200 Potri.008G161600 Potri.001G003100 Potri.008G204900 Potri.001G458700 Potri.018G063300 Potri.019G045000 Potri.001G113100 Potri.001G351000 Potri.013G099500 Potri.001G364900 Potri.008G157400 Potri.003G005800 Potri.002G071000 Potri.011G047300 Potri.002G191900 Potri.004G067000 Potri.006G062500 Potri.006G151600 Potri.001G015300 Potri.007G147300 Potri.015G074600 Potri.001G362600 Potri.016G132700 Potri.013G156500 Potri.006G198700 Potri.001G365100 Potri.009G025900 Potri.010G023600 Potri.009G010000 Potri.001G320000 Potri.015G003500 Potri.014G143200 Potri.001G391100
GO:0006793	phosphorus metabolic process	29	Potri.011G072300 Potri.006G141500 Potri.004G108200 Potri.006G079600 Potri.014G147300 Potri.015G093100 Potri.002G009300 Potri.001G218100 Potri.004G095700 Potri.001G393200 Potri.007G067900 Potri.002G036200 Potri.001G217700 Potri.019G045000 Potri.012G054700 Potri.004G186200 Potri.004G015500 Potri.T023800 Potri.T021900 Potri.004G027400 Potri.011G068500 Potri.016G070500 Potri.011G128700 Potri.005G051600 Potri.019G048800 Potri.007G140800 Potri.009G010000 Potri.001G280200 Potri.011G125200
GO:0016310	phosphorylation	29	Potri.011G072300 Potri.006G141500 Potri.004G108200 Potri.006G079600 Potri.014G147300 Potri.015G093100 Potri.002G009300 Potri.001G218100 Potri.004G095700 Potri.001G393200 Potri.007G067900 Potri.002G036200 Potri.001G217700 Potri.019G045000 Potri.012G054700 Potri.004G186200 Potri.004G015500 Potri.T023800 Potri.T021900 Potri.004G027400 Potri.011G068500 Potri.016G070500 Potri.011G128700 Potri.005G051600

			Potri.019G048800 Potri.007G140800 Potri.009G010000 Potri.001G280200 Potri.011G125200
GO:0044238	primary metabolic process	110	Potri.T095600 Potri.011G072300 Potri.015G058400 Potri.001G173700 Potri.003G162800 Potri.008G102500 Potri.008G206200 Potri.003G111900 Potri.004G095700 Potri.014G046300 Potri.007G067900 Potri.001G218100 Potri.012G047300 Potri.T148900 Potri.005G039800 Potri.015G020300 Potri.002G093300 Potri.017G134100 Potri.006G204700 Potri.007G147300 Potri.004G027400 Potri.005G057500 Potri.002G104600 Potri.011G128700 Potri.001G100000 Potri.018G057000 Potri.005G254900 Potri.001G280200 Potri.005G093200 Potri.009G139800 Potri.001G457000 Potri.006G141500 Potri.010G221700 Potri.006G079600 Potri.001G041700 Potri.013G025900 Potri.004G205000 Potri.008G204900 Potri.005G141600 Potri.001G393200 Potri.013G099500 Potri.004G015500 Potri.005G073300 Potri.005G131600 Potri.001G449100 Potri.006G036500 Potri.012G123700 Potri.010G160200 Potri.019G048800 Potri.012G047400 Potri.011G146300 Potri.008G106700 Potri.006G202700 Potri.012G108500 Potri.006G002100 Potri.014G026500 Potri.010G113900 Potri.001G403900 Potri.004G108200 Potri.015G129500 Potri.001G343100 Potri.002G036200 Potri.012G054700 Potri.008G157400 Potri.007G010000 Potri.006G037300 Potri.019G064300 Potri.011G068500 Potri.011G152400 Potri.009G119700 Potri.004G010500 Potri.002G100600 Potri.001G061100 Potri.005G167800 Potri.007G140800 Potri.018G132100 Potri.006G188300 Potri.008G064700 Potri.001G227300 Potri.009G087600 Potri.010G004200 Potri.003G167700 Potri.011G150700 Potri.002G124800 Potri.003G076300 Potri.014G147300 Potri.001G252100 Potri.002G009300 Potri.016G050500 Potri.007G110600 Potri.001G287200 Potri.001G217700 Potri.T023800 Potri.012G045900 Potri.004G186200 Potri.T096300 Potri.015G074600 Potri.009G026800 Potri.T021900 Potri.011G125200 Potri.015G093100 Potri.016G057400 Potri.016G116000 Potri.016G070500 Potri.005G051600 Potri.002G128300 Potri.014G106300 Potri.001G320000 Potri.011G089700 Potri.016G069400
GO:0019538	protein metabolic process	48	Potri.014G026500 Potri.010G113900 Potri.011G072300 Potri.006G141500 Potri.003G167700 Potri.004G108200 Potri.006G079600 Potri.011G150700 Potri.001G041700 Potri.003G162800 Potri.014G147300 Potri.004G205000 Potri.015G093100 Potri.002G009300 Potri.007G140800 Potri.003G076300 Potri.005G141600 Potri.002G036200 Potri.004G095700 Potri.001G393200 Potri.007G067900 Potri.001G218100 Potri.001G217700 Potri.T023800 Potri.012G054700 Potri.002G104600 Potri.005G254900 Potri.004G186200 Potri.004G015500 Potri.006G036500 Potri.011G146300 Potri.T021900 Potri.019G064300 Potri.006G204700 Potri.008G064700 Potri.004G027400 Potri.011G068500 Potri.005G057500 Potri.016G070500 Potri.011G128700 Potri.005G051600 Potri.019G048800 Potri.012G047400 Potri.001G280200 Potri.011G125200 Potri.006G202700 Potri.007G110600 Potri.016G069400
GO:0006464	protein modification process	35	Potri.010G113900 Potri.011G072300 Potri.006G141500 Potri.004G108200 Potri.006G079600 Potri.003G162800 Potri.014G147300 Potri.004G205000 Potri.015G093100 Potri.002G009300 Potri.007G140800 Potri.007G110600 Potri.002G036200 Potri.004G095700 Potri.001G393200 Potri.007G067900 Potri.001G218100 Potri.001G217700 Potri.T023800 Potri.012G054700 Potri.004G186200 Potri.004G015500 Potri.T021900 Potri.004G027400 Potri.011G068500 Potri.005G057500 Potri.016G070500 Potri.011G128700 Potri.005G051600 Potri.019G048800 Potri.012G047400 Potri.001G280200 Potri.011G125200 Potri.006G202700 Potri.016G069400

GO:0016567	protein ubiquitination	6	Potri.004G205000 Potri.005G057500 Potri.010G113900 Potri.007G110600 Potri.006G202700 Potri.016G069400
GO:0080090	regulation of primary metabolic process	16	Potri.008G157400 Potri.002G100600 Potri.003G111900 Potri.014G106300 Potri.009G119700 Potri.001G287200 Potri.005G039800 Potri.012G108500 Potri.005G131600 Potri.010G004200 Potri.016G050500 Potri.018G057000 Potri.002G124800 Potri.008G106700 Potri.009G139800 Potri.013G025900
GO:0006979	response to oxidative stress	11	Potri.016G125000 Potri.016G132700 Potri.013G156500 Potri.T160100 Potri.006G129900 Potri.001G458700 Potri.015G003500 Potri.005G108900 Potri.001G351000 Potri.014G143200 Potri.001G011500
GO:0023052	signaling	11	Potri.004G027400 Potri.004G201600 Potri.001G252100 Potri.014G103600 Potri.011G128700 Potri.019G048800 Potri.010G027100 Potri.T021900 Potri.013G028300 Potri.011G125200 Potri.T023900
GO:0055085	transmembrane transport	11	Potri.014G130500 Potri.002G078100 Potri.011G043100 Potri.016G111000 Potri.004G215600 Potri.005G246600 Potri.013G065800 Potri.004G019900 Potri.002G099600 Potri.006G110500 Potri.001G185700

Function go term	Description	Matched counts	Matched genes
GO:0030554	adenyl nucleotide binding	46	Potri.012G020800 Potri.005G064300 Potri.011G072300 Potri.006G141500 Potri.011G043100 Potri.004G108200 Potri.006G079600 Potri.005G021100 Potri.009G122100 Potri.014G147300 Potri.016G101100 Potri.014G130500 Potri.015G093100 Potri.002G009300 Potri.009G160600 Potri.002G036200 Potri.004G095700 Potri.001G393200 Potri.007G067900 Potri.001G218100 Potri.011G158700 Potri.T023800 Potri.001G217700 Potri.012G054700 Potri.001G233700 Potri.004G186200 Potri.015G084900 Potri.004G015500 Potri.017G140100 Potri.010G062500 Potri.011G068500 Potri.015G074600 Potri.008G047900 Potri.001G455300 Potri.004G027400 Potri.013G030600 Potri.016G070500 Potri.011G128700 Potri.006G198700 Potri.019G048800 Potri.001G134000 Potri.007G140800 Potri.001G280200 Potri.011G125200 Potri.005G051600 Potri.T021900
GO:0016209	antioxidant activity	14	Potri.006G137500 Potri.016G125000 Potri.002G071000 Potri.016G132700 Potri.013G156500 Potri.001G458700 Potri.T160100 Potri.006G129900 Potri.018G063300 Potri.015G003500 Potri.005G108900 Potri.001G351000 Potri.014G143200 Potri.001G011500
GO:0070001	aspartic-type peptidase activity	5	Potri.019G064300 Potri.001G041700 Potri.003G076300 Potri.002G104600 Potri.006G204700
GO:0005524	ATP binding	43	Potri.009G122100 Potri.005G064300 Potri.011G072300 Potri.006G141500 Potri.011G043100 Potri.004G108200 Potri.006G079600 Potri.005G021100 Potri.012G020800 Potri.014G147300 Potri.016G101100 Potri.014G130500 Potri.015G093100 Potri.002G009300 Potri.009G160600 Potri.002G036200 Potri.004G095700 Potri.001G393200 Potri.007G067900 Potri.001G218100 Potri.001G217700 Potri.T023800 Potri.012G054700 Potri.001G233700 Potri.004G186200 Potri.015G084900 Potri.004G015500 Potri.017G140100 Potri.010G062500 Potri.011G068500 Potri.008G047900 Potri.001G455300 Potri.004G027400 Potri.013G030600 Potri.016G070500 Potri.011G128700 Potri.005G051600 Potri.019G048800 Potri.001G134000 Potri.007G140800 Potri.001G280200 Potri.011G125200 Potri.T021900

GO:0004091	carboxylesterase activity	10	Potri.015G128300 Potri.002G145500 Potri.T107600 Potri.007G107300 Potri.010G109300 Potri.007G108300 Potri.016G001600 Potri.018G132100 Potri.001G173700 Potri.002G202600
GO:0050662	coenzyme binding	10	Potri.001G457000 Potri.006G198700 Potri.001G320000 Potri.011G158700 Potri.015G074600 Potri.008G204900 Potri.002G053000 Potri.012G098300 Potri.003G103700 Potri.013G099500
GO:0030234	enzyme regulator activity	13	Potri.015G128300 Potri.002G145500 Potri.T107600 Potri.019G080600 Potri.007G107300 Potri.010G109300 Potri.007G108300 Potri.016G001600 Potri.002G234600 Potri.009G028300 Potri.002G202600 Potri.003G106400 Potri.016G013700
GO:0020037	heme binding	21	Potri.001G365400 Potri.019G064200 Potri.016G125000 Potri.001G003100 Potri.019G064600 Potri.006G129900 Potri.001G362600 Potri.016G132700 Potri.013G156500 Potri.T160100 Potri.006G094700 Potri.014G143200 Potri.001G458700 Potri.001G365100 Potri.014G102700 Potri.015G003500 Potri.005G108900 Potri.001G351000 Potri.006G266200 Potri.001G364900 Potri.001G011500
GO:0016787	hydrolase activity	57	Potri.001G173700 Potri.001G227300 Potri.014G026500 Potri.005G064300 Potri.015G058400 Potri.011G043100 Potri.001G403900 Potri.006G002100 Potri.005G021100 Potri.009G160600 Potri.009G122100 Potri.003G034600 Potri.001G041700 Potri.014G130500 Potri.001G252100 Potri.T107600 Potri.007G107300 Potri.015G049100 Potri.003G076300 Potri.005G141600 Potri.009G087600 Potri.014G046300 Potri.002G104600 Potri.012G036400 Potri.001G233700 Potri.007G108300 Potri.015G128300 Potri.007G010000 Potri.008G047900 Potri.001G449100 Potri.016G001600 Potri.006G036500 Potri.011G146300 Potri.001G455300 Potri.011G089700 Potri.019G064300 Potri.002G145500 Potri.006G204700 Potri.017G055800 Potri.008G086700 Potri.016G116000 Potri.016G057400 Potri.011G152400 Potri.004G160100 Potri.008G086800 Potri.004G010500 Potri.001G061100 Potri.004G232900 Potri.001G100000 Potri.005G167800 Potri.010G062500 Potri.018G132100 Potri.002G202600 Potri.006G188300 Potri.008G064700 Potri.010G109300 Potri.008G079400
GO:0016798	hydrolase activity, acting on glycosyl bonds	14	Potri.005G167800 Potri.001G227300 Potri.006G002100 Potri.015G049100 Potri.007G010000 Potri.001G449100 Potri.001G100000 Potri.001G403900 Potri.004G010500 Potri.009G087600 Potri.006G188300 Potri.008G079400 Potri.016G057400 Potri.011G152400
GO:0005506	iron ion binding	21	Potri.001G365400 Potri.019G064200 Potri.016G125000 Potri.001G003100 Potri.019G064600 Potri.006G129900 Potri.001G362600 Potri.016G132700 Potri.013G156500 Potri.T160100 Potri.006G094700 Potri.014G143200 Potri.001G458700 Potri.001G365100 Potri.014G102700 Potri.015G003500 Potri.005G108900 Potri.001G351000 Potri.006G266200 Potri.001G364900 Potri.001G011500
GO:0017111	nucleoside-triphosphatase activity	11	Potri.014G130500 Potri.009G160600 Potri.005G064300 Potri.010G062500 Potri.011G043100 Potri.014G046300 Potri.008G047900 Potri.005G021100 Potri.001G455300 Potri.009G122100 Potri.001G233700
GO:0016491	oxidoreductase activity	50	Potri.001G365400 Potri.006G137500 Potri.016G125000 Potri.001G457000 Potri.011G158700 Potri.T160100 Potri.006G094700 Potri.007G040900 Potri.006G129900 Potri.019G064600 Potri.014G102700 Potri.005G108900 Potri.001G011500 Potri.019G121700 Potri.019G064200 Potri.008G161600 Potri.001G003100 Potri.008G204900 Potri.001G458700 Potri.018G063300 Potri.019G045000 Potri.001G113100 Potri.001G351000 Potri.013G099500

			Potri.001G364900 Potri.008G157400 Potri.003G005800 Potri.002G071000 Potri.011G047300 Potri.002G191900 Potri.004G067000 Potri.015G081000 Potri.006G062500 Potri.006G151600 Potri.001G015300 Potri.007G147300 Potri.015G074600 Potri.001G362600 Potri.016G132700 Potri.013G156500 Potri.006G198700 Potri.001G365100 Potri.008G194100 Potri.009G025900 Potri.010G023600 Potri.009G010000 Potri.001G320000 Potri.015G003500 Potri.014G143200 Potri.001G391100
GO:0030599	pectinesterase activity	8	Potri.015G128300 Potri.002G145500 Potri.T107600 Potri.007G107300 Potri.010G109300 Potri.007G108300 Potri.016G001600 Potri.002G202600
GO:0004601	peroxidase activity	11	Potri.016G125000 Potri.016G132700 Potri.013G156500 Potri.T160100 Potri.006G129900 Potri.001G458700 Potri.015G003500 Potri.005G108900 Potri.001G351000 Potri.014G143200 Potri.001G011500
GO:0042578	phosphoric ester hydrolase activity	5	Potri.001G252100 Potri.003G034600 Potri.015G058400 Potri.004G232900 Potri.001G061100
GO:0004672	protein kinase activity	27	Potri.011G072300 Potri.006G141500 Potri.004G108200 Potri.006G079600 Potri.014G147300 Potri.015G093100 Potri.002G009300 Potri.001G218100 Potri.004G095700 Potri.001G393200 Potri.007G067900 Potri.002G036200 Potri.001G217700 Potri.T023800 Potri.012G054700 Potri.004G186200 Potri.004G015500 Potri.T021900 Potri.004G027400 Potri.011G068500 Potri.016G070500 Potri.011G128700 Potri.005G051600 Potri.019G048800 Potri.007G140800 Potri.001G280200 Potri.011G125200
GO:0003700	transcription factor activity	7	Potri.003G111900 Potri.009G119700 Potri.002G100600 Potri.012G108500 Potri.010G004200 Potri.002G124800 Potri.008G106700
GO:0016757	transferase activity, transferring glycosyl groups	12	Potri.014G088300 Potri.001G158000 Potri.006G022300 Potri.007G141700 Potri.016G021600 Potri.006G023700 Potri.013G118700 Potri.016G021100 Potri.010G121800 Potri.005G073800 Potri.010G162100 Potri.010G160200
GO:0016772	transferase activity, transferring phosphorus-containing groups	30	Potri.011G072300 Potri.006G141500 Potri.004G108200 Potri.006G079600 Potri.014G147300 Potri.015G093100 Potri.002G009300 Potri.001G218100 Potri.004G095700 Potri.001G393200 Potri.007G067900 Potri.002G036200 Potri.001G217700 Potri.T023800 Potri.012G054700 Potri.008G102500 Potri.T148900 Potri.004G186200 Potri.004G015500 Potri.T021900 Potri.004G027400 Potri.011G068500 Potri.016G070500 Potri.011G128700 Potri.005G051600 Potri.019G048800 Potri.001G134000 Potri.007G140800 Potri.001G280200 Potri.011G125200
GO:0005215	transporter activity	13	Potri.007G044000 Potri.014G130500 Potri.002G078100 Potri.009G073500 Potri.002G225500 Potri.011G043100 Potri.014G046300 Potri.014G124200 Potri.006G268200 Potri.006G102800 Potri.019G056500 Potri.016G111000 Potri.001G185700
GO:0004842	ubiquitin-protein ligase activity	6	Potri.004G205000 Potri.005G057500 Potri.010G113900 Potri.007G110600 Potri.006G202700 Potri.016G069400

Supplementary Table S2 (cont.): Details of GO terms enriched by up-regulated DEGs of Aig at **96 hpi** of three infection stages in Figure 4

Process go term	Description	Matched counts	Matched genes
GO:0009309	amine biosynthetic process	7	Potri.017G109300 Potri.009G061700 Potri.005G162800 Potri.009G072900 Potri.015G135400 Potri.008G060200 Potri.013G099500
GO:0016051	carbohydrate biosynthetic process	9	Potri.002G127400 Potri.012G128200 Potri.014G080300 Potri.004G059600 Potri.012G047400 Potri.005G135900 Potri.002G066600 Potri.005G201800 Potri.012G047300
GO:0005975	carbohydrate metabolic process	63	Potri.017G063500 Potri.010G141600 Potri.009G084600 Potri.001G227300 Potri.009G087600 Potri.019G093800 Potri.015G058400 Potri.001G325200 Potri.018G123000 Potri.001G403900 Potri.001G255100 Potri.005G257800 Potri.006G063300 Potri.010G040400 Potri.004G059600 Potri.017G130200 Potri.003G131700 Potri.007G089400 Potri.004G081300 Potri.006G188400 Potri.014G080300 Potri.010G152000 Potri.004G184200 Potri.016G054800 Potri.010G125800 Potri.013G060400 Potri.005G135900 Potri.002G066600 Potri.002G094000 Potri.008G108100 Potri.012G047300 Potri.001G171900 Potri.008G120000 Potri.007G015500 Potri.008G094200 Potri.007G010000 Potri.006G037300 Potri.011G155500 Potri.011G015600 Potri.013G095500 Potri.018G112000 Potri.012G128200 Potri.009G006600 Potri.004G019500 Potri.016G057400 Potri.010G160200 Potri.011G152400 Potri.011G094400 Potri.002G093300 Potri.002G127400 Potri.005G201800 Potri.001G222900 Potri.001G061100 Potri.018G024500 Potri.005G167800 Potri.012G047400 Potri.001G409900 Potri.012G045900 Potri.009G153900 Potri.004G123600 Potri.006G188300 Potri.017G139100 Potri.015G041300
GO:0046394	carboxylic acid biosynthetic process	11	Potri.017G109300 Potri.009G061700 Potri.001G463800 Potri.011G010400 Potri.006G218000 Potri.005G162800 Potri.009G072900 Potri.003G135200 Potri.015G135400 Potri.008G060200 Potri.013G099500
GO:0007154	cell communication	9	Potri.004G027400 Potri.T021600 Potri.016G102500 Potri.011G128700 Potri.001G412300 Potri.011G037100 Potri.T022600 Potri.011G125200 Potri.011G037000
GO:0008037	cell recognition	9	Potri.004G027400 Potri.T021600 Potri.016G102500 Potri.011G128700 Potri.001G412300 Potri.011G037100 Potri.T022600 Potri.011G125200 Potri.011G037000
GO:0044036	cell wall macromolecule metabolic process	5	Potri.019G093800 Potri.015G137500 Potri.010G141600 Potri.004G183500 Potri.013G102300
GO:0071554	cell wall organization or biogenesis	11	Potri.015G137500 Potri.010G141600 Potri.013G102300 Potri.013G013200 Potri.007G107300 Potri.018G051400 Potri.004G183500 Potri.002G202600 Potri.019G093800 Potri.002G145500 Potri.002G202500
GO:0044262	cellular carbohydrate metabolic process	27	Potri.017G063500 Potri.015G058400 Potri.013G095500 Potri.005G257800 Potri.010G040400 Potri.004G059600 Potri.007G015500 Potri.004G081300 Potri.014G080300 Potri.004G184200 Potri.005G135900 Potri.002G066600 Potri.018G024500 Potri.012G045900 Potri.001G325200 Potri.006G037300 Potri.011G015600 Potri.009G084600 Potri.012G128200 Potri.009G006600 Potri.013G060400 Potri.011G155500 Potri.002G093300 Potri.002G127400

			Potri.017G139100 Potri.001G061100 Potri.005G201800
GO:0044237	cellular metabolic process	234	Potri.006G193000 Potri.017G063500 Potri.011G072300 Potri.015G058400 Potri.005G100900 Potri.T021400 Potri.019G083200 Potri.002G030900 Potri.018G041000 Potri.019G018100 Potri.001G393200 Potri.003G162800 Potri.008G102500 Potri.010G213000 Potri.009G081400 Potri.003G149700 Potri.014G080300 Potri.T089900 Potri.018G091000 Potri.009G146800 Potri.004G095700 Potri.014G046300 Potri.007G067900 Potri.010G113900 Potri.001G218100 Potri.013G059600 Potri.019G045000 Potri.011G051600 Potri.019G094200 Potri.T148900 Potri.002G226700 Potri.009G107200 Potri.005G162800 Potri.005G039800 Potri.011G068500 Potri.T046300 Potri.013G108700 Potri.003G061700 Potri.006G263500 Potri.018G123000 Potri.002G017000 Potri.013G060400 Potri.004G027400 Potri.T021600 Potri.013G155900 Potri.001G412300 Potri.001G080900 Potri.T064400 Potri.011G128700 Potri.019G099200 Potri.005G257800 Potri.006G069700 Potri.018G057000 Potri.003G060000 Potri.016G069500 Potri.012G045900 Potri.017G108000 Potri.005G201800 Potri.T011000 Potri.010G156300 Potri.011G078000 Potri.009G139800 Potri.017G109300 Potri.001G457000 Potri.001G208600 Potri.006G141500 Potri.017G079500 Potri.019G034700 Potri.006G079600 Potri.013G054200 Potri.010G040400 Potri.001G040000 Potri.004G015500 Potri.002G045000 Potri.013G025900 Potri.006G057500 Potri.005G185500 Potri.008G160200 Potri.010G123500 Potri.011G028100 Potri.004G184200 Potri.001G328000 Potri.012G058700 Potri.005G135900 Potri.003G135200 Potri.013G099500 Potri.008G159100 Potri.009G075900 Potri.009G061700 Potri.008G141800 Potri.011G010400 Potri.017G151400 Potri.019G128600 Potri.005G131600 Potri.013G125500 Potri.011G015600 Potri.T022600 Potri.005G038100 Potri.001G117800 Potri.011G157100 Potri.006G066100 Potri.005G089600 Potri.019G109800 Potri.018G141500 Potri.008G204900 Potri.002G142800 Potri.019G048800 Potri.012G047400 Potri.009G010000 Potri.008G106700 Potri.006G202700 Potri.004G163800 Potri.019G078400 Potri.007G019600 Potri.004G135500 Potri.001G200000 Potri.014G189600 Potri.003G136100 Potri.006G025800 Potri.006G049200 Potri.002G180300 Potri.013G095500 Potri.009G100400 Potri.007G012700 Potri.002G019300 Potri.002G004900 Potri.006G083900 Potri.018G043900 Potri.007G110600 Potri.007G015500 Potri.018G131700 Potri.004G081300 Potri.010G142900 Potri.009G072900 Potri.016G051600 Potri.010G087900 Potri.011G000100 Potri.002G036200 Potri.002G066600 Potri.012G033200 Potri.012G054700 Potri.011G037000 Potri.015G018000 Potri.018G024500 Potri.006G033400 Potri.008G157400 Potri.001G325200 Potri.001G058200 Potri.019G111100 Potri.011G065900 Potri.006G166600 Potri.017G104800 Potri.006G037300 Potri.019G033000 Potri.003G033400 Potri.004G042600 Potri.018G138700 Potri.009G084600 Potri.017G050400 Potri.006G063300 Potri.011G153300 Potri.006G208100 Potri.002G053000 Potri.002G093300 Potri.019G007900 Potri.002G251700 Potri.002G100600 Potri.001G058800 Potri.001G061100 Potri.016G056500 Potri.001G156200 Potri.007G140800 Potri.017G152400 Potri.012G047300 Potri.015G135400 Potri.008G064700 Potri.016G070500 Potri.011G123500 Potri.001G463800 Potri.003G152600 Potri.008G217700 Potri.011G024700 Potri.007G019900 Potri.001G018700 Potri.012G119800 Potri.017G017500 Potri.010G004200 Potri.003G167700 Potri.011G150700 Potri.003G126800 Potri.004G059600 Potri.002G124800 Potri.014G147300 Potri.017G013700 Potri.011G155500 Potri.016G102500 Potri.002G009300 Potri.004G200100

			Potri.010G127300 Potri.012G108500 Potri.013G125600 Potri.013G006500 Potri.004G193000 Potri.001G287200 Potri.011G037100 Potri.001G217700 Potri.T023800 Potri.015G099200 Potri.005G254900 Potri.006G070500 Potri.004G186200 Potri.008G124300 Potri.011G067400 Potri.006G218000 Potri.001G095200 Potri.012G128200 Potri.009G006600 Potri.005G229500 Potri.008G060200 Potri.002G031900 Potri.001G085500 Potri.010G210000 Potri.017G129700 Potri.018G134200 Potri.002G127400 Potri.019G004700 Potri.005G051600 Potri.003G185700 Potri.001G014700 Potri.014G106300 Potri.016G050500 Potri.011G125200 Potri.017G139100 Potri.016G069400
GO:0044264	cellular polysaccharide metabolic process	7	Potri.002G066600 Potri.002G127400 Potri.004G059600 Potri.005G135900 Potri.012G128200 Potri.009G006600 Potri.005G201800
GO:0044267	cellular protein metabolic process	111	Potri.006G193000 Potri.019G099200 Potri.011G072300 Potri.005G100900 Potri.T021400 Potri.019G083200 Potri.018G041000 Potri.019G018100 Potri.003G162800 Potri.T089900 Potri.018G091000 Potri.009G146800 Potri.004G095700 Potri.007G067900 Potri.001G218100 Potri.019G094200 Potri.017G108000 Potri.013G108700 Potri.006G263500 Potri.002G017000 Potri.004G027400 Potri.T021600 Potri.T064400 Potri.011G128700 Potri.011G028100 Potri.016G069500 Potri.005G201800 Potri.011G078000 Potri.006G141500 Potri.006G079600 Potri.010G040400 Potri.001G040000 Potri.006G057500 Potri.007G140800 Potri.008G160200 Potri.004G184200 Potri.001G393200 Potri.009G075900 Potri.005G135900 Potri.T011000 Potri.004G015500 Potri.013G125500 Potri.T022600 Potri.001G117800 Potri.006G066100 Potri.019G109800 Potri.019G048800 Potri.012G047400 Potri.012G119800 Potri.006G202700 Potri.004G135500 Potri.001G200000 Potri.010G113900 Potri.009G100400 Potri.002G019300 Potri.002G004900 Potri.012G058700 Potri.019G078400 Potri.016G051600 Potri.010G087900 Potri.002G036200 Potri.012G033200 Potri.012G054700 Potri.011G037000 Potri.015G018000 Potri.018G024500 Potri.006G033400 Potri.019G111100 Potri.006G166600 Potri.003G033400 Potri.018G138700 Potri.T046300 Potri.019G128600 Potri.011G068500 Potri.019G007900 Potri.002G251700 Potri.005G185500 Potri.017G152400 Potri.017G151400 Potri.008G064700 Potri.019G004700 Potri.003G136100 Potri.008G217700 Potri.001G018700 Potri.017G017500 Potri.003G167700 Potri.011G150700 Potri.014G147300 Potri.016G102500 Potri.002G009300 Potri.010G127300 Potri.007G110600 Potri.013G125600 Potri.004G193000 Potri.011G037100 Potri.001G217700 Potri.T023800 Potri.005G254900 Potri.004G186200 Potri.008G124300 Potri.011G067400 Potri.001G095200 Potri.001G085500 Potri.001G412300 Potri.018G134200 Potri.016G070500 Potri.005G051600 Potri.003G185700 Potri.001G014700 Potri.011G125200 Potri.016G069400
GO:0006091	generation of precursor metabolites and energy	8	Potri.011G024700 Potri.007G015500 Potri.002G093300 Potri.013G060400 Potri.011G015600 Potri.005G257800 Potri.009G010000 Potri.019G045000
GO:0006629	lipid metabolic process	36	Potri.001G463800 Potri.006G228200 Potri.001G332300 Potri.018G123000 Potri.009G046600 Potri.006G063300 Potri.001G173700 Potri.017G102100 Potri.011G089700 Potri.001G252100 Potri.008G204900 Potri.017G133900 Potri.T004800 Potri.006G218000 Potri.010G188900 Potri.010G236800 Potri.007G133500 Potri.011G010400 Potri.005G135900 Potri.002G083700 Potri.T004600 Potri.001G342600 Potri.006G166700 Potri.009G107200 Potri.001G191400 Potri.012G128200 Potri.005G229500 Potri.016G116000 Potri.002G127400 Potri.005G201800

			Potri.002G128300 Potri.012G047400 Potri.018G132100 Potri.016G117500 Potri.003G061700 Potri.010G156300
GO:0043170	macromolecule metabolic process	212	Potri.006G193000 Potri.019G099200 Potri.011G072300 Potri.005G100900 Potri.T021400 Potri.019G083200 Potri.002G030900 Potri.018G041000 Potri.019G018100 Potri.001G393200 Potri.003G162800 Potri.008G102500 Potri.009G081400 Potri.003G149700 Potri.T089900 Potri.018G091000 Potri.009G146800 Potri.004G095700 Potri.007G067900 Potri.010G113900 Potri.001G218100 Potri.013G059600 Potri.011G051600 Potri.019G094200 Potri.T148900 Potri.002G226700 Potri.005G039800 Potri.009G098100 Potri.011G068500 Potri.001G312800 Potri.013G108700 Potri.006G263500 Potri.002G017000 Potri.013G060400 Potri.004G027400 Potri.T021600 Potri.013G155900 Potri.001G080900 Potri.T064400 Potri.011G128700 Potri.005G257800 Potri.018G057000 Potri.003G060000 Potri.016G069500 Potri.017G108000 Potri.005G201800 Potri.011G078000 Potri.009G139800 Potri.015G137500 Potri.019G093800 Potri.006G141500 Potri.017G079500 Potri.019G034700 Potri.014G018900 Potri.006G079600 Potri.013G054200 Potri.010G040400 Potri.001G040000 Potri.002G045000 Potri.001G041700 Potri.013G025900 Potri.006G057500 Potri.005G185500 Potri.008G160200 Potri.004G183500 Potri.003G195500 Potri.010G123500 Potri.011G028100 Potri.004G184200 Potri.001G328000 Potri.012G058700 Potri.005G135900 Potri.011G066800 Potri.002G104600 Potri.T011000 Potri.003G136100 Potri.013G102300 Potri.009G075900 Potri.004G015500 Potri.019G128600 Potri.005G131600 Potri.010G096600 Potri.013G125500 Potri.011G015600 Potri.009G055900 Potri.001G117800 Potri.011G157100 Potri.006G066100 Potri.019G109800 Potri.013G120200 Potri.018G141500 Potri.002G142800 Potri.019G048800 Potri.012G047400 Potri.T022600 Potri.008G106700 Potri.006G202700 Potri.004G163800 Potri.019G078400 Potri.007G019600 Potri.004G135500 Potri.001G200000 Potri.014G189600 Potri.014G026500 Potri.006G025800 Potri.006G049200 Potri.002G180300 Potri.009G100400 Potri.007G012700 Potri.002G019300 Potri.003G152600 Potri.002G004900 Potri.018G043900 Potri.007G110600 Potri.007G015500 Potri.010G142900 Potri.007G072300 Potri.016G051600 Potri.010G087900 Potri.001G212800 Potri.002G036200 Potri.002G066600 Potri.012G033200 Potri.006G153300 Potri.012G054700 Potri.011G037000 Potri.015G018000 Potri.018G024500 Potri.006G033400 Potri.008G157400 Potri.001G412300 Potri.001G058200 Potri.019G111100 Potri.011G155500 Potri.006G166600 Potri.017G104800 Potri.006G207900 Potri.003G033400 Potri.004G042600 Potri.018G138700 Potri.019G064300 Potri.T046300 Potri.011G153300 Potri.006G208100 Potri.002G093300 Potri.019G007900 Potri.002G251700 Potri.018G015100 Potri.002G100600 Potri.001G058800 Potri.001G208600 Potri.016G056500 Potri.001G156200 Potri.007G140800 Potri.019G033000 Potri.017G152400 Potri.012G047300 Potri.017G151400 Potri.008G064700 Potri.016G070500 Potri.011G123500 Potri.010G141600 Potri.008G217700 Potri.007G019900 Potri.001G018700 Potri.012G119800 Potri.017G017500 Potri.010G004200 Potri.003G167700 Potri.011G150700 Potri.008G203200 Potri.004G059600 Potri.002G124800 Potri.014G147300 Potri.017G013700 Potri.014G106300 Potri.016G102500 Potri.002G009300 Potri.016G050500 Potri.010G127300 Potri.012G108500 Potri.013G125600 Potri.004G193000 Potri.001G287200 Potri.011G037100 Potri.017G133200 Potri.001G217700 Potri.T023800 Potri.002G054900 Potri.015G099200 Potri.005G254900 Potri.004G186200 Potri.008G124300 Potri.011G067400 Potri.001G095200 Potri.012G128200 Potri.009G006600 Potri.010G220100

			Potri.002G031900 Potri.001G085500 Potri.004G208100 Potri.011G065900 Potri.018G134200 Potri.002G127400 Potri.019G004700 Potri.005G051600 Potri.003G185700 Potri.001G014700 Potri.017G084000 Potri.001G240600 Potri.011G125200 Potri.016G069400
GO:0051704	multi-organism process	11	Potri.004G027400 Potri.T021600 Potri.016G102500 Potri.011G128700 Potri.001G412300 Potri.012G047400 Potri.011G037100 Potri.T022600 Potri.011G125200 Potri.011G037000 Potri.012G047300
GO:0055114	oxidation reduction	91	Potri.016G125000 Potri.001G242600 Potri.006G094700 Potri.011G071100 Potri.001G463300 Potri.001G461800 Potri.013G053100 Potri.018G063300 Potri.019G045000 Potri.001G113100 Potri.001G451800 Potri.010G168200 Potri.001G176500 Potri.004G067000 Potri.006G062500 Potri.001G015300 Potri.001G270900 Potri.011G117600 Potri.016G132700 Potri.006G198700 Potri.007G143600 Potri.006G069700 Potri.001G098300 Potri.004G017700 Potri.017G109300 Potri.006G137500 Potri.011G150100 Potri.006G228200 Potri.001G457000 Potri.004G149000 Potri.001G331100 Potri.007G084700 Potri.008G204900 Potri.001G452600 Potri.005G143800 Potri.013G099500 Potri.011G020900 Potri.016G117500 Potri.018G046600 Potri.009G101700 Potri.005G089600 Potri.013G064200 Potri.001G459200 Potri.001G459100 Potri.011G161600 Potri.011G158700 Potri.001G462200 Potri.009G010000 Potri.T045500 Potri.011G047300 Potri.005G108900 Potri.019G064200 Potri.018G131700 Potri.008G161600 Potri.001G003100 Potri.010G193100 Potri.005G043400 Potri.003G219000 Potri.008G157400 Potri.007G106500 Potri.005G135300 Potri.011G158100 Potri.019G064600 Potri.013G156500 Potri.002G127400 Potri.003G214900 Potri.007G084200 Potri.015G135400 Potri.001G219300 Potri.001G391100 Potri.001G463800 Potri.008G106400 Potri.001G394400 Potri.004G015300 Potri.007G126600 Potri.007G049900 Potri.004G075800 Potri.001G015400 Potri.016G112000 Potri.005G079400 Potri.012G128200 Potri.005G229500 Potri.011G155900 Potri.001G462000 Potri.001G090000 Potri.009G064900 Potri.009G025900 Potri.001G270800 Potri.006G129900 Potri.015G003500 Potri.011G158500
GO:0006793	phosphorus metabolic process	90	Potri.006G193000 Potri.019G099200 Potri.011G072300 Potri.005G100900 Potri.T021400 Potri.019G083200 Potri.018G041000 Potri.019G018100 Potri.T089900 Potri.018G091000 Potri.009G146800 Potri.004G095700 Potri.007G067900 Potri.001G218100 Potri.019G045000 Potri.019G094200 Potri.011G024700 Potri.006G263500 Potri.002G017000 Potri.004G027400 Potri.T021600 Potri.T064400 Potri.011G128700 Potri.011G028100 Potri.017G108000 Potri.005G201800 Potri.010G156300 Potri.006G141500 Potri.006G079600 Potri.001G040000 Potri.006G057500 Potri.007G140800 Potri.008G160200 Potri.001G393200 Potri.009G075900 Potri.005G135900 Potri.T011000 Potri.004G015500 Potri.T022600 Potri.001G117800 Potri.006G066100 Potri.019G109800 Potri.019G048800 Potri.009G010000 Potri.004G135500 Potri.001G200000 Potri.009G100400 Potri.002G019300 Potri.002G004900 Potri.019G078400 Potri.016G051600 Potri.010G087900 Potri.002G036200 Potri.012G033200 Potri.012G054700 Potri.011G037000 Potri.015G018000 Potri.019G111100 Potri.006G166600 Potri.001G412300 Potri.018G138700 Potri.019G128600 Potri.011G068500 Potri.019G007900 Potri.002G251700 Potri.005G185500 Potri.017G152400 Potri.017G151400 Potri.019G004700 Potri.003G136100 Potri.001G018700 Potri.014G147300 Potri.016G102500 Potri.002G009300 Potri.004G193000 Potri.011G037100 Potri.001G217700 Potri.T023800

			Potri.006G070500 Potri.004G186200 Potri.008G124300 Potri.011G067400 Potri.001G095200 Potri.001G085500 Potri.018G134200 Potri.016G070500 Potri.005G051600 Potri.003G185700 Potri.001G014700 Potri.011G125200
GO:0016310	phosphorylation	89	Potri.006G193000 Potri.019G099200 Potri.011G072300 Potri.005G100900 Potri.T021400 Potri.019G083200 Potri.018G041000 Potri.019G018100 Potri.T089900 Potri.018G091000 Potri.019G078400 Potri.004G095700 Potri.007G067900 Potri.001G218100 Potri.019G045000 Potri.019G094200 Potri.011G024700 Potri.006G263500 Potri.002G017000 Potri.004G027400 Potri.T021600 Potri.T064400 Potri.011G128700 Potri.011G028100 Potri.017G108000 Potri.005G201800 Potri.010G156300 Potri.006G141500 Potri.006G079600 Potri.001G040000 Potri.006G057500 Potri.007G140800 Potri.008G160200 Potri.001G393200 Potri.009G075900 Potri.005G135900 Potri.T011000 Potri.004G015500 Potri.T022600 Potri.001G117800 Potri.006G066100 Potri.019G109800 Potri.019G048800 Potri.009G010000 Potri.004G135500 Potri.001G200000 Potri.009G100400 Potri.002G019300 Potri.002G004900 Potri.016G051600 Potri.010G087900 Potri.002G036200 Potri.012G033200 Potri.012G054700 Potri.011G037000 Potri.015G018000 Potri.019G111100 Potri.006G166600 Potri.001G412300 Potri.018G138700 Potri.019G128600 Potri.011G068500 Potri.019G007900 Potri.002G251700 Potri.005G185500 Potri.017G152400 Potri.017G151400 Potri.019G004700 Potri.003G136100 Potri.001G018700 Potri.014G147300 Potri.016G102500 Potri.002G009300 Potri.004G193000 Potri.011G037100 Potri.001G217700 Potri.T023800 Potri.006G070500 Potri.004G186200 Potri.008G124300 Potri.011G067400 Potri.001G095200 Potri.001G085500 Potri.018G134200 Potri.016G070500 Potri.005G051600 Potri.003G185700 Potri.001G014700 Potri.011G125200
GO:0005976	polysaccharide metabolic process	9	Potri.002G066600 Potri.010G141600 Potri.002G127400 Potri.019G093800 Potri.012G128200 Potri.005G135900 Potri.004G059600 Potri.009G006600 Potri.005G201800
GO:0044238	primary metabolic process	296	Potri.006G193000 Potri.017G063500 Potri.017G102100 Potri.001G332300 Potri.015G058400 Potri.005G100900 Potri.T021400 Potri.019G083200 Potri.002G030900 Potri.009G046600 Potri.018G041000 Potri.001G173700 Potri.019G018100 Potri.001G393200 Potri.003G162800 Potri.008G102500 Potri.010G213000 Potri.011G089700 Potri.004G015500 Potri.003G149700 Potri.014G080300 Potri.T089900 Potri.018G091000 Potri.009G146800 Potri.004G095700 Potri.014G046300 Potri.007G067900 Potri.016G054800 Potri.001G218100 Potri.013G059600 Potri.T004800 Potri.006G218000 Potri.002G094000 Potri.011G051600 Potri.008G108100 Potri.019G094200 Potri.T148900 Potri.002G226700 Potri.001G171900 Potri.011G072300 Potri.009G107200 Potri.005G162800 Potri.005G039800 Potri.019G078400 Potri.009G098100 Potri.011G068500 Potri.T046300 Potri.001G312800 Potri.013G108700 Potri.003G061700 Potri.001G191400 Potri.018G123000 Potri.001G255100 Potri.001G208600 Potri.002G017000 Potri.019G007900 Potri.004G027400 Potri.T021600 Potri.013G155900 Potri.001G412300 Potri.001G080900 Potri.T064400 Potri.013G099500 Potri.011G128700 Potri.019G099200 Potri.005G257800 Potri.010G113900 Potri.008G094200 Potri.011G028100 Potri.003G060000 Potri.016G069500 Potri.007G133500 Potri.016G117500 Potri.017G108000 Potri.005G201800 Potri.T011000 Potri.010G156300 Potri.011G078000 Potri.009G139800 Potri.017G109300 Potri.006G188400 Potri.006G228200 Potri.001G457000 Potri.019G093800 Potri.006G141500 Potri.017G079500 Potri.011G094400 Potri.019G034700 Potri.014G018900 Potri.006G079600

			Potri.013G054200 Potri.010G040400 Potri.001G040000 Potri.017G130200 Potri.002G045000 Potri.001G041700
			Potri.013G025900 Potri.006G057500 Potri.005G185500 Potri.008G160200 Potri.004G123600 Potri.003G195500
			Potri.010G123500 Potri.018G057000 Potri.004G184200 Potri.001G328000 Potri.013G060400 Potri.010G220100
			Potri.010G125800 Potri.012G058700 Potri.005G135900 Potri.001G156200 Potri.011G066800 Potri.002G104600
			Potri.008G159100 Potri.003G136100 Potri.009G075900 Potri.009G061700 Potri.011G010400 Potri.009G153900
			Potri.T004600 Potri.005G131600 Potri.010G096600 Potri.013G125500 Potri.011G015600 Potri.009G055900
			Potri.005G038100 Potri.018G112000 Potri.001G117800 Potri.011G157100 Potri.007G072300 Potri.010G160200
			Potri.019G109800 Potri.013G120200 Potri.006G166700 Potri.012G047300 Potri.002G142800 Potri.019G048800
			Potri.012G047400 Potri.001G409900 Potri.T022600 Potri.008G106700 Potri.006G202700 Potri.004G163800
			Potri.015G041300 Potri.007G019600 Potri.004G135500 Potri.001G200000 Potri.014G189600 Potri.014G026500
			Potri.006G025800 Potri.017G151400 Potri.006G049200 Potri.002G180300 Potri.001G222900 Potri.001G403900
			Potri.013G095500 Potri.009G100400 Potri.007G012700 Potri.017G133900 Potri.002G019300 Potri.003G152600
			Potri.002G004900 Potri.018G043900 Potri.007G110600 Potri.007G015500 Potri.004G081300 Potri.010G142900
			Potri.009G072900 Potri.006G066100 Potri.016G051600 Potri.010G087900 Potri.001G212800 Potri.002G036200
			Potri.011G000100 Potri.002G066600 Potri.012G033200 Potri.009G081400 Potri.012G054700 Potri.011G037000
			Potri.015G018000 Potri.010G236800 Potri.008G120000 Potri.006G033400 Potri.008G157400 Potri.004G208100
			Potri.001G325200 Potri.001G058200 Potri.019G111100 Potri.002G083700 Potri.006G166600 Potri.017G104800
			Potri.007G010000 Potri.006G037300 Potri.001G342600 Potri.006G207900 Potri.003G033400 Potri.004G042600
			Potri.018G138700 Potri.009G084600 Potri.006G153300 Potri.019G064300 Potri.003G131700 Potri.006G063300
			Potri.011G153300 Potri.006G208100 Potri.004G019500 Potri.002G093300 Potri.011G152400 Potri.002G251700
			Potri.018G015100 Potri.002G100600 Potri.001G058800 Potri.018G141500 Potri.001G061100 Potri.016G056500
			Potri.018G024500 Potri.005G167800 Potri.007G140800 Potri.019G033000 Potri.017G152400 Potri.018G132100
			Potri.015G135400 Potri.006G188300 Potri.008G064700 Potri.016G070500 Potri.011G123500 Potri.001G463800
			Potri.010G141600 Potri.014G106300 Potri.001G227300 Potri.009G087600 Potri.011G024700 Potri.007G019900
			Potri.001G018700 Potri.012G119800 Potri.017G017500 Potri.010G004200 Potri.003G167700 Potri.011G150700
			Potri.003G126800 Potri.008G217700 Potri.004G059600 Potri.002G124800 Potri.017G084000 Potri.014G147300
			Potri.017G013700 Potri.007G089400 Potri.011G155500 Potri.016G102500 Potri.002G009300 Potri.016G050500
			Potri.010G127300 Potri.012G108500 Potri.013G125600 Potri.004G193000 Potri.001G287200 Potri.011G037100
			Potri.017G133200 Potri.001G217700 Potri.T023800 Potri.010G188900 Potri.002G054900 Potri.015G099200
			Potri.005G254900 Potri.004G186200 Potri.008G124300 Potri.011G067400 Potri.012G045900 Potri.019G128600
			Potri.001G095200 Potri.002G031900 Potri.008G203200 Potri.012G128200 Potri.009G006600 Potri.001G252100
			Potri.005G229500 Potri.008G060200 Potri.016G057400 Potri.001G085500 Potri.016G116000 Potri.011G065900
			Potri.018G134200 Potri.002G127400 Potri.019G004700 Potri.005G051600 Potri.003G185700 Potri.001G014700
			Potri.002G128300 Potri.001G240600 Potri.008G204900 Potri.010G152000 Potri.011G125200 Potri.017G139100

			Potri.016G069400 Potri.006G263500
GO:0019538	protein metabolic process	135	Potri.006G193000 Potri.019G099200 Potri.011G072300 Potri.005G100900 Potri.T021400 Potri.019G083200 Potri.018G041000 Potri.019G018100 Potri.003G162800 Potri.T089900 Potri.018G091000 Potri.009G146800 Potri.004G095700 Potri.007G067900 Potri.001G218100 Potri.019G094200 Potri.017G108000 Potri.009G098100 Potri.001G312800 Potri.013G108700 Potri.006G263500 Potri.002G017000 Potri.004G027400 Potri.T021600 Potri.T064400 Potri.011G128700 Potri.011G028100 Potri.016G069500 Potri.005G201800 Potri.011G078000 Potri.006G141500 Potri.014G018900 Potri.006G079600 Potri.010G040400 Potri.001G040000 Potri.001G041700 Potri.006G057500 Potri.005G185500 Potri.008G160200 Potri.003G195500 Potri.004G184200 Potri.001G393200 Potri.009G075900 Potri.005G135900 Potri.011G066800 Potri.002G104600 Potri.T011000 Potri.004G015500 Potri.010G096600 Potri.013G125500 Potri.009G055900 Potri.001G117800 Potri.006G066100 Potri.019G109800 Potri.013G120200 Potri.019G048800 Potri.012G047400 Potri.T022600 Potri.012G119800 Potri.006G202700 Potri.004G135500 Potri.001G200000 Potri.014G026500 Potri.010G113900 Potri.009G100400 Potri.002G019300 Potri.002G004900 Potri.012G058700 Potri.019G078400 Potri.007G072300 Potri.016G051600 Potri.010G087900 Potri.001G212800 Potri.002G036200 Potri.012G033200 Potri.006G153300 Potri.012G054700 Potri.011G037000 Potri.015G018000 Potri.018G024500 Potri.006G033400 Potri.019G111100 Potri.006G166600 Potri.006G207900 Potri.003G033400 Potri.018G138700 Potri.019G064300 Potri.T046300 Potri.019G128600 Potri.011G068500 Potri.019G007900 Potri.002G251700 Potri.018G015100 Potri.007G140800 Potri.017G152400 Potri.017G151400 Potri.008G064700 Potri.016G070500 Potri.003G136100 Potri.008G217700 Potri.001G018700 Potri.017G017500 Potri.003G167700 Potri.011G150700 Potri.008G203200 Potri.014G147300 Potri.016G102500 Potri.002G009300 Potri.010G127300 Potri.007G110600 Potri.013G125600 Potri.004G193000 Potri.011G037100 Potri.017G133200 Potri.001G217700 Potri.T023800 Potri.002G054900 Potri.005G254900 Potri.004G186200 Potri.008G124300 Potri.011G067400 Potri.001G095200 Potri.010G220100 Potri.001G085500 Potri.004G208100 Potri.001G412300 Potri.018G134200 Potri.019G004700 Potri.005G051600 Potri.003G185700 Potri.001G014700 Potri.017G084000 Potri.001G240600 Potri.011G125200 Potri.016G069400
GO:0006464	protein modification process	96	Potri.006G193000 Potri.019G099200 Potri.011G072300 Potri.005G100900 Potri.T021400 Potri.019G083200 Potri.018G041000 Potri.019G018100 Potri.003G162800 Potri.T089900 Potri.018G091000 Potri.009G146800 Potri.004G095700 Potri.007G067900 Potri.001G218100 Potri.019G094200 Potri.017G108000 Potri.006G263500 Potri.002G017000 Potri.004G027400 Potri.T021600 Potri.T064400 Potri.011G128700 Potri.011G028100 Potri.016G069500 Potri.005G201800 Potri.011G078000 Potri.006G141500 Potri.006G079600 Potri.010G040400 Potri.001G040000 Potri.006G057500 Potri.007G140800 Potri.008G160200 Potri.004G184200 Potri.001G393200 Potri.009G075900 Potri.005G135900 Potri.T011000 Potri.004G015500 Potri.T022600 Potri.001G117800 Potri.006G066100 Potri.019G109800 Potri.019G048800 Potri.012G047400 Potri.006G202700 Potri.004G135500 Potri.001G200000 Potri.010G113900 Potri.009G100400 Potri.002G019300 Potri.002G004900 Potri.019G078400 Potri.016G051600 Potri.010G087900 Potri.002G036200 Potri.012G033200 Potri.012G054700 Potri.011G037000

			Potri.015G018000 Potri.018G024500 Potri.019G111100 Potri.006G166600 Potri.001G412300 Potri.018G138700<br>Potri.019G128600 Potri.011G068500 Potri.019G007900 Potri.002G251700 Potri.005G185500 Potri.017G152400<br>Potri.017G151400 Potri.019G004700 Potri.003G136100 Potri.001G018700 Potri.014G147300 Potri.016G102500<br>Potri.002G009300 Potri.007G110600 Potri.004G193000 Potri.011G037100 Potri.001G217700 Potri.T023800<br>Potri.004G186200 Potri.008G124300 Potri.011G067400 Potri.001G095200 Potri.001G085500 Potri.018G134200<br>Potri.016G070500 Potri.005G051600 Potri.003G185700 Potri.001G014700 Potri.011G125200 Potri.016G069400
GO:0016567	protein ubiquitination	5	Potri.010G113900 Potri.006G202700 Potri.016G069500 Potri.016G069400 Potri.007G110600
GO:0080090	regulation of primary metabolic process	50	Potri.011G123500 Potri.006G025800 Potri.001G208600 Potri.006G049200 Potri.002G180300 Potri.010G004200<br>Potri.019G034700 Potri.002G030900 Potri.013G054200 Potri.002G045000 Potri.002G124800 Potri.018G043900<br>Potri.013G025900 Potri.017G013700 Potri.009G081400 Potri.003G149700 Potri.010G142900 Potri.016G050500<br>Potri.012G108500 Potri.007G012700 Potri.001G328000 Potri.001G287200 Potri.013G059600 Potri.007G019900<br>Potri.011G051600 Potri.015G099200 Potri.008G157400 Potri.017G079500 Potri.001G058200 Potri.005G039800<br>Potri.017G104800 Potri.005G131600 Potri.019G033000 Potri.004G042600 Potri.011G157100 Potri.002G031900<br>Potri.011G153300 Potri.006G208100 Potri.011G065900 Potri.001G080900 Potri.018G141500 Potri.002G100600<br>Potri.001G058800 Potri.002G142800 Potri.016G056500 Potri.014G106300 Potri.018G057000 Potri.008G106700<br>Potri.004G163800 Potri.009G139800
GO:0009605	response to external stimulus	8	Potri.010G075200 Potri.011G110100 Potri.010G075300 Potri.010G075700 Potri.016G079000 Potri.009G028300<br>Potri.006G212000 Potri.010G075800
GO:0006979	response to oxidative stress	12	Potri.016G125000 Potri.005G135300 Potri.016G132700 Potri.013G156500 Potri.003G214900 Potri.006G129900<br>Potri.007G126600 Potri.004G015300 Potri.008G106400 Potri.015G003500 Potri.005G108900 Potri.T045500
GO:0009611	response to wounding	8	Potri.010G075200 Potri.011G110100 Potri.010G075300 Potri.010G075700 Potri.016G079000 Potri.009G028300<br>Potri.006G212000 Potri.010G075800
GO:0023052	signaling	29	Potri.T129300 Potri.005G215700 Potri.010G027100 Potri.009G046600 Potri.016G102500 Potri.008G160200<br>Potri.014G103600 Potri.011G037100 Potri.010G188900 Potri.011G014100 Potri.011G037000 Potri.004G201600<br>Potri.005G075300 Potri.T021600 Potri.T005100 Potri.001G412300 Potri.T022600 Potri.006G263500<br>Potri.013G028300 Potri.001G252100 Potri.016G010300 Potri.019G002500 Potri.004G027400 Potri.004G230000<br>Potri.011G128700 Potri.019G048800 Potri.011G125200 Potri.016G097800 Potri.019G002600
GO:0055085	transmembrane transport	34	Potri.002G078100 Potri.001G160400 Potri.002G005500 Potri.001G318500 Potri.008G126500 Potri.016G111000<br>Potri.006G110500 Potri.002G092500 Potri.010G055200 Potri.005G256100 Potri.002G099600 Potri.005G102800<br>Potri.005G246600 Potri.017G151100 Potri.006G158900 Potri.001G185700 Potri.011G024700 Potri.005G150400<br>Potri.012G019700 Potri.014G146800 Potri.012G043000 Potri.014G151800 Potri.001G094600 Potri.016G053600<br>Potri.010G026500 Potri.014G130500 Potri.001G010300 Potri.014G136500 Potri.010G211300 Potri.011G043100<br>Potri.017G135400 Potri.013G065800 Potri.006G006800 Potri.004G019900

Function go term	Description	Matched counts	Matched genes
GO:0003993	acid phosphatase activity	5	Potri.001G191000 Potri.006G110900 Potri.010G184600 Potri.016G139700 Potri.004G232900
GO:0022804	active transmembrane transporter activity	14	Potri.011G024700 Potri.014G130500 Potri.014G116000 Potri.010G055200 Potri.002G078100 Potri.011G043100 Potri.014G046300 Potri.005G102800 Potri.016G053600 Potri.012G081800 Potri.008G126500 Potri.001G094600 Potri.015G077600 Potri.008G159100
GO:0030554	adenyl nucleotide binding	151	Potri.006G193000 Potri.019G099200 Potri.005G064300 Potri.011G072300 Potri.005G100900 Potri.T021400 Potri.019G083200 Potri.018G041000 Potri.019G018100 Potri.001G463300 Potri.013G109600 Potri.T130200 Potri.010G055200 Potri.001G461800 Potri.T089900 Potri.018G091000 Potri.019G078400 Potri.004G095700 Potri.007G067900 Potri.001G218100 Potri.011G158100 Potri.019G094200 Potri.017G108000 Potri.010G168200 Potri.T149700 Potri.010G062500 Potri.013G030600 Potri.001G455300 Potri.002G017000 Potri.004G027400 Potri.T021600 Potri.001G406100 Potri.T064400 Potri.010G228600 Potri.004G159000 Potri.011G128700 Potri.006G198700 Potri.011G028100 Potri.009G100400 Potri.001G098300 Potri.005G201800 Potri.006G141500 Potri.T027200 Potri.006G079600 Potri.001G000800 Potri.001G040000 Potri.006G057500 Potri.016G101100 Potri.014G130500 Potri.005G185500 Potri.008G160200 Potri.005G038100 Potri.006G263500 Potri.001G393200 Potri.011G139100 Potri.009G075900 Potri.005G135900 Potri.019G018800 Potri.T011000 Potri.004G015500 Potri.001G445700 Potri.T022600 Potri.001G117800 Potri.001G348200 Potri.006G066100 Potri.001G435100 Potri.019G002500 Potri.001G459200 Potri.019G109800 Potri.014G001900 Potri.011G161600 Potri.011G158700 Potri.019G048800 Potri.001G462200 Potri.007G019600 Potri.004G135500 Potri.001G200000 Potri.001G433700 Potri.015G084900 Potri.002G019300 Potri.002G004900 Potri.T060300 Potri.016G051600 Potri.010G087900 Potri.002G036200 Potri.012G033200 Potri.012G054700 Potri.011G037000 Potri.003G099000 Potri.001G233700 Potri.017G140100 Potri.006G166600 Potri.T005100 Potri.003G033400 Potri.018G138700 Potri.009G084600 Potri.006G169000 Potri.008G073500 Potri.T046300 Potri.019G128600 Potri.011G068500 Potri.001G311300 Potri.019G007900 Potri.002G251700 Potri.015G018000 Potri.017G083200 Potri.011G043100 Potri.007G140800 Potri.017G152400 Potri.017G151400 Potri.001G459100 Potri.016G070500 Potri.003G136100 Potri.005G051600 Potri.001G018700 Potri.T129300 Potri.014G003200 Potri.019G111100 Potri.008G047900 Potri.016G102500 Potri.002G009300 Potri.009G160600 Potri.001G156200 Potri.004G193000 Potri.011G037100 Potri.001G217700 Potri.T023800 Potri.017G132100 Potri.014G147300 Potri.001G416300 Potri.006G070500 Potri.004G186200 Potri.008G124300 Potri.011G067400 Potri.001G095200 Potri.001G434000 Potri.011G155900 Potri.001G085500 Potri.014G005600 Potri.001G462000 Potri.001G412300 Potri.018G134200 Potri.019G004700 Potri.018G132000 Potri.003G185700 Potri.001G014700 Potri.017G084000 Potri.001G134000 Potri.011G125200 Potri.011G158500 Potri.019G002600
GO:0016209	antioxidant activity	16	Potri.006G137500 Potri.016G125000 Potri.005G135300 Potri.016G132700 Potri.013G156500 Potri.003G214900 Potri.018G063300 Potri.007G126600 Potri.006G129900 Potri.004G015300 Potri.008G106400 Potri.001G098300

			Potri.015G003500 Potri.005G108900 Potri.005G089600 Potri.T045500
GO:0070001	aspartic-type peptidase activity	8	Potri.001G041700 Potri.003G195500 Potri.001G240600 Potri.018G015100 Potri.008G203200 Potri.019G064300 Potri.002G054900 Potri.002G104600
GO:0005524	ATP binding	137	Potri.006G193000 Potri.019G099200 Potri.005G064300 Potri.011G072300 Potri.005G100900 Potri.T021400 Potri.019G083200 Potri.018G041000 Potri.019G018100 Potri.013G109600 Potri.T130200 Potri.010G055200 Potri.T089900 Potri.018G091000 Potri.019G078400 Potri.004G095700 Potri.007G067900 Potri.001G218100 Potri.019G094200 Potri.017G108000 Potri.T149700 Potri.010G062500 Potri.T021600 Potri.001G455300 Potri.002G017000 Potri.004G027400 Potri.013G030600 Potri.001G406100 Potri.T064400 Potri.010G228600 Potri.004G159000 Potri.011G128700 Potri.011G028100 Potri.009G100400 Potri.005G201800 Potri.006G141500 Potri.T027200 Potri.006G079600 Potri.001G000800 Potri.001G040000 Potri.006G057500 Potri.016G101100 Potri.014G130500 Potri.005G185500 Potri.008G160200 Potri.005G038100 Potri.006G263500 Potri.001G393200 Potri.011G139100 Potri.009G075900 Potri.005G135900 Potri.019G018800 Potri.T011000 Potri.004G015500 Potri.001G445700 Potri.T022600 Potri.001G117800 Potri.001G348200 Potri.006G066100 Potri.001G435100 Potri.019G002500 Potri.019G109800 Potri.008G073500 Potri.019G048800 Potri.007G019600 Potri.004G135500 Potri.001G200000 Potri.001G433700 Potri.015G084900 Potri.002G019300 Potri.002G004900 Potri.T060300 Potri.016G051600 Potri.010G087900 Potri.002G036200 Potri.012G033200 Potri.012G054700 Potri.011G037000 Potri.003G099000 Potri.001G233700 Potri.019G111100 Potri.006G166600 Potri.T005100 Potri.003G033400 Potri.018G138700 Potri.009G084600 Potri.006G169000 Potri.014G001900 Potri.T046300 Potri.019G128600 Potri.011G068500 Potri.001G311300 Potri.019G007900 Potri.002G251700 Potri.015G018000 Potri.017G083200 Potri.011G043100 Potri.007G140800 Potri.017G152400 Potri.017G151400 Potri.016G070500 Potri.003G136100 Potri.018G132000 Potri.001G018700 Potri.T129300 Potri.014G003200 Potri.017G140100 Potri.008G047900 Potri.016G102500 Potri.002G009300 Potri.009G160600 Potri.001G156200 Potri.004G193000 Potri.011G037100 Potri.001G217700 Potri.T023800 Potri.017G132100 Potri.014G147300 Potri.001G416300 Potri.006G070500 Potri.004G186200 Potri.008G124300 Potri.011G067400 Potri.001G095200 Potri.001G434000 Potri.001G085500 Potri.014G005600 Potri.001G412300 Potri.018G134200 Potri.019G004700 Potri.005G051600 Potri.003G185700 Potri.001G014700 Potri.017G084000 Potri.001G134000 Potri.011G125200 Potri.019G002600
GO:0005509	calcium ion binding	8	Potri.010G210000 Potri.001G024800 Potri.017G129700 Potri.013G006500 Potri.003G185700 Potri.005G215700 Potri.018G132100 Potri.001G040000
GO:0004091	carboxylesterase activity	15	Potri.T004600 Potri.008G102600 Potri.T107600 Potri.013G013200 Potri.001G119300 Potri.007G107300 Potri.007G108300 Potri.018G051400 Potri.T004800 Potri.001G173700 Potri.002G202600 Potri.002G145500 Potri.003G113600 Potri.002G202500 Potri.018G132100
GO:0004180	carboxypeptidase activity	5	Potri.007G072300 Potri.001G312800 Potri.009G055900 Potri.013G120200 Potri.010G220100
GO:0050662	coenzyme binding	28	Potri.001G457000 Potri.011G158700 Potri.001G098300 Potri.T143400 Potri.006G083900 Potri.001G463300

			Potri.004G200100 Potri.001G461800 Potri.002G053000 Potri.012G098300 Potri.011G158100 Potri.013G099500 Potri.001G459200 Potri.010G168200 Potri.008G141800 Potri.018G046600 Potri.012G128200 Potri.005G229500 Potri.011G155900 Potri.011G161600 Potri.001G462000 Potri.002G127400 Potri.006G198700 Potri.008G204900 Potri.001G462200 Potri.015G135400 Potri.001G459100 Potri.011G158500
GO:0030234	enzyme regulator activity	34	Potri.010G075200 Potri.019G011000 Potri.019G080600 Potri.013G013200 Potri.007G111700 Potri.010G196000 Potri.006G212000 Potri.002G202500 Potri.019G121900 Potri.008G102600 Potri.T107600 Potri.011G110100 Potri.001G119300 Potri.019G006900 Potri.004G000400 Potri.009G028300 Potri.010G075300 Potri.016G079000 Potri.010G075800 Potri.001G225800 Potri.004G067900 Potri.017G153600 Potri.007G107300 Potri.007G108300 Potri.019G124700 Potri.002G145500 Potri.007G111600 Potri.007G111500 Potri.010G075700 Potri.005G137500 Potri.018G051400 Potri.002G202600 Potri.019G124400 Potri.003G113600
GO:0050660	FAD binding	14	Potri.001G463300 Potri.001G462000 Potri.011G161600 Potri.006G198700 Potri.011G158700 Potri.001G462200 Potri.001G098300 Potri.001G461800 Potri.001G459200 Potri.011G158100 Potri.001G459100 Potri.011G155900 Potri.011G158500 Potri.010G168200
GO:0020037	heme binding	30	Potri.016G125000 Potri.001G242600 Potri.006G228200 Potri.004G149000 Potri.001G331100 Potri.007G084700 Potri.006G266200 Potri.019G064200 Potri.001G003100 Potri.004G015300 Potri.007G049900 Potri.005G143800 Potri.008G106400 Potri.005G135300 Potri.009G101700 Potri.005G108900 Potri.006G094700 Potri.001G270900 Potri.019G064600 Potri.009G064900 Potri.011G117600 Potri.016G132700 Potri.013G156500 Potri.003G214900 Potri.001G270800 Potri.006G129900 Potri.007G084200 Potri.015G003500 Potri.004G017700 Potri.T045500
GO:0016787	hydrolase activity	157	Potri.005G233400 Potri.002G243900 Potri.T004600 Potri.005G064300 Potri.015G058400 Potri.009G046600 Potri.001G173700 Potri.003G080400 Potri.010G184600 Potri.012G042200 Potri.003G131700 Potri.013G109600 Potri.008G102600 Potri.T107600 Potri.010G055200 Potri.015G049100 Potri.009G146800 Potri.014G046300 Potri.016G054800 Potri.006G110900 Potri.T004800 Potri.003G106100 Potri.008G190200 Potri.002G094000 Potri.008G108100 Potri.002G226700 Potri.001G171900 Potri.T149700 Potri.010G062500 Potri.009G098100 Potri.001G312800 Potri.009G107200 Potri.001G455300 Potri.001G255100 Potri.015G031300 Potri.017G055800 Potri.008G139100 Potri.001G403900 Potri.013G155900 Potri.004G160100 Potri.004G159000 Potri.004G232900 Potri.018G054800 Potri.008G094200 Potri.012G116000 Potri.008G196800 Potri.010G156300 Potri.003G113600 Potri.006G188400 Potri.019G093800 Potri.011G094400 Potri.001G191000 Potri.014G018900 Potri.001G000800 Potri.015G031400 Potri.017G130200 Potri.016G139700 Potri.018G024200 Potri.014G130500 Potri.013G013200 Potri.003G195500 Potri.010G123500 Potri.010G125800 Potri.011G066800 Potri.002G104600 Potri.008G159100 Potri.006G062200 Potri.016G042500 Potri.007G107300 Potri.007G108300 Potri.006G166700 Potri.009G055900 Potri.018G112000 Potri.001G348200 Potri.007G072300 Potri.013G120200 Potri.001G222900 Potri.001G409900 Potri.018G051400 Potri.005G075300 Potri.001G339900 Potri.015G041300 Potri.007G019600 Potri.017G063500 Potri.014G026500 Potri.003G030700 Potri.017G016800 Potri.002G202500 Potri.015G035000 Potri.008G134000 Potri.002G145500 Potri.001G119300 Potri.001G212800 Potri.001G423700 Potri.006G153300 Potri.010G236800

			Potri.008G120000 Potri.001G233700 Potri.004G208100 Potri.001G325200 Potri.001G324500 Potri.002G083700 Potri.007G010000 Potri.001G342600 Potri.006G207900 Potri.019G064300 Potri.008G073500 Potri.017G050400 Potri.003G161100 Potri.004G019500 Potri.001G311300 Potri.011G152400 Potri.008G086800 Potri.008G114300 Potri.001G061100 Potri.011G043100 Potri.012G036400 Potri.005G167800 Potri.018G132100 Potri.009G153900 Potri.006G188300 Potri.008G064700 Potri.011G024700 Potri.010G141600 Potri.018G121500 Potri.001G227300 Potri.009G087600 Potri.018G015100 Potri.003G126800 Potri.008G203200 Potri.017G102100 Potri.007G089400 Potri.008G047900 Potri.001G252100 Potri.009G160600 Potri.001G041700 Potri.003G034600 Potri.017G133200 Potri.010G188900 Potri.002G054900 Potri.001G416300 Potri.007G133500 Potri.007G021900 Potri.002G213100 Potri.009G006600 Potri.010G220100 Potri.016G057400 Potri.008G086700 Potri.016G116000 Potri.018G132000 Potri.017G084000 Potri.001G240600 Potri.010G152000 Potri.002G202600 Potri.011G089700 Potri.008G079400 Potri.001G191400
GO:0016798	hydrolase activity, acting on glycosyl bonds	36	Potri.010G141600 Potri.018G121500 Potri.006G188400 Potri.009G087600 Potri.019G093800 Potri.011G094400 Potri.017G130200 Potri.003G131700 Potri.007G089400 Potri.018G024200 Potri.001G227300 Potri.015G049100 Potri.001G409900 Potri.016G054800 Potri.002G094000 Potri.010G125800 Potri.008G108100 Potri.008G120000 Potri.001G171900 Potri.007G010000 Potri.008G094200 Potri.006G062200 Potri.018G112000 Potri.001G255100 Potri.009G006600 Potri.004G019500 Potri.016G057400 Potri.001G403900 Potri.011G152400 Potri.001G222900 Potri.005G167800 Potri.010G152000 Potri.009G153900 Potri.006G188300 Potri.015G041300 Potri.008G079400
GO:0005506	iron ion binding	34	Potri.016G125000 Potri.001G463800 Potri.001G242600 Potri.006G228200 Potri.008G106400 Potri.004G149000 Potri.001G331100 Potri.007G084700 Potri.006G266200 Potri.019G064200 Potri.001G003100 Potri.013G035700 Potri.004G015300 Potri.007G049900 Potri.005G143800 Potri.016G124700 Potri.005G135300 Potri.009G101700 Potri.005G108900 Potri.006G094700 Potri.001G270900 Potri.019G064600 Potri.009G064900 Potri.011G117600 Potri.016G132700 Potri.013G156500 Potri.003G214900 Potri.001G270800 Potri.006G129900 Potri.007G084200 Potri.001G098300 Potri.015G003500 Potri.004G017700 Potri.T045500
GO:0016298	lipase activity	7	Potri.001G252100 Potri.T004600 Potri.009G046600 Potri.018G132100 Potri.001G173700 Potri.010G188900 Potri.T004800
GO:0017111	nucleoside-triphosphatase activity	27	Potri.005G064300 Potri.017G016800 Potri.001G000800 Potri.003G126800 Potri.013G109600 Potri.008G047900 Potri.010G055200 Potri.009G160600 Potri.014G046300 Potri.001G416300 Potri.008G159100 Potri.011G024700 Potri.007G021900 Potri.005G075300 Potri.T149700 Potri.010G062500 Potri.014G130500 Potri.001G455300 Potri.001G311300 Potri.001G348200 Potri.008G073500 Potri.004G159000 Potri.018G132000 Potri.011G043100 Potri.017G084000 Potri.001G233700 Potri.007G019600
GO:0016491	oxidoreductase activity	104	Potri.016G125000 Potri.001G242600 Potri.006G094700 Potri.011G071100 Potri.001G463300 Potri.001G461800 Potri.001G459100 Potri.013G053100 Potri.018G063300 Potri.019G045000 Potri.001G113100 Potri.001G451800 Potri.010G168200 Potri.001G176500 Potri.004G067000 Potri.006G062500 Potri.001G015300 Potri.001G270900 Potri.011G117600 Potri.016G132700 Potri.006G198700 Potri.007G143600 Potri.006G069700 Potri.001G098300

			Potri.016G129100 Potri.004G017700 Potri.017G109300 Potri.006G137500 Potri.011G150100 Potri.006G228200 Potri.001G457000 Potri.004G149000 Potri.001G331100 Potri.007G084700 Potri.008G204900 Potri.001G452600 Potri.005G143800 Potri.013G099500 Potri.011G020900 Potri.016G117500 Potri.008G141800 Potri.018G046600 Potri.015G081000 Potri.009G101700 Potri.005G089600 Potri.013G064200 Potri.005G108900 Potri.016G073900 Potri.011G161600 Potri.011G158700 Potri.008G194100 Potri.001G462200 Potri.009G010000 Potri.T045500 Potri.011G047300 Potri.006G083900 Potri.007G040900 Potri.019G064200 Potri.018G131700 Potri.008G161600 Potri.001G003100 Potri.010G193100 Potri.005G136200 Potri.005G043400 Potri.003G219000 Potri.008G157400 Potri.001G459200 Potri.007G106500 Potri.005G135300 Potri.011G158100 Potri.002G127400 Potri.008G149200 Potri.013G156500 Potri.003G214900 Potri.007G084200 Potri.015G135400 Potri.001G219300 Potri.001G391100 Potri.001G463800 Potri.008G106400 Potri.019G064600 Potri.005G245700 Potri.012G143600 Potri.001G394400 Potri.004G200100 Potri.004G015300 Potri.007G126600 Potri.007G049900 Potri.004G075800 Potri.001G015400 Potri.016G112000 Potri.005G079400 Potri.012G128200 Potri.005G229500 Potri.011G155900 Potri.004G199900 Potri.001G462000 Potri.001G090000 Potri.009G064900 Potri.009G025900 Potri.001G270800 Potri.006G129900 Potri.015G003500 Potri.011G158500
GO:0030599	pectinesterase activity	11	Potri.008G102600 Potri.T107600 Potri.013G013200 Potri.001G119300 Potri.007G107300 Potri.007G108300 Potri.018G051400 Potri.002G202600 Potri.002G145500 Potri.002G202500 Potri.003G113600
GO:0004601	peroxidase activity	13	Potri.016G125000 Potri.005G135300 Potri.016G132700 Potri.013G156500 Potri.003G214900 Potri.006G129900 Potri.007G126600 Potri.004G015300 Potri.008G106400 Potri.001G098300 Potri.015G003500 Potri.005G108900 Potri.T045500
GO:0042578	phosphoric ester hydrolase activity	19	Potri.017G063500 Potri.001G325200 Potri.008G196800 Potri.001G252100 Potri.001G324500 Potri.015G058400 Potri.001G061100 Potri.009G146800 Potri.018G054800 Potri.001G191000 Potri.006G110900 Potri.009G046600 Potri.003G034600 Potri.008G190200 Potri.010G188900 Potri.010G184600 Potri.016G139700 Potri.010G156300 Potri.004G232900
GO:0004672	protein kinase activity	84	Potri.009G100400 Potri.004G135500 Potri.001G200000 Potri.006G193000 Potri.003G136100 Potri.016G051600 Potri.001G018700 Potri.011G072300 Potri.006G141500 Potri.005G100900 Potri.019G048800 Potri.T021400 Potri.001G040000 Potri.019G083200 Potri.006G079600 Potri.018G041000 Potri.002G019300 Potri.019G018100 Potri.002G004900 Potri.014G147300 Potri.006G057500 Potri.007G067900 Potri.016G102500 Potri.002G009300 Potri.005G185500 Potri.008G160200 Potri.T089900 Potri.018G091000 Potri.002G036200 Potri.004G095700 Potri.001G393200 Potri.004G193000 Potri.006G166600 Potri.001G218100 Potri.017G152400 Potri.009G075900 Potri.T023800 Potri.012G033200 Potri.001G217700 Potri.012G054700 Potri.011G037000 Potri.015G018000 Potri.019G094200 Potri.T011000 Potri.004G186200 Potri.008G124300 Potri.011G067400 Potri.004G015500 Potri.019G111100 Potri.006G263500 Potri.019G078400 Potri.019G128600 Potri.001G095200 Potri.T021600 Potri.005G135900 Potri.001G412300 Potri.010G087900 Potri.018G138700 Potri.001G117800 Potri.006G066100 Potri.002G017000 Potri.001G085500 Potri.019G099200 Potri.004G027400 Potri.019G109800 Potri.011G068500

			Potri.005G201800 Potri.018G134200 Potri.019G007900 Potri.002G251700 Potri.T064400 Potri.019G004700 Potri.011G128700 Potri.005G051600 Potri.003G185700 Potri.001G014700 Potri.007G140800 Potri.011G028100 Potri.T022600 Potri.017G151400 Potri.017G108000 Potri.011G125200 Potri.016G070500 Potri.011G037100
GO:0004674	protein serine/threonine kinase activity	8	Potri.T021600 Potri.011G128700 Potri.011G037100 Potri.001G412300 Potri.002G036200 Potri.T022600 Potri.T023800 Potri.001G040000
GO:0017171	serine hydrolase activity	9	Potri.013G120200 Potri.014G026500 Potri.001G312800 Potri.006G207900 Potri.001G212800 Potri.014G018900 Potri.009G055900 Potri.010G220100 Potri.007G072300
GO:0003700	transcription factor activity	28	Potri.002G142800 Potri.006G025800 Potri.007G019900 Potri.006G049200 Potri.010G004200 Potri.002G030900 Potri.002G124800 Potri.018G043900 Potri.017G013700 Potri.010G142900 Potri.012G108500 Potri.001G328000 Potri.013G059600 Potri.011G051600 Potri.015G099200 Potri.017G079500 Potri.001G058200 Potri.017G104800 Potri.019G033000 Potri.004G042600 Potri.011G157100 Potri.002G031900 Potri.002G100600 Potri.001G058800 Potri.001G208600 Potri.016G056500 Potri.008G106700 Potri.004G163800
GO:0016757	transferase activity, transferring glycosyl groups	28	Potri.018G123000 Potri.006G063300 Potri.010G040400 Potri.004G059600 Potri.T148700 Potri.004G123600 Potri.004G184200 Potri.010G160200 Potri.002G066600 Potri.008G024800 Potri.018G024500 Potri.014G088300 Potri.016G021600 Potri.001G100700 Potri.013G118700 Potri.006G023600 Potri.006G047200 Potri.009G006600 Potri.010G162100 Potri.006G023700 Potri.010G195600 Potri.002G162300 Potri.007G132400 Potri.001G416800 Potri.016G021500 Potri.008G101000 Potri.016G021100 Potri.005G073800
GO:0016772	transferase activity, transferring phosphorus-containing groups	93	Potri.006G193000 Potri.019G099200 Potri.011G072300 Potri.005G100900 Potri.T021400 Potri.019G083200 Potri.018G041000 Potri.019G018100 Potri.008G102500 Potri.T089900 Potri.018G091000 Potri.019G078400 Potri.004G095700 Potri.007G067900 Potri.001G218100 Potri.019G094200 Potri.T148900 Potri.017G108000 Potri.006G263500 Potri.002G017000 Potri.013G060400 Potri.004G027400 Potri.T021600 Potri.T064400 Potri.011G128700 Potri.005G257800 Potri.011G028100 Potri.005G201800 Potri.006G141500 Potri.006G079600 Potri.001G040000 Potri.006G057500 Potri.007G140800 Potri.008G160200 Potri.001G393200 Potri.009G075900 Potri.005G135900 Potri.T011000 Potri.004G015500 Potri.011G015600 Potri.T022600 Potri.001G117800 Potri.006G066100 Potri.019G109800 Potri.019G048800 Potri.004G135500 Potri.001G200000 Potri.009G100400 Potri.002G019300 Potri.002G004900 Potri.016G051600 Potri.010G087900 Potri.002G036200 Potri.012G033200 Potri.012G054700 Potri.011G037000 Potri.015G018000 Potri.019G111100 Potri.006G166600 Potri.001G412300 Potri.018G138700 Potri.009G084600 Potri.019G128600 Potri.011G068500 Potri.019G007900 Potri.002G251700 Potri.005G185500 Potri.017G152400 Potri.017G151400 Potri.016G070500 Potri.003G136100 Potri.003G152600 Potri.001G018700 Potri.014G147300 Potri.016G102500 Potri.002G009300 Potri.004G193000 Potri.011G037100 Potri.001G217700 Potri.T023800 Potri.006G070500 Potri.004G186200 Potri.008G124300 Potri.011G067400 Potri.001G095200 Potri.001G085500 Potri.018G134200 Potri.019G004700 Potri.005G051600 Potri.003G185700 Potri.001G014700 Potri.001G134000 Potri.011G125200
GO:0005215	transporter activity	44	Potri.006G239700 Potri.006G034000 Potri.001G375200 Potri.014G116000 Potri.002G078100 Potri.001G160400

			Potri.002G005500 Potri.001G318500 Potri.014G136500 Potri.016G111000 Potri.010G026500 Potri.016G055300 Potri.010G055200 Potri.005G256100 Potri.001G185700 Potri.014G046300 Potri.005G102800 Potri.006G268200 Potri.014G132500 Potri.019G056500 Potri.004G032500 Potri.006G158900 Potri.008G159100 Potri.011G024700 Potri.008G170100 Potri.005G188600 Potri.012G043000 Potri.007G044000 Potri.001G374300 Potri.001G094600 Potri.016G053600 Potri.002G092500 Potri.014G130500 Potri.001G010300 Potri.T133800 Potri.008G126500 Potri.001G069800 Potri.016G024900 Potri.010G211300 Potri.011G043100 Potri.012G081800 Potri.017G135400 Potri.006G006800 Potri.015G077600
GO:0004842	ubiquitin-protein ligase activity	5	Potri.010G113900 Potri.006G202700 Potri.016G069500 Potri.016G069400 Potri.007G110600

Supplementary Table S2 (cont.): Details of GO terms enriched by down-regulated DEGs of Aig at **6 hpi** of three infection stages in Figure 4

Process go term	Description	Matched counts	Matched genes
GO:0009309	amine biosynthetic process	6	Potri.010G153500 Potri.004G163300 Potri.012G011700 Potri.009G072900 Potri.015G017500 Potri.001G278400
GO:0005975	carbohydrate metabolic process	15	Potri.007G011200 Potri.018G094800 Potri.018G063500 Potri.017G032300 Potri.015G037000 Potri.012G041100 Potri.001G226100 Potri.008G174100 Potri.T101100 Potri.010G092900 Potri.014G147900 Potri.001G225900 Potri.017G040800 Potri.006G136700 Potri.006G276600
GO:0046394	carboxylic acid biosynthetic process	5	Potri.010G153500 Potri.001G278400 Potri.015G017500 Potri.012G011700 Potri.009G072900
GO:0044262	cellular carbohydrate metabolic process	6	Potri.007G011200 Potri.018G094800 Potri.018G063500 Potri.012G041100 Potri.006G136700 Potri.006G276600
GO:0044237	cellular metabolic process	52	Potri.007G011200 Potri.001G218800 Potri.018G063500 Potri.014G160500 Potri.001G286700 Potri.004G163300 Potri.T059200 Potri.010G001500 Potri.002G057400 Potri.004G047500 Potri.015G002300 Potri.005G087200 Potri.005G195000 Potri.006G276600 Potri.013G100700 Potri.002G182000 Potri.016G090300 Potri.004G203400 Potri.007G111200 Potri.002G167400 Potri.009G072900 Potri.012G005900 Potri.010G232200 Potri.013G079700 Potri.009G130100 Potri.013G059900 Potri.014G147900 Potri.008G159000 Potri.008G009300 Potri.001G278400 Potri.005G257000 Potri.016G038000 Potri.019G086200 Potri.010G153500 Potri.017G032300 Potri.018G094800 Potri.015G017500 Potri.010G105700 Potri.011G025200 Potri.008G135200 Potri.016G126100 Potri.001G397200 Potri.004G138400 Potri.009G146500 Potri.012G041100 Potri.001G249200 Potri.012G011700 Potri.T137400 Potri.006G136700 Potri.006G035400 Potri.017G053800 Potri.017G063900
GO:0044267	cellular protein metabolic	10	Potri.007G111200 Potri.014G160500 Potri.001G286700 Potri.009G146500 Potri.010G232200 Potri.010G001500

	process		Potri.009G130100 Potri.013G059900 Potri.019G086200 Potri.008G009300
GO:0006629	lipid metabolic process	6	Potri.005G218500 Potri.017G032300 Potri.018G063900 Potri.003G081500 Potri.011G025200 Potri.006G276600
GO:0043170	macromolecule metabolic process	34	Potri.001G218800 Potri.014G160500 Potri.001G286700 Potri.010G001500 Potri.004G047500 Potri.015G002300 Potri.005G087200 Potri.005G195000 Potri.006G276600 Potri.002G182000 Potri.016G090300 Potri.004G203400 Potri.007G111200 Potri.002G167400 Potri.012G005900 Potri.010G232200 Potri.013G079700 Potri.009G130100 Potri.008G174100 Potri.013G059900 Potri.019G086200 Potri.008G009300 Potri.008G135200 Potri.016G038000 Potri.T101100 Potri.018G094800 Potri.010G105700 Potri.016G126100 Potri.014G077900 Potri.001G397200 Potri.009G146500 Potri.T137400 Potri.017G040800 Potri.017G053800
GO:0055114	oxidation reduction	23	Potri.009G110500 Potri.012G011700 Potri.001G152600 Potri.010G189800 Potri.014G193800 Potri.001G355100 Potri.006G024300 Potri.001G382400 Potri.002G234000 Potri.005G032400 Potri.005G257000 Potri.017G046200 Potri.001G015500 Potri.009G101700 Potri.015G017500 Potri.011G025200 Potri.014G147700 Potri.014G020400 Potri.014G038700 Potri.001G334700 Potri.005G251600 Potri.014G072000 Potri.017G063900
GO:0006793	phosphorus metabolic process	9	Potri.007G111200 Potri.014G160500 Potri.009G146500 Potri.010G232200 Potri.010G001500 Potri.013G059900 Potri.019G086200 Potri.008G009300 Potri.014G147900
GO:0016310	phosphorylation	7	Potri.007G111200 Potri.009G146500 Potri.010G001500 Potri.013G059900 Potri.019G086200 Potri.008G009300 Potri.014G147900
GO:0005976	polysaccharide metabolic process	5	Potri.T101100 Potri.018G094800 Potri.006G276600 Potri.008G174100 Potri.017G040800
GO:0044238	primary metabolic process	59	Potri.007G011200 Potri.001G218800 Potri.018G063500 Potri.014G160500 Potri.001G286700 Potri.004G163300 Potri.T059200 Potri.001G226100 Potri.002G057400 Potri.004G047500 Potri.015G002300 Potri.005G087200 Potri.005G195000 Potri.006G276600 Potri.013G100700 Potri.002G182000 Potri.003G081500 Potri.004G203400 Potri.007G111200 Potri.002G167400 Potri.009G072900 Potri.012G005900 Potri.010G232200 Potri.013G079700 Potri.009G130100 Potri.008G174100 Potri.013G059900 Potri.014G147900 Potri.001G225900 Potri.008G009300 Potri.001G278400 Potri.005G257000 Potri.016G038000 Potri.010G092900 Potri.019G086200 Potri.010G153500 Potri.017G032300 Potri.018G063900 Potri.016G090300 Potri.T101100 Potri.018G094800 Potri.015G017500 Potri.010G105700 Potri.011G025200 Potri.010G001500 Potri.008G135200 Potri.016G126100 Potri.005G218500 Potri.001G397200 Potri.015G037000 Potri.009G146500 Potri.012G041100 Potri.012G011700 Potri.T137400 Potri.017G040800 Potri.006G136700 Potri.006G035400 Potri.017G053800 Potri.017G063900
GO:0019538	protein metabolic process	10	Potri.007G111200 Potri.014G160500 Potri.001G286700 Potri.009G146500 Potri.010G232200 Potri.010G001500 Potri.009G130100 Potri.013G059900 Potri.019G086200 Potri.008G009300
GO:0006464	protein modification process	8	Potri.007G111200 Potri.014G160500 Potri.009G146500 Potri.010G232200 Potri.010G001500 Potri.013G059900 Potri.019G086200 Potri.008G009300
GO:0080090	regulation of primary	17	Potri.002G182000 Potri.016G090300 Potri.001G397200 Potri.004G203400 Potri.T137400 Potri.012G005900

	metabolic process		Potri.013G079700 Potri.002G167400 Potri.001G218800 Potri.015G002300 Potri.010G105700 Potri.005G087200 Potri.004G047500 Potri.005G195000 Potri.008G135200 Potri.016G038000 Potri.016G126100
GO:0023052	signaling	7	Potri.014G030100 Potri.007G111200 Potri.012G005900 Potri.015G002300 Potri.019G086200 Potri.013G059900 Potri.016G038000
GO:0055085	transmembrane transport	8	Potri.013G115600 Potri.003G197400 Potri.019G085800 Potri.018G085200 Potri.016G103500 Potri.006G170900 Potri.010G116700 Potri.011G042100
Function go term	Description	Matched counts	Matched genes
GO:0030554	adenyl nucleotide binding	16	Potri.010G153500 Potri.007G011200 Potri.007G111200 Potri.001G286700 Potri.009G146500 Potri.017G031800 Potri.010G001500 Potri.012G011700 Potri.017G145300 Potri.001G152600 Potri.013G059900 Potri.014G147900 Potri.008G009300 Potri.015G017500 Potri.008G069100 Potri.019G086200
GO:0005524	ATP binding	13	Potri.010G153500 Potri.007G011200 Potri.007G111200 Potri.001G286700 Potri.009G146500 Potri.017G031800 Potri.010G001500 Potri.017G145300 Potri.013G059900 Potri.014G147900 Potri.008G009300 Potri.008G069100 Potri.019G086200
GO:0020037	heme binding	7	Potri.001G334700 Potri.005G251600 Potri.009G101700 Potri.010G189800 Potri.014G072000 Potri.014G020400 Potri.014G038700
GO:0016787	hydrolase activity	22	Potri.017G031800 Potri.014G160500 Potri.002G082400 Potri.001G226100 Potri.019G079000 Potri.010G109400 Potri.003G081500 Potri.006G035400 Potri.010G232200 Potri.008G174100 Potri.001G225900 Potri.010G107300 Potri.018G063900 Potri.T101100 Potri.002G024100 Potri.017G113700 Potri.014G129400 Potri.001G466200 Potri.005G218500 Potri.018G094800 Potri.017G040800 Potri.008G180500
GO:0016798	hydrolase activity, acting on glycosyl bonds	6	Potri.018G094800 Potri.001G226100 Potri.008G174100 Potri.T101100 Potri.001G225900 Potri.017G040800
GO:0005506	iron ion binding	7	Potri.001G334700 Potri.005G251600 Potri.009G101700 Potri.010G189800 Potri.014G072000 Potri.014G020400 Potri.014G038700
GO:0016491	oxidoreductase activity	31	Potri.011G122400 Potri.004G235600 Potri.017G046200 Potri.005G032400 Potri.001G152600 Potri.010G189800 Potri.014G193800 Potri.001G355100 Potri.006G024300 Potri.001G382400 Potri.002G234000 Potri.004G235500 Potri.002G057400 Potri.005G257000 Potri.012G011700 Potri.009G110500 Potri.002G208400 Potri.001G416500 Potri.001G015500 Potri.009G101700 Potri.015G017500 Potri.011G025200 Potri.014G147700 Potri.014G020400 Potri.014G038700 Potri.015G037000 Potri.001G334700 Potri.005G251600 Potri.014G072000 Potri.014G134200 Potri.017G063900
GO:0004672	protein kinase activity	6	Potri.007G111200 Potri.009G146500 Potri.010G001500 Potri.013G059900 Potri.019G086200 Potri.008G009300
GO:0003700	transcription factor activity	8	Potri.004G047500 Potri.004G203400 Potri.T137400 Potri.002G167400 Potri.001G397200 Potri.005G087200 Potri.005G195000 Potri.016G126100

GO:0016757	transferase activity, transferring glycosyl groups	9	Potri.006G055600 Potri.018G094800 Potri.017G032300 Potri.007G141900 Potri.008G189400 Potri.016G016200 Potri.016G016500 Potri.004G069600 Potri.009G044600
GO:0016772	transferase activity, transferring phosphorus-containing groups	10	Potri.007G111200 Potri.009G146500 Potri.012G041100 Potri.010G001500 Potri.013G059900 Potri.019G086200 Potri.008G159000 Potri.008G009300 Potri.017G053800 Potri.014G147900
GO:0005215	transporter activity	11	Potri.002G249100 Potri.013G115600 Potri.003G197400 Potri.019G085800 Potri.018G010000 Potri.016G103500 Potri.006G170900 Potri.010G116700 Potri.011G042100 Potri.018G085200 Potri.016G098200

Supplementary Table S2 (cont.): Details of GO terms enriched by down-regulated DEGs of Aig at 3**6 hpi** of three infection stages in Figure 4

Process go term	Description	Matched counts	Matched genes
GO:0005975	carbohydrate metabolic process	16	Potri.003G038500 Potri.001G027400 Potri.017G032300 Potri.004G182000 Potri.018G152200 Potri.008G144500 Potri.017G040800 Potri.015G032500 Potri.017G057900 Potri.T101100 Potri.004G150400 Potri.006G276600 Potri.018G094800 Potri.003G030900 Potri.019G077600 Potri.011G154300
GO:0046394	carboxylic acid biosynthetic process	5	Potri.009G072900 Potri.001G278400 Potri.T029700 Potri.012G011700 Potri.010G011300
GO:0044262	cellular carbohydrate metabolic process	6	Potri.018G094800 Potri.008G144500 Potri.015G032500 Potri.003G030900 Potri.004G150400 Potri.006G276600
GO:0044237	cellular metabolic process	93	Potri.015G056900 Potri.001G286700 Potri.008G144500 Potri.014G111400 Potri.011G146500 Potri.012G005900 Potri.013G133100 Potri.013G079700 Potri.009G130100 Potri.001G108100 Potri.019G034800 Potri.001G278400 Potri.004G038000 Potri.006G226800 Potri.014G106000 Potri.009G169900 Potri.008G114200 Potri.002G081800 Potri.002G186600 Potri.016G126100 Potri.009G015700 Potri.013G031100 Potri.009G146500 Potri.013G144600 Potri.012G011700 Potri.003G030900 Potri.006G201900 Potri.T084500 Potri.007G008000 Potri.001G218800 Potri.005G060500 Potri.005G098200 Potri.018G003100 Potri.006G276600 Potri.002G046200 Potri.004G211700 Potri.002G161600 Potri.004G150400 Potri.016G068200 Potri.010G011900 Potri.013G090800 Potri.005G239300 Potri.017G032300 Potri.015G032500 Potri.012G088200 Potri.006G058800 Potri.010G254700 Potri.004G060400 Potri.006G035400 Potri.011G033400 Potri.010G011300 Potri.001G409300 Potri.011G031600 Potri.018G053600 Potri.005G214800 Potri.014G094200 Potri.T029700 Potri.009G072900 Potri.010G232200 Potri.016G038000 Potri.010G242600 Potri.005G119300 Potri.008G029300 Potri.018G094800 Potri.016G003400 Potri.009G052900 Potri.001G133200 Potri.001G249200 Potri.019G128100 Potri.009G146700 Potri.002G113600 Potri.006G221000

			Potri.003G157900 Potri.016G140100 Potri.001G113800 Potri.015G093200 Potri.016G090300 Potri.003G196900 Potri.016G102500 Potri.013G100700 Potri.010G001500 Potri.013G101000 Potri.008G135200 Potri.004G209700 Potri.T146400 Potri.005G228100 Potri.006G273000 Potri.002G179800 Potri.010G053500 Potri.T008700 Potri.004G138400 Potri.009G141600 Potri.005G203200
GO:0044267	cellular protein metabolic process	26	Potri.011G033400 Potri.001G286700 Potri.001G409300 Potri.002G046200 Potri.016G102500 Potri.002G161600 Potri.010G232200 Potri.010G001500 Potri.009G130100 Potri.013G090800 Potri.008G029300 Potri.004G209700 Potri.T146400 Potri.013G133100 Potri.016G003400 Potri.011G146500 Potri.009G015700 Potri.T008700 Potri.009G146500 Potri.013G144600 Potri.016G140100 Potri.019G128100 Potri.T084500 Potri.006G273000 Potri.009G146700 Potri.005G228100
GO:0006732	coenzyme metabolic process	5	Potri.011G031600 Potri.T029700 Potri.010G011300 Potri.008G114200 Potri.009G169900
GO:0006855	multidrug transport	6	Potri.012G050500 Potri.013G115600 Potri.012G051900 Potri.015G147600 Potri.003G116700 Potri.010G116700
GO:0006629	lipid metabolic process	8	Potri.T029700 Potri.017G032300 Potri.010G011300 Potri.018G063900 Potri.004G150400 Potri.010G011900 Potri.002G228300 Potri.006G276600
GO:0043170	macromolecule metabolic process	71	Potri.015G056900 Potri.004G182000 Potri.001G286700 Potri.001G409300 Potri.008G144500 Potri.005G060500 Potri.018G053600 Potri.005G098200 Potri.001G330800 Potri.002G228300 Potri.002G256300 Potri.006G276600 Potri.009G031900 Potri.006G141700 Potri.002G046200 Potri.016G090300 Potri.003G196900 Potri.004G211700 Potri.002G161600 Potri.012G005900 Potri.013G133100 Potri.013G079700 Potri.009G130100 Potri.001G108100 Potri.014G094200 Potri.001G218800 Potri.002G179800 Potri.013G090800 Potri.016G140100 Potri.008G135200 Potri.016G038000 Potri.009G052900 Potri.005G119300 Potri.019G128100 Potri.004G038000 Potri.008G029300 Potri.004G209700 Potri.006G226800 Potri.T146400 Potri.005G214800 Potri.010G232200 Potri.014G106000 Potri.010G001500 Potri.012G088200 Potri.018G094800 Potri.T101100 Potri.001G356900 Potri.005G228100 Potri.006G273000 Potri.011G033400 Potri.016G102500 Potri.002G186600 Potri.011G146500 Potri.016G126100 Potri.018G106800 Potri.006G058800 Potri.009G015700 Potri.010G053500 Potri.T008700 Potri.009G146500 Potri.004G060400 Potri.013G144600 Potri.009G141600 Potri.014G111400 Potri.016G068200 Potri.006G201900 Potri.017G040800 Potri.T084500 Potri.005G203200 Potri.009G146700 Potri.016G003400
GO:0055114	oxidation reduction	56	Potri.014G037400 Potri.016G031700 Potri.006G221000 Potri.003G126100 Potri.008G144500 Potri.017G046200 Potri.004G138700 Potri.010G088800 Potri.013G029900 Potri.001G152600 Potri.009G143500 Potri.010G059400 Potri.005G251600 Potri.009G022800 Potri.001G382400 Potri.001G113800 Potri.002G121300 Potri.010G059500 Potri.002G025300 Potri.006G024300 Potri.008G178000 Potri.011G100600 Potri.013G083600 Potri.010G011900 Potri.011G134900 Potri.019G034800 Potri.007G096200 Potri.011G158100 Potri.010G242600 Potri.012G011700 Potri.005G060500 Potri.007G026500 Potri.007G016400 Potri.001G015500 Potri.009G169900 Potri.009G153600 Potri.009G101700 Potri.001G083900 Potri.002G013400 Potri.002G081800 Potri.018G075000 Potri.012G089900 Potri.014G020400 Potri.003G192300 Potri.002G121200 Potri.013G031100 Potri.002G189900 Potri.004G150400

			Potri.001G464800 Potri.001G334700 Potri.014G021500 Potri.T156400 Potri.014G037900 Potri.014G072000 Potri.016G118000 Potri.015G134600
GO:0006793	phosphorus metabolic process	22	Potri.011G033400 Potri.004G209700 Potri.001G409300 Potri.018G003100 Potri.016G102500 Potri.002G161600 Potri.010G232200 Potri.010G001500 Potri.013G090800 Potri.008G029300 Potri.T146400 Potri.013G133100 Potri.011G146500 Potri.T008700 Potri.009G146500 Potri.013G144600 Potri.016G140100 Potri.003G030900 Potri.019G128100 Potri.T084500 Potri.006G273000 Potri.009G146700
GO:0016310	phosphorylation	19	Potri.004G209700 Potri.011G033400 Potri.T146400 Potri.002G161600 Potri.T008700 Potri.009G146500 Potri.013G144600 Potri.013G133100 Potri.010G001500 Potri.016G140100 Potri.001G409300 Potri.019G128100 Potri.013G090800 Potri.006G273000 Potri.T084500 Potri.016G102500 Potri.018G003100 Potri.009G146700 Potri.011G146500
GO:0005976	polysaccharide metabolic process	5	Potri.T101100 Potri.018G094800 Potri.006G276600 Potri.004G182000 Potri.017G040800
GO:0044238	primary metabolic process	99	Potri.015G056900 Potri.004G182000 Potri.001G286700 Potri.008G144500 Potri.014G111400 Potri.002G228300 Potri.011G154300 Potri.009G031900 Potri.011G146500 Potri.012G005900 Potri.013G133100 Potri.013G079700 Potri.009G130100 Potri.001G108100 Potri.019G034800 Potri.001G278400 Potri.004G038000 Potri.006G226800 Potri.018G063900 Potri.014G106000 Potri.001G027400 Potri.009G169900 Potri.002G186600 Potri.016G126100 Potri.009G015700 Potri.009G146500 Potri.013G144600 Potri.012G011700 Potri.003G030900 Potri.006G201900 Potri.T084500 Potri.007G008000 Potri.001G218800 Potri.005G060500 Potri.005G098200 Potri.006G276600 Potri.002G046200 Potri.004G211700 Potri.002G161600 Potri.004G150400 Potri.016G068200 Potri.010G011900 Potri.013G090800 Potri.017G032300 Potri.019G077600 Potri.015G032500 Potri.012G088200 Potri.001G356900 Potri.006G058800 Potri.010G254700 Potri.004G060400 Potri.001G330800 Potri.006G035400 Potri.003G038500 Potri.011G033400 Potri.010G011300 Potri.001G409300 Potri.018G053600 Potri.005G214800 Potri.014G094200 Potri.T029700 Potri.009G072900 Potri.010G232200 Potri.017G057900 Potri.002G256300 Potri.016G038000 Potri.010G242600 Potri.005G119300 Potri.008G029300 Potri.T101100 Potri.018G094800 Potri.016G003400 Potri.009G052900 Potri.019G128100 Potri.017G040800 Potri.009G146700 Potri.002G113600 Potri.018G152200 Potri.003G157900 Potri.016G140100 Potri.006G141700 Potri.015G093200 Potri.016G090300 Potri.003G196900 Potri.016G102500 Potri.013G100700 Potri.010G001500 Potri.013G101000 Potri.008G135200 Potri.004G209700 Potri.T146400 Potri.005G228100 Potri.006G273000 Potri.002G179800 Potri.018G106800 Potri.010G053500 Potri.T008700 Potri.009G141600 Potri.005G203200
GO:0019538	protein metabolic process	34	Potri.015G056900 Potri.001G286700 Potri.001G409300 Potri.011G033400 Potri.016G140100 Potri.002G228300 Potri.009G031900 Potri.006G141700 Potri.002G046200 Potri.016G102500 Potri.002G161600 Potri.010G232200 Potri.010G001500 Potri.009G130100 Potri.002G256300 Potri.013G090800 Potri.018G106800 Potri.008G029300 Potri.004G209700 Potri.T146400 Potri.013G133100 Potri.001G356900 Potri.005G228100 Potri.011G146500 Potri.009G015700 Potri.T008700 Potri.009G146500 Potri.013G144600 Potri.001G330800 Potri.019G128100

			Potri.T084500 Potri.006G273000 Potri.009G146700 Potri.016G003400
GO:0006464	protein modification process	22	Potri.011G033400 Potri.008G029300 Potri.001G409300 Potri.002G046200 Potri.016G102500 Potri.002G161600 Potri.010G232200 Potri.010G001500 Potri.013G090800 Potri.004G209700 Potri.T146400 Potri.013G133100 Potri.005G228100 Potri.011G146500 Potri.T008700 Potri.009G146500 Potri.013G144600 Potri.016G140100 Potri.019G128100 Potri.T084500 Potri.006G273000 Potri.009G146700
GO:0080090	regulation of primary metabolic process	30	Potri.015G056900 Potri.016G068200 Potri.018G053600 Potri.005G098200 Potri.014G094200 Potri.016G090300 Potri.004G211700 Potri.012G005900 Potri.013G079700 Potri.001G108100 Potri.001G218800 Potri.016G126100 Potri.008G135200 Potri.016G038000 Potri.009G052900 Potri.005G119300 Potri.004G038000 Potri.004G209700 Potri.006G226800 Potri.014G106000 Potri.014G111400 Potri.002G186600 Potri.002G179800 Potri.006G058800 Potri.010G053500 Potri.004G060400 Potri.009G141600 Potri.005G214800 Potri.006G201900 Potri.005G203200
GO:0023052	signaling	11	Potri.014G030100 Potri.006G069300 Potri.016G102500 Potri.006G098200 Potri.001G409300 Potri.012G005900 Potri.014G106000 Potri.011G033400 Potri.019G128100 Potri.016G038000 Potri.002G179800
GO:0055085	transmembrane transport	15	Potri.001G249800 Potri.003G116700 Potri.012G050500 Potri.003G034700 Potri.013G115600 Potri.009G043800 Potri.019G085800 Potri.001G145200 Potri.012G051900 Potri.015G147600 Potri.010G116700 Potri.011G042100 Potri.008G151100 Potri.018G085200 Potri.001G152300
**Function go term**	**Description**	**Matched counts**	**Matched genes**
GO:0022804	active transmembrane transporter activity	8	Potri.013G009400 Potri.012G050500 Potri.003G034700 Potri.013G115600 Potri.012G051900 Potri.015G147600 Potri.010G116700 Potri.003G116700
GO:0030554	adenyl nucleotide binding	38	Potri.006G221000 Potri.015G056900 Potri.001G286700 Potri.001G409300 Potri.005G060500 Potri.008G058500 Potri.001G152600 Potri.011G033400 Potri.018G003100 Potri.015G093200 Potri.010G206600 Potri.002G161600 Potri.003G034700 Potri.002G056400 Potri.013G133100 Potri.010G001500 Potri.013G090800 Potri.011G158100 Potri.004G209700 Potri.T146400 Potri.005G213300 Potri.012G088200 Potri.017G145300 Potri.016G003400 Potri.010G205700 Potri.016G102500 Potri.011G146500 Potri.T008700 Potri.009G146500 Potri.013G144600 Potri.012G011700 Potri.016G140100 Potri.009G143500 Potri.019G128100 Potri.T084500 Potri.006G273000 Potri.009G146700 Potri.001G464800
GO:0016209	antioxidant activity	6	Potri.003G126100 Potri.005G251600 Potri.010G059400 Potri.007G096200 Potri.013G083600 Potri.010G059500
GO:0005524	ATP binding	31	Potri.015G056900 Potri.001G286700 Potri.001G409300 Potri.008G058500 Potri.011G033400 Potri.018G003100 Potri.015G093200 Potri.010G206600 Potri.002G161600 Potri.003G034700 Potri.002G056400 Potri.013G133100 Potri.010G001500 Potri.013G090800 Potri.004G209700 Potri.T146400 Potri.005G213300 Potri.012G088200 Potri.017G145300 Potri.016G003400 Potri.010G205700 Potri.016G102500 Potri.011G146500 Potri.T008700 Potri.009G146500 Potri.013G144600 Potri.016G140100 Potri.019G128100 Potri.T084500 Potri.006G273000 Potri.009G146700

GO:0050662	coenzyme binding	9	Potri.006G221000 Potri.001G464800 Potri.005G060500 Potri.012G011700 Potri.009G169900 Potri.001G152600 Potri.009G143500 Potri.011G158100 Potri.001G133200
GO:0030234	enzyme regulator activity	8	Potri.019G010900 Potri.006G069300 Potri.019G006900 Potri.004G000400 Potri.010G109400 Potri.014G044100 Potri.001G032900 Potri.007G111700
GO:0050660	FAD binding	7	Potri.006G221000 Potri.001G464800 Potri.005G060500 Potri.012G011700 Potri.001G152600 Potri.009G143500 Potri.011G158100
GO:0020037	heme binding	21	Potri.014G037400 Potri.005G251600 Potri.011G100600 Potri.002G121200 Potri.012G089900 Potri.T156400 Potri.014G020400 Potri.013G083600 Potri.016G031700 Potri.001G334700 Potri.014G021500 Potri.014G072000 Potri.009G101700 Potri.001G083900 Potri.014G037900 Potri.007G026500 Potri.007G096200 Potri.002G121300 Potri.003G192300 Potri.007G016400 Potri.002G025300
GO:0016787	hydrolase activity	34	Potri.003G038500 Potri.015G056900 Potri.004G182000 Potri.018G152200 Potri.003G157900 Potri.010G109400 Potri.002G228300 Potri.011G154300 Potri.009G031900 Potri.006G141700 Potri.014G073100 Potri.003G034700 Potri.010G232200 Potri.017G057900 Potri.002G256300 Potri.003G112600 Potri.010G107300 Potri.008G029300 Potri.018G063900 Potri.T099600 Potri.001G027400 Potri.012G088200 Potri.018G094800 Potri.T101100 Potri.001G356900 Potri.017G113700 Potri.001G466200 Potri.018G106800 Potri.013G009400 Potri.019G062600 Potri.001G330800 Potri.014G044100 Potri.017G040800 Potri.006G035400
GO:0016798	hydrolase activity, acting on glycosyl bonds	10	Potri.003G038500 Potri.018G094800 Potri.004G182000 Potri.018G152200 Potri.001G027400 Potri.017G057900 Potri.T101100 Potri.017G040800 Potri.003G112600 Potri.011G154300
GO:0005506	iron ion binding	22	Potri.014G037400 Potri.008G144500 Potri.014G021500 Potri.002G121300 Potri.002G025300 Potri.011G100600 Potri.013G083600 Potri.016G031700 Potri.007G026500 Potri.007G016400 Potri.007G096200 Potri.009G101700 Potri.001G083900 Potri.014G020400 Potri.003G192300 Potri.002G121200 Potri.012G089900 Potri.001G334700 Potri.005G251600 Potri.T156400 Potri.014G037900 Potri.014G072000
GO:0016491	oxidoreductase activity	61	Potri.014G037400 Potri.016G031700 Potri.006G221000 Potri.003G126100 Potri.004G235600 Potri.008G144500 Potri.017G046200 Potri.004G138700 Potri.010G088800 Potri.013G029900 Potri.001G152600 Potri.009G143500 Potri.010G059400 Potri.005G251600 Potri.002G156300 Potri.009G022800 Potri.003G141800 Potri.002G121300 Potri.010G059500 Potri.002G025300 Potri.006G024300 Potri.008G178000 Potri.011G100600 Potri.013G083600 Potri.007G026500 Potri.004G235500 Potri.010G011900 Potri.011G134900 Potri.019G034800 Potri.007G096200 Potri.011G158100 Potri.010G242600 Potri.012G011700 Potri.005G060500 Potri.002G208400 Potri.007G016400 Potri.001G416500 Potri.001G015500 Potri.009G169900 Potri.009G153600 Potri.009G101700 Potri.001G083900 Potri.002G013400 Potri.002G081800 Potri.018G075000 Potri.014G020400 Potri.003G192300 Potri.005G064000 Potri.002G121200 Potri.012G089900 Potri.002G189900 Potri.001G464800 Potri.001G334700 Potri.014G021500 Potri.T156400 Potri.014G037900 Potri.014G072000 Potri.014G134200 Potri.001G382400 Potri.016G118000 Potri.015G134600
GO:0004672	protein kinase activity	18	Potri.004G209700 Potri.011G033400 Potri.T146400 Potri.002G161600 Potri.T008700 Potri.009G146500

			Potri.013G144600 Potri.013G133100 Potri.010G001500 Potri.016G140100 Potri.001G409300 Potri.019G128100 Potri.013G090800 Potri.006G273000 Potri.T084500 Potri.016G102500 Potri.009G146700 Potri.011G146500
GO:0003700	transcription factor activity	14	Potri.006G058800 Potri.005G119300 Potri.006G226800 Potri.004G060400 Potri.016G068200 Potri.001G108100 Potri.018G053600 Potri.005G214800 Potri.006G201900 Potri.005G203200 Potri.002G186600 Potri.009G052900 Potri.014G094200 Potri.016G126100
GO:0016757	transferase activity, transferring glycosyl groups	17	Potri.006G055600 Potri.017G051900 Potri.017G052300 Potri.002G162300 Potri.010G232700 Potri.014G041800 Potri.013G005800 Potri.010G042000 Potri.014G096100 Potri.010G150400 Potri.018G094800 Potri.016G016200 Potri.002G168600 Potri.016G057000 Potri.016G016500 Potri.004G069600 Potri.017G032300
GO:0016769	transferase activity, transferring nitrogenous groups	5	Potri.002G113600 Potri.007G008000 Potri.001G162800 Potri.016G132200 Potri.008G187400
GO:0016772	transferase activity, transferring phosphorus-containing groups	25	Potri.011G033400 Potri.001G409300 Potri.018G003100 Potri.015G093200 Potri.003G196900 Potri.016G102500 Potri.002G161600 Potri.002G056400 Potri.013G133100 Potri.010G001500 Potri.013G090800 Potri.004G209700 Potri.T146400 Potri.019G077600 Potri.015G032500 Potri.011G146500 Potri.T008700 Potri.009G146500 Potri.013G144600 Potri.016G140100 Potri.003G030900 Potri.019G128100 Potri.T084500 Potri.006G273000 Potri.009G146700
GO:0005215	transporter activity	20	Potri.013G009400 Potri.001G145200 Potri.010G034300 Potri.012G050500 Potri.002G249100 Potri.003G034700 Potri.010G094400 Potri.013G115600 Potri.019G085800 Potri.018G085200 Potri.T180400 Potri.018G010000 Potri.015G147600 Potri.016G098200 Potri.010G116700 Potri.011G042100 Potri.008G151100 Potri.011G072100 Potri.012G051900 Potri.003G116700

Supplementary Table S2 (cont.): Details of GO terms enriched by down-regulated DEGs of Aig at **96 hpi** of three infection stages in Figure 4

Process go term	Description	Matched counts	Matched genes
GO:0005975	carbohydrate metabolic process	16	Potri.019G010800 Potri.007G011200 Potri.009G041800 Potri.017G032300 Potri.001G226100 Potri.010G229300 Potri.T101100 Potri.001G254800 Potri.001G095800 Potri.019G049700 Potri.001G225900 Potri.017G040800 Potri.010G092900 Potri.001G129600 Potri.015G126900 Potri.014G147900
GO:0044237	cellular metabolic process	76	Potri.009G123400 Potri.007G011200 Potri.001G218800 Potri.012G043700 Potri.014G160500 Potri.001G286700 Potri.018G138200 Potri.T059200 Potri.019G086200 Potri.001G092400 Potri.008G029300 Potri.019G028100 Potri.003G157900 Potri.004G047500 Potri.015G002300 Potri.013G051300 Potri.005G087200 Potri.005G195000 Potri.015G147500 Potri.008G177100 Potri.013G100700 Potri.010G153500 Potri.019G005300 Potri.014G047500 Potri.004G203400 Potri.007G111200 Potri.012G005900 Potri.013G133100 Potri.T156100

			Potri.009G036700 Potri.004G163300 Potri.013G059900 Potri.019G049700 Potri.008G159000 Potri.008G009300 Potri.012G134100 Potri.005G257000 Potri.016G038000 Potri.014G045100 Potri.005G196600 Potri.011G052700 Potri.001G095800 Potri.014G147900 Potri.005G144100 Potri.014G066500 Potri.T146400 Potri.017G032300 Potri.010G232200 Potri.010G169600 Potri.002G137700 Potri.016G084400 Potri.019G131300 Potri.005G228100 Potri.011G025200 Potri.005G180700 Potri.010G130000 Potri.008G135200 Potri.015G126900 Potri.006G052400 Potri.001G397200 Potri.011G074600 Potri.003G139300 Potri.019G011400 Potri.T137400 Potri.015G030700 Potri.007G142100 Potri.001G249200 Potri.016G140100 Potri.006G202100 Potri.004G046100 Potri.006G228400 Potri.013G143200 Potri.010G176500 Potri.017G063900 Potri.002G176900 Potri.010G105700
GO:0044267	cellular protein metabolic process	26	Potri.013G133100 Potri.014G160500 Potri.001G286700 Potri.018G138200 Potri.013G051300 Potri.008G177100 Potri.019G005300 Potri.014G047500 Potri.007G111200 Potri.010G232200 Potri.013G059900 Potri.019G086200 Potri.008G009300 Potri.005G196600 Potri.008G029300 Potri.005G144100 Potri.T146400 Potri.002G137700 Potri.016G084400 Potri.005G228100 Potri.006G052400 Potri.019G011400 Potri.015G030700 Potri.007G142100 Potri.016G140100 Potri.006G228400
GO:0006629	lipid metabolic process	6	Potri.T051900 Potri.017G032300 Potri.018G063900 Potri.005G068700 Potri.002G128000 Potri.011G025200
GO:0043170	macromolecule metabolic process	60	Potri.009G123400 Potri.001G218800 Potri.016G084400 Potri.012G043700 Potri.014G160500 Potri.001G286700 Potri.018G138200 Potri.001G092400 Potri.004G047500 Potri.015G002300 Potri.013G051300 Potri.005G087200 Potri.005G195000 Potri.015G147500 Potri.008G177100 Potri.019G005300 Potri.014G047500 Potri.004G203400 Potri.007G111200 Potri.012G005900 Potri.013G133100 Potri.006G202100 Potri.009G036700 Potri.002G256300 Potri.013G059900 Potri.019G049700 Potri.001G095800 Potri.008G009300 Potri.012G134100 Potri.008G135200 Potri.016G038000 Potri.014G045100 Potri.005G196600 Potri.019G086200 Potri.008G029300 Potri.005G144100 Potri.014G066500 Potri.T146400 Potri.010G232200 Potri.010G169600 Potri.002G137700 Potri.T101100 Potri.019G131300 Potri.005G228100 Potri.010G130000 Potri.014G077900 Potri.006G052400 Potri.001G397200 Potri.005G180700 Potri.003G139300 Potri.019G011400 Potri.T137400 Potri.015G030700 Potri.007G142100 Potri.016G140100 Potri.004G046100 Potri.006G228400 Potri.017G040800 Potri.002G176900 Potri.010G105700
GO:0055114	oxidation reduction	15	Potri.006G024300 Potri.011G074600 Potri.001G095800 Potri.016G044200 Potri.001G334700 Potri.013G143200 Potri.011G052700 Potri.011G025200 Potri.001G464800 Potri.005G257000 Potri.002G234000 Potri.014G020400 Potri.017G063900 Potri.014G038700 Potri.T110600
GO:0006793	phosphorus metabolic process	20	Potri.019G005300 Potri.014G047500 Potri.010G232200 Potri.T146400 Potri.007G111200 Potri.014G160500 Potri.008G029300 Potri.015G030700 Potri.007G142100 Potri.013G133100 Potri.005G144100 Potri.016G140100 Potri.002G137700 Potri.006G228400 Potri.013G059900 Potri.014G147900 Potri.013G051300 Potri.008G009300 Potri.019G011400 Potri.019G086200
GO:0016310	phosphorylation	17	Potri.019G005300 Potri.014G047500 Potri.T146400 Potri.007G111200 Potri.019G011400 Potri.015G030700 Potri.007G142100 Potri.013G133100 Potri.005G144100 Potri.016G140100 Potri.002G137700 Potri.006G228400

			Potri.013G059900 Potri.014G147900 Potri.013G051300 Potri.008G009300 Potri.019G086200
GO:0044238	primary metabolic process	85	Potri.009G123400 Potri.007G011200 Potri.001G218800 Potri.012G043700 Potri.014G160500 Potri.001G286700 Potri.018G138200 Potri.T059200 Potri.001G092400 Potri.005G144100 Potri.003G157900 Potri.004G047500 Potri.015G002300 Potri.013G051300 Potri.005G087200 Potri.011G052700 Potri.015G147500 Potri.008G177100 Potri.013G100700 Potri.010G153500 Potri.019G005300 Potri.014G047500 Potri.004G203400 Potri.007G111200 Potri.012G005900 Potri.013G133100 Potri.006G202100 Potri.009G036700 Potri.002G256300 Potri.005G195000 Potri.005G068700 Potri.013G059900 Potri.019G049700 Potri.001G225900 Potri.008G009300 Potri.001G226100 Potri.012G134100 Potri.005G257000 Potri.016G038000 Potri.014G045100 Potri.005G196600 Potri.014G147900 Potri.008G029300 Potri.T051900 Potri.009G041800 Potri.T146400 Potri.017G032300 Potri.018G063900 Potri.010G232200 Potri.010G169600 Potri.010G229300 Potri.002G137700 Potri.T101100 Potri.019G131300 Potri.001G254800 Potri.001G129600 Potri.005G228100 Potri.011G025200 Potri.010G176500 Potri.019G086200 Potri.010G130000 Potri.008G135200 Potri.015G126900 Potri.019G010800 Potri.016G084400 Potri.006G052400 Potri.001G397200 Potri.005G180700 Potri.003G139300 Potri.019G011400 Potri.T137400 Potri.015G030700 Potri.007G142100 Potri.016G140100 Potri.004G046100 Potri.006G228400 Potri.004G163300 Potri.001G095800 Potri.002G128000 Potri.017G040800 Potri.010G092900 Potri.014G066500 Potri.017G063900 Potri.002G176900 Potri.010G105700
GO:0019538	protein metabolic process	27	Potri.013G133100 Potri.014G160500 Potri.001G286700 Potri.018G138200 Potri.013G051300 Potri.008G177100 Potri.019G005300 Potri.014G047500 Potri.007G111200 Potri.010G232200 Potri.002G256300 Potri.013G059900 Potri.019G086200 Potri.008G009300 Potri.005G196600 Potri.008G029300 Potri.005G144100 Potri.T146400 Potri.002G137700 Potri.016G084400 Potri.005G228100 Potri.006G052400 Potri.019G011400 Potri.015G030700 Potri.007G142100 Potri.016G140100 Potri.006G228400
GO:0006464	protein modification process	21	Potri.019G005300 Potri.014G047500 Potri.010G232200 Potri.T146400 Potri.007G111200 Potri.014G160500 Potri.008G029300 Potri.015G030700 Potri.007G142100 Potri.013G133100 Potri.005G144100 Potri.016G140100 Potri.002G137700 Potri.006G228400 Potri.013G059900 Potri.005G228100 Potri.013G051300 Potri.008G009300 Potri.019G011400 Potri.008G177100 Potri.019G086200
GO:0080090	regulation of primary metabolic process	24	Potri.009G123400 Potri.001G218800 Potri.001G092400 Potri.004G047500 Potri.015G002300 Potri.012G043700 Potri.005G087200 Potri.005G195000 Potri.004G203400 Potri.012G005900 Potri.006G202100 Potri.012G134100 Potri.008G135200 Potri.016G038000 Potri.014G045100 Potri.014G066500 Potri.019G131300 Potri.010G105700 Potri.010G130000 Potri.001G397200 Potri.003G139300 Potri.T137400 Potri.004G046100 Potri.002G176900
GO:0023052	signaling	10	Potri.014G030100 Potri.007G111200 Potri.019G011400 Potri.012G005900 Potri.012G048200 Potri.013G059900 Potri.019G086200 Potri.015G002300 Potri.016G038000 Potri.010G080900
GO:0055085	transmembrane transport	6	Potri.019G063500 Potri.013G115600 Potri.016G103500 Potri.010G116700 Potri.010G116900 Potri.008G049600

Function go term	Description	Matched counts	Matched genes
GO:0022804	active transmembrane transporter activity	5	Potri.013G115600 Potri.010G116900 Potri.003G059700 Potri.010G116700 Potri.019G063500
GO:0030554	adenyl nucleotide binding	36	Potri.007G011200 Potri.013G133100 Potri.001G286700 Potri.018G138200 Potri.T016200 Potri.013G051300 Potri.019G011400 Potri.008G054600 Potri.T013300 Potri.007G127900 Potri.010G153500 Potri.019G005300 Potri.001G426200 Potri.007G111200 Potri.017G031800 Potri.017G014900 Potri.013G059900 Potri.014G147900 Potri.008G009300 Potri.019G086200 Potri.005G144100 Potri.T146400 Potri.004G214300 Potri.014G047500 Potri.002G137700 Potri.017G145300 Potri.001G254800 Potri.010G205700 Potri.T066200 Potri.015G030700 Potri.007G142100 Potri.010G188200 Potri.016G140100 Potri.006G228400 Potri.001G464800 Potri.008G069100
GO:0005524	ATP binding	35	Potri.007G011200 Potri.013G133100 Potri.001G286700 Potri.018G138200 Potri.T016200 Potri.013G051300 Potri.019G011400 Potri.008G054600 Potri.T013300 Potri.007G127900 Potri.010G153500 Potri.019G005300 Potri.001G426200 Potri.007G111200 Potri.017G031800 Potri.017G014900 Potri.013G059900 Potri.014G147900 Potri.008G009300 Potri.019G086200 Potri.005G144100 Potri.T146400 Potri.004G214300 Potri.014G047500 Potri.002G137700 Potri.017G145300 Potri.001G254800 Potri.010G205700 Potri.T066200 Potri.015G030700 Potri.007G142100 Potri.010G188200 Potri.016G140100 Potri.006G228400 Potri.008G069100
GO:0004091	carboxylesterase activity	5	Potri.014G129400 Potri.005G068700 Potri.010G109400 Potri.006G135100 Potri.T156100
GO:0016831	carboxy-lyase activity	7	Potri.007G011200 Potri.004G163300 Potri.T059200 Potri.T006000 Potri.012G062600 Potri.T063100 Potri.T005800
GO:0030234	enzyme regulator activity	5	Potri.014G129400 Potri.010G109400 Potri.006G135100 Potri.001G149600 Potri.014G036000
GO:0016787	hydrolase activity	30	Potri.017G031800 Potri.014G160500 Potri.002G082400 Potri.001G226100 Potri.003G157900 Potri.019G079000 Potri.010G109400 Potri.007G127900 Potri.009G104300 Potri.010G232200 Potri.T156100 Potri.002G256300 Potri.005G174800 Potri.005G068700 Potri.001G225900 Potri.008G029300 Potri.004G214300 Potri.018G063900 Potri.010G169600 Potri.010G229300 Potri.T101100 Potri.010G176500 Potri.014G129400 Potri.019G010800 Potri.005G180700 Potri.T122700 Potri.017G040800 Potri.008G180500 Potri.006G135100 Potri.001G129600
GO:0016798	hydrolase activity, acting on glycosyl bonds	9	Potri.019G010800 Potri.005G180700 Potri.010G169600 Potri.001G226100 Potri.010G229300 Potri.T101100 Potri.001G225900 Potri.017G040800 Potri.001G129600
GO:0016491	oxidoreductase activity	16	Potri.006G024300 Potri.011G074600 Potri.001G095800 Potri.002G208400 Potri.016G044200 Potri.001G334700 Potri.011G052700 Potri.011G025200 Potri.014G134200 Potri.001G464800 Potri.005G257000 Potri.002G234000 Potri.014G020400 Potri.017G063900 Potri.014G038700 Potri.T110600
GO:0004672	protein kinase activity	16	Potri.019G005300 Potri.014G047500 Potri.T146400 Potri.007G111200 Potri.019G011400 Potri.015G030700 Potri.007G142100 Potri.013G133100 Potri.005G144100 Potri.016G140100 Potri.002G137700 Potri.006G228400

			Potri.013G059900 Potri.019G086200 Potri.013G051300 Potri.008G009300
GO:0003700	transcription factor activity	13	Potri.009G123400 Potri.001G397200 Potri.004G203400 Potri.003G139300 Potri.T137400 Potri.001G092400 Potri.005G195000 Potri.019G131300 Potri.004G047500 Potri.012G043700 Potri.005G087200 Potri.012G134100 Potri.014G045100
GO:0016757	transferase activity, transferring glycosyl groups	9	Potri.017G052300 Potri.017G032300 Potri.010G137000 Potri.007G141900 Potri.017G051900 Potri.009G041800 Potri.019G049700 Potri.016G057000 Potri.014G096100
GO:0016772	transferase activity, transferring phosphorus-containing groups	19	Potri.019G005300 Potri.014G047500 Potri.008G159000 Potri.T146400 Potri.007G111200 Potri.019G011400 Potri.015G030700 Potri.007G142100 Potri.013G133100 Potri.005G144100 Potri.016G140100 Potri.002G137700 Potri.006G228400 Potri.001G254800 Potri.013G059900 Potri.014G147900 Potri.013G051300 Potri.008G009300 Potri.019G086200
GO:0005215	transporter activity	12	Potri.T000200 Potri.002G249100 Potri.019G063500 Potri.013G115600 Potri.003G059700 Potri.016G103500 Potri.010G116700 Potri.008G061100 Potri.016G098200 Potri.010G116900 Potri.008G049600 Potri.010G001400

Supplementary Table S3: Details of GO terms enriched by up-regulated DEGs of Leu at **6hpi** of three infection stages in Figure 4

Process go term	Description	Matched counts	Matched genes
GO:0005975	carbohydrate metabolic process	24	Potri.018G152200 Potri.004G117800 Potri.010G145800 Potri.006G048100 Potri.002G173600 Potri.009G080600 Potri.013G125000 Potri.014G111800 Potri.018G112000 Potri.009G006600 Potri.016G057400 Potri.008G186000 Potri.019G010800 Potri.002G093300 Potri.001G222900 Potri.015G024200 Potri.002G060500 Potri.012G033900 Potri.002G089800 Potri.005G167800 Potri.008G079500 Potri.006G188300 Potri.004G110200 Potri.019G093700
GO:0007154	cell communication	10	Potri.004G027400 Potri.011G037300 Potri.010G015400 Potri.011G128900 Potri.011G125000 Potri.011G039100 Potri.011G129000 Potri.013G059900 Potri.T023600 Potri.001G414200
GO:0008037	cell recognition	10	Potri.004G027400 Potri.011G037300 Potri.010G015400 Potri.011G128900 Potri.011G125000 Potri.011G039100 Potri.011G129000 Potri.013G059900 Potri.T023600 Potri.001G414200
GO:0071554	cell wall organization or biogenesis	9	Potri.002G145500 Potri.004G117800 Potri.014G111800 Potri.015G127700 Potri.002G202600 Potri.003G191200 Potri.005G128200 Potri.013G125000 Potri.019G093700
GO:0044262	cellular carbohydrate metabolic process	9	Potri.002G093300 Potri.002G060500 Potri.004G117800 Potri.002G089800 Potri.002G173600 Potri.010G145800 Potri.009G006600 Potri.009G080600 Potri.008G186000
GO:0044237	cellular metabolic process	102	Potri.010G103100 Potri.006G193000 Potri.019G078300 Potri.001G046100 Potri.010G015400 Potri.010G188600 Potri.003G025600 Potri.009G154100 Potri.T003400 Potri.011G128900 Potri.006G049200

			Potri.002G070500 Potri.009G169900 Potri.004G027400 Potri.008G089000 Potri.001G080900 Potri.001G042400 Potri.004G058500 Potri.001G216100 Potri.011G129000 Potri.001G352400 Potri.009G118300 Potri.014G141400 Potri.004G061900 Potri.010G100500 Potri.006G148800 Potri.001G457000 Potri.006G105300 Potri.001G045500 Potri.001G154200 Potri.011G037300 Potri.005G128200 Potri.005G105000 Potri.002G129100 Potri.015G074500 Potri.002G173600 Potri.008G137700 Potri.013G059900 Potri.005G073300 Potri.009G080600 Potri.007G135100 Potri.002G114500 Potri.006G103200 Potri.010G043900 Potri.006G200600 Potri.011G125000 Potri.016G134900 Potri.001G168000 Potri.016G128300 Potri.006G202600 Potri.002G057200 Potri.014G195200 Potri.001G092900 Potri.015G103800 Potri.006G202700 Potri.004G209300 Potri.003G080600 Potri.004G117800 Potri.004G024600 Potri.014G101100 Potri.008G059900 Potri.012G067600 Potri.001G020600 Potri.006G186600 Potri.012G054700 Potri.017G135000 Potri.014G090300 Potri.010G166200 Potri.011G061700 Potri.001G140700 Potri.018G138700 Potri.002G240800 Potri.003G025800 Potri.008G186000 Potri.002G093300 Potri.005G205400 Potri.012G031700 Potri.015G061600 Potri.004G007500 Potri.011G149700 Potri.010G145800 Potri.002G089800 Potri.T023600 Potri.003G187000 Potri.004G083900 Potri.002G060500 Potri.009G016100 Potri.017G079500 Potri.004G208500 Potri.015G064100 Potri.013G103300 Potri.004G024400 Potri.016G066700 Potri.009G006600 Potri.T084700 Potri.006G263600 Potri.011G039100 Potri.004G025800 Potri.003G138600 Potri.016G069400 Potri.014G156400 Potri.001G414200
GO:0044267	cellular protein metabolic process	54	Potri.010G103100 Potri.004G061900 Potri.004G209300 Potri.006G193000 Potri.011G039100 Potri.019G078300 Potri.T023600 Potri.004G024600 Potri.014G101100 Potri.005G128200 Potri.008G059900 Potri.006G103200 Potri.011G037300 Potri.006G202600 Potri.010G015400 Potri.012G067600 Potri.002G129100 Potri.009G016100 Potri.010G188600 Potri.008G137700 Potri.013G059900 Potri.003G025600 Potri.012G054700 Potri.004G083900 Potri.017G135000 Potri.009G154100 Potri.002G070500 Potri.T003400 Potri.011G128900 Potri.010G043900 Potri.006G200600 Potri.011G125000 Potri.016G134900 Potri.013G103300 Potri.004G024400 Potri.014G195200 Potri.016G066700 Potri.008G186000 Potri.018G138700 Potri.003G025800 Potri.015G061600 Potri.004G027400 Potri.001G168000 Potri.T084700 Potri.004G058500 Potri.001G216100 Potri.002G089800 Potri.011G129000 Potri.004G025800 Potri.006G202700 Potri.016G069400 Potri.001G042400 Potri.014G156400 Potri.001G414200
GO:0006732	coenzyme metabolic process	5	Potri.001G457000 Potri.009G080600 Potri.002G240800 Potri.004G208500 Potri.009G169900
GO:0006629	lipid metabolic process	11	Potri.001G046100 Potri.001G252100 Potri.T095600 Potri.014G033200 Potri.002G128000 Potri.001G045500 Potri.001G140700 Potri.009G118300 Potri.005G177700 Potri.014G033900 Potri.007G040300
GO:0043170	macromolecule metabolic process	100	Potri.010G103100 Potri.006G193000 Potri.019G064800 Potri.019G078300 Potri.006G202600 Potri.010G015400 Potri.010G188600 Potri.003G025600 Potri.009G154100 Potri.T003400 Potri.011G128900 Potri.006G049200 Potri.002G070500 Potri.014G111800 Potri.004G027400 Potri.008G089000 Potri.001G080900 Potri.001G042400 Potri.004G058500 Potri.001G216100 Potri.011G129000 Potri.001G352400 Potri.002G060500

			Potri.004G061900 Potri.006G148800 Potri.006G105300 Potri.001G154200 Potri.011G037300 Potri.005G128200 Potri.005G105000 Potri.002G129100 Potri.015G074500 Potri.002G173600 Potri.008G137700 Potri.013G059900 Potri.006G200600 Potri.009G080600 Potri.007G135100 Potri.013G125000 Potri.002G114500 Potri.006G103200 Potri.010G043900 Potri.005G249200 Potri.005G205400 Potri.011G125000 Potri.016G134900 Potri.001G168000 Potri.016G128300 Potri.002G057200 Potri.014G195200 Potri.001G092900 Potri.006G202700 Potri.004G209300 Potri.003G080600 Potri.006G068900 Potri.013G070300 Potri.004G117800 Potri.004G024600 Potri.014G141400 Potri.014G101100 Potri.008G059900 Potri.012G067600 Potri.006G186600 Potri.012G054700 Potri.017G135000 Potri.014G090300 Potri.010G166200 Potri.011G061700 Potri.018G138700 Potri.003G025800 Potri.008G186000 Potri.002G093300 Potri.005G204600 Potri.015G103900 Potri.T084700 Potri.012G031700 Potri.015G061600 Potri.004G007500 Potri.011G149700 Potri.002G089800 Potri.008G203200 Potri.T023600 Potri.004G083900 Potri.009G016100 Potri.002G054900 Potri.017G079500 Potri.019G073800 Potri.015G064100 Potri.013G103300 Potri.004G024400 Potri.016G066700 Potri.009G006600 Potri.006G263600 Potri.011G039100 Potri.004G025800 Potri.003G138600 Potri.019G093700 Potri.016G069400 Potri.014G156400 Potri.001G414200
GO:0051704	multi-organism process	10	Potri.004G027400 Potri.011G037300 Potri.010G015400 Potri.011G128900 Potri.011G125000 Potri.011G039100 Potri.011G129000 Potri.013G059900 Potri.T023600 Potri.001G414200
GO:0055114	oxidation reduction	45	Potri.016G125000 Potri.T160000 Potri.007G115500 Potri.001G457000 Potri.018G146100 Potri.001G458900 Potri.T160100 Potri.011G162900 Potri.009G065000 Potri.001G045500 Potri.T079500 Potri.001G011500 Potri.001G463300 Potri.001G046100 Potri.018G065600 Potri.001G458700 Potri.007G126600 Potri.001G270400 Potri.008G087400 Potri.013G045000 Potri.003G159800 Potri.009G080600 Potri.002G114500 Potri.010G231500 Potri.001G422500 Potri.004G208500 Potri.005G144000 Potri.009G169900 Potri.001G140700 Potri.005G064400 Potri.003G103900 Potri.006G151600 Potri.001G167700 Potri.008G205200 Potri.011G155900 Potri.016G132800 Potri.019G057900 Potri.005G143900 Potri.014G143200 Potri.T163100 Potri.001G270800 Potri.011G158500 Potri.009G118300 Potri.017G064100 Potri.001G270300
GO:0006793	phosphorus metabolic process	41	Potri.004G061900 Potri.004G209300 Potri.006G193000 Potri.019G078300 Potri.004G024600 Potri.T023600 Potri.005G128200 Potri.008G059900 Potri.006G103200 Potri.011G037300 Potri.010G015400 Potri.012G067600 Potri.002G129100 Potri.010G188600 Potri.013G059900 Potri.003G025600 Potri.012G054700 Potri.009G154100 Potri.T003400 Potri.011G128900 Potri.010G043900 Potri.006G200600 Potri.011G125000 Potri.016G134900 Potri.013G103300 Potri.004G024400 Potri.014G195200 Potri.016G066700 Potri.018G138700 Potri.003G025800 Potri.015G061600 Potri.004G027400 Potri.001G168000 Potri.T084700 Potri.004G058500 Potri.011G039100 Potri.011G129000 Potri.004G025800 Potri.001G042400 Potri.014G156400 Potri.001G414200
GO:0016310	phosphorylation	41	Potri.004G061900 Potri.004G209300 Potri.006G193000 Potri.019G078300 Potri.004G024600 Potri.T023600 Potri.005G128200 Potri.008G059900 Potri.006G103200 Potri.011G037300 Potri.010G015400 Potri.012G067600 Potri.002G129100 Potri.010G188600 Potri.013G059900 Potri.003G025600 Potri.012G054700 Potri.009G154100 Potri.T003400 Potri.011G128900 Potri.010G043900 Potri.006G200600 Potri.011G125000 Potri.016G134900

			Potri.013G103300 Potri.004G024400 Potri.014G195200 Potri.016G066700 Potri.018G138700 Potri.003G025800 Potri.015G061600 Potri.004G027400 Potri.001G168000 Potri.T084700 Potri.004G058500 Potri.011G039100 Potri.011G129000 Potri.004G025800 Potri.001G042400 Potri.014G156400 Potri.001G414200
GO:0005976	polysaccharide metabolic process	7	Potri.002G060500 Potri.004G117800 Potri.002G173600 Potri.014G111800 Potri.009G006600 Potri.013G125000 Potri.019G093700
GO:0044238	primary metabolic process	133	Potri.010G103100 Potri.T095600 Potri.006G193000 Potri.019G064800 Potri.019G078300 Potri.001G046100 Potri.010G015400 Potri.010G188600 Potri.007G040300 Potri.014G033900 Potri.009G154100 Potri.T003400 Potri.011G128900 Potri.006G049200 Potri.002G070500 Potri.009G169900 Potri.004G027400 Potri.008G089000 Potri.001G080900 Potri.001G042400 Potri.004G058500 Potri.001G216100 Potri.011G129000 Potri.001G352400 Potri.009G118300 Potri.008G079500 Potri.004G110200 Potri.014G141400 Potri.004G061900 Potri.010G100500 Potri.006G148800 Potri.001G457000 Potri.006G105300 Potri.001G045500 Potri.001G154200 Potri.011G037300 Potri.005G128200 Potri.005G105000 Potri.002G129100 Potri.015G074500 Potri.008G186000 Potri.002G173600 Potri.008G137700 Potri.013G059900 Potri.005G073300 Potri.009G080600 Potri.007G135100 Potri.013G125000 Potri.002G114500 Potri.006G103200 Potri.010G043900 Potri.005G249200 Potri.006G200600 Potri.011G125000 Potri.016G134900 Potri.001G168000 Potri.016G128300 Potri.006G202600 Potri.018G112000 Potri.002G057200 Potri.001G222900 Potri.002G089800 Potri.015G103800 Potri.006G048100 Potri.006G202700 Potri.004G209300 Potri.003G080600 Potri.006G068900 Potri.013G070300 Potri.004G117800 Potri.004G024600 Potri.014G101100 Potri.008G059900 Potri.012G067600 Potri.001G020600 Potri.006G186600 Potri.012G054700 Potri.017G135000 Potri.003G025600 Potri.014G090300 Potri.010G166200 Potri.011G061700 Potri.001G140700 Potri.018G138700 Potri.002G240800 Potri.003G025800 Potri.015G061600 Potri.002G093300 Potri.005G204600 Potri.015G103900 Potri.005G205400 Potri.012G031700 Potri.005G167800 Potri.006G188300 Potri.004G007500 Potri.011G149700 Potri.008G203200 Potri.018G152200 Potri.010G145800 Potri.001G092900 Potri.005G177700 Potri.T023600 Potri.003G187000 Potri.004G083900 Potri.001G252100 Potri.002G060500 Potri.014G033200 Potri.002G128000 Potri.002G054900 Potri.009G016100 Potri.017G079500 Potri.019G073800 Potri.004G208500 Potri.015G064100 Potri.013G103300 Potri.004G024400 Potri.014G195200 Potri.016G066700 Potri.009G006600 Potri.014G111800 Potri.016G057400 Potri.T084700 Potri.006G263600 Potri.019G010800 Potri.015G024200 Potri.012G033900 Potri.011G039100 Potri.004G025800 Potri.003G138600 Potri.019G093700 Potri.016G069400 Potri.014G156400 Potri.001G414200
GO:0019538	protein metabolic process	63	Potri.010G103100 Potri.004G061900 Potri.004G209300 Potri.006G193000 Potri.011G039100 Potri.006G068900 Potri.013G070300 Potri.019G078300 Potri.T023600 Potri.004G024600 Potri.008G203200 Potri.014G101100 Potri.005G128200 Potri.008G059900 Potri.006G103200 Potri.011G037300 Potri.006G202600 Potri.010G015400 Potri.012G067600 Potri.002G129100 Potri.009G016100 Potri.010G188600 Potri.008G137700 Potri.013G059900 Potri.003G025600 Potri.002G054900 Potri.012G054700 Potri.004G083900 Potri.017G135000 Potri.009G154100 Potri.002G070500 Potri.T003400 Potri.011G128900 Potri.010G043900 Potri.005G249200

			Potri.006G200600 Potri.011G125000 Potri.016G134900 Potri.013G103300 Potri.019G064800 Potri.004G024400 Potri.014G195200 Potri.016G066700 Potri.008G186000 Potri.018G138700 Potri.019G073800 Potri.003G025800 Potri.015G061600 Potri.004G027400 Potri.005G204600 Potri.015G103900 Potri.001G168000 Potri.T084700 Potri.004G058500 Potri.001G216100 Potri.002G089800 Potri.011G129000 Potri.004G025800 Potri.006G202700 Potri.016G069400 Potri.001G042400 Potri.014G156400 Potri.001G414200
GO:0006464	protein modification process	54	Potri.010G103100 Potri.004G061900 Potri.004G209300 Potri.006G193000 Potri.011G039100 Potri.019G078300 Potri.T023600 Potri.004G024600 Potri.014G101100 Potri.005G128200 Potri.008G059900 Potri.006G103200 Potri.011G037300 Potri.006G202600 Potri.010G015400 Potri.012G067600 Potri.002G129100 Potri.009G016100 Potri.010G188600 Potri.008G137700 Potri.013G059900 Potri.003G025600 Potri.012G054700 Potri.004G083900 Potri.017G135000 Potri.009G154100 Potri.002G070500 Potri.T003400 Potri.011G128900 Potri.010G043900 Potri.006G200600 Potri.011G125000 Potri.016G134900 Potri.013G103300 Potri.004G024400 Potri.014G195200 Potri.016G066700 Potri.008G186000 Potri.018G138700 Potri.003G025800 Potri.015G061600 Potri.004G027400 Potri.001G168000 Potri.T084700 Potri.004G058500 Potri.001G216100 Potri.002G089800 Potri.011G129000 Potri.004G025800 Potri.006G202700 Potri.016G069400 Potri.001G042400 Potri.014G156400 Potri.001G414200
GO:0016567	protein ubiquitination	11	Potri.010G103100 Potri.006G202600 Potri.009G016100 Potri.002G070500 Potri.001G216100 Potri.008G137700 Potri.006G202700 Potri.014G101100 Potri.017G135000 Potri.016G069400 Potri.004G083900
GO:0080090	regulation of primary metabolic process	26	Potri.011G149700 Potri.006G148800 Potri.003G080600 Potri.006G105300 Potri.006G049200 Potri.001G154200 Potri.015G074500 Potri.006G186600 Potri.007G135100 Potri.017G079500 Potri.015G064100 Potri.014G090300 Potri.005G205400 Potri.010G166200 Potri.016G128300 Potri.011G061700 Potri.002G057200 Potri.006G263600 Potri.008G089000 Potri.001G080900 Potri.014G141400 Potri.012G031700 Potri.001G092900 Potri.001G352400 Potri.003G138600 Potri.004G007500
GO:0006979	response to oxidative stress	11	Potri.016G125000 Potri.T160000 Potri.T163100 Potri.014G143200 Potri.001G458900 Potri.T160100 Potri.007G126600 Potri.001G458700 Potri.017G064100 Potri.016G132800 Potri.001G011500
GO:0023052	signaling	13	Potri.004G027400 Potri.011G037300 Potri.010G015400 Potri.011G128900 Potri.011G125000 Potri.011G039100 Potri.011G129000 Potri.007G143300 Potri.013G059900 Potri.T023600 Potri.001G252100 Potri.005G215700 Potri.001G414200
GO:0055085	transmembrane transport	13	Potri.018G035500 Potri.002G078100 Potri.005G223500 Potri.015G081300 Potri.005G167300 Potri.014G097900 Potri.004G019900 Potri.006G266000 Potri.010G119100 Potri.002G187400 Potri.001G258600 Potri.012G131300 Potri.009G113600

Function go term	Description	Matched counts	Matched genes
GO:0022804	active transmembrane transporter activity	5	Potri.001G020600 Potri.010G119100 Potri.002G187400 Potri.012G131300 Potri.002G078100

GO:0030554	adenyl nucleotide binding	57	Potri.004G061900 Potri.004G209300 Potri.006G193000 Potri.019G078300 Potri.011G162900 Potri.004G024600 Potri.T023600 Potri.005G128200 Potri.008G059900 Potri.006G103200 Potri.001G463300 Potri.010G015400 Potri.012G069800 Potri.002G129100 Potri.006G200600 Potri.010G188600 Potri.008G087400 Potri.013G059900 Potri.003G025600 Potri.003G159800 Potri.012G054700 Potri.002G114500 Potri.009G154100 Potri.011G037300 Potri.012G069700 Potri.002G187400 Potri.T003400 Potri.011G128900 Potri.010G043900 Potri.005G249200 Potri.001G189500 Potri.011G125000 Potri.016G134900 Potri.013G103300 Potri.004G024400 Potri.014G195200 Potri.016G066700 Potri.006G115000 Potri.018G138700 Potri.006G169000 Potri.003G025800 Potri.011G155900 Potri.015G061600 Potri.012G067600 Potri.004G027400 Potri.013G030600 Potri.001G168000 Potri.T084700 Potri.004G058500 Potri.011G039100 Potri.011G129000 Potri.004G025800 Potri.007G143300 Potri.011G158500 Potri.001G042400 Potri.014G156400 Potri.001G414200
GO:0016209	antioxidant activity	12	Potri.016G125000 Potri.T160000 Potri.T163100 Potri.014G143200 Potri.001G458900 Potri.T160100 Potri.007G126600 Potri.001G458700 Potri.003G159800 Potri.017G064100 Potri.016G132800 Potri.001G011500
GO:0070001	aspartic-type peptidase activity	6	Potri.013G070300 Potri.005G204600 Potri.006G068900 Potri.019G064800 Potri.008G203200 Potri.002G054900
GO:0005524	ATP binding	50	Potri.004G061900 Potri.004G209300 Potri.006G193000 Potri.019G078300 Potri.004G024600 Potri.T023600 Potri.005G128200 Potri.008G059900 Potri.006G103200 Potri.011G037300 Potri.010G015400 Potri.012G069800 Potri.002G129100 Potri.010G188600 Potri.013G059900 Potri.003G025600 Potri.006G200600 Potri.012G054700 Potri.009G154100 Potri.012G069700 Potri.002G187400 Potri.T003400 Potri.011G128900 Potri.010G043900 Potri.005G249200 Potri.001G189500 Potri.011G125000 Potri.016G134900 Potri.013G103300 Potri.004G024400 Potri.014G195200 Potri.016G066700 Potri.006G115000 Potri.018G138700 Potri.006G169000 Potri.003G025800 Potri.015G061600 Potri.012G067600 Potri.004G027400 Potri.013G030600 Potri.001G168000 Potri.T084700 Potri.004G058500 Potri.011G039100 Potri.011G129000 Potri.004G025800 Potri.007G143300 Potri.001G042400 Potri.014G156400 Potri.001G414200
GO:0050662	coenzyme binding	16	Potri.001G463300 Potri.001G046100 Potri.004G208500 Potri.001G457000 Potri.018G065600 Potri.011G158500 Potri.008G087400 Potri.011G162900 Potri.001G045500 Potri.001G140700 Potri.009G118300 Potri.003G159800 Potri.009G080600 Potri.009G169900 Potri.011G155900 Potri.002G114500
GO:0030234	enzyme regulator activity	8	Potri.002G145500 Potri.011G110100 Potri.015G128700 Potri.019G080600 Potri.011G110400 Potri.015G127700 Potri.019G124700 Potri.002G202600
GO:0050660	FAD binding	7	Potri.001G463300 Potri.011G162900 Potri.008G087400 Potri.011G158500 Potri.003G159800 Potri.011G155900 Potri.002G114500
GO:0008066	glutamate receptor activity	5	Potri.001G374600 Potri.006G268200 Potri.011G062600 Potri.004G052400 Potri.004G052600
GO:0020037	heme binding	23	Potri.016G125000 Potri.T160000 Potri.007G115500 Potri.018G146100 Potri.001G458900 Potri.T160100 Potri.009G065000 Potri.001G011500 Potri.001G270400 Potri.001G458700 Potri.002G242500 Potri.001G422500 Potri.005G144000 Potri.005G064400 Potri.008G205200 Potri.016G132800 Potri.019G057900 Potri.005G143900

			Potri.014G143200 Potri.T163100 Potri.001G270800 Potri.017G064100 Potri.001G270300
GO:0016787	hydrolase activity	44	Potri.001G032400 Potri.005G074100 Potri.012G033900 Potri.008G203200 Potri.018G152200 Potri.006G068900 Potri.019G064800 Potri.002G187400 Potri.005G177700 Potri.005G167800 Potri.018G024200 Potri.005G105000 Potri.012G069800 Potri.015G128700 Potri.001G020600 Potri.002G171200 Potri.002G173600 Potri.015G127700 Potri.002G054900 Potri.013G125000 Potri.012G069700 Potri.013G070300 Potri.005G249200 Potri.001G189500 Potri.019G073800 Potri.014G111800 Potri.006G115000 Potri.018G112000 Potri.009G006600 Potri.002G145500 Potri.016G057400 Potri.019G010800 Potri.005G204600 Potri.015G103900 Potri.001G222900 Potri.015G024200 Potri.002G060500 Potri.001G252100 Potri.006G048100 Potri.008G079500 Potri.002G202600 Potri.006G188300 Potri.004G110200 Potri.019G093700
GO:0016798	hydrolase activity, acting on glycosyl bonds	19	Potri.019G010800 Potri.005G167800 Potri.018G024200 Potri.008G079500 Potri.001G222900 Potri.018G152200 Potri.015G024200 Potri.002G060500 Potri.012G033900 Potri.002G173600 Potri.014G111800 Potri.006G048100 Potri.018G112000 Potri.009G006600 Potri.006G188300 Potri.004G110200 Potri.016G057400 Potri.013G125000 Potri.019G093700
GO:0005506	iron ion binding	24	Potri.016G125000 Potri.T160000 Potri.007G115500 Potri.018G146100 Potri.001G458900 Potri.T160100 Potri.009G065000 Potri.001G011500 Potri.001G270400 Potri.001G458700 Potri.003G159800 Potri.002G242500 Potri.001G422500 Potri.005G144000 Potri.005G064400 Potri.008G205200 Potri.016G132800 Potri.019G057900 Potri.005G143900 Potri.014G143200 Potri.T163100 Potri.001G270800 Potri.017G064100 Potri.001G270300
GO:0017111	nucleoside-triphosphatase activity	8	Potri.012G069700 Potri.002G187400 Potri.005G105000 Potri.012G069800 Potri.001G020600 Potri.001G189500 Potri.006G115000 Potri.002G171200
GO:0016491	oxidoreductase activity	47	Potri.016G125000 Potri.T160000 Potri.007G115500 Potri.001G457000 Potri.T163100 Potri.018G146100 Potri.001G458900 Potri.T160100 Potri.011G162900 Potri.009G065000 Potri.001G045500 Potri.019G110000 Potri.T079500 Potri.001G011500 Potri.001G463300 Potri.001G046100 Potri.001G270400 Potri.018G065600 Potri.001G458700 Potri.007G126600 Potri.010G097600 Potri.008G087400 Potri.013G045000 Potri.003G159800 Potri.009G080600 Potri.002G114500 Potri.010G231500 Potri.001G422500 Potri.004G208500 Potri.005G144000 Potri.009G169900 Potri.001G140700 Potri.005G064400 Potri.006G151600 Potri.001G167700 Potri.008G205200 Potri.011G155900 Potri.016G132800 Potri.019G057900 Potri.005G143900 Potri.014G143200 Potri.008G144800 Potri.001G270800 Potri.011G158500 Potri.009G118300 Potri.017G064100 Potri.001G270300
GO:0004601	peroxidase activity	12	Potri.016G125000 Potri.T160000 Potri.T163100 Potri.014G143200 Potri.001G458900 Potri.T160100 Potri.007G126600 Potri.001G458700 Potri.003G159800 Potri.017G064100 Potri.016G132800 Potri.001G011500
GO:0004672	protein kinase activity	41	Potri.004G061900 Potri.004G209300 Potri.006G193000 Potri.019G078300 Potri.004G024600 Potri.T023600 Potri.005G128200 Potri.008G059900 Potri.006G103200 Potri.011G037300 Potri.010G015400 Potri.012G067600 Potri.002G129100 Potri.010G188600 Potri.013G059900 Potri.003G025600 Potri.012G054700 Potri.009G154100 Potri.T003400 Potri.011G128900 Potri.010G043900 Potri.006G200600 Potri.011G125000 Potri.016G134900 Potri.013G103300 Potri.004G024400 Potri.014G195200 Potri.016G066700 Potri.018G138700 Potri.003G025800

			Potri.015G061600 Potri.004G027400 Potri.001G168000 Potri.T084700 Potri.004G058500 Potri.011G039100 Potri.011G129000 Potri.004G025800 Potri.001G042400 Potri.014G156400 Potri.001G414200
GO:0004674	protein serine/threonine kinase activity	9	Potri.011G037300 Potri.010G015400 Potri.011G128900 Potri.011G125000 Potri.011G039100 Potri.011G129000 Potri.T023600 Potri.001G414200 Potri.009G154100
GO:0003700	transcription factor activity	17	Potri.017G079500 Potri.015G064100 Potri.003G080600 Potri.001G154200 Potri.014G141400 Potri.012G031700 Potri.006G049200 Potri.001G352400 Potri.001G092900 Potri.014G090300 Potri.016G128300 Potri.011G061700 Potri.003G138600 Potri.007G135100 Potri.006G105300 Potri.006G263600 Potri.004G007500
GO:0016757	transferase activity, transferring glycosyl groups	11	Potri.001G158000 Potri.006G120600 Potri.002G060500 Potri.004G117800 Potri.002G089800 Potri.016G097400 Potri.T178700 Potri.009G006600 Potri.003G191200 Potri.008G186000 Potri.015G045500
GO:0016772	transferase activity, transferring phosphorus-containing groups	41	Potri.004G061900 Potri.004G209300 Potri.006G193000 Potri.019G078300 Potri.004G024600 Potri.T023600 Potri.005G128200 Potri.008G059900 Potri.006G103200 Potri.011G037300 Potri.010G015400 Potri.012G067600 Potri.002G129100 Potri.010G188600 Potri.013G059900 Potri.003G025600 Potri.012G054700 Potri.009G154100 Potri.T003400 Potri.011G128900 Potri.010G043900 Potri.006G200600 Potri.011G125000 Potri.016G134900 Potri.013G103300 Potri.004G024400 Potri.014G195200 Potri.016G066700 Potri.018G138700 Potri.003G025800 Potri.015G061600 Potri.004G027400 Potri.001G168000 Potri.T084700 Potri.004G058500 Potri.011G039100 Potri.011G129000 Potri.004G025800 Potri.001G042400 Potri.014G156400 Potri.001G414200
GO:0005215	transporter activity	17	Potri.001G374600 Potri.008G170100 Potri.018G035500 Potri.002G078100 Potri.005G223500 Potri.005G167300 Potri.014G097900 Potri.004G052400 Potri.002G187400 Potri.001G020600 Potri.010G119100 Potri.006G268200 Potri.001G258600 Potri.011G062600 Potri.012G131300 Potri.009G113600 Potri.004G052600
GO:0004842	ubiquitin-protein ligase activity	11	Potri.010G103100 Potri.006G202600 Potri.009G016100 Potri.002G070500 Potri.001G216100 Potri.008G137700 Potri.006G202700 Potri.014G101100 Potri.017G135000 Potri.016G069400 Potri.004G083900

Supplementary Table S3 (cont.): Details of GO terms enriched by up-regulated DEGs of Leu at **36hpi** of three infection stages in Figure 4

Process go term	Description	Matched counts	Matched genes
GO:0016051	carbohydrate biosynthetic process	6	Potri.006G004300 Potri.007G089000 Potri.007G131200 Potri.004G117800 Potri.012G126100 Potri.015G066200
GO:0005975	carbohydrate metabolic process	18	Potri.T175200 Potri.007G089400 Potri.012G126100 Potri.018G111700 Potri.008G079500 Potri.018G152200 Potri.007G131200 Potri.004G117800 Potri.006G004300 Potri.002G089800 Potri.001G087100 Potri.015G092000 Potri.004G153800 Potri.018G112000 Potri.015G066200 Potri.006G048100 Potri.003G143900 Potri.007G089000
GO:0007154	cell communication	11	Potri.011G037300 Potri.004G027400 Potri.019G120000 Potri.011G128900 Potri.004G035800

			Potri.011G125000 Potri.011G039100 Potri.010G103300 Potri.013G059900 Potri.001G411700 Potri.005G056700
GO:0000902	cell morphogenesis	6	Potri.010G205700 Potri.012G017600 Potri.003G143600 Potri.001G042600 Potri.001G087500 Potri.008G054600
GO:0008037	cell recognition	10	Potri.011G037300 Potri.004G027400 Potri.019G120000 Potri.011G128900 Potri.011G125000 Potri.011G039100 Potri.010G103300 Potri.013G059900 Potri.001G411700 Potri.005G056700
GO:0071554	cell wall organization or biogenesis	6	Potri.T175200 Potri.014G149700 Potri.004G117800 Potri.002G202600 Potri.003G191200 Potri.005G128200
GO:0044262	cellular carbohydrate metabolic process	10	Potri.012G126100 Potri.007G089000 Potri.007G131200 Potri.004G117800 Potri.006G004300 Potri.002G089800 Potri.001G087100 Potri.015G092000 Potri.015G066200 Potri.003G143900
GO:0048869	cellular developmental process	6	Potri.010G205700 Potri.012G017600 Potri.003G143600 Potri.001G042600 Potri.001G087500 Potri.008G054600
GO:0044237	cellular metabolic process	154	Potri.010G082300 Potri.017G110500 Potri.006G193000 Potri.012G011300 Potri.001G347900 Potri.015G018200 Potri.019G008900 Potri.012G124100 Potri.015G092000 Potri.016G076100 Potri.019G018100 Potri.019G005300 Potri.001G077900 Potri.018G111700 Potri.003G149700 Potri.004G181900 Potri.013G114200 Potri.014G141000 Potri.006G251800 Potri.013G158500 Potri.004G025100 Potri.019G120000 Potri.011G128900 Potri.006G049200 Potri.001G114000 Potri.007G079800 Potri.011G003900 Potri.004G096800 Potri.003G211300 Potri.004G027400 Potri.004G023800 Potri.001G080900 Potri.005G197700 Potri.011G028100 Potri.010G103300 Potri.014G096200 Potri.005G093200 Potri.013G046100 Potri.003G143900 Potri.002G168700 Potri.004G024600 Potri.003G150100 Potri.006G148800 Potri.006G224100 Potri.017G079500 Potri.019G034700 Potri.002G129600 Potri.001G154200 Potri.T128600 Potri.011G037300 Potri.002G046200 Potri.005G128200 Potri.002G129100 Potri.004G025500 Potri.013G145300 Potri.016G068200 Potri.019G131800 Potri.013G059900 Potri.008G059900 Potri.006G200600 Potri.004G191400 Potri.005G085200 Potri.002G114500 Potri.006G004300 Potri.011G028800 Potri.012G007500 Potri.005G205400 Potri.011G125000 Potri.011G028600 Potri.001G168000 Potri.T064000 Potri.006G008800 Potri.008G103300 Potri.001G014100 Potri.010G121100 Potri.002G057200 Potri.005G056700 Potri.003G139300 Potri.T080900 Potri.002G089800 Potri.019G018500 Potri.001G087100 Potri.T091200 Potri.003G132700 Potri.007G019600 Potri.013G090300 Potri.012G126100 Potri.004G209300 Potri.019G006100 Potri.007G040100 Potri.015G141100 Potri.004G117800 Potri.008G112800 Potri.008G152300 Potri.004G061900 Potri.005G241100 Potri.001G411700 Potri.005G064100 Potri.002G004900 Potri.015G018100 Potri.008G091900 Potri.014G007200 Potri.012G067600 Potri.018G148300 Potri.004G164000 Potri.007G131200 Potri.001G457000 Potri.012G101000 Potri.004G106400 Potri.012G054700 Potri.006G044100 Potri.T136500 Potri.011G061700 Potri.016G003400 Potri.002G240800 Potri.003G185800 Potri.015G061600 Potri.011G068500 Potri.007G089000 Potri.001G058800 Potri.002G009400 Potri.012G031700 Potri.009G096600 Potri.010G177900 Potri.003G048100 Potri.017G151400 Potri.011G149700 Potri.016G062600 Potri.015G147700 Potri.005G190500 Potri.018G001600 Potri.010G004200 Potri.012G090500 Potri.003G187000 Potri.004G083900 Potri.019G099300 Potri.004G097100

			Potri.004G024400 Potri.016G045000 Potri.005G228100 Potri.018G088100 Potri.010G043700 Potri.006G109100 Potri.T084700 Potri.006G263600 Potri.018G134100 Potri.019G005700 Potri.011G039100 Potri.003G185700 Potri.009G141600 Potri.004G025800 Potri.015G066200 Potri.014G156400 Potri.018G138700
GO:0044267	cellular protein metabolic process	84	Potri.015G018100 Potri.018G134100 Potri.004G191400 Potri.004G024600 Potri.010G043700 Potri.003G150100 Potri.019G006100 Potri.007G040100 Potri.015G147700 Potri.012G011300 Potri.018G088100 Potri.015G018200 Potri.019G008900 Potri.004G096800 Potri.004G025100 Potri.005G241100 Potri.010G177900 Potri.019G018100 Potri.012G090500 Potri.001G411700 Potri.005G128200 Potri.002G004900 Potri.T128600 Potri.004G209300 Potri.011G037300 Potri.019G005300 Potri.T064000 Potri.018G111700 Potri.012G067600 Potri.002G129100 Potri.004G025500 Potri.004G024400 Potri.008G059900 Potri.016G003400 Potri.013G114200 Potri.004G025800 Potri.014G141000 Potri.019G120000 Potri.019G131800 Potri.013G059900 Potri.012G054700 Potri.003G185800 Potri.004G097100 Potri.011G028800 Potri.004G061900 Potri.011G128900 Potri.T136500 Potri.002G046200 Potri.006G200600 Potri.004G083900 Potri.011G125000 Potri.011G028600 Potri.001G168000 Potri.016G045000 Potri.006G008800 Potri.018G138700 Potri.008G152300 Potri.001G014100 Potri.010G121100 Potri.005G056700 Potri.003G211300 Potri.015G061600 Potri.019G005700 Potri.004G027400 Potri.004G023800 Potri.011G068500 Potri.018G148300 Potri.011G003900 Potri.T080900 Potri.002G009400 Potri.011G039100 Potri.003G185700 Potri.002G089800 Potri.006G193000 Potri.011G028100 Potri.010G103300 Potri.T091200 Potri.017G151400 Potri.015G066200 Potri.012G124100 Potri.013G046100 Potri.T084700 Potri.014G156400 Potri.005G228100
GO:0032502	developmental process	6	Potri.010G205700 Potri.012G017600 Potri.003G143600 Potri.001G042600 Potri.001G087500 Potri.008G054600
GO:0006629	lipid metabolic process	13	Potri.T004600 Potri.017G110500 Potri.007G089000 Potri.015G069600 Potri.018G063900 Potri.007G131200 Potri.009G057900 Potri.003G081500 Potri.T004800 Potri.015G066200 Potri.014G033900 Potri.010G236800 Potri.009G086100
GO:0043170	macromolecule metabolic process	145	Potri.006G193000 Potri.012G011300 Potri.001G347900 Potri.015G018200 Potri.019G008900 Potri.012G124100 Potri.016G076100 Potri.019G018100 Potri.019G005300 Potri.001G077900 Potri.018G111700 Potri.019G006100 Potri.004G181900 Potri.013G114200 Potri.014G141000 Potri.006G251800 Potri.004G057700 Potri.013G158500 Potri.004G025100 Potri.019G120000 Potri.011G128900 Potri.006G049200 Potri.001G114000 Potri.007G079800 Potri.011G003900 Potri.004G096800 Potri.003G211300 Potri.004G027400 Potri.004G023800 Potri.001G080900 Potri.007G102100 Potri.011G028100 Potri.010G103300 Potri.012G104600 Potri.014G096200 Potri.013G046100 Potri.T084700 Potri.002G168700 Potri.004G024600 Potri.003G150100 Potri.006G148800 Potri.006G224100 Potri.017G079500 Potri.019G034700 Potri.002G129600 Potri.001G154200 Potri.T128600 Potri.011G037300 Potri.002G046200 Potri.005G128200 Potri.002G129100 Potri.004G025500 Potri.013G145300 Potri.016G068200 Potri.019G131800 Potri.013G059900 Potri.008G059900 Potri.006G200600 Potri.005G085200 Potri.002G114500 Potri.006G004300 Potri.011G028800 Potri.012G007500 Potri.005G205400 Potri.011G125000

			Potri.011G028600 Potri.001G168000 Potri.T064000 Potri.006G008800 Potri.008G103300 Potri.001G014100 Potri.010G121100 Potri.002G057200 Potri.005G056700 Potri.003G149700 Potri.003G139300 Potri.T080900 Potri.002G089800 Potri.T091200 Potri.003G132700 Potri.007G019600 Potri.013G090300 Potri.004G191400 Potri.004G209300 Potri.017G148200 Potri.007G040100 Potri.015G141100 Potri.004G117800 Potri.008G112800 Potri.008G152300 Potri.004G061900 Potri.005G241100 Potri.001G411700 Potri.005G064100 Potri.002G004900 Potri.015G018100 Potri.008G091900 Potri.014G007200 Potri.012G067600 Potri.018G148300 Potri.007G131200 Potri.012G101000 Potri.012G054700 Potri.006G044100 Potri.T136500 Potri.011G061700 Potri.016G003400 Potri.002G124500 Potri.015G061600 Potri.002G013900 Potri.011G068500 Potri.005G204600 Potri.001G058800 Potri.002G009400 Potri.012G031700 Potri.010G177900 Potri.003G048100 Potri.017G151400 Potri.011G149700 Potri.016G062600 Potri.015G147700 Potri.010G004200 Potri.008G203200 Potri.012G090500 Potri.004G083900 Potri.T175200 Potri.019G099300 Potri.003G185800 Potri.004G097100 Potri.004G024400 Potri.016G045000 Potri.005G228100 Potri.018G088100 Potri.010G043700 Potri.006G109100 Potri.006G263600 Potri.018G134100 Potri.019G005700 Potri.011G039100 Potri.003G185700 Potri.009G141600 Potri.004G025800 Potri.015G066200 Potri.014G156400 Potri.018G138700
GO:0051704	multi-organism process	10	Potri.011G037300 Potri.004G027400 Potri.019G120000 Potri.011G128900 Potri.011G125000 Potri.011G039100 Potri.010G103300 Potri.013G059900 Potri.001G411700 Potri.005G056700
GO:0055114	oxidation reduction	42	Potri.017G110500 Potri.001G457000 Potri.006G057900 Potri.009G096600 Potri.001G113900 Potri.009G143500 Potri.018G121700 Potri.T079500 Potri.005G084500 Potri.001G463300 Potri.001G167700 Potri.007G131200 Potri.012G078700 Potri.001G229500 Potri.012G006300 Potri.001G270400 Potri.003G146800 Potri.013G045000 Potri.012G049500 Potri.002G114500 Potri.001G270800 Potri.005G144000 Potri.005G135300 Potri.005G079400 Potri.012G038400 Potri.007G103800 Potri.005G064400 Potri.010G049200 Potri.003G103900 Potri.001G268600 Potri.004G149000 Potri.008G205200 Potri.011G155900 Potri.004G106400 Potri.019G057900 Potri.005G143900 Potri.001G176600 Potri.001G334700 Potri.006G070000 Potri.013G160800 Potri.015G134600 Potri.005G172400
GO:0006793	phosphorus metabolic process	74	Potri.015G018100 Potri.018G134100 Potri.004G191400 Potri.004G024600 Potri.010G043700 Potri.003G150100 Potri.019G006100 Potri.012G011300 Potri.018G088100 Potri.015G018200 Potri.019G008900 Potri.004G096800 Potri.004G025100 Potri.019G018100 Potri.012G090500 Potri.001G411700 Potri.005G128200 Potri.002G004900 Potri.T128600 Potri.004G209300 Potri.011G037300 Potri.019G005300 Potri.T064000 Potri.018G111700 Potri.012G067600 Potri.002G129100 Potri.004G025500 Potri.004G024400 Potri.008G059900 Potri.013G114200 Potri.011G028100 Potri.019G120000 Potri.019G131800 Potri.013G059900 Potri.012G054700 Potri.003G185800 Potri.004G097100 Potri.011G028800 Potri.004G061900 Potri.011G128900 Potri.T136500 Potri.006G200600 Potri.011G125000 Potri.011G028600 Potri.001G168000 Potri.016G045000 Potri.018G138700 Potri.008G152300 Potri.001G014100 Potri.010G121100 Potri.005G056700 Potri.003G211300 Potri.015G061600 Potri.019G005700 Potri.004G027400 Potri.004G023800 Potri.011G068500 Potri.018G148300 Potri.011G003900

			Potri.T080900 Potri.002G009400 Potri.011G039100 Potri.003G185700 Potri.010G177900 Potri.006G193000 Potri.004G025800 Potri.010G103300 Potri.T091200 Potri.017G151400 Potri.015G066200 Potri.012G124100 Potri.013G046100 Potri.T084700 Potri.014G156400
GO:0016310	phosphorylation	74	Potri.015G018100 Potri.018G134100 Potri.004G191400 Potri.004G024600 Potri.010G043700 Potri.003G150100 Potri.019G006100 Potri.012G011300 Potri.018G088100 Potri.015G018200 Potri.019G008900 Potri.004G096800 Potri.004G025100 Potri.019G018100 Potri.012G090500 Potri.001G411700 Potri.005G128200 Potri.002G004900 Potri.T128600 Potri.004G209300 Potri.011G037300 Potri.019G005300 Potri.T064000 Potri.018G111700 Potri.012G067600 Potri.002G129100 Potri.004G025500 Potri.004G024400 Potri.008G059900 Potri.013G114200 Potri.011G028100 Potri.019G120000 Potri.019G131800 Potri.013G059900 Potri.012G054700 Potri.003G185800 Potri.004G097100 Potri.011G028800 Potri.004G061900 Potri.011G128900 Potri.T136500 Potri.006G200600 Potri.011G125000 Potri.011G028600 Potri.001G168000 Potri.016G045000 Potri.018G138700 Potri.008G152300 Potri.001G014100 Potri.010G121100 Potri.005G056700 Potri.003G211300 Potri.015G061600 Potri.019G005700 Potri.004G027400 Potri.004G023800 Potri.011G068500 Potri.018G148300 Potri.011G003900 Potri.T080900 Potri.002G009400 Potri.011G039100 Potri.003G185700 Potri.010G177900 Potri.006G193000 Potri.004G025800 Potri.010G103300 Potri.T091200 Potri.017G151400 Potri.015G066200 Potri.012G124100 Potri.013G046100 Potri.T084700 Potri.014G156400
GO:0005976	polysaccharide metabolic process	5	Potri.T175200 Potri.006G004300 Potri.007G131200 Potri.004G117800 Potri.015G066200
GO:0044238	primary metabolic process	175	Potri.017G110500 Potri.006G193000 Potri.012G011300 Potri.001G347900 Potri.015G018200 Potri.019G008900 Potri.012G124100 Potri.015G092000 Potri.016G076100 Potri.019G018100 Potri.019G005300 Potri.001G077900 Potri.018G111700 Potri.019G006100 Potri.004G181900 Potri.013G114200 Potri.014G141000 Potri.006G251800 Potri.004G057700 Potri.T004800 Potri.014G033900 Potri.008G103300 Potri.004G025100 Potri.019G120000 Potri.011G128900 Potri.011G125000 Potri.018G063900 Potri.006G049200 Potri.001G114000 Potri.007G079800 Potri.011G003900 Potri.004G096800 Potri.001G457000 Potri.003G211300 Potri.004G027400 Potri.004G023800 Potri.001G080900 Potri.007G102100 Potri.011G028100 Potri.010G103300 Potri.012G104600 Potri.008G079500 Potri.014G096200 Potri.005G093200 Potri.013G046100 Potri.003G143900 Potri.002G168700 Potri.004G024600 Potri.003G150100 Potri.006G148800 Potri.006G224100 Potri.017G079500 Potri.019G034700 Potri.003G081500 Potri.002G129600 Potri.001G154200 Potri.T128600 Potri.011G037300 Potri.002G046200 Potri.005G128200 Potri.002G129100 Potri.004G025500 Potri.013G145300 Potri.016G068200 Potri.019G131800 Potri.013G059900 Potri.008G059900 Potri.006G200600 Potri.004G191400 Potri.005G085200 Potri.002G114500 Potri.006G004300 Potri.011G028800 Potri.012G007500 Potri.005G205400 Potri.T004600 Potri.011G028600 Potri.001G168000 Potri.T064000 Potri.006G008800 Potri.018G112000 Potri.001G014100 Potri.010G121100 Potri.002G057200 Potri.005G056700 Potri.003G149700 Potri.015G069600 Potri.003G139300 Potri.T080900 Potri.002G089800 Potri.019G018500 Potri.001G087100 Potri.T091200 Potri.003G132700 Potri.007G019600

			Potri.013G090300 Potri.012G126100 Potri.004G209300 Potri.017G148200 Potri.007G040100 Potri.015G141100 Potri.004G117800 Potri.008G112800 Potri.008G152300 Potri.004G061900 Potri.005G241100 Potri.001G411700 Potri.005G064100 Potri.002G004900 Potri.015G018100 Potri.008G091900 Potri.014G007200 Potri.012G067600 Potri.018G148300 Potri.004G164000 Potri.007G131200 Potri.018G152200 Potri.012G101000 Potri.006G263600 Potri.012G054700 Potri.010G236800 Potri.013G158500 Potri.006G044100 Potri.T136500 Potri.009G057900 Potri.011G061700 Potri.018G138700 Potri.002G240800 Potri.002G124500 Potri.015G061600 Potri.009G086100 Potri.002G013900 Potri.011G068500 Potri.005G204600 Potri.007G089000 Potri.001G058800 Potri.002G009400 Potri.012G031700 Potri.009G096600 Potri.010G177900 Potri.003G048100 Potri.004G153800 Potri.017G151400 Potri.011G149700 Potri.016G062600 Potri.015G147700 Potri.018G001600 Potri.010G004200 Potri.008G203200 Potri.012G090500 Potri.003G187000 Potri.004G083900 Potri.T175200 Potri.007G089400 Potri.019G099300 Potri.003G185800 Potri.004G097100 Potri.004G024400 Potri.016G045000 Potri.005G228100 Potri.018G088100 Potri.010G043700 Potri.006G109100 Potri.T084700 Potri.004G106400 Potri.018G134100 Potri.019G005700 Potri.011G039100 Potri.003G185700 Potri.009G141600 Potri.004G025800 Potri.015G066200 Potri.006G048100 Potri.014G156400 Potri.016G003400
GO:0019538	protein metabolic process	92	Potri.006G193000 Potri.012G011300 Potri.015G018200 Potri.019G008900 Potri.012G124100 Potri.019G018100 Potri.019G005300 Potri.T064000 Potri.018G111700 Potri.019G006100 Potri.013G114200 Potri.014G141000 Potri.002G124500 Potri.004G057700 Potri.004G025100 Potri.019G120000 Potri.011G128900 Potri.011G003900 Potri.004G096800 Potri.003G211300 Potri.004G027400 Potri.004G023800 Potri.007G102100 Potri.011G028100 Potri.010G103300 Potri.012G104600 Potri.013G046100 Potri.T084700 Potri.004G024600 Potri.015G018100 Potri.T128600 Potri.011G037300 Potri.002G046200 Potri.002G129100 Potri.004G025500 Potri.008G059900 Potri.019G131800 Potri.013G059900 Potri.011G028800 Potri.006G200600 Potri.011G125000 Potri.011G028600 Potri.001G168000 Potri.006G008800 Potri.008G152300 Potri.010G121100 Potri.005G056700 Potri.T080900 Potri.002G089800 Potri.T091200 Potri.004G191400 Potri.004G209300 Potri.017G148200 Potri.007G040100 Potri.001G014100 Potri.004G061900 Potri.005G241100 Potri.001G411700 Potri.005G128200 Potri.002G004900 Potri.003G150100 Potri.012G067600 Potri.018G148300 Potri.012G054700 Potri.T136500 Potri.016G003400 Potri.003G185800 Potri.015G061600 Potri.002G013900 Potri.011G068500 Potri.005G204600 Potri.002G009400 Potri.010G177900 Potri.017G151400 Potri.015G147700 Potri.008G203200 Potri.012G090500 Potri.004G083900 Potri.004G097100 Potri.004G024400 Potri.016G045000 Potri.005G228100 Potri.018G088100 Potri.010G043700 Potri.018G134100 Potri.019G005700 Potri.011G039100 Potri.003G185700 Potri.004G025800 Potri.015G066200 Potri.014G156400 Potri.018G138700
GO:0006464	protein modification process	78	Potri.015G018100 Potri.018G134100 Potri.004G191400 Potri.004G024600 Potri.010G043700 Potri.003G150100 Potri.019G006100 Potri.012G011300 Potri.018G088100 Potri.015G018200 Potri.019G008900 Potri.004G096800 Potri.004G025100 Potri.010G177900 Potri.019G018100 Potri.012G090500 Potri.001G411700 Potri.005G128200 Potri.002G004900 Potri.T128600 Potri.004G209300 Potri.011G037300 Potri.019G005300

			Potri.T064000 Potri.018G111700 Potri.012G067600 Potri.002G129100 Potri.004G025500 Potri.004G024400 Potri.008G059900 Potri.013G114200 Potri.004G025800 Potri.019G120000 Potri.019G131800 Potri.013G059900 Potri.012G054700 Potri.003G185800 Potri.004G097100 Potri.011G028800 Potri.004G061900 Potri.011G128900 Potri.T136500 Potri.002G046200 Potri.006G200600 Potri.004G083900 Potri.011G125000 Potri.011G028600 Potri.001G168000 Potri.016G045000 Potri.005G228100 Potri.008G152300 Potri.001G014100 Potri.010G121100 Potri.005G056700 Potri.003G211300 Potri.015G061600 Potri.019G005700 Potri.004G027400 Potri.004G023800 Potri.011G068500 Potri.018G148300 Potri.011G003900 Potri.T080900 Potri.002G009400 Potri.011G039100 Potri.003G185700 Potri.002G089800 Potri.006G193000 Potri.011G028100 Potri.010G103300 Potri.T091200 Potri.017G151400 Potri.015G066200 Potri.012G124100 Potri.013G046100 Potri.T084700 Potri.014G156400 Potri.018G138700
GO:0080090	regulation of primary metabolic process	42	Potri.011G149700 Potri.006G148800 Potri.015G141100 Potri.006G049200 Potri.010G004200 Potri.019G034700 Potri.016G076100 Potri.002G129600 Potri.005G064100 Potri.001G154200 Potri.008G091900 Potri.014G007200 Potri.001G077900 Potri.003G149700 Potri.012G007500 Potri.004G181900 Potri.016G068200 Potri.006G224100 Potri.012G101000 Potri.019G099300 Potri.013G158500 Potri.005G085200 Potri.017G079500 Potri.005G205400 Potri.001G114000 Potri.011G061700 Potri.013G090300 Potri.008G103300 Potri.002G057200 Potri.006G109100 Potri.006G251800 Potri.006G263600 Potri.007G079800 Potri.001G080900 Potri.003G139300 Potri.001G058800 Potri.012G031700 Potri.009G141600 Potri.003G048100 Potri.014G096200 Potri.003G132700 Potri.002G168700
GO:0023052	signaling	17	Potri.011G037300 Potri.004G027400 Potri.004G230000 Potri.007G143300 Potri.019G120000 Potri.011G128900 Potri.004G035800 Potri.011G125000 Potri.011G039100 Potri.007G142500 Potri.010G103300 Potri.013G059900 Potri.017G084800 Potri.007G143000 Potri.001G411700 Potri.005G056700 Potri.008G220200
GO:0055085	transmembrane transport	27	Potri.002G078100 Potri.009G053800 Potri.005G256100 Potri.012G051900 Potri.010G119100 Potri.003G197100 Potri.006G110500 Potri.003G198400 Potri.018G149700 Potri.002G187400 Potri.004G162000 Potri.015G081300 Potri.014G097900 Potri.011G002600 Potri.006G266000 Potri.001G258600 Potri.001G375300 Potri.002G005500 Potri.012G131300 Potri.015G026700 Potri.006G240000 Potri.012G050500 Potri.003G154600 Potri.018G035500 Potri.001G165000 Potri.006G006800 Potri.004G019900
**Function go term**	**Description**	**Matched counts**	**Matched genes**
GO:0022804	active transmembrane transporter activity	16	Potri.002G187400 Potri.012G050500 Potri.003G154600 Potri.002G078100 Potri.004G162000 Potri.001G174500 Potri.003G198400 Potri.013G026700 Potri.011G002600 Potri.012G051900 Potri.010G119100 Potri.012G081800 Potri.003G197100 Potri.001G165000 Potri.012G131300 Potri.018G149700
GO:0030554	adenyl nucleotide binding	115	Potri.019G020700 Potri.006G193000 Potri.012G011300 Potri.015G018200 Potri.019G008900 Potri.012G124100 Potri.008G054600 Potri.019G018100 Potri.001G463300 Potri.019G005300 Potri.T064000 Potri.018G111700 Potri.008G220200 Potri.013G114200 Potri.011G040800 Potri.007G020900 Potri.012G069700

			Potri.004G025100 Potri.019G120000 Potri.011G128900 Potri.012G017600 Potri.001G114000 Potri.009G119100 Potri.011G003900 Potri.004G096800 Potri.003G211300 Potri.004G027400 Potri.004G023800 Potri.013G030600 Potri.002G216800 Potri.011G028100 Potri.010G103300 Potri.013G046100 Potri.002G009400 Potri.004G024600 Potri.015G018100 Potri.001G182600 Potri.004G091300 Potri.T128600 Potri.011G037300 Potri.012G069800 Potri.002G129100 Potri.004G025500 Potri.004G012500 Potri.007G142500 Potri.019G131800 Potri.013G059900 Potri.008G059900 Potri.002G114500 Potri.012G032400 Potri.011G028800 Potri.006G200600 Potri.011G125000 Potri.T171900 Potri.001G168000 Potri.001G087500 Potri.008G152300 Potri.010G121100 Potri.005G056700 Potri.014G097700 Potri.T080900 Potri.T091200 Potri.007G019600 Potri.004G191400 Potri.004G209300 Potri.019G006100 Potri.001G014100 Potri.004G061900 Potri.005G241100 Potri.001G042600 Potri.001G411700 Potri.005G128200 Potri.002G004900 Potri.003G150100 Potri.014G007200 Potri.012G067600 Potri.018G148300 Potri.012G054700 Potri.017G140100 Potri.T136500 Potri.004G162000 Potri.018G138700 Potri.011G143300 Potri.003G185800 Potri.015G061600 Potri.011G068500 Potri.T084700 Potri.010G177900 Potri.009G143500 Potri.017G151400 Potri.003G143600 Potri.016G013900 Potri.007G143300 Potri.012G090500 Potri.008G047900 Potri.003G198400 Potri.018G149700 Potri.004G097100 Potri.002G187400 Potri.004G024400 Potri.016G045000 Potri.010G205700 Potri.018G088100 Potri.010G043700 Potri.011G155900 Potri.018G134100 Potri.003G197100 Potri.019G005700 Potri.011G039100 Potri.003G185700 Potri.004G025800 Potri.015G066200 Potri.001G165000 Potri.014G156400 Potri.016G003400
GO:0005524	ATP binding	111	Potri.019G020700 Potri.006G193000 Potri.012G011300 Potri.015G018200 Potri.019G008900 Potri.012G124100 Potri.008G054600 Potri.019G018100 Potri.019G005300 Potri.T064000 Potri.018G111700 Potri.008G220200 Potri.013G114200 Potri.011G040800 Potri.007G020900 Potri.012G069700 Potri.004G025100 Potri.019G120000 Potri.011G128900 Potri.012G017600 Potri.001G114000 Potri.009G119100 Potri.011G003900 Potri.004G096800 Potri.003G211300 Potri.004G027400 Potri.004G023800 Potri.013G030600 Potri.002G216800 Potri.011G028100 Potri.010G103300 Potri.013G046100 Potri.T084700 Potri.004G024600 Potri.015G018100 Potri.001G182600 Potri.004G091300 Potri.T128600 Potri.011G037300 Potri.012G069800 Potri.002G129100 Potri.004G025500 Potri.004G012500 Potri.007G142500 Potri.019G131800 Potri.013G059900 Potri.008G059900 Potri.012G032400 Potri.011G028800 Potri.006G200600 Potri.011G125000 Potri.T171900 Potri.001G168000 Potri.001G087500 Potri.008G152300 Potri.010G121100 Potri.005G056700 Potri.014G097700 Potri.T080900 Potri.T091200 Potri.007G019600 Potri.004G191400 Potri.004G209300 Potri.019G006100 Potri.001G014100 Potri.004G061900 Potri.005G241100 Potri.001G042600 Potri.001G411700 Potri.005G128200 Potri.002G004900 Potri.003G150100 Potri.014G007200 Potri.012G067600 Potri.018G148300 Potri.012G054700 Potri.017G140100 Potri.T136500 Potri.004G162000 Potri.018G138700 Potri.011G143300 Potri.003G185800 Potri.015G061600 Potri.011G068500 Potri.002G009400 Potri.010G177900 Potri.017G151400 Potri.003G143600 Potri.016G013900 Potri.007G143300 Potri.012G090500 Potri.008G047900 Potri.003G198400 Potri.018G149700 Potri.004G097100 Potri.002G187400 Potri.004G024400 Potri.016G045000 Potri.010G205700 Potri.018G088100 Potri.010G043700

			Potri.018G134100 Potri.003G197100 Potri.019G005700 Potri.011G039100 Potri.003G185700 Potri.004G025800 Potri.015G066200 Potri.001G165000 Potri.014G156400 Potri.016G003400
GO:0005509	calcium ion binding	6	Potri.004G191400 Potri.018G141100 Potri.T080900 Potri.019G055200 Potri.003G185700 Potri.017G084800
GO:0004091	carboxylesterase activity	9	Potri.010G082300 Potri.015G069600 Potri.014G149700 Potri.T004600 Potri.009G057900 Potri.003G081500 Potri.T004800 Potri.002G202600 Potri.009G086100
GO:0050662	coenzyme binding	9	Potri.001G463300 Potri.017G110500 Potri.001G457000 Potri.007G131200 Potri.005G197700 Potri.009G096600 Potri.009G143500 Potri.011G155900 Potri.002G114500
GO:0030234	enzyme regulator activity	5	Potri.014G149700 Potri.002G202600 Potri.019G124500 Potri.019G124700 Potri.004G035800
GO:0020037	heme binding	18	Potri.019G057900 Potri.005G143900 Potri.012G049500 Potri.005G135300 Potri.005G144000 Potri.006G057900 Potri.001G334700 Potri.004G149000 Potri.001G270400 Potri.001G113900 Potri.003G146800 Potri.005G064400 Potri.010G049200 Potri.005G084500 Potri.013G160800 Potri.001G270800 Potri.008G205200 Potri.005G172400
GO:0016787	hydrolase activity	59	Potri.001G032400 Potri.010G082300 Potri.008G203200 Potri.017G148200 Potri.018G152200 Potri.001G182600 Potri.001G347900 Potri.014G022900 Potri.016G013900 Potri.018G141100 Potri.003G081500 Potri.010G184200 Potri.T175200 Potri.014G007200 Potri.018G024200 Potri.008G047900 Potri.018G111700 Potri.014G097700 Potri.015G069600 Potri.003G198400 Potri.003G161100 Potri.004G057700 Potri.018G063900 Potri.006G110900 Potri.T004800 Potri.003G106100 Potri.009G086100 Potri.010G236800 Potri.012G069700 Potri.002G187400 Potri.007G089400 Potri.004G162000 Potri.003G192600 Potri.001G114000 Potri.T004600 Potri.009G057900 Potri.001G069200 Potri.006G008800 Potri.018G112000 Potri.005G204600 Potri.001G127700 Potri.002G171200 Potri.002G124500 Potri.012G069800 Potri.002G013900 Potri.003G197100 Potri.014G149700 Potri.015G095900 Potri.007G102100 Potri.018G149700 Potri.001G087100 Potri.004G153800 Potri.012G104600 Potri.008G079500 Potri.002G202600 Potri.001G165000 Potri.006G048100 Potri.003G143900 Potri.007G019600
GO:0016798	hydrolase activity, acting on glycosyl bonds	13	Potri.T175200 Potri.007G089400 Potri.018G024200 Potri.018G141100 Potri.018G111700 Potri.018G112000 Potri.018G152200 Potri.001G087100 Potri.004G153800 Potri.008G079500 Potri.006G048100 Potri.003G143900 Potri.010G184200
GO:0005506	iron ion binding	18	Potri.019G057900 Potri.005G143900 Potri.012G049500 Potri.005G135300 Potri.005G144000 Potri.006G057900 Potri.001G334700 Potri.004G149000 Potri.001G270400 Potri.001G113900 Potri.003G146800 Potri.005G064400 Potri.010G049200 Potri.005G084500 Potri.013G160800 Potri.001G270800 Potri.008G205200 Potri.005G172400
GO:0016298	lipase activity	6	Potri.015G069600 Potri.T004600 Potri.009G057900 Potri.003G081500 Potri.T004800 Potri.009G086100
GO:0017111	nucleoside-triphosphatase activity	14	Potri.012G069700 Potri.002G187400 Potri.003G197100 Potri.014G097700 Potri.004G162000 Potri.001G182600 Potri.003G198400 Potri.016G013900 Potri.008G047900 Potri.001G165000 Potri.002G171200 Potri.018G149700 Potri.007G019600 Potri.012G069800

GO:0016491	oxidoreductase activity	45	Potri.017G110500 Potri.001G457000 Potri.001G176600 Potri.006G057900 Potri.009G096600 Potri.001G113900 Potri.009G143500 Potri.018G121700 Potri.T079500 Potri.005G084500 Potri.001G463300 Potri.001G167700 Potri.001G270400 Potri.007G131200 Potri.012G078700 Potri.001G229500 Potri.012G006300 Potri.010G097600 Potri.003G146800 Potri.013G045000 Potri.012G049500 Potri.002G114500 Potri.001G270800 Potri.005G144000 Potri.005G135300 Potri.005G079400 Potri.012G038400 Potri.007G103800 Potri.005G064400 Potri.010G049200 Potri.001G268600 Potri.004G149000 Potri.008G205200 Potri.011G155900 Potri.004G106400 Potri.019G057900 Potri.008G144800 Potri.007G134800 Potri.005G143900 Potri.005G197700 Potri.001G334700 Potri.006G070000 Potri.013G160800 Potri.015G134600 Potri.005G172400
GO:0004672	protein kinase activity	74	Potri.015G018100 Potri.018G134100 Potri.004G191400 Potri.004G024600 Potri.010G043700 Potri.003G150100 Potri.019G006100 Potri.012G011300 Potri.018G088100 Potri.015G018200 Potri.019G008900 Potri.004G096800 Potri.004G025100 Potri.019G018100 Potri.012G090500 Potri.001G411700 Potri.005G128200 Potri.002G004900 Potri.T128600 Potri.004G209300 Potri.011G037300 Potri.019G005300 Potri.T064000 Potri.018G111700 Potri.012G067600 Potri.002G129100 Potri.004G025500 Potri.004G024400 Potri.008G059900 Potri.013G114200 Potri.011G028100 Potri.019G120000 Potri.019G131800 Potri.013G059900 Potri.012G054700 Potri.003G185800 Potri.004G097100 Potri.011G028800 Potri.004G061900 Potri.011G128900 Potri.T136500 Potri.006G200600 Potri.011G125000 Potri.011G028600 Potri.001G168000 Potri.016G045000 Potri.018G138700 Potri.008G152300 Potri.001G014100 Potri.010G121100 Potri.005G056700 Potri.003G211300 Potri.015G061600 Potri.019G005700 Potri.004G027400 Potri.004G023800 Potri.011G068500 Potri.018G148300 Potri.011G003900 Potri.T080900 Potri.002G009400 Potri.011G039100 Potri.003G185700 Potri.010G177900 Potri.006G193000 Potri.004G025800 Potri.010G103300 Potri.T091200 Potri.017G151400 Potri.015G066200 Potri.012G124100 Potri.013G046100 Potri.T084700 Potri.014G156400
GO:0004674	protein serine/threonine kinase activity	10	Potri.011G037300 Potri.004G191400 Potri.019G120000 Potri.011G128900 Potri.018G148300 Potri.T080900 Potri.011G125000 Potri.011G039100 Potri.001G411700 Potri.003G185800
GO:0003700	transcription factor activity	25	Potri.015G141100 Potri.006G049200 Potri.010G004200 Potri.001G154200 Potri.008G091900 Potri.001G077900 Potri.016G068200 Potri.006G224100 Potri.012G101000 Potri.013G158500 Potri.005G085200 Potri.017G079500 Potri.011G061700 Potri.013G090300 Potri.008G103300 Potri.006G109100 Potri.006G251800 Potri.006G263600 Potri.007G079800 Potri.003G139300 Potri.001G058800 Potri.012G031700 Potri.014G096200 Potri.003G132700 Potri.002G168700
GO:0016757	transferase activity, transferring glycosyl groups	14	Potri.001G158000 Potri.006G120600 Potri.009G077500 Potri.004G164000 Potri.004G117800 Potri.006G004300 Potri.002G089800 Potri.016G097400 Potri.006G048500 Potri.003G191200 Potri.016G016500 Potri.001G282100 Potri.016G016100 Potri.015G045500
GO:0016772	transferase activity, transferring phosphorus-containing	77	Potri.015G018100 Potri.008G135800 Potri.004G191400 Potri.004G024600 Potri.010G043700 Potri.003G150100 Potri.019G006100 Potri.012G011300 Potri.018G088100 Potri.001G347900 Potri.015G018200 Potri.008G112800 Potri.019G008900 Potri.004G096800 Potri.004G025100 Potri.018G134100 Potri.019G018100

	groups		Potri.012G090500 Potri.001G411700 Potri.005G128200 Potri.002G004900 Potri.T128600 Potri.004G209300 Potri.011G037300 Potri.019G005300 Potri.T064000 Potri.018G111700 Potri.012G067600 Potri.002G129100 Potri.004G025500 Potri.004G024400 Potri.008G059900 Potri.013G114200 Potri.011G028100 Potri.019G120000 Potri.019G131800 Potri.013G059900 Potri.012G054700 Potri.003G185800 Potri.004G097100 Potri.011G028800 Potri.004G061900 Potri.011G128900 Potri.T136500 Potri.006G200600 Potri.011G125000 Potri.011G028600 Potri.001G168000 Potri.016G045000 Potri.018G138700 Potri.008G152300 Potri.001G014100 Potri.010G121100 Potri.005G056700 Potri.003G211300 Potri.015G061600 Potri.019G005700 Potri.004G027400 Potri.004G023800 Potri.011G068500 Potri.018G148300 Potri.011G003900 Potri.T080900 Potri.002G009400 Potri.011G039100 Potri.003G185700 Potri.010G177900 Potri.006G193000 Potri.004G025800 Potri.010G103300 Potri.T091200 Potri.017G151400 Potri.015G066200 Potri.012G124100 Potri.013G046100 Potri.T084700 Potri.014G156400
GO:0005215	transporter activity	34	Potri.001G375200 Potri.002G078100 Potri.009G053800 Potri.001G174500 Potri.012G051900 Potri.010G119100 Potri.012G071400 Potri.009G038800 Potri.001G258600 Potri.003G198400 Potri.001G044900 Potri.005G256100 Potri.018G149700 Potri.008G170100 Potri.002G187400 Potri.004G162000 Potri.014G097900 Potri.004G052400 Potri.011G002600 Potri.010G100800 Potri.003G197100 Potri.002G005500 Potri.012G131300 Potri.005G102600 Potri.001G374600 Potri.006G240000 Potri.012G050500 Potri.010G034700 Potri.003G154600 Potri.018G035500 Potri.013G026700 Potri.012G081800 Potri.001G165000 Potri.006G006800

Supplementary Table S3 (cont.): Details of GO terms enriched by up-regulated DEGs of Leu at **96hpi** of three infection stages in Figure 4

Process go term	Description	Matched counts	Matched genes
GO:0009309	amine biosynthetic process	13	Potri.010G153500 Potri.014G022800 Potri.005G162800 Potri.017G108800 Potri.005G073300 Potri.012G011700 Potri.013G061800 Potri.018G001600 Potri.012G043900 Potri.008G099300 Potri.008G040700 Potri.005G093200 Potri.004G106400
GO:0016051	carbohydrate biosynthetic process	23	Potri.007G011200 Potri.004G117800 Potri.012G126100 Potri.002G204400 Potri.006G022000 Potri.009G168600 Potri.007G020100 Potri.009G066100 Potri.007G131200 Potri.011G103700 Potri.002G173600 Potri.005G116200 Potri.019G049700 Potri.007G127000 Potri.006G004300 Potri.006G175500 Potri.012G071700 Potri.001G136200 Potri.008G053100 Potri.007G089000 Potri.014G129200 Potri.012G047300 Potri.015G066200
GO:0005975	carbohydrate metabolic process	99	Potri.006G068300 Potri.015G092000 Potri.015G023900 Potri.018G094000 Potri.009G168600 Potri.T167100 Potri.007G020100 Potri.018G111700 Potri.008G055900 Potri.008G174100 Potri.019G049700 Potri.012G047300 Potri.006G175500 Potri.009G060400 Potri.017G100200 Potri.001G223500 Potri.012G071700 Potri.001G136200 Potri.005G069000 Potri.016G015700 Potri.002G060500 Potri.002G204400 Potri.003G030900 Potri.008G079500

			Potri.004G110200 Potri.003G143900 Potri.019G093800 Potri.014G161300 Potri.001G224000 Potri.004G019500 Potri.014G184900 Potri.014G183800 Potri.002G173600 Potri.010G142800 Potri.009G080600 Potri.017G106900 Potri.013G125000 Potri.007G127000 Potri.006G004300 Potri.018G088300 Potri.018G095100 Potri.001G337400 Potri.018G112000 Potri.011G156100 Potri.001G222900 Potri.001G223800 Potri.002G089800 Potri.007G010300 Potri.001G409900 Potri.009G163700 Potri.017G063500 Potri.012G126100 Potri.018G063500 Potri.004G117800 Potri.006G144500 Potri.003G097300 Potri.001G222800 Potri.004G081300 Potri.009G066100 Potri.007G131200 Potri.011G103700 Potri.002G219600 Potri.T018000 Potri.008G053100 Potri.001G223900 Potri.002G093300 Potri.007G089000 Potri.005G167800 Potri.004G153800 Potri.007G011200 Potri.018G152200 Potri.014G141900 Potri.014G129200 Potri.016G028900 Potri.006G022000 Potri.T175200 Potri.006G048100 Potri.004G019300 Potri.007G105800 Potri.005G116200 Potri.001G255100 Potri.007G089400 Potri.004G109200 Potri.006G046100 Potri.T019200 Potri.014G111800 Potri.016G057400 Potri.002G126300 Potri.019G010800 Potri.014G185100 Potri.015G024200 Potri.012G033900 Potri.010G159900 Potri.001G068200 Potri.017G075800 Potri.010G141400 Potri.015G066200 Potri.019G093700 Potri.003G135600
GO:0046394	carboxylic acid biosynthetic process	16	Potri.010G153500 Potri.014G022800 Potri.005G162800 Potri.012G043900 Potri.005G073300 Potri.012G011700 Potri.013G061800 Potri.018G001600 Potri.008G099300 Potri.001G051500 Potri.008G040700 Potri.006G177500 Potri.005G093200 Potri.005G167400 Potri.001G100200 Potri.004G106400
GO:0007154	cell communication	50	Potri.010G025700 Potri.T022800 Potri.001G410800 Potri.T023600 Potri.011G034200 Potri.011G129000 Potri.011G037900 Potri.010G017100 Potri.011G035900 Potri.013G060000 Potri.010G025800 Potri.010G015400 Potri.T021200 Potri.001G409400 Potri.010G153400 Potri.001G228200 Potri.016G053800 Potri.007G111100 Potri.001G418100 Potri.011G037100 Potri.011G129300 Potri.010G018100 Potri.013G059900 Potri.001G411700 Potri.019G120000 Potri.011G037300 Potri.005G014700 Potri.010G018300 Potri.011G128900 Potri.011G035100 Potri.011G125000 Potri.011G034300 Potri.011G036500 Potri.011G036400 Potri.T022600 Potri.016G102500 Potri.011G035300 Potri.005G056700 Potri.004G027400 Potri.T021600 Potri.004G035800 Potri.011G039100 Potri.001G412400 Potri.013G121000 Potri.011G034400 Potri.010G103300 Potri.010G017800 Potri.011G034500 Potri.010G025500 Potri.001G414200
GO:0000902	cell morphogenesis	8	Potri.010G206600 Potri.012G017600 Potri.003G143600 Potri.008G054000 Potri.010G205700 Potri.001G087500 Potri.008G054600 Potri.001G042600
GO:0008037	cell recognition	48	Potri.010G025700 Potri.T022800 Potri.001G410800 Potri.T023600 Potri.011G034200 Potri.011G129000 Potri.011G037900 Potri.010G017100 Potri.011G035900 Potri.013G060000 Potri.010G025800 Potri.010G015400 Potri.T021200 Potri.001G409400 Potri.001G228200 Potri.016G053800 Potri.007G111100 Potri.001G418100 Potri.011G037100 Potri.011G129300 Potri.010G018100 Potri.013G059900 Potri.001G411700 Potri.019G120000 Potri.011G037300 Potri.005G014700 Potri.010G018300 Potri.011G128900 Potri.011G035100 Potri.011G125000 Potri.011G034300 Potri.011G036500 Potri.011G036400 Potri.T022600 Potri.016G102500 Potri.011G035300 Potri.005G056700 Potri.004G027400 Potri.T021600 Potri.011G039100 Potri.001G412400 Potri.013G121000

			Potri.011G034400 Potri.010G103300 Potri.010G017800 Potri.011G034500 Potri.010G025500 Potri.001G414200
GO:0044036	cell wall macromolecule metabolic process	8	Potri.T175200 Potri.010G078700 Potri.019G093800 Potri.014G111800 Potri.002G152600 Potri.005G128200 Potri.013G125000 Potri.019G093700
GO:0071554	cell wall organization or biogenesis	17	Potri.T175200 Potri.011G025400 Potri.001G033800 Potri.002G145500 Potri.010G078700 Potri.019G093800 Potri.002G202600 Potri.004G117800 Potri.014G111800 Potri.015G127700 Potri.014G127000 Potri.002G152600 Potri.003G191200 Potri.001G034100 Potri.005G128200 Potri.013G125000 Potri.019G093700
GO:0044262	cellular carbohydrate metabolic process	44	Potri.017G063500 Potri.007G011200 Potri.018G063500 Potri.014G161300 Potri.004G117800 Potri.012G126100 Potri.014G141900 Potri.006G068300 Potri.015G092000 Potri.002G204400 Potri.002G089800 Potri.018G094000 Potri.006G022000 Potri.009G168600 Potri.007G020100 Potri.004G081300 Potri.009G066100 Potri.007G131200 Potri.011G103700 Potri.002G173600 Potri.005G116200 Potri.019G049700 Potri.009G080600 Potri.017G106900 Potri.007G127000 Potri.006G004300 Potri.006G175500 Potri.018G095100 Potri.017G100200 Potri.012G071700 Potri.001G337400 Potri.001G136200 Potri.008G053100 Potri.002G093300 Potri.011G156100 Potri.007G089000 Potri.002G060500 Potri.014G129200 Potri.007G010300 Potri.003G097300 Potri.003G030900 Potri.015G066200 Potri.003G143900 Potri.003G135600
GO:0048869	cellular developmental process	8	Potri.010G206600 Potri.012G017600 Potri.003G143600 Potri.008G054000 Potri.010G205700 Potri.001G087500 Potri.008G054600 Potri.001G042600
GO:0006073	cellular glucan metabolic process	8	Potri.006G004300 Potri.018G095100 Potri.002G060500 Potri.004G117800 Potri.002G173600 Potri.001G136200 Potri.019G049700 Potri.003G097300
GO:0044237	cellular metabolic process	599	Potri.005G214100 Potri.001G404100 Potri.012G011300 Potri.019G078300 Potri.019G008900 Potri.002G033600 Potri.008G099300 Potri.009G081800 Potri.019G005300 Potri.001G077900 Potri.010G015400 Potri.002G047500 Potri.012G023300 Potri.013G133100 Potri.018G091000 Potri.014G164100 Potri.017G003100 Potri.015G074200 Potri.007G039800 Potri.017G141300 Potri.018G036600 Potri.019G094200 Potri.019G018500 Potri.004G038000 Potri.T003400 Potri.011G128900 Potri.001G310500 Potri.007G085300 Potri.010G081200 Potri.011G034300 Potri.002G113400 Potri.010G192100 Potri.001G136200 Potri.001G442200 Potri.002G186600 Potri.016G015700 Potri.017G009600 Potri.017G055400 Potri.T090000 Potri.002G075900 Potri.003G085300 Potri.013G121000 Potri.011G028100 Potri.018G019700 Potri.001G114000 Potri.002G114500 Potri.014G096200 Potri.T084700 Potri.002G168700 Potri.013G046400 Potri.004G024600 Potri.003G066300 Potri.T108900 Potri.T022800 Potri.005G113600 Potri.014G022800 Potri.018G034600 Potri.010G025800 Potri.001G038300 Potri.001G452200 Potri.010G017100 Potri.T007800 Potri.010G043900 Potri.002G129100 Potri.010G153400 Potri.008G166200 Potri.011G027500 Potri.018G083600 Potri.013G061800 Potri.008G137700 Potri.019G131800 Potri.013G059900 Potri.007G029500 Potri.003G186000 Potri.009G168600 Potri.011G047500 Potri.004G063500 Potri.002G193000 Potri.003G169100 Potri.001G441400 Potri.011G028600 Potri.007G011700 Potri.001G168000 Potri.012G123700 Potri.001G014100 Potri.011G037900 Potri.001G349900 Potri.005G056700 Potri.019G109400 Potri.003G149700 Potri.001G334900 Potri.004G060400 Potri.006G082500 Potri.005G082200 Potri.002G089800

			Potri.018G019800 Potri.T091200 Potri.008G106700 Potri.006G177500 Potri.019G099200 Potri.004G024900<br>Potri.015G141100 Potri.011G106400 Potri.005G214800 Potri.002G019300 Potri.001G411700 Potri.018G038100<br>Potri.011G035900 Potri.010G142900 Potri.005G167400 Potri.006G200600 Potri.007G131200 Potri.011G142100<br>Potri.006G126000 Potri.012G101000 Potri.005G119300 Potri.002G228200 Potri.004G060100 Potri.013G054000<br>Potri.014G090300 Potri.010G024900 Potri.005G240100 Potri.008G053100 Potri.006G074900 Potri.017G145200<br>Potri.017G050400 Potri.005G205400 Potri.016G056500 Potri.010G147700 Potri.005G181800 Potri.007G051000<br>Potri.006G241600 Potri.010G120600 Potri.005G215600 Potri.015G018000 Potri.018G001600 Potri.019G025500<br>Potri.004G025100 Potri.018G141700 Potri.005G139300 Potri.013G060000 Potri.014G019300 Potri.016G029900<br>Potri.012G108500 Potri.001G418100 Potri.016G123300 Potri.015G099200 Potri.009G016100 Potri.014G136400<br>Potri.014G195200 Potri.T136800 Potri.009G169900 Potri.004G106400 Potri.004G191900 Potri.011G039100<br>Potri.003G185700 Potri.004G025800 Potri.003G061700 Potri.016G069400 Potri.014G156400 Potri.002G228400<br>Potri.012G055500 Potri.012G043900 Potri.015G018200 Potri.013G148500 Potri.013G066700 Potri.001G051500<br>Potri.016G076100 Potri.018G094000 Potri.014G103000 Potri.006G138800 Potri.T064000 Potri.018G111700<br>Potri.001G256400 Potri.004G181900 Potri.013G114200 Potri.014G141000 Potri.011G129300 Potri.010G197800<br>Potri.002G226700 Potri.011G036400 Potri.005G070900 Potri.019G123500 Potri.012G071700 Potri.011G003900<br>Potri.012G042600 Potri.006G219300 Potri.010G181300 Potri.012G062300 Potri.010G078700 Potri.017G108800<br>Potri.011G129000 Potri.006G235500 Potri.005G093200 Potri.013G046100 Potri.003G143900 Potri.004G058500<br>Potri.003G150100 Potri.T032700 Potri.006G224100 Potri.006G105300 Potri.006G141500 Potri.T080600<br>Potri.004G066300 Potri.010G097700 Potri.001G154200 Potri.009G023600 Potri.010G129800 Potri.005G105000<br>Potri.004G025500 Potri.008G160200 Potri.003G079200 Potri.T132900 Potri.001G046100 Potri.010G103300<br>Potri.006G071600 Potri.001G046400 Potri.008G159100 Potri.016G059000 Potri.011G028800 Potri.011G034200<br>Potri.002G065600 Potri.018G114300 Potri.018G095100 Potri.015G018600 Potri.013G129800 Potri.016G068500<br>Potri.017G009500 Potri.011G157100 Potri.005G207300 Potri.005G130900 Potri.006G133300 Potri.001G092900<br>Potri.003G168800 Potri.T090100 Potri.001G099000 Potri.016G066700 Potri.003G132700 Potri.017G014200<br>Potri.012G126100 Potri.004G209300 Potri.019G006100 Potri.003G081200 Potri.004G117800 Potri.003G097300<br>Potri.018G088100 Potri.007G043800 Potri.002G004900 Potri.009G035500 Potri.019G084800 Potri.010G153500<br>Potri.004G051800 Potri.012G067600 Potri.015G122000 Potri.009G066100 Potri.010G224900 Potri.012G054700<br>Potri.011G112000 Potri.010G166200 Potri.011G169600 Potri.011G061700 Potri.008G035700 Potri.018G138700<br>Potri.002G240800 Potri.003G025800 Potri.008G068700 Potri.001G067600 Potri.002G093300 Potri.007G089000<br>Potri.009G119700 Potri.017G034500 Potri.010G177900 Potri.017G144500 Potri.005G037400 Potri.003G191100<br>Potri.001G343400 Potri.007G105000 Potri.010G025700 Potri.010G044100 Potri.011G075300 Potri.004G023900<br>Potri.005G257900 Potri.003G187000 Potri.002G043300 Potri.016G102500 Potri.001G026200 Potri.T043800<br>Potri.010G001500 Potri.005G214600 Potri.005G116200 Potri.018G109500 Potri.019G099300 Potri.005G251900<br>Potri.011G037300 Potri.004G208500 Potri.002G248900 Potri.T097300 Potri.016G045000 Potri.010G105700

			Potri.016G003400 Potri.001G325100 Potri.T059000 Potri.006G263600 Potri.016G018600 Potri.017G118100<br>Potri.010G237900 Potri.T075500 Potri.011G033900 Potri.006G273000 Potri.005G082000 Potri.007G127000<br>Potri.010G103100 Potri.001G286700 Potri.004G025200 Potri.014G052700 Potri.004G135300 Potri.015G092000<br>Potri.002G204400 Potri.011G123300 Potri.019G018100 Potri.013G042600 Potri.016G137900 Potri.003G105400<br>Potri.001G228200 Potri.018G141800 Potri.003G137000 Potri.012G047300 Potri.009G154100 Potri.006G175500<br>Potri.005G162800 Potri.007G012400 Potri.009G107200 Potri.002G181400 Potri.012G124100 Potri.006G251800<br>Potri.001G460600 Potri.006G068300 Potri.T021600 Potri.017G106900 Potri.008G089000 Potri.016G140200<br>Potri.T064400 Potri.014G141400 Potri.012G019900 Potri.005G197700 Potri.010G072300 Potri.001G352400<br>Potri.016G069500 Potri.015G030700 Potri.003G211700 Potri.007G045400 Potri.004G061900 Potri.010G100500<br>Potri.015G018100 Potri.008G116500 Potri.005G180200 Potri.006G225300 Potri.019G034700 Potri.001G045500<br>Potri.001G040000 Potri.008G080500 Potri.T128600 Potri.014G038600 Potri.002G173600 Potri.001G328000<br>Potri.013G158500 Potri.009G080600 Potri.003G211300 Potri.014G017200 Potri.004G014700 Potri.006G004300<br>Potri.006G103200 Potri.007G077600 Potri.016G011200 Potri.017G149000 Potri.015G031000 Potri.006G202600<br>Potri.007G135800 Potri.008G103300 Potri.010G083500 Potri.T133000 Potri.011G156100 Potri.018G141500<br>Potri.001G337400 Potri.006G202700 Potri.017G117700 Potri.007G019600 Potri.017G063500 Potri.018G063500<br>Potri.012G131900 Potri.004G233000 Potri.006G049200 Potri.T165400 Potri.T080500 Potri.002G129600<br>Potri.014G101100 Potri.008G059900 Potri.004G081300 Potri.004G056900 Potri.T021200 Potri.002G174500<br>Potri.006G186600 Potri.002G059100 Potri.008G159000 Potri.003G185800 Potri.017G084200 Potri.003G025600<br>Potri.006G058800 Potri.001G455500 Potri.010G018300 Potri.019G111100 Potri.T136500 Potri.002G186400<br>Potri.011G061800 Potri.001G410800 Potri.014G141900 Potri.005G236700 Potri.015G061600 Potri.002G198000<br>Potri.011G028300 Potri.007G079800 Potri.003G108200 Potri.019G007900 Potri.007G125000 Potri.001G058800<br>Potri.002G009400 Potri.012G031700 Potri.002G113600 Potri.004G007500 Potri.003G197500 Potri.007G011200<br>Potri.016G072300 Potri.001G073100 Potri.018G006000 Potri.005G195000 Potri.T023600 Potri.019G120000<br>Potri.T089800 Potri.007G136200 Potri.007G110600 Potri.011G035100 Potri.006G104200 Potri.001G100200<br>Potri.005G213200 Potri.004G097100 Potri.006G232100 Potri.015G064100 Potri.014G035500 Potri.004G024400<br>Potri.009G020700 Potri.010G043700 Potri.012G002800 Potri.019G005700 Potri.004G096800 Potri.001G412400<br>Potri.010G025500 Potri.003G135600 Potri.002G196200 Potri.017G110500 Potri.006G193000 Potri.011G038500<br>Potri.011G057000 Potri.004G023800 Potri.003G162500 Potri.005G072600 Potri.007G020100 Potri.001G316300<br>Potri.011G037100 Potri.004G040200 Potri.006G003400 Potri.T093800 Potri.019G049700 Potri.001G163700<br>Potri.T032200 Potri.003G003400 Potri.006G261900 Potri.004G226900 Potri.013G130000 Potri.015G111000<br>Potri.001G430700 Potri.017G100200 Potri.002G070500 Potri.T126700 Potri.004G027400 Potri.004G229800<br>Potri.002G060500 Potri.001G260800 Potri.012G011700 Potri.013G041000 Potri.001G228300 Potri.003G030900<br>Potri.009G118300 Potri.002G089900 Potri.017G034700 Potri.011G037700 Potri.006G148800 Potri.005G119200<br>Potri.011G145000 Potri.014G161300 Potri.004G024800 Potri.006G139700 Potri.005G098200 Potri.013G054200

			Potri.016G140300 Potri.002G046200 Potri.005G064100 Potri.002G021500 Potri.007G034500 Potri.011G029100 Potri.016G053800 Potri.019G072800 Potri.T008900 Potri.005G085200 Potri.001G104500 Potri.T011000 Potri.005G014700 Potri.004G023500 Potri.013G103300 Potri.005G073300 Potri.011G125000 Potri.009G154300 Potri.016G128300 Potri.T022600 Potri.013G090300 Potri.010G121100 Potri.018G148300 Potri.T080900 Potri.014G129200 Potri.015G103800 Potri.007G010300 Potri.002G004500 Potri.008G102800 Potri.017G036900 Potri.006G107600 Potri.004G191400 Potri.015G086800 Potri.011G056900 Potri.001G218800 Potri.013G123500 Potri.011G007800 Potri.005G128200 Potri.012G007500 Potri.014G155000 Potri.016G035700 Potri.001G020600 Potri.006G109100 Potri.002G090700 Potri.011G103700 Potri.004G155100 Potri.017G118400 Potri.011G051600 Potri.019G128600 Potri.004G073600 Potri.017G135000 Potri.012G113500 Potri.007G027000 Potri.017G007900 Potri.T128700 Potri.001G140700 Potri.011G058100 Potri.010G072400 Potri.008G116600 Potri.006G041100 Potri.017G152400 Potri.017G151400 Potri.008G040700 Potri.T009800 Potri.001G080900 Potri.011G149700 Potri.007G085700 Potri.013G135600 Potri.012G090500 Potri.001G233400 Potri.006G022000 Potri.004G083900 Potri.003G196600 Potri.010G234400 Potri.007G111100 Potri.009G167400 Potri.017G003200 Potri.017G079500 Potri.T096300 Potri.010G082000 Potri.017G117100 Potri.017G003800 Potri.T136400 Potri.006G052900 Potri.002G031900 Potri.018G134100 Potri.011G065900 Potri.T008700 Potri.011G058300 Potri.010G079200 Potri.009G141600 Potri.010G081100 Potri.011G034400 Potri.003G138600 Potri.015G066200 Potri.001G414200
GO:0044264	cellular polysaccharide metabolic process	21	Potri.002G204400 Potri.007G020100 Potri.006G004300 Potri.004G117800 Potri.018G095100 Potri.002G060500 Potri.007G131200 Potri.011G103700 Potri.009G066100 Potri.014G129200 Potri.002G173600 Potri.005G116200 Potri.003G097300 Potri.001G136200 Potri.019G049700 Potri.008G053100 Potri.015G066200 Potri.012G071700 Potri.006G022000 Potri.007G127000 Potri.009G168600
GO:0044267	cellular protein metabolic process	319	Potri.010G103100 Potri.006G193000 Potri.012G055500 Potri.004G025200 Potri.012G011300 Potri.014G052700 Potri.011G027500 Potri.019G078300 Potri.015G018200 Potri.004G023800 Potri.012G124100 Potri.010G079200 Potri.015G122000 Potri.001G410800 Potri.001G442200 Potri.019G018100 Potri.018G094000 Potri.003G105400 Potri.009G081800 Potri.007G020100 Potri.019G128600 Potri.T064000 Potri.010G015400 Potri.012G131900 Potri.002G004900 Potri.012G023300 Potri.014G038600 Potri.001G228200 Potri.013G133100 Potri.018G091000 Potri.014G141000 Potri.017G003100 Potri.003G150100 Potri.T093800 Potri.004G024400 Potri.003G137000 Potri.015G074200 Potri.015G061600 Potri.013G059900 Potri.T032200 Potri.002G198000 Potri.017G141300 Potri.010G025700 Potri.019G094200 Potri.009G154100 Potri.004G025100 Potri.T003400 Potri.011G128900 Potri.013G130000 Potri.011G035100 Potri.007G085300 Potri.002G070500 Potri.011G034300 Potri.012G071700 Potri.T022800 Potri.011G003900 Potri.T007800 Potri.005G251900 Potri.012G042600 Potri.006G219300 Potri.004G040200 Potri.017G009600 Potri.004G027400 Potri.019G008900 Potri.T021600 Potri.016G140200 Potri.010G078700 Potri.T064400 Potri.004G229800 Potri.012G019900 Potri.001G260800 Potri.003G085300 Potri.011G129000 Potri.011G169600 Potri.013G121000 Potri.011G028100 Potri.001G228300 Potri.016G069500 Potri.006G235500 Potri.015G030700 Potri.003G211700 Potri.016G011200 Potri.013G046100

			Potri.T084700 Potri.017G003800 Potri.019G084800 Potri.004G024600 Potri.011G037700 Potri.015G018100<br>Potri.T032700 Potri.002G075900 Potri.T108900 Potri.006G141500 Potri.004G024800 Potri.016G066700<br>Potri.004G096800 Potri.004G066300 Potri.010G097700 Potri.011G028800 Potri.003G066300 Potri.001G040000<br>Potri.010G025800 Potri.001G038300 Potri.005G214600 Potri.T128600 Potri.004G023500 Potri.011G037300<br>Potri.002G046200 Potri.005G072600 Potri.002G021500 Potri.002G129100 Potri.004G025500 Potri.007G034500<br>Potri.011G029100 Potri.015G031000 Potri.018G114300 Potri.016G053800 Potri.018G083600 Potri.T132900<br>Potri.004G097100 Potri.008G137700 Potri.019G131800 Potri.010G103300 Potri.007G039800 Potri.003G186000<br>Potri.009G168600 Potri.015G018600 Potri.007G127000 Potri.T011000 Potri.004G063500 Potri.005G014700<br>Potri.001G441400 Potri.011G034200 Potri.006G103200 Potri.007G077600 Potri.019G005300 Potri.013G103300<br>Potri.006G200600 Potri.003G025800 Potri.011G125000 Potri.011G028600 Potri.017G117100 Potri.001G168000<br>Potri.T022600 Potri.011G038500 Potri.017G009500 Potri.001G014100 Potri.018G111700 Potri.011G037900<br>Potri.007G011700 Potri.005G056700 Potri.019G109400 Potri.010G083500 Potri.T133000 Potri.017G034700<br>Potri.018G148300 Potri.001G349900 Potri.005G130900 Potri.T080900 Potri.006G133300 Potri.005G082200<br>Potri.002G089800 Potri.T090100 Potri.004G058500 Potri.T091200 Potri.009G020700 Potri.006G202700<br>Potri.017G117700 Potri.006G107600 Potri.010G025500 Potri.011G058100 Potri.019G099200 Potri.004G191400<br>Potri.015G086800 Potri.004G024900 Potri.004G209300 Potri.019G006100 Potri.004G233000 Potri.004G060100<br>Potri.006G052900 Potri.001G430700 Potri.003G025600 Potri.011G036400 Potri.011G106400 Potri.004G061900<br>Potri.T080500 Potri.014G164100 Potri.010G121100 Potri.002G019300 Potri.001G411700 Potri.014G101100<br>Potri.008G059900 Potri.009G035500 Potri.014G155000 Potri.011G035900 Potri.010G017100 Potri.012G067600<br>Potri.T021200 Potri.T165400 Potri.009G066100 Potri.011G142100 Potri.004G155100 Potri.004G226900<br>Potri.006G126000 Potri.002G174500 Potri.007G027000 Potri.010G224900 Potri.011G129300 Potri.012G054700<br>Potri.004G073600 Potri.017G135000 Potri.017G084200 Potri.017G118400 Potri.001G455500 Potri.010G018300<br>Potri.011G112000 Potri.T136500 Potri.T128700 Potri.009G167400 Potri.010G024900 Potri.005G240100<br>Potri.006G225300 Potri.008G035700 Potri.018G138700 Potri.004G191900 Potri.017G145200 Potri.017G007900<br>Potri.T080600 Potri.003G185800 Potri.008G068700 Potri.019G005700 Potri.011G028300 Potri.003G108200<br>Potri.019G007900 Potri.005G113600 Potri.007G125000 Potri.017G034500 Potri.002G009400 Potri.010G177900<br>Potri.007G135800 Potri.005G181800 Potri.017G152400 Potri.007G051000 Potri.017G151400 Potri.001G286700<br>Potri.T009800 Potri.006G261900 Potri.005G037400 Potri.003G191100 Potri.015G018000 Potri.011G039100<br>Potri.004G014700 Potri.018G034600 Potri.019G025500 Potri.005G139300 Potri.002G228200 Potri.011G075300<br>Potri.012G090500 Potri.004G023900 Potri.005G128200 Potri.019G111100 Potri.013G060000 Potri.T089800<br>Potri.T136800 Potri.016G102500 Potri.003G211300 Potri.001G026200 Potri.016G140300 Potri.007G110600<br>Potri.010G001500 Potri.004G025800 Potri.007G111100 Potri.001G418100 Potri.006G202600 Potri.011G037100<br>Potri.T126700 Potri.016G123300 Potri.019G120000 Potri.009G016100 Potri.005G213200 Potri.T023600<br>Potri.010G043900 Potri.002G248900 Potri.014G035500 Potri.T008900 Potri.T090000 Potri.016G029900

			Potri.009G154300 Potri.T097300 Potri.T136400 Potri.014G136400 Potri.014G195200 Potri.016G045000 Potri.016G003400 Potri.018G088100 Potri.010G043700 Potri.003G196600 Potri.016G035700 Potri.012G002800 Potri.018G134100 Potri.004G083900 Potri.013G114200 Potri.007G136200 Potri.T008700 Potri.017G118100 Potri.011G058300 Potri.T075500 Potri.003G185700 Potri.001G412400 Potri.008G160200 Potri.011G034400 Potri.017G003200 Potri.011G033900 Potri.015G066200 Potri.006G139700 Potri.006G273000 Potri.016G069400 Potri.014G156400 Potri.001G414200
GO:0006732	coenzyme metabolic process	11	Potri.007G045400 Potri.004G208500 Potri.011G145000 Potri.014G161300 Potri.009G169900 Potri.010G197800 Potri.002G240800 Potri.009G080600 Potri.017G106900 Potri.001G104500 Potri.003G135600
GO:0032502	developmental process	9	Potri.010G206600 Potri.012G017600 Potri.001G026200 Potri.003G143600 Potri.008G054000 Potri.010G205700 Potri.001G087500 Potri.008G054600 Potri.001G042600
GO:0006629	lipid metabolic process	69	Potri.002G083800 Potri.017G134600 Potri.017G110500 Potri.005G177700 Potri.001G263200 Potri.003G079200 Potri.017G102100 Potri.017G133800 Potri.003G213700 Potri.002G033600 Potri.001G045500 Potri.001G051500 Potri.003G081500 Potri.013G123500 Potri.006G003400 Potri.003G063100 Potri.007G100600 Potri.006G022000 Potri.009G168600 Potri.002G204400 Potri.007G020100 Potri.001G046100 Potri.001G252100 Potri.001G256400 Potri.015G069600 Potri.010G245200 Potri.014G027500 Potri.009G066100 Potri.014G033200 Potri.007G131200 Potri.011G103700 Potri.T143600 Potri.019G072800 Potri.005G068700 Potri.T004800 Potri.014G033900 Potri.003G003400 Potri.001G100200 Potri.001G046400 Potri.009G118300 Potri.003G071400 Potri.017G134200 Potri.018G063900 Potri.T004600 Potri.006G149700 Potri.009G057900 Potri.017G134100 Potri.012G071700 Potri.003G061700 Potri.006G177500 Potri.008G053100 Potri.014G033000 Potri.005G167400 Potri.016G015700 Potri.009G086100 Potri.017G133900 Potri.011G156100 Potri.007G089000 Potri.002G044700 Potri.T168600 Potri.010G237900 Potri.014G129200 Potri.010G236800 Potri.001G140700 Potri.002G004500 Potri.007G127000 Potri.015G066200 Potri.009G107200 Potri.T095600
GO:0043170	macromolecule metabolic process	527	Potri.001G404100 Potri.012G011300 Potri.019G078300 Potri.019G008900 Potri.009G081800 Potri.019G005300 Potri.001G077900 Potri.010G015400 Potri.003G149700 Potri.012G023300 Potri.013G133100 Potri.018G091000 Potri.008G174100 Potri.017G003100 Potri.015G074200 Potri.007G039800 Potri.002G152600 Potri.004G112300 Potri.017G141300 Potri.018G036600 Potri.019G094200 Potri.004G038000 Potri.011G151200 Potri.T003400 Potri.011G128900 Potri.007G085300 Potri.011G034300 Potri.002G113400 Potri.010G192100 Potri.001G136200 Potri.001G442200 Potri.002G186600 Potri.017G009600 Potri.017G055400 Potri.T090000 Potri.002G075900 Potri.003G085300 Potri.013G121000 Potri.011G028100 Potri.018G019700 Potri.001G114000 Potri.014G096200 Potri.002G009400 Potri.002G168700 Potri.004G024600 Potri.003G066300 Potri.T108900 Potri.T022800 Potri.005G113600 Potri.011G028800 Potri.018G034600 Potri.010G025800 Potri.001G038300 Potri.001G452200 Potri.010G017100 Potri.T007800 Potri.010G043900 Potri.002G129100 Potri.008G166200 Potri.011G027500 Potri.018G083600 Potri.008G137700 Potri.019G131800 Potri.013G059900 Potri.007G029500 Potri.003G186000 Potri.017G106900 Potri.011G047500 Potri.004G063500 Potri.002G193000 Potri.003G169100

			Potri.001G441400 Potri.011G028600 Potri.007G011700 Potri.001G168000 Potri.001G014100 Potri.011G037900<br>Potri.001G349900 Potri.005G056700 Potri.019G109400 Potri.004G060400 Potri.005G082200 Potri.002G089800<br>Potri.018G019800 Potri.T091200 Potri.008G106700 Potri.019G099200 Potri.004G024900 Potri.015G141100<br>Potri.011G106400 Potri.005G214800 Potri.014G164100 Potri.002G019300 Potri.001G411700 Potri.018G038100<br>Potri.011G035900 Potri.010G142900 Potri.005G207300 Potri.006G200600 Potri.007G131200 Potri.011G142100<br>Potri.006G126000 Potri.012G101000 Potri.005G119300 Potri.002G228200 Potri.004G060100 Potri.013G054000<br>Potri.T097300 Potri.010G024900 Potri.005G240100 Potri.008G053100 Potri.006G074900 Potri.017G145200<br>Potri.008G027500 Potri.009G167400 Potri.015G103900 Potri.005G205400 Potri.016G056500 Potri.010G147700<br>Potri.005G181800 Potri.007G051000 Potri.015G018000 Potri.019G025500 Potri.018G141700 Potri.013G135600<br>Potri.005G139300 Potri.T175200 Potri.013G060000 Potri.T136800 Potri.016G029900 Potri.012G108500<br>Potri.001G157500 Potri.001G418100 Potri.005G179900 Potri.002G054900 Potri.016G123300 Potri.015G099200<br>Potri.009G016100 Potri.004G037800 Potri.014G136400 Potri.005G130900 Potri.016G066700 Potri.004G191900<br>Potri.010G025700 Potri.003G185700 Potri.004G025800 Potri.001G325100 Potri.016G069400 Potri.014G156400<br>Potri.002G228400 Potri.012G055500 Potri.015G018200 Potri.013G066700 Potri.016G076100 Potri.018G094000<br>Potri.014G103000 Potri.006G138800 Potri.T064000 Potri.018G111700 Potri.019G006100 Potri.004G181900<br>Potri.013G114200 Potri.014G141000 Potri.006G050700 Potri.011G129300 Potri.003G025600 Potri.002G226700<br>Potri.011G036400 Potri.019G123500 Potri.012G071700 Potri.011G003900 Potri.012G042600 Potri.006G219300<br>Potri.010G181300 Potri.012G062300 Potri.010G078700 Potri.011G129000 Potri.006G235500 Potri.013G046100<br>Potri.004G058500 Potri.003G150100 Potri.T032700 Potri.006G224100 Potri.006G105300 Potri.006G141500<br>Potri.T080600 Potri.004G066300 Potri.010G097700 Potri.001G154200 Potri.009G023600 Potri.005G105000<br>Potri.004G025500 Potri.003G162500 Potri.T132900 Potri.010G103300 Potri.006G071600 Potri.013G125000<br>Potri.002G114500 Potri.016G059000 Potri.015G110000 Potri.011G034200 Potri.002G065600 Potri.018G114300<br>Potri.018G095100 Potri.015G018600 Potri.013G129800 Potri.017G009500 Potri.011G157100 Potri.007G072300<br>Potri.014G195200 Potri.006G133300 Potri.001G092900 Potri.T090100 Potri.001G099000 Potri.003G132700<br>Potri.004G209300 Potri.017G148200 Potri.003G081200 Potri.004G117800 Potri.003G097300 Potri.018G088100<br>Potri.007G043800 Potri.002G004900 Potri.009G035500 Potri.019G084800 Potri.005G247300 Potri.004G051800<br>Potri.012G067600 Potri.015G122000 Potri.009G066100 Potri.010G224900 Potri.012G054700 Potri.011G112000<br>Potri.010G166200 Potri.011G169600 Potri.011G061700 Potri.008G035700 Potri.018G138700 Potri.019G064300<br>Potri.003G025800 Potri.008G068700 Potri.001G067600 Potri.002G013900 Potri.002G093300 Potri.009G119700<br>Potri.017G034500 Potri.010G177900 Potri.005G037400 Potri.003G191100 Potri.007G105000 Potri.011G039100<br>Potri.012G091100 Potri.011G075300 Potri.004G023900 Potri.005G257900 Potri.002G043300 Potri.016G102500<br>Potri.001G026200 Potri.T043800 Potri.010G001500 Potri.005G214600 Potri.011G037100 Potri.018G109500<br>Potri.019G099300 Potri.T126700 Potri.011G037300 Potri.002G248900 Potri.013G103300 Potri.016G045000<br>Potri.010G105700 Potri.016G035700 Potri.006G263600 Potri.016G018600 Potri.017G118100 Potri.T075500

			Potri.011G033900 Potri.006G273000 Potri.005G082000 Potri.016G003400 Potri.010G103100 Potri.001G286700
			Potri.004G025200 Potri.014G052700 Potri.002G204400 Potri.011G123300 Potri.019G018100 Potri.013G042600
			Potri.016G137900 Potri.003G105400 Potri.001G228200 Potri.018G141800 Potri.003G137000 Potri.005G180200
			Potri.012G047300 Potri.009G154100 Potri.004G025100 Potri.007G012400 Potri.002G181400 Potri.012G124100
			Potri.006G251800 Potri.001G460600 Potri.T021600 Potri.008G089000 Potri.016G140200 Potri.T064400
			Potri.014G141400 Potri.012G019900 Potri.007G102100 Potri.010G072300 Potri.001G352400 Potri.016G069500
			Potri.015G030700 Potri.003G211700 Potri.004G061900 Potri.015G018100 Potri.019G093800 Potri.006G225300
			Potri.019G034700 Potri.001G040000 Potri.T128600 Potri.014G038600 Potri.002G173600 Potri.001G328000
			Potri.013G158500 Potri.009G080600 Potri.003G211300 Potri.004G014700 Potri.006G004300 Potri.006G103200
			Potri.007G077600 Potri.005G095600 Potri.016G011200 Potri.017G149000 Potri.015G031000 Potri.006G202600
			Potri.007G135800 Potri.008G103300 Potri.010G083500 Potri.T133000 Potri.018G141500 Potri.006G202700
			Potri.017G117700 Potri.010G025500 Potri.012G131900 Potri.004G233000 Potri.006G049200 Potri.T165400
			Potri.T080500 Potri.002G129600 Potri.014G101100 Potri.008G059900 Potri.004G056900 Potri.T021200
			Potri.009G168600 Potri.006G186600 Potri.002G059100 Potri.012G112200 Potri.017G084200 Potri.006G058800
			Potri.001G455500 Potri.010G018300 Potri.019G111100 Potri.T136500 Potri.002G186400 Potri.011G061800
			Potri.001G410800 Potri.005G236700 Potri.015G061600 Potri.002G198000 Potri.011G028300 Potri.007G079800
			Potri.003G108200 Potri.019G007900 Potri.007G125000 Potri.001G058800 Potri.T084700 Potri.012G031700
			Potri.004G007500 Potri.003G197500 Potri.016G072300 Potri.T023600 Potri.019G120000 Potri.T089800
			Potri.007G136200 Potri.007G110600 Potri.006G104200 Potri.003G185800 Potri.005G213200 Potri.004G097100
			Potri.015G064100 Potri.014G035500 Potri.004G024400 Potri.009G020700 Potri.010G043700 Potri.012G002800
			Potri.019G005700 Potri.001G412400 Potri.007G019600 Potri.003G135600 Potri.002G196200 Potri.006G193000
			Potri.011G038500 Potri.011G057000 Potri.004G023800 Potri.004G096800 Potri.005G072600 Potri.007G020100
			Potri.001G316300 Potri.005G116200 Potri.004G040200 Potri.T093800 Potri.019G049700 Potri.001G163700
			Potri.T032200 Potri.006G261900 Potri.004G226900 Potri.013G130000 Potri.011G035100 Potri.001G430700
			Potri.017G100200 Potri.002G070500 Potri.014G111800 Potri.005G251900 Potri.004G027400 Potri.013G041000
			Potri.002G060500 Potri.001G260800 Potri.004G229800 Potri.001G228300 Potri.012G104600 Potri.002G089900
			Potri.017G034700 Potri.011G037700 Potri.006G148800 Potri.005G119200 Potri.011G051600 Potri.014G161300
			Potri.004G024800 Potri.006G139700 Potri.005G098200 Potri.013G054200 Potri.016G140300 Potri.002G046200
			Potri.005G064100 Potri.002G021500 Potri.007G034500 Potri.011G029100 Potri.016G053800 Potri.T008900
			Potri.005G085200 Potri.007G127000 Potri.T011000 Potri.005G014700 Potri.004G023500 Potri.011G125000
			Potri.009G154300 Potri.016G128300 Potri.T022600 Potri.013G090300 Potri.010G121100 Potri.018G148300
			Potri.T080900 Potri.014G129200 Potri.007G010300 Potri.002G174500 Potri.006G107600 Potri.004G191400
			Potri.015G086800 Potri.011G056900 Potri.001G218800 Potri.011G007800 Potri.005G128200 Potri.012G007500
			Potri.014G155000 Potri.016G068500 Potri.006G109100 Potri.002G090700 Potri.011G103700 Potri.004G155100

			Potri.017G118400 Potri.019G128600 Potri.004G073600 Potri.017G135000 Potri.007G027000 Potri.017G007900 Potri.T128700 Potri.011G058100 Potri.010G072400 Potri.008G116600 Potri.005G204600 Potri.006G041100 Potri.017G152400 Potri.017G151400 Potri.T009800 Potri.001G080900 Potri.011G149700 Potri.007G085700 Potri.008G203200 Potri.013G033200 Potri.012G090500 Potri.005G195000 Potri.006G022000 Potri.004G083900 Potri.003G196600 Potri.010G234400 Potri.007G111100 Potri.014G090300 Potri.017G003200 Potri.017G079500 Potri.010G082000 Potri.017G117100 Potri.017G003800 Potri.T136400 Potri.006G052900 Potri.002G031900 Potri.018G134100 Potri.011G065900 Potri.T008700 Potri.011G058300 Potri.010G079200 Potri.009G141600 Potri.008G160200 Potri.011G034400 Potri.003G138600 Potri.019G093700 Potri.015G066200 Potri.001G414200
GO:0051704	multi-organism process	49	Potri.010G025700 Potri.T022800 Potri.001G410800 Potri.T023600 Potri.011G034200 Potri.011G129000 Potri.011G037900 Potri.010G017100 Potri.011G035900 Potri.013G060000 Potri.010G025800 Potri.010G015400 Potri.T021200 Potri.001G409400 Potri.001G228200 Potri.016G053800 Potri.007G111100 Potri.001G418100 Potri.019G120000 Potri.011G037100 Potri.011G129300 Potri.010G018100 Potri.013G059900 Potri.001G411700 Potri.012G047300 Potri.011G037300 Potri.005G014700 Potri.010G018300 Potri.011G128900 Potri.011G035100 Potri.011G125000 Potri.011G034300 Potri.011G036500 Potri.011G036400 Potri.T022600 Potri.016G102500 Potri.011G035300 Potri.005G056700 Potri.004G027400 Potri.T021600 Potri.011G039100 Potri.001G412400 Potri.013G121000 Potri.011G034400 Potri.010G103300 Potri.010G017800 Potri.011G034500 Potri.010G025500 Potri.001G414200
GO:0055114	oxidation reduction	204	Potri.008G099100 Potri.016G125000 Potri.019G088500 Potri.013G102700 Potri.001G268600 Potri.001G335900 Potri.009G169900 Potri.010G088800 Potri.011G162900 Potri.002G033600 Potri.001G113900 Potri.011G071100 Potri.001G331100 Potri.005G084500 Potri.019G130700 Potri.013G083600 Potri.001G463300 Potri.002G004500 Potri.001G046100 Potri.001G256400 Potri.006G128900 Potri.011G047200 Potri.005G146700 Potri.001G015300 Potri.T168600 Potri.001G335800 Potri.010G097800 Potri.003G159800 Potri.011G158100 Potri.001G422500 Potri.008G106400 Potri.010G168200 Potri.015G111000 Potri.011G159900 Potri.017G100200 Potri.006G022200 Potri.003G067700 Potri.012G038400 Potri.006G101800 Potri.016G058200 Potri.008G205200 Potri.001G105200 Potri.001G270900 Potri.001G307500 Potri.016G132700 Potri.001G334700 Potri.017G110500 Potri.009G107600 Potri.002G204400 Potri.002G114500 Potri.010G107500 Potri.012G096800 Potri.005G172400 Potri.011G150100 Potri.009G062800 Potri.011G162800 Potri.011G145000 Potri.001G330500 Potri.018G075000 Potri.004G149000 Potri.002G055300 Potri.001G045500 Potri.T127100 Potri.018G121700 Potri.002G130700 Potri.007G084700 Potri.008G080500 Potri.009G108600 Potri.014G193800 Potri.001G461700 Potri.011G157900 Potri.016G132900 Potri.003G079200 Potri.002G021000 Potri.007G074300 Potri.013G045000 Potri.002G088600 Potri.001G452600 Potri.009G080600 Potri.017G106900 Potri.001G046400 Potri.009G118300 Potri.011G020900 Potri.012G011700 Potri.018G046600 Potri.011G042500 Potri.001G337400 Potri.006G177500 Potri.010G049200 Potri.003G103900 Potri.006G151600 Potri.001G167700 Potri.005G207300 Potri.012G006300 Potri.009G043700 Potri.013G064200 Potri.009G033900 Potri.011G156100 Potri.011G161600 Potri.001G464800 Potri.014G020700

			Potri.014G129200 Potri.007G074900 Potri.001G263400 Potri.001G462200 Potri.015G002800 Potri.006G236500 Potri.001G070900 Potri.T045500 Potri.014G037400 Potri.010G073200 Potri.014G022800 Potri.005G113700 Potri.013G106200 Potri.005G200500 Potri.001G430700 Potri.004G034100 Potri.009G065000 Potri.011G047300 Potri.001G229500 Potri.013G123500 Potri.T079500 Potri.003G133300 Potri.005G113900 Potri.019G003200 Potri.005G220700 Potri.008G053100 Potri.007G115500 Potri.018G065600 Potri.007G131200 Potri.011G103700 Potri.011G155900 Potri.003G210900 Potri.005G043400 Potri.016G117100 Potri.003G173500 Potri.007G108700 Potri.012G113500 Potri.016G102100 Potri.014G161300 Potri.001G462600 Potri.005G144000 Potri.005G135300 Potri.008G179300 Potri.001G140700 Potri.005G064400 Potri.018G149300 Potri.017G092000 Potri.019G057900 Potri.001G461800 Potri.003G214900 Potri.001G121000 Potri.014G122800 Potri.005G026200 Potri.T030600 Potri.017G064100 Potri.001G459100 Potri.017G144500 Potri.001G190100 Potri.009G145400 Potri.018G146100 Potri.015G086000 Potri.001G461500 Potri.014G038000 Potri.012G091100 Potri.011G158800 Potri.006G022000 Potri.013G103000 Potri.001G382400 Potri.002G234000 Potri.001G249700 Potri.005G032400 Potri.007G126600 Potri.011G150300 Potri.001G270400 Potri.006G090600 Potri.004G075800 Potri.016G078300 Potri.001G011500 Potri.001G100200 Potri.006G101200 Potri.017G004600 Potri.001G462400 Potri.013G154400 Potri.001G451900 Potri.005G079400 Potri.002G013400 Potri.001G003100 Potri.T059000 Potri.010G183500 Potri.004G106400 Potri.013G076500 Potri.001G462000 Potri.005G143900 Potri.012G089900 Potri.009G064900 Potri.002G189900 Potri.014G143200 Potri.001G270800 Potri.004G208500 Potri.015G003500 Potri.013G160800 Potri.011G158500 Potri.003G135600
GO:0006793	phosphorus metabolic process	285	Potri.006G193000 Potri.012G055500 Potri.004G025200 Potri.012G011300 Potri.014G052700 Potri.019G078300 Potri.015G018200 Potri.013G148500 Potri.004G023800 Potri.004G096800 Potri.015G122000 Potri.001G410800 Potri.001G442200 Potri.019G018100 Potri.005G072600 Potri.003G105400 Potri.009G081800 Potri.007G020100 Potri.019G128600 Potri.T064000 Potri.010G015400 Potri.012G131900 Potri.002G004900 Potri.012G023300 Potri.014G038600 Potri.001G228200 Potri.013G133100 Potri.018G091000 Potri.014G164100 Potri.017G003100 Potri.003G150100 Potri.T093800 Potri.004G024400 Potri.003G137000 Potri.011G129300 Potri.015G061600 Potri.013G059900 Potri.T032200 Potri.017G141300 Potri.010G025700 Potri.019G094200 Potri.009G154100 Potri.004G025100 Potri.T003400 Potri.011G128900 Potri.005G070900 Potri.013G130000 Potri.011G035100 Potri.007G085300 Potri.011G034300 Potri.012G071700 Potri.T022800 Potri.011G003900 Potri.T007800 Potri.005G251900 Potri.012G124100 Potri.006G219300 Potri.004G040200 Potri.017G009600 Potri.004G027400 Potri.019G008900 Potri.T021600 Potri.016G140200 Potri.010G078700 Potri.T064400 Potri.T090000 Potri.004G058500 Potri.001G260800 Potri.003G085300 Potri.011G129000 Potri.011G169600 Potri.013G121000 Potri.011G028100 Potri.001G228300 Potri.003G030900 Potri.006G235500 Potri.015G030700 Potri.003G211700 Potri.016G011200 Potri.013G046100 Potri.T084700 Potri.017G003800 Potri.019G084800 Potri.004G024600 Potri.011G037700 Potri.015G018100 Potri.T032700 Potri.002G075900 Potri.T108900 Potri.006G141500 Potri.004G024800 Potri.T080600 Potri.004G066300 Potri.010G097700 Potri.011G028800

			Potri.003G066300 Potri.001G040000 Potri.010G025800 Potri.001G038300 Potri.005G214600 Potri.T128600 Potri.004G023500 Potri.011G037300 Potri.010G043900 Potri.002G129100 Potri.004G025500 Potri.007G034500 Potri.011G029100 Potri.018G114300 Potri.016G053800 Potri.T132900 Potri.004G097100 Potri.019G131800 Potri.010G103300 Potri.007G039800 Potri.003G186000 Potri.009G168600 Potri.007G127000 Potri.T011000 Potri.004G063500 Potri.005G014700 Potri.001G441400 Potri.011G034200 Potri.006G103200 Potri.007G077600 Potri.019G005300 Potri.013G103300 Potri.006G200600 Potri.015G018600 Potri.011G125000 Potri.011G028600 Potri.017G117100 Potri.001G168000 Potri.T022600 Potri.011G038500 Potri.017G009500 Potri.001G014100 Potri.018G111700 Potri.011G037900 Potri.007G011700 Potri.005G056700 Potri.019G109400 Potri.010G083500 Potri.T133000 Potri.017G034700 Potri.018G148300 Potri.001G349900 Potri.005G130900 Potri.T080900 Potri.006G082500 Potri.006G133300 Potri.005G082200 Potri.T090100 Potri.016G066700 Potri.T091200 Potri.009G020700 Potri.008G102800 Potri.017G117700 Potri.010G025500 Potri.011G058100 Potri.019G099200 Potri.004G191400 Potri.013G114200 Potri.004G024900 Potri.004G209300 Potri.019G006100 Potri.004G233000 Potri.004G060100 Potri.006G052900 Potri.003G025600 Potri.011G036400 Potri.011G106400 Potri.004G061900 Potri.T080500 Potri.011G027500 Potri.010G121100 Potri.002G019300 Potri.001G411700 Potri.005G128200 Potri.008G059900 Potri.009G035500 Potri.014G155000 Potri.011G035900 Potri.010G017100 Potri.012G067600 Potri.T021200 Potri.009G066100 Potri.011G142100 Potri.004G155100 Potri.004G226900 Potri.006G126000 Potri.007G027000 Potri.010G224900 Potri.012G054700 Potri.015G018000 Potri.017G118400 Potri.001G455500 Potri.010G018300 Potri.011G112000 Potri.T136500 Potri.T128700 Potri.009G167400 Potri.006G225300 Potri.018G138700 Potri.004G191900 Potri.017G145200 Potri.017G007900 Potri.003G025800 Potri.003G185800 Potri.008G068700 Potri.011G028300 Potri.003G108200 Potri.019G007900 Potri.005G113600 Potri.007G125000 Potri.017G034500 Potri.002G009400 Potri.011G058300 Potri.010G177900 Potri.005G181800 Potri.017G152400 Potri.017G151400 Potri.T009800 Potri.006G261900 Potri.005G037400 Potri.015G086800 Potri.001G343400 Potri.011G039100 Potri.004G014700 Potri.010G044100 Potri.019G025500 Potri.005G139300 Potri.002G228200 Potri.011G075300 Potri.012G090500 Potri.004G023900 Potri.019G111100 Potri.013G060000 Potri.T089800 Potri.T136800 Potri.016G102500 Potri.003G211300 Potri.016G140300 Potri.010G001500 Potri.004G025800 Potri.007G111100 Potri.001G418100 Potri.011G037100 Potri.T126700 Potri.016G123300 Potri.019G120000 Potri.005G213200 Potri.T023600 Potri.002G248900 Potri.014G035500 Potri.T008900 Potri.016G029900 Potri.009G154300 Potri.T097300 Potri.T136400 Potri.014G136400 Potri.014G195200 Potri.016G045000 Potri.018G088100 Potri.010G043700 Potri.003G196600 Potri.016G035700 Potri.012G002800 Potri.018G134100 Potri.019G005700 Potri.T008700 Potri.017G118100 Potri.010G237900 Potri.T075500 Potri.003G185700 Potri.001G412400 Potri.008G160200 Potri.011G034400 Potri.017G003200 Potri.011G033900 Potri.015G066200 Potri.006G139700 Potri.006G273000 Potri.014G156400 Potri.001G414200
GO:0016310	phosphorylation	282	Potri.006G193000 Potri.012G055500 Potri.004G025200 Potri.012G011300 Potri.014G052700 Potri.019G078300 Potri.015G018200 Potri.013G148500 Potri.004G023800 Potri.004G096800 Potri.015G122000

			Potri.001G410800 Potri.001G442200 Potri.019G018100 Potri.005G072600 Potri.003G105400 Potri.009G081800<br>Potri.007G020100 Potri.019G128600 Potri.T064000 Potri.010G015400 Potri.012G131900 Potri.002G004900<br>Potri.012G023300 Potri.014G038600 Potri.001G228200 Potri.013G133100 Potri.018G091000 Potri.014G164100<br>Potri.017G003100 Potri.003G150100 Potri.T093800 Potri.004G024400 Potri.003G137000 Potri.011G129300<br>Potri.015G061600 Potri.013G059900 Potri.T032200 Potri.017G141300 Potri.010G025700 Potri.019G094200<br>Potri.009G154100 Potri.004G025100 Potri.T003400 Potri.011G128900 Potri.005G070900 Potri.013G130000<br>Potri.011G035100 Potri.007G085300 Potri.011G034300 Potri.012G071700 Potri.T022800 Potri.011G003900<br>Potri.T007800 Potri.005G251900 Potri.012G124100 Potri.006G219300 Potri.004G040200 Potri.017G009600<br>Potri.004G027400 Potri.019G008900 Potri.T021600 Potri.016G140200 Potri.010G078700 Potri.T064400<br>Potri.T090000 Potri.004G058500 Potri.001G260800 Potri.003G085300 Potri.011G129000 Potri.011G169600<br>Potri.013G121000 Potri.011G028100 Potri.001G228300 Potri.006G235500 Potri.015G030700 Potri.003G211700<br>Potri.016G011200 Potri.013G046100 Potri.T084700 Potri.017G003800 Potri.019G084800 Potri.004G024600<br>Potri.011G037700 Potri.015G018100 Potri.T032700 Potri.002G075900 Potri.T108900 Potri.006G141500<br>Potri.004G024800 Potri.T080600 Potri.004G066300 Potri.010G097700 Potri.011G028800 Potri.003G066300<br>Potri.001G040000 Potri.010G025800 Potri.001G038300 Potri.005G214600 Potri.T128600 Potri.004G023500<br>Potri.011G037300 Potri.010G043900 Potri.002G129100 Potri.004G025500 Potri.007G034500 Potri.011G029100<br>Potri.018G114300 Potri.016G053800 Potri.T132900 Potri.004G097100 Potri.019G131800 Potri.010G103300<br>Potri.007G039800 Potri.003G186000 Potri.009G168600 Potri.007G127000 Potri.T011000 Potri.004G063500<br>Potri.005G014700 Potri.001G441400 Potri.011G034200 Potri.006G103200 Potri.007G077600 Potri.019G005300<br>Potri.013G103300 Potri.006G200600 Potri.015G018600 Potri.011G125000 Potri.011G028600 Potri.017G117100<br>Potri.001G168000 Potri.T022600 Potri.011G038500 Potri.017G009500 Potri.001G014100 Potri.018G111700<br>Potri.011G037900 Potri.007G011700 Potri.005G056700 Potri.019G109400 Potri.010G083500 Potri.T133000<br>Potri.017G034700 Potri.018G148300 Potri.001G349900 Potri.005G130900 Potri.T080900 Potri.006G133300<br>Potri.005G082200 Potri.T090100 Potri.016G066700 Potri.T091200 Potri.009G020700 Potri.008G102800<br>Potri.017G117700 Potri.010G025500 Potri.011G058100 Potri.019G099200 Potri.004G191400 Potri.013G114200<br>Potri.004G024900 Potri.004G209300 Potri.019G006100 Potri.004G233000 Potri.004G060100 Potri.006G052900<br>Potri.003G025600 Potri.011G036400 Potri.011G106400 Potri.004G061900 Potri.T080500 Potri.011G027500<br>Potri.010G121100 Potri.002G019300 Potri.001G411700 Potri.005G128200 Potri.008G059900 Potri.009G035500<br>Potri.014G155000 Potri.011G035900 Potri.010G017100 Potri.012G067600 Potri.T021200 Potri.009G066100<br>Potri.011G142100 Potri.004G155100 Potri.004G226900 Potri.006G126000 Potri.007G027000 Potri.010G224900<br>Potri.012G054700 Potri.015G018000 Potri.017G118400 Potri.001G455500 Potri.010G018300 Potri.011G112000<br>Potri.T136500 Potri.T128700 Potri.009G167400 Potri.006G225300 Potri.018G138700 Potri.004G191900<br>Potri.017G145200 Potri.017G007900 Potri.003G025800 Potri.003G185800 Potri.008G068700 Potri.011G028300<br>Potri.003G108200 Potri.019G007900 Potri.005G113600 Potri.007G125000 Potri.017G034500 Potri.002G009400

			Potri.011G058300 Potri.010G177900 Potri.005G181800 Potri.017G152400 Potri.017G151400 Potri.T009800 Potri.006G261900 Potri.005G037400 Potri.015G086800 Potri.001G343400 Potri.011G039100 Potri.004G014700 Potri.010G044100 Potri.019G025500 Potri.005G139300 Potri.002G228200 Potri.011G075300 Potri.012G090500 Potri.004G023900 Potri.019G111100 Potri.013G060000 Potri.T089800 Potri.T136800 Potri.016G102500 Potri.003G211300 Potri.016G140300 Potri.010G001500 Potri.004G025800 Potri.007G111100 Potri.001G418100 Potri.011G037100 Potri.T126700 Potri.016G123300 Potri.019G120000 Potri.005G213200 Potri.T023600 Potri.002G248900 Potri.014G035500 Potri.T008900 Potri.016G029900 Potri.009G154300 Potri.T097300 Potri.T136400 Potri.014G136400 Potri.014G195200 Potri.016G045000 Potri.018G088100 Potri.010G043700 Potri.003G196600 Potri.012G002800 Potri.018G134100 Potri.019G005700 Potri.T008700 Potri.017G118100 Potri.010G237900 Potri.T075500 Potri.003G185700 Potri.001G412400 Potri.008G160200 Potri.011G034400 Potri.017G003200 Potri.011G033900 Potri.015G066200 Potri.006G139700 Potri.006G273000 Potri.014G156400 Potri.001G414200
GO:0005976	polysaccharide metabolic process	27	Potri.019G093800 Potri.004G117800 Potri.002G204400 Potri.002G173600 Potri.006G022000 Potri.009G168600 Potri.T175200 Potri.007G020100 Potri.009G066100 Potri.007G131200 Potri.011G103700 Potri.008G174100 Potri.005G116200 Potri.019G049700 Potri.013G125000 Potri.007G127000 Potri.006G004300 Potri.018G095100 Potri.014G111800 Potri.001G136200 Potri.008G053100 Potri.012G071700 Potri.002G060500 Potri.014G129200 Potri.015G066200 Potri.019G093700 Potri.003G097300
GO:0044238	primary metabolic process	683	Potri.001G404100 Potri.006G046100 Potri.012G011300 Potri.019G078300 Potri.019G008900 Potri.002G033600 Potri.008G099300 Potri.015G023900 Potri.T143600 Potri.009G081800 Potri.019G005300 Potri.001G077900 Potri.010G015400 Potri.002G047500 Potri.012G023300 Potri.013G133100 Potri.018G091000 Potri.008G174100 Potri.017G003100 Potri.015G074200 Potri.007G039800 Potri.004G112300 Potri.017G141300 Potri.018G036600 Potri.019G094200 Potri.019G018500 Potri.004G038000 Potri.011G151200 Potri.T003400 Potri.011G128900 Potri.009G060400 Potri.007G085300 Potri.011G034300 Potri.002G113400 Potri.010G192100 Potri.001G136200 Potri.001G442200 Potri.002G186600 Potri.016G015700 Potri.017G009600 Potri.017G055400 Potri.T090000 Potri.002G075900 Potri.003G085300 Potri.013G121000 Potri.011G028100 Potri.018G019700 Potri.001G114000 Potri.002G114500 Potri.014G096200 Potri.T011000 Potri.002G009400 Potri.002G168700 Potri.013G046400 Potri.004G024600 Potri.003G066300 Potri.T108900 Potri.T022800 Potri.005G113600 Potri.014G022800 Potri.011G028800 Potri.003G081500 Potri.018G034600 Potri.014G184900 Potri.010G025800 Potri.001G038300 Potri.001G452200 Potri.004G023500 Potri.T007800 Potri.010G043900 Potri.002G129100 Potri.008G166200 Potri.011G027500 Potri.018G083600 Potri.013G061800 Potri.008G137700 Potri.019G131800 Potri.013G059900 Potri.007G029500 Potri.003G186000 Potri.009G168600 Potri.011G047500 Potri.004G063500 Potri.002G193000 Potri.011G125000 Potri.017G134200 Potri.001G441400 Potri.011G028600 Potri.007G011700 Potri.001G168000 Potri.012G123700 Potri.018G112000 Potri.001G014100 Potri.011G037900 Potri.001G349900 Potri.005G056700 Potri.019G109400 Potri.003G149700 Potri.001G334900 Potri.004G060400 Potri.005G082200

			Potri.002G089800 Potri.018G019800 Potri.T091200 Potri.009G020700 Potri.006G177500 Potri.002G083800<br>Potri.019G099200 Potri.004G024900 Potri.015G141100 Potri.011G106400 Potri.005G214800 Potri.014G164100<br>Potri.002G019300 Potri.001G411700 Potri.018G038100 Potri.001G222800 Potri.011G035900 Potri.010G142900<br>Potri.007G072300 Potri.006G200600 Potri.007G131200 Potri.011G142100 Potri.006G126000 Potri.012G101000<br>Potri.005G119300 Potri.002G228200 Potri.004G060100 Potri.013G054000 Potri.T018000 Potri.010G024900<br>Potri.005G240100 Potri.008G053100 Potri.006G074900 Potri.017G145200 Potri.008G027500 Potri.009G167400<br>Potri.015G103900 Potri.005G205400 Potri.016G056500 Potri.010G147700 Potri.005G167800 Potri.005G181800<br>Potri.007G051000 Potri.006G241600 Potri.010G120600 Potri.017G134600 Potri.005G215600 Potri.015G018000<br>Potri.018G152200 Potri.017G133800 Potri.018G001600 Potri.019G025500 Potri.004G025100 Potri.018G141700<br>Potri.013G135600 Potri.005G139300 Potri.T175200 Potri.013G060000 Potri.T136800 Potri.016G029900<br>Potri.012G108500 Potri.013G042600 Potri.001G157500 Potri.001G418100 Potri.005G179900 Potri.002G054900<br>Potri.016G123300 Potri.015G099200 Potri.009G016100 Potri.004G037800 Potri.014G136400 Potri.014G195200<br>Potri.016G066700 Potri.T019200 Potri.002G044700 Potri.009G169900 Potri.016G057400 Potri.002G126300<br>Potri.004G106400 Potri.004G191900 Potri.011G039100 Potri.003G185700 Potri.010G159900 Potri.001G068200<br>Potri.004G025800 Potri.003G061700 Potri.016G069400 Potri.014G156400 Potri.002G228400 Potri.012G055500<br>Potri.012G043900 Potri.015G018200 Potri.013G066700 Potri.001G051500 Potri.016G076100 Potri.018G094000<br>Potri.014G103000 Potri.T167100 Potri.006G138800 Potri.T064000 Potri.018G111700 Potri.001G256400<br>Potri.004G181900 Potri.013G114200 Potri.014G141000 Potri.006G050700 Potri.011G129300 Potri.T004800<br>Potri.002G226700 Potri.011G036400 Potri.019G123500 Potri.001G223500 Potri.012G071700 Potri.011G003900<br>Potri.017G148200 Potri.012G042600 Potri.006G219300 Potri.010G181300 Potri.003G003400 Potri.010G078700<br>Potri.017G108800 Potri.011G129000 Potri.006G235500 Potri.005G093200 Potri.013G046100 Potri.003G143900<br>Potri.004G058500 Potri.003G150100 Potri.T032700 Potri.006G224100 Potri.006G105300 Potri.002G219600<br>Potri.006G141500 Potri.T080600 Potri.004G066300 Potri.010G097700 Potri.014G183800 Potri.009G023600<br>Potri.005G105000 Potri.004G025500 Potri.008G160200 Potri.003G079200 Potri.T132900 Potri.001G046100<br>Potri.010G103300 Potri.006G071600 Potri.013G125000 Potri.001G046400 Potri.008G159100 Potri.005G247300<br>Potri.016G059000 Potri.015G110000 Potri.011G034200 Potri.018G088300 Potri.002G065600 Potri.018G114300<br>Potri.018G095100 Potri.015G018600 Potri.013G129800 Potri.017G009500 Potri.011G157100 Potri.005G207300<br>Potri.005G130900 Potri.006G133300 Potri.001G092900 Potri.T090100 Potri.001G099000 Potri.003G132700<br>Potri.017G014200 Potri.012G126100 Potri.004G209300 Potri.019G006100 Potri.003G081200 Potri.004G117800<br>Potri.006G144500 Potri.003G097300 Potri.007G043800 Potri.002G004900 Potri.009G035500 Potri.019G084800<br>Potri.010G153500 Potri.004G051800 Potri.012G067600 Potri.015G122000 Potri.001G067600 Potri.009G066100<br>Potri.005G180200 Potri.012G054700 Potri.010G236800 Potri.011G112000 Potri.010G166200 Potri.011G169600<br>Potri.011G061700 Potri.008G035700 Potri.018G138700 Potri.002G240800 Potri.019G064300 Potri.003G025800<br>Potri.008G068700 Potri.009G086100 Potri.002G013900 Potri.002G093300 Potri.007G089000 Potri.009G119700

			Potri.017G034500 Potri.010G177900 Potri.005G037400 Potri.003G191100 Potri.007G105000 Potri.001G263200<br>Potri.010G025700 Potri.014G141900 Potri.012G091100 Potri.011G075300 Potri.017G102100 Potri.004G023900<br>Potri.005G257900 Potri.003G187000 Potri.002G043300 Potri.016G102500 Potri.001G026200 Potri.T043800<br>Potri.010G001500 Potri.005G214600 Potri.005G116200 Potri.018G109500 Potri.019G099300 Potri.T126700<br>Potri.010G017100 Potri.011G037300 Potri.004G208500 Potri.002G248900 Potri.004G109200 Potri.T097300<br>Potri.016G045000 Potri.010G105700 Potri.016G003400 Potri.001G325100 Potri.016G035700 Potri.006G263600<br>Potri.016G018600 Potri.014G185100 Potri.017G118100 Potri.015G024200 Potri.010G237900 Potri.T075500<br>Potri.011G033900 Potri.006G273000 Potri.019G093700 Potri.007G127000 Potri.010G103100 Potri.008G106700<br>Potri.T095600 Potri.001G286700 Potri.004G025200 Potri.014G052700 Potri.015G092000 Potri.002G204400<br>Potri.011G123300 Potri.019G018100 Potri.007G100600 Potri.016G137900 Potri.003G105400 Potri.008G055900<br>Potri.001G228200 Potri.018G141800 Potri.003G137000 Potri.014G033900 Potri.012G047300 Potri.009G154100<br>Potri.006G175500 Potri.005G162800 Potri.018G063900 Potri.007G012400 Potri.009G107200 Potri.002G181400<br>Potri.012G124100 Potri.006G251800 Potri.001G460600 Potri.006G068300 Potri.T021600 Potri.017G106900<br>Potri.008G089000 Potri.016G140200 Potri.T064400 Potri.014G141400 Potri.012G019900 Potri.007G102100<br>Potri.010G072300 Potri.001G352400 Potri.016G069500 Potri.015G030700 Potri.003G211700 Potri.004G110200<br>Potri.007G045400 Potri.004G061900 Potri.010G100500 Potri.015G018100 Potri.019G093800 Potri.006G225300<br>Potri.003G213700 Potri.019G034700 Potri.001G045500 Potri.001G040000 Potri.T128600 Potri.003G169100<br>Potri.014G038600 Potri.002G173600 Potri.001G328000 Potri.013G158500 Potri.009G080600 Potri.003G211300<br>Potri.014G017200 Potri.004G014700 Potri.006G004300 Potri.006G103200 Potri.007G077600 Potri.005G095600<br>Potri.016G011200 Potri.017G149000 Potri.015G031000 Potri.006G202600 Potri.007G135800 Potri.008G103300<br>Potri.010G083500 Potri.T133000 Potri.011G156100 Potri.018G141500 Potri.001G223800 Potri.001G337400<br>Potri.006G202700 Potri.017G117700 Potri.010G025500 Potri.017G063500 Potri.018G063500 Potri.012G131900<br>Potri.004G233000 Potri.006G049200 Potri.010G224900 Potri.T165400 Potri.T080500 Potri.002G129600<br>Potri.014G101100 Potri.008G059900 Potri.004G081300 Potri.004G056900 Potri.T021200 Potri.002G174500<br>Potri.006G186600 Potri.010G245200 Potri.002G059100 Potri.012G112200 Potri.017G084200 Potri.003G025600<br>Potri.006G058800 Potri.001G455500 Potri.010G018300 Potri.019G111100 Potri.T136500 Potri.002G186400<br>Potri.009G057900 Potri.011G061800 Potri.001G410800 Potri.005G236700 Potri.006G048100 Potri.015G061600<br>Potri.002G198000 Potri.011G028300 Potri.007G079800 Potri.003G108200 Potri.019G007900 Potri.007G125000<br>Potri.001G058800 Potri.T084700 Potri.012G031700 Potri.002G113600 Potri.004G007500 Potri.003G197500<br>Potri.007G011200 Potri.016G072300 Potri.001G073100 Potri.018G006000 Potri.005G177700 Potri.005G195000<br>Potri.T023600 Potri.019G120000 Potri.014G033200 Potri.007G089400 Potri.T089800 Potri.001G252100<br>Potri.007G136200 Potri.007G110600 Potri.011G035100 Potri.006G104200 Potri.003G185800 Potri.001G100200<br>Potri.005G213200 Potri.004G097100 Potri.015G064100 Potri.014G035500 Potri.004G024400 Potri.018G088100<br>Potri.010G043700 Potri.012G002800 Potri.019G005700 Potri.004G096800 Potri.012G033900 Potri.001G412400

			Potri.007G019600 Potri.010G141400 Potri.003G135600 Potri.002G196200 Potri.017G110500 Potri.006G193000 Potri.011G038500 Potri.011G057000 Potri.014G033000 Potri.004G023800 Potri.003G162500 Potri.007G105800 Potri.005G072600 Potri.007G020100 Potri.001G316300 Potri.011G037100 Potri.004G040200 Potri.006G003400 Potri.T093800 Potri.019G049700 Potri.001G163700 Potri.T032200 Potri.012G062300 Potri.006G261900 Potri.003G071400 Potri.004G226900 Potri.013G130000 Potri.015G111000 Potri.001G430700 Potri.017G100200 Potri.002G070500 Potri.017G134100 Potri.014G111800 Potri.006G149700 Potri.005G251900 Potri.005G069000 Potri.004G027400 Potri.004G229800 Potri.002G060500 Potri.001G260800 Potri.012G011700 Potri.013G041000 Potri.001G228300 Potri.003G030900 Potri.012G104600 Potri.002G089900 Potri.017G034700 Potri.011G037700 Potri.006G148800 Potri.005G119200 Potri.011G145000 Potri.014G161300 Potri.001G224000 Potri.004G024800 Potri.004G019500 Potri.006G139700 Potri.005G098200 Potri.013G054200 Potri.003G063100 Potri.016G140300 Potri.002G046200 Potri.005G064100 Potri.002G021500 Potri.007G034500 Potri.011G029100 Potri.016G053800 Potri.010G142800 Potri.019G072800 Potri.T008900 Potri.005G085200 Potri.001G104500 Potri.009G118300 Potri.005G014700 Potri.008G079500 Potri.013G103300 Potri.005G073300 Potri.T004600 Potri.017G117100 Potri.016G128300 Potri.T022600 Potri.013G090300 Potri.010G121100 Potri.018G148300 Potri.001G222900 Potri.T080900 Potri.014G129200 Potri.015G103800 Potri.007G010300 Potri.001G409900 Potri.002G004500 Potri.006G107600 Potri.009G163700 Potri.004G191400 Potri.015G086800 Potri.011G056900 Potri.001G218800 Potri.013G123500 Potri.017G133900 Potri.011G007800 Potri.005G128200 Potri.012G007500 Potri.014G155000 Potri.016G068500 Potri.015G069600 Potri.001G020600 Potri.006G109100 Potri.002G090700 Potri.011G103700 Potri.004G155100 Potri.005G068700 Potri.017G118400 Potri.011G051600 Potri.019G128600 Potri.004G073600 Potri.017G135000 Potri.012G113500 Potri.007G027000 Potri.017G007900 Potri.T128700 Potri.001G140700 Potri.011G058100 Potri.010G072400 Potri.008G116600 Potri.001G223900 Potri.005G204600 Potri.006G041100 Potri.017G152400 Potri.004G153800 Potri.017G151400 Potri.008G040700 Potri.T009800 Potri.001G080900 Potri.011G149700 Potri.007G085700 Potri.001G255100 Potri.008G203200 Potri.013G033200 Potri.012G090500 Potri.001G233400 Potri.016G028900 Potri.006G022000 Potri.004G083900 Potri.003G196600 Potri.004G019300 Potri.001G154200 Potri.010G234400 Potri.007G111100 Potri.014G027500 Potri.014G090300 Potri.017G003200 Potri.017G079500 Potri.T096300 Potri.010G082000 Potri.009G154300 Potri.017G003800 Potri.T136400 Potri.006G052900 Potri.002G031900 Potri.018G134100 Potri.019G010800 Potri.011G065900 Potri.005G167400 Potri.T008700 Potri.T168600 Potri.011G058300 Potri.010G079200 Potri.009G141600 Potri.010G081100 Potri.011G034400 Potri.017G075800 Potri.003G138600 Potri.005G082000 Potri.015G066200 Potri.001G414200
GO:0019538	protein metabolic process	342	Potri.012G011300 Potri.019G078300 Potri.019G008900 Potri.009G081800 Potri.019G005300 Potri.010G015400 Potri.012G023300 Potri.013G133100 Potri.018G091000 Potri.014G164100 Potri.017G003100 Potri.015G074200 Potri.007G039800 Potri.004G112300 Potri.017G141300 Potri.019G094200 Potri.011G151200 Potri.T003400 Potri.011G128900 Potri.007G085300 Potri.011G034300 Potri.T007800 Potri.001G442200 Potri.017G009600 Potri.T090000 Potri.002G075900 Potri.003G085300 Potri.013G121000 Potri.011G028100

			Potri.010G103300 Potri.002G009400 Potri.004G024600 Potri.003G066300 Potri.T108900 Potri.T022800<br>Potri.005G113600 Potri.015G110000 Potri.018G034600 Potri.010G025800 Potri.001G038300 Potri.005G214600<br>Potri.010G017100 Potri.011G037300 Potri.002G129100 Potri.018G114300 Potri.018G083600 Potri.008G137700<br>Potri.019G131800 Potri.013G059900 Potri.003G186000 Potri.009G168600 Potri.004G063500 Potri.001G441400<br>Potri.011G028600 Potri.007G011700 Potri.001G168000 Potri.001G014100 Potri.011G037900 Potri.005G056700<br>Potri.019G109400 Potri.005G082200 Potri.002G089800 Potri.T091200 Potri.009G020700 Potri.019G099200<br>Potri.004G024900 Potri.011G106400 Potri.001G228300 Potri.002G019300 Potri.001G411700 Potri.011G035900<br>Potri.011G142100 Potri.006G126000 Potri.002G228200 Potri.016G011200 Potri.010G024900 Potri.005G240100<br>Potri.017G145200 Potri.008G027500 Potri.015G103900 Potri.005G181800 Potri.007G051000 Potri.015G018000<br>Potri.009G154300 Potri.019G025500 Potri.005G139300 Potri.013G060000 Potri.T136800 Potri.016G029900<br>Potri.009G016100 Potri.001G157500 Potri.001G418100 Potri.005G179900 Potri.002G054900 Potri.016G123300<br>Potri.004G037800 Potri.014G136400 Potri.005G130900 Potri.016G066700 Potri.010G025700 Potri.003G185700<br>Potri.004G025800 Potri.016G069400 Potri.014G156400 Potri.012G055500 Potri.015G018200 Potri.018G094000<br>Potri.T064000 Potri.018G111700 Potri.019G006100 Potri.013G114200 Potri.014G141000 Potri.006G050700<br>Potri.011G129300 Potri.003G025600 Potri.011G036400 Potri.012G071700 Potri.011G003900 Potri.012G042600<br>Potri.006G219300 Potri.004G040200 Potri.010G078700 Potri.011G129000 Potri.006G235500 Potri.013G046100<br>Potri.004G058500 Potri.003G150100 Potri.T032700 Potri.006G141500 Potri.T080600 Potri.004G066300<br>Potri.010G097700 Potri.004G025500 Potri.008G160200 Potri.T132900 Potri.011G028800 Potri.011G034200<br>Potri.010G043900 Potri.011G027500 Potri.006G200600 Potri.015G018600 Potri.017G009500 Potri.007G072300<br>Potri.014G195200 Potri.006G133300 Potri.T090100 Potri.004G209300 Potri.017G148200 Potri.002G004900<br>Potri.009G035500 Potri.015G018100 Potri.005G247300 Potri.012G067600 Potri.015G122000 Potri.009G066100<br>Potri.010G224900 Potri.012G054700 Potri.011G112000 Potri.006G225300 Potri.008G035700 Potri.018G138700<br>Potri.019G064300 Potri.003G025800 Potri.008G068700 Potri.002G013900 Potri.015G030700 Potri.010G177900<br>Potri.005G037400 Potri.003G191100 Potri.017G135000 Potri.011G039100 Potri.012G091100 Potri.011G075300<br>Potri.004G023900 Potri.016G102500 Potri.001G026200 Potri.010G001500 Potri.011G037100 Potri.005G251900<br>Potri.002G248900 Potri.013G103300 Potri.016G045000 Potri.016G035700 Potri.017G118100 Potri.T075500<br>Potri.011G033900 Potri.006G273000 Potri.016G003400 Potri.010G103100 Potri.001G286700 Potri.004G025200<br>Potri.014G052700 Potri.019G018100 Potri.003G105400 Potri.001G228200 Potri.003G137000 Potri.009G154100<br>Potri.004G025100 Potri.012G124100 Potri.T021600 Potri.016G140200 Potri.T064400 Potri.012G019900<br>Potri.007G102100 Potri.016G069500 Potri.017G034500 Potri.003G211700 Potri.004G061900 Potri.019G084800<br>Potri.011G169600 Potri.001G040000 Potri.T128600 Potri.014G038600 Potri.004G014700 Potri.006G103200<br>Potri.007G077600 Potri.015G031000 Potri.006G202600 Potri.010G083500 Potri.T133000 Potri.006G202700<br>Potri.017G117700 Potri.010G025500 Potri.012G131900 Potri.004G233000 Potri.001G430700 Potri.T165400<br>Potri.T080500 Potri.014G101100 Potri.008G059900 Potri.018G148300 Potri.002G174500 Potri.007G027000

			Potri.012G112200 Potri.017G084200 Potri.001G455500 Potri.010G018300 Potri.019G111100 Potri.T136500 Potri.009G167400 Potri.001G410800 Potri.015G061600 Potri.002G198000 Potri.011G028300 Potri.003G108200 Potri.019G007900 Potri.007G125000 Potri.T084700 Potri.T023600 Potri.019G120000 Potri.T089800 Potri.007G136200 Potri.007G110600 Potri.005G213200 Potri.004G097100 Potri.014G035500 Potri.004G024400 Potri.018G088100 Potri.010G043700 Potri.012G002800 Potri.019G005700 Potri.001G412400 Potri.006G193000 Potri.011G038500 Potri.004G023800 Potri.004G096800 Potri.005G072600 Potri.007G020100 Potri.003G211300 Potri.T093800 Potri.T032200 Potri.006G261900 Potri.004G226900 Potri.013G130000 Potri.011G035100 Potri.002G070500 Potri.T126700 Potri.004G027400 Potri.004G229800 Potri.001G260800 Potri.015G086800 Potri.012G104600 Potri.004G191900 Potri.017G034700 Potri.011G037700 Potri.004G024800 Potri.006G139700 Potri.016G140300 Potri.002G046200 Potri.002G021500 Potri.007G034500 Potri.011G029100 Potri.016G053800 Potri.007G127000 Potri.T011000 Potri.005G014700 Potri.004G023500 Potri.011G125000 Potri.001G349900 Potri.T022600 Potri.T097300 Potri.010G121100 Potri.T021200 Potri.T080900 Potri.006G107600 Potri.004G191400 Potri.005G128200 Potri.014G155000 Potri.004G155100 Potri.017G118400 Potri.019G128600 Potri.004G073600 Potri.005G095600 Potri.017G007900 Potri.T128700 Potri.011G058100 Potri.003G185800 Potri.005G204600 Potri.007G135800 Potri.017G152400 Potri.017G151400 Potri.T009800 Potri.008G203200 Potri.013G033200 Potri.012G090500 Potri.004G060100 Potri.004G083900 Potri.003G196600 Potri.007G111100 Potri.T008900 Potri.017G117100 Potri.017G003800 Potri.T136400 Potri.006G052900 Potri.018G134100 Potri.T008700 Potri.011G058300 Potri.010G079200 Potri.011G034400 Potri.017G003200 Potri.015G066200 Potri.001G414200
GO:0006464	protein modification process	306	Potri.010G103100 Potri.006G193000 Potri.012G055500 Potri.004G025200 Potri.012G011300 Potri.014G052700 Potri.019G078300 Potri.015G018200 Potri.004G023800 Potri.012G124100 Potri.010G079200 Potri.015G122000 Potri.001G410800 Potri.001G442200 Potri.019G018100 Potri.018G094000 Potri.003G105400 Potri.009G081800 Potri.007G020100 Potri.019G128600 Potri.T064000 Potri.010G015400 Potri.012G131900 Potri.002G004900 Potri.012G023300 Potri.014G038600 Potri.001G228200 Potri.013G133100 Potri.018G091000 Potri.014G164100 Potri.017G003100 Potri.003G150100 Potri.T093800 Potri.004G024400 Potri.003G137000 Potri.015G074200 Potri.015G061600 Potri.013G059900 Potri.T032200 Potri.017G141300 Potri.010G025700 Potri.019G094200 Potri.009G154100 Potri.004G025100 Potri.T003400 Potri.011G128900 Potri.013G130000 Potri.011G035100 Potri.007G085300 Potri.002G070500 Potri.011G034300 Potri.012G071700 Potri.T022800 Potri.011G003900 Potri.T007800 Potri.005G251900 Potri.012G042600 Potri.006G219300 Potri.004G040200 Potri.017G009600 Potri.004G027400 Potri.019G008900 Potri.T021600 Potri.016G140200 Potri.010G078700 Potri.T064400 Potri.004G229800 Potri.012G019900 Potri.001G260800 Potri.003G085300 Potri.011G129000 Potri.011G169600 Potri.013G121000 Potri.011G028100 Potri.001G228300 Potri.016G069500 Potri.006G235500 Potri.015G030700 Potri.003G211700 Potri.016G011200 Potri.013G046100 Potri.T084700 Potri.017G003800 Potri.019G084800 Potri.004G024600 Potri.011G037700 Potri.015G018100 Potri.T032700 Potri.002G075900

			Potri.T108900 Potri.006G141500 Potri.004G024800 Potri.016G066700 Potri.004G096800 Potri.004G066300<br>Potri.010G097700 Potri.011G028800 Potri.003G066300 Potri.001G040000 Potri.010G025800 Potri.001G038300<br>Potri.005G214600 Potri.T128600 Potri.004G023500 Potri.011G037300 Potri.002G046200 Potri.005G072600<br>Potri.010G043900 Potri.002G129100 Potri.004G025500 Potri.007G034500 Potri.011G029100 Potri.015G031000<br>Potri.018G114300 Potri.016G053800 Potri.018G083600 Potri.T132900 Potri.004G097100 Potri.008G137700<br>Potri.019G131800 Potri.010G103300 Potri.007G039800 Potri.003G186000 Potri.009G168600 Potri.015G018600<br>Potri.007G127000 Potri.T011000 Potri.004G063500 Potri.005G014700 Potri.001G441400 Potri.011G034200<br>Potri.006G103200 Potri.007G077600 Potri.019G005300 Potri.013G103300 Potri.006G200600 Potri.003G025800<br>Potri.011G125000 Potri.011G028600 Potri.017G117100 Potri.001G168000 Potri.T022600 Potri.011G038500<br>Potri.017G009500 Potri.001G014100 Potri.018G111700 Potri.011G037900 Potri.007G011700 Potri.005G056700<br>Potri.019G109400 Potri.010G083500 Potri.T133000 Potri.017G034700 Potri.018G148300 Potri.001G349900<br>Potri.005G130900 Potri.T080900 Potri.006G133300 Potri.005G082200 Potri.002G089800 Potri.T090100<br>Potri.004G058500 Potri.T091200 Potri.009G020700 Potri.006G202700 Potri.017G117700 Potri.006G107600<br>Potri.010G025500 Potri.011G058100 Potri.019G099200 Potri.004G191400 Potri.015G086800 Potri.004G024900<br>Potri.004G209300 Potri.019G006100 Potri.004G233000 Potri.004G060100 Potri.006G052900 Potri.001G430700<br>Potri.003G025600 Potri.011G036400 Potri.011G106400 Potri.004G061900 Potri.T080500 Potri.011G027500<br>Potri.010G121100 Potri.002G019300 Potri.001G411700 Potri.014G101100 Potri.008G059900 Potri.009G035500<br>Potri.014G155000 Potri.011G035900 Potri.010G017100 Potri.012G067600 Potri.T021200 Potri.002G174500<br>Potri.009G066100 Potri.011G142100 Potri.004G155100 Potri.004G226900 Potri.006G126000 Potri.007G027000<br>Potri.010G224900 Potri.011G129300 Potri.012G054700 Potri.017G135000 Potri.017G118400 Potri.001G455500<br>Potri.010G018300 Potri.011G112000 Potri.T136500 Potri.T128700 Potri.009G167400 Potri.005G240100<br>Potri.006G225300 Potri.008G035700 Potri.018G138700 Potri.004G191900 Potri.017G145200 Potri.017G007900<br>Potri.T080600 Potri.003G185800 Potri.008G068700 Potri.011G028300 Potri.003G108200 Potri.019G007900<br>Potri.005G113600 Potri.007G125000 Potri.017G034500 Potri.002G009400 Potri.010G177900 Potri.005G181800<br>Potri.017G152400 Potri.007G051000 Potri.017G151400 Potri.T009800 Potri.006G261900 Potri.005G037400<br>Potri.003G191100 Potri.015G018000 Potri.011G039100 Potri.004G014700 Potri.019G025500 Potri.005G139300<br>Potri.002G228200 Potri.011G075300 Potri.012G090500 Potri.004G023900 Potri.005G128200 Potri.019G111100<br>Potri.013G060000 Potri.T089800 Potri.T136800 Potri.016G102500 Potri.003G211300 Potri.016G140300<br>Potri.007G110600 Potri.010G001500 Potri.004G025800 Potri.007G111100 Potri.001G418100 Potri.006G202600<br>Potri.011G037100 Potri.T126700 Potri.016G123300 Potri.019G120000 Potri.009G016100 Potri.005G213200<br>Potri.T023600 Potri.002G248900 Potri.014G035500 Potri.T008900 Potri.T090000 Potri.016G029900<br>Potri.009G154300 Potri.T097300 Potri.T136400 Potri.014G136400 Potri.014G195200 Potri.016G045000<br>Potri.018G088100 Potri.010G043700 Potri.003G196600 Potri.016G035700 Potri.012G002800 Potri.018G134100<br>Potri.004G083900 Potri.013G114200 Potri.019G005700 Potri.T008700 Potri.017G118100 Potri.011G058300

			Potri.T075500 Potri.003G185700 Potri.001G412400 Potri.008G160200 Potri.011G034400 Potri.017G003200 Potri.011G033900 Potri.015G066200 Potri.006G139700 Potri.006G273000 Potri.016G069400 Potri.014G156400 Potri.001G414200
GO:0016567	protein ubiquitination	23	Potri.010G103100 Potri.014G101100 Potri.004G083900 Potri.006G202600 Potri.002G174500 Potri.007G110600 Potri.018G083600 Potri.008G137700 Potri.015G074200 Potri.007G051000 Potri.017G135000 Potri.009G016100 Potri.005G240100 Potri.002G070500 Potri.015G031000 Potri.008G035700 Potri.012G042600 Potri.012G019900 Potri.010G079200 Potri.006G107600 Potri.016G069500 Potri.006G202700 Potri.016G069400
GO:0080090	regulation of primary metabolic process	145	Potri.002G196200 Potri.001G404100 Potri.011G057000 Potri.013G066700 Potri.011G123300 Potri.016G076100 Potri.013G042600 Potri.016G137900 Potri.014G103000 Potri.006G138800 Potri.001G077900 Potri.003G149700 Potri.004G181900 Potri.006G251800 Potri.018G141800 Potri.001G163700 Potri.004G038000 Potri.019G123500 Potri.001G114000 Potri.002G113400 Potri.010G192100 Potri.002G228400 Potri.003G162500 Potri.002G186600 Potri.010G181300 Potri.012G062300 Potri.001G460600 Potri.017G055400 Potri.008G089000 Potri.001G080900 Potri.013G041000 Potri.014G141400 Potri.010G072300 Potri.001G352400 Potri.018G019700 Potri.002G089900 Potri.002G168700 Potri.001G218800 Potri.011G007800 Potri.006G148800 Potri.002G090700 Potri.006G224100 Potri.005G180200 Potri.006G105300 Potri.002G193000 Potri.010G072400 Potri.019G034700 Potri.005G098200 Potri.013G054200 Potri.010G147700 Potri.001G154200 Potri.001G452200 Potri.005G064100 Potri.012G007500 Potri.008G166200 Potri.001G328000 Potri.013G158500 Potri.007G029500 Potri.006G071600 Potri.005G085200 Potri.011G047500 Potri.016G059000 Potri.003G169100 Potri.002G065600 Potri.005G205400 Potri.014G096200 Potri.017G149000 Potri.016G128300 Potri.013G090300 Potri.008G103300 Potri.011G157100 Potri.002G059100 Potri.001G316300 Potri.006G058800 Potri.018G141500 Potri.004G060400 Potri.001G092900 Potri.001G099000 Potri.018G019800 Potri.008G106700 Potri.003G132700 Potri.015G141100 Potri.003G081200 Potri.006G049200 Potri.011G056900 Potri.005G214800 Potri.002G129600 Potri.007G043800 Potri.018G038100 Potri.004G051800 Potri.016G068500 Potri.004G056900 Potri.010G142900 Potri.006G109100 Potri.006G186600 Potri.012G101000 Potri.011G051600 Potri.005G119300 Potri.013G054000 Potri.002G186400 Potri.010G166200 Potri.011G061700 Potri.011G061800 Potri.006G074900 Potri.005G236700 Potri.008G116600 Potri.007G079800 Potri.009G119700 Potri.001G058800 Potri.012G031700 Potri.016G056500 Potri.006G041100 Potri.004G007500 Potri.003G197500 Potri.011G149700 Potri.013G135600 Potri.007G105000 Potri.007G085700 Potri.018G141700 Potri.016G072300 Potri.005G195000 Potri.005G257900 Potri.002G043300 Potri.T043800 Potri.012G108500 Potri.009G023600 Potri.014G090300 Potri.018G109500 Potri.006G104200 Potri.019G099300 Potri.015G099200 Potri.017G079500 Potri.015G064100 Potri.010G082000 Potri.013G129800 Potri.010G105700 Potri.002G031900 Potri.006G263600 Potri.016G018600 Potri.011G065900 Potri.001G067600 Potri.009G141600 Potri.003G138600 Potri.001G325100 Potri.005G082000
GO:0009605	response to external stimulus	11	Potri.010G075200 Potri.011G110100 Potri.010G153400 Potri.011G110400 Potri.T083400 Potri.010G075700 Potri.016G079000 Potri.009G028300 Potri.010G075300 Potri.010G075800 Potri.005G221000

GO:0006979	response to oxidative stress	16	Potri.016G125000 Potri.001G011500 Potri.016G132900 Potri.003G214900 Potri.014G143200 Potri.005G135300 Potri.008G106400 Potri.007G126600 Potri.013G154400 Potri.016G132700 Potri.017G064100 Potri.015G003500 Potri.001G105200 Potri.016G058200 Potri.013G083600 Potri.T045500
GO:0009611	response to wounding	10	Potri.010G075200 Potri.011G110100 Potri.010G075300 Potri.011G110400 Potri.T083400 Potri.010G075700 Potri.016G079000 Potri.009G028300 Potri.010G075800 Potri.005G221000
GO:0023052	signaling	82	Potri.003G197500 Potri.010G025700 Potri.005G070900 Potri.T022800 Potri.011G036400 Potri.019G097500 Potri.T023600 Potri.007G143300 Potri.007G142500 Potri.013G097000 Potri.017G084800 Potri.011G037100 Potri.011G034200 Potri.011G129000 Potri.001G418100 Potri.011G037900 Potri.010G017100 Potri.011G035900 Potri.013G060000 Potri.T039200 Potri.010G015400 Potri.008G220200 Potri.T021200 Potri.001G409400 Potri.008G160200 Potri.010G153400 Potri.001G228200 Potri.016G053800 Potri.012G048200 Potri.007G111100 Potri.011G136300 Potri.T093800 Potri.T005100 Potri.019G114800 Potri.011G129300 Potri.019G002500 Potri.010G018100 Potri.013G059900 Potri.013G148500 Potri.001G411700 Potri.019G120000 Potri.019G113500 Potri.005G215700 Potri.011G037300 Potri.005G014700 Potri.006G041100 Potri.010G018300 Potri.011G128900 Potri.011G125000 Potri.011G035100 Potri.011G036500 Potri.005G075300 Potri.011G034300 Potri.011G008800 Potri.008G159300 Potri.009G109400 Potri.T022600 Potri.019G002600 Potri.015G039700 Potri.T011800 Potri.007G143000 Potri.001G252100 Potri.011G035300 Potri.016G102500 Potri.005G056700 Potri.001G410800 Potri.004G027400 Potri.004G230000 Potri.T021600 Potri.019G114500 Potri.004G035800 Potri.011G039100 Potri.001G412400 Potri.013G121000 Potri.011G034400 Potri.010G103300 Potri.010G025800 Potri.010G017800 Potri.011G034500 Potri.001G363400 Potri.010G025500 Potri.001G414200
GO:0055085	transmembrane transport	66	Potri.011G130500 Potri.007G126800 Potri.012G005100 Potri.018G035500 Potri.002G078100 Potri.003G197400 Potri.002G049500 Potri.012G011000 Potri.007G003100 Potri.001G111000 Potri.010G089800 Potri.005G177200 Potri.003G197100 Potri.006G110500 Potri.007G027900 Potri.013G083300 Potri.010G026500 Potri.002G005500 Potri.010G055200 Potri.003G198400 Potri.003G134900 Potri.004G207100 Potri.006G265500 Potri.006G252500 Potri.008G150600 Potri.018G149700 Potri.004G083300 Potri.017G141600 Potri.002G187400 Potri.004G162000 Potri.015G081300 Potri.014G097900 Potri.004G151700 Potri.012G033400 Potri.006G266000 Potri.004G033600 Potri.014G000800 Potri.002G092500 Potri.012G051900 Potri.015G059600 Potri.001G258600 Potri.001G375300 Potri.001G362200 Potri.012G131300 Potri.005G099300 Potri.015G026700 Potri.014G113200 Potri.010G116900 Potri.012G050500 Potri.010G194100 Potri.001G456400 Potri.002G129400 Potri.007G100700 Potri.009G021700 Potri.018G085200 Potri.003G121400 Potri.010G119100 Potri.012G082100 Potri.001G296100 Potri.008G179500 Potri.017G135400 Potri.014G179400 Potri.006G240000 Potri.004G019900 Potri.001G294100 Potri.002G180100
Function go term	Description	Matched counts	Matched genes
GO:0022804	active transmembrane	32	Potri.005G215600 Potri.002G078100 Potri.001G174500 Potri.012G051900 Potri.007G100700

	transporter activity		Potri.018G006000 Potri.005G018700 Potri.003G059700 Potri.002G047500 Potri.010G055200 Potri.001G020600 Potri.003G198400 Potri.003G134900 Potri.003G121400 Potri.010G116900 Potri.018G149700 Potri.008G159100 Potri.017G141600 Potri.002G187400 Potri.001G233400 Potri.004G162000 Potri.012G033400 Potri.015G059600 Potri.003G197100 Potri.001G362200 Potri.012G131300 Potri.014G113200 Potri.012G050500 Potri.010G119100 Potri.010G081100 Potri.001G296100 Potri.008G179500
GO:0030554	adenyl nucleotide binding	420	Potri.019G020700 Potri.012G011300 Potri.019G078300 Potri.003G150200 Potri.019G008900 Potri.008G099300 Potri.009G081800 Potri.001G463300 Potri.019G005300 Potri.010G015400 Potri.008G220200 Potri.010G055200 Potri.012G023300 Potri.013G133100 Potri.018G091000 Potri.014G164100 Potri.017G003100 Potri.015G061600 Potri.017G141300 Potri.019G094200 Potri.T003400 Potri.011G128900 Potri.006G158600 Potri.007G085300 Potri.011G034300 Potri.011G052800 Potri.T007800 Potri.001G442200 Potri.017G009600 Potri.T090000 Potri.002G075900 Potri.003G085300 Potri.013G121000 Potri.011G028100 Potri.010G103300 Potri.002G009400 Potri.004G024600 Potri.T028500 Potri.003G066300 Potri.T108900 Potri.T022800 Potri.T027200 Potri.005G113600 Potri.011G028800 Potri.010G025800 Potri.001G038300 Potri.005G214600 Potri.010G017100 Potri.011G037300 Potri.002G129100 Potri.011G027500 Potri.007G142500 Potri.019G131800 Potri.013G059900 Potri.003G186000 Potri.009G168600 Potri.004G063500 Potri.001G441400 Potri.T171900 Potri.001G168000 Potri.011G042500 Potri.001G014100 Potri.011G037900 Potri.001G349900 Potri.005G056700 Potri.019G109400 Potri.015G007800 Potri.001G334900 Potri.005G082200 Potri.T091200 Potri.009G020700 Potri.019G099200 Potri.004G024900 Potri.011G106400 Potri.001G228300 Potri.012G020800 Potri.002G019300 Potri.001G411700 Potri.011G035900 Potri.005G167400 Potri.001G363400 Potri.011G142100 Potri.006G126000 Potri.015G100900 Potri.016G011200 Potri.017G140100 Potri.T026900 Potri.010G024900 Potri.019G002600 Potri.006G169000 Potri.017G145200 Potri.005G181800 Potri.019G113500 Potri.009G154300 Potri.019G025500 Potri.005G139300 Potri.013G060000 Potri.T136800 Potri.016G029900 Potri.001G418100 Potri.011G159900 Potri.016G123300 Potri.001G114000 Potri.002G187400 Potri.001G462400 Potri.013G097000 Potri.014G136400 Potri.005G130900 Potri.T105600 Potri.005G073100 Potri.011G155900 Potri.005G084600 Potri.T024900 Potri.010G025700 Potri.003G185700 Potri.004G025800 Potri.014G156400 Potri.012G055500 Potri.015G018200 Potri.006G022100 Potri.T064000 Potri.018G111700 Potri.001G070900 Potri.013G114200 Potri.011G040800 Potri.011G129300 Potri.010G197800 Potri.003G159800 Potri.007G020900 Potri.005G034700 Potri.011G036400 Potri.010G168200 Potri.001G134300 Potri.012G071700 Potri.009G119100 Potri.011G003900 Potri.T011800 Potri.004G096800 Potri.006G219300 Potri.001G362200 Potri.004G040200 Potri.014G113200 Potri.013G030600 Potri.010G078700 Potri.011G129000 Potri.006G235500 Potri.013G046100 Potri.003G150100 Potri.T032700 Potri.006G141500 Potri.T080600 Potri.004G066300 Potri.010G097700 Potri.005G119900 Potri.012G069800 Potri.004G025500 Potri.008G160200 Potri.001G335900 Potri.T132900 Potri.002G114500 Potri.012G032400 Potri.015G110000 Potri.011G034200 Potri.018G088300 Potri.010G043900 Potri.018G114300 Potri.006G200600 Potri.015G018600 Potri.007G039300 Potri.017G009500 Potri.005G207300 Potri.012G067600 Potri.011G161600

			Potri.T129600 Potri.006G133300 Potri.T090100 Potri.016G066700 Potri.008G179500 Potri.012G097300<br>Potri.004G209300 Potri.019G006100 Potri.002G004900 Potri.009G035500 Potri.019G084800 Potri.010G153500<br>Potri.011G162800 Potri.015G122000 Potri.009G066100 Potri.010G224900 Potri.012G054700 Potri.001G049000<br>Potri.011G112000 Potri.004G162000 Potri.012G033400 Potri.011G169600 Potri.018G138700 Potri.003G025800<br>Potri.008G068700 Potri.015G030700 Potri.010G177900 Potri.005G037400 Potri.001G343400 Potri.011G039100<br>Potri.001G461500 Potri.010G044100 Potri.007G143300 Potri.011G075300 Potri.004G023900 Potri.016G102500<br>Potri.010G001500 Potri.011G037100 Potri.011G046400 Potri.005G251900 Potri.018G149700 Potri.002G248900<br>Potri.013G103300 Potri.T026800 Potri.014G195200 Potri.016G045000 Potri.001G462000 Potri.003G197100<br>Potri.017G118100 Potri.010G237900 Potri.T075500 Potri.T026600 Potri.011G008800 Potri.011G033900<br>Potri.006G273000 Potri.011G158500 Potri.016G003400 Potri.003G183200 Potri.001G286700 Potri.004G025200<br>Potri.014G052700 Potri.008G054600 Potri.019G018100 Potri.003G105400 Potri.006G128900 Potri.001G228200<br>Potri.005G146700 Potri.003G137000 Potri.009G154100 Potri.004G025100 Potri.001G189500 Potri.006G115000<br>Potri.002G181400 Potri.012G124100 Potri.001G107900 Potri.006G156900 Potri.T021600 Potri.009G147100<br>Potri.016G140200 Potri.T064400 Potri.004G058500 Potri.017G034500 Potri.003G211700 Potri.004G061900<br>Potri.003G133300 Potri.015G018100 Potri.001G025400 Potri.006G225300 Potri.001G040000 Potri.T128600<br>Potri.014G038600 Potri.004G014700 Potri.006G103200 Potri.007G077600 Potri.T130200 Potri.010G206600<br>Potri.007G011700 Potri.010G083500 Potri.T133000 Potri.016G053800 Potri.001G464800 Potri.012G002800<br>Potri.017G117700 Potri.007G019600 Potri.012G131900 Potri.004G233000 Potri.001G430700 Potri.004G034100<br>Potri.T080500 Potri.001G042600 Potri.008G059900 Potri.018G148300 Potri.008G054000 Potri.007G027000<br>Potri.003G185800 Potri.003G025600 Potri.001G455500 Potri.010G018300 Potri.019G111100 Potri.T136500<br>Potri.009G167400 Potri.T005100 Potri.001G410800 Potri.T032200 Potri.007G039800 Potri.011G028300<br>Potri.003G108200 Potri.001G311300 Potri.019G007900 Potri.007G125000 Potri.T084700 Potri.T154200<br>Potri.005G026200 Potri.001G048700 Potri.007G011200 Potri.003G143600 Potri.T023600 Potri.019G120000<br>Potri.T089800 Potri.008G047900 Potri.019G114800 Potri.005G213200 Potri.004G097100 Potri.014G035500<br>Potri.004G024400 Potri.015G059600 Potri.018G088100 Potri.010G043700 Potri.019G005700 Potri.001G412400<br>Potri.010G025500 Potri.006G193000 Potri.011G038500 Potri.003G198400 Potri.011G162900 Potri.004G023800<br>Potri.005G072600 Potri.006G147100 Potri.007G020100 Potri.001G461800 Potri.003G211300 Potri.T093800<br>Potri.001G462600 Potri.011G158100 Potri.006G261900 Potri.012G069700 Potri.004G226900 Potri.012G017600<br>Potri.013G130000 Potri.011G035100 Potri.T126700 Potri.014G102300 Potri.004G027400 Potri.001G260800<br>Potri.012G011700 Potri.002G216800 Potri.015G086800 Potri.004G191900 Potri.017G034700 Potri.011G037700<br>Potri.005G119200 Potri.004G024800 Potri.006G139700 Potri.001G121000 Potri.003G178900 Potri.016G140300<br>Potri.001G461700 Potri.011G157900 Potri.007G012400 Potri.018G138500 Potri.007G034500 Potri.011G029100<br>Potri.002G175600 Potri.007G127000 Potri.T011000 Potri.008G003300 Potri.005G014700 Potri.004G023500<br>Potri.011G125000 Potri.019G113700 Potri.T022600 Potri.001G087500 Potri.T097300 Potri.T104100

			Potri.010G121100 Potri.019G002500 Potri.T021200 Potri.T080900 Potri.001G261500 Potri.001G462200 Potri.008G102800 Potri.004G191400 Potri.019G097500 Potri.005G128200 Potri.014G155000 Potri.004G155100 Potri.017G118400 Potri.019G128600 Potri.004G073600 Potri.015G018000 Potri.017G007900 Potri.011G058100 Potri.001G312200 Potri.017G152400 Potri.017G151400 Potri.T009800 Potri.001G459100 Potri.002G228200 Potri.012G090500 Potri.004G060100 Potri.011G158800 Potri.003G196600 Potri.007G111100 Potri.001G406100 Potri.T008900 Potri.017G117100 Potri.017G003800 Potri.T136400 Potri.006G052900 Potri.010G205700 Potri.018G134100 Potri.T008700 Potri.011G058300 Potri.011G034400 Potri.017G003200 Potri.015G066200 Potri.001G414200
GO:0016209	antioxidant activity	20	Potri.016G125000 Potri.001G011500 Potri.014G122800 Potri.013G154400 Potri.016G132700 Potri.003G214900 Potri.014G143200 Potri.016G132900 Potri.005G135300 Potri.008G106400 Potri.007G126600 Potri.005G026200 Potri.003G159800 Potri.017G064100 Potri.015G003500 Potri.001G105200 Potri.016G058200 Potri.013G083600 Potri.001G070900 Potri.T045500
GO:0070001	aspartic-type peptidase activity	6	Potri.005G204600 Potri.005G179900 Potri.008G203200 Potri.019G064300 Potri.002G054900 Potri.005G095600
GO:0005524	ATP binding	386	Potri.019G020700 Potri.012G011300 Potri.019G078300 Potri.003G150200 Potri.019G008900 Potri.008G099300 Potri.009G081800 Potri.019G005300 Potri.010G015400 Potri.008G220200 Potri.010G055200 Potri.012G023300 Potri.013G133100 Potri.018G091000 Potri.014G164100 Potri.017G003100 Potri.015G061600 Potri.017G141300 Potri.019G094200 Potri.T003400 Potri.011G128900 Potri.006G158600 Potri.007G085300 Potri.011G034300 Potri.011G052800 Potri.T007800 Potri.001G442200 Potri.017G009600 Potri.T090000 Potri.002G075900 Potri.003G085300 Potri.013G121000 Potri.011G028100 Potri.010G103300 Potri.002G009400 Potri.004G024600 Potri.T028500 Potri.003G066300 Potri.T108900 Potri.T022800 Potri.T027200 Potri.005G113600 Potri.015G110000 Potri.010G025800 Potri.001G038300 Potri.005G214600 Potri.010G017100 Potri.011G037300 Potri.002G129100 Potri.011G027500 Potri.007G142500 Potri.019G131800 Potri.013G059900 Potri.003G186000 Potri.009G168600 Potri.004G063500 Potri.001G441400 Potri.T171900 Potri.001G168000 Potri.001G014100 Potri.011G037900 Potri.001G349900 Potri.005G056700 Potri.019G109400 Potri.015G007800 Potri.001G334900 Potri.005G082200 Potri.T091200 Potri.009G020700 Potri.019G099200 Potri.004G024900 Potri.011G106400 Potri.001G228300 Potri.012G020800 Potri.002G019300 Potri.001G411700 Potri.011G035900 Potri.001G363400 Potri.011G142100 Potri.006G126000 Potri.015G100900 Potri.016G011200 Potri.017G140100 Potri.T026900 Potri.010G024900 Potri.019G002600 Potri.006G169000 Potri.017G145200 Potri.005G181800 Potri.019G113500 Potri.019G025500 Potri.005G139300 Potri.013G060000 Potri.T136800 Potri.016G029900 Potri.001G418100 Potri.016G123300 Potri.001G114000 Potri.002G187400 Potri.013G097000 Potri.014G136400 Potri.005G130900 Potri.016G066700 Potri.005G073100 Potri.005G084600 Potri.T024900 Potri.010G025700 Potri.003G185700 Potri.004G025800 Potri.014G156400 Potri.012G055500 Potri.015G018200 Potri.006G022100 Potri.T064000 Potri.010G206600 Potri.013G114200 Potri.011G040800 Potri.011G129300 Potri.010G197800

			Potri.007G020900 Potri.005G034700 Potri.011G036400 Potri.001G134300 Potri.012G071700 Potri.009G119100<br>Potri.011G003900 Potri.T011800 Potri.004G096800 Potri.006G219300 Potri.001G362200 Potri.004G040200<br>Potri.014G113200 Potri.013G030600 Potri.010G078700 Potri.011G129000 Potri.006G235500 Potri.013G046100<br>Potri.003G150100 Potri.T032700 Potri.006G141500 Potri.T080600 Potri.004G066300 Potri.010G097700<br>Potri.005G119900 Potri.012G069800 Potri.004G025500 Potri.008G160200 Potri.T132900 Potri.012G032400<br>Potri.011G028800 Potri.011G034200 Potri.018G088300 Potri.010G043900 Potri.018G114300 Potri.006G200600<br>Potri.015G018600 Potri.007G039300 Potri.017G009500 Potri.005G167400 Potri.T129600 Potri.006G133300<br>Potri.T090100 Potri.T105600 Potri.008G179500 Potri.012G097300 Potri.004G209300 Potri.019G006100<br>Potri.002G004900 Potri.009G035500 Potri.019G084800 Potri.010G153500 Potri.012G067600 Potri.015G122000<br>Potri.009G066100 Potri.010G224900 Potri.012G054700 Potri.001G049000 Potri.011G112000 Potri.004G162000<br>Potri.012G033400 Potri.011G169600 Potri.018G138700 Potri.003G025800 Potri.008G068700 Potri.015G030700<br>Potri.010G177900 Potri.005G037400 Potri.001G343400 Potri.011G039100 Potri.010G044100 Potri.007G143300<br>Potri.011G075300 Potri.004G023900 Potri.016G102500 Potri.010G001500 Potri.011G037100 Potri.011G046400<br>Potri.005G251900 Potri.018G149700 Potri.002G248900 Potri.013G103300 Potri.T026800 Potri.014G195200<br>Potri.016G045000 Potri.003G197100 Potri.017G118100 Potri.010G237900 Potri.T075500 Potri.T026600<br>Potri.011G008800 Potri.011G033900 Potri.006G273000 Potri.016G003400 Potri.003G183200 Potri.001G286700<br>Potri.004G025200 Potri.014G052700 Potri.008G054600 Potri.019G018100 Potri.003G105400 Potri.001G228200<br>Potri.003G137000 Potri.009G154100 Potri.004G025100 Potri.001G189500 Potri.006G115000 Potri.002G181400<br>Potri.012G124100 Potri.001G107900 Potri.006G156900 Potri.T021600 Potri.009G147100 Potri.016G140200<br>Potri.T064400 Potri.004G058500 Potri.017G034500 Potri.003G211700 Potri.004G061900 Potri.015G018100<br>Potri.001G025400 Potri.006G225300 Potri.001G040000 Potri.T128600 Potri.014G038600 Potri.004G014700<br>Potri.006G103200 Potri.007G077600 Potri.T130200 Potri.018G111700 Potri.007G011700 Potri.010G083500<br>Potri.T133000 Potri.016G053800 Potri.012G002800 Potri.017G117700 Potri.007G019600 Potri.012G131900<br>Potri.004G233000 Potri.T080500 Potri.001G042600 Potri.008G059900 Potri.018G148300 Potri.008G054000<br>Potri.007G027000 Potri.003G185800 Potri.003G025600 Potri.001G455500 Potri.010G018300 Potri.019G111100<br>Potri.T136500 Potri.009G167400 Potri.019G113700 Potri.T005100 Potri.001G410800 Potri.007G039800<br>Potri.011G028300 Potri.003G108200 Potri.001G311300 Potri.019G007900 Potri.007G125000 Potri.T084700<br>Potri.T154200 Potri.001G048700 Potri.007G011200 Potri.003G143600 Potri.T023600 Potri.019G120000<br>Potri.T089800 Potri.008G047900 Potri.019G114800 Potri.005G213200 Potri.004G097100 Potri.014G035500<br>Potri.004G024400 Potri.015G059600 Potri.018G088100 Potri.010G043700 Potri.019G005700 Potri.001G412400<br>Potri.010G025500 Potri.006G193000 Potri.011G038500 Potri.003G198400 Potri.004G023800 Potri.005G072600<br>Potri.006G147100 Potri.007G020100 Potri.003G211300 Potri.T093800 Potri.T032200 Potri.006G261900<br>Potri.012G069700 Potri.004G226900 Potri.012G017600 Potri.013G130000 Potri.011G035100 Potri.T126700<br>Potri.014G102300 Potri.004G027400 Potri.001G260800 Potri.002G216800 Potri.015G086800 Potri.004G191900

			Potri.017G034700 Potri.011G037700 Potri.005G119200 Potri.004G024800 Potri.006G139700 Potri.003G178900 Potri.016G140300 Potri.007G012400 Potri.018G138500 Potri.007G034500 Potri.011G029100 Potri.002G175600 Potri.007G127000 Potri.T011000 Potri.008G003300 Potri.005G014700 Potri.004G023500 Potri.011G125000 Potri.009G154300 Potri.T022600 Potri.001G087500 Potri.T097300 Potri.T104100 Potri.010G121100 Potri.019G002500 Potri.T021200 Potri.T080900 Potri.001G261500 Potri.008G102800 Potri.004G191400 Potri.019G097500 Potri.005G128200 Potri.014G155000 Potri.004G155100 Potri.017G118400 Potri.019G128600 Potri.004G073600 Potri.015G018000 Potri.017G007900 Potri.011G058100 Potri.001G312200 Potri.017G152400 Potri.017G151400 Potri.T009800 Potri.002G228200 Potri.012G090500 Potri.004G060100 Potri.003G196600 Potri.007G111100 Potri.001G406100 Potri.T008900 Potri.017G117100 Potri.017G003800 Potri.T136400 Potri.006G052900 Potri.010G205700 Potri.018G134100 Potri.T008700 Potri.011G058300 Potri.011G034400 Potri.017G003200 Potri.015G066200 Potri.001G414200
GO:0005509	calcium ion binding	18	Potri.004G191900 Potri.004G191400 Potri.018G141100 Potri.T080900 Potri.019G055200 Potri.015G109200 Potri.002G075900 Potri.008G159300 Potri.T132900 Potri.003G185700 Potri.012G048200 Potri.T080500 Potri.006G149700 Potri.017G084800 Potri.001G040000 Potri.005G215700 Potri.002G126300 Potri.009G154100
GO:0004091	carboxylesterase activity	19	Potri.011G025400 Potri.005G214100 Potri.001G263200 Potri.002G145500 Potri.002G044700 Potri.015G069600 Potri.015G128700 Potri.014G027500 Potri.T004600 Potri.009G057900 Potri.005G068700 Potri.015G127700 Potri.003G081500 Potri.T004800 Potri.006G149700 Potri.002G202600 Potri.007G100600 Potri.014G127000 Potri.009G086100
GO:0016831	carboxy-lyase activity	7	Potri.007G011200 Potri.010G100500 Potri.017G108800 Potri.010G237900 Potri.003G168800 Potri.018G091400 Potri.003G061700
GO:0050662	coenzyme binding	73	Potri.007G131200 Potri.003G133300 Potri.013G102700 Potri.011G162800 Potri.011G145000 Potri.011G103700 Potri.014G161300 Potri.001G430700 Potri.004G034100 Potri.011G162900 Potri.002G033600 Potri.001G045500 Potri.002G204400 Potri.009G118300 Potri.004G208500 Potri.011G158800 Potri.006G022000 Potri.001G461700 Potri.013G103000 Potri.001G463300 Potri.014G019300 Potri.001G256400 Potri.001G461800 Potri.001G070900 Potri.011G129400 Potri.001G335900 Potri.005G026200 Potri.005G146700 Potri.011G155900 Potri.002G004500 Potri.001G046100 Potri.005G116200 Potri.011G033100 Potri.001G462600 Potri.003G159800 Potri.017G092000 Potri.009G080600 Potri.017G106900 Potri.018G065600 Potri.001G046400 Potri.012G011700 Potri.010G129800 Potri.001G462400 Potri.010G168200 Potri.017G110500 Potri.011G159900 Potri.001G461500 Potri.007G029600 Potri.018G046600 Potri.009G169900 Potri.011G042500 Potri.001G337400 Potri.001G140700 Potri.011G157900 Potri.008G053100 Potri.005G207300 Potri.011G158100 Potri.008G116500 Potri.014G022800 Potri.001G462000 Potri.001G459100 Potri.011G156100 Potri.011G161600 Potri.001G464800 Potri.005G197700 Potri.001G121000 Potri.014G129200 Potri.001G462200 Potri.002G114500 Potri.006G128900 Potri.006G236500 Potri.011G158500 Potri.003G135600
GO:0030234	enzyme regulator activity	29	Potri.011G025400 Potri.010G075200 Potri.019G011000 Potri.010G075300 Potri.015G128700

			Potri.001G309900 Potri.007G111700 Potri.019G121900 Potri.002G145500 Potri.011G110100 Potri.004G035800 Potri.019G006900 Potri.004G000400 Potri.015G127700 Potri.009G028300 Potri.010G075800 Potri.001G225800 Potri.004G067900 Potri.019G124700 Potri.005G221000 Potri.007G111600 Potri.019G010900 Potri.007G111500 Potri.011G110400 Potri.T083400 Potri.010G075700 Potri.016G079000 Potri.002G202600 Potri.014G127000
GO:0050660	FAD binding	34	Potri.003G133300 Potri.011G162800 Potri.001G430700 Potri.004G034100 Potri.011G162900 Potri.001G121000 Potri.011G158800 Potri.001G461700 Potri.001G463300 Potri.011G157900 Potri.001G461800 Potri.001G070900 Potri.001G335900 Potri.005G026200 Potri.005G146700 Potri.001G462600 Potri.003G159800 Potri.011G158100 Potri.002G114500 Potri.001G462400 Potri.010G168200 Potri.011G159900 Potri.001G461500 Potri.011G042500 Potri.005G207300 Potri.011G155900 Potri.001G462000 Potri.011G161600 Potri.001G464800 Potri.012G011700 Potri.001G462200 Potri.006G128900 Potri.001G459100 Potri.011G158500
GO:0008066	glutamate receptor activity	9	Potri.001G374600 Potri.001G375200 Potri.004G052400 Potri.018G011700 Potri.018G096500 Potri.018G009900 Potri.006G268200 Potri.011G062600 Potri.004G052600
GO:0020037	heme binding	62	Potri.014G037400 Potri.009G145400 Potri.016G125000 Potri.007G115500 Potri.018G146100 Potri.001G330500 Potri.004G149000 Potri.014G038000 Potri.001G113900 Potri.T127100 Potri.007G084700 Potri.001G331100 Potri.005G084500 Potri.019G130700 Potri.013G083600 Potri.001G011500 Potri.014G020700 Potri.001G003100 Potri.001G249700 Potri.016G132900 Potri.013G106200 Potri.001G270400 Potri.009G065000 Potri.009G043700 Potri.002G088600 Potri.001G422500 Potri.008G106400 Potri.015G086000 Potri.013G154400 Potri.005G144000 Potri.005G135300 Potri.006G022200 Potri.002G130700 Potri.005G143900 Potri.005G064400 Potri.010G049200 Potri.018G149300 Potri.016G058200 Potri.005G220700 Potri.008G205200 Potri.009G108600 Potri.003G173500 Potri.013G076500 Potri.019G057900 Potri.001G334700 Potri.009G033900 Potri.001G270900 Potri.012G089900 Potri.009G064900 Potri.016G132700 Potri.007G074300 Potri.003G214900 Potri.001G270800 Potri.008G099100 Potri.007G074900 Potri.017G064100 Potri.015G003500 Potri.013G160800 Potri.012G096800 Potri.014G143200 Potri.005G172400 Potri.T045500
GO:0016787	hydrolase activity	197	Potri.011G025400 Potri.005G214100 Potri.015G137100 Potri.014G168400 Potri.001G107900 Potri.T081000 Potri.006G068300 Potri.015G023900 Potri.007G105800 Potri.008G159100 Potri.007G100600 Potri.T143600 Potri.T167100 Potri.018G111700 Potri.002G047500 Potri.010G055200 Potri.008G055900 Potri.009G155800 Potri.008G174100 Potri.006G050700 Potri.006G110900 Potri.T004800 Potri.003G106100 Potri.004G112300 Potri.014G113200 Potri.001G048700 Potri.002G226700 Potri.001G223900 Potri.011G151200 Potri.009G060400 Potri.018G063900 Potri.001G310500 Potri.001G114000 Potri.001G223500 Potri.001G069200 Potri.007G012400 Potri.011G052800 Potri.009G107200 Potri.002G181400 Potri.006G149700 Potri.001G255100 Potri.005G069000 Potri.001G362200 Potri.003G003400 Potri.009G147100 Potri.002G060500 Potri.007G112700 Potri.007G102100 Potri.013G014700 Potri.012G104600 Potri.008G079500 Potri.004G110200 Potri.003G143900 Potri.018G141100 Potri.005G119200 Potri.019G093800 Potri.002G219600 Potri.001G224000 Potri.003G213700 Potri.004G019500 Potri.015G110000 Potri.003G081500 Potri.003G178900 Potri.014G184900 Potri.014G183800 Potri.002G156200

			Potri.018G024200 Potri.005G105000 Potri.012G069800 Potri.001G466300 Potri.002G173600 Potri.010G142800 Potri.019G072800 Potri.012G089400 Potri.005G204600 Potri.013G125000 Potri.005G024100 Potri.008G003300 Potri.005G075300 Potri.018G095100 Potri.T004600 Potri.001G127700 Potri.008G134000 Potri.018G112000 Potri.012G069700 Potri.007G072300 Potri.001G466200 Potri.008G196800 Potri.001G222900 Potri.001G223800 Potri.006G082500 Potri.006G048100 Potri.014G027500 Potri.001G409900 Potri.008G179500 Potri.007G019600 Potri.009G163700 Potri.002G083800 Potri.017G063500 Potri.016G006000 Potri.012G097300 Potri.017G148200 Potri.017G134600 Potri.006G144500 Potri.003G097300 Potri.005G018700 Potri.001G222800 Potri.005G247300 Potri.003G183200 Potri.016G035700 Potri.002G145500 Potri.015G069600 Potri.001G020600 Potri.016G057400 Potri.005G068700 Potri.012G112200 Potri.001G049000 Potri.005G095600 Potri.010G236800 Potri.009G104000 Potri.001G233400 Potri.001G324500 Potri.T018000 Potri.009G057900 Potri.012G033400 Potri.002G135100 Potri.019G064300 Potri.017G050400 Potri.014G022900 Potri.008G027500 Potri.009G086100 Potri.002G013900 Potri.001G311300 Potri.015G103900 Potri.001G312200 Potri.015G095900 Potri.005G167800 Potri.004G153800 Potri.001G189500 Potri.010G120600 Potri.006G115000 Potri.001G032400 Potri.005G074100 Potri.012G033900 Potri.008G203200 Potri.005G215600 Potri.001G263200 Potri.018G152200 Potri.017G102100 Potri.003G071400 Potri.001G073100 Potri.006G150600 Potri.018G006000 Potri.005G177700 Potri.013G033200 Potri.T175200 Potri.003G192600 Potri.008G047900 Potri.001G252100 Potri.004G019300 Potri.016G139700 Potri.003G198400 Potri.001G157500 Potri.004G162000 Potri.015G127700 Potri.005G179900 Potri.011G046400 Potri.002G054900 Potri.018G149700 Potri.002G187400 Potri.015G048700 Potri.007G089400 Potri.004G109200 Potri.004G037800 Potri.006G046100 Potri.T019200 Potri.002G044700 Potri.005G073100 Potri.002G171200 Potri.002G126300 Potri.010G184200 Potri.019G010800 Potri.003G197100 Potri.003G161100 Potri.014G185100 Potri.015G024200 Potri.015G059600 Potri.015G128700 Potri.010G159900 Potri.010G081100 Potri.014G127000 Potri.010G141400 Potri.002G202600 Potri.008G180500 Potri.014G111800 Potri.010G107300 Potri.019G093700
GO:0016798	hydrolase activity, acting on glycosyl bonds	60	Potri.018G141100 Potri.018G152200 Potri.019G093800 Potri.002G219600 Potri.001G224000 Potri.006G144500 Potri.006G068300 Potri.015G023900 Potri.002G173600 Potri.014G184900 Potri.014G183800 Potri.001G222800 Potri.T175200 Potri.006G048100 Potri.018G024200 Potri.018G111700 Potri.004G019300 Potri.007G105800 Potri.008G055900 Potri.008G174100 Potri.010G142800 Potri.019G072800 Potri.004G109200 Potri.001G255100 Potri.010G184200 Potri.013G125000 Potri.019G010800 Potri.T167100 Potri.010G141400 Potri.009G060400 Potri.007G089400 Potri.T018000 Potri.018G095100 Potri.001G223500 Potri.006G046100 Potri.014G111800 Potri.003G097300 Potri.T019200 Potri.018G112000 Potri.005G069000 Potri.016G057400 Potri.002G126300 Potri.003G003400 Potri.001G223900 Potri.004G019500 Potri.014G185100 Potri.001G222900 Potri.001G223800 Potri.002G060500 Potri.012G033900 Potri.010G159900 Potri.005G167800 Potri.001G409900 Potri.004G153800 Potri.008G079500 Potri.015G024200 Potri.004G110200 Potri.003G143900 Potri.019G093700 Potri.009G163700
GO:0005506	iron ion binding	70	Potri.014G037400 Potri.009G145400 Potri.016G125000 Potri.003G133300 Potri.007G115500

			Potri.018G146100 Potri.001G330500 Potri.004G149000 Potri.014G038000 Potri.004G135300 Potri.T127100 Potri.007G084700 Potri.001G331100 Potri.005G084500 Potri.019G130700 Potri.013G083600 Potri.001G011500 Potri.014G020700 Potri.001G003100 Potri.001G249700 Potri.016G132900 Potri.001G070900 Potri.013G106200 Potri.001G270400 Potri.009G065000 Potri.009G043700 Potri.003G159800 Potri.015G086000 Potri.001G100200 Potri.001G422500 Potri.008G106400 Potri.005G144000 Potri.013G154400 Potri.001G270800 Potri.017G100200 Potri.002G088600 Potri.005G135300 Potri.006G022200 Potri.002G130700 Potri.005G143900 Potri.006G177500 Potri.005G064400 Potri.010G049200 Potri.018G149300 Potri.016G058200 Potri.005G220700 Potri.008G205200 Potri.009G108600 Potri.003G173500 Potri.013G076500 Potri.019G057900 Potri.001G334700 Potri.009G033900 Potri.001G270900 Potri.012G089900 Potri.009G064900 Potri.016G132700 Potri.007G074300 Potri.003G214900 Potri.001G113900 Potri.008G099100 Potri.007G074900 Potri.005G026200 Potri.017G064100 Potri.015G003500 Potri.013G160800 Potri.012G096800 Potri.014G143200 Potri.005G172400 Potri.T045500
GO:0016298	lipase activity	13	Potri.001G252100 Potri.002G044700 Potri.015G069600 Potri.001G263200 Potri.014G027500 Potri.T004600 Potri.009G057900 Potri.005G068700 Potri.003G081500 Potri.T004800 Potri.006G149700 Potri.007G100600 Potri.009G086100
GO:0051287	NAD or NADH binding	5	Potri.014G161300 Potri.006G236500 Potri.017G092000 Potri.014G022800 Potri.018G046600
GO:0017111	nucleoside-triphosphatase activity	45	Potri.003G183200 Potri.012G097300 Potri.005G215600 Potri.005G119200 Potri.001G107900 Potri.012G069700 Potri.002G187400 Potri.018G006000 Potri.003G178900 Potri.008G047900 Potri.005G105000 Potri.002G047500 Potri.010G055200 Potri.001G020600 Potri.003G198400 Potri.011G046400 Potri.014G113200 Potri.001G049000 Potri.018G149700 Potri.001G048700 Potri.008G003300 Potri.001G362200 Potri.015G110000 Potri.001G233400 Potri.004G162000 Potri.001G189500 Potri.005G075300 Potri.012G033400 Potri.002G135100 Potri.007G012400 Potri.011G052800 Potri.006G115000 Potri.002G181400 Potri.003G197100 Potri.005G073100 Potri.002G171200 Potri.012G069800 Potri.009G147100 Potri.001G311300 Potri.001G312200 Potri.010G081100 Potri.008G179500 Potri.008G159100 Potri.007G019600 Potri.015G059600
GO:0016491	oxidoreductase activity	233	Potri.008G099100 Potri.016G125000 Potri.019G088500 Potri.013G102700 Potri.001G268600 Potri.003G079200 Potri.009G169900 Potri.010G088800 Potri.011G162900 Potri.002G033600 Potri.001G113900 Potri.011G071100 Potri.016G035200 Potri.001G331100 Potri.005G084500 Potri.019G130700 Potri.013G083600 Potri.T168600 Potri.001G463300 Potri.002G004500 Potri.001G046100 Potri.011G022500 Potri.001G256400 Potri.006G128900 Potri.011G047200 Potri.004G235500 Potri.005G146700 Potri.001G015300 Potri.002G091200 Potri.001G335800 Potri.010G097800 Potri.003G159800 Potri.011G158100 Potri.001G422500 Potri.008G106400 Potri.010G168200 Potri.015G111000 Potri.011G159900 Potri.017G100200 Potri.006G022200 Potri.003G067700 Potri.012G038400 Potri.006G101800 Potri.016G058200 Potri.008G205200 Potri.001G105200 Potri.007G134800 Potri.001G270900 Potri.001G307500 Potri.016G132700 Potri.008G144800 Potri.001G245000 Potri.017G110500 Potri.009G107600 Potri.002G204400 Potri.002G114500 Potri.010G107500 Potri.012G096800 Potri.005G172400 Potri.011G150100 Potri.009G062800 Potri.011G162800 Potri.004G235600 Potri.011G145000 Potri.001G330500

			Potri.018G075000 Potri.004G149000 Potri.002G055300 Potri.001G045500 Potri.T127100 Potri.018G121700 Potri.002G130700 Potri.007G084700 Potri.008G080500 Potri.009G108600 Potri.014G193800 Potri.001G461700 Potri.011G157900 Potri.016G132900 Potri.011G129400 Potri.001G335900 Potri.002G021000 Potri.013G045000 Potri.002G088600 Potri.001G452600 Potri.009G080600 Potri.017G106900 Potri.001G046400 Potri.009G118300 Potri.011G020900 Potri.011G058800 Potri.012G011700 Potri.017G064100 Potri.018G046600 Potri.014G143200 Potri.011G042500 Potri.001G337400 Potri.006G177500 Potri.010G049200 Potri.001G003100 Potri.006G151600 Potri.001G167700 Potri.005G207300 Potri.012G006300 Potri.009G043700 Potri.013G064200 Potri.009G033900 Potri.011G156100 Potri.011G161600 Potri.001G464800 Potri.014G020700 Potri.014G129200 Potri.007G074900 Potri.001G263400 Potri.001G462200 Potri.015G002800 Potri.006G236500 Potri.001G070900 Potri.T045500 Potri.014G037400 Potri.010G073200 Potri.014G022800 Potri.010G097600 Potri.013G106200 Potri.005G200500 Potri.001G430700 Potri.004G034100 Potri.009G065000 Potri.011G047300 Potri.001G229500 Potri.013G123500 Potri.T079500 Potri.003G133300 Potri.006G226900 Potri.005G113900 Potri.019G003200 Potri.010G092400 Potri.T059000 Potri.008G053100 Potri.007G115500 Potri.010G245200 Potri.018G065600 Potri.007G131200 Potri.011G103700 Potri.006G090600 Potri.011G155900 Potri.003G210900 Potri.005G043400 Potri.016G117100 Potri.004G049800 Potri.003G173500 Potri.004G065200 Potri.004G199900 Potri.007G108700 Potri.012G113500 Potri.016G102100 Potri.014G161300 Potri.001G462600 Potri.005G144000 Potri.005G135300 Potri.005G197700 Potri.008G179300 Potri.001G140700 Potri.005G064400 Potri.004G135300 Potri.018G149300 Potri.017G092000 Potri.008G116500 Potri.019G057900 Potri.001G461800 Potri.008G149200 Potri.003G214900 Potri.001G121000 Potri.014G122800 Potri.003G069300 Potri.005G026200 Potri.T030600 Potri.017G017300 Potri.001G459100 Potri.017G144500 Potri.001G190100 Potri.009G145400 Potri.004G208500 Potri.018G146100 Potri.015G086000 Potri.001G461500 Potri.005G113700 Potri.001G334700 Potri.006G105900 Potri.014G038000 Potri.012G091100 Potri.011G158800 Potri.006G022000 Potri.011G033100 Potri.013G103000 Potri.001G382400 Potri.002G234000 Potri.001G249700 Potri.005G032400 Potri.001G327800 Potri.007G126600 Potri.011G150300 Potri.001G270400 Potri.005G116200 Potri.004G075800 Potri.016G078300 Potri.001G011500 Potri.001G100200 Potri.006G101200 Potri.017G004600 Potri.001G462400 Potri.013G154400 Potri.001G451900 Potri.007G029600 Potri.005G079400 Potri.002G013400 Potri.005G220700 Potri.010G183500 Potri.004G106400 Potri.013G076500 Potri.001G462000 Potri.006G089800 Potri.005G143900 Potri.012G089900 Potri.009G064900 Potri.002G189900 Potri.007G074300 Potri.001G270800 Potri.017G075800 Potri.015G003500 Potri.013G160800 Potri.011G158500 Potri.003G135600
GO:0030599	pectinesterase activity	6	Potri.011G025400 Potri.002G145500 Potri.015G128700 Potri.015G127700 Potri.002G202600 Potri.014G127000
GO:0004601	peroxidase activity	19	Potri.016G125000 Potri.001G011500 Potri.013G154400 Potri.016G132700 Potri.003G214900 Potri.014G143200 Potri.016G132900 Potri.005G135300 Potri.008G106400 Potri.007G126600 Potri.005G026200 Potri.003G159800 Potri.017G064100 Potri.015G003500 Potri.001G105200 Potri.016G058200 Potri.013G083600 Potri.001G070900 Potri.T045500

GO:0042578	phosphoric ester hydrolase activity	7	Potri.017G063500 Potri.008G196800 Potri.001G252100 Potri.001G324500 Potri.006G110900 Potri.016G035700 Potri.016G139700
GO:0004672	protein kinase activity	277	Potri.006G193000 Potri.012G055500 Potri.004G025200 Potri.012G011300 Potri.014G052700 Potri.019G078300 Potri.015G018200 Potri.004G023800 Potri.004G096800 Potri.015G122000 Potri.001G410800 Potri.001G442200 Potri.019G018100 Potri.005G072600 Potri.003G105400 Potri.009G081800 Potri.007G020100 Potri.019G128600 Potri.T064000 Potri.010G015400 Potri.012G131900 Potri.002G004900 Potri.012G023300 Potri.014G038600 Potri.001G228200 Potri.013G133100 Potri.018G091000 Potri.014G164100 Potri.017G003100 Potri.003G150100 Potri.T093800 Potri.004G024400 Potri.003G137000 Potri.011G129300 Potri.015G061600 Potri.013G059900 Potri.T032200 Potri.017G141300 Potri.010G025700 Potri.019G094200 Potri.009G154100 Potri.004G025100 Potri.T003400 Potri.011G128900 Potri.013G130000 Potri.011G035100 Potri.007G085300 Potri.011G034300 Potri.012G071700 Potri.T022800 Potri.011G003900 Potri.T007800 Potri.005G251900 Potri.012G124100 Potri.006G219300 Potri.004G040200 Potri.017G009600 Potri.004G027400 Potri.019G008900 Potri.T021600 Potri.016G140200 Potri.010G078700 Potri.T064400 Potri.T090000 Potri.004G058500 Potri.001G260800 Potri.003G085300 Potri.011G129000 Potri.011G169600 Potri.013G121000 Potri.011G028100 Potri.001G228300 Potri.006G235500 Potri.015G030700 Potri.003G211700 Potri.016G011200 Potri.013G046100 Potri.T084700 Potri.017G003800 Potri.019G084800 Potri.004G024600 Potri.011G037700 Potri.015G018100 Potri.T032700 Potri.002G075900 Potri.T108900 Potri.006G141500 Potri.004G024800 Potri.T080600 Potri.004G066300 Potri.010G097700 Potri.011G028800 Potri.003G066300 Potri.001G040000 Potri.010G025800 Potri.001G038300 Potri.005G214600 Potri.T128600 Potri.006G103200 Potri.011G037300 Potri.010G043900 Potri.002G129100 Potri.004G025500 Potri.007G034500 Potri.011G029100 Potri.018G114300 Potri.016G053800 Potri.T132900 Potri.004G097100 Potri.019G131800 Potri.010G103300 Potri.007G039800 Potri.003G186000 Potri.009G168600 Potri.007G127000 Potri.T011000 Potri.004G063500 Potri.005G014700 Potri.001G441400 Potri.011G034200 Potri.T166400 Potri.007G077600 Potri.019G005300 Potri.013G103300 Potri.006G200600 Potri.015G018600 Potri.011G125000 Potri.011G028600 Potri.017G117100 Potri.001G168000 Potri.T022600 Potri.011G038500 Potri.017G009500 Potri.001G014100 Potri.018G111700 Potri.011G037900 Potri.007G011700 Potri.005G056700 Potri.019G109400 Potri.010G083500 Potri.T133000 Potri.017G034700 Potri.018G148300 Potri.001G349900 Potri.005G130900 Potri.T080900 Potri.006G133300 Potri.005G082200 Potri.T090100 Potri.016G066700 Potri.T091200 Potri.009G020700 Potri.012G002800 Potri.017G117700 Potri.010G025500 Potri.011G058100 Potri.019G099200 Potri.004G191400 Potri.013G114200 Potri.004G024900 Potri.004G209300 Potri.019G006100 Potri.004G233000 Potri.004G060100 Potri.006G052900 Potri.003G025600 Potri.011G036400 Potri.011G106400 Potri.004G061900 Potri.T080500 Potri.011G027500 Potri.010G121100 Potri.002G019300 Potri.001G411700 Potri.005G128200 Potri.008G059900 Potri.009G035500 Potri.014G155000 Potri.011G035900 Potri.010G017100 Potri.012G067600 Potri.T021200 Potri.009G066100 Potri.011G142100 Potri.004G155100 Potri.004G226900 Potri.006G126000 Potri.007G027000 Potri.010G224900 Potri.012G054700 Potri.015G018000

			Potri.017G118400 Potri.001G455500 Potri.010G018300 Potri.011G112000 Potri.T136500 Potri.T128700 Potri.009G167400 Potri.006G225300 Potri.018G138700 Potri.004G191900 Potri.017G145200 Potri.017G007900 Potri.003G025800 Potri.003G185800 Potri.008G068700 Potri.011G028300 Potri.003G108200 Potri.019G007900 Potri.005G113600 Potri.007G125000 Potri.017G034500 Potri.002G009400 Potri.010G177900 Potri.005G181800 Potri.017G152400 Potri.017G151400 Potri.T009800 Potri.006G261900 Potri.005G037400 Potri.015G086800 Potri.011G039100 Potri.004G014700 Potri.019G025500 Potri.005G139300 Potri.002G228200 Potri.011G075300 Potri.012G090500 Potri.004G023900 Potri.019G111100 Potri.013G060000 Potri.T089800 Potri.T136800 Potri.016G102500 Potri.003G211300 Potri.016G140300 Potri.010G001500 Potri.004G025800 Potri.007G111100 Potri.001G418100 Potri.011G037100 Potri.T126700 Potri.016G123300 Potri.019G120000 Potri.005G213200 Potri.T023600 Potri.002G248900 Potri.014G035500 Potri.T008900 Potri.016G029900 Potri.009G154300 Potri.T097300 Potri.T136400 Potri.014G136400 Potri.014G195200 Potri.004G023500 Potri.016G045000 Potri.018G088100 Potri.010G043700 Potri.003G196600 Potri.018G134100 Potri.019G005700 Potri.T008700 Potri.017G118100 Potri.011G058300 Potri.T075500 Potri.003G185700 Potri.001G412400 Potri.008G160200 Potri.011G034400 Potri.017G003200 Potri.011G033900 Potri.015G066200 Potri.006G139700 Potri.006G273000 Potri.014G156400 Potri.001G414200
GO:0004674	protein serine/threonine kinase activity	41	Potri.004G191400 Potri.010G025700 Potri.T022800 Potri.T023600 Potri.T080500 Potri.001G411700 Potri.001G040000 Potri.019G120000 Potri.010G025800 Potri.001G038300 Potri.010G017100 Potri.011G037300 Potri.T089800 Potri.010G015400 Potri.018G148300 Potri.T132900 Potri.001G418100 Potri.011G037100 Potri.011G129300 Potri.003G186000 Potri.T126700 Potri.003G185800 Potri.009G154100 Potri.005G014700 Potri.001G441400 Potri.010G018300 Potri.011G128900 Potri.T128700 Potri.011G125000 Potri.T097300 Potri.T022600 Potri.001G410800 Potri.011G037900 Potri.T021600 Potri.T021200 Potri.T080900 Potri.011G039100 Potri.011G129000 Potri.T166400 Potri.010G025500 Potri.001G414200
GO:0017171	serine hydrolase activity	7	Potri.002G013900 Potri.005G247300 Potri.011G151200 Potri.004G037800 Potri.007G102100 Potri.004G112300 Potri.007G072300
GO:0016229	steroid dehydrogenase activity	17	Potri.001G046100 Potri.011G156100 Potri.001G256400 Potri.T168600 Potri.007G131200 Potri.011G103700 Potri.014G129200 Potri.002G004500 Potri.002G033600 Potri.001G045500 Potri.002G204400 Potri.001G140700 Potri.009G118300 Potri.008G053100 Potri.017G110500 Potri.006G022000 Potri.001G046400
GO:0003700	transcription factor activity	97	Potri.002G196200 Potri.011G057000 Potri.013G042600 Potri.016G137900 Potri.006G138800 Potri.001G077900 Potri.010G181300 Potri.001G163700 Potri.019G123500 Potri.002G113400 Potri.010G192100 Potri.002G228400 Potri.003G162500 Potri.002G186600 Potri.006G251800 Potri.012G062300 Potri.001G460600 Potri.017G055400 Potri.014G141400 Potri.010G072300 Potri.001G352400 Potri.018G019700 Potri.014G096200 Potri.002G168700 Potri.011G007800 Potri.002G090700 Potri.006G224100 Potri.011G051600 Potri.006G105300 Potri.002G193000 Potri.010G072400 Potri.001G154200 Potri.009G023600 Potri.014G103000 Potri.008G166200 Potri.001G328000 Potri.013G158500 Potri.007G029500 Potri.006G071600 Potri.005G085200 Potri.016G059000

			Potri.003G169100 Potri.002G065600 Potri.017G149000 Potri.016G128300 Potri.013G090300 Potri.008G103300 Potri.011G157100 Potri.002G059100 Potri.006G058800 Potri.004G060400 Potri.001G092900 Potri.001G099000 Potri.018G019800 Potri.008G106700 Potri.003G132700 Potri.015G141100 Potri.003G081200 Potri.006G049200 Potri.011G056900 Potri.005G214800 Potri.007G043800 Potri.018G038100 Potri.004G051800 Potri.010G142900 Potri.006G109100 Potri.012G101000 Potri.005G119300 Potri.014G090300 Potri.011G061700 Potri.011G061800 Potri.007G079800 Potri.009G119700 Potri.001G058800 Potri.012G031700 Potri.016G056500 Potri.010G147700 Potri.004G007500 Potri.013G135600 Potri.007G085700 Potri.016G072300 Potri.005G195000 Potri.005G257900 Potri.002G043300 Potri.T043800 Potri.012G108500 Potri.006G104200 Potri.015G099200 Potri.017G079500 Potri.015G064100 Potri.010G082000 Potri.002G031900 Potri.006G263600 Potri.016G018600 Potri.001G067600 Potri.003G138600 Potri.005G082000
GO:0016757	transferase activity, transferring glycosyl groups	55	Potri.007G045400 Potri.001G158000 Potri.009G077500 Potri.004G117800 Potri.006G047200 Potri.016G097400 Potri.018G140400 Potri.006G231100 Potri.016G016200 Potri.001G303300 Potri.001G030600 Potri.003G100500 Potri.002G191600 Potri.018G094000 Potri.006G022900 Potri.009G044600 Potri.016G016100 Potri.009G095100 Potri.T148700 Potri.001G033800 Potri.009G099000 Potri.016G028900 Potri.017G039600 Potri.006G023700 Potri.008G024900 Potri.019G049700 Potri.006G048500 Potri.016G014400 Potri.001G034100 Potri.014G146000 Potri.015G076500 Potri.015G045500 Potri.006G055600 Potri.006G004300 Potri.016G114200 Potri.017G052000 Potri.018G095100 Potri.008G192600 Potri.007G141900 Potri.001G136200 Potri.003G191200 Potri.016G016500 Potri.001G302400 Potri.016G015700 Potri.002G162300 Potri.003G210400 Potri.006G120600 Potri.002G060500 Potri.007G132400 Potri.002G089800 Potri.001G282100 Potri.001G281900 Potri.002G098400 Potri.008G123500 Potri.003G097300
GO:0016769	transferase activity, transferring nitrogenous groups	12	Potri.006G241600 Potri.016G132200 Potri.001G099400 Potri.004G229800 Potri.002G113600 Potri.002G113900 Potri.T079800 Potri.015G103800 Potri.002G163700 Potri.017G014200 Potri.002G091500 Potri.003G132300
GO:0016772	transferase activity, transferring phosphorus-containing groups	296	Potri.006G193000 Potri.012G055500 Potri.004G025200 Potri.012G011300 Potri.014G052700 Potri.019G078300 Potri.015G018200 Potri.013G148500 Potri.004G023800 Potri.004G096800 Potri.015G122000 Potri.001G410800 Potri.001G442200 Potri.019G018100 Potri.005G072600 Potri.003G105400 Potri.009G081800 Potri.007G020100 Potri.019G128600 Potri.T064000 Potri.010G015400 Potri.012G131900 Potri.002G004900 Potri.012G023300 Potri.014G038600 Potri.001G228200 Potri.013G133100 Potri.018G091000 Potri.014G164100 Potri.017G003100 Potri.003G150100 Potri.T093800 Potri.004G024400 Potri.003G137000 Potri.011G129300 Potri.015G061600 Potri.013G059900 Potri.T032200 Potri.017G141300 Potri.010G025700 Potri.019G094200 Potri.009G154100 Potri.004G025100 Potri.T003400 Potri.011G128900 Potri.005G070900 Potri.013G130000 Potri.011G035100 Potri.007G085300 Potri.010G081200 Potri.011G034300 Potri.012G071700 Potri.T022800 Potri.011G003900 Potri.015G013600 Potri.T007800 Potri.005G251900 Potri.012G124100 Potri.006G219300 Potri.004G040200 Potri.017G009600 Potri.004G027400 Potri.019G008900 Potri.T021600 Potri.016G140200

			Potri.010G078700 Potri.T064400 Potri.T090000 Potri.004G058500 Potri.001G260800 Potri.003G085300<br>Potri.011G129000 Potri.011G169600 Potri.013G121000 Potri.011G028100 Potri.001G228300 Potri.003G030900<br>Potri.T166400 Potri.006G235500 Potri.015G030700 Potri.003G211700 Potri.017G034700 Potri.016G011200<br>Potri.013G046100 Potri.T084700 Potri.017G003800 Potri.008G135800 Potri.004G024600 Potri.011G037700<br>Potri.015G018100 Potri.T032700 Potri.002G075900 Potri.T108900 Potri.006G141500 Potri.004G024800<br>Potri.T080600 Potri.014G099000 Potri.010G097700 Potri.011G028800 Potri.003G066300 Potri.001G040000<br>Potri.010G025800 Potri.001G038300 Potri.005G214600 Potri.T128600 Potri.006G103200 Potri.011G037300<br>Potri.010G043900 Potri.002G129100 Potri.004G025500 Potri.007G034500 Potri.011G029100 Potri.018G114300<br>Potri.016G053800 Potri.T132900 Potri.004G097100 Potri.019G131800 Potri.010G103300 Potri.007G039800<br>Potri.003G186000 Potri.009G168600 Potri.007G127000 Potri.T011000 Potri.004G063500 Potri.005G014700<br>Potri.001G441400 Potri.011G034200 Potri.018G088300 Potri.007G077600 Potri.019G005300 Potri.013G103300<br>Potri.006G200600 Potri.015G018600 Potri.011G125000 Potri.011G028600 Potri.017G117100 Potri.001G168000<br>Potri.T022600 Potri.011G038500 Potri.017G009500 Potri.001G014100 Potri.018G111700 Potri.011G037900<br>Potri.007G011700 Potri.005G056700 Potri.019G109400 Potri.010G083500 Potri.T133000 Potri.001G334900<br>Potri.018G148300 Potri.001G349900 Potri.005G130900 Potri.T080900 Potri.006G133300 Potri.005G082200<br>Potri.007G010300 Potri.T090100 Potri.016G066700 Potri.005G197500 Potri.009G020700 Potri.008G102800<br>Potri.017G117700 Potri.010G025500 Potri.011G058100 Potri.019G099200 Potri.004G191400 Potri.013G114200<br>Potri.004G024900 Potri.004G209300 Potri.019G006100 Potri.004G233000 Potri.004G060100 Potri.006G052900<br>Potri.003G025600 Potri.011G036400 Potri.011G106400 Potri.004G061900 Potri.T080500 Potri.011G027500<br>Potri.010G121100 Potri.002G019300 Potri.001G411700 Potri.005G128200 Potri.008G059900 Potri.009G035500<br>Potri.014G155000 Potri.011G035900 Potri.010G017100 Potri.012G067600 Potri.T021200 Potri.009G066100<br>Potri.011G142100 Potri.004G155100 Potri.004G226900 Potri.006G126000 Potri.007G027000 Potri.008G159000<br>Potri.010G224900 Potri.012G054700 Potri.015G018000 Potri.017G118400 Potri.001G455500 Potri.010G018300<br>Potri.011G112000 Potri.T136500 Potri.T128700 Potri.009G167400 Potri.006G225300 Potri.018G138700<br>Potri.004G191900 Potri.017G145200 Potri.017G007900 Potri.003G025800 Potri.003G185800 Potri.008G068700<br>Potri.011G028300 Potri.004G066300 Potri.003G108200 Potri.019G007900 Potri.005G113600 Potri.007G125000<br>Potri.017G034500 Potri.002G009400 Potri.011G058300 Potri.010G177900 Potri.018G036600 Potri.005G181800<br>Potri.017G152400 Potri.017G151400 Potri.T009800 Potri.006G261900 Potri.005G037400 Potri.015G086800<br>Potri.001G343400 Potri.019G084800 Potri.011G039100 Potri.004G014700 Potri.010G044100 Potri.019G025500<br>Potri.005G139300 Potri.002G228200 Potri.011G075300 Potri.012G090500 Potri.004G023900 Potri.001G412400<br>Potri.019G111100 Potri.013G060000 Potri.T089800 Potri.T136800 Potri.016G102500 Potri.003G211300<br>Potri.016G140300 Potri.010G001500 Potri.004G025800 Potri.007G111100 Potri.001G418100 Potri.011G037100<br>Potri.T126700 Potri.016G123300 Potri.019G120000 Potri.T091200 Potri.005G213200 Potri.T023600<br>Potri.002G248900 Potri.014G035500 Potri.T008900 Potri.016G029900 Potri.009G154300 Potri.T097300

			Potri.T136400 Potri.014G136400 Potri.014G195200 Potri.004G023500 Potri.016G045000 Potri.018G088100 Potri.010G043700 Potri.003G196600 Potri.012G002800 Potri.018G134100 Potri.005G084600 Potri.019G005700 Potri.T008700 Potri.017G118100 Potri.010G237900 Potri.T075500 Potri.003G185700 Potri.006G120700 Potri.008G160200 Potri.011G034400 Potri.017G003200 Potri.011G033900 Potri.015G066200 Potri.006G139700 Potri.006G273000 Potri.014G156400 Potri.001G414200
GO:0005215	transporter activity	86	Potri.011G130500 Potri.010G119100 Potri.011G062600 Potri.013G083300 Potri.007G027900 Potri.002G047500 Potri.010G055200 Potri.006G102800 Potri.001G174500 Potri.001G044900 Potri.017G141600 Potri.014G097900 Potri.004G151700 Potri.012G051900 Potri.001G362200 Potri.004G052600 Potri.014G113200 Potri.002G129400 Potri.009G021700 Potri.003G121400 Potri.011G146900 Potri.001G294100 Potri.007G126800 Potri.001G375200 Potri.004G207100 Potri.003G059700 Potri.007G100700 Potri.010G026500 Potri.016G121400 Potri.004G083300 Potri.010G126300 Potri.004G063000 Potri.008G159100 Potri.018G011700 Potri.001G374600 Potri.008G179500 Potri.014G179400 Potri.008G119600 Potri.003G197400 Potri.019G118400 Potri.005G018700 Potri.009G038800 Potri.001G013400 Potri.002G092500 Potri.001G020600 Potri.003G134900 Potri.006G268200 Potri.014G132500 Potri.011G072100 Potri.010G116900 Potri.004G033600 Potri.008G170100 Potri.001G233400 Potri.004G162000 Potri.004G052400 Potri.012G033400 Potri.010G100800 Potri.014G000800 Potri.001G258600 Potri.002G049500 Potri.008G150600 Potri.012G050500 Potri.010G034700 Potri.018G035500 Potri.001G296100 Potri.002G180100 Potri.005G215600 Potri.002G078100 Potri.002G005500 Potri.018G009900 Potri.018G006000 Potri.007G003100 Potri.010G089800 Potri.010G063500 Potri.003G198400 Potri.018G149700 Potri.003G109800 Potri.002G187400 Potri.015G059600 Potri.012G131300 Potri.003G197100 Potri.018G096500 Potri.018G085200 Potri.010G081100 Potri.017G135400 Potri.006G240000
GO:0004842	ubiquitin-protein ligase activity	23	Potri.010G103100 Potri.014G101100 Potri.004G083900 Potri.006G202600 Potri.002G174500 Potri.007G110600 Potri.018G083600 Potri.008G137700 Potri.015G074200 Potri.007G051000 Potri.017G135000 Potri.009G016100 Potri.005G240100 Potri.002G070500 Potri.015G031000 Potri.008G035700 Potri.012G042600 Potri.012G019900 Potri.010G079200 Potri.006G107600 Potri.016G069500 Potri.006G202700 Potri.016G069400

Supplementary Table S3: Details of GO terms enriched by down-regulated DEGs of Leu at **6hpi of** three infection stages in Figure 4 cont.

Process go term	Description	Matched counts	Matched genes
GO:0044237	cellular metabolic process	5	Potri.017G146600 Potri.005G252000 Potri.014G149400 Potri.006G069400 Potri.016G142800
GO:0006629	lipid metabolic process	6	Potri.018G089500 Potri.018G088800 Potri.018G089400 Potri.018G089300 Potri.018G088500 Potri.004G051900
GO:0043170	macromolecule metabolic	7	Potri.010G220200 Potri.009G141800 Potri.005G252000 Potri.017G146600 Potri.014G149400

	process		Potri.006G069400 Potri.016G142800
GO:0044238	primary metabolic process	14	Potri.010G220200 Potri.009G141800 Potri.018G089500 Potri.018G088800 Potri.005G252000 Potri.018G089400 Potri.017G146600 Potri.014G149400 Potri.018G089300 Potri.018G088500 Potri.006G069400 Potri.016G142800 Potri.014G122200 Potri.004G051900
GO:0019538	protein metabolic process	5	Potri.017G146600 Potri.010G220200 Potri.014G149400 Potri.016G142800 Potri.005G252000
Function go term	Description	Matched counts	Matched genes
GO:0016787	hydrolase activity	9	Potri.010G220200 Potri.018G089500 Potri.018G088800 Potri.018G089400 Potri.009G141800 Potri.018G089300 Potri.018G088500 Potri.014G122200 Potri.004G051900

Supplementary Table S3 (cont.): Details of GO terms enriched by down-regulated DEGs of Leu at **36hpi** of three infection stages in Figure 4

Process go term	Description	Matched counts	Matched genes
GO:0005975	carbohydrate metabolic process	25	Potri.003G038500 Potri.008G189200 Potri.013G152400 Potri.008G131400 Potri.003G088700 Potri.008G056000 Potri.003G123700 Potri.009G141800 Potri.003G139600 Potri.002G200300 Potri.014G125100 Potri.010G125800 Potri.001G225900 Potri.003G139100 Potri.004G159800 Potri.002G197200 Potri.005G059500 Potri.T101100 Potri.011G044300 Potri.010G160200 Potri.011G155500 Potri.003G159700 Potri.001G071000 Potri.002G069000 Potri.017G040800
GO:0046394	carboxylic acid biosynthetic process	7	Potri.001G463800 Potri.009G072900 Potri.009G078200 Potri.011G050800 Potri.014G180300 Potri.010G029100 Potri.008G200100
GO:0044262	cellular carbohydrate metabolic process	8	Potri.011G155500 Potri.003G159700 Potri.013G152400 Potri.001G071000 Potri.014G125100 Potri.003G123700 Potri.003G088700 Potri.002G200300
GO:0006073	cellular glucan metabolic process	5	Potri.001G071000 Potri.014G125100 Potri.013G152400 Potri.003G159700 Potri.002G200300
GO:0044237	cellular metabolic process	67	Potri.009G139400 Potri.002G239700 Potri.002G242700 Potri.006G114400 Potri.001G463800 Potri.013G112900 Potri.001G144800 Potri.004G108200 Potri.005G239900 Potri.011G150700 Potri.014G024200 Potri.003G088700 Potri.001G246400 Potri.003G123700 Potri.015G074600 Potri.019G050200 Potri.007G023600 Potri.002G021800 Potri.005G036600 Potri.002G009300 Potri.017G134900 Potri.018G096100 Potri.009G078200 Potri.011G050800 Potri.014G180300 Potri.002G200300 Potri.014G125100 Potri.007G099400 Potri.002G053000 Potri.005G252000

			Potri.012G071100 Potri.019G091900 Potri.006G235000 Potri.006G104300 Potri.017G081000 Potri.002G003400 Potri.008G038900 Potri.005G257500 Potri.010G185300 Potri.016G126300 Potri.019G052500 Potri.001G106100 Potri.003G205400 Potri.013G080300 Potri.013G152400 Potri.001G264000 Potri.016G096600 Potri.002G137700 Potri.001G229700 Potri.002G143400 Potri.011G155500 Potri.014G025300 Potri.003G159700 Potri.004G138400 Potri.013G048800 Potri.013G082700 Potri.008G200100 Potri.010G029100 Potri.001G071000 Potri.009G072900 Potri.013G082600 Potri.015G043400 Potri.016G142800 Potri.004G150300 Potri.006G035400 Potri.001G209700 Potri.T058000
GO:0044264	cellular polysaccharide metabolic process	5	Potri.001G071000 Potri.014G125100 Potri.013G152400 Potri.003G159700 Potri.002G200300
GO:0044267	cellular protein metabolic process	27	Potri.009G139400 Potri.002G242700 Potri.006G114400 Potri.013G112900 Potri.004G108200 Potri.013G048800 Potri.011G150700 Potri.001G246400 Potri.005G036600 Potri.002G009300 Potri.017G134900 Potri.018G096100 Potri.005G252000 Potri.012G071100 Potri.006G235000 Potri.006G104300 Potri.002G003400 Potri.005G257500 Potri.010G185300 Potri.016G126300 Potri.019G052500 Potri.003G205400 Potri.016G096600 Potri.002G137700 Potri.016G142800 Potri.001G209700 Potri.T058000
GO:0046039	GTP metabolic process	5	Potri.017G081000 Potri.001G106100 Potri.005G239900 Potri.006G035400 Potri.002G021800
GO:0006629	lipid metabolic process	18	Potri.002G219700 Potri.019G024400 Potri.018G089500 Potri.004G172000 Potri.019G015100 Potri.011G050800 Potri.018G089400 Potri.014G180300 Potri.006G166700 Potri.018G089300 Potri.018G088800 Potri.015G043400 Potri.004G150300 Potri.001G463800 Potri.001G263700 Potri.T050800 Potri.010G086100 Potri.018G088500
GO:0043170	macromolecule metabolic process	60	Potri.009G139400 Potri.002G242700 Potri.006G114400 Potri.013G112900 Potri.013G152400 Potri.011G155400 Potri.004G108200 Potri.013G048800 Potri.011G150700 Potri.014G024200 Potri.015G104700 Potri.001G246400 Potri.003G088700 Potri.017G084000 Potri.009G031900 Potri.007G023600 Potri.018G143400 Potri.002G200300 Potri.007G099200 Potri.005G036600 Potri.002G009300 Potri.017G134900 Potri.005G091700 Potri.018G096100 Potri.007G106300 Potri.014G125100 Potri.007G099400 Potri.012G105500 Potri.005G252000 Potri.012G071100 Potri.019G091900 Potri.006G235000 Potri.006G104300 Potri.009G141800 Potri.002G003400 Potri.008G038900 Potri.005G257500 Potri.010G185300 Potri.016G126300 Potri.019G052500 Potri.T101100 Potri.003G205400 Potri.015G074600 Potri.001G264000 Potri.016G096600 Potri.018G111000 Potri.001G229700 Potri.011G155500 Potri.006G076200 Potri.018G014800 Potri.014G025300 Potri.003G159700 Potri.006G183200 Potri.002G137700 Potri.001G071000 Potri.001G065900 Potri.016G142800 Potri.017G040800 Potri.001G209700 Potri.T058000
GO:0007018	microtubule-based	15	Potri.001G360200 Potri.004G031600 Potri.006G082900 Potri.006G210700 Potri.010G153000

	movement		Potri.005G150800 Potri.011G031200 Potri.006G048600 Potri.012G054400 Potri.002G235500 Potri.004G193700 Potri.002G110600 Potri.015G044600 Potri.002G201000 Potri.T113300
GO:0009166	nucleotide catabolic process	5	Potri.017G081000 Potri.001G106100 Potri.005G239900 Potri.006G035400 Potri.002G021800
GO:0055114	oxidation reduction	20	Potri.007G002400 Potri.007G108400 Potri.010G089000 Potri.001G463800 Potri.005G247700 Potri.004G150300 Potri.013G125300 Potri.006G137500 Potri.010G236700 Potri.018G051300 Potri.011G050800 Potri.014G180300 Potri.004G134800 Potri.001G401300 Potri.018G033400 Potri.018G063300 Potri.015G074600 Potri.006G154500 Potri.014G135500 Potri.005G108900
GO:0006793	phosphorus metabolic process	25	Potri.009G139400 Potri.002G242700 Potri.006G114400 Potri.004G108200 Potri.013G048800 Potri.001G246400 Potri.005G036600 Potri.002G009300 Potri.017G134900 Potri.018G096100 Potri.005G252000 Potri.012G071100 Potri.006G235000 Potri.006G104300 Potri.002G003400 Potri.005G257500 Potri.010G185300 Potri.016G126300 Potri.002G137700 Potri.003G205400 Potri.019G052500 Potri.002G143400 Potri.016G142800 Potri.001G209700 Potri.T058000
GO:0016310	phosphorylation	25	Potri.009G139400 Potri.002G242700 Potri.006G114400 Potri.004G108200 Potri.013G048800 Potri.001G246400 Potri.005G036600 Potri.002G009300 Potri.017G134900 Potri.018G096100 Potri.005G252000 Potri.012G071100 Potri.006G235000 Potri.006G104300 Potri.002G003400 Potri.005G257500 Potri.010G185300 Potri.016G126300 Potri.002G137700 Potri.003G205400 Potri.019G052500 Potri.002G143400 Potri.016G142800 Potri.001G209700 Potri.T058000
GO:0005976	polysaccharide metabolic process	8	Potri.002G200300 Potri.003G159700 Potri.013G152400 Potri.T101100 Potri.014G125100 Potri.017G040800 Potri.001G071000 Potri.009G141800
GO:0044238	primary metabolic process	107	Potri.009G139400 Potri.013G112900 Potri.001G246400 Potri.003G123700 Potri.009G031900 Potri.018G096100 Potri.009G078200 Potri.014G125100 Potri.003G139100 Potri.012G105500 Potri.001G225900 Potri.004G159800 Potri.002G197200 Potri.019G015100 Potri.011G044300 Potri.013G080300 Potri.016G096600 Potri.018G111000 Potri.006G076200 Potri.004G172000 Potri.010G029100 Potri.013G048800 Potri.016G142800 Potri.001G263700 Potri.019G024400 Potri.001G144800 Potri.008G131400 Potri.011G155400 Potri.003G088700 Potri.018G143400 Potri.007G099200 Potri.001G065900 Potri.003G139600 Potri.011G050800 Potri.014G180300 Potri.007G106300 Potri.018G089300 Potri.010G125800 Potri.006G235000 Potri.010G185300 Potri.005G059500 Potri.006G166700 Potri.003G205400 Potri.019G052500 Potri.001G229700 Potri.010G160200 Potri.014G025300 Potri.017G134900 Potri.006G183200 Potri.001G071000 Potri.009G072900 Potri.013G082600 Potri.015G043400 Potri.006G035400 Potri.001G209700 Potri.T058000 Potri.003G038500 Potri.002G219700 Potri.002G242700 Potri.006G114400 Potri.018G089400 Potri.004G108200 Potri.005G239900 Potri.014G024200 Potri.015G104700 Potri.007G023600 Potri.009G141800 Potri.005G036600 Potri.018G088800 Potri.017G081000 Potri.002G200300

			Potri.002G137700 Potri.006G104300 Potri.002G003400 Potri.008G038900 Potri.011G155500 Potri.016G126300 Potri.001G106100 Potri.T050800 Potri.008G200100 Potri.002G021800 Potri.005G091700 Potri.004G150300 Potri.017G040800 Potri.001G463800 Potri.018G089500 Potri.008G189200 Potri.013G152400 Potri.011G150700 Potri.008G056000 Potri.010G086100 Potri.019G050200 Potri.002G009300 Potri.005G257500 Potri.007G099400 Potri.012G071100 Potri.019G091900 Potri.018G014800 Potri.005G252000 Potri.015G074600 Potri.001G264000 Potri.T101100 Potri.013G082700 Potri.017G084000 Potri.003G159700 Potri.018G088500 Potri.002G069000
GO:0019538	protein metabolic process	41	Potri.009G139400 Potri.002G242700 Potri.006G114400 Potri.013G112900 Potri.011G155400 Potri.004G108200 Potri.013G048800 Potri.011G150700 Potri.001G246400 Potri.015G104700 Potri.009G031900 Potri.018G143400 Potri.007G099200 Potri.018G111000 Potri.002G009300 Potri.017G134900 Potri.005G091700 Potri.018G096100 Potri.005G257500 Potri.007G106300 Potri.012G105500 Potri.005G252000 Potri.012G071100 Potri.006G235000 Potri.006G104300 Potri.002G003400 Potri.005G036600 Potri.010G185300 Potri.016G126300 Potri.019G052500 Potri.003G205400 Potri.016G096600 Potri.002G137700 Potri.006G076200 Potri.018G014800 Potri.006G183200 Potri.017G084000 Potri.001G065900 Potri.016G142800 Potri.001G209700 Potri.T058000
GO:0006464	protein modification process	24	Potri.009G139400 Potri.002G242700 Potri.006G114400 Potri.004G108200 Potri.013G048800 Potri.001G246400 Potri.005G036600 Potri.002G009300 Potri.017G134900 Potri.018G096100 Potri.005G252000 Potri.012G071100 Potri.006G235000 Potri.006G104300 Potri.002G003400 Potri.005G257500 Potri.010G185300 Potri.016G126300 Potri.002G137700 Potri.003G205400 Potri.019G052500 Potri.016G142800 Potri.001G209700 Potri.T058000
GO:0080090	regulation of primary metabolic process	7	Potri.014G025300 Potri.008G038900 Potri.007G099400 Potri.014G024200 Potri.019G091900 Potri.001G229700 Potri.007G023600
GO:0055085	transmembrane transport	9	Potri.005G040000 Potri.008G017100 Potri.013G108200 Potri.008G127700 Potri.008G000300 Potri.001G331700 Potri.012G144000 Potri.005G037000 Potri.002G092400
**Function go term**	**Description**	**Matched counts**	**Matched genes**
GO:0030554	adenyl nucleotide binding	43	Potri.009G139400 Potri.006G082900 Potri.002G242700 Potri.006G114400 Potri.010G153000 Potri.011G031200 Potri.006G048600 Potri.002G235500 Potri.013G048800 Potri.001G246400 Potri.005G036600 Potri.T113300 Potri.017G134900 Potri.018G096100 Potri.005G150800 Potri.012G054400 Potri.006G210700 Potri.005G252000 Potri.012G071100 Potri.010G153600 Potri.006G104300 Potri.006G235000 Potri.002G003400 Potri.004G031600 Potri.005G257500 Potri.010G185300 Potri.016G126300 Potri.002G137700 Potri.015G074600 Potri.003G205400 Potri.004G193700 Potri.001G264000 Potri.002G110600 Potri.019G052500 Potri.002G009300 Potri.001G360200 Potri.015G044600 Potri.004G108200 Potri.017G084000 Potri.002G201000 Potri.016G142800

			Potri.001G209700 Potri.T058000
GO:0005524	ATP binding	42	Potri.009G139400 Potri.006G082900 Potri.002G242700 Potri.006G114400 Potri.010G153000 Potri.011G031200 Potri.006G048600 Potri.002G235500 Potri.013G048800 Potri.001G246400 Potri.005G036600 Potri.T113300 Potri.017G134900 Potri.018G096100 Potri.005G150800 Potri.012G054400 Potri.006G210700 Potri.005G252000 Potri.012G071100 Potri.010G153600 Potri.006G104300 Potri.006G235000 Potri.002G003400 Potri.004G031600 Potri.005G257500 Potri.010G185300 Potri.016G126300 Potri.002G137700 Potri.003G205400 Potri.004G193700 Potri.001G264000 Potri.002G110600 Potri.019G052500 Potri.002G009300 Potri.001G360200 Potri.015G044600 Potri.004G108200 Potri.017G084000 Potri.002G201000 Potri.016G142800 Potri.001G209700 Potri.T058000
GO:0004180	carboxypeptidase activity	5	Potri.001G065900 Potri.015G104700 Potri.012G105500 Potri.005G091700 Potri.006G183200
GO:0020037	heme binding	8	Potri.007G002400 Potri.013G125300 Potri.010G236700 Potri.018G051300 Potri.009G110800 Potri.004G134800 Potri.006G154500 Potri.005G108900
GO:0016787	hydrolase activity	70	Potri.003G038500 Potri.002G219700 Potri.019G024400 Potri.018G089500 Potri.008G189200 Potri.006G082900 Potri.010G153000 Potri.005G059500 Potri.006G048600 Potri.008G056000 Potri.002G235500 Potri.005G239900 Potri.018G089400 Potri.011G155400 Potri.015G104700 Potri.001G106100 Potri.009G031900 Potri.018G143400 Potri.002G021800 Potri.007G099200 Potri.T113300 Potri.018G088800 Potri.005G091700 Potri.001G065900 Potri.009G078200 Potri.003G139600 Potri.018G014800 Potri.005G150800 Potri.007G106300 Potri.012G054400 Potri.018G089300 Potri.003G139100 Potri.012G105500 Potri.001G225900 Potri.003G112600 Potri.010G125800 Potri.010G153600 Potri.002G201000 Potri.009G141800 Potri.004G159800 Potri.002G197200 Potri.004G031600 Potri.003G076900 Potri.006G210700 Potri.019G031200 Potri.017G076400 Potri.011G031200 Potri.T101100 Potri.006G166700 Potri.011G044300 Potri.013G152400 Potri.003G159700 Potri.014G117100 Potri.002G110600 Potri.018G111000 Potri.001G360200 Potri.006G076200 Potri.015G044600 Potri.004G172000 Potri.006G137800 Potri.004G193700 Potri.006G183200 Potri.017G084000 Potri.001G071000 Potri.017G081000 Potri.018G088500 Potri.002G069000 Potri.017G040800 Potri.006G035400 Potri.002G257700
GO:0016798	hydrolase activity, acting on glycosyl bonds	18	Potri.003G038500 Potri.001G071000 Potri.004G159800 Potri.002G197200 Potri.008G189200 Potri.003G159700 Potri.003G139600 Potri.005G059500 Potri.T101100 Potri.008G056000 Potri.011G044300 Potri.003G139100 Potri.001G225900 Potri.017G040800 Potri.003G112600 Potri.010G125800 Potri.013G152400 Potri.009G141800
GO:0005506	iron ion binding	10	Potri.007G002400 Potri.001G463800 Potri.013G125300 Potri.010G236700 Potri.018G051300 Potri.009G110800 Potri.004G134800 Potri.014G180300 Potri.006G154500 Potri.005G108900
GO:0003777	microtubule motor activity	15	Potri.001G360200 Potri.004G031600 Potri.006G082900 Potri.006G210700 Potri.010G153000

			Potri.005G150800 Potri.011G031200 Potri.006G048600 Potri.012G054400 Potri.002G235500 Potri.004G193700 Potri.002G110600 Potri.015G044600 Potri.002G201000 Potri.T113300
GO:0017111	nucleoside-triphosphatase activity	22	Potri.006G082900 Potri.010G153000 Potri.006G048600 Potri.002G235500 Potri.005G239900 Potri.002G201000 Potri.T113300 Potri.017G081000 Potri.005G150800 Potri.012G054400 Potri.010G153600 Potri.004G031600 Potri.006G210700 Potri.011G031200 Potri.001G106100 Potri.002G110600 Potri.004G193700 Potri.001G360200 Potri.015G044600 Potri.017G084000 Potri.002G021800 Potri.006G035400
GO:0016491	oxidoreductase activity	27	Potri.007G002400 Potri.007G108400 Potri.001G463800 Potri.005G247700 Potri.010G089000 Potri.010G243700 Potri.001G401300 Potri.008G214800 Potri.011G050800 Potri.014G180300 Potri.014G115300 Potri.018G063300 Potri.006G154500 Potri.014G135500 Potri.010G236700 Potri.006G137500 Potri.001G416500 Potri.015G074600 Potri.018G033400 Potri.005G108900 Potri.008G214500 Potri.013G125300 Potri.018G051300 Potri.008G214600 Potri.004G134800 Potri.004G150300 Potri.014G134200
GO:0004672	protein kinase activity	24	Potri.009G139400 Potri.002G242700 Potri.006G114400 Potri.004G108200 Potri.013G048800 Potri.001G246400 Potri.005G036600 Potri.002G009300 Potri.017G134900 Potri.018G096100 Potri.005G252000 Potri.012G071100 Potri.006G235000 Potri.006G104300 Potri.002G003400 Potri.005G257500 Potri.010G185300 Potri.016G126300 Potri.002G137700 Potri.003G205400 Potri.019G052500 Potri.016G142800 Potri.001G209700 Potri.T058000
GO:0017171	serine hydrolase activity	10	Potri.018G143400 Potri.006G076200 Potri.001G065900 Potri.006G183200 Potri.005G091700 Potri.012G105500 Potri.011G155400 Potri.015G104700 Potri.018G111000 Potri.009G031900
GO:0003700	transcription factor activity	5	Potri.014G025300 Potri.019G091900 Potri.001G229700 Potri.008G038900 Potri.014G024200
GO:0016757	transferase activity, transferring glycosyl groups	8	Potri.002G200300 Potri.003G159700 Potri.013G152400 Potri.001G071000 Potri.014G125100 Potri.016G020900 Potri.002G044200 Potri.010G160200
GO:0016772	transferase activity, transferring phosphorus-containing groups	25	Potri.009G139400 Potri.002G242700 Potri.006G114400 Potri.004G108200 Potri.013G048800 Potri.001G246400 Potri.003G088700 Potri.005G036600 Potri.002G009300 Potri.017G134900 Potri.018G096100 Potri.005G252000 Potri.012G071100 Potri.006G235000 Potri.006G104300 Potri.002G003400 Potri.005G257500 Potri.010G185300 Potri.016G126300 Potri.002G137700 Potri.003G205400 Potri.019G052500 Potri.016G142800 Potri.001G209700 Potri.T058000
GO:0005215	transporter activity	17	Potri.005G040000 Potri.002G249100 Potri.008G017100 Potri.012G144000 Potri.005G109200 Potri.001G331700 Potri.010G112900 Potri.003G050900 Potri.016G098200 Potri.008G000300 Potri.013G108200 Potri.008G100300 Potri.006G121700 Potri.005G037000 Potri.004G216500 Potri.002G092400 Potri.001G186700

Supplementary Table S3: Details of GO terms enriched by down-regulated DEGs of Leu at **96hpi** of three infection stages in Figure 4 cont.

Process go term	Description	Matched counts	Matched genes
GO:0009309	amine biosynthetic process	6	Potri.010G249600 Potri.016G036900 Potri.008G200100 Potri.010G029100 Potri.008G198800 Potri.015G135400
GO:0016051	carbohydrate biosynthetic process	11	Potri.011G069600 Potri.006G181900 Potri.001G012200 Potri.012G128200 Potri.018G103900 Potri.001G320000 Potri.002G257900 Potri.002G200300 Potri.014G125100 Potri.004G059600 Potri.001G237200
GO:0005975	carbohydrate metabolic process	69	Potri.004G086400 Potri.014G158400 Potri.014G146100 Potri.009G073800 Potri.014G146600 Potri.010G141600 Potri.004G059600 Potri.004G162400 Potri.017G040800 Potri.002G257900 Potri.002G023900 Potri.017G130200 Potri.003G088700 Potri.003G159700 Potri.001G092200 Potri.008G189200 Potri.002G069000 Potri.002G200300 Potri.011G069600 Potri.002G224600 Potri.010G042100 Potri.014G122200 Potri.002G007300 Potri.013G152400 Potri.009G141800 Potri.008G063800 Potri.014G125100 Potri.006G235100 Potri.002G094000 Potri.001G237200 Potri.008G084500 Potri.010G008600 Potri.010G125800 Potri.008G120000 Potri.003G139100 Potri.009G124100 Potri.004G159800 Potri.002G197200 Potri.001G200400 Potri.011G155500 Potri.005G120500 Potri.008G132700 Potri.005G238600 Potri.004G021000 Potri.005G059500 Potri.T101100 Potri.014G126900 Potri.011G044300 Potri.006G094400 Potri.018G068600 Potri.012G128200 Potri.013G095500 Potri.003G131700 Potri.007G008500 Potri.010G160200 Potri.004G013900 Potri.011G152400 Potri.004G175800 Potri.008G100500 Potri.006G181900 Potri.001G012200 Potri.011G159000 Potri.011G154100 Potri.001G071000 Potri.018G103900 Potri.012G106500 Potri.001G320000 Potri.001G299000 Potri.006G071200
GO:0046394	carboxylic acid biosynthetic process	13	Potri.010G249600 Potri.010G255500 Potri.016G036900 Potri.011G050800 Potri.008G200100 Potri.010G029100 Potri.014G180300 Potri.001G234500 Potri.001G463800 Potri.013G015100 Potri.011G162000 Potri.015G135400 Potri.010G125300
GO:0044036	cell wall macromolecule metabolic process	5	Potri.009G141800 Potri.014G146600 Potri.010G141600 Potri.004G183500 Potri.002G084800
GO:0071554	cell wall organization or biogenesis	13	Potri.006G134500 Potri.010G141600 Potri.011G135000 Potri.003G076900 Potri.014G146600 Potri.015G013700 Potri.007G107300 Potri.009G141800 Potri.010G109400 Potri.004G183500 Potri.014G117100 Potri.012G014500 Potri.002G084800
GO:0044262	cellular carbohydrate	29	Potri.009G073800 Potri.004G162400 Potri.002G257900 Potri.013G152400 Potri.004G059600

	metabolic process		Potri.003G088700 Potri.003G159700 Potri.011G069600 Potri.002G200300 Potri.014G125100 Potri.006G235100 Potri.004G013900 Potri.008G084500 Potri.009G124100 Potri.004G021000 Potri.007G008500 Potri.014G146100 Potri.012G128200 Potri.013G095500 Potri.001G237200 Potri.004G175800 Potri.011G155500 Potri.006G181900 Potri.001G012200 Potri.001G071000 Potri.018G103900 Potri.012G106500 Potri.001G320000 Potri.006G071200
GO:0006073	cellular glucan metabolic process	15	Potri.001G071000 Potri.011G069600 Potri.006G181900 Potri.001G012200 Potri.018G103900 Potri.003G159700 Potri.002G257900 Potri.007G008500 Potri.002G200300 Potri.014G125100 Potri.004G021000 Potri.014G146100 Potri.004G059600 Potri.013G152400 Potri.006G071200
GO:0044237	cellular metabolic process	363	Potri.014G099900 Potri.016G115200 Potri.013G112900 Potri.007G009200 Potri.009G139400 Potri.014G180300 Potri.T099400 Potri.014G175600 Potri.011G139800 Potri.014G139300 Potri.014G125100 Potri.013G059600 Potri.006G069400 Potri.002G070900 Potri.011G126700 Potri.006G130000 Potri.001G287400 Potri.006G255200 Potri.019G131300 Potri.010G052000 Potri.005G010700 Potri.018G068700 Potri.008G061000 Potri.016G142800 Potri.012G031100 Potri.002G108000 Potri.001G144800 Potri.001G156600 Potri.008G130000 Potri.010G249600 Potri.005G207200 Potri.016G114400 Potri.T161200 Potri.003G107600 Potri.006G034300 Potri.006G235000 Potri.001G155100 Potri.001G374200 Potri.012G126500 Potri.013G080400 Potri.002G137700 Potri.013G143100 Potri.016G078600 Potri.010G120100 Potri.010G255500 Potri.014G025300 Potri.010G254700 Potri.003G175700 Potri.005G218300 Potri.T058000 Potri.010G027800 Potri.005G086500 Potri.004G162400 Potri.002G257900 Potri.005G206700 Potri.008G151600 Potri.014G038300 Potri.003G207200 Potri.017G134900 Potri.017G081000 Potri.011G079500 Potri.013G039000 Potri.003G125400 Potri.001G079600 Potri.008G157400 Potri.015G105000 Potri.015G141200 Potri.001G407100 Potri.016G126300 Potri.001G106100 Potri.009G062500 Potri.011G045600 Potri.001G075500 Potri.017G081300 Potri.001G465800 Potri.011G079600 Potri.001G048200 Potri.015G135400 Potri.014G029700 Potri.001G018700 Potri.017G017500 Potri.003G126800 Potri.001G155700 Potri.004G005300 Potri.019G050200 Potri.007G007600 Potri.013G117000 Potri.018G022300 Potri.004G055700 Potri.013G100700 Potri.006G229300 Potri.012G071100 Potri.014G106000 Potri.001G079900 Potri.015G074600 Potri.012G128200 Potri.003G083100 Potri.002G055700 Potri.005G068500 Potri.012G114600 Potri.001G262400 Potri.006G071200 Potri.005G155700 Potri.003G088700 Potri.008G172400 Potri.003G156500 Potri.002G180300 Potri.012G112300 Potri.009G100200 Potri.001G317400 Potri.012G063800 Potri.018G139800 Potri.005G020500 Potri.010G029100 Potri.006G117200 Potri.013G025900 Potri.005G079700 Potri.005G139500 Potri.002G055400 Potri.003G205400 Potri.017G016700 Potri.001G386000 Potri.001G229700 Potri.004G175800 Potri.005G055300 Potri.015G043400 Potri.012G119800 Potri.017G130600 Potri.003G115300 Potri.006G114400 Potri.008G081000 Potri.018G065200 Potri.005G239900

			Potri.003G148900 Potri.012G058700 Potri.007G023600 Potri.005G239200 Potri.006G260100
			Potri.006G235100 Potri.006G104300 Potri.018G091200 Potri.011G031300 Potri.010G167600
			Potri.005G258600 Potri.002G129800 Potri.002G253800 Potri.002G233600 Potri.003G093700
			Potri.005G172600 Potri.013G015100 Potri.002G070700 Potri.001G300700 Potri.005G188700
			Potri.003G136100 Potri.005G237300 Potri.010G197300 Potri.004G059600 Potri.015G093100
			Potri.018G103900 Potri.019G028100 Potri.013G101000 Potri.012G095600 Potri.002G034400
			Potri.005G209500 Potri.018G113400 Potri.007G033700 Potri.014G045100 Potri.004G209700
			Potri.016G116400 Potri.005G252000 Potri.001G095200 Potri.004G031500 Potri.007G008500
			Potri.008G198800 Potri.001G264000 Potri.010G247200 Potri.010G175300 Potri.001G333300
			Potri.001G320000 Potri.001G467300 Potri.002G052800 Potri.014G111400 Potri.018G028000
			Potri.018G022200 Potri.009G073800 Potri.019G043300 Potri.015G127400 Potri.013G041400
			Potri.001G081500 Potri.001G066700 Potri.003G196000 Potri.010G183400 Potri.013G026000
			Potri.001G177500 Potri.008G041000 Potri.004G212600 Potri.002G239700 Potri.019G063100
			Potri.018G026500 Potri.008G161100 Potri.011G162000 Potri.002G053000 Potri.013G156000
			Potri.005G239300 Potri.011G168700 Potri.009G124100 Potri.007G115100 Potri.005G063300
			Potri.004G021000 Potri.013G070700 Potri.010G032700 Potri.002G189300 Potri.013G082600
			Potri.006G220100 Potri.010G058200 Potri.006G099500 Potri.001G012200 Potri.012G106500
			Potri.006G035400 Potri.013G026500 Potri.005G130400 Potri.004G140100 Potri.008G161200
			Potri.012G128700 Potri.015G062200 Potri.001G471900 Potri.001G027900 Potri.004G108200
			Potri.013G048800 Potri.002G045000 Potri.002G044900 Potri.004G156000 Potri.012G106600
			Potri.005G241500 Potri.001G404700 Potri.007G105900 Potri.013G079500 Potri.006G033400
			Potri.005G257500 Potri.019G033000 Potri.004G013900 Potri.011G093700 Potri.001G191800
			Potri.008G200100 Potri.008G181900 Potri.006G228400 Potri.001G270000 Potri.018G091600
			Potri.017G115900 Potri.017G108000 Potri.001G463800 Potri.002G096700 Potri.016G134600
			Potri.008G087800 Potri.002G009300 Potri.011G164800 Potri.001G234500 Potri.008G084500
			Potri.003G056900 Potri.019G091900 Potri.001G126100 Potri.002G143300 Potri.002G143400
			Potri.007G007400 Potri.008G067300 Potri.016G070500 Potri.013G025700 Potri.008G159400
			Potri.014G172400 Potri.008G035500 Potri.006G254200 Potri.006G255600 Potri.002G030900
			Potri.010G125300 Potri.001G246400 Potri.004G084000 Potri.018G096100 Potri.012G068800
			Potri.010G093400 Potri.006G051700 Potri.001G056700 Potri.004G001900 Potri.T171800 Potri.004G121800
			Potri.013G080300 Potri.006G165600 Potri.014G146100 Potri.005G218800 Potri.002G114800
			Potri.001G237200 Potri.013G155900 Potri.016G036900 Potri.014G165100 Potri.008G204400
			Potri.010G125400 Potri.012G047700 Potri.005G218200 Potri.019G036000 Potri.003G020400
			Potri.013G041300 Potri.008G204900 Potri.011G050800 Potri.001G331600 Potri.014G198400

			Potri.003G183100 Potri.010G089400 Potri.015G112200 Potri.001G448400 Potri.003G171500 Potri.007G121500 Potri.005G027900 Potri.012G091700 Potri.003G093200 Potri.001G071000 Potri.002G082200 Potri.013G075400 Potri.005G053900 Potri.013G128200 Potri.016G144100 Potri.011G033400 Potri.007G046900 Potri.002G242700 Potri.018G053600 Potri.013G095500 Potri.001G467100 Potri.011G069600 Potri.005G036600 Potri.014G100100 Potri.001G372300 Potri.016G051600 Potri.002G200300 Potri.005G067000 Potri.008G038900 Potri.006G166600 Potri.011G067500 Potri.004G183100 Potri.006G181900 Potri.002G256400 Potri.002G021800 Potri.004G150300 Potri.016G055400 Potri.006G167700 Potri.013G152400 Potri.011G150700 Potri.019G033900 Potri.012G127800 Potri.002G003400 Potri.009G049400 Potri.010G137300 Potri.001G384700 Potri.006G144000 Potri.015G034100 Potri.008G116800 Potri.005G155300 Potri.001G074700 Potri.002G107800 Potri.011G155500 Potri.010G221100 Potri.010G065200 Potri.004G138400 Potri.013G082700 Potri.003G159700 Potri.008G002900
GO:0044264	cellular polysaccharide metabolic process	18	Potri.001G071000 Potri.011G069600 Potri.001G320000 Potri.001G012200 Potri.018G103900 Potri.003G159700 Potri.012G128200 Potri.002G257900 Potri.007G008500 Potri.002G200300 Potri.014G125100 Potri.004G021000 Potri.014G146100 Potri.004G059600 Potri.006G181900 Potri.013G152400 Potri.001G237200 Potri.006G071200
GO:0044267	cellular protein metabolic process	111	Potri.009G139400 Potri.001G467300 Potri.013G112900 Potri.007G009200 Potri.001G246400 Potri.007G046900 Potri.011G139800 Potri.018G096100 Potri.006G051700 Potri.002G070900 Potri.012G128700 Potri.017G108000 Potri.006G130000 Potri.013G156000 Potri.010G183400 Potri.009G100200 Potri.008G204400 Potri.016G142800 Potri.004G212600 Potri.006G117200 Potri.005G079700 Potri.005G139500 Potri.003G107600 Potri.001G467100 Potri.001G066700 Potri.003G183100 Potri.006G235000 Potri.002G137700 Potri.003G205400 Potri.006G220100 Potri.005G086500 Potri.005G027900 Potri.001G386000 Potri.010G120100 Potri.003G175700 Potri.001G331600 Potri.012G119800 Potri.T058000 Potri.017G130600 Potri.016G144100 Potri.011G033400 Potri.003G115300 Potri.004G140100 Potri.002G242700 Potri.006G114400 Potri.004G108200 Potri.013G048800 Potri.014G038300 Potri.012G058700 Potri.006G260100 Potri.005G036600 Potri.017G134900 Potri.016G051600 Potri.012G106600 Potri.005G241500 Potri.001G404700 Potri.013G039000 Potri.006G104300 Potri.018G091200 Potri.006G033400 Potri.015G105000 Potri.015G141200 Potri.002G003400 Potri.005G257500 Potri.012G095600 Potri.005G067000 Potri.016G126300 Potri.011G045600 Potri.004G013900 Potri.002G233600 Potri.001G075500 Potri.001G465800 Potri.011G093700 Potri.005G172600 Potri.008G130000 Potri.016G114400 Potri.010G058200 Potri.001G300700 Potri.017G115900 Potri.005G188700 Potri.016G055400 Potri.003G136100 Potri.002G096700 Potri.004G001900 Potri.001G018700 Potri.017G017500 Potri.011G150700 Potri.004G005300 Potri.012G127800 Potri.016G134600 Potri.015G093100

			Potri.002G009300 Potri.013G025700 Potri.018G022300 Potri.011G164800 Potri.004G084000 Potri.006G166600 Potri.012G071100 Potri.001G384700 Potri.004G209700 Potri.001G126100 Potri.011G067500 Potri.005G252000 Potri.001G095200 Potri.008G116800 Potri.003G083100 Potri.013G128200 Potri.016G070500 Potri.001G333300 Potri.006G228400 Potri.001G262400
GO:0006091	generation of precursor metabolites and energy	34	Potri.014G172400 Potri.001G056700 Potri.016G115200 Potri.005G130400 Potri.015G062200 Potri.004G162400 Potri.006G255600 Potri.003G020400 Potri.003G088700 Potri.014G029700 Potri.T099400 Potri.019G063100 Potri.013G117000 Potri.018G026500 Potri.005G239200 Potri.006G235100 Potri.011G079500 Potri.008G084500 Potri.007G033700 Potri.014G198400 Potri.005G239300 Potri.009G124100 Potri.001G407100 Potri.T171800 Potri.005G258600 Potri.010G032700 Potri.002G189300 Potri.003G171500 Potri.011G126700 Potri.008G067300 Potri.006G099500 Potri.010G221100 Potri.014G165100 Potri.008G041000
GO:0046039	GTP metabolic process	8	Potri.002G021800 Potri.017G081000 Potri.001G106100 Potri.019G036000 Potri.005G239900 Potri.003G126800 Potri.003G125400 Potri.006G035400
GO:0006629	lipid metabolic process	53	Potri.008G012800 Potri.002G219700 Potri.019G024400 Potri.018G089500 Potri.015G089700 Potri.011G050800 Potri.018G089400 Potri.001G027900 Potri.004G183100 Potri.001G463800 Potri.011G162000 Potri.001G173700 Potri.010G125300 Potri.003G073800 Potri.010G086100 Potri.010G003400 Potri.011G063800 Potri.018G088800 Potri.015G039900 Potri.010G047900 Potri.001G252900 Potri.002G034400 Potri.014G180300 Potri.001G234500 Potri.018G089300 Potri.018G088500 Potri.013G015100 Potri.013G079500 Potri.005G038800 Potri.005G242800 Potri.004G150300 Potri.019G015100 Potri.001G342600 Potri.013G070700 Potri.006G166700 Potri.003G196000 Potri.001G191400 Potri.012G128200 Potri.004G051900 Potri.T050800 Potri.012G123900 Potri.001G237200 Potri.003G093700 Potri.001G317400 Potri.011G079600 Potri.008G204900 Potri.014G014400 Potri.015G043400 Potri.016G117500 Potri.001G320000 Potri.010G125400 Potri.001G263700 Potri.019G024800
GO:0043170	macromolecule metabolic process	292	Potri.014G099900 Potri.009G139400 Potri.008G035500 Potri.014G106000 Potri.013G112900 Potri.007G009200 Potri.005G155700 Potri.003G164000 Potri.018G028000 Potri.011G155400 Potri.003G088700 Potri.009G031900 Potri.002G030900 Potri.004G140100 Potri.011G139800 Potri.002G114800 Potri.008G172400 Potri.019G043300 Potri.018G096100 Potri.015G127400 Potri.012G068800 Potri.003G156500 Potri.013G041400 Potri.006G153300 Potri.010G093400 Potri.014G125100 Potri.013G059600 Potri.006G069400 Potri.012G105500 Potri.002G070900 Potri.012G128700 Potri.005G256000 Potri.017G108000 Potri.006G130000 Potri.008G081000 Potri.005G063000 Potri.001G440300 Potri.019G131300 Potri.001G312800 Potri.013G155900 Potri.010G183400 Potri.014G146100 Potri.018G111000 Potri.006G204700 Potri.001G237200 Potri.018G068700 Potri.006G076200 Potri.016G024500 Potri.002G180300 Potri.009G100200

			Potri.001G291800 Potri.005G067000 Potri.014G139300 Potri.008G204400 Potri.008G061000
			Potri.001G079600 Potri.010G220200 Potri.001G177500 Potri.014G111400 Potri.016G142800
			Potri.016G000600 Potri.014G074600 Potri.001G448400 Potri.012G047700 Potri.012G031100
			Potri.007G023600 Potri.009G124100 Potri.001G467100 Potri.002G108000 Potri.012G063800
			Potri.002G096700 Potri.002G055400 Potri.001G467300 Potri.005G020500 Potri.011G033400
			Potri.001G246400 Potri.014G018900 Potri.011G076700 Potri.006G117200 Potri.002G120400
			Potri.002G045000 Potri.014G038300 Potri.013G025900 Potri.018G143400 Potri.013G041300
			Potri.005G207200 Potri.001G065900 Potri.015G141200 Potri.005G079700 Potri.008G161100
			Potri.001G048200 Potri.005G139500 Potri.006G166600 Potri.T161200 Potri.003G107600 Potri.018G014500
			Potri.013G156000 Potri.003G183100 Potri.006G235000 Potri.002G104600 Potri.018G105700
			Potri.006G260100 Potri.001G155100 Potri.007G115100 Potri.001G374200 Potri.012G126500
			Potri.011G067500 Potri.004G021000 Potri.002G137700 Potri.003G205400 Potri.006G220100
			Potri.005G086500 Potri.013G143100 Potri.005G027900 Potri.001G386000 Potri.001G229700
			Potri.018G091600 Potri.004G175800 Potri.010G120100 Potri.001G290800 Potri.013G120200
			Potri.005G055300 Potri.003G175700 Potri.003G093200 Potri.006G183200 Potri.006G073800
			Potri.001G071000 Potri.002G082200 Potri.005G252000 Potri.013G075400 Potri.005G218300
			Potri.005G053900 Potri.017G131800 Potri.012G119800 Potri.004G207600 Potri.T058000 Potri.017G130600
			Potri.004G212600 Potri.016G144100 Potri.006G141200 Potri.003G115300 Potri.003G056900
			Potri.008G161200 Potri.004G173900 Potri.014G146600 Potri.002G242700 Potri.006G114400
			Potri.004G162400 Potri.004G121800 Potri.002G257900 Potri.005G241500 Potri.018G053600
			Potri.001G074700 Potri.004G108200 Potri.013G048800 Potri.003G071800 Potri.015G104700
			Potri.019G002100 Potri.004G183500 Potri.012G058700 Potri.003G207200 Potri.015G142000
			Potri.011G066900 Potri.011G069600 Potri.005G036600 Potri.014G100100 Potri.017G134900
			Potri.001G372300 Potri.002G044900 Potri.004G156000 Potri.016G051600 Potri.001G331600
			Potri.012G106600 Potri.002G200300 Potri.006G235100 Potri.001G404700 Potri.004G085000
			Potri.013G039000 Potri.006G051700 Potri.006G104300 Potri.018G091200 Potri.006G033400
			Potri.008G157400 Potri.015G105000 Potri.001G012200 Potri.018G140900 Potri.008G038900
			Potri.005G257500 Potri.010G167600 Potri.009G049400 Potri.016G126300 Potri.019G033000 Potri.T101100
			Potri.003G159700 Potri.002G129800 Potri.011G045600 Potri.014G025300 Potri.011G150700
			Potri.013G152400 Potri.004G013900 Potri.002G233600 Potri.001G075500 Potri.017G081300
			Potri.002G003400 Potri.001G465800 Potri.011G093700 Potri.012G091700 Potri.001G191800
			Potri.012G131500 Potri.005G172600 Potri.008G130000 Potri.005G091700 Potri.016G114400
			Potri.013G025700 Potri.001G270000 Potri.017G040800 Potri.002G070700 Potri.001G300700
			Potri.017G115900 Potri.005G188700 Potri.016G055400 Potri.003G136100 Potri.010G141600

			Potri.010G197300 Potri.006G167700 Potri.004G001900 Potri.001G018700 Potri.004G059600 Potri.007G046900 Potri.017G017500 Potri.010G058200 Potri.010G128200 Potri.001G450600 Potri.001G028200 Potri.010G175300 Potri.001G155700 Potri.004G005300 Potri.012G127800 Potri.005G218200 Potri.016G134600 Potri.001G066700 Potri.008G087800 Potri.010G003400 Potri.001G333300 Potri.015G093100 Potri.002G009300 Potri.018G103900 Potri.018G022300 Potri.004G055700 Potri.011G164800 Potri.004G084000 Potri.012G095600 Potri.008G084500 Potri.010G137300 Potri.012G071100 Potri.019G091900 Potri.018G014800 Potri.014G045100 Potri.002G084800 Potri.001G384700 Potri.009G141800 Potri.004G209700 Potri.001G126100 Potri.006G255200 Potri.001G079900 Potri.015G034100 Potri.001G095200 Potri.007G008500 Potri.015G074600 Potri.008G116800 Potri.017G016700 Potri.005G155300 Potri.012G128200 Potri.001G264000 Potri.002G143300 Potri.003G083100 Potri.007G007400 Potri.007G007600 Potri.011G155500 Potri.006G181900 Potri.010G065200 Potri.013G128200 Potri.016G070500 Potri.010G247200 Potri.019G033900 Potri.001G240600 Potri.006G228400 Potri.008G159400 Potri.015G051800 Potri.001G320000 Potri.001G262400 Potri.006G071200
GO:0007018	microtubule-based movement	26	Potri.006G082900 Potri.013G020700 Potri.010G153000 Potri.003G223800 Potri.002G235500 Potri.012G058400 Potri.006G085300 Potri.011G131900 Potri.T113300 Potri.010G069400 Potri.012G054400 Potri.007G014800 Potri.015G044600 Potri.001G416300 Potri.001G233700 Potri.004G031600 Potri.001G360200 Potri.006G040700 Potri.005G116900 Potri.002G110600 Potri.014G024700 Potri.009G026000 Potri.014G125700 Potri.006G210700 Potri.011G165200 Potri.002G201000
GO:0009166	nucleotide catabolic process	8	Potri.002G021800 Potri.017G081000 Potri.001G106100 Potri.019G036000 Potri.005G239900 Potri.003G126800 Potri.003G125400 Potri.006G035400
GO:0055114	oxidation reduction	112	Potri.018G112600 Potri.007G108400 Potri.006G141400 Potri.005G183000 Potri.010G139300 Potri.011G114300 Potri.012G068800 Potri.018G063300 Potri.002G083200 Potri.004G140900 Potri.007G044300 Potri.001G287400 Potri.010G236700 Potri.001G118500 Potri.012G112300 Potri.003G066600 Potri.001G237200 Potri.001G176100 Potri.016G036900 Potri.001G079000 Potri.004G134800 Potri.016G117500 Potri.005G100400 Potri.010G125400 Potri.010G050100 Potri.007G002400 Potri.003G112700 Potri.017G037900 Potri.007G018400 Potri.001G200100 Potri.008G069300 Potri.002G126100 Potri.001G401300 Potri.018G053700 Potri.015G074100 Potri.010G249600 Potri.018G015700 Potri.008G204900 Potri.011G050800 Potri.014G180300 Potri.T161200 Potri.014G029100 Potri.006G096900 Potri.014G135500 Potri.008G025500 Potri.004G150300 Potri.016G136100 Potri.017G142700 Potri.010G032700 Potri.002G107800 Potri.002G018000 Potri.005G161900 Potri.011G162000 Potri.004G175800 Potri.006G069600 Potri.005G178300 Potri.007G023300 Potri.004G156400 Potri.001G248700 Potri.001G111500 Potri.014G043100 Potri.004G010100 Potri.005G247700 Potri.006G137500 Potri.005G112900 Potri.016G025000

			Potri.010G189800 Potri.005G138400 Potri.008G161600 Potri.001G200800 Potri.001G252900 Potri.018G033400 Potri.003G219000 Potri.008G157400 Potri.018G109700 Potri.013G148900 Potri.004G018800 Potri.004G183100 Potri.008G158300 Potri.014G140500 Potri.003G093700 Potri.004G156500 Potri.018G051300 Potri.011G079600 Potri.013G079500 Potri.015G135400 Potri.007G122100 Potri.001G463800 Potri.002G214800 Potri.005G254100 Potri.005G124000 Potri.014G111600 Potri.007G007600 Potri.009G081300 Potri.002G220400 Potri.006G229300 Potri.002G034400 Potri.001G015400 Potri.007G026500 Potri.016G112000 Potri.006G027300 Potri.015G074600 Potri.003G039000 Potri.006G248000 Potri.012G128200 Potri.007G038300 Potri.013G125300 Potri.013G027000 Potri.018G076600 Potri.011G120200 Potri.006G199100 Potri.001G320000
GO:0006793	phosphorus metabolic process	99	Potri.009G139400 Potri.001G467300 Potri.007G009200 Potri.002G052800 Potri.001G246400 Potri.011G139800 Potri.018G096100 Potri.006G051700 Potri.002G070900 Potri.012G128700 Potri.017G108000 Potri.006G130000 Potri.T171800 Potri.013G156000 Potri.010G183400 Potri.006G255600 Potri.008G204400 Potri.016G142800 Potri.004G212600 Potri.006G117200 Potri.018G026500 Potri.005G139500 Potri.003G107600 Potri.001G066700 Potri.003G183100 Potri.006G235000 Potri.015G112200 Potri.002G137700 Potri.003G205400 Potri.006G220100 Potri.005G086500 Potri.007G121500 Potri.001G386000 Potri.010G120100 Potri.003G175700 Potri.T058000 Potri.017G130600 Potri.010G027800 Potri.016G144100 Potri.011G033400 Potri.007G046900 Potri.002G242700 Potri.006G114400 Potri.004G108200 Potri.013G048800 Potri.014G038300 Potri.006G260100 Potri.005G036600 Potri.017G134900 Potri.016G051600 Potri.005G241500 Potri.013G039000 Potri.006G104300 Potri.018G091200 Potri.015G105000 Potri.015G141200 Potri.002G003400 Potri.005G257500 Potri.012G095600 Potri.005G067000 Potri.016G126300 Potri.011G045600 Potri.002G233600 Potri.001G465800 Potri.011G093700 Potri.008G130000 Potri.016G114400 Potri.010G058200 Potri.001G300700 Potri.017G115900 Potri.005G188700 Potri.016G055400 Potri.003G136100 Potri.004G001900 Potri.001G018700 Potri.004G005300 Potri.012G127800 Potri.016G134600 Potri.015G093100 Potri.002G009300 Potri.013G025700 Potri.013G117000 Potri.018G022300 Potri.011G164800 Potri.004G084000 Potri.006G166600 Potri.012G071100 Potri.001G384700 Potri.004G209700 Potri.001G126100 Potri.011G067500 Potri.005G252000 Potri.001G095200 Potri.008G116800 Potri.002G143400 Potri.016G070500 Potri.001G333300 Potri.006G228400 Potri.012G114600
GO:0016310	phosphorylation	98	Potri.009G139400 Potri.001G467300 Potri.007G009200 Potri.002G052800 Potri.001G246400 Potri.011G139800 Potri.018G096100 Potri.006G051700 Potri.002G070900 Potri.012G128700 Potri.017G108000 Potri.006G130000 Potri.T171800 Potri.013G156000 Potri.010G183400 Potri.006G255600 Potri.008G204400 Potri.016G142800 Potri.004G212600 Potri.006G117200 Potri.018G026500

			Potri.005G139500 Potri.003G107600 Potri.001G066700 Potri.003G183100 Potri.006G235000 Potri.015G112200 Potri.002G137700 Potri.003G205400 Potri.006G220100 Potri.005G086500 Potri.007G121500 Potri.001G386000 Potri.010G120100 Potri.003G175700 Potri.T058000 Potri.017G130600 Potri.010G027800 Potri.016G144100 Potri.011G033400 Potri.007G046900 Potri.002G242700 Potri.006G114400 Potri.004G108200 Potri.013G048800 Potri.014G038300 Potri.006G260100 Potri.005G036600 Potri.017G134900 Potri.016G051600 Potri.005G241500 Potri.013G039000 Potri.006G104300 Potri.018G091200 Potri.015G141200 Potri.002G003400 Potri.005G257500 Potri.012G095600 Potri.005G067000 Potri.016G126300 Potri.011G045600 Potri.002G233600 Potri.001G465800 Potri.011G093700 Potri.008G130000 Potri.016G114400 Potri.010G058200 Potri.001G300700 Potri.017G115900 Potri.005G188700 Potri.016G055400 Potri.003G136100 Potri.004G001900 Potri.001G018700 Potri.004G005300 Potri.012G127800 Potri.016G134600 Potri.015G093100 Potri.002G009300 Potri.013G025700 Potri.013G117000 Potri.018G022300 Potri.011G164800 Potri.004G084000 Potri.006G166600 Potri.012G071100 Potri.001G384700 Potri.004G209700 Potri.001G126100 Potri.011G067500 Potri.005G252000 Potri.001G095200 Potri.008G116800 Potri.002G143400 Potri.016G070500 Potri.001G333300 Potri.006G228400 Potri.012G114600
GO:0015979	photosynthesis	56	Potri.014G172400 Potri.016G115200 Potri.014G029700 Potri.015G062200 Potri.006G254200 Potri.018G065200 Potri.005G063300 Potri.002G256400 Potri.005G206700 Potri.003G148900 Potri.008G151600 Potri.005G239300 Potri.T099400 Potri.014G175600 Potri.007G105900 Potri.019G063100 Potri.005G130400 Potri.019G028100 Potri.012G106600 Potri.005G239200 Potri.011G079500 Potri.007G033700 Potri.014G198400 Potri.011G126700 Potri.005G218800 Potri.011G168700 Potri.001G056700 Potri.001G081500 Potri.006G144000 Potri.001G407100 Potri.010G089400 Potri.011G031300 Potri.009G049400 Potri.005G258600 Potri.004G031500 Potri.001G471900 Potri.010G032700 Potri.009G062500 Potri.002G189300 Potri.010G255500 Potri.003G171500 Potri.006G034300 Potri.010G052000 Potri.002G253800 Potri.002G055700 Potri.005G010700 Potri.008G067300 Potri.003G020400 Potri.002G239700 Potri.005G068500 Potri.010G221100 Potri.014G165100 Potri.006G099500 Potri.008G181900 Potri.008G041000 Potri.013G026500
GO:0005976	polysaccharide metabolic process	23	Potri.010G141600 Potri.014G146600 Potri.002G257900 Potri.013G152400 Potri.004G059600 Potri.002G200300 Potri.011G069600 Potri.003G159700 Potri.009G141800 Potri.014G125100 Potri.001G320000 Potri.004G021000 Potri.007G008500 Potri.T101100 Potri.014G146100 Potri.012G128200 Potri.001G237200 Potri.006G181900 Potri.001G012200 Potri.001G071000 Potri.018G103900 Potri.017G040800 Potri.006G071200
GO:0044238	primary metabolic process	411	Potri.008G012800 Potri.014G099900 Potri.009G139400 Potri.013G112900 Potri.007G009200 Potri.014G180300 Potri.001G173700 Potri.011G139800 Potri.002G007300 Potri.010G047900

			Potri.003G071800 Potri.014G125100 Potri.013G059600 Potri.006G069400 Potri.002G070900
			Potri.006G130000 Potri.006G255200 Potri.019G131300 Potri.011G044300 Potri.001G191400
			Potri.012G123900 Potri.018G068700 Potri.006G076200 Potri.008G061000 Potri.016G142800
			Potri.016G000600 Potri.014G074600 Potri.001G263700 Potri.012G031100 Potri.019G024400
			Potri.002G108000 Potri.001G144800 Potri.008G130000 Potri.018G143400 Potri.010G249600
			Potri.005G207200 Potri.016G114400 Potri.T161200 Potri.003G107600 Potri.010G125800 Potri.016G024500
			Potri.002G104600 Potri.018G105700 Potri.001G155100 Potri.005G038800 Potri.001G374200
			Potri.012G126500 Potri.013G080400 Potri.005G059500 Potri.013G143100 Potri.010G120100
			Potri.013G120200 Potri.010G254700 Potri.003G175700 Potri.006G073800 Potri.005G218300
			Potri.004G207600 Potri.T058000 Potri.014G139300 Potri.017G016700 Potri.004G162400 Potri.018G089400
			Potri.002G257900 Potri.014G014400 Potri.015G104700 Potri.014G038300 Potri.003G207200
			Potri.017G134900 Potri.017G081000 Potri.001G252900 Potri.013G039000 Potri.003G125400
			Potri.001G079600 Potri.008G157400 Potri.015G105000 Potri.015G141200 Potri.002G003400
			Potri.016G126300 Potri.001G106100 Potri.011G045600 Potri.013G156000 Potri.001G075500
			Potri.017G081300 Potri.011G152400 Potri.011G079600 Potri.001G048200 Potri.015G135400
			Potri.010G141600 Potri.001G018700 Potri.017G017500 Potri.010G128200 Potri.001G450600
			Potri.003G126800 Potri.001G155700 Potri.004G005300 Potri.001G092200 Potri.019G050200
			Potri.007G007600 Potri.013G117000 Potri.018G022300 Potri.004G055700 Potri.013G100700
			Potri.006G229300 Potri.012G071100 Potri.005G063000 Potri.001G079900 Potri.015G074600
			Potri.012G128200 Potri.003G083100 Potri.002G009300 Potri.001G240600 Potri.015G051800
			Potri.001G262400 Potri.006G071200 Potri.003G164000 Potri.005G155700 Potri.011G155400
			Potri.003G088700 Potri.015G002200 Potri.008G172400 Potri.003G156500 Potri.012G105500
			Potri.002G197200 Potri.008G132700 Potri.001G440300 Potri.006G204700 Potri.009G100200
			Potri.001G291800 Potri.001G317400 Potri.011G063800 Potri.011G066900 Potri.012G063800
			Potri.018G139800 Potri.005G020500 Potri.010G029100 Potri.002G120400 Potri.013G025900
			Potri.005G079700 Potri.005G139500 Potri.002G094000 Potri.002G055400 Potri.010G008600
			Potri.003G205400 Potri.018G068600 Potri.005G086500 Potri.001G386000 Potri.001G229700
			Potri.004G175800 Potri.005G055300 Potri.015G043400 Potri.012G119800 Potri.017G130600
			Potri.019G024800 Potri.003G115300 Potri.014G146600 Potri.006G114400 Potri.008G081000
			Potri.002G180300 Potri.005G239900 Potri.012G058700 Potri.007G023600 Potri.008G063800
			Potri.018G088800 Potri.006G260100 Potri.006G235100 Potri.006G104300 Potri.018G091200
			Potri.018G140900 Potri.010G167600 Potri.002G129800 Potri.002G233600 Potri.003G093700
			Potri.012G131500 Potri.005G172600 Potri.013G015100 Potri.001G299000 Potri.002G070700
			Potri.001G300700 Potri.005G188700 Potri.003G136100 Potri.018G089500 Potri.005G120500

			Potri.001G028200	Potri.010G197300	Potri.004G059600	Potri.015G093100	Potri.006G228400	
			Potri.013G101000	Potri.012G095600	Potri.002G034400	Potri.018G113400	Potri.014G045100	
			Potri.004G209700	Potri.005G252000	Potri.005G238600	Potri.001G095200	Potri.007G008500	
			Potri.008G198800	Potri.001G264000	Potri.010G247200	Potri.010G175300	Potri.001G333300	
			Potri.001G320000	Potri.001G467300	Potri.014G111400	Potri.018G028000	Potri.009G031900	
			Potri.018G022200	Potri.009G073800	Potri.019G043300	Potri.015G039900	Potri.015G127400	
			Potri.013G041400	Potri.018G089300	Potri.003G139100	Potri.014G106000	Potri.003G196000	
			Potri.010G183400	Potri.018G111000	Potri.006G235000	Potri.001G177500	Potri.016G117500	
			Potri.004G212600	Potri.014G018900	Potri.017G130200	Potri.018G026500	Potri.008G161100	
			Potri.011G162000	Potri.001G465800	Potri.001G066700	Potri.009G124100	Potri.007G115100	
			Potri.004G021000	Potri.013G070700	Potri.006G166700	Potri.013G082600	Potri.006G220100	
			Potri.010G058200	Potri.002G137700	Potri.001G012200	Potri.006G183200	Potri.012G106500	
			Potri.006G035400	Potri.002G219700	Potri.006G141200	Potri.004G140100	Potri.008G161200	
			Potri.012G128700	Potri.004G121800	Potri.001G027900	Potri.004G108200	Potri.013G048800	
			Potri.010G093400	Potri.014G158400	Potri.018G014500	Potri.008G189200	Potri.010G042100	
			Potri.002G044900	Potri.004G156000	Potri.012G106600	Potri.005G241500	Potri.001G404700	
			Potri.009G049400	Potri.013G079500	Potri.006G033400	Potri.010G220200	Potri.005G257500	
			Potri.004G173900	Potri.001G342600	Potri.019G033000	Potri.004G051900	Potri.014G025300	Potri.T050800
			Potri.004G013900	Potri.008G100500	Potri.011G093700	Potri.001G191800	Potri.008G200100	
			Potri.001G156600	Potri.005G091700	Potri.001G270000	Potri.017G040800	Potri.018G091600	
			Potri.017G115900	Potri.001G463800	Potri.002G096700	Potri.016G134600	Potri.008G087800	
			Potri.019G015100	Potri.011G164800	Potri.001G234500	Potri.008G084500	Potri.003G056900	
			Potri.019G091900	Potri.009G141800	Potri.001G126100	Potri.005G242800	Potri.006G094400	
			Potri.002G143300	Potri.007G007400	Potri.016G070500	Potri.018G103900	Potri.008G159400	
			Potri.002G069000	Potri.002G023900	Potri.008G035500	Potri.006G255600	Potri.002G030900	
			Potri.010G125300	Potri.001G246400	Potri.004G084000	Potri.018G096100	Potri.012G068800	
			Potri.002G045000	Potri.006G051700	Potri.017G108000	Potri.004G001900	Potri.004G159800	Potri.T171800
			Potri.001G312800	Potri.013G080300	Potri.014G146100	Potri.002G114800	Potri.001G237200	
			Potri.013G155900	Potri.016G036900	Potri.008G204400	Potri.010G125400	Potri.012G047700	
			Potri.005G218200	Potri.019G036000	Potri.011G076700	Potri.003G073800	Potri.019G002100	
			Potri.013G041300	Potri.001G065900	Potri.011G050800	Potri.005G256000	Potri.001G331600	
			Potri.003G183100	Potri.001G200400	Potri.001G448400	Potri.005G027900	Potri.010G160200	
			Potri.001G290800	Potri.012G091700	Potri.011G159000	Potri.003G093200	Potri.001G071000	
			Potri.002G082200	Potri.013G075400	Potri.005G053900	Potri.017G131800	Potri.013G128200	

			Potri.004G086400 Potri.016G144100 Potri.011G033400 Potri.007G046900 Potri.002G242700<br>Potri.018G053600 Potri.001G074700 Potri.014G122200 Potri.001G467100 Potri.015G142000<br>Potri.011G069600 Potri.002G224600 Potri.014G100100 Potri.001G372300 Potri.014G126900<br>Potri.016G051600 Potri.002G200300 Potri.004G085000 Potri.005G067000 Potri.006G153300<br>Potri.008G120000 Potri.008G038900 Potri.006G166600 Potri.011G067500 Potri.T101100 Potri.004G183100<br>Potri.005G036600 Potri.003G131700 Potri.011G154100 Potri.002G021800 Potri.013G025700<br>Potri.004G150300 Potri.016G055400 Potri.015G089700 Potri.006G167700 Potri.013G152400<br>Potri.006G117200 Potri.011G150700 Potri.019G033900 Potri.012G127800 Potri.010G086100<br>Potri.010G003400 Potri.010G137300 Potri.018G014800 Potri.001G384700 Potri.015G034100<br>Potri.008G116800 Potri.005G155300 Potri.013G095500 Potri.011G155500 Potri.006G181900<br>Potri.010G065200 Potri.013G082700 Potri.003G159700 Potri.008G204900 Potri.018G088500<br>Potri.008G002900
GO:0019538	protein metabolic process	160	Potri.009G139400 Potri.001G467300 Potri.013G112900 Potri.007G009200 Potri.003G164000<br>Potri.011G155400 Potri.009G031900 Potri.007G046900 Potri.011G139800 Potri.018G096100<br>Potri.006G117200 Potri.003G071800 Potri.006G051700 Potri.012G105500 Potri.002G070900<br>Potri.012G128700 Potri.005G256000 Potri.017G108000 Potri.006G130000 Potri.005G063000<br>Potri.001G440300 Potri.001G312800 Potri.010G183400 Potri.018G111000 Potri.006G204700<br>Potri.006G076200 Potri.016G024500 Potri.009G100200 Potri.001G291800 Potri.008G204400<br>Potri.016G142800 Potri.016G000600 Potri.014G074600 Potri.004G212600 Potri.001G467100<br>Potri.011G033400 Potri.001G246400 Potri.014G018900 Potri.011G076700 Potri.002G120400<br>Potri.014G038300 Potri.018G143400 Potri.001G065900 Potri.005G079700 Potri.005G139500<br>Potri.003G107600 Potri.018G014500 Potri.013G156000 Potri.003G183100 Potri.006G235000<br>Potri.002G104600 Potri.018G105700 Potri.006G260100 Potri.002G137700 Potri.003G205400<br>Potri.006G220100 Potri.005G086500 Potri.005G027900 Potri.001G386000 Potri.010G120100<br>Potri.001G290800 Potri.013G120200 Potri.003G175700 Potri.006G183200 Potri.006G073800<br>Potri.017G131800 Potri.012G119800 Potri.004G207600 Potri.T058000 Potri.017G130600 Potri.016G144100<br>Potri.006G141200 Potri.003G115300 Potri.004G140100 Potri.004G173900 Potri.002G242700<br>Potri.006G114400 Potri.004G108200 Potri.013G048800 Potri.015G104700 Potri.019G002100<br>Potri.012G058700 Potri.015G142000 Potri.005G241500 Potri.005G036600 Potri.017G134900<br>Potri.016G051600 Potri.001G331600 Potri.012G106600 Potri.011G066900 Potri.001G404700<br>Potri.004G085000 Potri.013G039000 Potri.006G153300 Potri.006G104300 Potri.018G091200<br>Potri.006G033400 Potri.010G220200 Potri.015G105000 Potri.015G141200 Potri.018G140900<br>Potri.005G257500 Potri.012G095600 Potri.005G067000 Potri.016G126300 Potri.011G045600<br>Potri.011G150700 Potri.004G013900 Potri.002G233600 Potri.001G075500 Potri.001G465800

			Potri.011G093700 Potri.012G131500 Potri.005G172600 Potri.008G130000 Potri.005G091700 Potri.016G114400 Potri.010G058200 Potri.001G300700 Potri.017G115900 Potri.005G188700 Potri.016G055400 Potri.003G136100 Potri.002G096700 Potri.004G001900 Potri.001G018700 Potri.017G017500 Potri.010G128200 Potri.001G450600 Potri.001G028200 Potri.004G005300 Potri.012G127800 Potri.002G003400 Potri.016G134600 Potri.001G066700 Potri.010G003400 Potri.001G333300 Potri.015G093100 Potri.002G009300 Potri.013G025700 Potri.018G022300 Potri.011G164800 Potri.004G084000 Potri.006G166600 Potri.012G071100 Potri.018G014800 Potri.001G384700 Potri.004G209700 Potri.001G126100 Potri.011G067500 Potri.005G252000 Potri.001G095200 Potri.008G116800 Potri.003G083100 Potri.013G128200 Potri.016G070500 Potri.001G240600 Potri.006G228400 Potri.015G051800 Potri.001G262400
GO:0006464	protein modification process	94	Potri.009G139400 Potri.001G467300 Potri.007G009200 Potri.001G246400 Potri.004G140100 Potri.011G139800 Potri.018G096100 Potri.006G051700 Potri.002G070900 Potri.012G128700 Potri.017G108000 Potri.006G130000 Potri.013G156000 Potri.010G183400 Potri.009G100200 Potri.008G204400 Potri.016G142800 Potri.004G212600 Potri.006G117200 Potri.005G139500 Potri.003G107600 Potri.001G066700 Potri.003G183100 Potri.006G235000 Potri.002G137700 Potri.003G205400 Potri.006G220100 Potri.005G086500 Potri.001G386000 Potri.010G120100 Potri.003G175700 Potri.T058000 Potri.017G130600 Potri.016G144100 Potri.011G033400 Potri.007G046900 Potri.002G242700 Potri.006G114400 Potri.004G108200 Potri.013G048800 Potri.014G038300 Potri.006G260100 Potri.005G036600 Potri.017G134900 Potri.016G051600 Potri.012G106600 Potri.005G241500 Potri.005G067000 Potri.006G104300 Potri.018G091200 Potri.015G105000 Potri.015G141200 Potri.002G003400 Potri.005G257500 Potri.006G166600 Potri.011G067500 Potri.016G126300 Potri.011G045600 Potri.004G013900 Potri.002G233600 Potri.001G465800 Potri.011G093700 Potri.008G130000 Potri.016G114400 Potri.010G058200 Potri.001G300700 Potri.017G115900 Potri.005G188700 Potri.016G055400 Potri.003G136100 Potri.004G001900 Potri.001G018700 Potri.013G039000 Potri.004G005300 Potri.012G127800 Potri.016G134600 Potri.015G093100 Potri.002G009300 Potri.013G025700 Potri.018G022300 Potri.011G164800 Potri.004G084000 Potri.012G095600 Potri.012G071100 Potri.001G384700 Potri.004G209700 Potri.001G126100 Potri.005G252000 Potri.001G095200 Potri.008G116800 Potri.003G083100 Potri.016G070500 Potri.001G333300 Potri.006G228400
GO:0080090	regulation of primary metabolic process	84	Potri.014G099900 Potri.010G197300 Potri.002G108000 Potri.008G161200 Potri.008G161100 Potri.017G016700 Potri.004G121800 Potri.005G020500 Potri.008G157400 Potri.005G155700 Potri.014G111400 Potri.018G028000 Potri.002G045000 Potri.001G155700 Potri.013G075400 Potri.010G065200 Potri.008G087800 Potri.003G207200 Potri.002G114800 Potri.002G143300 Potri.005G207200 Potri.019G043300 Potri.002G044900 Potri.015G127400 Potri.004G055700

			Potri.004G156000 Potri.013G041400 Potri.008G172400 Potri.010G093400 Potri.013G059600 Potri.006G069400 Potri.005G155300 Potri.006G167700 Potri.009G049400 Potri.003G056900 Potri.019G091900 Potri.007G023600 Potri.014G045100 Potri.001G079600 Potri.005G053900 Potri.001G155100 Potri.004G209700 Potri.007G115100 Potri.014G106000 Potri.001G374200 Potri.008G038900 Potri.012G126500 Potri.010G167600 Potri.001G079900 Potri.006G255200 Potri.015G034100 Potri.002G180300 Potri.019G131300 Potri.013G041300 Potri.012G047700 Potri.002G129800 Potri.003G093200 Potri.014G100100 Potri.019G033000 Potri.001G229700 Potri.007G007400 Potri.001G372300 Potri.018G068700 Potri.018G053600 Potri.017G081300 Potri.014G025300 Potri.005G055300 Potri.001G177500 Potri.001G191800 Potri.002G055400 Potri.010G247200 Potri.012G063800 Potri.008G061000 Potri.005G218200 Potri.002G082200 Potri.001G048200 Potri.002G030900 Potri.005G218300 Potri.001G270000 Potri.001G448400 Potri.002G070700 Potri.018G091600 Potri.013G025900 Potri.012G031100
GO:0006979	response to oxidative stress	7	Potri.017G037900 Potri.006G069600 Potri.004G134800 Potri.005G100400 Potri.002G018000 Potri.005G161900 Potri.007G122100
GO:0023052	signaling	13	Potri.009G162300 Potri.011G061500 Potri.011G033400 Potri.014G102200 Potri.002G176800 Potri.011G067500 Potri.014G106000 Potri.T106300 Potri.002G082200 Potri.013G156000 Potri.001G275200 Potri.014G103600 Potri.008G061300
GO:0055085	transmembrane transport	52	Potri.005G255400 Potri.012G047200 Potri.016G115500 Potri.006G255600 Potri.008G127700 Potri.016G103500 Potri.001G331700 Potri.001G139600 Potri.005G245900 Potri.003G215600 Potri.002G019600 Potri.005G102800 Potri.013G117000 Potri.009G006400 Potri.018G026500 Potri.013G108200 Potri.005G037300 Potri.017G081100 Potri.005G037000 Potri.017G116400 Potri.009G045800 Potri.T171800 Potri.015G072600 Potri.009G138900 Potri.006G099900 Potri.001G354900 Potri.001G348300 Potri.001G124200 Potri.009G061100 Potri.012G077400 Potri.009G151400 Potri.005G040000 Potri.010G003000 Potri.009G010700 Potri.015G038700 Potri.008G129400 Potri.008G017100 Potri.003G018800 Potri.003G094400 Potri.002G211100 Potri.001G160400 Potri.001G251200 Potri.012G070300 Potri.006G060900 Potri.006G054700 Potri.008G000300 Potri.010G112800 Potri.012G144000 Potri.001G068600 Potri.002G092400 Potri.011G002400 Potri.010G111700
**Function go term**	**Description**	**Matched counts**	**Matched genes**
GO:0022804	active transmembrane transporter activity	17	Potri.002G019600 Potri.008G017100 Potri.003G094400 Potri.008G186600 Potri.010G046300 Potri.018G139800 Potri.008G000300 Potri.005G102800 Potri.006G054700 Potri.009G061100 Potri.001G354900 Potri.012G081800 Potri.001G139600 Potri.014G116000 Potri.010G003000 Potri.017G081100 Potri.011G002400

GO:0030554	adenyl nucleotide binding	148	Potri.009G139400 Potri.009G073800 Potri.018G074500 Potri.005G064300 Potri.001G467300 Potri.007G009200 Potri.001G246400 Potri.011G139800 Potri.011G131900 Potri.003G094400 Potri.018G096100 Potri.012G068800 Potri.006G051700 Potri.002G070900 Potri.015G044600 Potri.012G128700 Potri.017G108000 Potri.006G130000 Potri.004G031600 Potri.001G066700 Potri.010G183400 Potri.003G183100 Potri.007G014800 Potri.010G200500 Potri.001G079000 Potri.010G188200 Potri.008G204400 Potri.016G142800 Potri.004G212600 Potri.006G082900 Potri.004G061700 Potri.003G223800 Potri.002G235500 Potri.006G117200 Potri.006G085300 Potri.002G019600 Potri.005G139500 Potri.006G166600 Potri.T161200 Potri.003G107600 Potri.013G156000 Potri.017G081100 Potri.006G235000 Potri.014G125700 Potri.002G137700 Potri.003G205400 Potri.006G220100 Potri.005G086500 Potri.016G078600 Potri.014G024700 Potri.010G120100 Potri.012G091700 Potri.003G175700 Potri.002G201000 Potri.005G067000 Potri.001G111500 Potri.T058000 Potri.017G130600 Potri.010G027800 Potri.016G144100 Potri.011G033400 Potri.007G046900 Potri.002G242700 Potri.006G114400 Potri.004G108200 Potri.013G048800 Potri.001G139600 Potri.014G038300 Potri.006G260100 Potri.005G036600 Potri.T113300 Potri.017G134900 Potri.001G200800 Potri.016G051600 Potri.005G241500 Potri.001G037300 Potri.013G039000 Potri.006G104300 Potri.018G091200 Potri.001G233700 Potri.015G141200 Potri.007G080600 Potri.010G069400 Potri.012G095600 Potri.009G049400 Potri.016G126300 Potri.001G354900 Potri.006G040700 Potri.010G153000 Potri.005G116900 Potri.011G045600 Potri.002G110600 Potri.009G026000 Potri.002G233600 Potri.001G360200 Potri.001G465800 Potri.011G093700 Potri.004G236500 Potri.013G020700 Potri.008G130000 Potri.016G114400 Potri.010G058200 Potri.001G300700 Potri.017G115900 Potri.005G188700 Potri.016G055400 Potri.003G136100 Potri.004G001900 Potri.001G018700 Potri.012G058400 Potri.004G005300 Potri.012G127800 Potri.002G003400 Potri.016G134600 Potri.001G134000 Potri.015G093100 Potri.007G007600 Potri.013G025700 Potri.018G022300 Potri.005G257500 Potri.011G164800 Potri.004G084000 Potri.012G054400 Potri.018G113400 Potri.012G071100 Potri.001G416300 Potri.001G384700 Potri.004G209700 Potri.001G126100 Potri.011G067500 Potri.005G252000 Potri.005G238600 Potri.001G095200 Potri.017G142700 Potri.015G074600 Potri.003G039000 Potri.008G116800 Potri.001G264000 Potri.010G003000 Potri.002G009300 Potri.006G210700 Potri.016G070500 Potri.009G160600 Potri.011G165200 Potri.001G333300 Potri.006G228400 Potri.014G139300 Potri.T100800
GO:0016209	antioxidant activity	10	Potri.006G137500 Potri.012G112300 Potri.017G037900 Potri.006G069600 Potri.004G134800 Potri.018G063300 Potri.005G100400 Potri.002G018000 Potri.005G161900 Potri.007G122100
GO:0070001	aspartic-type peptidase activity	14	Potri.006G204700 Potri.005G063000 Potri.001G240600 Potri.010G128200 Potri.015G051800 Potri.001G028200 Potri.016G000600 Potri.017G131800 Potri.004G085000 Potri.019G002100 Potri.018G014800 Potri.002G104600 Potri.018G014500 Potri.010G003400

GO:0005524	ATP binding	139	Potri.009G139400 Potri.009G073800 Potri.018G074500 Potri.005G064300 Potri.001G467300 Potri.007G009200 Potri.001G246400 Potri.011G139800 Potri.011G131900 Potri.003G094400 Potri.018G096100 Potri.014G139300 Potri.006G051700 Potri.002G070900 Potri.015G044600 Potri.012G128700 Potri.017G108000 Potri.006G130000 Potri.004G031600 Potri.013G156000 Potri.010G183400 Potri.007G014800 Potri.010G200500 Potri.010G188200 Potri.008G204400 Potri.016G142800 Potri.004G212600 Potri.006G082900 Potri.004G061700 Potri.003G223800 Potri.002G235500 Potri.006G117200 Potri.006G085300 Potri.002G019600 Potri.005G139500 Potri.006G166600 Potri.017G081100 Potri.003G107600 Potri.001G066700 Potri.003G183100 Potri.006G235000 Potri.014G125700 Potri.002G137700 Potri.003G205400 Potri.006G220100 Potri.005G086500 Potri.016G078600 Potri.014G024700 Potri.010G120100 Potri.012G091700 Potri.003G175700 Potri.002G201000 Potri.005G067000 Potri.T058000 Potri.017G130600 Potri.010G027800 Potri.016G144100 Potri.011G033400 Potri.007G046900 Potri.002G242700 Potri.006G114400 Potri.004G108200 Potri.013G048800 Potri.001G139600 Potri.014G038300 Potri.006G260100 Potri.005G036600 Potri.T113300 Potri.017G134900 Potri.016G051600 Potri.005G241500 Potri.001G037300 Potri.013G039000 Potri.006G104300 Potri.018G091200 Potri.001G233700 Potri.015G141200 Potri.007G080600 Potri.010G069400 Potri.012G095600 Potri.009G049400 Potri.016G126300 Potri.001G354900 Potri.006G040700 Potri.010G153000 Potri.005G116900 Potri.011G045600 Potri.002G110600 Potri.009G026000 Potri.002G233600 Potri.001G360200 Potri.001G465800 Potri.011G093700 Potri.004G236500 Potri.013G020700 Potri.008G130000 Potri.016G114400 Potri.010G058200 Potri.001G300700 Potri.017G115900 Potri.005G188700 Potri.016G055400 Potri.003G136100 Potri.004G001900 Potri.001G018700 Potri.012G058400 Potri.004G005300 Potri.012G127800 Potri.002G003400 Potri.016G134600 Potri.001G134000 Potri.015G093100 Potri.002G009300 Potri.013G025700 Potri.018G022300 Potri.005G257500 Potri.011G164800 Potri.004G084000 Potri.012G054400 Potri.018G113400 Potri.012G071100 Potri.001G416300 Potri.001G384700 Potri.004G209700 Potri.001G126100 Potri.011G067500 Potri.005G252000 Potri.005G238600 Potri.001G095200 Potri.008G116800 Potri.001G264000 Potri.010G003000 Potri.006G210700 Potri.016G070500 Potri.009G160600 Potri.011G165200 Potri.001G333300 Potri.006G228400 Potri.T100800
GO:0005509	calcium ion binding	12	Potri.005G068500 Potri.005G130400 Potri.011G031300 Potri.004G031500 Potri.009G062500 Potri.005G206700 Potri.007G092500 Potri.013G026500 Potri.007G033700 Potri.006G034300 Potri.002G055700 Potri.005G010700
GO:0004091	carboxylesterase activity	15	Potri.006G134500 Potri.003G122000 Potri.003G086600 Potri.011G135000 Potri.003G076900 Potri.015G013700 Potri.007G107300 Potri.015G128400 Potri.003G073800 Potri.010G109400 Potri.008G132600 Potri.006G137800 Potri.001G173700 Potri.014G117100 Potri.012G014500

GO:0004180	carboxypeptidase activity	12	Potri.010G220200 Potri.003G164000 Potri.013G120200 Potri.001G291800 Potri.001G065900 Potri.006G183200 Potri.005G091700 Potri.001G312800 Potri.001G290800 Potri.012G105500 Potri.015G104700 Potri.018G105700
GO:0050662	coenzyme binding	28	Potri.002G107800 Potri.010G249600 Potri.007G007600 Potri.008G204900 Potri.001G200800 Potri.T161200 Potri.002G034400 Potri.005G209500 Potri.002G053000 Potri.013G079500 Potri.004G028900 Potri.004G150300 Potri.017G142700 Potri.015G074600 Potri.003G039000 Potri.012G128200 Potri.004G183100 Potri.001G237200 Potri.013G026000 Potri.003G093700 Potri.001G079000 Potri.011G079600 Potri.001G111500 Potri.015G135400 Potri.001G320000 Potri.010G125400 Potri.002G179500 Potri.012G068800
GO:0030234	enzyme regulator activity	16	Potri.006G134500 Potri.003G122000 Potri.003G086600 Potri.006G092600 Potri.011G135000 Potri.015G013700 Potri.007G107300 Potri.015G128400 Potri.010G109400 Potri.005G137500 Potri.002G234600 Potri.008G132600 Potri.006G137800 Potri.016G104400 Potri.012G014500 Potri.009G140100
GO:0050660	FAD binding	9	Potri.007G007600 Potri.001G079000 Potri.001G200800 Potri.017G142700 Potri.015G074600 Potri.T161200 Potri.003G039000 Potri.001G111500 Potri.012G068800
GO:0046527	glucosyltransferase activity	8	Potri.011G069600 Potri.006G181900 Potri.001G012200 Potri.018G103900 Potri.002G257900 Potri.002G200300 Potri.014G125100 Potri.004G059600
GO:0020037	heme binding	29	Potri.007G002400 Potri.017G037900 Potri.007G018400 Potri.001G200100 Potri.002G126100 Potri.006G141400 Potri.010G189800 Potri.005G124000 Potri.010G139300 Potri.014G029100 Potri.004G140900 Potri.001G118500 Potri.007G026500 Potri.010G236700 Potri.008G025500 Potri.004G018800 Potri.002G018000 Potri.005G161900 Potri.014G043100 Potri.003G066600 Potri.013G125300 Potri.006G069600 Potri.013G027000 Potri.018G051300 Potri.009G110800 Potri.004G134800 Potri.005G100400 Potri.010G050100 Potri.007G122100
GO:0016787	hydrolase activity	204	Potri.003G164000 Potri.011G135000 Potri.018G074500 Potri.005G064300 Potri.007G014800 Potri.004G051900 Potri.001G173700 Potri.011G155400 Potri.003G080400 Potri.012G042200 Potri.009G031900 Potri.011G131900 Potri.002G007300 Potri.010G047900 Potri.003G156500 Potri.003G071800 Potri.003G139100 Potri.012G105500 Potri.001G354900 Potri.002G094000 Potri.015G044600 Potri.003G122000 Potri.004G159800 Potri.002G197200 Potri.004G031600 Potri.008G100500 Potri.008G132700 Potri.005G063000 Potri.001G440300 Potri.011G044300 Potri.001G191400 Potri.014G146100 Potri.008G189200 Potri.015G031300 Potri.018G111000 Potri.006G204700 Potri.T101100 Potri.006G076200 Potri.013G155900 Potri.004G160100 Potri.001G291800 Potri.001G317400 Potri.016G000600 Potri.014G074600 Potri.014G126900 Potri.011G063800 Potri.012G131500 Potri.019G024400 Potri.006G082900 Potri.019G036000 Potri.018G139800 Potri.003G223800 Potri.001G191000 Potri.002G235500 Potri.005G038800 Potri.011G154100

			Potri.015G104700 Potri.002G120400 Potri.017G130200 Potri.018G089500 Potri.006G085300<br>Potri.018G143400 Potri.002G021800 Potri.002G019600 Potri.001G065900 Potri.014G125700<br>Potri.011G153800 Potri.017G081100 Potri.018G089300 Potri.010G125800 Potri.005G256000<br>Potri.010G008600 Potri.016G024500 Potri.002G104600 Potri.018G105700 Potri.008G063800<br>Potri.015G142000 Potri.009G077900 Potri.006G062200 Potri.003G076900 Potri.016G042500<br>Potri.007G107300 Potri.004G021000 Potri.005G059500 Potri.002G257700 Potri.006G166700<br>Potri.018G068600 Potri.014G024700 Potri.T113300 Potri.001G290800 Potri.013G120200 Potri.008G061300<br>Potri.003G131200 Potri.011G159000 Potri.003G094400 Potri.006G183200 Potri.002G201000<br>Potri.012G106500 Potri.017G131800 Potri.006G035400 Potri.004G207600 Potri.019G024800<br>Potri.004G086400 Potri.002G219700 Potri.006G141200 Potri.014G146600 Potri.008G081000<br>Potri.018G089400 Potri.010G109400 Potri.005G239900 Potri.001G312800 Potri.014G122200<br>Potri.001G139600 Potri.019G002100 Potri.006G198800 Potri.018G014500 Potri.002G096700<br>Potri.006G134500 Potri.004G092500 Potri.002G224600 Potri.010G042100 Potri.018G088800<br>Potri.017G081000 Potri.013G070700 Potri.011G066900 Potri.008G132600 Potri.004G085000<br>Potri.001G459400 Potri.003G125400 Potri.006G153300 Potri.008G120000 Potri.001G233700<br>Potri.010G220200 Potri.015G105000 Potri.010G141600 Potri.007G080600 Potri.003G073800<br>Potri.010G069400 Potri.004G173900 Potri.001G342600 Potri.006G040700 Potri.010G153000<br>Potri.001G106100 Potri.005G116900 Potri.001G275500 Potri.015G085200 Potri.014G117100<br>Potri.002G110600 Potri.003G131700 Potri.013G152400 Potri.009G026000 Potri.012G091700<br>Potri.001G360200 Potri.011G152400 Potri.015G013700 Potri.002G211100 Potri.004G236500<br>Potri.001G156600 Potri.001G200400 Potri.005G091700 Potri.011G076700 Potri.006G137800<br>Potri.017G040800 Potri.001G299000 Potri.012G058400 Potri.014G158400 Potri.015G089700<br>Potri.013G020700 Potri.005G120500 Potri.011G165200 Potri.010G128200 Potri.001G450600<br>Potri.001G028200 Potri.003G126800 Potri.001G092200 Potri.010G003400 Potri.001G071000<br>Potri.009G160600 Potri.012G054400 Potri.018G088500 Potri.010G137300 Potri.018G014800<br>Potri.001G416300 Potri.009G141800 Potri.003G086600 Potri.005G242800 Potri.015G128400<br>Potri.007G008500 Potri.006G094400 Potri.014G018900 Potri.001G074700 Potri.010G003000<br>Potri.012G014500 Potri.006G210700 Potri.003G159700 Potri.016G065000 Potri.010G175300<br>Potri.001G240600 Potri.015G051800 Potri.002G069000 Potri.019G031200 Potri.002G023900<br>Potri.008G079400 Potri.006G071200
GO:0016798	hydrolase activity, acting on glycosyl bonds	50	Potri.004G086400 Potri.014G158400 Potri.014G146100 Potri.014G146600 Potri.010G141600<br>Potri.005G120500 Potri.002G023900 Potri.010G137300 Potri.011G154100 Potri.014G122200<br>Potri.017G130200 Potri.001G092200 Potri.008G189200 Potri.003G159700 Potri.002G224600<br>Potri.010G042100 Potri.002G007300 Potri.013G152400 Potri.003G156500 Potri.009G141800

			Potri.002G094000 Potri.010G008600 Potri.010G125800 Potri.008G120000 Potri.003G139100 Potri.004G159800 Potri.002G197200 Potri.001G200400 Potri.008G081000 Potri.008G132700 Potri.006G062200 Potri.004G021000 Potri.005G059500 Potri.T101100 Potri.014G126900 Potri.011G044300 Potri.018G068600 Potri.001G074700 Potri.003G131700 Potri.007G008500 Potri.008G100500 Potri.011G152400 Potri.011G159000 Potri.010G175300 Potri.001G071000 Potri.012G106500 Potri.017G040800 Potri.001G299000 Potri.008G079400 Potri.006G071200
GO:0005506	iron ion binding	37	Potri.007G002400 Potri.001G463800 Potri.017G037900 Potri.007G018400 Potri.001G200100 Potri.002G126100 Potri.006G141400 Potri.010G189800 Potri.005G124000 Potri.010G139300 Potri.013G035700 Potri.014G180300 Potri.014G029100 Potri.004G140900 Potri.016G124700 Potri.008G025500 Potri.007G026500 Potri.010G236700 Potri.001G118500 Potri.017G142700 Potri.011G162000 Potri.006G165600 Potri.004G018800 Potri.002G018000 Potri.005G161900 Potri.014G043100 Potri.003G066600 Potri.013G125300 Potri.006G069600 Potri.001G079000 Potri.018G051300 Potri.009G110800 Potri.004G134800 Potri.013G027000 Potri.005G100400 Potri.010G050100 Potri.007G122100
GO:0003777	microtubule motor activity	26	Potri.006G082900 Potri.013G020700 Potri.010G153000 Potri.003G223800 Potri.002G235500 Potri.012G058400 Potri.006G085300 Potri.011G131900 Potri.T113300 Potri.010G069400 Potri.012G054400 Potri.007G014800 Potri.015G044600 Potri.001G416300 Potri.001G233700 Potri.004G031600 Potri.001G360200 Potri.006G040700 Potri.005G116900 Potri.002G110600 Potri.014G024700 Potri.009G026000 Potri.014G125700 Potri.006G210700 Potri.011G165200 Potri.002G201000
GO:0017111	nucleoside-triphosphatase activity	48	Potri.007G014800 Potri.006G082900 Potri.018G074500 Potri.005G064300 Potri.013G020700 Potri.018G139800 Potri.011G165200 Potri.002G235500 Potri.005G239900 Potri.003G126800 Potri.001G139600 Potri.006G085300 Potri.002G021800 Potri.011G131900 Potri.002G019600 Potri.003G094400 Potri.017G081000 Potri.T113300 Potri.012G054400 Potri.003G125400 Potri.017G081100 Potri.015G044600 Potri.001G416300 Potri.001G233700 Potri.007G080600 Potri.004G031600 Potri.001G360200 Potri.001G354900 Potri.006G040700 Potri.010G153000 Potri.001G106100 Potri.005G116900 Potri.002G110600 Potri.014G024700 Potri.009G026000 Potri.014G125700 Potri.012G091700 Potri.006G210700 Potri.010G069400 Potri.008G061300 Potri.019G036000 Potri.009G160600 Potri.004G236500 Potri.002G201000 Potri.006G035400 Potri.003G223800 Potri.010G003000 Potri.012G058400
GO:0016491	oxidoreductase activity	131	Potri.008G012800 Potri.018G112600 Potri.007G108400 Potri.001G403300 Potri.002G073000 Potri.006G213000 Potri.006G141400 Potri.005G183000 Potri.010G139300 Potri.015G039900 Potri.012G068800 Potri.018G063300 Potri.002G083200 Potri.004G140900 Potri.007G044300 Potri.001G287400 Potri.010G236700 Potri.001G118500 Potri.013G026000 Potri.003G066600 Potri.001G237200 Potri.007G074000 Potri.012G112300 Potri.016G036900 Potri.001G079000

			Potri.004G134800 Potri.006G206800 Potri.016G117500 Potri.005G100400 Potri.010G125400 Potri.010G050100 Potri.002G179500 Potri.007G002400 Potri.003G112700 Potri.017G037900 Potri.007G018400 Potri.001G200100 Potri.008G069300 Potri.002G126100 Potri.001G401300 Potri.018G053700 Potri.003G141800 Potri.015G074100 Potri.010G249600 Potri.018G015700 Potri.008G204900 Potri.011G050800 Potri.013G135500 Potri.014G180300 Potri.T161200 Potri.014G029100 Potri.006G096900 Potri.014G135500 Potri.008G025500 Potri.004G150300 Potri.016G136100 Potri.017G142700 Potri.014G134200 Potri.015G081000 Potri.002G107800 Potri.002G018000 Potri.005G161900 Potri.011G162000 Potri.004G175800 Potri.010G255500 Potri.006G069600 Potri.005G178300 Potri.007G023300 Potri.004G156400 Potri.012G143600 Potri.011G114300 Potri.001G111500 Potri.014G043100 Potri.004G010100 Potri.005G247700 Potri.006G137500 Potri.010G243700 Potri.005G112900 Potri.016G025000 Potri.011G120700 Potri.010G189800 Potri.005G138400 Potri.008G161600 Potri.001G200800 Potri.001G252900 Potri.014G115300 Potri.018G033400 Potri.003G219000 Potri.008G157400 Potri.018G109700 Potri.013G148900 Potri.004G018800 Potri.004G183100 Potri.008G158300 Potri.014G140500 Potri.003G093700 Potri.004G156500 Potri.018G051300 Potri.011G079600 Potri.013G079500 Potri.015G135400 Potri.007G122100 Potri.001G463800 Potri.002G214800 Potri.005G254100 Potri.005G124000 Potri.014G111600 Potri.007G007600 Potri.001G248700 Potri.009G081300 Potri.002G220400 Potri.006G229300 Potri.002G034400 Potri.004G028900 Potri.001G015400 Potri.007G026500 Potri.019G054800 Potri.016G112000 Potri.006G027300 Potri.015G074600 Potri.003G039000 Potri.006G248000 Potri.012G128200 Potri.007G038300 Potri.001G176100 Potri.013G125300 Potri.013G027000 Potri.014G133700 Potri.011G120200 Potri.006G199100 Potri.001G320000
GO:0030599	pectinesterase activity	13	Potri.006G134500 Potri.003G122000 Potri.003G086600 Potri.011G135000 Potri.003G076900 Potri.015G013700 Potri.007G107300 Potri.015G128400 Potri.010G109400 Potri.008G132600 Potri.006G137800 Potri.014G117100 Potri.012G014500
GO:0004601	peroxidase activity	7	Potri.017G037900 Potri.006G069600 Potri.004G134800 Potri.005G100400 Potri.002G018000 Potri.005G161900 Potri.007G122100
GO:0042578	phosphoric ester hydrolase activity	7	Potri.015G105000 Potri.003G131200 Potri.008G063800 Potri.001G191000 Potri.002G069000 Potri.006G094400 Potri.002G257700
GO:0004672	protein kinase activity	88	Potri.009G139400 Potri.001G467300 Potri.007G009200 Potri.001G246400 Potri.011G139800 Potri.018G096100 Potri.006G051700 Potri.002G070900 Potri.012G128700 Potri.017G108000 Potri.006G130000 Potri.013G156000 Potri.010G183400 Potri.008G204400 Potri.016G142800 Potri.004G212600 Potri.006G117200 Potri.005G139500 Potri.003G107600 Potri.001G066700 Potri.003G183100 Potri.006G235000 Potri.002G137700 Potri.003G205400 Potri.006G220100 Potri.005G086500 Potri.001G386000 Potri.010G120100 Potri.003G175700 Potri.T058000 Potri.017G130600

			Potri.016G144100 Potri.011G033400 Potri.007G046900 Potri.002G242700 Potri.006G114400 Potri.004G108200 Potri.013G048800 Potri.014G038300 Potri.006G260100 Potri.005G036600 Potri.017G134900 Potri.016G051600 Potri.005G241500 Potri.005G067000 Potri.006G104300 Potri.018G091200 Potri.015G141200 Potri.002G003400 Potri.005G257500 Potri.006G166600 Potri.011G067500 Potri.016G126300 Potri.011G045600 Potri.002G233600 Potri.001G465800 Potri.011G093700 Potri.008G130000 Potri.016G114400 Potri.010G058200 Potri.001G300700 Potri.017G115900 Potri.005G188700 Potri.016G055400 Potri.003G136100 Potri.004G001900 Potri.001G018700 Potri.013G039000 Potri.004G005300 Potri.012G127800 Potri.016G134600 Potri.015G093100 Potri.002G009300 Potri.006G228400 Potri.018G022300 Potri.011G164800 Potri.004G084000 Potri.012G095600 Potri.012G071100 Potri.001G384700 Potri.004G209700 Potri.001G126100 Potri.005G252000 Potri.001G095200 Potri.008G116800 Potri.016G070500 Potri.001G333300 Potri.013G025700
GO:0017171	serine hydrolase activity	28	Potri.003G164000 Potri.011G155400 Potri.014G018900 Potri.001G450600 Potri.002G120400 Potri.015G104700 Potri.009G031900 Potri.018G143400 Potri.001G065900 Potri.003G071800 Potri.012G105500 Potri.018G105700 Potri.010G220200 Potri.015G142000 Potri.006G141200 Potri.004G173900 Potri.001G440300 Potri.001G312800 Potri.018G111000 Potri.006G076200 Potri.001G290800 Potri.013G120200 Potri.001G291800 Potri.012G131500 Potri.006G183200 Potri.005G091700 Potri.011G076700 Potri.014G074600
GO:0016229	steroid dehydrogenase activity	11	Potri.003G093700 Potri.008G204900 Potri.001G320000 Potri.011G079600 Potri.004G183100 Potri.002G034400 Potri.001G237200 Potri.004G150300 Potri.012G128200 Potri.010G125400 Potri.013G079500
GO:0003700	transcription factor activity	38	Potri.014G099900 Potri.010G197300 Potri.006G167700 Potri.005G020500 Potri.018G053600 Potri.005G155700 Potri.002G030900 Potri.018G028000 Potri.001G155700 Potri.001G372300 Potri.019G043300 Potri.010G093400 Potri.013G059600 Potri.006G069400 Potri.007G007400 Potri.019G091900 Potri.014G045100 Potri.001G079600 Potri.001G155100 Potri.007G115100 Potri.001G374200 Potri.008G038900 Potri.001G079900 Potri.015G034100 Potri.019G033000 Potri.019G131300 Potri.013G075400 Potri.005G155300 Potri.002G114800 Potri.001G229700 Potri.012G047700 Potri.014G025300 Potri.005G055300 Potri.010G247200 Potri.008G061000 Potri.001G048200 Potri.018G091600 Potri.012G031100
GO:0016757	transferase activity, transferring glycosyl groups	37	Potri.002G132900 Potri.010G129400 Potri.002G257900 Potri.013G152400 Potri.016G020900 Potri.004G059600 Potri.002G044200 Potri.007G140600 Potri.008G018600 Potri.011G069600 Potri.010G242900 Potri.002G200300 Potri.014G125100 Potri.008G189400 Potri.005G061600 Potri.002G200200 Potri.006G179700 Potri.004G021000 Potri.007G008500 Potri.001G100700 Potri.016G086400 Potri.014G146100 Potri.003G159700 Potri.014G029900 Potri.010G042000

			Potri.010G160200 Potri.004G013900 Potri.013G066200 Potri.006G181900 Potri.001G012200 Potri.007G005900 Potri.001G416800 Potri.001G071000 Potri.018G103900 Potri.008G006500 Potri.011G047700 Potri.006G071200
GO:0016772	transferase activity, transferring phosphorus-containing groups	104	Potri.009G139400 Potri.009G073800 Potri.001G467300 Potri.007G009200 Potri.001G246400 Potri.003G088700 Potri.011G139800 Potri.018G096100 Potri.006G051700 Potri.002G070900 Potri.012G128700 Potri.017G108000 Potri.006G130000 Potri.013G156000 Potri.003G196000 Potri.010G183400 Potri.008G204400 Potri.016G142800 Potri.004G212600 Potri.006G164400 Potri.004G061700 Potri.006G117200 Potri.005G139500 Potri.003G107600 Potri.001G066700 Potri.003G183100 Potri.006G235000 Potri.002G137700 Potri.003G205400 Potri.006G220100 Potri.005G086500 Potri.016G078600 Potri.001G386000 Potri.010G120100 Potri.012G091700 Potri.003G175700 Potri.T058000 Potri.017G130600 Potri.010G027800 Potri.016G144100 Potri.011G033400 Potri.007G046900 Potri.002G242700 Potri.006G114400 Potri.001G027900 Potri.004G108200 Potri.013G048800 Potri.014G038300 Potri.006G260100 Potri.005G036600 Potri.017G134900 Potri.016G051600 Potri.005G241500 Potri.006G235100 Potri.013G039000 Potri.006G104300 Potri.018G091200 Potri.015G141200 Potri.002G003400 Potri.005G257500 Potri.012G095600 Potri.005G067000 Potri.016G126300 Potri.011G045600 Potri.002G233600 Potri.001G465800 Potri.011G093700 Potri.001G090300 Potri.008G130000 Potri.016G114400 Potri.010G058200 Potri.001G300700 Potri.017G115900 Potri.005G188700 Potri.016G055400 Potri.003G136100 Potri.004G001900 Potri.001G018700 Potri.005G237300 Potri.004G005300 Potri.012G127800 Potri.016G134600 Potri.001G134000 Potri.015G093100 Potri.002G009300 Potri.013G025700 Potri.018G022300 Potri.011G164800 Potri.004G084000 Potri.006G166600 Potri.018G113400 Potri.008G084500 Potri.012G071100 Potri.001G384700 Potri.004G209700 Potri.001G126100 Potri.011G067500 Potri.005G252000 Potri.005G238600 Potri.001G095200 Potri.008G116800 Potri.016G070500 Potri.001G333300 Potri.006G228400
GO:0005215	transporter activity	62	Potri.005G255400 Potri.002G092400 Potri.014G116000 Potri.008G186600 Potri.016G113300 Potri.018G139800 Potri.003G111500 Potri.006G255600 Potri.003G050900 Potri.016G103500 Potri.003G128600 Potri.009G136600 Potri.001G139600 Potri.001G068600 Potri.003G215600 Potri.002G019600 Potri.005G102800 Potri.013G117000 Potri.018G026500 Potri.013G108200 Potri.004G216500 Potri.005G037300 Potri.008G000300 Potri.017G081100 Potri.005G037000 Potri.017G116400 Potri.009G128200 Potri.008G147300 Potri.011G002400 Potri.019G055800 Potri.T171800 Potri.002G225500 Potri.006G128000 Potri.015G072600 Potri.012G081800 Potri.001G354900 Potri.002G199600 Potri.016G098200 Potri.009G061100 Potri.012G077400 Potri.009G151400 Potri.005G040000 Potri.010G003000 Potri.009G010700 Potri.001G455000 Potri.008G045100 Potri.008G017100 Potri.003G018800 Potri.014G157500 Potri.003G094400 Potri.002G211100

			Potri.001G160400 Potri.008G173800 Potri.010G112900 Potri.012G070300 Potri.006G054700 Potri.006G121700 Potri.012G144000 Potri.001G331700 Potri.012G070700 Potri.010G046300 Potri.010G111700

Supplementary Table S4: Details of Mapman analysis for Aig in Figure 5a. Expression pattern of each gene was presant by contrasting 6hpi_vs_CK, 36hpi_vs_CK, and 96hpi_vs_CK. The measured value of gene expression of genes was log2(fold change). The scale on the top right corner representes the value of log2(fold change).

Gene ID	Classfications in Mapman	*Arabidopsis thaliana*-symbol	*Arabidopsis thaliana*-defline	6 hpi	36 hpi	96 hpi
Potri.009 G153600	ABA	AAO2,AO3,AOgamma,AtAO-2,AtAO3	aldehyde oxidase 2	-2.33047	-0.11954	-0.3094
Potri.005 G138400	ABA	ABA1,ATABA1,ATZEP,IBS3,LOS6,NPQ2,ZEP	zeaxanthin epoxidase (ZEP) (ABA1)	3.572164	3.417359	1.691595
Potri.011 G112400	ABA	ATNCED3,NCED3,SIS7,STO1	nine-cis-epoxycarotenoid dioxygenase 3	-0.60753	-2.75396	-3.31947
Potri.006 G206500	ABA	ABA2,ATABA2,ATSDR1,GIN1,ISI4,SDR1,SIS4,SRE1	NAD(P)-binding Rossmann-fold superfamily protein	5.950037	3.237734	-11.2469
Potri.004 G235400	ABA	CYP707A1	cytochrome P450, family 707, subfamily A, polypeptide 1	2.512228	1.818214	-0.22021
Potri.006 G164600	ABA	ABI1,AtABI1	Protein phosphatase 2C family protein	-2.21112	-0.79862	-0.31787
Potri.006 G025800	ABA	AREB3,DPBF3	ABA-responsive element binding protein 3	2.761512	1.473832	2.220854
Potri.003 G165500	ABA	0	GRAM domain family protein	-2.65294	-2.59992	-0.65068
Potri.006 G069500	Abiotic stress	ATSPX2,SPX2	SPX domain gene 2	0.650292	4.262209	4.643887
Potri.002	Abiotic stress	ATSPX3,SPX3	SPX domain gene 3	12.21263	21.15102	19.66213

G143900						
Potri.008 G106400	Abiotic stress	ALPHA-DOX1,DIOX1,DOX1,PADOX-1	Peroxidase superfamily protein	-1.85387	2.178179	2.840375
Potri.003 G060000	Abiotic stress	UVR3	DNA photolyase family protein	1.651965	3.359219	3.711916
Potri.001 G420800	Abiotic stress	CHL	chloroplastic lipocalin	1.524473	2.216234	3.097325
Potri.001 G102400	Abiotic stress	ATOSM34,OSM34	osmotin 34	1.492433	4.291377	4.510996
Potri.005 G082800	Abiotic stress	GGT1	gamma-glutamyl transpeptidase 1	2.141456	2.584345	2.454168
Potri.001 G358200	Abiotic stress	ATBAG3,BAG3	BCL-2-associated athanogene 3	7.17559	6.672957	5.017564
Potri.001 G110300	Abiotic stress	ATBAG1,BAG1	BCL-2-associated athanogene 1	7.036069	3.6633	4.609712
Potri.009 G039200	Abiotic stress	0	HSP20-like chaperones superfamily protein	-3.55676	-1.06278	-0.58075
Potri.011 G139100	Abiotic stress	0	heat shock protein 70 (Hsp 70) family protein	1.581925	1.829191	2.072082
Potri.010 G195700	Abiotic stress	0	HSP20-like chaperones superfamily protein	-0.61065	-0.09874	3.227694
Potri.003 G071100	Abiotic stress	0	HSP20-like chaperones superfamily protein	-2.01537	-1.51882	-0.70515
Potri.010 G113400	Abiotic stress	0	Chaperone DnaJ-domain superfamily protein	-4.46354	-2.4591	-2.92149
Potri.015 G056900	Abiotic stress	ATHSP101,HOT1,HSP101	heat shock protein 101	-3.12467	-0.63736	-0.80809
Potri.001 G043100	Abiotic stress	J8	Chaperone DnaJ-domain superfamily protein	-0.41136	1.658585	2.112566
Potri.005 G113100	Abiotic stress	0	Chaperone DnaJ-domain superfamily protein	-2.89688	0.526247	2.664295
Potri.006 G226800	Abiotic stress	ATHSFA2,HSFA2	heat shock transcription factor A2	-2.23898	-0.77117	0.154133

Potri.002 G103900	Abiotic stress	0	Chaperone DnaJ-domain superfamily protein	1.493331	2.440881	2.816627
Potri.008 G069100	Abiotic stress	0	Double Clp-N motif-containing P-loop nucleoside triphosphate hydrolases superfamily protein	-0.3017	-2.76381	-2.1245
Potri.016 G043900	Abiotic stress	0	Heat shock protein DnaJ with tetratricopeptide repeat	1.383895	1.843388	2.08602
Potri.010 G205700	Abiotic stress	ATHSP70,HSP70	heat shock protein 70	-1.95788	-1.86846	-2.13613
Potri.006 G001300	Abiotic stress	0	Chaperone DnaJ-domain superfamily protein	6.117644	2.28981	1.086368
Potri.002 G198000	Abiotic stress	ATERDJ3B,ERDJ3B	DNAJ heat shock family protein	1.974963	1.721597	1.40652
Potri.001 G108100	Abiotic stress	AT-HSFB2B,HSFB2B	winged-helix DNA-binding transcription factor family protein	-2.80509	-1.66684	-1.41065
Potri.001 G319100	Abiotic stress	J20	DNAJ-like 20	1.128689	-0.35958	-2.2733
Potri.011 G031600	Abiotic stress	HSA32	Aldolase-type TIM barrel family protein	-2.01045	-1.63948	-0.90083
Potri.003 G033400	Abiotic stress	AtHsp90-7,AtHsp90.7,HSP90.7,SHD	Chaperone protein htpG family protein	0.950371	1.010968	1.97489
Potri.003 G109200	Abiotic stress	ATHSP23.6-MITO,HSP23.6-MITO	mitochondrion-localized small heat shock protein 23.6	-4.31891	-2.83638	-2.03161
Potri.012 G022400	Abiotic stress	HSP21	heat shock protein 21	2.573144	1.73386	4.162586
Potri.009 G119800	Abiotic stress	0	DNAJ heat shock N-terminal domain-containing protein	1.363541	1.986242	2.92905
Potri.008 G054600	Abiotic stress	AT-HSC70-1,HSC70,HSC70-1,HSP70-1	heat shock cognate protein 70-1	-1.34222	-1.81097	-2.07152
Potri.011 G051600	Abiotic stress	ATHSF3,ATHSFA1B,HSF3,HSFA1B	heat shock factor 3	1.283716	2.580563	3.251329
Potri.006 G221900	Abiotic stress	0	Chaperone DnaJ-domain superfamily protein	0.004846	1.103394	2.205788
Potri.009	Abiotic stress	ATJ2,J2	DNAJ homologue 2	-2.30291	-1.01559	-0.69905

G015700						
Potri.012 G098800	Abiotic stress	0	DNAJ heat shock N-terminal domain-containing protein	2.639789	1.754464	1.606671
Potri.001 G286700	Abiotic stress	AtHsp90.4,Hsp81.4	HEAT SHOCK PROTEIN 81.4	-2.4005	-2.22054	-2.46262
Potri.007 G122100	Abiotic stress	RCI3,RCI3A	Peroxidase superfamily protein	6.972604	10.39713	9.964783
Potri.001 G070400	Abiotic stress	0	Calcium-dependent lipid-binding (CaLB domain) family protein	0.726692	-1.5168	-2.75383
Potri.001 G454300	Abiotic stress	0	S-adenosyl-L-methionine-dependent methyltransferases superfamily protein	0.679446	2.70175	2.207265
Potri.002 G098000	Abiotic stress	OSU1,QUA2,TSD2	S-adenosyl-L-methionine-dependent methyltransferases superfamily protein	2.744646	2.148541	1.379895
Potri.002 G075800	Abiotic stress	0	S-adenosyl-L-methionine-dependent methyltransferases superfamily protein	1.350309	2.724691	1.756236
Potri.005 G020900	Abiotic stress	0	Drought-responsive family protein	-2.09054	-1.09141	-1.01325
Potri.005 G002100	Abiotic stress	RCI2A	Low temperature and salt responsive protein family	2.7687	0.865315	1.233639
Potri.002 G226800	Abiotic stress	0	ERD (early-responsive to dehydration stress) family protein	2.528933	0.458422	-0.71852
Potri.002 G036700	Abiotic stress	0	S-adenosyl-L-methionine-dependent methyltransferases superfamily protein	5.441067	4.316992	4.076279
Potri.001 G380000	Abiotic stress	0	0	-3.59826	-1.94984	1.021139
Potri.003 G087600	Abiotic stress	0	S-adenosyl-L-methionine-dependent methyltransferases superfamily protein	2.071336	2.920244	2.918969
Potri.016 G040600	Abiotic stress	0	S-adenosyl-L-methionine-dependent methyltransferases superfamily protein	-1.56233	-2.38921	4.971232
Potri.004 G136000	Abiotic stress	ATRD22,RD22	BURP domain-containing protein	3.26301	2.188655	3.785178
Potri.007 G104000	Abiotic stress	0	S-adenosyl-L-methionine-dependent methyltransferases superfamily protein	2.594468	1.234839	0.486855

Potri.T156100	Abiotic stress	ATCLH1,ATHCOR1,CLH1,CORI1	chlorophyllase 1	-0.47591	-2.30741	-3.45845
Potri.008G075200	Abiotic stress	ATWI-12,SAG20,WI12	senescence associated gene 20	2.044505	1.222251	2.600944
Potri.T162200	Abiotic stress	0	Wound-responsive family protein	2.427031	1.864003	1.825064
Potri.013G147700	Abiotic stress	0	Wound-responsive family protein	-2.57386	-1.38457	-0.99624
Potri.002G013200	Abiotic stress	ERD10,LTI29,LTI45	Dehydrin family protein	-0.35059	-3.768	-4.43069
Potri.004G033000	Abiotic stress	MLP423	MLP-like protein 423	1.117798	5.94265	3.881824
Potri.008G213100	Abiotic stress	MLP423	MLP-like protein 423	-0.17225	0.696327	3.170231
Potri.008G121900	Abiotic stress	0	Adenine nucleotide alpha hydrolases-like superfamily protein	-2.4532	-0.45722	0.685224
Potri.010G111000	Abiotic stress	MLP28	MLP-like protein 28	-1.23695	0.844351	3.467744
Potri.013G141900	Abiotic stress	ATGER1,GER1,GLP1	germin-like protein 1	2.72045	3.454451	0.944661
Potri.009G117500	Abiotic stress	RD2	Adenine nucleotide alpha hydrolases-like superfamily protein	2.377631	0.365788	0.661392
Potri.006G230600	Abiotic stress	PYL2,RCAR14	PYR1-like 2	-2.48463	-5.3038	-1.83399
Potri.019G128200	Abiotic stress	CHAL,EPFL6	allergen-related	2.660235	4.542277	2.653391
Potri.008G073400	Abiotic stress	PYL6,RCAR9	PYR1-like 6	0.94412	3.598051	-0.36646
Potri.002G184900	Abiotic stress	GLP10	germin-like protein 10	4.029347	1.855034	0.657074
Potri.002G093100	Abiotic stress	SAH7	Pollen Ole e 1 allergen and extensin family protein	2.264219	2.825999	3.328067
Potri.004	Abiotic stress	0	Pollen Ole e 1 allergen and extensin family protein	-3.42005	3.968829	3.921064

G114300						
Potri.004 G051500	Abiotic stress	0	Polyketide cyclase/dehydrase and lipid transport superfamily protein	2.14316	-0.12216	2.593179
Potri.009 G140400	Abiotic stress	0	RmlC-like cupins superfamily protein	-11.6443	-11.6443	7.257491
Potri.007 G033200	Abiotic stress	0	CAP (Cysteine-rich secretory proteins, Antigen 5, and Pathogenesis-related 1 protein) superfamily protein	2.105648	1.883398	1.078964
Potri.019 G061700	Auxin	ATMES17,MES17	methyl esterase 17	2.040006	-0.75165	-0.63309
Potri.002 G082400	Auxin	ILL6	IAA-leucine resistant (ILR)-like gene 6	-0.57801	-2.91592	-2.32773
Potri.016 G035300	Auxin	ATPIN1,PIN1	Auxin efflux carrier family protein	5.411274	3.38174	-0.10154
Potri.005 G174000	Auxin	LAX3	like AUX1 3	2.049246	0.854041	0.426064
Potri.004 G033900	Auxin	TIR1	F-box/RNI-like superfamily protein	-2.0138	0.224252	0.200278
Potri.014 G146800	Auxin	AGR,AGR1,ATPIN2,EIR1,PIN2, WAV6	Auxin efflux carrier family protein	1.725741	1.670524	2.108416
Potri.001 G164800	Auxin	0	Dormancy/auxin associated family protein	-2.82362	-2.25816	-1.67054
Potri.008 G158300	Auxin	0	NAD(P)-linked oxidoreductase superfamily protein	3.418308	2.342827	1.82458
Potri.002 G234000	Auxin	0	NAD(P)-linked oxidoreductase superfamily protein	-1.181	-2.64668	-2.40613
Potri.014 G147700	Auxin	ATB2	NAD(P)-linked oxidoreductase superfamily protein	-1.34561	-2.0422	-1.62473
Potri.003 G071000	Auxin	0	SAUR-like auxin-responsive protein family	5.001365	1.894105	0.042495
Potri.001 G298300	Auxin	GH3.1	Auxin-responsive GH3 family protein	-0.0943	-2.99762	-0.6911
Potri.008 G037900	Auxin	0	SAUR-like auxin-responsive protein family	19.99955	16.58908	13.55365

Potri.005 G096400	Auxin	0	SAUR-like auxin-responsive protein family	-3.24238	0.247456	-0.18291
Potri.004 G113200	Auxin	0	O-fucosyltransferase family protein	-2.31927	-1.52672	-1.22738
Potri.014 G098700	Auxin	0	Cytochrome b561/ferric reductase transmembrane with DOMON related domain	4.051553	1.972665	0.795429
Potri.019 G103500	Auxin	DFL2,GH3-10	Auxin-responsive GH3 family protein	4.295118	2.367463	-0.33702
Potri.001 G403000	Auxin	0	Aluminium induced protein with YGL and LRDR motifs	-2.72646	-0.50109	0.633454
Potri.009 G126000	Auxin	0	SAUR-like auxin-responsive protein family	1.97657	-2.69797	-1.57105
Potri.010 G156600	Auxin	0	Auxin-responsive family protein	16.82119	14.40896	13.14421
Potri.012 G065700	Beta glucanase	PDCB3	plasmodesmata callose-binding protein 3	1.961224	1.274139	1.568703
Potri.005 G202400	Beta glucanase	0	Carbohydrate-binding X8 domain superfamily protein	1.495547	2.731699	3.201753
Potri.007 G111000	Beta glucanase	0	Carbohydrate-binding X8 domain superfamily protein	4.206294	1.647588	2.814636
Potri.011 G094400	Beta glucanase	0	O-Glycosyl hydrolases family 17 protein	2.560411	2.323084	1.973391
Potri.001 G449100	Beta glucanase	0	Glycosyl hydrolase superfamily protein	4.667177	4.160765	4.039324
Potri.017 G130200	Beta glucanase	0	O-Glycosyl hydrolases family 17 protein	1.841005	3.268142	2.633897
Potri.014 G158400	Beta glucanase	0	O-Glycosyl hydrolases family 17 protein	4.000875	2.177871	0.896081
Potri.016 G057400	Beta glucanase	BG1	beta-1,3-glucanase 1	2.124243	4.074629	3.644909
Potri.001 G255100	Beta glucanase	0	Glycosyl hydrolase superfamily protein	-2.09945	-1.47871	2.267304
Potri.007	Brassinost.	CLM,CYP90B1,DWF4,PSC1,SA	Cytochrome P450 superfamily protein	-2.01976	-1.27009	-1.66775

G026500		V1,SNP2				
Potri.010 G189800	Brassinost.	CBB3,CPD,CYP90,CYP90A,CYP90A1,DWF3	Cytochrome P450 superfamily protein	-0.48725	-2.16874	-1.09093
Potri.003 G192300	Brassinost.	BAS1,CYP72B1,CYP734A1	Cytochrome P450 superfamily protein	-2.05799	-1.19525	-1.26375
Potri.T05 2100	Brassinost.	SQE1,XF1	FAD/NAD(P)-binding oxidoreductase family protein	2.627055	1.068825	0.486975
Potri.006 G079300	Brassinost.	CAS1	cycloartenol synthase 1	2.70948	2.489939	2.212078
Potri.008 G140500	Brassinost.	BRL2,VH1	BRI1-like 2	2.954446	1.503905	0.471182
Potri.016 G126300	Brassinost.	IMK2	inflorescence meristem receptor-like kinase 2	4.075073	2.026699	1.8593
Potri.007 G030700	Brassinost.	BZR1	Brassinosteroid signalling positive regulator (BZR1) family protein	-1.02632	-1.65285	-2.02329
Potri.001 G386900	Brassinost.	BEH4	BES1/BZR1 homolog 4	-1.86903	-2.48417	-1.66951
Potri.004 G206600	Brassinost.	EXL5	EXORDIUM like 5	2.408429	0.716854	-1.22824
Potri.007 G121800	B-ZIP	0	TraB family protein	1.835079	2.294075	3.218285
Potri.004 G203400	B-ZIP	bZIP21,TGA9	bZIP transcription factor family protein	-1.07301	-2.26624	-1.99549
Potri.002 G082000	B-ZIP	0	Protein of unknown function (DUF630 and DUF632)	2.360508	2.284867	2.259253
Potri.010 G132700	B-ZIP	0	ATPase E1-E2 type family protein / haloacid dehalogenase-like hydrolase family protein	3.308271	1.916427	1.603613
Potri.002 G031900	B-ZIP	AtbZIP44,bZIP44	basic leucine-zipper 44	0.032356	2.776957	2.852716
Potri.014 G094200	B-ZIP	GBF3	G-box binding factor 3	-2.54739	-1.13428	-0.71361
Potri.010 G004200	B-ZIP	HYH	HY5-homolog	1.997633	2.438541	2.351034

Potri.008 G106700	B-ZIP	ATBZIP53,BZIP53	basic region/leucine zipper motif 53	0.634544	3.006328	3.606953
Potri.004 G163800	B-ZIP	0	Basic-leucine zipper (bZIP) transcription factor family protein	1.203632	2.19541	2.614815
Potri.006 G058800	B-ZIP	bZIP65,TGA10	bZIP transcription factor family protein	-6.56703	-3.32007	1.517121
Potri.010 G142900	B-ZIP	AtbZIP1,bZIP1	basic leucine-zipper 1	-1.6478	1.40639	1.931565
Potri.008 G060100	Cell wall	CYT1,EMB101,GMP1,SOZ1,VTC1	Glucose-1-phosphate adenylyltransferase family protein	3.198397	2.084281	1.877745
Potri.004 G189900	Cell wall	AXS2	UDP-D-apiose/UDP-D-xylose synthase 2	2.057635	0.556269	-0.15731
Potri.008 G094300	Cell wall	0	UDP-glucose 6-dehydrogenase family protein	3.630643	0.622862	0.378121
Potri.004 G118600	Cell wall	0	UDP-glucose 6-dehydrogenase family protein	2.510025	-0.01703	-0.65407
Potri.001 G237200	Cell wall	UXS5	UDP-XYL synthase 5	5.351751	3.006498	1.637991
Potri.012 G128200	Cell wall	GAE2	UDP-D-glucuronate 4-epimerase 2	2.136249	2.996847	3.125992
Potri.001 G320000	Cell wall	GAE6	UDP-D-glucuronate 4-epimerase 6	2.361398	2.450405	1.025669
Potri.005 G116200	Cell wall	GMD2,MUR1,MUR_1	NAD(P)-binding Rossmann-fold superfamily protein	4.102415	0.895172	-0.59032
Potri.011 G156100	Cell wall	HSR8,MUR4,UXE1	NAD(P)-binding Rossmann-fold superfamily protein	2.379115	0.005326	-0.83231
Potri.002 G061900	Cell wall	AtkdsA1	Aldolase-type TIM barrel family protein	2.665692	2.597737	2.001595
Potri.011 G120100	Cell wall	KDSB	Nucleotide-diphospho-sugar transferases superfamily protein	2.15197	1.457873	1.208143
Potri.014 G080300	Cell wall	ATPMM,PMM	phosphomannomutase	1.499697	1.939999	2.159442
Potri.006	Cell wall	GalAK	galacturonic acid kinase	2.331581	1.80555	2.270163

G070500						
Potri.018 G003100	Cell wall	ARA1,ATISA1,ISA1	arabinose kinase	-2.27073	-1.67466	-1.19879
Potri.018 G009300	Cell wall	ATCSLC05,ATCSLC5,CSLC05, CSLC5	Cellulose-synthase-like C5	4.152703	4.432318	3.223786
Potri.008 G026400	Cell wall	ATCSLA09,ATCSLA9,CSLA09, CSLA9,RAT4	Nucleotide-diphospho-sugar transferases superfamily protein	4.53449	2.700572	1.041488
Potri.014 G125100	Cell wall	ATCSLD5,CSLD5,SOS6	cellulose synthase-like D5	2.379607	-0.28223	-0.16407
Potri.002 G066600	Cell wall	CESA09,CESA9	cellulose synthase A9	2.20388	4.290589	3.502729
Potri.013 G082200	Cell wall	ATCSLD3,CSLD3,KJK	cellulose synthase-like D3	-1.57082	-1.84543	-1.96825
Potri.004 G059600	Cell wall	ATCESA8,CESA8,IRX1,LEW2	cellulose synthase family protein	5.26153	3.555135	2.3122
Potri.018 G029400	Cell wall	AtCESA1,CESA1,RSW1	cellulose synthase 1	2.670279	0.713996	-0.34591
Potri.006 G052600	Cell wall	ATCESA3,ATH-B,CESA3,CEV1 ,IXR1	Cellulose synthase family protein	2.060868	1.403505	1.255111
Potri.006 G181900	Cell wall	ATCESA7,CESA7,IRX3,MUR10	Cellulose synthase family protein	3.961914	2.089802	1.011964
Potri.002 G257900	Cell wall	CESA4,IRX5,NWS2	cellulose synthase A4	4.750442	2.504407	1.132009
Potri.010 G001100	Cell wall	ATSEB1,COBL7,SEB1	COBRA-like protein-7 precursor	2.273387	1.944847	1.086027
Potri.015 G060100	Cell wall	COBL4,IRX6	COBRA-like extracellular glycosyl-phosphatidyl inositol-anchored protein family	4.072198	2.626395	1.819705
Potri.015 G060000	Cell wall	COB	COBRA-like extracellular glycosyl-phosphatidyl inositol-anchored protein family	3.463903	2.166024	1.568487
Potri.003 G191200	Cell wall	ATFT1,ATFUT1,FT1,MUR2	fucosyltransferase 1	3.059478	0.862087	0.813489
Potri.013 G067400	Cell wall	0	exostosin family protein	6.023497	6.533781	6.955204

Potri.006 G064800	Cell wall	0	Exostosin family protein	-0.75707	1.901874	2.273423
Potri.010 G040400	Cell wall	0	Galactosyltransferase family protein	0.789764	1.78308	2.085896
Potri.002 G132900	Cell wall	ATGATL1,GATL1,GLZ1,PARVUS	Nucleotide-diphospho-sugar transferases superfamily protein	2.921299	0.351594	0.811281
Potri.006 G131000	Cell wall	IRX9	Nucleotide-diphospho-sugar transferases superfamily protein	5.351396	2.778879	2.15416
Potri.014 G029900	Cell wall	GUX2,PGSIP3	plant glycogenin-like starch initiation protein 3	4.66806	2.824077	2.092323
Potri.001 G416800	Cell wall	GAUT12,IRX8,LGT6	galacturonosyltransferase 12	6.136344	4.047791	3.309247
Potri.002 G151400	Cell wall	GAUT1,LGT1	galacturonosyltransferase 1	2.108262	1.55617	1.263965
Potri.014 G162900	Cell wall	FLA7	FASCICLIN-like arabinoogalactan 7	6.812854	2.377899	1.578591
Potri.014 G135100	Cell wall	AGP26,ATAGP26	arabinogalactan protein 26	2.136368	4.215615	4.143495
Potri.014 G071700	Cell wall	FLA10	FASCICLIN-like arabinogalactan-protein 10	3.261724	1.966279	1.962775
Potri.014 G168100	Cell wall	FLA2	FASCICLIN-like arabinogalactan 2	7.672972	6.878345	4.585993
Potri.005 G144900	Cell wall	AGP18,ATAGP18	arabinogalactan protein 18	5.362945	4.333375	1.877456
Potri.006 G129200	Cell wall	ATFLA11,FLA11	FASCICLIN-like arabinogalactan-protein 11	4.941675	2.87161	1.62195
Potri.008 G012400	Cell wall	FLA17	FASCICLIN-like arabinogalactan protein 17 precursor	4.387004	2.864914	2.293263
Potri.001 G367900	Cell wall	FLA1	FASCICLIN-like arabinogalactan 1	3.955075	2.971059	2.271718
Potri.001 G004100	Cell wall	AGP14,ATAGP14	arabinogalactan protein 14	4.617713	3.600724	2.786495
Potri.001	Cell wall	AGP1,ATAGP1	arabinogalactan protein 1	2.189422	1.315445	0.014456

G310400						
Potri.006 G081200	Cell wall	LRX2	leucine-rich repeat/extensin 2	2.542762	1.956222	0.443151
Potri.002 G175400	Cell wall	0	0	5.614931	3.958279	2.9429
Potri.009 G098700	Cell wall	0	Leucine-rich repeat (LRR) family protein	2.722223	1.216655	0.323337
Potri.018 G075900	Cell wall	0	Leucine-rich repeat (LRR) family protein	2.822635	1.070033	-0.0007
Potri.002 G070100	Cell wall	ATEXT3,EXT3,RSH	extensin 3	7.126324	8.393735	6.253574
Potri.005 G190100	Cell wall	ATEXT1,ATEXT4,EXT1,EXT4,ORG5	extensin 4	2.082735	2.944076	4.729337
Potri.008 G153000	Cell wall	0	FRIGIDA-like protein	-1.59181	-1.85772	-2.0012
Potri.004 G117800	Cell wall	ATRGP2,RGP2	reversibly glycosylated polypeptide 2	2.190091	-0.43128	-1.49326
Potri.006 G062200	Cell wall	AtGUS3,GUS3	glucuronidase 3	4.052193	4.158547	3.331916
Potri.015 G137500	Cell wall	0	peptidoglycan-binding LysM domain-containing protein	0.283883	1.470976	3.802826
Potri.002 G023900	Cell wall	AtGH9B5,GH9B5	glycosyl hydrolase 9B5	2.087622	0.830352	0.296022
Potri.014 G157600	Cell wall	AtGH9B8,GH9B8	glycosyl hydrolase 9B8	4.241257	0.486762	0.012503
Potri.009 G153900	Cell wall	0	Glycosyl hydrolase family protein	0.646942	1.88001	2.145435
Potri.014 G122200	Cell wall	ATBXL2,BXL2	beta-xylosidase 2	6.198691	4.010421	2.189488
Potri.002 G094000	Cell wall	0	Glycosyl hydrolase family protein	1.25653	2.050794	2.105996
Potri.008 G108100	Cell wall	ATBXL1,BXL1	beta-xylosidase 1	0.965445	-1.58308	4.086601

Potri.002 G110100	Cell wall	0	Rhamnogalacturonate lyase family protein	0.98175	-1.58333	4.695576
Potri.010 G152000	Cell wall	0	Pectin lyase-like superfamily protein	-4.61406	2.632671	6.302081
Potri.008 G190000	Cell wall	PG2	polygalacturonase 2	3.563668	5.63215	4.742901
Potri.001 G171900	Cell wall	0	Pectin lyase-like superfamily protein	2.243133	1.845288	2.527958
Potri.016 G054800	Cell wall	ADPG2,PGAZAT	polygalacturonase abscission zone A. thaliana	0.571026	2.421496	3.260896
Potri.008 G048600	Cell wall	0	Pectin lyase-like superfamily protein	15.35619	15.71439	18.29148
Potri.003 G131700	Cell wall	0	Pectin lyase-like superfamily protein	0.787328	1.391146	2.737004
Potri.001 G100000	Cell wall	0	Pectin lyase-like superfamily protein	-1.2416	2.056679	1.411333
Potri.006 G058600	Cell wall	ATPGIP1,PGIP1	polygalacturonase inhibiting protein 1	-0.5747	2.086168	2.995588
Potri.004 G021000	Cell wall	XTH8	xyloglucan endotransglucosylase/hydrolase 8	4.829001	1.777786	-0.09433
Potri.008 G088300	Cell wall	AT-EXP1,ATEXP1,ATEXPA1,ATHEXP ALPHA 1.2,EXP1,EXPA1	expansin A1	-0.88547	-2.40803	-0.54147
Potri.010 G167200	Cell wall	AT-EXP1,ATEXP1,ATEXPA1,ATHEXP ALPHA 1.2,EXP1,EXPA1	expansin A1	-0.52607	-4.26986	-2.48701
Potri.018 G041300	Cell wall	PNP-A	plant natriuretic peptide A	8.526742	5.443023	-0.42434
Potri.009 G006600	Cell wall	XTH32	xyloglucan endotransglucosylase/hydrolase 32	1.302386	1.272769	4.052646
Potri.016 G135200	Cell wall	ATEXP8,ATEXPA8,ATHEXP ALPHA 1.11,EXP8,EXPA8	expansin A8	-2.16855	-1.92896	-2.57873
Potri.002	Cell wall	XTH16	xyloglucan endotransglucosylase/hydrolase 16	3.339685	2.81394	0.55965

G236200						
Potri.019 G125000	Cell wall	XTH9	xyloglucan endotransglucosylase/hydrolase 9	7.847328	4.136917	1.499874
Potri.005 G201200	Cell wall	XTH15,XTR7	xyloglucan endotransglucosylase/hydrolase 15	3.858026	-0.31371	0.628046
Potri.003 G083200	Cell wall	AT-EXPR,ATEXLB1,ATEXPR1, ATHEXP BETA 3.1,EXLB1,EXPR	expansin-like B1	2.809063	1.587799	4.306359
Potri.005 G007200	Cell wall	XTH23,XTR6	xyloglucan endotransglycosylase 6	2.432217	-1.03822	-1.99197
Potri.006 G249500	Cell wall	0	Barwin-related endoglucanase	3.852411	4.319546	2.943214
Potri.004 G181700	Cell wall	ATEXLA2,ATEXPL2,ATHEXP BETA 2.2,EXLA2,EXPL2	expansin-like A2	2.191196	-1.93265	-3.06189
Potri.001 G071000	Cell wall	EXGT-A4,XTH5	xyloglucan endotransglucosylase/hydrolase 5	5.133429	1.67426	-0.7827
Potri.018 G094800	Cell wall	TCH4,XTH22	Xyloglucan endotransglucosylase/hydrolase family protein	-5.15112	-3.82394	-1.6774
Potri.002 G202600	Cell wall	0	Plant invertase/pectin methylesterase inhibitor superfamily	4.254912	4.798514	2.423095
Potri.011 G135000	Cell wall	ATPMEPCRA,PMEPCRA	methylesterase PCR A	2.20086	0.206029	0.4469
Potri.013 G013200	Cell wall	0	Plant invertase/pectin methylesterase inhibitor superfamily	0.939382	0.324213	2.035547
Potri.018 G051400	Cell wall	ATPME3,PME3	pectin methylesterase 3	3.163949	6.208189	6.44799
Potri.015 G013700	Cell wall	0	Plant invertase/pectin methylesterase inhibitor superfamily	2.238077	1.825116	0.755213
Potri.007 G107300	Cell wall	0	Plant invertase/pectin methylesterase inhibitor superfamily	0.139402	2.335827	3.284979
Potri.006 G135100	Cell wall	0	Plant invertase/pectin methylesterase inhibitor superfamily	1.780329	-2.24438	-2.56255
Potri.005	Cell wall	0	Pectinacetylesterase family protein	2.706553	2.362292	3.369047

G001500						
Potri.014 G110900	Cell wall	0	Pectinacetylesterase family protein	2.115257	2.156766	1.841368
Potri.003 G046200	Cell wall	0	Pectinacetylesterase family protein	4.927142	4.940866	3.797269
Potri.012 G142300	Cell wall	0	Pectinacetylesterase family protein	17.81362	16.48879	15.31907
Potri.010 G004400	Cell wall	0	Pectinacetylesterase family protein	16.32316	15.27625	14.81566
Potri.004 G233900	Cell wall	0	Pectinacetylesterase family protein	3.61088	3.437476	3.409214
Potri.010 G109400	Cell wall	0	Plant invertase/pectin methylesterase inhibitor superfamily	-3.72242	-4.77507	-4.31513
Potri.002 G145500	Cell wall	0	Plant invertase/pectin methylesterase inhibitor superfamily	3.415437	4.024837	3.140437
Potri.014 G149700	Cell wall	PME61	pectin methylesterase 61	2.848446	1.046112	-0.02531
Potri.002 G202500	Cell wall	0	Plant invertase/pectin methylesterase inhibitor superfamily	5.059821	6.548655	3.823302
Potri.011 G065900	DOF	0	Dof-type zinc finger DNA-binding family protein	-0.09281	-0.50295	2.684593
Potri.014 G036600	DOF	0	Dof-type zinc finger DNA-binding family protein	2.838224	1.541537	0.960723
Potri.005 G131600	DOF	OBP1	OBF binding protein 1	0.538616	2.601403	3.863159
Potri.004 G046100	DOF	OBP4	OBF binding protein 4	-1.40995	-1.45388	-2.07439
Potri.019 G073300	ERF	0	Integrase-type DNA-binding superfamily protein	3.059104	-4.48589	-6.112
Potri.001 G092400	ERF	0	Integrase-type DNA-binding superfamily protein	-0.22201	-2.21065	-4.6198
Potri.002 G065600	ERF	0	Integrase-type DNA-binding superfamily protein	2.824649	1.143026	-0.39078

Potri.016 G126100	ERF	0	Integrase-type DNA-binding superfamily protein	-3.36504	-2.49064	-1.96563
Potri.002 G201600	ERF	HRE2	Integrase-type DNA-binding superfamily protein	4.620306	0.272384	-1.64597
Potri.002 G167400	ERF	CRF4	cytokinin response factor 4	-0.82422	-2.14493	-2.46735
Potri.018 G043900	ERF	TINY2	Integrase-type DNA-binding superfamily protein	3.024675	3.771361	5.544769
Potri.012 G134100	ERF	CBF4,DREB1D	C-repeat-binding factor 4	-1.41971	-5.31593	-5.20213
Potri.012 G134000	ERF	0	Integrase-type DNA-binding superfamily protein	2.61278	-2.11544	-2.80568
Potri.012 G108500	ERF	0	Integrase-type DNA-binding superfamily protein	2.242882	4.891389	6.405141
Potri.005 G087200	ERF	ABR1	Integrase-type DNA-binding superfamily protein	2.120409	-2.41535	-2.88509
Potri.009 G099200	Ethylene	CDCP1,LEJ1	Cystathionine beta-synthase (CBS) family protein	2.389833	0.96545	0.73589
Potri.006 G062500	Ethylene	0	2-oxoglutarate (2OG) and Fe(II)-dependent oxygenase superfamily protein	2.583367	4.450378	6.071233
Potri.002 G113900	Ethylene	ACS8	1-amino-cyclopropane-1-carboxylate synthase 8	-12.7912	1.262986	4.816055
Potri.011 G020900	Ethylene	ACO4,EAT1,EFE	ethylene-forming enzyme	0.622864	4.038904	3.920705
Potri.006 G151600	Ethylene	ACO1,ATACO1	ACC oxidase 1	5.978599	3.212676	1.195559
Potri.004 G047500	Ethylene	ATERF12,ERF12	ERF domain protein 12	-0.03744	-3.94131	-4.28505
Potri.001 G397200	Ethylene	ATERF-4,ATERF4,ERF4,RAP2.5	ethylene responsive element binding factor 4	-0.34717	-2.06417	-2.35874
Potri.010 G074300	Ethylene	ETR2	Signal transduction histidine kinase, hybrid-type, ethylene sensor	2.185329	-0.07589	-0.16933
Potri.005	Ethylene	0	Integrase-type DNA-binding superfamily protein	4.071186	1.972355	0.162776

G223100						
Potri.003 G080600	Ethylene	ATERF6,ERF-6-6,ERF6	ethylene responsive element binding factor 6	3.321246	0.076258	-1.25752
Potri.004 G051700	Ethylene	ATERF-1,ERF-1	ethylene responsive element binding factor 1	2.679008	-0.39149	-1.17734
Potri.004 G051800	Ethylene	0	Integrase-type DNA-binding superfamily protein	2.891307	1.042621	0.676351
Potri.T01 2600	Ethylene	0	0	4.261617	3.196281	2.866281
Potri.004 G047600	Ethylene	ATERF-9,ATERF9,ERF9	erf domain protein 9	1.751297	0.292711	2.106857
Potri.018 G075200	Ethylene	ATMBF1C,MBF1C	multiprotein bridging factor 1C	-3.16123	-2.93491	-1.32828
Potri.014 G122000	Ethylene	0	Adenine nucleotide alpha hydrolases-like superfamily protein	-1.1658	-2.27858	-2.72152
Potri.T03 5100	Glutathione-S-transferase	ATGST1,ATGSTF3,ATGSTF6,ERD11,GST1,GSTF6	glutathione S-transferase 6	-2.01246	-1.27927	-1.02184
Potri.001 G458700	Glutathione-S-transferase	0	Peroxidase superfamily protein	18.811	17.73573	15.88066
Potri.T16 0100	Glutathione-S-transferase	0	Peroxidase superfamily protein	4.383442	3.421207	1.445481
Potri.001 G431600	Glutathione-S-transferase	ATGSTU25,GSTU25	glutathione S-transferase TAU 25	-4.70015	-2.16562	2.668936
Potri.004 G078100	Glutathione-S-transferase	0	microsomal glutathione s-transferase, putative	-2.18586	-0.58388	-0.59503
Potri.001 G437300	Glutathione-S-transferase	ATGSTU22,GSTU22	glutathione S-transferase TAU 22	-3.85124	-0.83485	0.297453
Potri.001 G431400	Glutathione-S-transferase	ATGSTU19,GST8,GSTU19	glutathione S-transferase TAU 19	-6.77155	-0.59616	3.072936
Potri.014 G069600	Glutathione-S-transferase	ATGSTZ1,GST18,GSTZ1	glutathione S-transferase zeta 1	-2.07528	-1.52751	-1.14231
Potri.010 G060900	Glutathione-S-transferase	ATGSTU7,GST25,GSTU7	glutathione S-transferase tau 7	-1.97405	0.748159	2.404203

Potri.015 G042000	Glutathione-S-transferase	ATGSTU4,GST22,GSTU4	glutathione S-transferase tau 4	2.27945	0.397709	0.653232
Potri.002 G015100	Glutathione-S-transferase	ATGSTF11,ATGSTF6,GSTF11	glutathione S-transferase F11	2.53265	3.738672	1.65337
Potri.016 G104500	Glutathione-S-transferase	ATGSTU8,GSTU8	glutathione S-transferase TAU 8	-5.28862	-2.84933	-0.25804
Potri.016 G083500	Glutathione-S-transferase	GSTL3	Glutathione S-transferase family protein	2.688046	0.193246	0.73364
Potri.017 G138800	Glutathione-S-transferase	ATGSTF12,GST26,GSTF12,TT19	glutathione S-transferase phi 12	13.2281	24.05798	20.86276
Potri.009 G039200	Heat shock proteins	0	HSP20-like chaperones superfamily protein	-3.55676	-1.06278	-0.58075
Potri.011 G139100	Heat shock proteins	0	heat shock protein 70 (Hsp 70) family protein	1.581925	1.829191	2.072082
Potri.010 G195700	Heat shock proteins	0	HSP20-like chaperones superfamily protein	-0.61065	-0.09874	3.227694
Potri.003 G071100	Heat shock proteins	0	HSP20-like chaperones superfamily protein	-2.01537	-1.51882	-0.70515
Potri.010 G113400	Heat shock proteins	0	Chaperone DnaJ-domain superfamily protein	-4.46354	-2.4591	-2.92149
Potri.015 G056900	Heat shock proteins	ATHSP101,HOT1,HSP101	heat shock protein 101	-3.12467	-0.63736	-0.80809
Potri.001 G043100	Heat shock proteins	J8	Chaperone DnaJ-domain superfamily protein	-0.41136	1.658585	2.112566
Potri.005 G113100	Heat shock proteins	0	Chaperone DnaJ-domain superfamily protein	-2.89688	0.526247	2.664295
Potri.006 G226800	Heat shock proteins	ATHSFA2,HSFA2	heat shock transcription factor A2	-2.23898	-0.77117	0.154133
Potri.002 G103900	Heat shock proteins	0	Chaperone DnaJ-domain superfamily protein	1.493331	2.440881	2.816627
Potri.008 G069100	Heat shock proteins	0	Double Clp-N motif-containing P-loop nucleoside triphosphate hydrolases superfamily protein	-0.3017	-2.76381	-2.1245
Potri.016	Heat shock	0	Heat shock protein DnaJ with tetratricopeptide repeat	1.383895	1.843388	2.08602

G043900	proteins					
Potri.010 G205700	Heat shock proteins	ATHSP70,HSP70	heat shock protein 70	-1.95788	-1.86846	-2.13613
Potri.006 G001300	Heat shock proteins	0	Chaperone DnaJ-domain superfamily protein	6.117644	2.28981	1.086368
Potri.002 G198000	Heat shock proteins	ATERDJ3B,ERDJ3B	DNAJ heat shock family protein	1.974963	1.721597	1.40652
Potri.001 G108100	Heat shock proteins	AT-HSFB2B,HSFB2B	winged-helix DNA-binding transcription factor family protein	-2.80509	-1.66684	-1.41065
Potri.001 G319100	Heat shock proteins	J20	DNAJ-like 20	1.128689	-0.35958	-2.2733
Potri.011 G031600	Heat shock proteins	HSA32	Aldolase-type TIM barrel family protein	-2.01045	-1.63948	-0.90083
Potri.003 G033400	Heat shock proteins	AtHsp90-7,AtHsp90.7,HSP90.7,SHD	Chaperone protein htpG family protein	0.950371	1.010968	1.97489
Potri.003 G109200	Heat shock proteins	ATHSP23.6-MITO,HSP23.6-MITO	mitochondrion-localized small heat shock protein 23.6	-4.31891	-2.83638	-2.03161
Potri.012 G022400	Heat shock proteins	HSP21	heat shock protein 21	2.573144	1.73386	4.162586
Potri.009 G119800	Heat shock proteins	0	DNAJ heat shock N-terminal domain-containing protein	1.363541	1.986242	2.92905
Potri.008 G054600	Heat shock proteins	AT-HSC70-1,HSC70,HSC70-1,HSP70-1	heat shock cognate protein 70-1	-1.34222	-1.81097	-2.07152
Potri.011 G051600	Heat shock proteins	ATHSF3,ATHSFA1B,HSF3,HSFA1B	heat shock factor 3	1.283716	2.580563	3.251329
Potri.006 G221900	Heat shock proteins	0	Chaperone DnaJ-domain superfamily protein	0.004846	1.103394	2.205788
Potri.009 G015700	Heat shock proteins	ATJ2,J2	DNAJ homologue 2	-2.30291	-1.01559	-0.69905
Potri.012 G098800	Heat shock proteins	0	DNAJ heat shock N-terminal domain-containing protein	2.639789	1.754464	1.606671
Potri.001 G286700	Heat shock proteins	AtHsp90.4,Hsp81.4	HEAT SHOCK PROTEIN 81.4	-2.4005	-2.22054	-2.46262

Potri.009 G099200	JA	CDCP1,LEJ1	Cystathionine beta-synthase (CBS) family protein	2.389833	0.96545	0.73589
Potri.005 G032400	JA	ATLOX1,LOX1	lipoxygenase 1	-1.30368	-2.15148	-1.40328
Potri.008 G178000	JA	0	PLAT/LH2 domain-containing lipoxygenase family protein	-2.6193	0.012167	0.256348
Potri.001 G015300	JA	ATLOX2,LOX2	lipoxygenase 2	1.446234	3.849458	3.09802
Potri.004 G149000	JA	AOS,CYP74A,DDE2	allene oxide synthase	-0.15691	-3.55605	-3.28064
Potri.015 G035800	JA	JAI3,JAZ3,TIFY6B	jasmonate-zim-domain protein 3	-2.63677	-1.00438	-0.94089
Potri.012 G009200	MAPK	AtRLP33,RLP33	receptor like protein 33	-1.86658	-1.83818	-4.1252
Potri.014 G155000	MAPK	MAPKKK18	mitogen-activated protein kinase kinase kinase 18	3.04798	-1.97003	-1.67417
Potri.015 G030700	MAPK	ATMKK9,MKK9	MAP kinase kinase 9	-1.65873	-2.02609	-3.72388
Potri.005 G201800	MAPK	ATMPK20,MPK20	MAP kinase 20	1.305015	2.099663	2.558762
Potri.001 G085500	MAPK	ATWNK8,WNK8	with no lysine (K) kinase 8	0.220466	1.747309	2.211977
Potri.019 G128600	MAPK	WNK4,ZIK2	with no lysine (K) kinase 4	0.635872	1.623066	2.437614
Potri.005 G140100	MAPK	0	PLC-like phosphodiesterases superfamily protein	1.702752	2.408474	1.483632
Potri.005 G001600	MYB	ATMYB61,MYB61	myb domain protein 61	12.77767	14.8213	16.11124
Potri.002 G173900	MYB	ATMYB3,MYB3	myb domain protein 3	-2.11132	-0.01267	1.254629
Potri.008 G122100	MYB	AtMYB62,BW62B,BW62C,MYB62	myb domain protein 62	-3.58028	-4.59166	-1.59333
Potri.002	MYB	ATMYB14,MYB14,MYB14AT	myb domain protein 14	3.055498	1.521687	1.041109

G038500						
Potri.019 G032700	MYB	0	Homeodomain-like superfamily protein	2.597029	1.41954	1.333894
Potri.005 G224100	MYB	ATMYB15,ATY19,MYB15	myb domain protein 15	3.930767	-2.10834	-0.84768
Potri.004 G088100	MYB	AtMYB6,MYB6	myb domain protein 6	-2.33833	0.516479	-0.7191
Potri.003 G114100	MYB	AtMYB42,MYB42	myb domain protein 42	3.33641	2.868916	3.380617
Potri.018 G038000	MYB	ATMYB3R-1,ATMYB3R1,MYB 3R-1,MYB3R1,PC-MYB1	Homeodomain-like protein	-0.57467	-1.53971	-1.98757
Potri.002 G128900	MYB	ATMYB73,MYB73	myb domain protein 73	-0.71391	-2.00455	-1.53637
Potri.017 G130300	MYB	AtMYB43,MYB43	myb domain protein 43	0.971889	3.411449	2.503745
Potri.013 G067000	MYB	AtMYB56,MYB56	myb domain protein 56	4.445574	0.816174	0.865055
Potri.012 G084100	MYB	ATMYB86,MYB86	myb domain protein 86	2.420206	1.281119	0.81143
Potri.001 G169600	MYB	ATMYB23,ATMYBRTF,MYB23	myb domain protein 23	-0.29772	3.784366	3.711845
Potri.014 G037200	MYB	ATS,KAN4	Homeodomain-like superfamily protein	2.106868	1.934705	0.902872
Potri.001 G470500	MYB	AtMYB103,ATMYB80,MS188, MYB103	myb domain protein 103	2.180619	0.760089	1.001807
Potri.002 G188100	MYB	ATRL6,RL6,RSM3	RAD-like 6	3.689915	5.558696	4.521999
Potri.006 G097300	MYB	0	Duplicated homeodomain-like superfamily protein	2.547734	2.512291	1.181492
Potri.004 G015100	MYB	CPC	Homeodomain-like superfamily protein	-1.86855	0.634209	2.564403
Potri.006 G133000	MYB	0	Homeodomain-like superfamily protein	-2.29127	-1.54327	-1.64518

Potri.001 G219100	MYB	0	Duplicated homeodomain-like superfamily protein	2.736375	1.416867	2.628856
Potri.008 G064200	MYB	0	Duplicated homeodomain-like superfamily protein	2.619816	5.455427	3.001122
Potri.004 G074300	MYB	RVE1	Homeodomain-like superfamily protein	-0.94083	-2.1371	-3.36232
Potri.012 G031200	MYB	TRY	Homeodomain-like superfamily protein	-0.55693	3.627767	3.311767
Potri.005 G135300	Peroxidases	0	Peroxidase superfamily protein	0.590525	3.620506	3.045867
Potri.002 G065300	Peroxidases	0	Peroxidase superfamily protein	2.145887	0.97643	-0.88472
Potri.007 G096200	Peroxidases	0	Peroxidase superfamily protein	-2.09938	0.02891	-0.95437
Potri.006 G129900	Peroxidases	0	Peroxidase superfamily protein	3.479468	4.275797	3.131464
Potri.016 G125000	Peroxidases	0	Peroxidase superfamily protein	6.621506	7.491229	7.204969
Potri.001 G351000	Peroxidases	0	Peroxidase superfamily protein	4.793272	3.252365	2.893168
Potri.003 G214900	Peroxidases	0	Peroxidase superfamily protein	-3.87213	-0.72371	6.545228
Potri.004 G015300	Peroxidases	PRXR1	Peroxidase superfamily protein	2.059645	1.514349	2.933374
Potri.T04 5500	Peroxidases	0	Peroxidase superfamily protein	1.762111	1.392	2.698772
Potri.013 G083600	Peroxidases	0	Peroxidase superfamily protein	1.438483	2.621355	3.1773
Potri.001 G011500	Peroxidases	ATPA2,PA2	peroxidase 2	2.118798	4.053849	0.748336
Potri.005 G108900	Peroxidases	0	Peroxidase superfamily protein	3.505573	2.694245	2.269725
Potri.007	Peroxidases	RHS19	root hair specific 19	1.989279	0.69683	-0.32008

G053400						
Potri.008 G188000	Proteolysis	0	Protein of Unknown Function (DUF239)	-1.67063	-2.3902	-1.42554
Potri.006 G207900	Proteolysis	0	alpha/beta-Hydrolases superfamily protein	0.069259	0.942378	2.061009
Potri.001 G330800	Proteolysis	0	Peptidase family M48 family protein	-2.13627	0.165517	1.136541
Potri.004 G215400	Proteolysis	scpl48	serine carboxypeptidase-like 48	2.160966	1.909754	0.290184
Potri.006 G204700	Proteolysis	0	Eukaryotic aspartyl protease family protein	1.626392	2.286161	0.139256
Potri.018 G106800	Proteolysis	0	Cytosol aminopeptidase family protein	-2.44154	-1.77731	-1.97875
Potri.004 G208100	Proteolysis	0	Peptidase M20/M25/M40 family protein	12.1157	13.26467	16.23379
Potri.013 G120200	Proteolysis	SCPL19,SNG2	serine carboxypeptidase-like 19	1.205745	2.141767	2.552293
Potri.006 G153300	Proteolysis	0	Peptidase M28 family protein	1.359309	1.707441	2.74688
Potri.001 G212800	Proteolysis	0	Serine carboxypeptidase S28 family protein	3.221309	4.432941	5.829715
Potri.009 G028300	Proteolysis	0	Serine protease inhibitor, potato inhibitor I-type family protein	2.894731	6.257976	4.84285
Potri.007 G035900	Proteolysis	0	Prolyl oligopeptidase family protein	2.452155	1.855578	-0.45738
Potri.014 G074600	Proteolysis	ATSBT1.1,SBTI1.1	subtilase family protein	2.141771	1.566162	0.564111
Potri.002 G256300	Proteolysis	SDD1	Subtilase family protein	-2.69594	-1.91623	-3.49978
Potri.001 G163600	Proteolysis	0	Subtilase family protein	4.008454	4.164251	1.936129
Potri.011 G146300	Proteolysis	0	Subtilase family protein	1.157106	3.436629	1.40911

Potri.001 G440300	Proteolysis	0	Subtilase family protein	2.041847	2.121152	1.558792
Potri.011 G076700	Proteolysis	0	subtilase family protein	2.824227	3.003537	1.508543
Potri.002 G124500	Proteolysis	0	Subtilisin-like serine endopeptidase family protein	0.780854	3.341539	3.331167
Potri.014 G018900	Proteolysis	ARA12	Subtilase family protein	3.240826	2.839357	2.366898
Potri.005 G141600	Proteolysis	RD21,RD21A	Granulin repeat cysteine protease family protein	-0.53285	1.159913	2.176708
Potri.001 G435500	Proteolysis	0	OTU-like cysteine protease family protein	-0.4425	1.2075	2.051203
Potri.001 G032900	Proteolysis	ATCYS6,ATCYSB,CYSB	cystatin B	0.709746	0.936931	3.247121
Potri.006 G141700	Proteolysis	0	Cysteine proteinases superfamily protein	-2.84032	-2.11256	-1.28098
Potri.006 G057400	Proteolysis	0	Cysteine proteinases superfamily protein	3.267012	1.692922	1.450843
Potri.010 G234300	Proteolysis	0	Cysteine proteinases superfamily protein	0.160379	-1.14969	-2.19824
Potri.011 G066800	Proteolysis	0	Granulin repeat cysteine protease family protein	2.459027	4.968231	2.526883
Potri.002 G054900	Proteolysis	0	Eukaryotic aspartyl protease family protein	3.005543	1.016603	2.590378
Potri.001 G240600	Proteolysis	0	Eukaryotic aspartyl protease family protein	0.323474	1.454326	2.954911
Potri.001 G356900	Proteolysis	APA1,ATAPA1	aspartic proteinase A1	-2.18519	-0.83837	-0.69881
Potri.001 G041700	Proteolysis	0	Eukaryotic aspartyl protease family protein	3.165595	7.030823	5.251179
Potri.019 G002100	Proteolysis	0	Eukaryotic aspartyl protease family protein	4.902087	2.93792	0.975989
Potri.008	Proteolysis	0	Eukaryotic aspartyl protease family protein	2.608	1.833948	1.053817

G058000						
Potri.005 G063000	Proteolysis	0	Eukaryotic aspartyl protease family protein	5.611053	4.901412	2.065892
Potri.001 G158600	Proteolysis	0	Eukaryotic aspartyl protease family protein	4.850953	3.994962	1.867527
Potri.002 G092100	Proteolysis	0	Eukaryotic aspartyl protease family protein	2.653947	0.864128	0.32613
Potri.006 G036500	Proteolysis	SCPL45	serine carboxypeptidase-like 45	0.744004	2.292318	1.456356
Potri.001 G312800	Proteolysis	scpl18	serine carboxypeptidase-like 18	1.468107	2.783677	2.304062
Potri.014 G036000	Proteolysis	0	Serine protease inhibitor (SERPIN) family protein	-0.68634	-2.11289	-2.40646
Potri.009 G031900	Proteolysis	ATRBL1,RBL1	RHOMBOID-like 1	-2.02338	-0.92662	-0.39773
Potri.010 G220100	Proteolysis	SCPL27	serine carboxypeptidase-like 27	4.255183	3.009087	2.069277
Potri.014 G108900	Proteolysis	0	serine protease inhibitor, Kazal-type family protein	-20.1359	-1.51611	4.452087
Potri.009 G055900	Proteolysis	scpl40	serine carboxypeptidase-like 40	2.586251	4.654926	4.572328
Potri.014 G108800	Proteolysis	0	serine protease inhibitor, Kazal-type family protein	-17.9651	-3.78697	3.727544
Potri.007 G072300	Proteolysis	scpl35	serine carboxypeptidase-like 35	1.275519	3.803472	3.025027
Potri.017 G133200	Proteolysis	MAP2B	methionine aminopeptidase 2B	-0.77963	-0.81894	2.064231
Potri.017 G084000	Proteolysis	ATFTSH6,FTSH6	FTSH protease 6	3.616223	4.788874	5.186471
Potri.007 G019600	Proteolysis	0	P-loop containing nucleoside triphosphate hydrolases superfamily protein	0.020782	1.337779	2.369072
Potri.012 G020800	Proteolysis	0	P-loop containing nucleoside triphosphate hydrolases superfamily protein	3.208325	2.229535	1.301623

Potri.007G020900	Proteolysis	BCS1	cytochrome BC1 synthesis	2.47286	0.057075	0.66625
Potri.011G111200	Proteolysis	0	P-loop containing nucleoside triphosphate hydrolases superfamily protein	3.336157	-0.57194	-0.45497
Potri.006G169000	Proteolysis	0	P-loop containing nucleoside triphosphate hydrolases superfamily protein	1.066921	1.451322	2.870365
Potri.012G104100	Proteolysis	UBC27	ubiquitin-conjugating enzyme 27	-2.00914	-0.59939	-0.252
Potri.004G211600	Proteolysis	0	Ubiquitin-like superfamily protein	3.313599	5.150199	5.275691
Potri.004G021500	Proteolysis	UBQ10	polyubiquitin 10	-1.5671	-2.24598	-2.04164
Potri.001G101900	Proteolysis	UBC5	ubiquitin-conjugating enzyme 5	0.016412	1.675133	2.212024
Potri.004G043500	Proteolysis	ATUBC24,PHO2,UBC24	phosphate 2	3.847682	5.33189	4.869638
Potri.006G110200	Proteolysis	ATUBC8,UBC8	ubiquitin conjugating enzyme 8	-2.04951	-0.91302	-0.97179
Potri.008G053800	Proteolysis	ATUBC7,UBC7	ubiquitin carrier protein 7	-2.11198	-0.89148	-1.16905
Potri.005G081200	Proteolysis	RHA2A	RING-H2 finger A2A	0.989282	0.567595	2.769234
Potri.005G255200	Proteolysis	0	RING/U-box superfamily protein	-2.16202	-0.58992	-0.51812
Potri.010G113900	Proteolysis	0	ARM repeat superfamily protein	2.91967	2.659636	2.692164
Potri.008G177100	Proteolysis	0	ARM repeat superfamily protein	0.713869	-1.73774	-2.2824
Potri.008G087200	Proteolysis	0	RING/U-box superfamily protein	-2.20647	-1.80853	-2.1663
Potri.002G085800	Proteolysis	0	RING/U-box superfamily protein	0.019606	0.166503	1.883769
Potri.005	Proteolysis	0	RING/U-box superfamily protein	0.790978	1.70072	2.056868

G160900						
Potri.002 G101800	Proteolysis	0	RING/U-box superfamily protein	2.595851	1.760483	1.439983
Potri.001 G162000	Proteolysis	ATL3	RING/U-box superfamily protein	2.12036	0.352583	1.135067
Potri.002 G006400	Proteolysis	ATL8	RING/U-box superfamily protein	0.332307	-0.96498	-2.40128
Potri.005 G081300	Proteolysis	RHA2B	RING-H2 finger protein 2B	4.127514	1.499857	0.710289
Potri.004 G093500	Proteolysis	0	RING/FYVE/PHD zinc finger superfamily protein	1.827269	2.685498	2.586983
Potri.006 G190300	Proteolysis	0	RING/U-box superfamily protein	2.296677	2.58033	1.274451
Potri.018 G098000	Proteolysis	0	RING/U-box superfamily protein	3.40483	0.140711	0.39719
Potri.011 G068800	Proteolysis	0	RING/FYVE/PHD zinc finger superfamily protein	1.957075	1.8336	1.618879
Potri.003 G178700	Proteolysis	PRT1	proteolysis 1	3.529849	1.15883	2.039798
Potri.011 G050700	Proteolysis	0	RING/U-box superfamily protein	-1.99848	-0.20677	0.335333
Potri.006 G123500	Proteolysis	0	RING/U-box superfamily protein	-0.92714	2.239554	2.364949
Potri.002 G140600	Proteolysis	ATL4,TL4	TOXICOS EN LEVADURA 4	-3.26348	-2.51447	0.302815
Potri.002 G165200	Proteolysis	0	RING/U-box superfamily protein	-2.46071	-2.53645	-1.60528
Potri.004 G003100	Proteolysis	0	RING/U-box superfamily protein	1.615318	3.665581	2.660496
Potri.001 G304100	Proteolysis	0	RING/U-box superfamily protein	-1.75585	-2.06351	-2.26964
Potri.005 G228100	Proteolysis	0	ARM repeat superfamily protein	-2.50402	-1.61239	-2.10242

Potri.016 G115300	Proteolysis	0	RING/U-box superfamily protein	2.229955	1.286484	1.236632
Potri.016 G134000	Proteolysis	0	ARM repeat superfamily protein	5.898223	3.513231	3.472902
Potri.008 G062600	Proteolysis	0	RING/FYVE/PHD zinc finger superfamily protein	1.999292	0.648959	2.560776
Potri.016 G003000	Proteolysis	RGLG2	RING domain ligase2	2.689042	1.232658	0.706914
Potri.006 G002600	Proteolysis	RGLG2	RING domain ligase2	0.574718	0.97505	2.629988
Potri.018 G046900	Proteolysis	0	RING/U-box superfamily protein	2.625339	0.689262	-1.44753
Potri.006 G245400	Proteolysis	0	CHY-type/CTCHY-type/RING-type Zinc finger protein	-1.6262	-2.18723	-0.52628
Potri.015 G073600	Proteolysis	0	RING/U-box superfamily protein	-1.97798	-0.51335	0.242172
Potri.010 G072700	Proteolysis	0	RING/U-box superfamily protein	4.925523	0.194899	1.104014
Potri.007 G138700	Proteolysis	ATRING1A,RING1A	RING 1A	3.708149	0.244116	0.228209
Potri.002 G140500	Proteolysis	0	RING/U-box superfamily protein	-2.52537	-1.11649	-1.04003
Potri.001 G415600	Proteolysis	0	zinc finger protein-related	2.388902	3.014156	3.481739
Potri.012 G014700	Proteolysis	0	F-box family protein	-1.1924	-1.81849	-2.52381
Potri.001 G007000	Proteolysis	0	Galactose oxidase/kelch repeat superfamily protein	-1.98488	-2.04881	-1.10324
Potri.003 G171300	Proteolysis	0	Galactose oxidase/kelch repeat superfamily protein	-2.08295	-1.87012	-1.66969
Potri.009 G024000	Proteolysis	KUF1	KAR-UP F-box 1	0.856265	-1.06497	-2.55988
Potri.T04	Proteolysis	0	peptidoglycan-binding LysM domain-containing protein	-1.22655	-2.59525	-1.32803

8500						
Potri.008 G135200	Proteolysis	ADO3,FKF1	flavin-binding, kelch repeat, f box 1	-6.36688	-8.16825	-8.70483
Potri.005 G185700	Proteolysis	ATSKP2;2,SKP2B	RNI-like superfamily protein	-2.18879	-1.99072	-1.55772
Potri.009 G139500	Proteolysis	0	F-box/RNI-like superfamily protein	-1.87571	-2.30463	-1.95431
Potri.004 G199300	Proteolysis	0	F-box family protein	2.282807	2.376368	3.156236
Potri.001 G273900	Proteolysis	0	F-box family protein	4.648901	2.267141	1.116301
Potri.009 G047600	Proteolysis	0	F-box family protein	4.231489	2.467355	1.801236
Potri.014 G022100	Proteolysis	SLY1	F-box family protein	-1.98457	-0.23667	-0.50935
Potri.008 G217700	Proteolysis	ATCUL1,AXR6,CUL1	cullin 1	1.205537	2.526158	3.476284
Potri.013 G098900	PR-proteins	0	Leucine-rich repeat (LRR) family protein	3.613774	2.468576	1.106415
Potri.003 G216300	PR-proteins	0	Disease resistance-responsive (dirigent-like protein) family protein	2.900248	1.663943	1.691289
Potri.011 G046900	PR-proteins	0	disease resistance protein (TIR class), putative	4.708823	3.155639	1.804733
Potri.001 G096500	PR-proteins	0	Disease resistance-responsive (dirigent-like protein) family protein	1.490681	2.809667	2.00677
Potri.012 G028500	PR-proteins	AtRLP12,RLP12	receptor like protein 12	-1.72794	-1.73516	-3.73403
Potri.011 G014100	PR-proteins	0	Disease resistance protein (TIR-NBS class)	0.238536	1.316274	2.752822
Potri.011 G104600	PR-proteins	AtRLP19,RLP19	receptor like protein 19	2.856458	0.907162	-0.84622
Potri.T17 3200	PR-proteins	0	Disease resistance-responsive (dirigent-like protein) family protein	-0.9616	-1.89582	-2.53147

Potri.012 G007800	PR-proteins	AtRLP35,RLP35	receptor like protein 35	-2.2213	-1.81337	-3.3083
Potri.014 G003200	PR-proteins	0	LRR and NB-ARC domains-containing disease resistance protein	2.212277	1.368611	2.513278
Potri.013 G030600	PR-proteins	0	NB-ARC domain-containing disease resistance protein	-0.80298	-1.44991	-2.01092
Potri.010 G150500	PR-proteins	ATHS1,HS1	heat stable protein 1	2.991657	2.607335	2.286533
Potri.001 G003200	PR-proteins	AtRLP46,RLP46	receptor like protein 46	2.20961	1.400533	0.082818
Potri.005 G062700	PR-proteins	RLM3	disease resistance protein (TIR-NBS class), putative	3.078038	2.044284	0.74125
Potri.001 G426200	PR-proteins	0	NB-ARC domain-containing disease resistance protein	-0.16369	-0.32553	-3.03764
Potri.001 G406100	PR-proteins	0	NB-ARC domain-containing disease resistance protein	0.3007	-0.64842	-19.191
Potri.017 G103300	PR-proteins	0	disease resistance protein (TIR-NBS-LRR class), putative	2.69946	1.955342	2.499045
Potri.T00 5100	PR-proteins	0	Disease resistance protein (TIR-NBS-LRR class) family	0.55137	0.936613	3.080936
Potri.004 G230000	PR-proteins	0	Disease resistance protein (TIR-NBS-LRR class) family	0.780378	1.262345	2.09617
Potri.001 G309900	PR-proteins	0	Kunitz family trypsin and protease inhibitor protein	0.803369	3.793201	7.755307
Potri.004 G000400	PR-proteins	ATKTI1,KTI1	kunitz trypsin inhibitor 1	-4.79783	-1.96859	3.983675
Potri.019 G121900	PR-proteins	0	Kunitz family trypsin and protease inhibitor protein	4.539736	4.542392	2.103913
Potri.012 G053200	R genes	0	transmembrane receptors;ATP binding	2.386936	1.181356	0.904149
Potri.001 G416500	Redox state	ATCXXS1,CXXS1	C-terminal cysteine residue is changed to a serine 1	-4.96775	-2.99555	0.342861
Potri.001	Redox state	TTL1	tetratricopetide-repeat thioredoxin-like 1	3.456041	2.932299	1.781678

G392600						
Potri.008 G194100	Redox state	ATH8,TH8	thioredoxin H-type 8	3.442589	2.771258	2.799232
Potri.005 G245700	Redox state	ATCDSP32,CDSP32	chloroplastic drought-induced stress protein of 32 kD	0.257013	1.784387	2.064476
Potri.002 G040700	Redox state	0	2-oxoglutarate (2OG) and Fe(II)-dependent oxygenase superfamily protein	2.573661	-0.18421	-3.02669
Potri.006 G266200	Redox state	AtMAPR2,MAPR2	membrane-associated progesterone binding protein 2	1.923894	3.39773	3.716468
Potri.002 G242500	Redox state	ATCB5-B,B5 #4,CB5-B	cytochrome B5 isoform B	2.421217	1.594689	1.239333
Potri.004 G146000	Redox state	0	2-oxoglutarate (2OG) and Fe(II)-dependent oxygenase superfamily protein	8.003119	2.504317	1.752272
Potri.017 G111700	Redox state	ACYB-1,CYB-1	cytochrome B561-1	5.291638	5.347744	6.58252
Potri.005 G079400	Redox state	0	Cupredoxin superfamily protein	-0.45914	0.589711	2.668361
Potri.017 G125100	Redox state	DHAR3	dehydroascorbate reductase 1	2.202927	2.887488	3.055903
Potri.001 G219300	Redox state	0	Plant L-ascorbate oxidase	3.463427	3.381258	2.953864
Potri.001 G355600	Redox state	VTC2	mannose-1-phosphate guanylyltransferase (GDP)s;GDP-galactose:mannose-1-phosphate guanylyltransferases;GDP-galactose:glucose-1-phosphate guanylyltransferases;GDP-galactose:myoinositol-1-phosphate guanylyltransferases;glucose-1-phosphate guanylyltransferase	-2.07942	-0.69444	-0.42478
Potri.010 G156300	Redox state	VTC4	Inositol monophosphatase family protein	1.892728	2.12458	2.353627
Potri.007 G126600	Redox state	ATGPX2,GPX2	glutathione peroxidase 2	0.699779	1.884033	2.571629
Potri.003 G126100	Redox state	ATGPX6,GPX6,LSC803,PHGP X	glutathione peroxidase 6	-2.82426	-1.91961	-1.24238
Potri.005	Redox state	GSH2,GSHB	glutathione synthetase 2	0.506781	2.691988	2.673206

G038100						
Potri.009 G110800	Redox state	AHB1,ARATH GLB1,ATGLB1,GLB1,HB1,NSH B1	hemoglobin 1	3.570238	1.615449	-1.90162
Potri.006 G244300	Redox state	ATGLB3,GLB3	hemoglobin 3	3.066736	1.247609	1.547409
Potri.018 G071600	Redox state	0	SOUL heme-binding family protein	0.45166	2.119291	1.954755
Potri.003 G141800	Redox state	0	Glutaredoxin family protein	-3.22256	-0.32287	-0.26459
Potri.002 G208400	Redox state	0	Thioredoxin superfamily protein	-2.35495	-2.94773	-5.44536
Potri.014 G134200	Redox state	0	Thioredoxin superfamily protein	-3.17149	-2.28852	-2.90583
Potri.006 G137500	Redox state	ATPRX Q	Thioredoxin superfamily protein	1.752665	3.285174	3.394752
Potri.013 G031100	Redox state	CSD1	copper/zinc superoxide dismutase 1	-2.24514	-1.24018	-1.02149
Potri.001 G113800	Redox state	ATCCS,CCS	copper chaperone for SOD1	-2.26852	-1.75602	-1.03311
Potri.019 G057300	Redox state	ATMSD1,MEE33,MSD1	manganese superoxide dismutase 1	2.027434	0.359193	0.277322
Potri.005 G251600	Redox state	CAT2	catalase 2	-3.94973	-3.32395	-1.72632
Potri.005 G089600	Redox state	FSD3	Fe superoxide dismutase 3	0.847124	1.870748	2.159164
Potri.015 G110400	Redox state	FSD2	Fe superoxide dismutase 2	2.27213	1.862286	0.199144
Potri.006 G137300	Respiratory burst	0	Riboflavin synthase-like superfamily protein	2.48467	1.905265	0.742907
Potri.001 G098300	Respiratory burst	ATRBOH F,ATRBOHF,RBOH F,RBOHAP108,RBOHF	respiratory burst oxidase protein F	1.872623	3.292376	2.31439
Potri.003	Respiratory	ATRBOHD,RBOHD	respiratory burst oxidase homologue D	2.387431	1.134581	1.133992

G159800	burst					
Potri.007 G141700	SA	UGT74E2	Uridine diphosphate glycosyltransferase 74E2	-3.04856	-3.25528	-2.10415
Potri.008 G136200	SA	0	S-adenosyl-L-methionine-dependent methyltransferases superfamily protein	1.565382	2.963384	2.228441
Potri.017 G122700	SA	0	S-adenosyl-L-methionine-dependent methyltransferases superfamily protein	-2.75983	3.132572	4.396867
Potri.006 G218000	Secondary metabolites	FDH,KCS10	3-ketoacyl-CoA synthase 10	-1.03049	1.976533	2.700362
Potri.017 G090700	Secondary metabolites	MUB2	membrane-anchored ubiquitin-fold protein 2	2.218238	1.321678	1.237581
Potri.004 G150400	Secondary metabolites	CLB6,HDR,ISPH	4-hydroxy-3-methylbut-2-enyl diphosphate reductase	-5.34556	-1.27454	0.857556
Potri.015 G043400	Secondary metabolites	GGR	geranylgeranyl reductase	6.934292	1.032989	0.863395
Potri.001 G457000	Secondary metabolites	HMG1,HMGR1	hydroxy methylglutaryl CoA reductase 1	-1.94191	-2.12237	-2.68342
Potri.002 G057400	Secondary metabolites	HPD,PDS1	phytoene desaturation 1	-1.8222	-2.01397	-1.11536
Potri.003 G005800	Secondary metabolites	0	FAD/NAD(P)-binding oxidoreductase family protein	0.110467	2.44919	1.876445
Potri.017 G138900	Secondary metabolites	PSY	PHYTOENE SYNTHASE	2.876242	-0.701	0.0807
Potri.001 G265400	Secondary metabolites	ATCCD1,ATNCED1,CCD1,NCED1	carotenoid cleavage dioxygenase 1	1.525943	3.925043	4.00122
Potri.013 G053100	Secondary metabolites	AVDE1,NPQ1	non-photochemical quenching 1	0.739058	1.900578	2.304143
Potri.004 G037900	Secondary metabolites	GES,TPS04,TPS4	terpene synthase 04	2.27229	4.871845	4.80818
Potri.007 G118600	Secondary metabolites	ATTPS03,TPS03	terpene synthase 03	4.446896	3.661573	3.845547
Potri.005 G095500	Secondary metabolites	ATTPS21,TPS21	terpene synthase 21	-0.54852	-4.13555	-1.3614

Potri.007 G074400	Secondary metabolites	ATTPS21,TPS21	terpene synthase 21	2.674343	-0.34919	-0.74188
Potri.002 G012800	Secondary metabolites	OPCL1	OPC-8:0 CoA ligase1	-1.66336	-4.28646	-6.20927
Potri.004 G053500	Secondary metabolites	0	HXXXD-type acyl-transferase family protein	0.760598	2.360074	2.371432
Potri.001 G133200	Secondary metabolites	ATPRR1,PRR1	pinoresinol reductase 1	-2.89887	1.632273	1.340462
Potri.010 G186300	Secondary metabolites	0	HXXXD-type acyl-transferase family protein	2.240674	2.93133	1.408841
Potri.009 G139700	Secondary metabolites	0	O-methyltransferase family protein	3.912829	5.096629	4.008487
Potri.016 G112400	Secondary metabolites	0	HXXXD-type acyl-transferase family protein	4.058582	1.954657	0.17152
Potri.016 G091100	Secondary metabolites	ATPAL1,PAL1	PHE ammonia lyase 1	1.471383	2.06967	0.593173
Potri.018 G146100	Secondary metabolites	ATC4H,C4H,CYP73A5,REF3	cinnamate-4-hydroxylase	5.228671	1.507638	0.204164
Potri.006 G169600	Secondary metabolites	4CL2,AT4CL2	4-coumarate:CoA ligase 2	3.911596	7.047617	2.384994
Potri.004 G102000	Secondary metabolites	0	AMP-dependent synthetase and ligase family protein	-0.77955	-0.17535	-2.87743
Potri.010 G057000	Secondary metabolites	0	AMP-dependent synthetase and ligase family protein	4.617093	0.529551	2.559783
Potri.018 G104700	Secondary metabolites	HCT	hydroxycinnamoyl-CoA shikimate/quinate hydroxycinnamoyl transferase	-3.11702	-0.83996	0.46839
Potri.001 G046100	Secondary metabolites	ATCCR1,CCR1,IRX4	cinnamoyl coa reductase 1	3.065849	1.449449	-0.37034
Potri.007 G016400	Secondary metabolites	CYP84A1,FAH1	ferulic acid 5-hydroxylase 1	-2.9057	-1.04299	-0.62701
Potri.011 G150500	Secondary metabolites	ATOMT1,OMT1	O-methyltransferase 1	-2.43606	-2.10255	-2.14404
Potri.006	Secondary	ATCAD1,CAD1	cinnamyl-alcohol dehydrogenase	-4.45293	-4.41798	-2.73562

G024300	metabolites					
Potri.001 G268600	Secondary metabolites	ATCAD7,CAD7,ELI3,ELI3-1	elicitor-activated gene 3-1	4.325405	-0.11996	-0.10295
Potri.011 G158500	Secondary metabolites	0	FAD-binding Berberine family protein	1.059837	1.502704	2.192316
Potri.006 G040900	Secondary metabolites	SSL2	strictosidine synthase-like 2	1.636218	1.543991	2.059355
Potri.008 G172500	Secondary metabolites	ATLEUC1,IIL1	isopropyl malate isomerase large subunit 1	0.055728	-2.91537	-1.64425
Potri.012 G032700	Secondary metabolites	ATSOT16,ATST5A,CORI-7,SO T16	sulfotransferase 16	-5.14343	-0.59208	-0.37013
Potri.002 G025300	Secondary metabolites	ATR4,CYP83B1,RED1,RNT1,S UR2	cytochrome P450, family 83, subfamily B, polypeptide 1	-2.66757	-0.07554	1.259191
Potri.002 G064000	Secondary metabolites	0	Pyridoxal phosphate (PLP)-dependent transferases superfamily protein	-2.32398	0.230799	0.197661
Potri.014 G180300	Secondary metabolites	CER1	Fatty acid hydroxylase superfamily	3.027717	0.852003	0.334315
Potri.009 G116700	Secondary metabolites	KCS4	3-ketoacyl-CoA synthase 4	2.822118	1.516643	1.21255
Potri.001 G106800	Secondary metabolites	ASAT1,ATASAT1,ATSAT1	acyl-CoA sterol acyl transferase 1	2.608794	1.318452	0.558371
Potri.001 G007100	Secondary metabolites	0	2-oxoglutarate (2OG) and Fe(II)-dependent oxygenase superfamily protein	2.084582	-1.83029	-0.16873
Potri.002 G162300	Secondary metabolites	0	UDP-Glycosyltransferase superfamily protein	-3.45405	2.964579	3.505973
Potri.010 G192400	Secondary metabolites	0	HXXXD-type acyl-transferase family protein	2.285344	-0.26848	1.320665
Potri.006 G101100	Secondary metabolites	0	2-oxoglutarate (2OG) and Fe(II)-dependent oxygenase superfamily protein	9.320405	-0.12435	1.324588
Potri.001 G113100	Secondary metabolites	ANS,LDOX,TDS4,TT18	leucoanthocyanidin dioxygenase	2.016293	8.544129	4.337345
Potri.008 G024800	Secondary metabolites	0	UDP-Glycosyltransferase superfamily protein	0.596996	3.536339	4.908728

Potri.001 G455200	Secondary metabolites	0	HXXXD-type acyl-transferase family protein	2.074138	-1.57213	1.231352
Potri.010 G213000	Secondary metabolites	A11,CFI,TT5	Chalcone-flavanone isomerase family protein	-0.25931	3.390689	1.998921
Potri.009 G044600	Secondary metabolites	UGT71D1	UDP-glucosyl transferase 71D1	-1.66092	-2.49363	-1.88653
Potri.002 G168600	Secondary metabolites	GT72B1,UGT72B1	UDP-Glycosyltransferase superfamily protein	-2.49691	-0.94204	-2.15702
Potri.011 G150100	Secondary metabolites	0	2-oxoglutarate (2OG) and Fe(II)-dependent oxygenase superfamily protein	-1.36249	2.725753	5.544657
Potri.002 G127400	Secondary metabolites	FLDH	NAD(P)-binding Rossmann-fold superfamily protein	-0.10734	1.463351	2.582512
Potri.002 G033600	Secondary metabolites	DFR,M318,TT3	dihydroflavonol 4-reductase	3.174246	10.89437	7.462899
Potri.006 G141400	Secondary metabolites	CYP75B1,D501,TT7	Cytochrome P450 superfamily protein	1.584049	5.106828	7.326427
Potri.001 G355100	Secondary metabolites	ATSRG1,SRG1	senescence-related gene 1	-1.74637	-3.48929	0.288946
Potri.006 G062400	Secondary metabolites	0	2-oxoglutarate (2OG) and Fe(II)-dependent oxygenase superfamily protein	-0.66077	1.440449	1.916748
Potri.010 G023600	Secondary metabolites	0	2-oxoglutarate (2OG) and Fe(II)-dependent oxygenase superfamily protein	6.827148	6.464105	4.646901
Potri.009 G025900	Secondary metabolites	0	2-oxoglutarate (2OG) and Fe(II)-dependent oxygenase superfamily protein	1.479175	2.492286	2.007386
Potri.002 G127400	Secondary metabolites	FLDH	NAD(P)-binding Rossmann-fold superfamily protein	-0.10734	1.463351	2.582512
Potri.013 G118700	Secondary metabolites	UGT78D2	UDP-glucosyl transferase 78D2	11.98996	22.54325	17.57892
Potri.002 G034400	Secondary metabolites	0	NmrA-like negative transcriptional regulator family protein	2.823511	0.52066	0.426782
Potri.009 G118100	Secondary metabolites	0	NmrA-like negative transcriptional regulator family protein	4.317241	1.272588	0.092442
Potri.019	Secondary	LAC3	laccase 3	4.086505	2.458609	1.866645

G121700	metabolites					
Potri.006 G096900	Secondary metabolites	ATLMCO4,IRX12,LAC4,LMCO 4	Laccase/Diphenol oxidase family protein	5.905893	2.873437	2.788323
Potri.008 G073700	Secondary metabolites	LAC5	laccase 5	3.615142	1.619032	0.830481
Potri.008 G073800	Secondary metabolites	LAC12	laccase 12	5.346381	3.850419	2.38143
Potri.011 G071100	Secondary metabolites	LAC14	laccase 14	9.107924	9.898919	16.96742
Potri.006 G087100	Secondary metabolites	ATLAC17,LAC17	laccase 17	4.661772	-0.03738	-0.67275
Potri.007 G064300	Signaling	ATMLO8,MLO8	Seven transmembrane MLO family protein	-3.92394	-0.06592	0.023249
Potri.006 G129700	Signaling	ATMLO12,MLO12	Seven transmembrane MLO family protein	-1.87385	-2.41358	-1.23117
Potri.006 G172100	Signaling	ATGLR2.2,GLR2.2	glutamate receptor 2.2	2.195217	-1.82639	-2.88667
Potri.006 G268200	Signaling	ATGLR2.8,GLR2.8	glutamate receptor 2.8	3.9044	3.59886	4.459353
Potri.001 G374300	Signaling	ATGLR2.7,GLR2.7	glutamate receptor 2.7	2.091625	3.779418	3.346147
Potri.013 G090800	Signaling	AKIN10,KIN10,SNRK1.1	SNF1 kinase homolog 10	-2.32198	0.147811	0.474512
Potri.006 G094300	Signaling	0	PAR1 protein	2.653748	0.156016	1.130636
Potri.002 G098600	Signaling	EXO	Phosphate-responsive 1 family protein	2.135486	0.528899	-1.40464
Potri.012 G141800	Signaling	0	PAR1 protein	5.059159	4.626097	3.475231
Potri.001 G311700	Signaling	EXL2	EXORDIUM like 2	-0.79796	-1.508	-2.19703
Potri.001 G144100	Signaling	PIRL4	plant intracellular ras group-related LRR 4	-2.17801	-1.39504	-0.06653

Potri.019 G094200	Signaling	0	Leucine-rich repeat transmembrane protein kinase protein	0.650324	2.142572	2.431255
Potri.004 G108200	Signaling	NIK1	NSP-interacting kinase 1	1.880904	1.526925	2.174529
Potri.006 G228400	Signaling	0	leucine-rich repeat transmembrane protein kinase family protein	-1.42292	-0.56699	-3.64448
Potri.004 G095700	Signaling	0	Leucine-rich repeat protein kinase family protein	2.743736	3.001641	2.208366
Potri.002 G251700	Signaling	TMKL1	transmembrane kinase-like 1	2.666852	3.501114	3.166535
Potri.006 G057500	Signaling	0	Leucine-rich repeat protein kinase family protein	3.882314	4.110298	4.910517
Potri.011 G163700	Signaling	0	Leucine-rich receptor-like protein kinase family protein	2.871403	1.801544	1.696173
Potri.001 G095200	Signaling	0	Leucine-rich repeat protein kinase family protein	2.285283	2.180003	2.444817
Potri.007 G048800	Signaling	0	Leucine-rich repeat protein kinase family protein	2.201918	2.149427	1.181425
Potri.018 G045500	Signaling	0	Leucine-rich receptor-like protein kinase family protein	2.398129	2.056526	1.915228
Potri.014 G068700	Signaling	0	Leucine-rich repeat protein kinase family protein	6.669272	5.428672	5.266444
Potri.001 G117800	Signaling	0	Leucine-rich repeat protein kinase family protein	-1.25169	0.159987	2.065349
Potri.001 G161600	Signaling	SRF6	STRUBBELIG-receptor family 6	2.82432	0.993948	1.2518
Potri.013 G144600	Signaling	SRF3	STRUBBELIG-receptor family 3	-2.47217	-1.78979	-0.28286
Potri.004 G005400	Signaling	SRF8	STRUBBELIG-receptor family 8	2.185482	0.383571	0.979817
Potri.004 G005300	Signaling	SRF8	STRUBBELIG-receptor family 8	2.779226	1.26757	1.768376
Potri.013	Signaling	0	Leucine-rich repeat protein kinase family protein	2.047643	0.401316	0.834125

G064300						
Potri.015 G093100	Signaling	0	Leucine-rich repeat protein kinase family protein	3.390564	2.545743	0.838758
Potri.001 G280200	Signaling	0	Leucine-rich repeat protein kinase family protein	2.58779	2.020983	1.977811
Potri.003 G117500	Signaling	0	Leucine-rich repeat protein kinase family protein	2.314752	-0.53533	0.219378
Potri.014 G147300	Signaling	0	Leucine-rich receptor-like protein kinase family protein	-0.37408	2.378978	2.934808
Potri.016 G140100	Signaling	0	Leucine-rich repeat protein kinase family protein	-3.03453	-2.57246	-4.14984
Potri.003 G046400	Signaling	0	Leucine-rich repeat protein kinase family protein	1.960452	1.949137	2.01871
Potri.004 G135500	Signaling	0	Leucine-rich repeat transmembrane protein kinase	4.096385	3.489147	2.113464
Potri.001 G393200	Signaling	0	Protein kinase superfamily protein	1.804084	4.204717	1.98423
Potri.T00 8700	Signaling	0	Leucine-rich repeat transmembrane protein kinase	-1.63402	-2.17763	-1.05186
Potri.010 G155100	Signaling	0	Leucine-rich repeat transmembrane protein kinase	0.68683	2.51397	2.846348
Potri.011 G072300	Signaling	RKF1	receptor-like kinase in flowers 1	1.075363	2.47012	2.785311
Potri.001 G385600	Signaling	0	Leucine-rich repeat transmembrane protein kinase	2.955966	2.588065	1.587859
Potri.011 G106400	Signaling	0	Leucine-rich repeat transmembrane protein kinase	3.657859	3.873765	4.372594
Potri.006 G255100	Signaling	0	Leucine-rich repeat transmembrane protein kinase	-0.90283	1.471525	2.736853
Potri.011 G049600	Signaling	0	Protein kinase superfamily protein	4.023055	1.933915	1.453163
Potri.019 G004800	Signaling	0	Leucine-rich repeat transmembrane protein kinase	1.296348	-0.80044	-3.63088

Potri.009 G020400	Signaling	TMK1	transmembrane kinase 1	3.240371	2.760905	1.36134
Potri.016 G070500	Signaling	0	Leucine-rich repeat protein kinase family protein	2.73756	3.558437	3.459472
Potri.001 G217700	Signaling	0	Leucine-rich repeat protein kinase family protein	5.158705	4.16877	4.484835
Potri.011 G169600	Signaling	BRL1	BRI1 like	2.040782	0.260351	0.555296
Potri.010 G097700	Signaling	ATPSKR1,PSKR1	phytosulfokin receptor 1	3.380785	0.062284	-0.66665
Potri.006 G051700	Signaling	0	Leucine-rich repeat protein kinase family protein	3.842428	3.222866	2.827711
Potri.009 G081800	Signaling	LRR XI-23,RLK7	Leucine-rich receptor-like protein kinase family protein	3.19344	1.721744	1.217071
Potri.011 G169600	Signaling	BRL1	BRI1 like	2.040782	0.260351	0.555296
Potri.019 G078400	Signaling	0	Leucine-rich receptor-like protein kinase family protein	2.306279	2.250665	2.253966
Potri.008 G009300	Signaling	ATPEPR1,PEPR1	PEP1 receptor 1	-0.40443	-2.74359	-2.88675
Potri.012 G088900	Signaling	GSO1	Leucine-rich repeat transmembrane protein kinase	2.347923	2.167785	2.028474
Potri.001 G467300	Signaling	0	Leucine-rich receptor-like protein kinase family protein	3.457728	2.476405	1.950586
Potri.003 G107600	Signaling	PXY	Leucine-rich repeat protein kinase family protein	2.363707	1.123654	0.202665
Potri.013 G051300	Signaling	0	Leucine-rich repeat protein kinase family protein	-1.07227	-1.24962	-2.07559
Potri.006 G099100	Signaling	0	Leucine-rich repeat protein kinase family protein	-2.22399	-1.95099	-2.23754
Potri.012 G124200	Signaling	0	Leucine-rich repeat receptor-like protein kinase family protein	-1.43021	0.306798	2.255522
Potri.017	Signaling	EFR	EF-TU receptor	-1.06511	4.056305	8.982568

G150800						
Potri.004 G065400	Signaling	FLS2	Leucine-rich receptor-like protein kinase family protein	2.904041	0.534982	-0.2444
Potri.004 G014700	Signaling	PR5K	PR5-like receptor kinase	3.638439	2.896923	3.008049
Potri.005 G144100	Signaling	0	Protein kinase superfamily protein	-0.51542	-1.02521	-2.03046
Potri.018 G091000	Signaling	0	Protein kinase superfamily protein	0.662825	1.166781	2.184974
Potri.008 G133500	Signaling	PDLP6	plasmodesmata-located protein 6	2.320957	2.028259	0.170239
Potri.011 G028800	Signaling	CRK29	cysteine-rich RLK (RECEPTOR-like protein kinase) 29	2.804122	1.063579	-0.64331
Potri.004 G026400	Signaling	CRK10,RLK4	cysteine-rich RLK (RECEPTOR-like protein kinase) 10	-3.17488	1.291974	3.063542
Potri.004 G209300	Signaling	0	Concanavalin A-like lectin protein kinase family protein	0.055344	1.042596	3.092079
Potri.005 G189500	Signaling	0	Concanavalin A-like lectin protein kinase family protein	4.665223	0.253099	-1.18979
Potri.012 G054700	Signaling	0	Protein kinase superfamily protein	2.242836	2.77857	2.240731
Potri.017 G034600	Signaling	0	Protein kinase superfamily protein	-2.39604	-2.85651	-1.80253
Potri.015 G018000	Signaling	0	Protein kinase superfamily protein	-0.51985	-0.01796	2.030998
Potri.T06 4400	Signaling	0	Protein kinase superfamily protein	1.51618	1.940621	3.43925
Potri.001 G200000	Signaling	0	Protein kinase protein with adenine nucleotide alpha hydrolases-like domain	-0.56678	2.169249	2.208159
Potri.005 G185500	Signaling	0	Protein kinase superfamily protein	13.54451	19.6599	20.12592
Potri.005 G181800	Signaling	0	Protein kinase superfamily protein	2.886759	-0.49429	-0.60186

Potri.011 G037700	Signaling	CBRLK1,RKS2,SD1-13	S-domain-1 13	3.182549	1.576286	-4.13147
Potri.019 G086200	Signaling	0	lectin protein kinase family protein	-0.90441	-2.55447	-3.94972
Potri.001 G409300	Signaling	ARK3,RK3	receptor kinase 3	2.305893	1.087844	2.125318
Potri.004 G027800	Signaling	B120	S-locus lectin protein kinase family protein	2.63573	1.040992	0.96253
Potri.001 G412000	Signaling	0	S-locus lectin protein kinase family protein	-3.32513	-1.14516	2.719433
Potri.T02 3800	Signaling	0	S-locus lectin protein kinase family protein	3.749601	3.023137	2.312935
Potri.003 G185700	Signaling	0	Wall-associated kinase family protein	-0.17576	2.250296	2.833983
Potri.001 G040000	Signaling	0	Wall-associated kinase family protein	1.638552	1.478239	3.104135
Potri.010 G221200	Signaling	ATCRR2,CCR2	CRINKLY4 related 2	4.360744	3.446375	0.908383
Potri.010 G188600	Signaling	ATCRR3,CCR3	CRINKLY4 related 3	2.072001	-1.21376	-2.1526
Potri.006 G098200	Signaling	AHP5	histidine-containing phosphotransfer factor 5	-2.04051	-1.12822	-0.48767
Potri.005 G043700	Signaling	0	Leucine-rich repeat transmembrane protein kinase protein	7.076084	7.085089	5.574805
Potri.019 G109800	Signaling	0	Leucine-rich repeat protein kinase family protein	0.312334	1.996112	2.504759
Potri.009 G010400	Signaling	NCRK	Protein kinase superfamily protein	4.313684	3.685587	2.748282
Potri.010 G010100	Signaling	AtRLP27,RLP27	receptor like protein 27	-1.87563	-2.09578	-2.88378
Potri.009 G100400	Signaling	0	Protein kinase superfamily protein	2.084342	1.435002	2.209944
Potri.007	Signaling	0	Receptor-like protein kinase-related family protein	3.764335	6.650095	7.154958

G120400						
Potri.016 G129500	Signaling	0	Nuclear transport factor 2 (NTF2) family protein	5.363302	3.538719	0.74077
Potri.016 G051600	Signaling	0	Leucine-rich repeat receptor-like protein kinase family protein	2.14866	2.785128	5.536722
Potri.011 G058300	Signaling	0	Protein kinase superfamily protein	2.228897	0.967147	0.13341
Potri.012 G048200	Signaling	0	Calcium-binding EF-hand family protein	-1.82877	-2.07514	-2.54994
Potri.014 G030100	Signaling	0	Calcium-binding EF-hand family protein	-5.28615	-4.31317	-4.05469
Potri.014 G030700	Signaling	ATCDPK1,CDPK1,CPK10	calcium-dependent protein kinase 1	-1.24725	-3.09874	-1.27917
Potri.010 G107100	Signaling	0	EF hand calcium-binding protein family	0.182712	4.25251	4.044529
Potri.002 G017000	Signaling	CPK29	calcium-dependent protein kinase 29	0.344815	0.905432	2.260967
Potri.005 G259900	Signaling	CML38	calmodulin-like 38	-2.08894	-1.82514	-0.50665
Potri.004 G143500	Signaling	0	Protein of unknown function (DUF1645)	2.946505	1.598462	1.744825
Potri.004 G202200	Signaling	0	Calcium-binding EF-hand family protein	1.264561	0.599575	2.878567
Potri.007 G127000	Signaling	CPK24	calcium-dependent protein kinase 24	2.566071	1.705505	0.717518
Potri.002 G055200	Signaling	NPG1	tetratricopeptide repeat (TPR)-containing protein	4.146265	3.956399	3.334264
Potri.007 G128600	Signaling	MSS3	Calcium-binding EF-hand family protein	3.464694	4.162775	1.213334
Potri.014 G101700	Signaling	0	Calcium-binding EF-hand family protein	3.336504	3.558793	2.87671
Potri.001 G375700	Signaling	IQD10	IQ-domain 10	5.541341	3.497106	1.711975

Potri.008 G159100	Signaling	0	ATPase E1-E2 type family protein / haloacid dehalogenase-like hydrolase family protein	-1.54114	-1.50249	2.726807
Potri.005 G215700	Signaling	CML11	calmodulin-like 11	2.783759	2.736153	3.931461
Potri.015 G145900	Signaling	0	alpha/beta-Hydrolases superfamily protein	3.533363	1.800785	1.451927
Potri.005 G128100	Signaling	CML41	calmodulin-like 41	0.534144	2.82009	2.55821
Potri.016 G034100	Signaling	0	IQ calmodulin-binding motif family protein	2.789228	3.435806	2.649549
Potri.005 G215600	Signaling	0	ATPase E1-E2 type family protein / haloacid dehalogenase-like hydrolase family protein	2.536616	0.064754	-0.00743
Potri.002 G178500	Signaling	iqd17	IQ-domain 17	2.177282	0.274515	-0.11068
Potri.004 G015500	Signaling	CPK21	calcium-dependent protein kinase 21	2.05955	3.943273	3.952039
Potri.011 G022300	Signaling	ATEHD2,EHD2	EPS15 homology domain 2	1.982804	1.105676	1.30064
Potri.019 G083200	Signaling	ATCPK4,CPK4	calcium-dependent protein kinase 4	0.763805	2.190746	3.147202
Potri.005 G052800	Signaling	CAM8	calmodulin 8	2.500045	1.382993	-2.45883
Potri.003 G122800	Signaling	IQD22	IQ-domain 22	-2.70056	-1.84024	-1.48989
Potri.013 G010700	Signaling	0	Calmodulin-binding protein	-2.49396	-0.98629	0.041763
Potri.004 G027200	Signaling	0	Calcium-binding EF-hand family protein	13.77796	16.54343	16.06729
Potri.018 G061900	Signaling	EDA39	calmodulin-binding family protein	2.111094	0.661671	0.701495
Potri.004 G166900	Signaling	0	Calcium-binding EF-hand family protein	-3.05591	-2.32095	-1.74422
Potri.006	Signaling	iqd2	IQ-domain 2	1.805724	2.480492	2.484329

G131100						
Potri.010 G223900	Signaling	ATSYTC,NTMC2T1.3,NTMC2TYPE1.3,SYT3,SYTC	Calcium-dependent lipid-binding (CaLB domain) family protein	0.216257	1.229073	2.542103
Potri.001 G021000	Signaling	IQD11	IQ-domain 11	4.393975	2.387144	1.679696
Potri.003 G141400	Signaling	ATSOS3,CBL4,SOS3	Calcium-binding EF-hand family protein	1.98793	1.848726	3.048727
Potri.002 G127000	Signaling	CML37,CML39	calmodulin like 37	-3.75206	-2.22808	-2.18941
Potri.012 G007600	Signaling	0	Calcium-binding endonuclease/exonuclease/phosphatase family	2.983809	3.275994	2.016403
Potri.013 G151000	Signaling	PBP1	pinoid-binding protein 1	1.620091	0.339364	3.428321
Potri.005 G020200	Signaling	0	Calmodulin-binding protein	-2.1561	0.585647	1.591298
Potri.001 G070600	Signaling	ATCNX1,CNX1	calnexin 1	-1.71106	-1.25946	-1.99187
Potri.012 G077000	Signaling	0	Calmodulin binding protein-like	-2.00845	-0.09668	-0.97956
Potri.004 G139900	Signaling	ATPIP5K1,ATPIPK1,PIP5K1	phosphatidylinositol-4-phosphate 5-kinase 1	2.33008	1.305351	1.042925
Potri.013 G116400	Signaling	FAB1D	FORMS APLOID AND BINUCLEATE CELLS 1A	2.227454	0.326778	0.276506
Potri.003 G196000	Signaling	0	Phosphatidylinositol-4-phosphate 5-kinase family protein	15.71545	12.97096	12.46217
Potri.010 G188900	Signaling	0	Phosphoinositide-specific phospholipase C family protein	0.204589	0.399551	2.305603
Potri.009 G046600	Signaling	ATPLC2,PLC2	phospholipase C 2	-0.70927	1.908045	3.123607
Potri.008 G068400	Signaling	ATPLC2,PLC2	phospholipase C 2	2.13096	1.192037	0.903053
Potri.001 G252100	Signaling	ATPLC4,PLC4	phosphatidylinositol-speciwc phospholipase C4	3.876907	3.92817	2.679952

Potri.009 G084600	Signaling	0	Inositol 1,3,4-trisphosphate 5/6-kinase family protein	-0.21472	4.231438	4.077669
Potri.003 G106400	Signaling	ATROPGEF14,ROPGEF14	RHO guanyl-nucleotide exchange factor 14	-0.58226	1.951121	0.362348
Potri.008 G089300	Signaling	emb1579	ATP/GTP-binding protein family	2.560538	0.83097	2.196344
Potri.014 G103600	Signaling	0	Rho GTPase activating protein with PAK-box/P21-Rho-binding domain	3.105633	2.691784	2.424562
Potri.002 G234600	Signaling	SCN1	Immunoglobulin E-set superfamily protein	1.916457	3.661686	1.920343
Potri.001 G172700	Signaling	0	Ran BP2/NZF zinc finger-like superfamily protein	4.672205	1.491475	0.898262
Potri.002 G081500	Signaling	AGG1,ATAGG1,GG1	Ggamma-subunit 1	0.13057	2.261153	2.713599
Potri.005 G198800	Signaling	ATRABG3A,RABG3A	RAB GTPase homolog G3A	1.996115	1.860844	1.654274
Potri.006 G069300	Signaling	0	Ypt/Rab-GAP domain of gyp1p superfamily protein	-2.252	-1.29841	-0.54206
Potri.002 G014700	Signaling	0	SEC14 cytosolic factor family protein / phosphoglyceride transfer family protein	-0.45591	1.553935	2.195322
Potri.016 G097800	Signaling	ATRAB,ATRAB ALPHA,ATRAB18B,ATRABC2A ,RABC2A	RAB GTPase homolog C2A	2.119816	1.96652	1.938724
Potri.008 G062000	Signaling	ATROPGEF5,ROPGEF5	ROP guanine nucleotide exchange factor 5	0.826017	1.177681	2.476998
Potri.005 G075300	Signaling	AtRABH1e,RABH1e	RAB GTPase homolog H1E	1.768786	1.299838	2.064263
Potri.004 G201600	Signaling	0	Rho GTPase activating protein with PAK-box/P21-Rho-binding domain	3.126438	3.348959	4.568588
Potri.006 G251300	Signaling	0	Ran BP2/NZF zinc finger-like superfamily protein	-0.91604	-1.53225	-2.28668
Potri.011 G061500	Signaling	ARAC2,ATRAC2,ATROP7,RAC 2,ROP7	RAC-like 2	3.20743	1.979693	1.896178

Potri.017 G016800	Signaling	0	Guanylate-binding family protein	0.834633	1.680615	2.543839
Potri.016 G010300	Signaling	AtRABA5a,RABA5a	RAB GTPase homolog A5A	1.245451	3.743244	2.866915
Potri.015 G105500	Signaling	AGD1	ARF-GAP domain 1	3.544853	1.758743	1.639681
Potri.014 G155000	Signaling	MAPKKK18	mitogen-activated protein kinase kinase kinase 18	3.04798	-1.97003	-1.67417
Potri.015 G030700	Signaling	ATMKK9,MKK9	MAP kinase kinase 9	-1.65873	-2.02609	-3.72388
Potri.005 G201800	Signaling	ATMPK20,MPK20	MAP kinase 20	1.305015	2.099663	2.558762
Potri.001 G085500	Signaling	ATWNK8,WNK8	with no lysine (K) kinase 8	0.220466	1.747309	2.211977
Potri.019 G128600	Signaling	WNK4,ZIK2	with no lysine (K) kinase 4	0.635872	1.623066	2.437614
Potri.005 G140100	Signaling	0	PLC-like phosphodiesterases superfamily protein	1.702752	2.408474	1.483632
Potri.002 G097500	Signaling	GF14 IOTA,GRF12	general regulatory factor 12	-1.54305	-2.69653	-3.54957
Potri.009 G097100	Signaling	0	Leucine-rich repeat (LRR) family protein	2.152994	0.240686	-0.74479
Potri.017 G059500	Signaling	RALFL31	ralf-like 31	2.32378	3.005981	3.191825
Potri.001 G321500	Signaling	RALFL32	ralf-like 32	0.866403	4.267407	3.500471
Potri.005 G139100	Signaling	RALFL34	ralf-like 34	7.302853	6.381264	4.04739
Potri.018 G046800	Signaling	AHP4	HPT phosphotransmitter 4	3.176004	1.265941	2.385154
Potri.010 G027100	Signaling	AHP1	histidine-containing phosphotransmitter 1	0.402242	2.348019	2.633576
Potri.014	Signaling	PAP3,PIF3,POC1	phytochrome interacting factor 3	-2.21112	-1.45023	-1.77591

G111400						
Potri.010 G053500	Signaling	PLP,PLPA,PLPB,PLPC	PAS/LOV protein B	-2.62665	-2.16507	-1.21938
Potri.015 G062600	Signaling	PKS1	phytochrome kinase substrate 1	-2.16031	-1.8622	-1.18393
Potri.005 G146400	Signaling	NPY2	Phototropic-responsive NPH3 family protein	2.351638	1.355809	0.673123
Potri.007 G128800	Signaling	0	Far-red impaired responsive (FAR1) family protein	1.5019	1.163793	0.901309
Potri.004 G189800	Signaling	0	Phototropic-responsive NPH3 family protein	5.988664	2.532106	1.049186
Potri.007 G128700	Signaling	0	Far-red impaired responsive (FAR1) family protein	0.085589	1.247041	2.111513
Potri.006 G264300	Signaling	ENP,MAB4,NPY1	Phototropic-responsive NPH3 family protein	1.11479	2.458163	2.109186
Potri.015 G139300	Signaling	FRS5	FAR1-related sequence 5	1.331176	2.153939	2.458412
Potri.009 G157600	Signaling	LSH1	Protein of unknown function (DUF640)	3.095043	1.554312	-0.2046
Potri.016 G003700	Signaling	0	Phototropic-responsive NPH3 family protein	2.102231	1.371513	0.626503
Potri.001 G415200	Signaling	PAT1	GRAS family transcription factor	-0.20283	-1.89565	-2.8508
Potri.005 G130700	Signaling	0	Phototropic-responsive NPH3 family protein	3.449455	2.6828	2.0546
Potri.005 G246600	Signaling	MSL4	mechanosensitive channel of small conductance-like 4	2.749225	2.920227	2.089699
Potri.001 G168200	Transcription factors	AT-RSH3,ATRSH3,RSH3	RELA/SPOT homolog 3	3.088173	0.969607	0.273118
Potri.004 G060400	WRKY	ATWRKY65,WRKY65	WRKY DNA-binding protein 65	-2.02041	0.003011	0.243708
Potri.011 G157100	WRKY	AR411,ATWRKY14,WRKY14	WRKY DNA-binding protein 14	-1.09591	2.318897	4.336206

Potri.001 G208600	WRKY	ATWRKY9,WRKY9	WRKY DNA-binding protein 9	0.94362	1.183855	6.71114
Potri.001 G044500	WRKY	ATWRKY40,WRKY40	WRKY DNA-binding protein 40	3.279493	1.540491	0.887498
Potri.003 G111900	WRKY	WRKY21	WRKY DNA-binding protein 21	2.336812	4.236169	2.39178
Potri.017 G104800	WRKY	AtWRKY35,MEE24,WRKY35	WRKY DNA-binding protein 35	0.701182	0.923685	2.23441
Potri.006 G105300	WRKY	ATWRKY33,WRKY33	WRKY DNA-binding protein 33	2.135106	0.37821	-0.76773
Potri.002 G186600	WRKY	AtWRKY47,WRKY47	WRKY family transcription factor	-2.19763	-1.01287	0.737661
Potri.001 G092900	WRKY	AtWRKY41,WRKY41	WRKY family transcription factor	2.508923	1.897765	0.26935
Potri.001 G352400	WRKY	ATWRKY28,WRKY28	WRKY DNA-binding protein 28	3.064086	0.8167	0.915888
Potri.005 G141400	WRKY	ATWRKY7,WRKY7	WRKY DNA-binding protein 7	1.968544	-0.55531	-2.97628
Potri.006 G072400	WRKY	ATWRKY11,WRKY11	WRKY DNA-binding protein 11	2.3106	0.411148	-0.65117
Potri.001 G058800	WRKY	ATWRKY75,WRKY75	WRKY DNA-binding protein 75	-3.22359	-0.29202	2.198178
Potri.017 G079500	WRKY	ATWRKY72,WRKY72	WRKY DNA-binding protein 72	-0.38877	1.24795	2.539469
Potri.012 G031700	WRKY	ATWRKY30,WRKY30	WRKY DNA-binding protein 30	5.630631	3.23951	2.694392
Potri.001 G099000	WRKY	ATWRKY27,WRKY27	WRKY DNA-binding protein 27	9.488091	3.446696	4.780755

-2 0 2

Supplementary Table S5: Details of Mapman analysis for Leu in Figure 5a. Expression pattern of each gene was presant by contrasting 6hpi_vs_CK, 36hpi_vs_CK, and 96hpi_vs_CK. The measured value of gene expression of genes was log2(fold change). The scale on the top right corner representes the value of log2(fold change).

Gene ID	Classfications in Mapman	*Arabidopsis thaliana*-symbol	*Arabidopsis thaliana*-defline	6 hpi	36 hpi	96 hpi
Potri.005G237900	ABA	0	Abscisic acid-responsive (TB2/DP1, HVA22) family protein	-1.02075	-2.24877	-4.06816
Potri.005G138400	ABA	ABA1,ATABA1,ATZEP,IBS3,LOS6,NPQ2,ZEP	zeaxanthin epoxidase (ZEP) (ABA1)	-0.11898	-0.24226	-3.25054
Potri.011G112400	ABA	ATNCED3,NCED3,SIS7,STO1	nine-cis-epoxycarotenoid dioxygenase 3	1.948463	-0.49601	3.455414
Potri.005G069100	ABA	CCD4,NCED4	nine-cis-epoxycarotenoid dioxygenase 4	-0.54441	-0.25011	-2.92804
Potri.006G206800	ABA	ABA2,ATABA2,ATSDR1,GIN1,ISI4,SDR1,SIS4,SRE1	NAD(P)-binding Rossmann-fold superfamily protein	-2.40375	-1.21648	-4.1094
Potri.006G164600	ABA	ABI1,AtABI1	Protein phosphatase 2C family protein	0.567394	0.726198	3.521931
Potri.004G068000	ABA	0	GRAM domain-containing protein / ABA-responsive protein-related	0.802345	1.976617	2.742941
Potri.001G323300	ABA	0	GRAM domain family protein	1.288984	0.511854	3.957617
Potri.017G108400	ABA	0	Late embryogenesis abundant protein (LEA) family protein	0.379921	-1.18769	-2.61004
Potri.008G106400	Abiotic stress	ALPHA-DOX1,DIOX1,DOX1,PADOX-1	Peroxidase superfamily protein	-0.00037	1.910852	3.449549
Potri.001G420800	Abiotic stress	CHL	chloroplastic lipocalin	-0.5991	-1.99182	-2.41375

Potri.001G102400	Abiotic stress	ATOSM34,OSM34	osmotin 34	5.586793	8.001575	12.84938
Potri.012G133400	Abiotic stress	ATBAG1,BAG1	BCL-2-associated athanogene 1	-0.31105	-0.90551	-2.59947
Potri.007G082500	Abiotic stress	ATATH13,ATH13,ATOSA1,OSA1	ABC2 homolog 13	-0.19601	-0.07812	-2.84064
Potri.004G187400	Abiotic stress	0	HSP20-like chaperones superfamily protein	-2.4821	0.147875	1.130324
Potri.010G195700	Abiotic stress	0	HSP20-like chaperones superfamily protein	-1.86401	2.261195	4.185699
Potri.003G071100	Abiotic stress	0	HSP20-like chaperones superfamily protein	-2.39014	-2.03053	-0.74794
Potri.005G020300	Abiotic stress	0	Chaperone DnaJ-domain superfamily protein	1.144206	1.364878	2.492773
Potri.002G074600	Abiotic stress	0	DNAJ heat shock N-terminal domain-containing protein	-1.25476	-1.99184	-2.7417
Potri.007G135800	Abiotic stress	0	DNAJ heat shock family protein	-2.22173	-1.12445	-1.39624
Potri.008G043500	Abiotic stress	0	Chaperone DnaJ-domain superfamily protein	-0.39376	-2.19853	-1.67299
Potri.010G188200	Abiotic stress	0	Double Clp-N motif-containing P-loop nucleoside triphosphate hydrolases superfamily protein	0.727975	-0.59171	-2.29865
Potri.002G166300	Abiotic stress	ATBAG6,BAG6	BCL-2-associated athanogene 6	-0.39406	-1.21333	3.202199
Potri.016G120000	Abiotic stress	ATERDJ3A,TMS1	DNAJ heat shock N-terminal domain-containing protein	-0.23829	0.617823	2.038462
Potri.001G042600	Abiotic stress	ATHSP70,HSP70	heat shock protein 70	2.062015	3.230078	6.819344
Potri.006G001300	Abiotic stress	0	Chaperone DnaJ-domain superfamily protein	1.395833	-4.67478	-5.9025
Potri.002G198000	Abiotic stress	ATERDJ3B,ERDJ3B	DNAJ heat shock family protein	1.549206	1.457471	2.97603

Potri.002G197300	Abiotic stress	0	Heat shock protein DnaJ with tetratricopeptide repeat	-1.2941	-0.87977	-2.41758
Potri.013G109000	Abiotic stress	0	Chaperone DnaJ-domain superfamily protein	0.178565	0.555519	-3.51007
Potri.006G022100	Abiotic stress	0	heat shock protein 70 (Hsp 70) family protein	1.294398	1.791268	2.484197
Potri.005G241100	Abiotic stress	AtHsp90-7,AtHsp90.7,HSP90.7,SHD	Chaperone protein htpG family protein	0.969055	2.034695	1.976027
Potri.010G053400	Abiotic stress	HSP21	heat shock protein 21	-2.04917	-0.78966	-1.16389
Potri.006G073800	Abiotic stress	0	Double Clp-N motif-containing P-loop nucleoside triphosphate hydrolases superfamily protein	-0.52215	-1.13173	-2.24662
Potri.007G043800	Abiotic stress	AT-HSFB1,ATHSF4,HSF4,HSFB1	heat shock factor 4	0.542855	0.659754	3.245497
Potri.008G054000	Abiotic stress	AT-HSC70-1,HSC70,HSC70-1,HSP70-1	heat shock cognate protein 70-1	0.269083	2.412062	3.444059
Potri.016G088600	Abiotic stress	ATP58IPK,P58IPK	homolog of mamallian P58IPK	0.964662	0.844921	2.232328
Potri.006G223900	Abiotic stress	HSP17.6II	17.6 kDa class II heat shock protein	-2.47023	-0.99864	-1.15888
Potri.011G051600	Abiotic stress	ATHSF3,ATHSFA1B,HSF3,HSFA1B	heat shock factor 3	0.615283	1.449618	4.459966
Potri.007G072600	Abiotic stress	0	DNAJ heat shock N-terminal domain-containing protein	1.983199	0.530005	2.019585
Potri.018G034600	Abiotic stress	0	DNAJ heat shock family protein	1.284432	0.540925	2.701889
Potri.001G087500	Abiotic stress	BIP,BIP2	Heat shock protein 70 (Hsp 70) family protein	2.243436	2.397077	2.711837
Potri.008G101	Abiotic stress	0	Chaperone DnaJ-domain superfamily protein	1.916776	0.350424	-2.20316

800						
Potri.004G073600	Abiotic stress	ATHS83,AtHsp90-1,ATHSP90.1,HSP81-1,HSP81.1,HSP83,HSP90.1	heat shock protein 90.1	-3.2991	-0.71741	1.816314
Potri.001G286700	Abiotic stress	AtHsp90.4,Hsp81.4	HEAT SHOCK PROTEIN 81.4	-0.58471	2.073889	3.278657
Potri.007G122100	Abiotic stress	RCI3,RCI3A	Peroxidase superfamily protein	-0.4115	0.084785	-4.04023
Potri.016G104600	Abiotic stress	0	Adenine nucleotide alpha hydrolases-like superfamily protein	0.450037	0.553967	2.07754
Potri.012G144600	Abiotic stress	0	Calcium-dependent lipid-binding (CaLB domain) family protein	1.074935	0.753414	2.404434
Potri.001G358300	Abiotic stress	ERD4	Early-responsive to dehydration stress protein (ERD4)	-0.36534	-1.49107	-2.64729
Potri.003G102400	Abiotic stress	0	S-adenosyl-L-methionine-dependent methyltransferases superfamily protein	-1.28343	-1.23319	-3.45261
Potri.001G133800	Abiotic stress	0	early-responsive to dehydration stress protein (ERD4)	-1.23763	-0.91981	-2.12259
Potri.002G226800	Abiotic stress	0	ERD (early-responsive to dehydration stress) family protein	-1.04546	-2.9339	-1.42079
Potri.010G012100	Abiotic stress	ATDI21,DI21	drought-induced 21	0.553546	0.02072	2.029961
Potri.004G136000	Abiotic stress	ATRD22,RD22	BURP domain-containing protein	2.885447	6.264934	7.771164
Potri.015G076500	Abiotic stress	SRO5	similar to RCD one 5	0.606823	-0.1539	2.161105
Potri.005G214100	Abiotic stress	ATCLH1,ATHCOR1,CLH1,CORI1	chlorophyllase 1	5.019034	2.298919	5.995235
Potri.010G182	Abiotic stress	ATWI-12,SA	senescence associated gene 20	0.971046	0.047109	2.690528

200		G20,WI12				
Potri.019G116500	Abiotic stress	0	Wound-responsive family protein	-1.02344	1.44286	2.796931
Potri.001G088800	Abiotic stress	0	DCD (Development and Cell Death) domain protein	2.111138	2.099432	3.838856
Potri.002G134300	Abiotic stress	JAC1	J-domain protein required for chloroplast accumulation response 1	0.801392	2.155554	1.340066
Potri.013G000500	Abiotic stress	GLP5	germin-like protein 5	2.019335	2.57366	3.833896
Potri.010G038200	Abiotic stress	GLP7	germin-like protein 7	-0.6682	-0.75928	-3.03114
Potri.015G068200	Abiotic stress	0	RmlC-like cupins superfamily protein	-0.66981	3.194388	3.837107
Potri.004G033000	Abiotic stress	MLP423	MLP-like protein 423	19.06983	15.87217	14.25046
Potri.004G032900	Abiotic stress	MLP423	MLP-like protein 423	19.18403	16.36234	14.88172
Potri.005G177100	Abiotic stress	0	Adenine nucleotide alpha hydrolases-like superfamily protein	-1.77829	-2.93359	-3.1762
Potri.008G121900	Abiotic stress	0	Adenine nucleotide alpha hydrolases-like superfamily protein	-0.08236	-2.17466	-0.76344
Potri.010G140200	Abiotic stress	0	Adenine nucleotide alpha hydrolases-like superfamily protein	-1.11318	-1.86646	-3.53191
Potri.008G131100	Abiotic stress	MLP43	MLP-like protein 43	-0.55192	-9.86131	-10.5206
Potri.001G169000	Abiotic stress	ATGER1,GER1,GLP1	germin-like protein 1	-0.69902	-1.0781	-4.89739
Potri.004G156200	Abiotic stress	RD2	Adenine nucleotide alpha hydrolases-like superfamily protein	0.605649	0.189173	2.137102
Potri.002G205300	Abiotic stress	0	Adenine nucleotide alpha hydrolases-like superfamily protein	1.282342	2.377	4.605626
Potri.014G110400	Abiotic stress	GLP10	germin-like protein 10	0.284749	-1.18275	-2.99412

Potri.004G114300	Abiotic stress	0	Pollen Ole e 1 allergen and extensin family protein	-0.88954	-1.46638	-3.16892
Potri.006G142600	Abiotic stress	ATGER3,GER3,GLP3,GLP3A,GLP3B	germin 3	-3.10837	-2.8842	-6.13567
Potri.004G051500	Abiotic stress	0	Polyketide cyclase/dehydrase and lipid transport superfamily protein	-0.87141	-2.76004	-4.95012
Potri.009G140400	Abiotic stress	0	RmlC-like cupins superfamily protein	0	0	20.64351
Potri.012G111500	Abiotic stress	0	RmlC-like cupins superfamily protein	-1.04214	-0.60215	-2.15454
Potri.008G129400	Auxin	ATPIN3,PIN3	Auxin efflux carrier family protein	-0.07435	-1.10537	-2.32129
Potri.012G047200	Auxin	ATPIN1,PIN1	Auxin efflux carrier family protein	-0.25349	-1.55056	-2.79741
Potri.006G142400	Auxin	0	O-fucosyltransferase family protein	1.173112	1.110988	1.945382
Potri.014G136800	Auxin	0	Auxin-responsive GH3 family protein	1.424206	1.549734	3.345465
Potri.009G141000	Auxin	0	SAUR-like auxin-responsive protein family	-0.72151	0.349579	-4.64412
Potri.009G141200	Auxin	0	SAUR-like auxin-responsive protein family	-0.56069	-2.34121	-5.62757
Potri.009G140900	Auxin	0	SAUR-like auxin-responsive protein family	-0.96245	-2.44669	-4.6953
Potri.009G141100	Auxin	SAUR68	SAUR-like auxin-responsive protein family	-1.3493	-2.04655	-4.88086
Potri.008G158300	Auxin	0	NAD(P)-linked oxidoreductase superfamily protein	-0.47797	-0.67306	-2.70808
Potri.002G234000	Auxin	0	NAD(P)-linked oxidoreductase superfamily protein	-0.16057	0.612924	2.630774
Potri.001G298300	Auxin	GH3.1	Auxin-responsive GH3 family protein	0.692348	-1.96679	2.422107

Potri.010G065200	Auxin	IAA13	auxin-induced protein 13	-0.43189	-1.54739	-2.33417
Potri.008G172400	Auxin	IAA13	auxin-induced protein 13	-0.5079	-0.97437	-2.02059
Potri.002G176400	Auxin	0	SAUR-like auxin-responsive protein family	0.561515	-0.18938	3.021762
Potri.002G206400	Auxin	GH3.9	putative indole-3-acetic acid-amido synthetase GH3.9	-0.72597	-0.33717	-3.31379
Potri.005G096400	Auxin	0	SAUR-like auxin-responsive protein family	1.104015	2.429196	3.807369
Potri.002G145300	Auxin	0	SAUR-like auxin-responsive protein family	4.030228	2.967985	7.463472
Potri.014G098700	Auxin	0	Cytochrome b561/ferric reductase transmembrane with DOMON related domain	-0.92421	0.074062	-4.62308
Potri.013G144300	Auxin	DFL2,GH3-10	Auxin-responsive GH3 family protein	-0.92067	-2.19316	-4.90382
Potri.001G119900	Auxin	0	SAUR-like auxin-responsive protein family	3.936746	2.286238	5.418701
Potri.003G113100	Auxin	0	SAUR-like auxin-responsive protein family	3.113302	1.137855	5.402174
Potri.004G040600	Auxin	0	Aluminium induced protein with YGL and LRDR motifs	0.184581	-1.16354	2.59528
Potri.004G164400	Auxin	0	SAUR-like auxin-responsive protein family	-3.04664	-3.06493	-5.67964
Potri.004G165300	Auxin	0	SAUR-like auxin-responsive protein family	-2.13226	-2.64915	-3.72353
Potri.004G164300	Auxin	0	SAUR-like auxin-responsive protein family	-0.36921	-1.67612	-4.44247
Potri.004G165400	Auxin	0	SAUR-like auxin-responsive protein family	-1.60887	-3.16341	-3.33691
Potri.009G126300	Auxin	0	SAUR-like auxin-responsive protein family	-1.58636	-1.34922	-3.04068
Potri.017G043	Auxin	0	SAUR-like auxin-responsive protein family	-0.83738	-2.56693	-3.13801

400						
Potri.002G222700	Auxin	0	Auxin-responsive family protein	4.475561	3.928045	7.494545
Potri.001G060400	Auxin	0	SAUR-like auxin-responsive protein family	-12.0221	3.904347	7.166432
Potri.011G129700	Auxin	DFL1,GH3.6	Auxin-responsive GH3 family protein	-0.92723	-0.66948	2.51765
Potri.012G065700	Beta glucanase	PDCB3	plasmodesmata callose-binding protein 3	-0.59844	-0.38112	-2.24822
Potri.001G380600	Beta glucanase	0	Carbohydrate-binding X8 domain superfamily protein	-1.23897	-3.53405	-4.39436
Potri.013G119500	Beta glucanase	PDCB5	plasmodesmata callose-binding protein 5	-0.44537	-0.32731	-2.31544
Potri.011G152400	Beta glucanase	0	Glycosyl hydrolase superfamily protein	0.090362	-0.01462	-2.34628
Potri.004G086400	Beta glucanase	0	O-Glycosyl hydrolases family 17 protein	-1.05793	-1.46831	-2.70513
Potri.002G224600	Beta glucanase	0	O-Glycosyl hydrolases family 17 protein	-0.72919	-1.63489	-3.88687
Potri.014G183800	Beta glucanase	0	O-Glycosyl hydrolases family 17 protein	0.449391	1.552232	2.329733
Potri.009G163700	Beta glucanase	0	Glycosyl hydrolase superfamily protein	3.184486	0.822427	4.861825
Potri.008G055900	Beta glucanase	0	O-Glycosyl hydrolases family 17 protein	-0.38686	-2.55059	-0.57518
Potri.006G046100	Beta glucanase	BG1	beta-1,3-glucanase 1	4.464935	2.930046	6.289645
Potri.001G255100	Beta glucanase	0	Glycosyl hydrolase superfamily protein	1.695531	2.275363	9.597705
Potri.002G007300	Beta glucanase	0	O-Glycosyl hydrolases family 17 protein	-2.20854	-2.09719	-3.9144
Potri.018G068600	Beta glucanase	0	O-Glycosyl hydrolases family 17 protein	-1.16987	-1.2101	-2.20378

Potri.008G012800	Brassinost.	0	3-oxo-5-alpha-steroid 4-dehydrogenase family protein	-0.65713	0.048715	-2.90624
Potri.005G124000	Brassinost.	CLM,CYP90B1,DWF4,PSC1,SAV1,SNP2	Cytochrome P450 superfamily protein	-0.41751	-1.28931	-2.18804
Potri.010G189800	Brassinost.	CBB3,CPD,CYP90,CYP90A,CYP90A1,DWF3	Cytochrome P450 superfamily protein	-0.62496	-1.87534	-2.60448
Potri.006G154500	Brassinost.	BAS1,CYP72B1,CYP734A1	Cytochrome P450 superfamily protein	-1.43457	-3.02751	-0.22971
Potri.001G200100	Brassinost.	CYP90D1	cytochrome P450, family 90, subfamily D, polypeptide 1	-0.25387	-0.61669	-3.0284
Potri.007G018400	Brassinost.	ROT3	Cytochrome P450 superfamily protein	-0.43318	-1.27455	-2.99889
Potri.001G263700	Brassinost.	CPH,SMT1	sterol methyltransferase 1	-0.18035	-2.45135	-3.60253
Potri.002G114500	Brassinost.	SQE1,XF1	FAD/NAD(P)-binding oxidoreductase family protein	2.549049	2.018167	3.785308
Potri.018G148800	Brassinost.	CAS1	cycloartenol synthase 1	-0.67851	-0.3523	-2.22148
Potri.005G146700	Brassinost.	SQE2	squalene epoxidase 2	-0.33639	-1.30226	1.949167
Potri.012G067600	Brassinost.	0	Leucine-rich repeat protein kinase family protein	6.435109	6.368382	9.051744
Potri.006G104300	Brassinost.	IMK2	inflorescence meristem receptor-like kinase 2	-1.07419	-2.89382	-4.12285
Potri.005G086500	Brassinost.	ATBRI1,BIN1,BRI1,CBB2,DWF2	Leucine-rich receptor-like protein kinase family protein	-0.65023	-0.90458	-2.69537
Potri.004G206600	Brassinost.	EXL5	EXORDIUM like 5	-1.17582	-1.19047	-3.65792

Potri.006G183200	Brassinost.	BRS1,SCPL24	alpha/beta-Hydrolases superfamily protein	-2.01072	-2.15644	-2.27898
Potri.001G450700	Brassinost.	BRH1	brassinosteroid-responsive RING-H2	0.758327	0.635563	3.020978
Potri.005G108400	B-ZIP	0	Protein of unknown function (DUF630 and DUF632)	2.225286	1.300284	3.756951
Potri.005G257900	B-ZIP	ATBZIP60,BZIP60	basic region/leucine zipper motif 60	0.549053	0.860517	2.413908
Potri.008G111200	B-ZIP	0	ATPase E1-E2 type family protein / haloacid dehalogenase-like hydrolase family protein	2.276112	1.347269	3.446424
Potri.002G031900	B-ZIP	AtbZIP44,bZIP44	basic leucine-zipper 44	0.290004	0.92749	2.062307
Potri.002G090700	B-ZIP	0	bZIP transcription factor family protein	1.631017	0.873329	3.039587
Potri.010G004200	B-ZIP	HYH	HY5-homolog	1.427216	2.478015	-0.12911
Potri.001G374200	B-ZIP	ATBZIP61,BZIP61	Basic-leucine zipper (bZIP) transcription factor family protein	-1.32211	-3.37499	-3.97435
Potri.002G196200	B-ZIP	ATBZIP53,BZIP53	basic region/leucine zipper motif 53	0.212084	0.004689	1.967646
Potri.006G058800	B-ZIP	bZIP65,TGA10	bZIP transcription factor family protein	-0.67501	-1.27367	7.176062
Potri.006G251800	B-ZIP	HY5,TED 5	Basic-leucine zipper (bZIP) transcription factor family protein	1.372714	2.174175	3.04486
Potri.010G142900	B-ZIP	AtbZIP1,bZIP1	basic leucine-zipper 1	-0.01442	1.018675	1.993405
Potri.007G085700	B-ZIP	TGA1	bZIP transcription factor family protein	-0.45214	0.21262	4.475555
Potri.005G082000	B-ZIP	TGA1	bZIP transcription factor family protein	0.376064	1.547446	4.567273
Potri.003G123700	Cell wall	ATUGE1,UGE1	UDP-D-glucose/UDP-D-galactose 4-epimerase 1	-0.56812	-2.97124	-0.93227
Potri.017G092	Cell wall	0	UDP-glucose 6-dehydrogenase family protein	0.959567	0.830348	2.051052

000						
Potri.008G053100	Cell wall	UXS6	UDP-XYL synthase 6	0.037137	0.639327	5.074876
Potri.001G237200	Cell wall	UXS5	UDP-XYL synthase 5	-0.63182	-0.07587	-4.21489
Potri.002G204400	Cell wall	ATUXS2,AUD1,UXS2	NAD(P)-binding Rossmann-fold superfamily protein	0.691878	1.409506	2.950497
Potri.012G128200	Cell wall	GAE2	UDP-D-glucuronate 4-epimerase 2	-0.30124	-1.29742	-3.0909
Potri.001G320000	Cell wall	GAE6	UDP-D-glucuronate 4-epimerase 6	-0.05531	-0.35046	-2.0758
Potri.005G116200	Cell wall	GMD2,MUR1,MUR_1	NAD(P)-binding Rossmann-fold superfamily protein	1.659315	0.760493	2.789039
Potri.006G022000	Cell wall	HSR8,MUR4,UXE1	NAD(P)-binding Rossmann-fold superfamily protein	1.511484	1.777704	3.339986
Potri.011G156100	Cell wall	HSR8,MUR4,UXE1	NAD(P)-binding Rossmann-fold superfamily protein	1.930042	0.63577	2.675925
Potri.011G103700	Cell wall	ATRHM1,RHM1,ROL1	rhamnose biosynthesis 1	0.704836	1.086803	2.024652
Potri.001G343400	Cell wall	ATGLCAK,GLCAK	glucuronokinase G	0.570618	0.833977	3.379334
Potri.018G009300	Cell wall	ATCSLC05,ATCSLC5,CSLC05,CSLC5	Cellulose-synthase-like C5	-0.64202	-1.37377	-3.14485
Potri.006G116900	Cell wall	ATCSLA09,ATCSLA9,CSLA09,CSLA9,RAT4	Nucleotide-diphospho-sugar transferases superfamily protein	-0.67775	-2.86594	-4.58843
Potri.009G149700	Cell wall	ATCSLA02,ATCSLA2,CSLA02,CSLA2	cellulose synthase-like A02	-0.8552	-1.6375	-2.29633
Potri.002G200300	Cell wall	ATCSLD5,CSLD5,SOS6	cellulose synthase-like D5	-1.96763	-2.91755	-3.65884

Potri.006G004300	Cell wall	ATCSLE1,CSLE1	cellulose synthase like E1	1.053995	4.168661	2.958619
Potri.001G136200	Cell wall	ATCSLD3,CSLD3,KJK	cellulose synthase-like D3	0.058664	0.370822	2.530247
Potri.004G059600	Cell wall	ATCESA8,CESA8,IRX1,LEW2	cellulose synthase family protein	-0.91898	0.480891	-4.78988
Potri.006G181900	Cell wall	ATCESA7,CESA7,IRX3,MUR10	Cellulose synthase family protein	-0.85684	-0.84538	-5.33582
Potri.002G257900	Cell wall	CESA4,IRX5,NWS2	cellulose synthase A4	-0.28558	0.679006	-2.77536
Potri.011G135200	Cell wall	COBL10	COBRA-like protein 10 precursor	-2.60158	-0.42146	-0.91136
Potri.001G033800	Cell wall	ATFT1,ATFUT1,FT1,MUR2	fucosyltransferase 1	5.81626	3.468599	6.566928
Potri.002G132900	Cell wall	ATGATL1,GATL1,GLZ1,PARVUS	Nucleotide-diphospho-sugar transferases superfamily protein	0.096922	-0.99298	-3.23539
Potri.016G086400	Cell wall	IRX9	Nucleotide-diphospho-sugar transferases superfamily protein	-0.46797	0.203443	-4.31277
Potri.005G061600	Cell wall	GUX1,PGSIP1	plant glycogenin-like starch initiation protein 1	-0.01924	0.026218	-3.78062
Potri.014G029900	Cell wall	GUX2,PGSIP3	plant glycogenin-like starch initiation protein 3	-0.51989	0.230391	-3.1289
Potri.001G416800	Cell wall	GAUT12,IRX8,LGT6	galacturonosyltransferase 12	-0.83354	-0.69162	-5.26289
Potri.013G144200	Cell wall	0	cell wall protein precursor, putative	-1.72609	-3.85842	-6.57833
Potri.002G223300	Cell wall	FLA7	FASCICLIN-like arabinoogalactan 7	-1.23041	-1.63169	-4.27862
Potri.013G120600	Cell wall	FLA6	FASCICLIN-like arabinogalactan 6	-0.22452	-2.72411	-18.1976

Potri.014G135100	Cell wall	AGP26,ATAGP26	arabinogalactan protein 26	-0.61786	0.018741	-2.42839
Potri.006G276200	Cell wall	FLA14	FASCICLIN-like arabinogalactan protein 14 precursor	-2.58522	-2.48126	-1.87803
Potri.014G071700	Cell wall	FLA10	FASCICLIN-like arabinogalactan-protein 10	-0.30785	-0.53441	-2.53681
Potri.001G094700	Cell wall	AGP20,AtAGP20	arabinogalactan protein 20	-0.56359	-1.64202	-2.47724
Potri.014G168100	Cell wall	FLA2	FASCICLIN-like arabinogalactan 2	-1.4705	-3.96038	-5.1691
Potri.005G144900	Cell wall	AGP18,ATAGP18	arabinogalactan protein 18	-0.0135	-1.2148	-3.23488
Potri.001G320800	Cell wall	ATFLA11,FLA11	FASCICLIN-like arabinogalactan-protein 11	-0.57321	-0.32429	-5.88212
Potri.006G200300	Cell wall	FLA17	FASCICLIN-like arabinogalactan protein 17 precursor	0.196883	-0.77144	-2.44867
Potri.001G367900	Cell wall	FLA1	FASCICLIN-like arabinogalactan 1	-0.09419	-1.68595	-2.43757
Potri.011G093500	Cell wall	FLA1	FASCICLIN-like arabinogalactan 1	-0.40284	-1.64647	-2.5154
Potri.003G220900	Cell wall	AGP14,ATAGP14	arabinogalactan protein 14	0.158277	-0.19082	-3.03198
Potri.017G050200	Cell wall	AGP1,ATAGP1	arabinogalactan protein 1	1.081826	1.078568	4.089593
Potri.004G168600	Cell wall	ATPRP4,PRP4	proline-rich protein 4	-1.29173	-2.14476	-4.66179
Potri.010G083100	Cell wall	0	Leucine-rich repeat (LRR) family protein	-0.56707	-0.0506	-2.8559
Potri.006G245600	Cell wall	0	Leucine-rich repeat (LRR) family protein	-0.68224	-0.85713	-2.05597
Potri.002G070100	Cell wall	ATEXT3,EXT3,RSH	extensin 3	-2.67983	-2.26594	-1.34534
Potri.005G190	Cell wall	ATEXT1,ATE	extensin 4	4.562785	5.209459	7.869787

100		XT4,EXT1,EXT4,ORG5				
Potri.004G117800	Cell wall	ATRGP2,RGP2	reversibly glycosylated polypeptide 2	2.680063	2.549703	4.171971
Potri.006G062200	Cell wall	AtGUS3,GUS3	glucuronidase 3	4.775909	4.850043	6.886336
Potri.002G023900	Cell wall	AtGH9B5,GH9B5	glycosyl hydrolase 9B5	-1.08289	-1.91302	-2.96168
Potri.002G197200	Cell wall	ATBXL2,BXL2	beta-xylosidase 2	-3.03029	-1.15105	-5.16021
Potri.002G094000	Cell wall	0	Glycosyl hydrolase family protein	0.156691	-1.06	-3.16106
Potri.010G141400	Cell wall	ATBXL1,BXL1	beta-xylosidase 1	-0.97681	-0.07351	4.26325
Potri.005G120500	Cell wall	0	Glycosyl hydrolase superfamily protein	-1.61178	-1.23292	-3.2874
Potri.002G110100	Cell wall	0	Rhamnogalacturonate lyase family protein	-0.80538	0.314323	-2.74243
Potri.011G159000	Cell wall	0	Pectin lyase-like superfamily protein	-0.62528	-0.10043	-2.38837
Potri.008G100500	Cell wall	0	Pectin lyase-like superfamily protein	0.717247	-1.15423	-17.3706
Potri.008G189200	Cell wall	0	Pectin lyase-like superfamily protein	-1.37073	-3.07686	-5.38901
Potri.008G182200	Cell wall	0	Pectate lyase family protein	-0.44773	-3.65015	-5.51995
Potri.008G190000	Cell wall	PG2	polygalacturonase 2	-0.55339	-0.95735	-3.72007
Potri.009G060400	Cell wall	QRT2	Pectin lyase-like superfamily protein	-10.9469	-10.9469	7.501473
Potri.010G008600	Cell wall	0	Pectin lyase-like superfamily protein	0.116705	-1.1168	-2.82309
Potri.001G339	Cell wall	0	Pectin lyase-like superfamily protein	-3.53138	-0.15791	4.124281

500						
Potri.006G122000	Cell wall	0	Pectin lyase-like superfamily protein	-2.17513	-2.64171	-2.66869
Potri.003G131700	Cell wall	0	Pectin lyase-like superfamily protein	-1.39179	-0.44427	-2.66482
Potri.001G052300	Cell wall	0	Pectin lyase-like superfamily protein	-2.3474	-4.6961	-6.59547
Potri.003G139100	Cell wall	0	Pectin lyase-like superfamily protein	-1.4386	-2.65148	-3.81083
Potri.002G110300	Cell wall	0	Rhamnogalacturonate lyase family protein	-1.08978	-0.8117	-3.16143
Potri.007G105800	Cell wall	0	Pectin lyase-like superfamily protein	0.861623	0.086432	4.081325
Potri.008G032700	Cell wall	0	Pectin lyase-like superfamily protein	0.780513	-1.68438	2.564285
Potri.006G058600	Cell wall	ATPGIP1,PGIP1	polygalacturonase inhibiting protein 1	3.768846	1.642597	6.462725
Potri.015G087800	Cell wall	0	Pectin lyase-like superfamily protein	-0.74041	-2.6347	-3.9127
Potri.004G021000	Cell wall	XTH8	xyloglucan endotransglucosylase/hydrolase 8	-0.99213	-1.83778	-2.96105
Potri.003G097300	Cell wall	XTH30,XTR4	xyloglucan endotransglucosylase/hydrolase 30	0.508717	0.749786	2.509546
Potri.014G066300	Cell wall	ATEXPB2,ATHEXP BETA 1.4,EXPB2	expansin B2	-1.33403	0.17306	5.133307
Potri.004G123200	Cell wall	AT-EXP1,ATEXP1,ATEXPA1,ATHEXP ALPHA 1.2,EXP1,EXPA1	expansin A1	-0.79964	-7.66891	-19.8533
Potri.013G060	Cell wall	ATEXP15,ATE	expansin A15	-1.01993	-0.89686	-2.14401

800		XPA15,ATHEXP ALPHA 1.3,EXP15,EXPA15				
Potri.006G155000	Cell wall	PNP-A	plant natriuretic peptide A	3.594194	4.219048	7.412695
Potri.009G006600	Cell wall	XTH32	xyloglucan endotransglucosylase/hydrolase 32	3.825115	2.625444	0.647023
Potri.006G086100	Cell wall	ATEXP3,ATEXPA3,ATHEXP ALPHA 1.9,EXP3	Barwin-like endoglucanases superfamily protein	-0.65899	-1.68429	-3.59879
Potri.001G240900	Cell wall	ATEXP4,ATEXPA4,ATHEXP ALPHA 1.6,EXPA4	expansin A4	-0.91208	-2.33089	-1.82219
Potri.013G154700	Cell wall	ATEXP8,ATEXPA8,ATHEXP ALPHA 1.11,EXP8,EXPA8	expansin A8	-0.96548	-2.42155	-4.39471
Potri.014G146100	Cell wall	XTH16	xyloglucan endotransglucosylase/hydrolase 16	-0.19227	-1.22851	-2.60388
Potri.013G152400	Cell wall	XTH9	xyloglucan endotransglucosylase/hydrolase 9	-0.34955	-2.18823	-3.42957
Potri.002G060500	Cell wall	XTH15,XTR7	xyloglucan endotransglucosylase/hydrolase 15	4.087783	2.719468	5.954843
Potri.003G083200	Cell wall	AT-EXPR,ATEXLB1,ATEXPR1,ATHEXP BETA 3.1,EXLB1,EXPR	expansin-like B1	2.408803	1.853596	6.896547

Potri.006G071200	Cell wall	XTH23,XTR6	xyloglucan endotransglycosylase 6	-0.7445	-1.29889	-2.61701
Potri.019G101900	Cell wall	ATEXPB3,ATHEXP BETA 1.6,EXPB3	expansin B3	-1.12366	-2.55134	-3.0952
Potri.018G031900	Cell wall	0	Barwin-related endoglucanase	0.613228	0.212461	2.733501
Potri.001G071000	Cell wall	EXGT-A4,XTH5	xyloglucan endotransglucosylase/hydrolase 5	-0.58318	-3.02475	-4.44564
Potri.007G008500	Cell wall	XTH6	xyloglucan endotransglucosylase/hydrolase 6	-0.76084	-1.92599	-3.91263
Potri.002G202600	Cell wall	0	Plant invertase/pectin methylesterase inhibitor superfamily	8.661923	6.13705	7.865714
Potri.011G025400	Cell wall	ATPMEPCRA,PMEPCRA	methylesterase PCR A	-1.79675	0.631787	-3.11716
Potri.012G014500	Cell wall	0	Plant invertase/pectin methylesterase inhibitor superfamily	0.088247	-1.6013	-2.78575
Potri.006G134500	Cell wall	ATPME44,PME44	pectin methylesterase 44	0.814396	-1.32373	-2.30408
Potri.007G107300	Cell wall	0	Plant invertase/pectin methylesterase inhibitor superfamily	-1.00074	0.13906	-2.2157
Potri.014G117100	Cell wall	0	Pectin lyase-like superfamily protein	-1.9893	-2.6527	-5.68809
Potri.003G076900	Cell wall	0	Pectin lyase-like superfamily protein	-0.32969	-2.42927	-2.54504
Potri.003G046200	Cell wall	0	Pectinacetylesterase family protein	-0.15492	-1.17813	-2.61752
Potri.010G004400	Cell wall	0	Pectinacetylesterase family protein	-0.02096	-2.86195	-3.077
Potri.010G109400	Cell wall	0	Plant invertase/pectin methylesterase inhibitor superfamily	0.988273	-1.33122	-5.59796
Potri.002G145500	Cell wall	0	Plant invertase/pectin methylesterase inhibitor superfamily	7.232153	1.929622	5.248796

Potri.014G149700	Cell wall	PME61	pectin methylesterase 61	1.367322	5.207264	1.748238
Potri.002G070700	DOF	0	Dof-type zinc finger DNA-binding family protein	-0.32891	-0.76192	-2.9589
Potri.004G056900	DOF	0	Dof-type zinc finger DNA-binding family protein	0.122987	-1.04728	3.001515
Potri.002G129600	DOF	0	Dof-type zinc finger DNA-binding family protein	1.428876	2.524091	2.947619
Potri.013G066700	DOF	CDF3	cycling DOF factor 3	1.07455	0.160393	2.119824
Potri.004G121800	DOF	CDF2	cycling DOF factor 2	0.191223	-0.19258	-3.44325
Potri.008G087800	DOF	CDF2	cycling DOF factor 2	0.054209	-0.75263	-2.76892
Potri.011G047500	DOF	OBP4	OBF binding protein 4	-0.14831	-0.737	-2.99268
Potri.014G099900	ERF	0	Integrase-type DNA-binding superfamily protein	0.557323	-0.61675	-2.29299
Potri.006G138800	ERF	0	Integrase-type DNA-binding superfamily protein	4.888392	-1.73094	2.01405
Potri.001G163700	ERF	RAP2.12	related to AP2 12	1.011207	0.727318	2.118465
Potri.003G139300	ERF	0	Integrase-type DNA-binding superfamily protein	18.21119	16.55351	13.62656
Potri.013G135600	ERF	0	Integrase-type DNA-binding superfamily protein	-10.3762	2.973021	3.069728
Potri.002G065600	ERF	0	Integrase-type DNA-binding superfamily protein	-0.10975	-0.23717	2.34343
Potri.016G018600	ERF	HRD	Integrase-type DNA-binding superfamily protein	-2.3665	-1.15034	2.032808
Potri.006G104200	ERF	AtERF48,DREB2C	Integrase-type DNA-binding superfamily protein	1.14077	2.743366	5.033005
Potri.001G155	ERF	0	Integrase-type DNA-binding superfamily protein	-0.42621	-2.04751	-3.81952

700						
Potri.017G055 400	ERF	0	Integrase-type DNA-binding superfamily protein	0.398232	0.317714	2.773931
Potri.013G158 500	ERF	CRF4	cytokinin response factor 4	-0.22232	2.157129	2.510489
Potri.001G067 600	ERF	Rap2.6L	related to AP2 6l	-0.77019	-0.52804	3.457194
Potri.018G038 100	ERF	0	Integrase-type DNA-binding superfamily protein	0.754337	0.133648	4.456519
Potri.018G028 000	ERF	SHN2	Integrase-type DNA-binding superfamily protein	-0.15833	0.113813	-2.42153
Potri.012G108 500	ERF	0	Integrase-type DNA-binding superfamily protein	2.986575	2.105368	3.866273
Potri.005G195 000	ERF	ABR1	Integrase-type DNA-binding superfamily protein	3.459886	0.229295	6.452082
Potri.010G107 500	Ethylene	0	2-oxoglutarate (2OG) and Fe(II)-dependent oxygenase superfamily protein	1.538178	1.619807	3.370735
Potri.009G099 200	Ethylene	CDCP1,LEJ1	Cystathionine beta-synthase (CBS) family protein	3.509333	3.291459	6.215693
Potri.018G121 700	Ethylene	0	2-oxoglutarate (2OG) and Fe(II)-dependent oxygenase superfamily protein	1.184273	1.962586	3.841198
Potri.002G163 700	Ethylene	ACS1,AT-AC S1	ACC synthase 1	14.56055	11.82402	21.13717
Potri.001G099 400	Ethylene	ACS6,ATACS 6	1-aminocyclopropane-1-carboxylic acid (acc) synthase 6	2.658372	2.379664	4.653013
Potri.002G113 900	Ethylene	ACS8	1-amino-cyclopropane-1-carboxylate synthase 8	0	0	17.37251
Potri.011G020 900	Ethylene	ACO4,EAT1,E FE	ethylene-forming enzyme	0.421632	1.86956	2.619968
Potri.006G151 600	Ethylene	ACO1,ATAC O1	ACC oxidase 1	4.84944	2.514122	3.389168
Potri.006G069 400	Ethylene	SHN1,WIN1	Integrase-type DNA-binding superfamily protein	-2.55483	0.421886	-5.28923

Potri.011G056900	Ethylene	ATERF12,ERF12	ERF domain protein 12	2.10801	0.997863	5.306901
Potri.010G072400	Ethylene	0	Integrase-type DNA-binding superfamily protein	15.02556	12.86016	18.64027
Potri.008G166200	Ethylene	ATERF1,ERF1	ethylene response factor 1	2.354332	2.748921	6.356165
Potri.003G080600	Ethylene	ATERF6,ERF-6-6,ERF6	ethylene responsive element binding factor 6	8.395156	4.272249	5.577546
Potri.001G079900	Ethylene	ATERF-1,ERF-1	ethylene responsive element binding factor 1	-1.24911	-1.1185	-2.62247
Potri.004G051800	Ethylene	0	Integrase-type DNA-binding superfamily protein	-1.06061	0.709036	1.986893
Potri.001G017600	Ethylene	0	0	-0.1405	-1.25032	-3.86474
Potri.001G079600	Ethylene	0	Integrase-type DNA-binding superfamily protein	-0.55803	-1.76985	-3.87776
Potri.001G048200	Ethylene	0	Integrase-type DNA-binding superfamily protein	-0.0394	-2.04854	-3.12107
Potri.002G043300	Ethylene	ATERF-9,ATERF9,ERF9	erf domain protein 9	0.406305	3.658974	5.252415
Potri.001G154200	Ethylene	ATERF-5,ATERF5,ERF5	ethylene responsive element binding factor 5	3.130818	2.538216	2.250801
Potri.019G112000	Ethylene	0	basic helix-loop-helix (bHLH) DNA-binding superfamily protein	0.825814	1.407691	2.754945
Potri.010G208600	Ethylene	0	basic helix-loop-helix (bHLH) DNA-binding superfamily protein	2.283296	0.893577	4.89934
Potri.008G114600	Ethylene	IDA	Putative membrane lipoprotein	3.031939	1.505371	4.750396
Potri.013G009800	Ethylene	0	Adenine nucleotide alpha hydrolases-like superfamily protein	0.261924	-2.20609	-2.05452
Potri.T035400	Glutathione-S-transferase	ATGST1,ATGSTF3,ATGSTF6,ERD11,GST	glutathione S-transferase 6	0.543924	1.698039	2.312713

		1,GSTF6				
Potri.001G458700	Glutathione-S-transferase	0	Peroxidase superfamily protein	18.23772	12.76814	15.66562
Potri.T160000	Glutathione-S-transferase	0	Peroxidase superfamily protein	5.95891	0.731241	2.904306
Potri.011G113400	Glutathione-S-transferase	ATGSTU24,GST,GSTU24	glutathione S-transferase TAU 24	3.532016	5.010725	8.266184
Potri.001G431600	Glutathione-S-transferase	ATGSTU25,GSTU25	glutathione S-transferase TAU 25	-2.09152	-3.53828	-0.76899
Potri.004G078100	Glutathione-S-transferase	0	microsomal glutathione s-transferase, putative	-0.93554	1.650334	2.214173
Potri.004G196700	Glutathione-S-transferase	ATGSTU10,GSTU10	glutathione S-transferase TAU 10	-0.31374	1.984681	2.920456
Potri.001G436600	Glutathione-S-transferase	ATGSTU19,GST8,GSTU19	glutathione S-transferase TAU 19	0.554114	0.371633	2.006785
Potri.002G254000	Glutathione-S-transferase	ATGSTU7,GST25,GSTU7	glutathione S-transferase tau 7	1.544209	3.802614	5.792666
Potri.015G042000	Glutathione-S-transferase	ATGSTU4,GST22,GSTU4	glutathione S-transferase tau 4	2.760144	2.434617	3.729668
Potri.002G015100	Glutathione-S-transferase	ATGSTF11,ATGSTF6,GSTF11	glutathione S-transferase F11	-0.98439	-3.03057	-6.39744
Potri.006G024200	Glutathione-S-transferase	ATGSTU8,GSTU8	glutathione S-transferase TAU 8	3.97842	2.057036	7.159201
Potri.015G121600	Glutathione-S-transferase	0	Glutathione S-transferase family protein	0.426063	0.735879	3.279666
Potri.004G187400	Heat shock proteins	0	HSP20-like chaperones superfamily protein	-2.4821	0.147875	1.130324
Potri.010G195700	Heat shock proteins	0	HSP20-like chaperones superfamily protein	-1.86401	2.261195	4.185699
Potri.003G071100	Heat shock proteins	0	HSP20-like chaperones superfamily protein	-2.39014	-2.03053	-0.74794
Potri.005G020	Heat shock proteins	0	Chaperone DnaJ-domain superfamily protein	1.144206	1.364878	2.492773

300						
Potri.002G074600	Heat shock proteins	0	DNAJ heat shock N-terminal domain-containing protein	-1.25476	-1.99184	-2.7417
Potri.007G135800	Heat shock proteins	0	DNAJ heat shock family protein	-2.22173	-1.12445	-1.39624
Potri.008G043500	Heat shock proteins	0	Chaperone DnaJ-domain superfamily protein	-0.39376	-2.19853	-1.67299
Potri.010G188200	Heat shock proteins	0	Double Clp-N motif-containing P-loop nucleoside triphosphate hydrolases superfamily protein	0.727975	-0.59171	-2.29865
Potri.002G166300	Heat shock proteins	ATBAG6,BAG6	BCL-2-associated athanogene 6	-0.39406	-1.21333	3.202199
Potri.016G120000	Heat shock proteins	ATERDJ3A,TMS1	DNAJ heat shock N-terminal domain-containing protein	-0.23829	0.617823	2.038462
Potri.001G042600	Heat shock proteins	ATHSP70,HSP70	heat shock protein 70	2.062015	3.230078	6.819344
Potri.006G001300	Heat shock proteins	0	Chaperone DnaJ-domain superfamily protein	1.395833	-4.67478	-5.9025
Potri.002G198000	Heat shock proteins	ATERDJ3B,ERDJ3B	DNAJ heat shock family protein	1.549206	1.457471	2.97603
Potri.002G197300	Heat shock proteins	0	Heat shock protein DnaJ with tetratricopeptide repeat	-1.2941	-0.87977	-2.41758
Potri.013G109000	Heat shock proteins	0	Chaperone DnaJ-domain superfamily protein	0.178565	0.555519	-3.51007
Potri.006G022100	Heat shock proteins	0	heat shock protein 70 (Hsp 70) family protein	1.294398	1.791268	2.484197
Potri.005G241100	Heat shock proteins	AtHsp90-7,AtHsp90.7,HSP90.7,SHD	Chaperone protein htpG family protein	0.969055	2.034695	1.976027
Potri.010G053400	Heat shock proteins	HSP21	heat shock protein 21	-2.04917	-0.78966	-1.16389
Potri.006G073800	Heat shock proteins	0	Double Clp-N motif-containing P-loop nucleoside triphosphate hydrolases superfamily protein	-0.52215	-1.13173	-2.24662
Potri.007G043	Heat shock proteins	AT-HSFB1,A	heat shock factor 4	0.542855	0.659754	3.245497

800		THSF4,HSF4,HSFB1				
Potri.008G054000	Heat shock proteins	AT-HSC70-1,HSC70,HSC70-1,HSP70-1	heat shock cognate protein 70-1	0.269083	2.412062	3.444059
Potri.016G088600	Heat shock proteins	ATP58IPK,P58IPK	homolog of mamallian P58IPK	0.964662	0.844921	2.232328
Potri.006G223900	Heat shock proteins	HSP17.6II	17.6 kDa class II heat shock protein	-2.47023	-0.99864	-1.15888
Potri.011G051600	Heat shock proteins	ATHSF3,ATHSFA1B,HSF3,HSFA1B	heat shock factor 3	0.615283	1.449618	4.459966
Potri.007G072600	Heat shock proteins	0	DNAJ heat shock N-terminal domain-containing protein	1.983199	0.530005	2.019585
Potri.018G034600	Heat shock proteins	0	DNAJ heat shock family protein	1.284432	0.540925	2.701889
Potri.001G087500	Heat shock proteins	BIP,BIP2	Heat shock protein 70 (Hsp 70) family protein	2.243436	2.397077	2.711837
Potri.008G101800	Heat shock proteins	0	Chaperone DnaJ-domain superfamily protein	1.916776	0.350424	-2.20316
Potri.004G073600	Heat shock proteins	ATHS83,AtHsp90-1,ATHSP90.1,HSP81-1,HSP81.1,HSP83,HSP90.1	heat shock protein 90.1	-3.2991	-0.71741	1.816314
Potri.001G286700	Heat shock proteins	AtHsp90.4,Hsp81.4	HEAT SHOCK PROTEIN 81.4	-0.58471	2.073889	3.278657
Potri.009G099200	JA	CDCP1,LEJ1	Cystathionine beta-synthase (CBS) family protein	3.509333	3.291459	6.215693
Potri.001G167700	JA	LOX3	lipoxygenase 3	2.27586	2.249656	4.696287
Potri.005G032400	JA	ATLOX1,LOX1	lipoxygenase 1	-0.11632	-0.22566	3.022469

Potri.001G015300	JA	ATLOX2,LOX2	lipoxygenase 2	1.121098	-0.43886	-2.39443
Potri.002G130700	JA	AOS,CYP74A,DDE2	allene oxide synthase	2.593448	2.057868	4.664273
Potri.013G102700	JA	ATOPR2,OPR2	12-oxophytodienoate reductase 2	0.768612	-1.76037	3.815046
Potri.018G065600	JA	DDE1,OPR3	oxophytodienoate-reductase 3	3.420312	-0.85126	4.303513
Potri.012G044900	JA	JAI3,JAZ3,TIFY6B	jasmonate-zim-domain protein 3	0.653428	2.980454	0.77604
Potri.014G155000	MAPK	MAPKKK18	mitogen-activated protein kinase kinase kinase 18	1.403221	0.07734	2.541587
Potri.015G030700	MAPK	ATMKK9,MKK9	MAP kinase kinase 9	0.414795	1.096224	2.672163
Potri.008G130000	MAPK	ATMPK15,MPK15	MAP kinase 15	0.150825	-0.36329	-2.18221
Potri.007G020100	MAPK	ATMPK7,MPK7	MAP kinase 7	0.375724	0.827206	2.38134
Potri.009G066100	MAPK	ATMAPK3,ATMPK3,MPK3	mitogen-activated protein kinase 3	0.840259	1.268059	2.827765
Potri.016G134600	MAPK	ATWNK5,WNK5,ZIK1	with no lysine (K) kinase 5	-0.19452	-0.85263	-3.57592
Potri.019G128600	MAPK	WNK4,ZIK2	with no lysine (K) kinase 4	1.874732	3.910477	6.109323
Potri.002G129100	MAPK	MAPKKK5	mitogen-activated protein kinase kinase kinase 5	2.270076	2.651658	4.760434
Potri.006G275900	MYB	ATMYB3,MYB3	myb domain protein 3	0.18165	-3.73724	-5.89534
Potri.015G046200	MYB	AtMYB116,MYB116	myb domain protein 116	0.801198	0.244466	4.823622
Potri.001G099800	MYB	AtMYB103,MYB103	myb domain protein 103	-0.89285	0.003345	-6.89269
Potri.008G122	MYB	AtMYB62,BW	myb domain protein 62	2.278647	2.600001	4.73604

100		62B,BW62C,MYB62				
Potri.005G096600	MYB	ATMYB63,MYB63	myb domain protein 63	-1.93036	1.038558	4.839538
Potri.014G100800	MYB	ATMYB7,ATY49,MYB7	myb domain protein 7	-1.23284	-2.49977	-2.40649
Potri.002G038500	MYB	ATMYB14,MYB14,MYB14AT	myb domain protein 14	3.120098	-0.35623	3.020664
Potri.017G112300	MYB	AS1,ATMYB91,ATPHAN,MYB91	myb-like HTH transcriptional regulator family protein	-0.12171	-0.56825	-2.27985
Potri.002G191800	MYB	ATMYB2,MYB2	myb domain protein 2	10.4439	13.58048	17.55992
Potri.008G101400	MYB	AtMYB108,BOS1,MYB108	myb domain protein 108	2.521692	2.309691	4.809925
Potri.019G036400	MYB	ATMYB5,MYB5	myb domain protein 5	0.618005	-0.64877	2.420514
Potri.005G224100	MYB	ATMYB15,ATY19,MYB15	myb domain protein 15	5.437281	4.985401	6.660149
Potri.010G195000	MYB	AtMYB109,MYB109	myb domain protein 109	0.536055	1.319922	2.830621
Potri.002G157600	MYB	AtMYB17,MYB17	myb domain protein 17	-1.31785	-3.39716	-3.2952
Potri.002G185900	MYB	AtMYB55,MYB55	myb domain protein 55	-0.08162	-0.82112	-2.02229
Potri.012G127700	MYB	AtMYB42,MYB42	myb domain protein 42	-0.92373	-0.58088	-3.39139
Potri.009G134000	MYB	ATMYB4,MYB4	myb domain protein 4	-1.74612	-2.60384	-3.18092
Potri.006G241700	MYB	AtMYB3R4,MYB3R-4	myb domain protein 3r-4	-1.4203	-2.12232	-3.16104
Potri.009G053	MYB	ATMYB46,M	myb domain protein 46	-1.14403	-0.16985	-3.39891

900		YB46				
Potri.003G064 600	MYB	ATMYB66,MYB66,WER,WER1	myb domain protein 66	-0.71397	-3.11034	-5.47158
Potri.017G130 300	MYB	AtMYB43,MYB43	myb domain protein 43	1.551338	0.628116	2.805616
Potri.013G067 000	MYB	AtMYB56,MYB56	myb domain protein 56	2.319104	-0.4109	1.404222
Potri.002G130 200	MYB	ATS,KAN4	Homeodomain-like superfamily protein	2.351179	4.969485	6.037567
Potri.004G215 100	MYB	AtMYB36,MYB36	myb domain protein 36	5.398639	5.475469	7.931754
Potri.002G113 700	MYB	ATMYB68,MYB68	myb domain protein 68	1.340891	3.688904	6.379272
Potri.002G180 800	MYB	LHY,LHY1	Homeodomain-like superfamily protein	-0.10628	0.316499	-6.76205
Potri.010G055 000	MYB	0	Homeodomain-like superfamily protein	-0.884	-1.08156	-2.79731
Potri.012G038 300	MYB	EPR1,RVE7	Homeodomain-like superfamily protein	-1.47733	-1.3731	-3.95476
Potri.004G021 300	MYB	CPC	Homeodomain-like superfamily protein	-0.42969	-2.152	-1.75358
Potri.016G083 900	MYB	0	Homeodomain-like superfamily protein	-0.70524	-0.43409	-6.11837
Potri.009G116 600	MYB	ATRL1,RL1,RSM2	RAD-like 1	0.627332	2.345189	-1.15274
Potri.010G240 800	MYB	0	Duplicated homeodomain-like superfamily protein	0.25838	-1.04137	1.993354
Potri.008G064 200	MYB	0	Duplicated homeodomain-like superfamily protein	-0.6206	-0.56772	-2.66852
Potri.005G135 300	Peroxidases	0	Peroxidase superfamily protein	-0.03399	2.192596	2.690571
Potri.006G069	Peroxidases	0	Peroxidase superfamily protein	2.715289	2.272175	2.656943

600						
Potri.003G214900	Peroxidases	0	Peroxidase superfamily protein	-14.9177	1.044461	2.734086
Potri.004G134800	Peroxidases	0	Peroxidase superfamily protein	-0.02825	-2.49951	-4.00231
Potri.013G083600	Peroxidases	0	Peroxidase superfamily protein	4.706363	2.181965	3.964694
Potri.001G011500	Peroxidases	ATPA2,PA2	peroxidase 2	9.741529	6.004204	5.383578
Potri.002G018000	Peroxidases	0	Peroxidase superfamily protein	-0.13996	-2.81623	-1.86281
Potri.017G064100	Peroxidases	RHS19	root hair specific 19	2.501763	3.252281	6.347085
Potri.012G112200	Proteolysis	AMC1,ATMC1,ATMCPB1,LOL3,MCP1B	metacaspase 1	2.270378	2.101686	3.860863
Potri.005G179900	Proteolysis	0	Eukaryotic aspartyl protease family protein	10.76708	14.36086	18.00679
Potri.010G053600	Proteolysis	0	0	-0.32823	-1.26792	-2.13628
Potri.010G044300	Proteolysis	0	Protein of Unknown Function (DUF239)	-0.71209	-1.44449	-2.55078
Potri.002G013900	Proteolysis	0	Prolyl oligopeptidase family protein	1.008413	2.001098	2.839387
Potri.018G111000	Proteolysis	0	alpha/beta-Hydrolases superfamily protein	-0.93224	-2.59863	-3.21372
Potri.006G204700	Proteolysis	0	Eukaryotic aspartyl protease family protein	-0.48038	-2.00596	-2.77857
Potri.014G075600	Proteolysis	0	UBX domain-containing protein	-0.33666	-1.63943	-2.09363
Potri.007G099200	Proteolysis	0	Eukaryotic aspartyl protease family protein	-0.71824	-2.37638	-1.03308
Potri.009G111	Proteolysis	0	S-adenosyl-L-methionine-dependent methyltransferases superfamily	-1.08553	-1.5015	-3.8229

300			protein			
Potri.016G024500	Proteolysis	AtMC9,MC9	metacaspase 9	-1.58881	-0.1098	-2.17476
Potri.013G120200	Proteolysis	SCPL19,SNG2	serine carboxypeptidase-like 19	-1.0368	-1.58811	-2.40854
Potri.006G153300	Proteolysis	0	Peptidase M28 family protein	-0.74284	-2.046	-3.39442
Potri.005G221000	Proteolysis	0	Serine protease inhibitor, potato inhibitor I-type family protein	5.411788	3.069693	7.178863
Potri.005G113200	Proteolysis	ATRMR1,RMR1	receptor homology region transmembrane domain ring H2 motif protein 1	-1.09267	0.536715	2.078889
Potri.014G074600	Proteolysis	ATSBT1.1,SBTI1.1	subtilase family protein	0.165929	-0.81069	-2.13618
Potri.001G450600	Proteolysis	ATSBT3.3,SBT3.3	Subtilase family protein	0.743073	-1.57731	-4.30285
Potri.007G102100	Proteolysis	AIR3	Subtilisin-like serine endopeptidase family protein	0.931083	4.068182	4.315449
Potri.006G141200	Proteolysis	0	Subtilase family protein	0.324283	-0.62816	-2.19139
Potri.006G076200	Proteolysis	SLP3	subtilisin-like serine protease 3	-0.99493	-2.04486	-3.13753
Potri.003G071800	Proteolysis	0	Subtilase family protein	-0.70399	-1.41581	-2.42266
Potri.001G440300	Proteolysis	0	Subtilase family protein	-1.05139	-2.30955	-3.53291
Potri.018G143400	Proteolysis	0	PA-domain containing subtilase family protein	-0.96531	-2.52611	-4.87306
Potri.004G173900	Proteolysis	SLP2	subtilisin-like serine protease 2	-0.87274	-0.63251	-2.09907
Potri.011G076700	Proteolysis	0	subtilase family protein	-0.51177	-1.2575	-2.12075
Potri.012G131500	Proteolysis	ATSBT1.3,SBT1.3	subtilase 1.3	0.114268	-0.13415	-2.15655

Potri.002G124 500	Proteolysis	0	Subtilisin-like serine endopeptidase family protein	-0.1645	2.028702	1.893859
Potri.002G120 400	Proteolysis	ARA12	Subtilase family protein	0.418222	-0.10704	-2.94662
Potri.010G153 400	Proteolysis	APG8H,ATG8 I	Ubiquitin-like superfamily protein	0.918733	-0.56933	3.311984
Potri.004G057 700	Proteolysis	XBCP3	xylem bark cysteine peptidase 3	1.62268	2.566603	1.983463
Potri.005G256 000	Proteolysis	XCP2	xylem cysteine peptidase 2	-0.71344	-0.62261	-3.1505
Potri.017G148 200	Proteolysis	ULP1A	UB-like protease 1A	-0.35146	2.162187	3.636511
Potri.001G225 800	Proteolysis	ATCYS6,ATC YSB,CYSB	cystatin B	1.70726	1.330808	4.860472
Potri.010G234 300	Proteolysis	0	Cysteine proteinases superfamily protein	0.53338	0.858694	2.477204
Potri.004G207 600	Proteolysis	XCP1	xylem cysteine peptidase 1	-0.32864	-1.41701	-2.15764
Potri.008G036 900	Proteolysis	0	Cysteine proteinases superfamily protein	2.059563	1.942236	4.409865
Potri.002G054 900	Proteolysis	0	Eukaryotic aspartyl protease family protein	5.396173	2.298292	6.811897
Potri.001G240 600	Proteolysis	0	Eukaryotic aspartyl protease family protein	-0.72373	-2.80183	-4.51149
Potri.010G128 200	Proteolysis	0	Eukaryotic aspartyl protease family protein	0.30117	-0.57223	-2.62003
Potri.010G003 400	Proteolysis	0	Saposin-like aspartyl protease family protein	0.280305	-0.97928	-4.02299
Potri.017G131 800	Proteolysis	0	Eukaryotic aspartyl protease family protein	0.182858	-1.06271	-2.31845
Potri.019G002 100	Proteolysis	0	Eukaryotic aspartyl protease family protein	-0.11889	-0.15556	-2.23407
Potri.005G063	Proteolysis	0	Eukaryotic aspartyl protease family protein	-0.74561	-2.08494	-2.05052

000						
Potri.004G037800	Proteolysis	scpl31	serine carboxypeptidase-like 31	9.787813	14.4391	17.02198
Potri.003G164000	Proteolysis	SCPL45	serine carboxypeptidase-like 45	-0.15218	-1.99279	-3.6505
Potri.001G065900	Proteolysis	SCPL45	serine carboxypeptidase-like 45	-1.01462	-2.55817	-3.67716
Potri.001G291800	Proteolysis	scpl18	serine carboxypeptidase-like 18	-1.07446	-1.22962	-2.36784
Potri.001G290800	Proteolysis	scpl6	serine carboxypeptidase-like 6	-1.17072	-1.01865	-2.35646
Potri.009G031900	Proteolysis	ATRBL1,RBL1	RHOMBOID-like 1	-1.44958	-3.05653	-2.76912
Potri.004G088700	Proteolysis	DegP7	DegP protease 7	-0.4093	1.84182	2.700141
Potri.010G220200	Proteolysis	SCPL27	serine carboxypeptidase-like 27	-2.52854	-1.52737	-3.68225
Potri.014G109000	Proteolysis	0	serine protease inhibitor, Kazal-type family protein	0.487082	-2.71956	5.448684
Potri.014G108800	Proteolysis	0	serine protease inhibitor, Kazal-type family protein	2.435287	-1.31467	7.220448
Potri.015G142000	Proteolysis	ATRBL3,RBL3	RHOMBOID-like protein 3	-0.64548	-1.80684	-2.3205
Potri.007G072300	Proteolysis	scpl35	serine carboxypeptidase-like 35	0.635136	1.79898	3.339077
Potri.005G091700	Proteolysis	SCPL34	serine carboxypeptidase-like 34	-0.83196	-2.32723	-4.4229
Potri.015G110000	Proteolysis	CLPD,ERD1,SAG15	Clp ATPase	0.468492	1.29954	4.375695
Potri.001G289700	Proteolysis	ATEGY3,EGY3	ethylene-dependent gravitropism-deficient and yellow-green-like 3	-1.41725	-1.21197	-3.00966
Potri.008G027500	Proteolysis	0	Matrixin family protein	2.019482	1.255993	3.350169

Potri.019G073800	Proteolysis	MMP	matrix metalloproteinase	2.535647	1.408426	0.728418
Potri.006G050700	Proteolysis	0	Insulinase (Peptidase family M16) family protein	0.319959	1.631651	1.986579
Potri.001G157500	Proteolysis	0	Matrixin family protein	1.343427	1.086978	3.815022
Potri.017G084000	Proteolysis	ATFTSH6,FTSH6	FTSH protease 6	-0.67164	-2.11532	-1.47291
Potri.005G249200	Proteolysis	FTSH5,VAR1	FtsH extracellular protease family	2.152036	1.355694	1.588541
Potri.005G119200	Proteolysis	0	P-loop containing nucleoside triphosphate hydrolases superfamily protein	0.795214	2.518699	3.36109
Potri.002G175600	Proteolysis	0	P-loop containing nucleoside triphosphate hydrolases superfamily protein	1.370652	1.621656	4.857492
Potri.017G123800	Proteolysis	0	P-loop containing nucleoside triphosphate hydrolases superfamily protein	1.087476	1.739054	3.443904
Potri.007G012400	Proteolysis	BCS1	cytochrome BC1 synthesis	1.517693	2.652453	2.2222
Potri.011G052800	Proteolysis	NSF	AAA-type ATPase family protein	1.131431	1.802229	3.089012
Potri.011G143300	Proteolysis	0	AAA-type ATPase family protein	1.505236	1.869808	0.514302
Potri.005G119900	Proteolysis	0	P-loop containing nucleoside triphosphate hydrolases superfamily protein	-0.23583	-0.2947	2.206882
Potri.004G012500	Proteolysis	AATP1	AAA-ATPase 1	-0.02894	2.164353	3.586598
Potri.006G169000	Proteolysis	0	P-loop containing nucleoside triphosphate hydrolases superfamily protein	4.603632	2.41302	7.518304
Potri.014G039900	Proteolysis	DA1	DA1	0.633744	1.163128	3.634478
Potri.001G090300	Proteolysis	ATPI4K GAMMA 4,PI4K	phosphoinositide 4-kinase gamma 4	-0.9949	-1.67939	-2.89552

		GAMMA 4,UBDK GAMMA 4				
Potri.004G002 000	Proteolysis	0	Ubiquitin-specific protease family C19-related protein	-0.13994	-1.49379	-3.56829
Potri.015G013 600	Proteolysis	0	Phosphatidylinositol 3- and 4-kinase ;Ubiquitin family protein	0.731917	0.458633	2.797208
Potri.004G211 600	Proteolysis	0	Ubiquitin-like superfamily protein	-2.72658	-3.04614	-1.38118
Potri.003G011 300	Proteolysis	0	ubiquitin family protein	0.929298	1.331084	1.894997
Potri.011G026 600	Proteolysis	UBQ3	polyubiquitin 3	0.478328	-0.18953	2.892762
Potri.006G129 600	Proteolysis	UBQ4	ubiquitin 4	-0.5685	0.042434	2.314117
Potri.001G254 500	Proteolysis	UBC20	ubiquitin-conjugating enzyme 20	-0.70593	-2.1049	-4.27999
Potri.004G043 500	Proteolysis	ATUBC24,PH O2,UBC24	phosphate 2	2.844253	3.165469	4.103398
Potri.009G049 600	Proteolysis	UBC19	ubiquitin-conjugating enzyme19	-1.43019	-3.07924	-4.07148
Potri.002G205 400	Proteolysis	BAH1,NLA	SPX (SYG1/Pho81/XPR1) domain-containing protein	-0.92039	-2.05995	0.34983
Potri.006G154 800	Proteolysis	0	RING/FYVE/PHD zinc finger superfamily protein	0.724176	1.438228	2.219234
Potri.010G053 000	Proteolysis	0	RING/U-box superfamily protein	-1.48648	-2.91976	-3.2011
Potri.001G148 600	Proteolysis	0	SBP (S-ribonuclease binding protein) family protein	1.590296	0.913225	2.494181
Potri.019G010 600	Proteolysis	0	RING/U-box superfamily protein	0.504848	5.391027	7.354993
Potri.001G067 700	Proteolysis	ORTH2,VIM1	Zinc finger (C3HC4-type RING finger) family protein	0.376552	1.55568	1.953726

Potri.015G074200	Proteolysis	0	ARM repeat superfamily protein	1.223066	0.713661	2.828178
Potri.002G131300	Proteolysis	ARI8,ATARI8	IBR domain-containing protein	0.277907	0.75529	1.957228
Potri.017G049800	Proteolysis	0	RING/U-box superfamily protein	0.498537	1.103109	2.930651
Potri.001G162000	Proteolysis	ATL3	RING/U-box superfamily protein	2.199829	1.50568	5.616657
Potri.015G043900	Proteolysis	0	RING/U-box superfamily protein	-0.0306	0.719829	2.491776
Potri.012G019900	Proteolysis	0	ARM repeat superfamily protein	0.311322	1.663446	2.696843
Potri.005G081300	Proteolysis	RHA2B	RING-H2 finger protein 2B	-2.35072	-1.11828	2.67368
Potri.010G115600	Proteolysis	0	RING/FYVE/PHD zinc finger superfamily protein	1.167635	0.58852	2.507355
Potri.007G064400	Proteolysis	RHA3A	RING-H2 finger A3A	-0.37497	-2.64702	1.598154
Potri.018G098000	Proteolysis	0	RING/U-box superfamily protein	1.596069	-0.86492	2.398545
Potri.007G051000	Proteolysis	0	RING/U-box superfamily protein with ARM repeat domain	0.482546	0.385473	1.912184
Potri.007G031600	Proteolysis	0	RING/U-box superfamily protein	0.672258	1.121201	2.248188
Potri.001G154500	Proteolysis	0	RING/U-box superfamily protein	0.792229	2.721924	3.921076
Potri.003G148400	Proteolysis	0	Zinc finger (C3HC4-type RING finger) family protein	0.525153	0.535002	3.15752
Potri.011G063100	Proteolysis	0	RING/U-box superfamily protein	2.407268	5.815004	7.493202
Potri.002G170300	Proteolysis	0	RING/U-box superfamily protein	0.580386	1.707982	3.045463
Potri.005G036	Proteolysis	ATL6	RING/U-box superfamily protein	1.542821	0.856725	2.773276

800						
Potri.003G148300	Proteolysis	0	RING/U-box superfamily protein	0.519773	0.078274	1.962377
Potri.013G091300	Proteolysis	0	RING/U-box superfamily protein	-0.17354	-2.08071	-2.57982
Potri.008G219200	Proteolysis	ATL2,TL2	TOXICOS EN LEVADURA 2	2.244291	0.691452	2.128106
Potri.001G336800	Proteolysis	0	zinc finger (C3HC4-type RING finger) family protein	0.257764	0.850867	2.237741
Potri.001G243300	Proteolysis	0	zinc finger (C3HC4-type RING finger) family protein	2.063256	0.636817	1.637756
Potri.006G000900	Proteolysis	0	Zinc finger (C3HC4-type RING finger) family protein	0.000786	-0.95041	-2.57231
Potri.008G035700	Proteolysis	ATPUB14,PUB14	plant U-box 14	0.467161	0.759701	1.977562
Potri.014G053600	Proteolysis	ATL4,TL4	TOXICOS EN LEVADURA 4	-2.25432	-1.08532	0.998643
Potri.009G062100	Proteolysis	BB,BB2	RING/U-box superfamily protein	-0.81978	-1.92164	-3.02673
Potri.004G042700	Proteolysis	ATRMA1,RMA1	RING membrane-anchor 1	0.269449	-0.07665	3.114766
Potri.011G120800	Proteolysis	ATRMA3,RMA3	RING membrane-anchor 3	0.431851	-0.16292	1.91157
Potri.018G085000	Proteolysis	0	RING/U-box superfamily protein	1.469677	2.211788	1.650623
Potri.018G098500	Proteolysis	0	RING/U-box superfamily protein	-0.3595	-0.7745	-3.01373
Potri.017G049300	Proteolysis	0	RING/U-box superfamily protein	-0.50101	1.498034	2.403302
Potri.005G228100	Proteolysis	0	ARM repeat superfamily protein	1.115876	2.114064	1.274862
Potri.018G119600	Proteolysis	0	RING/U-box superfamily protein	0.623014	0.999374	3.745266

Potri.006G107600	Proteolysis	0	ARM repeat superfamily protein	-0.635	0.102838	2.987181
Potri.019G130100	Proteolysis	0	RING/U-box superfamily protein	-1.38294	-2.19007	-5.06641
Potri.016G105900	Proteolysis	0	RING/FYVE/PHD zinc finger superfamily protein	0.596807	0.948401	2.464233
Potri.005G071300	Proteolysis	ATRING1,RING1	RING/U-box superfamily protein	2.316847	2.7048	2.008097
Potri.016G003000	Proteolysis	RGLG2	RING domain ligase2	6.291951	3.358528	6.670016
Potri.010G049600	Proteolysis	RGLG2	RING domain ligase2	1.038234	1.19903	3.837361
Potri.013G025400	Proteolysis	0	RING/U-box superfamily protein	-0.10922	-0.57223	2.754661
Potri.018G005600	Proteolysis	0	RING/U-box superfamily protein	0.397911	0.378752	2.509484
Potri.003G129900	Proteolysis	0	RING/U-box superfamily protein	0.72353	-0.12616	2.279292
Potri.014G053500	Proteolysis	0	RING/U-box superfamily protein	0.103487	0.759668	2.108531
Potri.001G453800	Proteolysis	0	RING/U-box superfamily protein	-1.04942	-0.38482	-2.13354
Potri.011G094800	Proteolysis	0	RING/U-box superfamily protein	-0.01825	0.83315	3.272697
Potri.001G001500	Proteolysis	ATCRT1	RING/U-box superfamily protein	0.578372	0.087957	1.893455
Potri.009G034800	Proteolysis	0	zinc finger (C3HC4-type RING finger) family protein	2.612097	1.010206	3.129646
Potri.005G244800	Proteolysis	0	RING/U-box superfamily protein	0.186959	1.176373	3.943473
Potri.018G104900	Proteolysis	SKIP1	SKP1 interacting partner 1	0.124656	0.651446	2.628539
Potri.007G048	Proteolysis	SKIP2,VFB4	SKP1/ASK1-interacting protein 2	1.906101	-0.15365	2.359401

200						
Potri.010G042900	Proteolysis	0	Kelch repeat-containing F-box family protein	-1.52043	-2.19113	-3.32342
Potri.003G062000	Proteolysis	0	Galactose oxidase/kelch repeat superfamily protein	2.639431	1.089809	-0.07681
Potri.001G230600	Proteolysis	KUF1	KAR-UP F-box 1	1.957133	-0.66363	2.753312
Potri.002G034600	Proteolysis	AtTLP6,TLP6	tubby like protein 6	0.230366	-0.5812	-2.07972
Potri.008G006300	Proteolysis	0	Kelch repeat-containing F-box family protein	-1.04294	-1.88178	-2.22996
Potri.011G042900	Proteolysis	0	FBD, F-box and Leucine Rich Repeat domains containing protein	-0.83959	0.15071	-3.14889
Potri.005G105000	Proteolysis	AMR1	ascorbic acid mannose pathway regulator 1	2.242317	1.557739	2.630374
Potri.010G059200	Proteolysis	0	Galactose oxidase/kelch repeat superfamily protein	-0.78322	-0.10902	2.946237
Potri.013G008800	Proteolysis	0	F-box family protein	11.34272	12.79393	18.00944
Potri.010G105700	Proteolysis	ADO3,FKF1	flavin-binding, kelch repeat, f box 1	1.39132	-0.4574	2.922586
Potri.002G099400	Proteolysis	0	transferases, transferring glycosyl groups	0.542371	0.677348	1.982137
Potri.008G095600	Proteolysis	ATUPS2,UPS2	ureide permease 2	1.553167	1.404123	3.106116
Potri.008G095500	Proteolysis	ATUPS2,UPS2	ureide permease 2	2.344351	1.306338	4.266379
Potri.004G199300	Proteolysis	0	F-box family protein	5.134864	4.694027	4.641178
Potri.001G318300	Proteolysis	0	F-box family protein	13.33333	15.32396	20.44907
Potri.001G331500	Proteolysis	0	Galactose oxidase/kelch repeat superfamily protein	0.355364	-0.53576	2.440144

Potri.009G047600	Proteolysis	0	F-box family protein	3.498625	0.315582	2.631251
Potri.011G024200	Proteolysis	0	F-box/RNI-like/FBD-like domains-containing protein	0.835013	1.122345	2.262745
Potri.001G181000	Proteolysis	0	F-box family protein	1.097352	3.718315	6.072967
Potri.014G016200	Proteolysis	0	Galactose oxidase/kelch repeat superfamily protein	1.812687	2.601683	5.251883
Potri.004G005700	Proteolysis	0	0	-0.32222	2.064647	2.360203
Potri.005G027900	Proteolysis	ATCUL1,AXR6,CUL1	cullin 1	-0.4726	-0.84393	-3.79278
Potri.007G140400	Proteolysis	BT3	BTB and TAZ domain protein 3	0.11142	-0.36871	-2.12603
Potri.006G008800	Proteolysis	PAC1	20S proteasome alpha subunit C1	10.02401	12.3273	10.58896
Potri.T028500	PR-proteins	0	Disease resistance protein (CC-NBS-LRR class) family	0.043603	-0.24929	2.166294
Potri.013G098900	PR-proteins	0	Leucine-rich repeat (LRR) family protein	3.303059	0.622261	2.806352
Potri.001G437700	PR-proteins	AtRLP7,RLP7	receptor like protein 7	10.14851	16.56898	18.27601
Potri.018G138500	PR-proteins	0	NB-ARC domain-containing disease resistance protein	0.539835	0.738272	2.013661
Potri.003G150200	PR-proteins	0	Disease resistance protein (CC-NBS-LRR class) family	-10.6276	4.554116	5.946045
Potri.003G216200	PR-proteins	0	Disease resistance-responsive (dirigent-like protein) family protein	1.032842	3.093089	3.987283
Potri.003G134600	PR-proteins	0	Disease resistance-responsive (dirigent-like protein) family protein	3.589147	4.707692	6.364103
Potri.012G020600	PR-proteins	AtRLP12,RLP12	receptor like protein 12	0.028374	3.325459	3.583546
Potri.007G143000	PR-proteins	0	Disease resistance protein (TIR-NBS class)	1.148706	3.495537	4.173694

Potri.001G214600	PR-proteins	0	Disease resistance-responsive (dirigent-like protein) family protein	2.466897	1.138317	4.284659
Potri.001G043800	PR-proteins	0	disease resistance family protein / LRR family protein	0	10.69047	16.64317
Potri.001G023800	PR-proteins	0	Disease resistance-responsive (dirigent-like protein) family protein	-0.34079	-1.3164	-4.48695
Potri.017G015400	PR-proteins	0	LRR and NB-ARC domains-containing disease resistance protein	0.508205	1.112553	2.034039
Potri.001G025400	PR-proteins	0	NB-ARC domain-containing disease resistance protein	4.202361	5.889681	6.532919
Potri.010G150500	PR-proteins	ATHS1,HS1	heat stable protein 1	-0.58241	-3.12351	-4.45608
Potri.T026600	PR-proteins	0	LRR and NB-ARC domains-containing disease resistance protein	1.114927	1.759417	3.122711
Potri.019G114800	PR-proteins	0	Disease resistance protein (TIR-NBS-LRR class) family	-0.24918	1.442276	2.519544
Potri.009G162300	PR-proteins	SCRL11	SCR-like 11	-2.24623	-2.37834	-3.16694
Potri.001G406100	PR-proteins	0	NB-ARC domain-containing disease resistance protein	-1.11261	-1.51349	-2.42455
Potri.013G097000	PR-proteins	0	disease resistance protein (TIR-NBS-LRR class), putative	0.025211	0.623909	2.129621
Potri.012G033900	PR-proteins	ATCHIA,CHIA	chitinase A	5.573381	0.711111	9.753336
Potri.T005100	PR-proteins	0	Disease resistance protein (TIR-NBS-LRR class) family	0.254646	0.927642	2.727291
Potri.001G363400	PR-proteins	0	Disease resistance protein (TIR-NBS-LRR class) family	5.117267	5.219554	7.414864
Potri.011G105300	PR-proteins	AtRLP54,RLP54	receptor like protein 54	-0.99006	4.251037	5.437235
Potri.011G040800	PR-proteins	0	P-loop containing nucleoside triphosphate hydrolases superfamily protein	-0.23248	2.404297	2.586722
Potri.006G195300	PR-proteins	0	Disease resistance-responsive (dirigent-like protein) family protein	0.580876	1.190106	3.125615
Potri.007G039	PR-proteins	0	Disease resistance protein (CC-NBS-LRR class) family	-1.47454	1.747199	5.454162

200						
Potri.001G309900	PR-proteins	0	Kunitz family trypsin and protease inhibitor protein	-1.10417	-2.84838	7.665021
Potri.001G166800	PR-proteins	0	inter-alpha-trypsin inhibitor heavy chain-related	0.040956	-0.62319	-2.16512
Potri.004G000400	PR-proteins	ATKTI1,KTI1	kunitz trypsin inhibitor 1	-1.28148	2.565705	10.67383
Potri.019G121900	PR-proteins	0	Kunitz family trypsin and protease inhibitor protein	3.33964	2.945831	2.67194
Potri.011G008800	Redox state	0	transmembrane receptors;ATP binding	-1.12511	0.787369	4.701616
Potri.001G416500	Redox state	ATCXXS1,CXXS1	C-terminal cysteine residue is changed to a serine 1	-0.22682	-2.98514	0.631311
Potri.007G074000	Redox state	ATHX,ATX,THX	thioredoxin X	-0.54213	-0.55085	-2.98404
Potri.019G054800	Redox state	ATF1,TRXF1	thioredoxin F-type 1	-0.21007	-0.33536	-2.5194
Potri.002G073000	Redox state	ATHM4,ATM4,TRX-M4	thioredoxin M-type 4	2.386527	1.872624	1.347791
Potri.010G255500	Redox state	FTRA2	ferredoxin/thioredoxin reductase subunit A (variable subunit) 2	-0.30112	-1.37653	-2.24599
Potri.014G122800	Redox state	ATPDI11,ATPDIL2-1,MEE30,PDI11,UNE5	thioredoxin family protein	1.010198	1.408995	2.095789
Potri.008G040100	Redox state	ATPDI1,ATPDIL1-3,PDI1,PDIL1-3	PDI-like 1-3	0.513688	1.346075	2.373372
Potri.010G073200	Redox state	0	2-oxoglutarate (2OG) and Fe(II)-dependent oxygenase superfamily protein	3.823258	4.409556	5.520323
Potri.010G131100	Redox state	0	Cytochrome b561/ferric reductase transmembrane protein family	-0.43935	-1.69187	-3.56539
Potri.002G242	Redox state	ATCB5-B,B5	cytochrome B5 isoform B	2.054132	1.692202	0.702328

500		#4,CB5-B				
Potri.009G107600	Redox state	0	2-oxoglutarate (2OG) and Fe(II)-dependent oxygenase superfamily protein	0.636868	0.809247	2.17563
Potri.011G080400	Redox state	0	Cytochrome b561/ferric reductase transmembrane protein family	-0.33867	-1.94108	-2.15037
Potri.015G143700	Redox state	ACYB-2	Cytochrome b561/ferric reductase transmembrane protein family	-1.48355	-2.47572	-2.02145
Potri.005G161900	Redox state	APX4,TL29	ascorbate peroxidase 4	-0.31293	-1.65843	-2.73403
Potri.005G079400	Redox state	0	Cupredoxin superfamily protein	3.79756	2.02802	3.850256
Potri.017G125100	Redox state	DHAR3	dehydroascorbate reductase 1	-1.00543	-1.85125	-2.15229
Potri.017G126100	Redox state	VTC2	mannose-1-phosphate guanylyltransferase (GDP)s;GDP-galactose:mannose-1-phosphate guanylyltransferases;GDP-galactose:glucose-1-phosphate guanylyltransferases;GDP-galactose:myoinositol-1-phosphate guanylyltransferases;glucose-1-phosphate guanylyltransferase	1.526277	1.965772	6.587136
Potri.009G081300	Redox state	0	NAD(P)-linked oxidoreductase superfamily protein	-0.63196	-1.27168	-2.73154
Potri.007G126600	Redox state	ATGPX2,GPX2	glutathione peroxidase 2	2.919168	0.845442	2.585527
Potri.001G105200	Redox state	ATGPX6,GPX6,LSC803,PHGPX	glutathione peroxidase 6	0.638863	-0.60843	2.43138
Potri.001G104500	Redox state	ATECS1,CAD2,GSH1,GSHA,PAD2,RML1	glutamate-cysteine ligase	0.160902	1.630395	2.90845
Potri.009G110800	Redox state	AHB1,ARATHGLB1,ATGLB1,GLB1,HB1,NSHB1	hemoglobin 1	0.303617	-4.84316	-4.46998

Potri.016G098500	Redox state	0	SOUL heme-binding family protein	1.108483	1.816907	2.72298
Potri.004G049800	Redox state	GRX480,roxy19	Thioredoxin superfamily protein	1.2858	1.967387	3.397819
Potri.003G141800	Redox state	0	Glutaredoxin family protein	0.40504	0.849643	-5.57343
Potri.008G144800	Redox state	0	glutaredoxin-related	3.077656	2.992098	5.792134
Potri.008G214500	Redox state	0	Glutaredoxin family protein	0.581746	-3.59526	0.153504
Potri.014G133700	Redox state	0	Thioredoxin superfamily protein	-0.52909	-2.98769	-3.49569
Potri.014G134200	Redox state	0	Thioredoxin superfamily protein	-1.73704	-2.30772	-4.0324
Potri.002G091200	Redox state	0	electron carriers;protein disulfide oxidoreductases	0.649035	0.726827	2.216275
Potri.006G226900	Redox state	0	Thioredoxin superfamily protein	0.398482	1.477863	2.796357
Potri.010G243700	Redox state	0	Glutaredoxin family protein	-1.66795	-3.05597	-3.53996
Potri.008G214600	Redox state	0	Thioredoxin superfamily protein	-0.99109	-2.61338	-1.26683
Potri.006G137500	Redox state	ATPRX Q	Thioredoxin superfamily protein	-1.32988	-2.99422	-4.24268
Potri.005G100400	Redox state	CAT2	catalase 2	-0.523	-1.15499	-3.91825
Potri.012G112300	Respiratory burst	FSD2	Fe superoxide dismutase 2	-0.22495	-0.40064	-2.17376
Potri.005G026200	Respiratory burst	ATRBOHB,ATRBOHB-BETA,RBOHB	respiratory burst oxidase homolog B	0.475159	1.373881	2.108139
Potri.003G133300	Respiratory burst	ATRBOHF,ATRBOHF,R	respiratory burst oxidase protein F	0.391851	0.278624	2.055627

		BOH F,RBOHAP10 8,RBOHF				
Potri.001G070 900	R-genes	ATRBOHD,R BOHD	respiratory burst oxidase homologue D	2.689079	1.371825	3.831546
Potri.004G179 200	SA	UGT74E2	Uridine diphosphate glycosyltransferase 74E2	0.322364	0.296812	2.571941
Potri.T138800	SA	0	S-adenosyl-L-methionine-dependent methyltransferases superfamily protein	1.870225	2.304128	4.384516
Potri.007G021 400	SA	0	S-adenosyl-L-methionine-dependent methyltransferases superfamily protein	-0.9871	-1.1169	-2.94885
Potri.007G140 600	SA	UGT74F1	UDP-glycosyltransferase 74 F1	-1.20225	-1.61561	-2.96661
Potri.007G002 100	Secondary metabolites	CAMS1	camelliol C synthase 1	7.797727	6.425008	6.539204
Potri.012G068 800	Secondary metabolites	0	Pyridine nucleotide-disulphide oxidoreductase family protein	-0.60369	-0.82409	-2.97787
Potri.017G090 700	Secondary metabolites	MUB2	membrane-anchored ubiquitin-fold protein 2	1.000685	1.132113	2.361031
Potri.015G043 400	Secondary metabolites	GGR	geranylgeranyl reductase	-0.09772	-4.81536	-5.53157
Potri.014G168 700	Secondary metabolites	ACAT2,EMB1 276	acetoacetyl-CoA thiolase 2	1.86282	1.248248	2.128307
Potri.001G457 000	Secondary metabolites	HMG1,HMG R1	hydroxy methylglutaryl CoA reductase 1	2.766137	3.338701	1.524356
Potri.010G237 900	Secondary metabolites	0	GHMP kinase family protein	1.857329	1.15105	2.649191
Potri.006G003 400	Secondary metabolites	FPS1	farnesyl diphosphate synthase 1	1.018457	0.865046	2.07728
Potri.017G014 200	Secondary metabolites	0	Tyrosine transaminase family protein	-1.22416	0.498821	3.587446
Potri.018G090 700	Secondary metabolites	ATHPT,HPT1, TPT1,VTE2	homogentisate phytyltransferase 1	0.450619	1.211289	2.683188

Potri.008G159400	Secondary metabolites	APG1,E37,IEP37,VTE3	S-adenosyl-L-methionine-dependent methyltransferases superfamily protein	-0.39831	-0.69622	-4.41695
Potri.006G240100	Secondary metabolites	ATSDX1,VTE1	tocopherol cyclase, chloroplast / vitamin E deficient 1 (VTE1) / sucrose export defective 1 (SXD1)	0.897062	1.664002	2.259382
Potri.001G100200	Secondary metabolites	B1,BCH1,BETA-OHASE 1,chy1	beta-hydroxylase 1	1.243839	1.754294	4.304873
Potri.018G043000	Secondary metabolites	ATCCD1,ATNCED1,CCD1,NCED1	carotenoid cleavage dioxygenase 1	5.628338	5.319014	7.450953
Potri.004G037900	Secondary metabolites	GES,TPS04,TPS4	terpene synthase 04	3.760261	-1.47689	2.550192
Potri.011G032300	Secondary metabolites	ATTPS14,TPS14	terpene synthase 14	2.412742	-2.36265	-7.13444
Potri.011G031800	Secondary metabolites	ATTPS-CIN,TPS-CIN	terpene synthase-like sequence-1,8-cineole	1.397785	6.02622	10.30643
Potri.005G095500	Secondary metabolites	ATTPS21,TPS21	terpene synthase 21	3.993412	4.632149	7.028172
Potri.010G125400	Secondary metabolites	0	NAD(P)-binding Rossmann-fold superfamily protein	-0.12087	0.047813	-3.10247
Potri.001G140700	Secondary metabolites	0	NAD(P)-binding Rossmann-fold superfamily protein	4.357466	5.119516	5.129341
Potri.002G004500	Secondary metabolites	0	NAD(P)-binding Rossmann-fold superfamily protein	1.48403	0.654017	2.044936
Potri.010G186300	Secondary metabolites	0	HXXXD-type acyl-transferase family protein	2.634152	1.383611	1.672208
Potri.004G050400	Secondary metabolites	0	O-methyltransferase family protein	5.401817	4.120194	4.159227
Potri.006G265500	Secondary metabolites	0	Major facilitator superfamily protein	3.353414	2.704234	3.598161
Potri.014G025500	Secondary metabolites	0	HXXXD-type acyl-transferase family protein	2.762704	2.852178	5.780399
Potri.018G146	Secondary	ATC4H,C4H,	cinnamate-4-hydroxylase	2.383399	0.459376	3.582944

100	metabolites	CYP73A5,REF 3				
Potri.001G036 900	Secondary metabolites	4CL2,AT4CL2	4-coumarate:CoA ligase 2	-0.31257	-0.83381	-3.88951
Potri.003G099 700	Secondary metabolites	0	AMP-dependent synthetase and ligase family protein	-1.85462	-1.69993	-2.26912
Potri.010G057 000	Secondary metabolites	0	AMP-dependent synthetase and ligase family protein	0.552884	0.763284	3.832278
Potri.001G042 900	Secondary metabolites	HCT	hydroxycinnamoyl-CoA shikimate/quinate hydroxycinnamoyl transferase	0.223173	-0.61174	-3.63944
Potri.001G304 800	Secondary metabolites	CCoAOMT1	S-adenosyl-L-methionine-dependent methyltransferases superfamily protein	1.717949	1.538156	3.149283
Potri.001G045 500	Secondary metabolites	ATCCR1,CCR 1,IRX4	cinnamoyl coa reductase 1	7.593198	5.061266	9.271197
Potri.017G110 500	Secondary metabolites	0	NAD(P)-binding Rossmann-fold superfamily protein	-1.13969	2.315328	2.134105
Potri.001G451 100	Secondary metabolites	ATOMT1,OM T1	O-methyltransferase 1	0.76677	1.545058	3.311994
Potri.016G078 300	Secondary metabolites	ATCAD6,CA D6	cinnamyl alcohol dehydrogenase 6	1.114099	1.692927	2.596087
Potri.001G268 600	Secondary metabolites	ATCAD7,CA D7,ELI3,ELI3-1	elicitor-activated gene 3-1	2.745709	3.22399	6.361847
Potri.011G158 500	Secondary metabolites	0	FAD-binding Berberine family protein	4.524538	2.894804	7.107256
Potri.013G026 000	Secondary metabolites	0	NAD(P)-binding Rossmann-fold superfamily protein	-0.12307	-0.1958	-2.69415
Potri.006G040 900	Secondary metabolites	SSL2	strictosidine synthase-like 2	-0.66078	-1.25441	-4.13106
Potri.005G099 400	Secondary metabolites	ATSSL4,SSL4	strictosidine synthase-like 4	-1.12094	-1.58489	-2.42848
Potri.008G110 000	Secondary metabolites	0	Calcium-dependent phosphotriesterase superfamily protein	0.297667	-0.44794	-2.62663

Potri.008G172500	Secondary metabolites	ATLEUC1,IIL1	isopropyl malate isomerase large subunit 1	-0.70589	-2.71876	-1.15013
Potri.002G064000	Secondary metabolites	0	Pyridoxal phosphate (PLP)-dependent transferases superfamily protein	0.295366	1.820803	3.188952
Potri.014G180300	Secondary metabolites	CER1	Fatty acid hydroxylase superfamily	-0.7865	-2.1194	-3.66493
Potri.010G125300	Secondary metabolites	CER6,CUT1,G2,KCS6,POP1	3-ketoacyl-CoA synthase 6	-0.09182	-0.02495	-2.21395
Potri.001G106800	Secondary metabolites	ASAT1,ATASAT1,ATSAT1	acyl-CoA sterol acyl transferase 1	18.63922	16.56692	18.40577
Potri.006G177500	Secondary metabolites	CER3,FLP1,WAX2,YRE	Fatty acid hydroxylase superfamily	-2.13158	-0.24044	3.075039
Potri.001G030600	Secondary metabolites	0	UDP-Glycosyltransferase superfamily protein	13.25372	13.45105	19.18744
Potri.010G192400	Secondary metabolites	0	HXXXD-type acyl-transferase family protein	3.686043	-2.28678	2.812957
Potri.006G101200	Secondary metabolites	0	2-oxoglutarate (2OG) and Fe(II)-dependent oxygenase superfamily protein	1.359614	-1.92547	-4.62079
Potri.019G118000	Secondary metabolites	0	HXXXD-type acyl-transferase family protein	2.546053	1.709978	5.772704
Potri.001G051500	Secondary metabolites	ATCHS,CHS,TT4	Chalcone and stilbene synthase family protein	1.000823	4.337658	4.749
Potri.009G044600	Secondary metabolites	UGT71D1	UDP-glucosyl transferase 71D1	0.033277	0.162334	3.550468
Potri.001G451900	Secondary metabolites	0	2-oxoglutarate (2OG) and Fe(II)-dependent oxygenase superfamily protein	-2.54014	-1.00215	2.121971
Potri.012G006300	Secondary metabolites	DMR6	2-oxoglutarate (2OG) and Fe(II)-dependent oxygenase superfamily protein	-0.80785	4.263664	6.2488
Potri.006G179700	Secondary metabolites	0	UDP-Glycosyltransferase superfamily protein	-1.93902	-0.38585	-5.68195
Potri.002G033600	Secondary metabolites	DFR,M318,TT3	dihydroflavonol 4-reductase	1.040074	3.906418	3.497574
Potri.005G113	Secondary	F3\'H,F3H,TT	flavanone 3-hydroxylase	-0.1841	2.272905	1.99338

700	metabolites	6				
Potri.003G066600	Secondary metabolites	CYP75B1,D501,TT7	Cytochrome P450 superfamily protein	-2.58891	-4.30087	-5.61074
Potri.001G382400	Secondary metabolites	ATSRG1,SRG1	senescence-related gene 1	2.240411	4.524403	7.521959
Potri.002G034400	Secondary metabolites	0	NmrA-like negative transcriptional regulator family protein	-0.60195	-1.1531	-3.22763
Potri.008G116500	Secondary metabolites	0	NAD(P)-binding Rossmann-fold superfamily protein	1.145183	3.515849	4.67993
Potri.009G118300	Secondary metabolites	0	NmrA-like negative transcriptional regulator family protein	2.103358	0.871423	2.543112
Potri.001G248700	Secondary metabolites	ATLMCO4,IRX12,LAC4,LMCO4	Laccase/Diphenol oxidase family protein	-0.01197	-0.55465	-3.26839
Potri.004G156400	Secondary metabolites	LAC11	laccase 11	0.196914	-0.75241	-3.64148
Potri.010G183500	Secondary metabolites	LAC12	laccase 12	0.029704	1.285153	3.292484
Potri.005G200500	Secondary metabolites	LAC14	laccase 14	0	0	20.1757
Potri.001G401300	Secondary metabolites	ATLAC17,LAC17	laccase 17	-0.63808	-2.40124	-6.20241
Potri.015G069600	Signaling	ATEDS1,EDS1	alpha/beta-Hydrolases superfamily protein	0.718497	2.580894	3.78753
Potri.005G111400	Signaling	ATMLO4,MLO4	Seven transmembrane MLO family protein	-0.95369	-1.30229	-4.54991
Potri.010G220500	Signaling	ATMLO6,MLO6	Seven transmembrane MLO family protein	2.28493	0.518222	1.50675
Potri.004G050000	Signaling	ATMLO12,MLO12	Seven transmembrane MLO family protein	-0.26168	0.519371	3.440631
Potri.007G146900	Signaling	ATMLO1,MLO1	Seven transmembrane MLO family protein	-0.26198	0.110979	2.008176
Potri.018G011	Signaling	ATGLR2.2,GL	glutamate receptor 2.2	12.13532	12.16395	16.45204

700		R2.2				
Potri.006G268200	Signaling	ATGLR2.8,GLR2.8	glutamate receptor 2.8	3.991762	3.958067	5.960406
Potri.001G374600	Signaling	ATGLR2.7,GLR2.7	glutamate receptor 2.7	5.896573	6.56488	9.310988
Potri.005G102600	Signaling	ATGLR3.6,GLR3.6	glutamate receptor 3.6	1.119582	2.944696	1.317312
Potri.006G094300	Signaling	0	PAR1 protein	2.893879	0.888674	4.445067
Potri.002G098800	Signaling	EXO	Phosphate-responsive 1 family protein	2.468022	1.273499	4.970655
Potri.012G141800	Signaling	0	PAR1 protein	4.483168	4.971905	6.767134
Potri.001G144100	Signaling	PIRL4	plant intracellular ras group-related LRR 4	1.638263	2.037558	4.153959
Potri.019G094200	Signaling	0	Leucine-rich repeat transmembrane protein kinase protein	-0.23438	0.532654	2.626513
Potri.002G242700	Signaling	0	Leucine-rich repeat protein kinase family protein	-0.24078	-3.63606	-4.45548
Potri.003G166100	Signaling	ATSERK2,SERK2	somatic embryogenesis receptor-like kinase 2	0.224019	1.440592	2.784091
Potri.001G061700	Signaling	ATSERK1,SERK1	somatic embryogenesis receptor-like kinase 1	4.451782	0.387044	4.676918
Potri.004G108200	Signaling	NIK1	NSP-interacting kinase 1	-0.12243	-2.33661	-4.32922
Potri.004G066300	Signaling	RKL1	receptor-like kinase 1	-0.18911	-1.22133	-2.79907
Potri.010G058200	Signaling	0	Leucine-rich repeat protein kinase family protein	-0.47721	-0.66562	-4.06055
Potri.006G228400	Signaling	0	leucine-rich repeat transmembrane protein kinase family protein	-1.39841	-0.28885	-4.15263
Potri.010G120100	Signaling	0	Leucine-rich repeat protein kinase family protein	-0.31847	-1.15259	-2.06039

Potri.001G209700	Signaling	0	Leucine-rich repeat protein kinase family protein	-2.22166	-2.28518	-1.60408
Potri.006G139700	Signaling	0	Leucine-rich repeat protein kinase family protein	-0.26704	-0.13918	4.622524
Potri.001G465800	Signaling	0	Leucine-rich receptor-like protein kinase family protein	-0.9964	-0.4765	-2.51395
Potri.001G095200	Signaling	0	Leucine-rich repeat protein kinase family protein	-0.27857	-1.38206	-2.59982
Potri.001G300700	Signaling	0	Leucine-rich repeat protein kinase family protein	-1.17332	-0.35239	-2.57592
Potri.007G046900	Signaling	0	Leucine-rich repeat protein kinase family protein	-0.4023	-1.99033	-3.41175
Potri.012G128700	Signaling	0	Leucine-rich repeat protein kinase family protein	-0.39001	-1.88085	-3.0431
Potri.011G045600	Signaling	SRF3	STRUBBELIG-receptor family 3	-0.82625	-1.19269	-2.96477
Potri.004G005400	Signaling	SRF8	STRUBBELIG-receptor family 8	-0.18959	-1.74676	-3.16928
Potri.004G005300	Signaling	SRF8	STRUBBELIG-receptor family 8	-0.20858	-1.73763	-2.90024
Potri.010G185300	Signaling	MRH1	Leucine-rich repeat protein kinase family protein	-0.59372	-2.07482	-1.97843
Potri.001G333300	Signaling	0	Leucine-rich repeat protein kinase family protein	-0.17676	-1.24396	-3.11541
Potri.002G233600	Signaling	0	Leucine-rich receptor-like protein kinase family protein	-0.23015	-0.60514	-3.30114
Potri.004G058500	Signaling	MEE62	Leucine-rich repeat protein kinase family protein	1.929073	1.341789	3.460771
Potri.019G131800	Signaling	0	Leucine-rich repeat protein kinase family protein	3.032873	3.797054	7.302612
Potri.006G114400	Signaling	0	Leucine-rich repeat protein kinase family protein	0.15618	-3.18405	-6.50613
Potri.016G140	Signaling	0	Leucine-rich repeat protein kinase family protein	-0.00523	1.868041	5.313092

200						
Potri.T008900	Signaling	0	Leucine-rich repeat transmembrane protein kinase	0.554176	0.34238	2.593156
Potri.001G386000	Signaling	0	Leucine-rich repeat transmembrane protein kinase	3.101856	5.468771	8.01074
Potri.011G112000	Signaling	0	Protein kinase superfamily protein	1.577078	2.428809	5.525831
Potri.011G075300	Signaling	0	Leucine-rich repeat transmembrane protein kinase	-10.7596	3.866001	7.842211
Potri.011G073500	Signaling	0	Leucine-rich repeat transmembrane protein kinase	0.603065	-0.05238	2.269636
Potri.T008000	Signaling	0	Leucine-rich repeat transmembrane protein kinase	1.050764	0.904906	4.53086
Potri.004G063500	Signaling	RKF1	receptor-like kinase in flowers 1	0.153292	0.811287	4.464619
Potri.016G011200	Signaling	0	Leucine-rich repeat transmembrane protein kinase	-0.33481	1.352758	2.108007
Potri.003G025600	Signaling	0	Leucine-rich repeat transmembrane protein kinase	2.374108	0.743292	2.317374
Potri.006G255100	Signaling	0	Leucine-rich repeat transmembrane protein kinase	2.107885	3.618216	6.090145
Potri.004G040200	Signaling	0	Protein kinase superfamily protein	0.869743	0.827243	2.209515
Potri.001G384700	Signaling	0	Leucine-rich repeat transmembrane protein kinase	1.368576	2.329433	3.548338
Potri.004G084000	Signaling	TMK1	transmembrane kinase 1	-1.85785	-2.87733	-4.5386
Potri.016G070500	Signaling	0	Leucine-rich repeat protein kinase family protein	-0.50478	-1.84339	-2.72921
Potri.013G114200	Signaling	0	leucine-rich repeat transmembrane protein kinase family protein	1.238096	2.172757	3.166725
Potri.011G169600	Signaling	BRL1	BRI1 like	-0.29864	0.638101	2.004477
Potri.001G310700	Signaling	ATPSKR1,PSKR1	phytosulfokin receptor 1	-0.05735	1.037236	1.982059

Potri.006G051700	Signaling	0	Leucine-rich repeat protein kinase family protein	-0.5237	-1.60489	-2.59893
Potri.001G349900	Signaling	0	Leucine-rich repeat protein kinase family protein	0.659382	1.154965	3.016728
Potri.015G141200	Signaling	EMS1,EXS	Leucine-rich repeat transmembrane protein kinase	-0.55422	-0.36492	-2.63685
Potri.010G177900	Signaling	BIR1	BAK1-interacting receptor-like kinase 1	0.608878	2.084225	3.631972
Potri.013G048800	Signaling	0	Leucine-rich receptor-like protein kinase family protein	-0.47386	-2.86264	-5.13765
Potri.009G081800	Signaling	LRR XI-23,RLK7	Leucine-rich receptor-like protein kinase family protein	2.164425	1.215733	3.375961
Potri.011G058100	Signaling	HSL1	HAESA-like 1	0.438754	1.025647	3.137137
Potri.011G169600	Signaling	BRL1	BRI1 like	-0.29864	0.638101	2.004477
Potri.005G241500	Signaling	ATCLV1,CLV1,FAS3,FLO5	Leucine-rich receptor-like protein kinase family protein	-0.13771	-1.57674	-2.23011
Potri.012G090500	Signaling	EVR,SOBIR1	Leucine-rich repeat protein kinase family protein	1.429592	4.369484	5.435925
Potri.003G175700	Signaling	0	Leucine-rich repeat receptor-like protein kinase family protein	-1.95955	-1.96775	-3.96414
Potri.006G235500	Signaling	0	Protein kinase family protein with leucine-rich repeat domain	1.231332	2.631076	4.384953
Potri.002G070900	Signaling	0	Leucine-rich repeat transmembrane protein kinase family protein	-0.9232	-2.00117	-4.63756
Potri.001G467300	Signaling	0	Leucine-rich receptor-like protein kinase family protein	-0.52884	-1.26437	-3.77433
Potri.001G126100	Signaling	PXY	Leucine-rich repeat protein kinase family protein	-0.29819	-1.41463	-2.72261
Potri.019G025500	Signaling	0	Leucine-rich repeat protein kinase family protein	-1.12846	1.394383	3.068857
Potri.007G009	Signaling	BAM1	Leucine-rich receptor-like protein kinase family protein	-0.1916	-1.29677	-3.32983

200						
Potri.003G149900	Signaling	0	Leucine-rich repeat protein kinase family protein	-1.50692	3.481758	4.830363
Potri.003G108200	Signaling	0	Leucine-rich repeat receptor-like protein kinase family protein	2.940223	1.362726	5.348294
Potri.017G150800	Signaling	EFR	EF-TU receptor	2.968041	4.004376	1.674857
Potri.006G220100	Signaling	ER,QRP1	Leucine-rich receptor-like protein kinase family protein	-0.54446	-0.59053	-2.75236
Potri.012G071100	Signaling	0	Leucine-rich repeat protein kinase family protein	-0.32099	-2.34826	-3.49915
Potri.004G155100	Signaling	0	Leucine-rich repeat protein kinase family protein	0.407463	0.751933	1.994062
Potri.004G096800	Signaling	0	receptor serine/threonine kinase, putative	-1.19866	2.319759	4.028871
Potri.004G014700	Signaling	PR5K	PR5-like receptor kinase	1.901797	3.60983	4.10659
Potri.018G091000	Signaling	0	Protein kinase superfamily protein	2.567828	4.545715	7.586453
Potri.010G043700	Signaling	CRK2	cysteine-rich RLK (RECEPTOR-like protein kinase) 2	0.675609	2.066251	2.792612
Potri.010G043900	Signaling	CRK3	cysteine-rich RLK (RECEPTOR-like protein kinase) 3	2.486613	0.83408	3.590767
Potri.004G023500	Signaling	CRK25	cysteine-rich RLK (RECEPTOR-like protein kinase) 25	5.255805	4.544362	6.787614
Potri.018G111700	Signaling	CRK34	cysteine-rich RLK (RECEPTOR-like protein kinase) 34	1.146192	4.369024	7.618932
Potri.011G028600	Signaling	CRK29	cysteine-rich RLK (RECEPTOR-like protein kinase) 29	2.116303	3.335435	5.506361
Potri.004G023800	Signaling	CRK10,RLK4	cysteine-rich RLK (RECEPTOR-like protein kinase) 10	4.135064	3.87193	5.916356
Potri.011G029100	Signaling	CRK14	cysteine-rich RLK (RECEPTOR-like protein kinase) 14	0.761854	0.939744	3.481178

Potri.011G029400	Signaling	CRK26	cysteine-rich RLK (RECEPTOR-like protein kinase) 26	4.285199	3.198641	4.473394
Potri.005G036600	Signaling	0	Protein kinase superfamily protein	-0.45396	-4.14488	-5.00763
Potri.009G035500	Signaling	0	Concanavalin A-like lectin protein kinase family protein	-1.31798	0.621708	3.285993
Potri.003G196600	Signaling	0	Concanavalin A-like lectin protein kinase family protein	0.380119	1.094207	2.192716
Potri.001G455500	Signaling	0	Concanavalin A-like lectin protein kinase family protein	6.059453	5.536089	7.891428
Potri.011G093700	Signaling	0	Concanavalin A-like lectin protein kinase family protein	0.604233	-1.04995	-2.26205
Potri.012G054700	Signaling	0	Protein kinase superfamily protein	2.644838	3.554455	5.08942
Potri.015G121900	Signaling	0	Protein kinase superfamily protein	0.663916	2.029859	3.105991
Potri.010G121100	Signaling	0	Protein kinase superfamily protein	3.134725	1.934565	2.829805
Potri.004G096500	Signaling	0	Protein kinase superfamily protein	-0.05201	2.596148	3.857506
Potri.017G009600	Signaling	0	Protein kinase superfamily protein	2.744613	3.657244	8.109579
Potri.012G002800	Signaling	0	Protein kinase superfamily protein	-0.07192	2.046495	3.709502
Potri.007G125000	Signaling	0	Protein kinase superfamily protein	0.021137	1.085743	4.27876
Potri.004G097100	Signaling	0	Protein kinase family protein	0.44263	4.30159	5.283919
Potri.012G054500	Signaling	0	Protein kinase superfamily protein	2.301567	2.451301	2.466831
Potri.005G128200	Signaling	0	protein kinase family protein / peptidoglycan-binding LysM domain-containing protein	2.307198	2.007926	4.73359
Potri.014G156	Signaling	CERK1,LYSM	chitin elicitor receptor kinase 1	2.814817	2.348796	5.478099

400		RLK1				
Potri.007G027000	Signaling	PERK4	roline-rich extensin-like receptor kinase 4	-0.68858	2.146272	3.239352
Potri.014G038300	Signaling	0	Protein kinase protein with adenine nucleotide alpha hydrolases-like domain	-0.58105	-1.51288	-2.4172
Potri.005G181800	Signaling	0	Protein kinase superfamily protein	4.700856	2.723294	6.289634
Potri.014G136400	Signaling	RKF3	receptor-like kinase in in flowers 3	-0.11814	1.014567	2.669859
Potri.011G037700	Signaling	CBRLK1,RKS2,SD1-13	S-domain-1 13	1.703182	1.651141	4.855289
Potri.010G103300	Signaling	0	lectin protein kinase family protein	1.056492	1.99345	3.834256
Potri.011G036500	Signaling	ARK1,RK1	receptor kinase 1	0.267941	0.419569	3.433745
Potri.001G412400	Signaling	ARK3,RK3	receptor kinase 3	3.141919	2.461132	5.716241
Potri.001G441400	Signaling	B120	S-locus lectin protein kinase family protein	4.565838	2.622514	6.039261
Potri.001G409400	Signaling	0	S-locus lectin protein kinase family protein	3.539698	1.797557	3.875632
Potri.018G148300	Signaling	WAKL1	wall associated kinase-like 1	1.480525	2.841679	3.948202
Potri.001G039400	Signaling	WAKL2	wall associated kinase-like 2	-0.14089	-0.61352	3.360994
Potri.T166400	Signaling	0	Wall-associated kinase family protein	0.237314	2.017592	6.302945
Potri.003G185700	Signaling	0	Wall-associated kinase family protein	3.432128	5.721232	8.016522
Potri.T080600	Signaling	0	Wall-associated kinase family protein	14.18146	16.46345	21.1725
Potri.002G075900	Signaling	WAK2	wall-associated kinase 2	2.359393	1.883793	4.183361
Potri.001G040000	Signaling	0	Wall-associated kinase family protein	0.283228	0.430471	3.504956

Potri.001G038300	Signaling	RFO1,WAKL22	Wall-associated kinase family protein	-0.37295	0.42563	4.191064
Potri.005G130900	Signaling	0	Protein kinase superfamily protein	-0.25848	1.085858	2.150373
Potri.008G059900	Signaling	0	Protein kinase family protein	3.576823	4.858814	7.175894
Potri.001G260800	Signaling	0	Protein kinase superfamily protein	-0.0716	1.013013	3.442756
Potri.008G068700	Signaling	ATCRR3,CCR3	CRINKLY4 related 3	1.967369	0.598459	1.840395
Potri.013G103300	Signaling	CCR4	CRINKLY4 related 4	3.238893	3.329214	5.556666
Potri.005G043700	Signaling	0	Leucine-rich repeat transmembrane protein kinase protein	4.744346	2.439977	3.782688
Potri.003G027200	Signaling	AtRLP1,RLP1	receptor like protein 1	-1.77673	1.04291	5.417912
Potri.003G029200	Signaling	AtRLP1,RLP1	receptor like protein 1	1.353692	1.659043	5.388969
Potri.019G109400	Signaling	0	Leucine-rich repeat protein kinase family protein	-0.31532	0.374661	2.555248
Potri.004G212600	Signaling	NCRK	Protein kinase superfamily protein	-0.09572	-0.97424	-2.48617
Potri.007G120400	Signaling	0	Receptor-like protein kinase-related family protein	2.396731	0.893864	3.617548
Potri.006G103200	Signaling	LECRKA4.2	lectin receptor kinase a4.1	2.724106	1.481359	3.203919
Potri.016G129500	Signaling	0	Nuclear transport factor 2 (NTF2) family protein	0.576821	-1.08004	-5.10404
Potri.016G051600	Signaling	0	Leucine-rich repeat receptor-like protein kinase family protein	-0.37582	-0.37252	-2.0217
Potri.001G066700	Signaling	CRN,SOL2	Protein kinase superfamily protein	-1.40055	-0.93311	-2.18915
Potri.017G130	Signaling	PDLP7	plasmodesmata-located protein 7	-0.05859	0.502933	-3.26436

800						
Potri.011G058300	Signaling	0	Protein kinase superfamily protein	2.638429	1.471385	3.305567
Potri.002G182500	Signaling	0	Calcium-binding endonuclease/exonuclease/phosphatase family	-1.06931	-2.06529	-1.97032
Potri.019G055200	Signaling	AtCRT3,CRT3,EBS2,PSL1	calreticulin 3	2.06998	2.98549	2.945397
Potri.013G029100	Signaling	BON3	Calcium-dependent phospholipid-binding Copine family protein	14.43992	15.49595	21.72924
Potri.012G048200	Signaling	0	Calcium-binding EF-hand family protein	0.361719	0.946216	2.113436
Potri.002G077300	Signaling	0	Calcium-binding EF-hand family protein	2.167377	3.621254	4.649417
Potri.014G070700	Signaling	0	EF hand calcium-binding protein family	-0.48375	-1.38658	-2.41066
Potri.001G375300	Signaling	0	sodium/calcium exchanger family protein / calcium-binding EF hand family protein	-0.58363	1.430769	4.46051
Potri.003G042700	Signaling	iqd8	IQ-domain 8	-1.04399	-1.95248	-5.57556
Potri.012G054900	Signaling	0	Calmodulin binding protein-like	2.331178	6.849011	9.12348
Potri.017G079600	Signaling	IQD31	IQ-domain 31	-0.01425	-0.80267	-2.02252
Potri.012G071700	Signaling	ATCPK30,CDPK1A,CPK30	calcium-dependent protein kinase 30	1.379744	2.280545	4.141845
Potri.005G259900	Signaling	CML38	calmodulin-like 38	0.23677	-0.14118	2.158193
Potri.004G143500	Signaling	0	Protein of unknown function (DUF1645)	2.775789	2.83628	4.283878
Potri.009G168600	Signaling	ATCDPK3,ATCPK6,CPK6	Calcium-dependent protein kinase family protein	0.071107	0.851648	2.268719
Potri.006G226600	Signaling	IQD6	IQ-domain 6	-0.29017	-1.20035	-2.46876

Potri.004G202200	Signaling	0	Calcium-binding EF-hand family protein	1.552522	1.116501	4.064206
Potri.007G127000	Signaling	CPK24	calcium-dependent protein kinase 24	0.947945	2.582718	3.350007
Potri.001G020600	Signaling	ACA4	autoinhibited Ca(2+)-ATPase, isoform 4	3.821955	1.064471	5.30865
Potri.002G218300	Signaling	0	Calcium-binding EF-hand family protein	-1.70468	2.99962	-3.60375
Potri.002G174900	Signaling	0	Calcium-binding EF-hand family protein	0.56527	4.330009	5.575645
Potri.006G047300	Signaling	0	Calcium-binding EF-hand family protein	2.507999	2.316623	3.40451
Potri.016G066700	Signaling	ATCPK2,CPK2	calmodulin-domain protein kinase cdpk isoform 2	2.396488	1.48325	3.927311
Potri.003G218800	Signaling	0	calmodulin-binding family protein	0.528557	1.025084	2.992628
Potri.001G375700	Signaling	IQD10	IQ-domain 10	-0.28877	-0.04066	-4.23019
Potri.001G190500	Signaling	IQD26	IQ-domain 26	-1.05292	-0.62368	-2.22768
Potri.008G159100	Signaling	0	ATPase E1-E2 type family protein / haloacid dehalogenase-like hydrolase family protein	-0.23732	0.387614	3.942813
Potri.005G215700	Signaling	CML11	calmodulin-like 11	7.833097	5.353802	9.486613
Potri.010G132800	Signaling	0	Calcium-binding EF-hand family protein	1.265895	0.982937	2.600714
Potri.015G012500	Signaling	iqd21	IQ-domain 21	-0.42215	-0.07302	-3.34419
Potri.012G016200	Signaling	iqd21	IQ-domain 21	-0.95085	-1.16008	-2.99956
Potri.007G031900	Signaling	CML41	calmodulin-like 41	-0.02523	1.410323	2.738791
Potri.016G034	Signaling	0	IQ calmodulin-binding motif family protein	-0.67271	-1.19317	-3.92441

100						
Potri.006G052900	Signaling	ATCPK32,CDPK32,CPK32	calcium-dependent protein kinase 32	1.583901	0.862071	4.216365
Potri.006G197500	Signaling	0	calmodulin-binding family protein	0.981215	3.867398	3.711647
Potri.002G047500	Signaling	0	ATPase E1-E2 type family protein / haloacid dehalogenase-like hydrolase family protein	-0.85914	1.946781	2.863342
Potri.T056000	Signaling	0	Calcium-binding EF-hand family protein	1.5676	4.065166	6.91748
Potri.006G112500	Signaling	CML42	calmodulin like 42	2.737905	2.786043	4.865821
Potri.001G108800	Signaling	IQD22	IQ-domain 22	2.664127	1.110593	0.942506
Potri.013G010700	Signaling	0	Calmodulin-binding protein	1.853844	3.973045	6.759171
Potri.001G411800	Signaling	0	Calcium-binding EF-hand family protein	1.958324	2.671075	3.214475
Potri.018G139800	Signaling	ACA10,ATACA10,CIF1	autoinhibited Ca(2+)-ATPase 10	-1.28328	-0.13879	-2.89014
Potri.T109200	Signaling	0	calcium-binding EF hand family protein	11.44546	14.80125	16.63821
Potri.018G061900	Signaling	EDA39	calmodulin-binding family protein	5.213842	5.457901	6.632296
Potri.006G046000	Signaling	IQD12	IQ-domain 12	-1.16993	-1.33896	-3.38129
Potri.008G038300	Signaling	ATSYTC,NTMC2T1.3,NTMC2TYPE1.3,SYT3,SYTC	Calcium-dependent lipid-binding (CaLB domain) family protein	0.429074	0.157328	2.245738
Potri.006G200600	Signaling	ATCPK1,CPK1	calcium dependent protein kinase 1	4.792146	5.351198	6.805756
Potri.001G021000	Signaling	IQD11	IQ-domain 11	0.058028	-0.35293	-2.07049
Potri.012G015100	Signaling	ATSOS3,CBL4,SOS3	Calcium-binding EF-hand family protein	3.202485	4.205824	7.31749

Potri.012G023300	Signaling	0	Calcium-dependent protein kinase (CDPK) family protein	0.398628	0.34416	3.165009
Potri.013G040500	Signaling	0	calcium-binding EF hand family protein	1.905958	1.261385	3.908103
Potri.014G161000	Signaling	iqd33	IQ-domain 33	-0.70948	-0.73877	-2.93581
Potri.017G126200	Signaling	CML24,TCH2	EF hand calcium-binding protein family	1.036349	0.591839	2.326419
Potri.004G122900	Signaling	0	Calcium-binding EF-hand family protein	4.365027	5.809149	7.091003
Potri.002G001400	Signaling	CML37,CML39	calmodulin like 37	2.868131	1.219498	3.69986
Potri.008G103900	Signaling	ATCP1,CP1	Ca2+-binding protein 1	1.587805	2.98295	3.636665
Potri.012G007600	Signaling	0	Calcium-binding endonuclease/exonuclease/phosphatase family	1.22298	2.285423	1.618812
Potri.013G151000	Signaling	PBP1	pinoid-binding protein 1	0.48093	3.560504	6.059189
Potri.005G020200	Signaling	0	Calmodulin-binding protein	1.202776	4.740873	7.561696
Potri.015G109200	Signaling	ATCNX1,CNX1	calnexin 1	1.499893	1.674854	2.261257
Potri.012G077000	Signaling	0	Calmodulin binding protein-like	-0.18957	0.305269	-2.57843
Potri.005G113600	Signaling	CPK28	calcium-dependent protein kinase 28	0.916589	1.182877	2.140013
Potri.001G027900	Signaling	0	Phosphatidylinositol-4-phosphate 5-kinase family protein	-0.62726	-0.59169	-4.62991
Potri.005G139400	Signaling	0	PLC-like phosphodiesterases superfamily protein	1.251374	3.238272	7.013793
Potri.001G252100	Signaling	ATPLC4,PLC4	phosphatidylinositol-speciwc phospholipase C4	1.973195	1.118815	3.857958
Potri.014G102	Signaling	ATRAB-A3,A	RAB GTPase homolog A3	-1.23815	-1.68011	-2.27295

200		TRABA3,RAB A3				
Potri.008G061 300	Signaling	ATRAB-A2B, ATRABA2B,R AB-A2B,RAB A2b	RAB GTPase homolog A2B	-1.23431	-2.16989	-3.84577
Potri.015G038 200	Signaling	0	zinc finger (Ran-binding) family protein	1.095141	2.150148	-0.67529
Potri.015G039 700	Signaling	AtRABA6a,R ABA6a	RAB GTPase homolog A6A	-0.12569	-0.56279	2.108249
Potri.009G109 400	Signaling	ATXLG1,XLG 1	extra-large G-protein 1	0.511465	0.827031	2.879519
Potri.002G176 800	Signaling	0	Rho GTPase activating protein with PAK-box/P21-Rho-binding domain	-0.99593	-1.10659	-2.66087
Potri.002G234 600	Signaling	SCN1	Immunoglobulin E-set superfamily protein	-0.30725	-1.09898	-2.51732
Potri.017G027 900	Signaling	PRA1.F4	prenylated RAB acceptor 1.F4	2.339239	5.451614	6.872269
Potri.002G081 500	Signaling	AGG1,ATAG G1,GG1	Ggamma-subunit 1	-1.0237	-1.26297	-2.50056
Potri.T106300	Signaling	0	Rho GTPase activating protein with PAK-box/P21-Rho-binding domain	-0.67461	-1.28837	-2.38127
Potri.009G115 300	Signaling	AGB1,ATAGB 1,ELK4	GTP binding protein beta 1	0.91218	0.550107	2.261825
Potri.009G140 100	Signaling	ATROPGEF1, ROPGEF1	rho guanyl-nucleotide exchange factor 1	-0.41393	-0.44455	-2.79989
Potri.006G092 600	Signaling	ATROPGEF7, ROPGEF7	RHO guanyl-nucleotide exchange factor 7	-0.67079	-1.21572	-2.42919
Potri.005G075 300	Signaling	AtRABH1e,R ABH1e	RAB GTPase homolog H1E	2.430168	2.230262	6.686446
Potri.006G251 300	Signaling	0	Ran BP2/NZF zinc finger-like superfamily protein	-0.18625	-0.38388	-2.82711
Potri.011G061 500	Signaling	ARAC2,ATRA C2,ATROP7,	RAC-like 2	-0.30845	-0.56706	-3.93957

		RAC2,ROP7				
Potri.015G070900	Signaling	0	Transducin/WD40 repeat-like superfamily protein	0.467698	3.015505	3.352724
Potri.011G136300	Signaling	ATSGP1,SGP1	Ras-related small GTP-binding family protein	1.63193	1.871936	4.216612
Potri.014G155000	Signaling	MAPKKK18	mitogen-activated protein kinase kinase kinase 18	1.403221	0.07734	2.541587
Potri.015G030700	Signaling	ATMKK9,MKK9	MAP kinase kinase 9	0.414795	1.096224	2.672163
Potri.008G130000	Signaling	ATMPK15,MPK15	MAP kinase 15	0.150825	-0.36329	-2.18221
Potri.007G020100	Signaling	ATMPK7,MPK7	MAP kinase 7	0.375724	0.827206	2.38134
Potri.009G066100	Signaling	ATMAPK3,ATMPK3,MPK3	mitogen-activated protein kinase 3	0.840259	1.268059	2.827765
Potri.016G134600	Signaling	ATWNK5,WNK5,ZIK1	with no lysine (K) kinase 5	-0.19452	-0.85263	-3.57592
Potri.019G128600	Signaling	WNK4,ZIK2	with no lysine (K) kinase 4	1.874732	3.910477	6.109323
Potri.002G129100	Signaling	MAPKKK5	mitogen-activated protein kinase kinase kinase 5	2.270076	2.651658	4.760434
Potri.014G131800	Signaling	ATRALF1,RALF1,RALFL1	rapid alkalinization factor 1	-1.09188	0.044004	2.860199
Potri.017G096500	Signaling	RALFL27	ralf-like 27	-0.6164	-1.15053	-2.19566
Potri.008G208200	Signaling	RALFL33	ralf-like 33	0.258782	-1.33287	-2.21875
Potri.007G044700	Signaling	RALFL34	ralf-like 34	-1.25281	-2.00805	-5.56484
Potri.010G223400	Signaling	0	Pleckstrin homology (PH) and lipid-binding START domains-containing protein	-0.53695	-0.12293	-2.21287
Potri.002G209700	Signaling	0	Phototropic-responsive NPH3 family protein	-0.05354	-2.10421	-2.57767

Potri.014G111400	Signaling	PAP3,PIF3,POC1	phytochrome interacting factor 3	-1.55321	-1.03055	-5.03986
Potri.010G046800	Signaling	0	Phototropic-responsive NPH3 family protein	0.111007	-0.71768	-2.74342
Potri.013G159000	Signaling	RPT2	Phototropic-responsive NPH3 family protein	-0.5857	-0.61647	-2.20677
Potri.002G055400	Signaling	PIF4,SRL2	phytochrome interacting factor 4	-0.32799	-1.29759	-2.14566
Potri.003G058800	Signaling	0	Phototropic-responsive NPH3 family protein	-0.24543	-0.9473	-3.12935
Potri.004G150300	Signaling	0	NAD(P)-binding Rossmann-fold superfamily protein	-1.23184	-2.64461	-3.57815
Potri.010G170900	Signaling	0	Phototropic-responsive NPH3 family protein	-0.79661	-0.71649	-3.0759
Potri.005G210200	Signaling	TIC	time for coffee	0.846177	0.47118	2.232055
Potri.008G157800	Signaling	ELIP,ELIP1	Chlorophyll A-B binding family protein	3.040182	4.757838	4.743994
Potri.001G066400	Signaling	CCL	CCR-like	-0.70002	-1.43711	-2.94162
Potri.004G189800	Signaling	0	Phototropic-responsive NPH3 family protein	-0.80534	-1.18302	-2.68702
Potri.016G009700	Signaling	SCL13	SCARECROW-like 13	0.823483	1.139135	2.036218
Potri.005G090000	Signaling	PKS4	phytochrome kinase substrate 4	-0.42969	-1.85718	-5.54207
Potri.009G157600	Signaling	LSH1	Protein of unknown function (DUF640)	-0.50459	-0.70462	-8.60495
Potri.007G112600	Signaling	JK218,NPH3,RPT3	Phototropic-responsive NPH3 family protein	-0.98708	-1.36526	-2.67147
Potri.008G091900	WRKY	WRKY4	WRKY DNA-binding protein 4	0.097314	3.333402	1.16073
Potri.004G060	WRKY	ATWRKY65,	WRKY DNA-binding protein 65	1.155466	2.299749	3.986507

400		WRKY65				
Potri.001G460600	WRKY	AR411,ATWRKY14,WRKY14	WRKY DNA-binding protein 14	0	12.17985	17.5552
Potri.002G228400	WRKY	ATWRKY6,WRKY6	WRKY family transcription factor	2.327125	1.250051	3.535641
Potri.018G019700	WRKY	ATWRKY40, WRKY40	WRKY DNA-binding protein 40	15.99818	17.83839	21.51459
Potri.006G105300	WRKY	ATWRKY33, WRKY33	WRKY DNA-binding protein 33	2.99434	1.568354	2.859671
Potri.002G193000	WRKY	ATWRKY23, WRKY23	WRKY DNA-binding protein 23	1.346026	0.040173	2.416299
Potri.005G055300	WRKY	ATWRKY39, WRKY39	WRKY DNA-binding protein 39	-0.15602	-1.28456	-1.93901
Potri.006G109100	WRKY	ATWRKY70, WRKY70	WRKY DNA-binding protein 70	-2.23127	2.438767	3.415496
Potri.003G132700	WRKY	AtWRKY22, WRKY22	WRKY family transcription factor	2.269079	0.807857	3.243708
Potri.002G186600	WRKY	AtWRKY47, WRKY47	WRKY family transcription factor	1.098917	1.800296	3.343828
Potri.001G092900	WRKY	AtWRKY41, WRKY41	WRKY family transcription factor	3.441268	1.271897	2.524416
Potri.001G352400	WRKY	ATWRKY28, WRKY28	WRKY DNA-binding protein 28	4.894988	0.791532	3.732837
Potri.002G168700	WRKY	ATWRKY53, WRKY53	WRKY family transcription factor	0.927953	2.187233	2.496854
Potri.014G024200	WRKY	ATWRKY7,WRKY7	WRKY DNA-binding protein 7	-0.85221	-2.33739	-1.76253
Potri.006G263600	WRKY	ATWRKY18, WRKY18	WRKY DNA-binding protein 18	2.263797	3.187428	4.19584
Potri.019G123500	WRKY	WRKY26	WRKY DNA-binding protein 26	0.262027	0.388747	2.471386
Potri.001G058	WRKY	ATWRKY75,	WRKY DNA-binding protein 75	-0.62309	3.00429	7.360923

800		WRKY75				
Potri.015G064100	WRKY	ATWRKY72, WRKY72	WRKY DNA-binding protein 72	5.942075	3.835499	7.138715
Potri.012G031700	WRKY	ATWRKY30, WRKY30	WRKY DNA-binding protein 30	4.76323	4.277651	6.844897
Potri.006G224100	WRKY	ATWRKY50, WRKY50	WRKY DNA-binding protein 50	2.672694	5.564347	5.732818
Potri.008G103300	WRKY	ATWRKY48, WRKY48	WRKY DNA-binding protein 48	1.544191	1.966301	4.320825
Potri.001G099000	WRKY	ATWRKY27, WRKY27	WRKY DNA-binding protein 27	3.255118	1.548012	6.440776
Potri.005G085200	WRKY	ATWRKY51, WRKY51	WRKY DNA-binding protein 51	-0.36768	6.215261	7.776216

-2 0 2

Supplementary Table S6: GO enrichment analysis of genes selected according to co-expression analysis (Figure 6). GO terms in "Up-Trends" were enriched by genes involved in cluster 16, cluster 22, and cluster 25 of Aig in Figure 6a. GO terms in "Down-Trends" were enriched by genes involved in cluster 0, cluster 3, and cluster 9 of Aig in Figure 6a. GO terms in "Up to down-Trends" were enriched by genes involved incluster 12, cluster 14, cluster 20, and cluster 23 of Aig in Figure 6a.

Up-Trends					Down-Trends					Up to down-Trends				
GO terms	Term type	Term	Query item	FDR	GO terms	Term type	Term	Query item	FDR	GO terms	Term type	Term	Query item	FDR
GO:0006950	P	response to stress	37	0.015	GO:0010467	P	gene expression	107	0.035	GO:0051179	P	localization	96	0.013
GO:0009605	P	response to external stimulus	11	0.000022	GO:0006412	P	translation	46	0.000039	GO:0006810	P	transport	95	0.013

GO:0009611	P	response to wounding	7	0.00056
GO:0016787	F	hydrolase activity	110	0.038
GO:0004866	F	endopeptidase inhibitor activity	14	2.9E-06
GO:0042578	F	phosphoric ester hydrolase activity	11	0.018
GO:0016791	F	phosphatase activity	8	0.018

GO:0051641	P	cellular localization	28	0.000039
GO:0046907	P	intracellular transport	26	0.000017
GO:0015031	P	protein transport	26	0.000039
GO:0033036	P	macromolecule localization	26	0.00022
GO:0006396	P	RNA processing	15	0.04
GO:0048193	P	Golgi vesicle transport	7	0.0038
GO:0009100	P	glycoprotein metabolic process	7	0.044
GO:0006486	P	protein amino acid glycosylation	7	0.044
GO:0070085	P	glycosylation	7	0.044
GO:0015977	P	carbon fixation	5	0.005
GO:0071704	P	organic substance metabolic process	5	0.005
GO:0006284	P	base-excision repair	5	0.044
GO:0010033	P	response to organic substance	5	0.044
GO:0009719	P	response to endogenous stimulus	5	0.044
GO:00097	P	response to hormone	5	0.044

GO:0044283	P	small molecule biosynthetic process	26	0.013
GO:0046394	P	carboxylic acid biosynthetic process	21	0.013
GO:0030259	P	lipid glycosylation	5	0.025
GO:0000096	P	sulfur amino acid metabolic process	5	0.041
GO:0003824	F	catalytic activity	460	0.0038
GO:0016765	F	transferase activity	11	0.0045

25		stimulus		
GO:0005488	F	binding	449	0.0001
GO:0005515	F	protein binding	153	0.04
GO:0003676	F	nucleic acid binding	152	4.8E-06
GO:0016817	F	hydrolase activity, acting on acid anhydrides	55	0.00011
GO:0008270	F	zinc ion binding	51	0.000043
GO:0005198	F	structural molecule activity	43	3.9E-06
GO:0003735	F	structural constituent of ribosome	39	4.8E-06
GO:0016887	F	ATPase activity	38	0.000011
GO:0003723	F	RNA binding	26	0.0025
GO:0004386	F	helicase activity	25	0.000063
GO:0008026	F	ATP-dependent helicase activity	21	2.4E-06
GO:0008565	F	protein transporter activity	13	0.000043
GO:0003924	F	GTPase activity	13	0.026
GO:0016830	F	carbon-carbon lyase activity	9	0.024
GO:0016831	F	carboxy-lyase activity	8	0.0053
GO:0004576	F	oligosaccharyl transferase activity	5	0.0036

GO:0044464	C	cell part	210	0.024
GO:0005623	C	cell	210	0.024
GO:0005622	C	intracellular	157	1.8E-06
GO:0044424	C	intracellular part	130	4.7E-06
GO:0043226	C	organelle	109	6E-07
GO:0032991	C	macromolecular complex	86	4E-08
GO:0005737	C	cytoplasm	83	6E-07
GO:0044444	C	cytoplasmic part	70	1.1E-07
GO:0043231	C	intracellular membrane-bounded organelle	68	0.00027
GO:0044422	C	organelle part	44	4.6E-06
GO:0043228	C	non-membrane-bounded organelle	43	0.000092
GO:0043234	C	protein complex	42	0.00084
GO:0005840	C	ribosome	39	1.7E-06
GO:0012505	C	endomembrane system	24	2.1E-08
GO:0031090	C	organelle membrane	18	0.00062
GO:0031982	C	vesicle	10	7.6E-06
GO:00057	C	Golgi apparatus	10	0.0002

94				1
GO:0046930	C	pore complex	9	8.5E-07
GO:0005643	C	nuclear pore	9	8.5E-07
GO:0012506	C	vesicle membrane	9	5.2E-06

Supplementary Table S6 (cont.): GO enrichment analysis of genes selected according to co-expression analysis. GO terms in "Up-Trends" were enriched by genes involved in cluster 16, cluster 22, and cluster 25 of Leu in Figure 6b. GO terms in "Down-Trends" were enriched by genes involved in cluster 0, cluster 3, and cluster 9 of Leu in Figure 6b. GO terms in "Up to down-Trends" were enriched by genes involved incluster 12, cluster 14, cluster 20, and cluster 23 of Leu in Figure 6b.

Up-regulation Trends					Down-regulation Trends					Up to down-regulation Trends				
GO terms	Term type	Term	Query item	FDR	GO terms	Term type	Term	Query item	FDR	GO terms	Term type	Term	Query item	FDR
GO:0043687	P	post-translational protein modification	262	2E-18	GO:0015979	P	photosynthesis	68	6.7E-24	GO:0009765	P	photosynthesis, light harvesting	8	0.014
GO:00	P	protein	247	2E-18	GO:00	P	photosynt	24	1.4E-13	GO:0006091	P	generation of	24	0.014

06468		amino acid phosphorylation			09765		hesis, light harvesting					precursor metabolites and energy		
GO:0006796	P	phosphate metabolic process	264	2E-18	GO:0008152	P	metabolic process	922	1.2E-06	GO:0008152	P	metabolic process	612	0.03
GO:0006793	P	phosphorus metabolic process	264	2E-18	GO:0006091	P	generation of precursor metabolites and energy	40	1.7E-06	GO:0009058	P	biosynthetic process	219	0.032
GO:0043412	P	macromolecule modification	274	4.2E-17	GO:0007017	P	microtubule-based process	29	0.00016	GO:0042254	P	ribosome biogenesis	10	0.042
GO:0044267	P	cellular protein metabolic process	298	0.000007	GO:0009416	P	response to light stimulus	15	0.00026	GO:0030001	P	metal ion transport	22	0.042
GO:0008152	P	metabolic process	881	7.9E-06	GO:0009628	P	response to abiotic stimulus	15	0.0011	GO:0006096	P	glycolysis	10	0.042
GO:0000003	P	reproduction	33	0.00015	GO:0007018	P	microtubule-based movement	19	0.0029	GO:0008610	P	lipid biosynthetic process	33	0.042
GO:0051704	P	multi-organism process	34	0.00015	GO:0044237	P	cellular metabolic process	581	0.0054	GO:0044249	P	cellular biosynthetic process	197	0.043
GO:0008037	P	cell recognition	33	0.00015	GO:0070271	P	protein complex biogenesis	20	0.013					

GO:0022414	P	reproductive process	33	0.00015	GO:0006461	P	protein complex assembly	20	0.013
GO:0032501	P	multicellular organismal process	35	0.00039	GO:0005976	P	polysaccharide metabolic process	32	0.013
GO:0044238	P	primary metabolic process	636	0.00039	GO:0044264	P	cellular polysaccharide metabolic process	28	0.015
GO:0019538	P	protein metabolic process	325	0.0004	GO:0006284	P	base-excision repair	9	0.019
GO:0044260	P	cellular macromolecule metabolic process	465	0.0004	GO:0046039	P	GTP metabolic process	10	0.019
GO:0044237	P	cellular metabolic process	569	0.00056	GO:0043933	P	macromolecular complex subunit organization	29	0.019
GO:0007154	P	cell communication	37	0.0043	GO:0005975	P	carbohydrate metabolic process	100	0.019
GO:0008652	P	cellular amino acid	21	0.0044	GO:0007049	P	cell cycle	13	0.032

		biosynthetic process									
GO:0009309	P	amine biosynthetic process	22	0.0064		GO:0000079	P	regulation of cyclin-dependent protein kinase activity	7	0.039	
GO:0016051	P	carbohydrate biosynthetic process	28	0.0072		GO:0009150	P	purine ribonucleotide metabolic process	20	0.04	
GO:0043170	P	macromolecule metabolic process	495	0.0073		GO:0034621	P	cellular macromolecular complex subunit organization	22	0.047	
GO:0044262	P	cellular carbohydrate metabolic process	54	0.0081		GO:0051536	F	iron-sulfur cluster binding	20	0.00078	
GO:0009987	P	cellular process	730	0.013		GO:0051540	F	metal cluster binding	20	0.00078	
GO:0046417	P	chorismate metabolic process	7	0.013		GO:0019104	F	DNA N-glycosylase activity	9	0.00078	

GO:0009073	P	aromatic amino acid family biosynthetic process	7	0.013	GO:0016799	F	hydrolase activity, hydrolyzing N-glycosyl compounds	9	0.0016
GO:0009611	P	response to wounding	7	0.015	GO:0003777	F	microtubule motor activity	19	0.0028
GO:0016567	P	protein ubiquitination	15	0.019	GO:0003774	F	motor activity	20	0.0048
GO:0023052	P	signaling	80	0.02	GO:0016987	F	sigma factor activity	6	0.0048
GO:0070647	P	protein modification by small protein conjugation or removal	15	0.021	GO:0005509	F	calcium ion binding	20	0.0074
GO:0016773	F	phosphotransferase activity, alcohol group as acceptor	265	1.3E-18	GO:0016620	F	oxidoreductase activity, acting on the aldehyde or oxo group of donors,	10	0.019

							NAD or NADP as acceptor		
GO:0016772	F	transferase activity, transferring phosphorus-containing groups	277	2.3E-15	GO:0016986	F	transcription initiation factor activity	6	0.035
GO:0016740	F	transferase activity	403	3.7E-14	GO:0016798	F	hydrolase activity, acting on glycosyl bonds	58	0.04
GO:0003824	F	catalytic activity	855	1.8E-09	GO:0019901	F	protein kinase binding	7	0.041
GO:0001883	F	purine nucleoside binding	359	1.2E-08	GO:0019899	F	enzyme binding	9	0.048
GO:0030554	F	adenyl nucleotide binding	359	1.2E-08	GO:0005507	F	copper ion binding	24	0.049
GO:0017076	F	purine nucleotide binding	378	8.1E-08	GO:0004190	F	aspartic-type endopeptidase activity	16	0.049
GO:0005529	F	sugar binding	44	9.7E-06	GO:0070001	F	aspartic-type peptidase	16	0.049

GO:0030246	F	carbohydrate binding	46	0.000034
GO:0003700	F	transcription factor activity	79	0.0024
GO:0043565	F	sequence-specific DNA binding	61	0.0026
GO:0016614	F	oxidoreductase activity, acting on CH-OH group of donors	46	0.0046
GO:0048037	F	cofactor binding	73	0.0047
GO:0016616	F	oxidoreductase activity, acting on the CH-OH group of donors, NAD or NADP as acceptor	44	0.0047
GO:0008762	F	UDP-N-acetylmu	16	0.0076

		activity		
GO:0009579	C	thylakoid	43	2.5E-14
GO:0034357	C	photosynthetic membrane	41	1.1E-13
GO:0019898	C	extrinsic to membrane	18	7.7E-10
GO:0016020	C	membrane	263	0.0024
GO:0032991	C	macromolecular complex	126	0.0096
GO:0044464	C	cell part	469	0.011
GO:0043234	C	protein complex	77	0.011

		ramate dehydrogenase activity							
GO:0003995	F	acyl-CoA dehydrogenase activity	5	0.018	GO:0005623	C	cell	469	0.011
GO:0004842	F	ubiquitin-protein ligase activity	15	0.021	GO:0005856	C	cytoskeleton	16	0.012
GO:0019787	F	small conjugating protein ligase activity	15	0.021	GO:0044430	C	cytoskeletal part	13	0.014
GO:0016769	F	transferase activity, transferring nitrogenous groups	13	0.021	GO:0009536	C	plastid	10	0.014
GO:0050662	F	coenzyme binding	53	0.03	GO:0009507	C	chloroplast	8	0.022
GO:0004867	F	serine-type endopeptidase inhibitor activity	7	0.041	GO:0044422	C	organelle part	60	0.031

GO:0005509	F	calcium ion binding	16	0.044	GO:0044446	C	intracellular organelle part	60	0.031
GO:0016758	F	transferase activity, transferring hexosyl groups	52	0.049	GO:0044424	C	intracellular part	222	0.045

Supplementary Table S7: KEGG enrichment analysis of genes selected according to co-expression analysis (Figure 6).

KEGG terms in "Up-Trends" were enriched by genes involved in cluster 16, cluster 22, and cluster 25 of Aig in Figure 6a. KEGG terms in "Down-Trends" were enriched by genes involved in cluster 0, cluster 3, and cluster 9 of Aig in Figure 6a. KEGG terms in "Up to down-Trends" were enriched by genes involved incluster 12, cluster 14, cluster 20, and cluster 23 of Aig in Figure 6a.

Up-regulation Trends			
Pathway	KO	Number of hit genes	P-value
Phenylpropanoid biosynthesis	Ko00940	11	0.007857
Photosynthesis	Ko00195	6	0.028884
Cyanoamino acid metabolism	Ko00460	5	0.034814
Valine, leucine and isoleucine degradation	Ko00280	4	0.046753
Sesquiterpenoid and triterpenoid biosynthesis	Ko00909	3	0.031302
Regulation of autophagy	Ko04140	3	0.047362

Down-regulation Trends			
Pathway	KO	Number of hit genes	P-value
Spliceosome	Ko03040	39	4.82E-13
Ribosome	Ko03010	31	0.010844
RNA transport	Ko03013	27	4.96E-07
Protein processing in endoplasmic reticulum	Ko04141	20	0.020764
mRNA surveillance pathway	Ko03015	16	0.000535
Ribosome biogenesis in eukaryotes	Ko03008	14	0.001341
Carbon fixation in photosynthetic organisms	Ko00710	10	0.004646
Citrate cycle (TCA cycle)	Ko00020	8	0.023212
N-Glycan biosynthesis	Ko00510	7	0.010304

Up to down-regulation Trends			
Pathway	KO	Number of hit genes	P-value
Starch and sucrose metabolism	Ko00500	15	0.001122
Phenylpropanoid biosynthesis	Ko00940	13	0.010328
Flavonoid biosynthesis	Ko00941	12	0.000235
Fatty acid elongation	Ko00062	5	0.018841
Steroid biosynthesis	Ko00100	5	0.018841
alpha-Linolenic acid metabolism	Ko00592	5	0.021104
Linoleic acid metabolism	Ko00591	3	0.006136
Arachidonic acid metabolism	Ko00590	3	0.038126
Flavone and flavonol biosynthesis	Ko00944	2	0.011345

Supplementary Table S7 (cont.): KEGG enrichment analysis of genes selected according to co-expression analysis (Figure 6).
KEGG terms in "Up-Trends" were enriched by genes involved in cluster 16, cluster 22, and cluster 25 of Leu in Figure 6b. KEGG terms in "Down-Trends" were enriched by genes involved in cluster 0, cluster 3, and cluster 9 of Leu in Figure 6b. KEGG terms in "Up to down-Trends" were enriched by genes involved incluster 12, cluster 14, cluster 20, and cluster 23 of Leu in Figure 6b.

Up-regulation Trends			
Pathway	KO	Number of hit genes	P-value
Plant-pathogen interaction	Ko04626	38	4.8E-10
Protein processing in endoplasmic reticulum	Ko04141	33	0.000296
Endocytosis	Ko04144	22	0.001981
Phenylpropanoid biosynthesis	Ko00940	21	0.013743
Cysteine and methionine metabolism	Ko00270	18	0.000779
Amino sugar and nucleotide sugar metabolism	Ko00520	18	0.022276
Peroxisome	Ko04146	14	0.008144
Glycerophospholipid metabolism	Ko00564	14	0.008144
Glycerolipid metabolism	Ko00561	12	0.000671
Phenylalanine, tyrosine and tryptophan biosynthesis	Ko00400	12	0.001591
Glycine, serine and threonine metabolism	Ko00260	11	0.027825
Glyoxylate and dicarboxylate metabolism	Ko00630	11	0.033331
Arginine and proline metabolism	Ko00330	10	0.007545
Fatty acid degradation	Ko00071	9	0.004482
Terpenoid backbone biosynthesis	Ko00900	9	0.040647
alpha-Linolenic acid metabolism	Ko00592	8	0.006709
beta-Alanine metabolism	Ko00410	7	0.038269
Ascorbate and aldarate metabolism	Ko00053	7	0.043085
Nicotinate and nicotinamide metabolism	Ko00760	6	0.00105
Flavonoid biosynthesis	Ko00941	5	0.022897
Glycosphingolipid biosynthesis - globo series	Ko00603	3	0.03023

Caffeine metabolism	Ko00232	2	0.018397
Down-regulation Trends			
Pathway	KO	Number of hit genes	P-value
Plant hormone signal transduction	Ko04075	33	0.023369
Photosynthesis	Ko00195	28	8.62E-12
Carbon fixation in photosynthetic organisms	Ko00710	21	1.37E-07
Glyoxylate and dicarboxylate metabolism	Ko00630	17	0.000125
Photosynthesis - antenna proteins	Ko00196	15	7.72E-12
Porphyrin and chlorophyll metabolism	Ko00860	15	6.07E-06
Fructose and mannose metabolism	Ko00051	15	0.000168
Glycerophospholipid metabolism	Ko00564	13	0.030409
Glycine, serine and threonine metabolism	Ko00260	11	0.041491
Pentose phosphate pathway	Ko00030	10	0.015023
Ascorbate and aldarate metabolism	Ko00053	9	0.006787
Glycerolipid metabolism	Ko00561	9	0.030867
Ubiquinone and other terpenoid-quinone biosynthesis	Ko00130	8	0.008168
Carotenoid biosynthesis	Ko00906	6	0.033528
Brassinosteroid biosynthesis	Ko00905	5	0.000205
Linoleic acid metabolism	Ko00591	3	0.035657
Up to down-regulation Trends			
Pathway	KO	Number of hit genes	P-value
Ribosome biogenesis in eukaryotes	Ko03008	24	4.01E-08
Purine metabolism	Ko00230	20	0.002019
Carbon fixation in photosynthetic organisms	Ko00710	9	0.028271
Fatty acid elongation	Ko00062	7	0.005424

Circadian rhythm - plant	Ko04712	7	0.006378
Biosynthesis of unsaturated fatty acids	Ko01040	7	0.007451
Flavonoid biosynthesis	Ko00941	7	0.008549
Cutin, suberine and wax biosynthesis	Ko00073	5	0.024879
Carotenoid biosynthesis	Ko00906	5	0.033031
Photosynthesis - antenna proteins	Ko00196	4	0.046282
Flavone and flavonol biosynthesis	Ko00944	2	0.021917
